# Alkyl-to-Alkyl Palladium Migration Enables Remote C–C Bond Formation

**DOI:** 10.1021/jacs.6c12509

**Published:** 2026-08-24

**Authors:** Yangjin Kuang, Daniel Zhou, Martin Tomanik

**Affiliations:** Department of Chemistry, 5894 New York University, New York, New York 10003, United States

## Abstract

Palladium-catalyzed C–H activation reactions are typically viewed as positionally static, with the site of reactivity dictated by the location of the initial metalation event. Herein, we report a strategy that overcomes this constraint by enabling migration of a σ-alkyl palladium intermediate prior to carbon–carbon bond formation via a previously inaccessible through-space alkyl-to-alkyl palladium shift. This two-component annulation of 2-bromoanilines or 2-bromophenols with allylic bromides proceeds through the formation of an alkyl–Pd species that undergoes translocation across the carbon framework via engagement of a distal C­(sp^3^)–H bond. This process enables a cross-dehydrogenative bond formation at a site far from the point of initial catalyst installation and furnishes tricyclic heterocycle products from simple precursors. Our transformation displays a broad substrate scope across both coupling partners and exhibits excellent heteroatom compatibility. Mechanistic studies support a pathway involving reversible C–H activation and migration driven by relief of steric congestion at the palladium center. These findings establish alkyl-to-alkyl palladium migration as a viable mode of reactivity and provide a foundation for reaction design based on catalyst translocation mechanisms.

## Graphical abstract

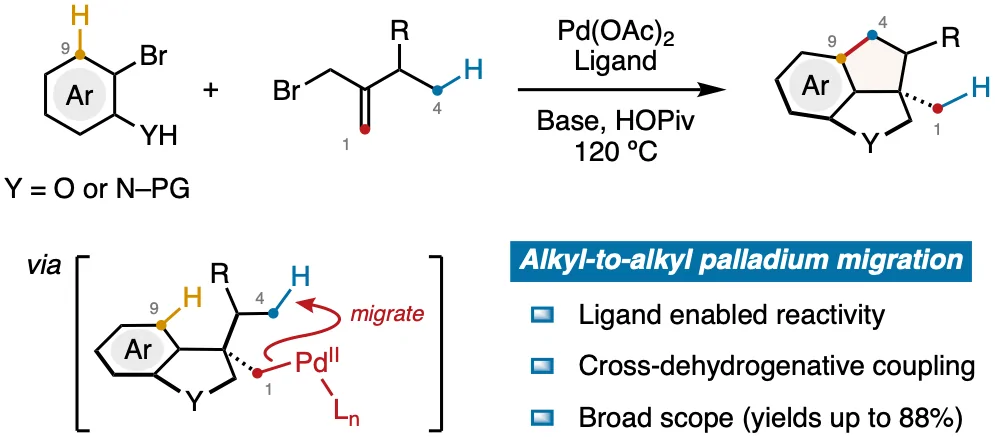

## Introduction

Palladium-catalyzed C–H activation has emerged as a powerful platform for the functionalization of organic molecules. Advances in directing group design,^1^ ligand properties,^2^ and increasingly mild reaction conditions have enabled the selective activation of strong and traditionally unreactive C–H bonds,^3^ allowing these motifs to be viewed as latent functional handles in synthesis. Despite these advances, C–H activation reactions remain largely conceptualized as positionally static transformations, where the site of functionalization is dictated by the initial formation of the carbon–palladium bond and subsequent reactivity is confined to the metalation locus (**1** → **2**, Figure 1A). Overcoming this positional constraint requires strategies that decouple the desired reactivity from the site of formation of the metalated species. One emerging solution is functional group migration,^4^ in which reactive elements are repositioned within a substrate rather than installed or removed. This type of positional reorganization of existing functionality can enable access to regions of molecular architecture that are otherwise inaccessible through conventional site-selective transformations (**5** → **6**, Figure 1B). However, despite their conceptual appeal, functional group migrations remain rare, largely owing to the challenge of orchestrating both activation and controlled reinstallation of a functional group in a single reaction manifold.^5^

**Figure 1:**
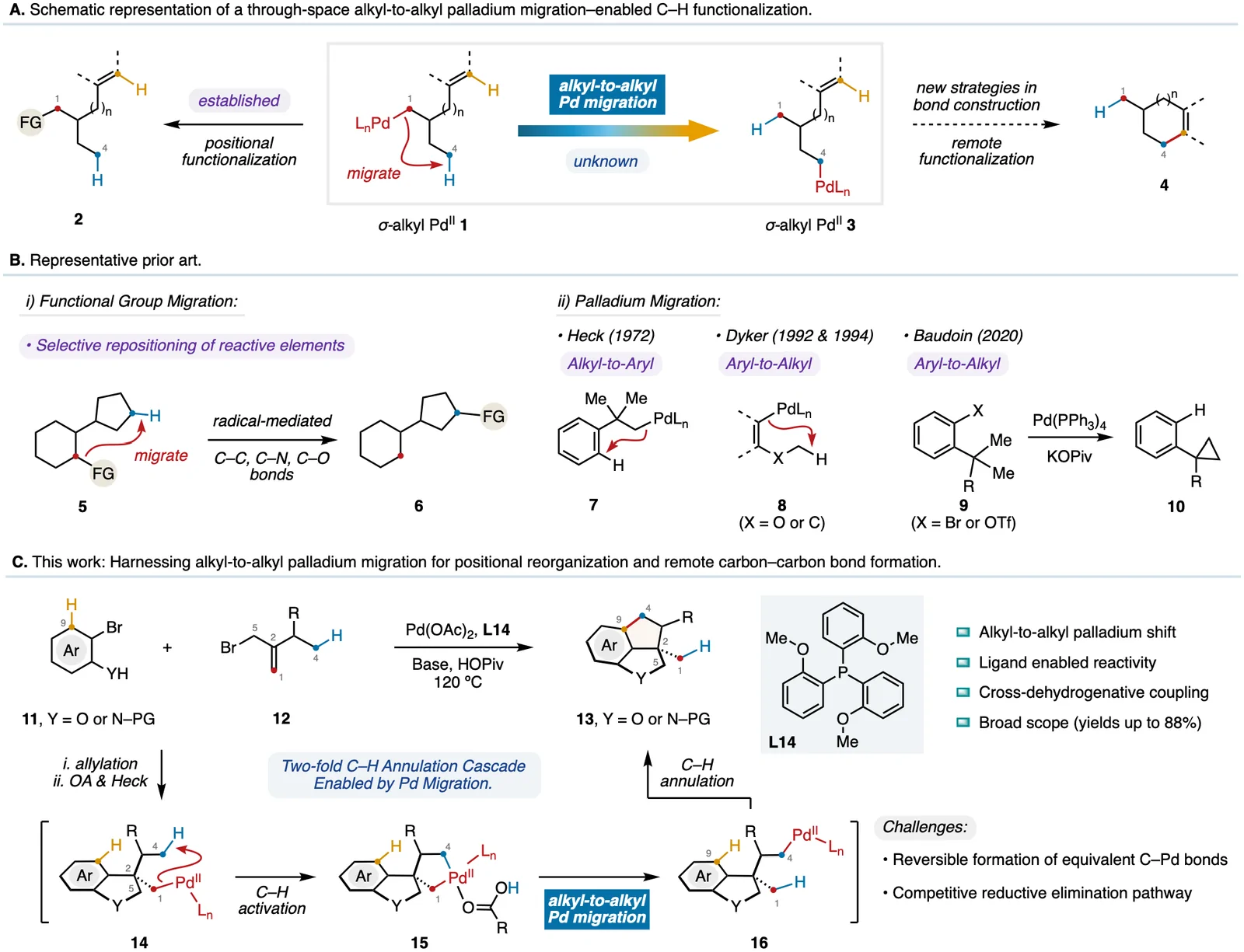
Alkyl-to-alkyl palladium migration cascade enables a remote carbon–carbon bond formation via a cross-dehydrogenative coupling.

We envisioned extending this conceptual framework to transition-metal catalysis by utilizing the σ-alkyl palladium intermediates generated in C–H activation reactions as migratable functional handles (**1** → **3**, Figure 1A). Although palladium migration has been documented in aryl–alkyl and vinyl–alkyl systems, a through-space alkyl-to-alkyl palladium migration remains elusive. Early studies by Heck demonstrated that alkyl–Pd intermediates can undergo migration to aryl positions prior to olefination (**7**, Figure 1B).^6^ Seminal works by Dyker showcased a Pd shift in the opposite direction, wherein the formation of a σ-alkyl palladium species occurs via aryl-to-alkyl migration within *ortho*-iodoanisole or *tert*-butyliodobenzene substrates (**8**).^7^ Notably, these transient and highly reactive intermediates have been leveraged for a variety of downstream functionalization reactions.^8^ In 2020, Baudoin reported an elegant single-step synthesis of cyclopropanes (**10**) via intramolecular coupling of aryl bromides or triflates.^9^ Despite these advances, controlled alkyl-to-alkyl palladium migration has not been realized, in part because the resulting alkyl palladium intermediates lack an intrinsic driving force for translocation.

Motivated by this challenge, we set out to develop a transformation that leverages C–H activation as a vehicle for σ-alkyl palladium migration. We hypothesized that engagement of a distal C–H bond could enable translocation of an initially formed alkyl–Pd intermediate, with relief of steric congestion and ligand effects providing the driving force to achieve migration across nearly equivalent C–H bonds. In this reaction manifold, an in situ generated σ-alkyl palladium intermediate undergoes an orchestrated cascade involving an alkyl-to-alkyl shift followed by C­(sp^2^)–H annulation where site selectivity is established dynamically through catalyst motion rather than fixed by the initial metalation event (**14** → **13**, Figure 1C). Crucially, the translocation of the alkyl–Pd intermediate enables a distal carbon–carbon bond formation via cross dehydrogenative coupling^10^ at a position that is far remote from the point of the initial Pd-alkyl generation.

Herein, we report successful realization of a palladium-catalyzed two-component annulation between 2-bromoanilines or 2-bromophenols (**11**) and substituted allylic bromides (**12**), enabled by an unprecedented alkyl-to-alkyl palladium migration. This cascade transformation furnishes tricyclic heterocycles from simple, acyclic precursors and demonstrates the utility of a through-space σ-alkyl palladium translocation as a strategy for functional group migration. The reaction cascade exhibits broad scope across both aryl and alkyl halide coupling partners, delivering fused indoline- and dihydrobenzofuran-containing scaffolds in high yields. Importantly, reorganization of the key alkyl–Pd intermediate enables downstream carbon–carbon bond formation at a position distal to its point of origin, establishing alkyl-to-alkyl palladium migration as a compelling disconnection strategy for the construction of pertinent, three-dimensional frameworks.

## Results and Discussion

In our reaction design, we envisioned a transformation in which the formation of a σ-alkyl palladium intermediate creates an opportunity for positional reorganization prior to bond formation (Figure 1C). Following a base-mediated allylation, oxidative addition, and regioselective carbopalladation, the resulting σ-alkyl palladium species **14** is positioned to engage a distal C­(sp^3^)–H bond and enable the alkyl-to-alkyl palladium migration event. Specifically, site-selective C­(sp^3^)–H activation of the C4 position would provide the five-membered palladacycle **15** that through protonation of the original C1 site of metalation (**15** → **16**), liberates the palladium catalyst and reestablishes it at the remote C4 position, four carbons away (highlighted blue). We postulate that relief of local steric congestion around the palladium center may be one of the key factors favoring this migration. The initially formed alkyl–Pd intermediate **14** is neopentyl-like and positioned in close proximity to the sterically demanding indoline framework. Moreover, migration repositions the alkyl–Pd species in close proximity to the C9 hydrogen, enabling rapid C­(sp^2^)–H activation and annulation. This downstream reactivity may serve as a productive trap for the migrated alkyl–Pd intermediate, thereby rendering the overall migration/annulation sequence favorable. However, our reaction design presents several challenges that could compromise the feasibility of the proposed migration. First, competitive reductive elimination from palladacycle **15** could generate an undesired spirocyclic cyclobutane.^11^ Second, achieving site-selective C­(sp^3^)–H activation at C4 over the proximal C5 position represents a significant selectivity challenge. Additionally, we anticipate that the formation of the palladacycle **15** may be reversible, as the two carbon–palladium bonds are expected to have comparable bond strengths, potentially complicating the product outcome. More broadly, these challenges are further amplified in an intermolecular context, where off-cycle reaction pathways and reduced control over generation of the key σ-alkyl palladium intermediate can erode productive reactivity.

With these considerations in mind, we initiated our investigation using tosyl-protected 2-bromoaniline **11a** and the *tert*-butyl-substituted allylic bromide **12a**. After considerable experimentation, we first observed the formation of the desired, fully annulated product **13a** in 18% when employing the combination of a monodentate tris­(*m*-methoxyphenyl)­phosphine ligand with a dual base system of cesium hydrogen carbonate and potassium pivalate in acetonitrile (Entry 1, Table 1). Analysis of the crude reaction mixture revealed significant quantities of unreacted starting materials, suggesting that the initial alkylation step was not proceeding efficiently. We found that employing the more basic rubidium carbonate resulted in complete consumption of the starting materials and improvement of the reaction yield to 27% (Entry 5, Table 1). We next investigated the influence of solvent on our cascade and found that noncoordinating aromatic solvents toluene (38%) and *p*-xylene (42%) promoted the desired transformation (Entries 9 and 10). The pivalate additive proved essential to the reaction, as its absence significantly diminished the reaction yield (10%). Furthermore, substituting potassium pivalate with pivalic acid further increased product formation (49%, Entry 13). The precise role of this bulky additive in our reaction manifold is difficult to discern, but this result is consistent with the findings by Larock^12^ and Baudoin,^9^ who employed pivalate additives in their aryl-to-alkyl migration reactions. Notably, in Entries 1–14, we consistently detected substantial quantities (up to 42% yield) of the unwanted cyclobutane byproduct arising from competitive reductive elimination from palladacycle **15**. To address this competing reactivity, our subsequent optimization focused on interrogating ligand electronics and steric effects to further drive product formation. An extensive ligand survey revealed that nitrogen-based ligands were ineffective in our cascade reaction (see **L1**, **L2**, and the Supporting Information). Only bulky, monodentate phosphine ligands generated sufficient quantities of the alkyl-to-alkyl migration product **13a**. Guided by this finding, we systematically evaluated the electronic and steric substituents of the ligand aromatic backbone and identified that the *ortho*-methoxy-substituted ligand **L14** furnished the desired fully annulated product in an excellent 86% yield. Notably, **L14** delivered a clean reaction profile where the cyclobutane side product was detected only in 4% yield. Attempts to further increase the steric environment around the ligand by substituting both *ortho* positions resulted in a markedly diminished yield (**L15**, 21%). Remarkably, replacing the *ortho*-methoxy with an *ortho*-methyl (**L13**) substituent completely suppressed the desired reactivity, underscoring the uniquely effective nature of ligand **L14** in enabling this alkyl-to-alkyl palladium migration cascade to give product **13a**. While not definitive, our optimization results suggest that the spatial placement of the methoxy substituent is critical for influencing the catalytic cycle, either through a weak secondary coordination interaction or by providing the requisite conformational organization of the ligand environment.

**Table 1:**
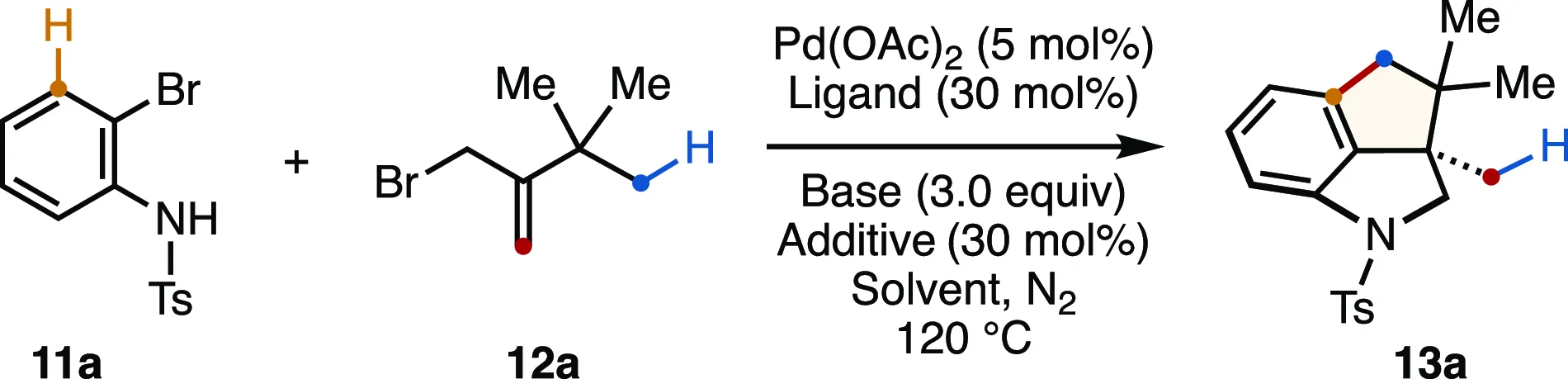

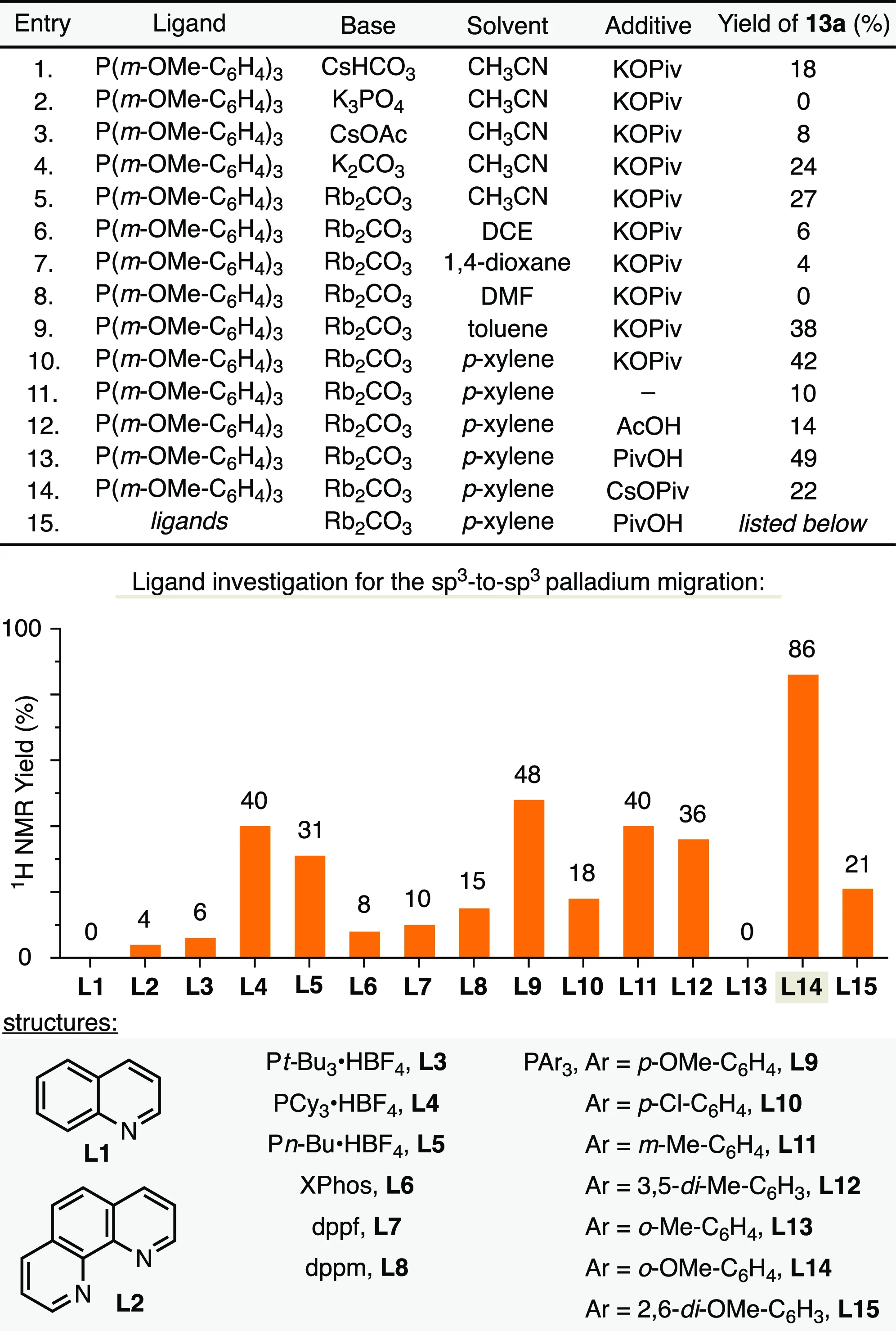
Discovery and Optimization of the Reaction Platform^a^ ^,^ ^b^

a Conditions: **11a** (0.10 mmol), **12a** (0.13 mmol), Pd­(OAc)_2_ (5 mol %), ligand (30 mol %), base (3.0 equiv), additive (30 mol %), solvent (1.0 mL), N_2_, 120 °C, 12 h.

b Yields were determined by ^1^H NMR analysis using CH_2_Br_2_ as an internal standard.

With the optimized conditions in hand, we first investigated the substrate scope of this reaction with respect to the aromatic 2-bromoaniline substrate (Scheme 1A). For example, we found that a variety of small and large alkyl substituents (**13a**–**13h**) including the bicyclic substrates **13h** were well tolerated in our reaction and gave the desired fully annulated heterocycle products in yields ranging from 50% to 86%. It is worth noting that the alkyl substituents could be positioned at either the *ortho*-, *meta*-, or *para*- positions of the aniline substrate without any influence on the reaction outcome. Next, we surveyed several electron-rich substrates. We found that the methoxy-substituted **13i** and the catechol **13j** were all well tolerated, giving products in 66 and 72% yield. Using a simple alkylation protocol (see the Supporting Information), the C4 hydroxy group within **11** could be elaborated to the cyclopropane (**13k**), azetidine (**13l**), and double Boc-protected alkyl amine (**13m**) substrates, which all gave the corresponding annulated products in 74%, 76%, and 73% yields, respectively. Substrate **13n** possessing a pendant benzoyl-protected oxygen was also tolerated; however, the desired product was detected in a decreased yield (45%). We attribute this result to the considerably increased steric environment around the nucleophilic nitrogen atom and the possibility of forming an unwanted chelate between the palladium catalyst and the chelating nitrogen and oxygen heteroatoms. We also evaluated substrates bearing electron-withdrawing groups such as the fluorine (**13o**), methyl ester (**13p**), and amide (**13q**) and isolated the desired products in good yields (56–88%).

**Scheme 1:**
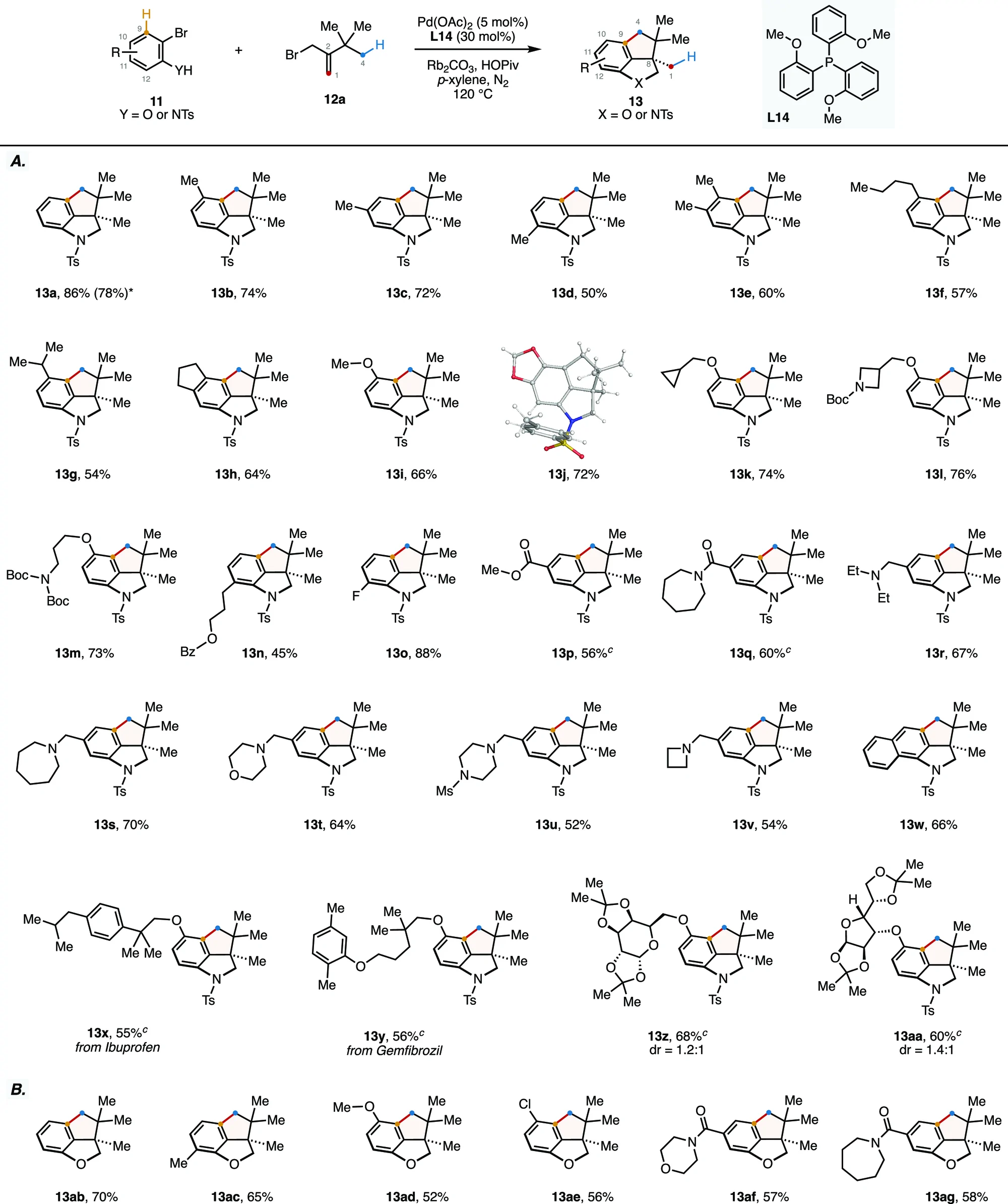
Substrate Scope for the Alkyl-to-Alkyl Palladium Migration Enabled C–H Annulation^a^ ^,^ ^b^ ^,^ ^c^

a Conditions: **11** (0.20 mmol), **12a** (0.26 mmol), Pd­(OAc)_2_ (5 mol%), tris­(o-methoxyphenyl)­phosphine (**L14**, 30 mol%), Rb_2_CO_3_ (3.0 equiv), HOPiv (30 mol%), *p*-xylene (2.0 mL), N_2_, 120 °C, 12 h.
b Isolated yields are reported.
c 36 h reaction time.
* Isolated yield on 1.0 mmol scale.

Next, we synthesized several cyclic and acyclic trialkyl amine-containing substrates, such as diethyl (**13r**), azepane (**13s**), morpholine (**13t**), piperazine (**13u**), and azetidine (**13v**). These substrates all afforded the annulated products in moderate to good yields (52–70%). Our reaction was also applicable to substrates appended with complex molecular scaffolds such as **13x** derived from the anti-inflammatory drug Ibuprofen, and **13y** that contains the backbone of the oral therapeutic Gemfibrozil, which is used to treat high cholesterol. Both of these substrates were successfully annulated in 55 and 56% yield. Similarly, the galactose (**13z**) and glucose (**13aa**) decorated starting materials gave the desired products in 68 and 60% yield, respectively. Additionally, we evaluated if our transformation could employ *ortho*-bromophenol precursors, which could provide access to oxygen-containing heterocyclic products. We were pleased to find that exposure of 2-bromophenol to our standard reaction conditions gave the desired dihydrobenzofuran-containing product **13ab** in 70% yield (Scheme 1B). With this result in hand, we examined five additional bromophenol substrates with varying electronic substituents (**13ac**–**13ag**) and obtained the desired products in 52–65% yield. Additionally, we investigated the efficiency of our transformation on a 1.0 mmol scale and isolated the desired product **13a** in 78% yield. For a survey of additional substrates that displayed only limited reactivity or were unsuccessful, see the Supporting Information.

To further explore the scope of our new reaction, we investigated the substitution on the allylic bromide coupling partner (Scheme 2A). We found that trialkyl-substituted bromide **12** gave the desired product **13ah** in high yield (70%) as a 1.4:1 mixture of diastereomers at the C9 position. The reaction could also tolerate incorporation of small ring systems at the C9 position, such as the cyclopropane **13ai** and oxetane **13aj** that provided the indoline heterocycles in 61 and 73% yield, respectively. The pendant oxygen could also be elaborated with common protecting groups such as the benzoyl (**13ak**) and pivaloyl (**13al**), furnishing products in 82% and 61% yield. Finally, our reaction was also compatible with a phthalimide-protected nitrogen heteroatom on the allylic bromide partner. We detected the desired product **13am** in 71% yield as a 2.3:1 mixture of diastereomers. Allylic halide coupling partners lacking a tertiary carbon center at the C3 position of **12** did not provide the desired products in the reaction protocol. Next, we explored the different electronic environments of the sulfonyl group on the bromoaniline **11** (Scheme 2B) and its impact on our reaction. Various substituents were found to be well tolerated, such as the methoxy (**13an**), cyclohexyl (**13ao**), naphthalene (**13ap**), or benzyl (**13aq**), all of which gave the desired products in moderate to high yields (51–82% yield). The sterically smaller mesylate (**13ar**) gave the indoline product in excellent yield (85%). Finally, the reaction could also tolerate the introduction of a morpholine heterocycle, giving product **13as** in 52% yield.

**Scheme 2:**
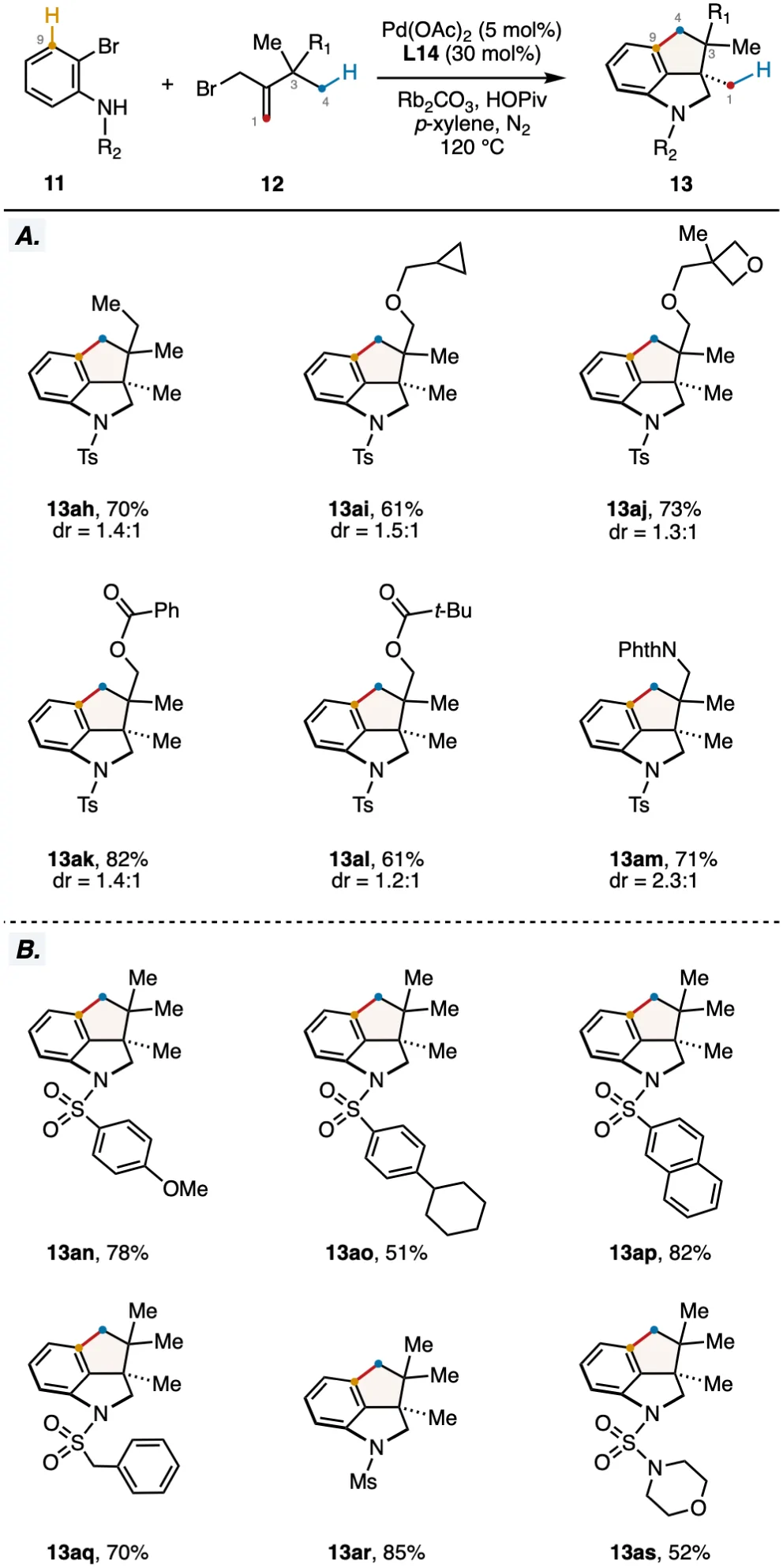
Substrate Scope of the Allylic Bromide Coupling Partner and the *N*–Sulfonyl Group[Fn sch2-fn5] [Fn sch2-fn6]

a Conditions: **11** (0.20 mmol), **12** (0.26 mmol), Pd­(OAc)_2_ (5 mol %), tris­(*o*-methoxyphenyl)­phosphine (**L14**, 30 mol %), Rb_2_CO_3_ (3.0 equiv), HOPiv (30 mol %), *p*-xylene (2.0 mL), N_2_, 120 °C, 12 h.
b Isolated yields are reported.

To showcase the diverse chemical space that is now easily accessible with our new σ-alkyl palladium migration as a strategy, we subjected several of our C–H annulation products to additional transformations, taking advantage of the functional group handles present in their scaffolds (Scheme 3A). First, we exposed carboxylic acid **17** to a palladium-catalyzed C–H hydroxylation reaction.^13^ Employing hydrogen peroxide in combination with Yu’s CarboxPyridone ligand **L16** and cesium acetate at 60 °C provided the C10-hydroxylated product **18** as an exclusive regioisomer in 64% yield. Next, we performed a nitrogen atom deletion reaction of the primary amine residue within **19**. To this end, we found that exposure of **19** to Levin’s *N*-pivaloyloxy-*N*-alkoxyamide reagent **21** ^14^ afforded the desired nitrogen-deleted product **20** in 55% yield. Given that our developed transformation readily accommodates aromatic halides, we next leveraged this functional group handle for C–N cross-coupling elaboration of the heterocyclic scaffold.^15^ Coupling between the chloride **13ae** and the secondary piperidine–morpholine amine **23**, a motif found in the oral therapeutic Alecensa used to treat non–small-cell lung cancer, furnished the aminated product **22** in 92% yield. Additionally, the sulfonamide protecting group could be readily cleaved from **13s** by treatment with methanesulfonic acid in a mixture of trifluoroacetic acid and thioanisole.^16^ Under these conditions, we obtained the unprotected indoline product **24** in 80% yield.

**Scheme 3:**
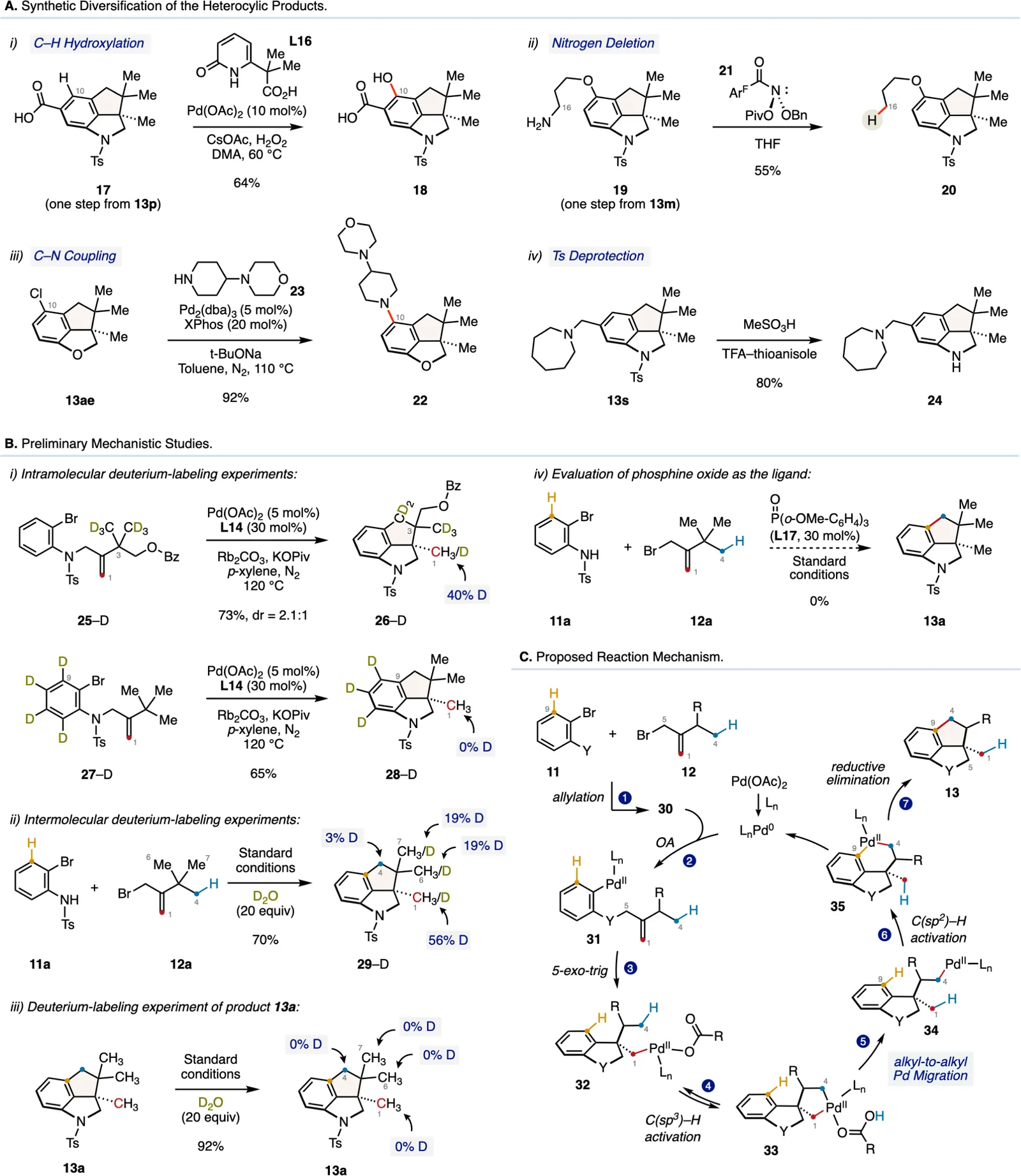
Heterocycle Diversification, Mechanistic Studies, and Proposed Catalytic Cycle[Fn sch3-fn7] [Fn sch3-fn8] [Fn sch3-fn9]

a Conditions: *i*) Pd­(OAc)_2_ (10 mol %), **L16** (10 mol %), CsOAc (1.5 equiv), H_2_O_2_ (3.5 equiv), DMA, 60 °C, 24 h. *ii*) **21** (1.2 equiv), THF, r.t., 1 h. *iii*) Pd_2_(dba)_3_ (5 mol %), XPhos (20 mol %), **23** (2.0 equiv), *t*-BuONa (2.0 equiv), toluene, 110 °C, 12 h. *iv*) MeSO_3_H (2.0 equiv), TFA–thioanisole (9:1 v/v), r.t., 12 h.
b
**25**–D or **27**–D (0.20 mmol), Pd­(OAc)_2_ (5 mol %), tris­(*o*-methoxyphenyl)­phosphine (**L14**, 30 mol %), Rb_2_CO_3_ (3.0 equiv), KOPiv (30 mol %), *p*-xylene (2.0 mL), N_2_, 120 °C, 12 h.
c Isolated yields were reported.

In order to gain more insight into the reaction mechanism, we performed several preliminary mechanistic studies to probe the reaction pathway (Scheme 3B). First, we sought to investigate the alkyl-to-alkyl palladium migration step via deuterium labeling experiments. To this end, we synthesized the two shown substrates, **25**–D and **27**–D, where the deuterium is installed either at both of the C3 methyl positions or on the aromatic ring system. We chose to perform these studies with the linear substrates to avoid potential artifacts from the initial alkylation step and limit any nonlabeled hydrogens in our reaction mixture. When we treated **25**–D with our reaction conditions, substituting pivalic acid for anhydrous potassium pivalate, we detected formation of the product **26**–D in 73% yield as a 2.1:1 mixture of diastereomers. Spectroscopic analysis of this product revealed significant labeling of the newly formed C1 methyl position (40% deuterium incorporation; see the Supporting Information for details). Conversely, exposure of **27**–D to the analogous reaction conditions gave product **28**–D without any deuterium incorporation to the C1 methyl group. These data are in good agreement with our designed reaction pathway proceeding via the alkyl-to-alkyl migration from carbon C1 to carbon C4 and refute the direct engagement of the originally formed C1 σ-alkyl palladium species **14** with the hydrogen at C9. We also performed intermolecular H/D exchange experiments by adding superstoichiometric amounts of deuterium oxide to our standard protocol (Scheme 3B). Under these conditions, product **29**–D was obtained in 70% yield, and spectroscopic analysis of this product showed deuterium labeling of the C1 methyl position (56%) and C6 and C7 methyl positions (19%). The C4 methylene position where the palladium catalyst migrates to displayed 3% deuterium incorporation. Importantly, we did not detect any deuteration when we treated product **13a** with these identical H/D exchange reaction conditions.

On the basis of these experiments, we propose that our transformation proceeds through the catalytic cycle shown in Scheme 3C. The process commences with a base-mediated allylation between **11** and **12**, followed by oxidative addition with the in situ generated palladium(0) catalyst to generate the linear intermediate **31**. Next, a regioselective 5-*exo*-trig Heck carbopalladation (**31** → **32**) provides the C1 σ-alkyl palladium species **32**. This complex undergoes a fast and reversible C­(sp^3^)–H activation at the C4 position to give the five-membered palladacycle **33**. A key regioselective protonation of **33** drives the 1,4-alkyl-to-alkyl palladium migration, generating the C4 σ-alkyl palladium species **34**. Subsequently, a second C–H activation at the C9 position forms the six-membered palladacycle **35**, which upon reductive elimination furnishes the heterocyclic product **13**. Notably, based on the H/D exchange experiments, the insertion into the C­(sp^2^)–H bond proceeds faster than the rate of equilibration between **32** and **33**, as indicated by the low levels of deuteration at the C4 position. Lastly, we confirmed that tris­(*o*-methoxyphenyl)­phosphine (**L14**)^17^ is the active ligand in this transformation, as the synthetically prepared phosphine monoxide (**L17**) did not yield any of the desired product **13a** and instead provided only the linear intermediate arising from the base-mediated allylation.

## Conclusion

In summary, we have developed a highly efficient cascade reaction that leverages an in situ generated σ-alkyl palladium species as a migratable handle for a through-space alkyl-to-alkyl shift, enabling distal carbon–carbon bond formation. This two-component manifold transforms readily available 2-bromoanilines or 2-bromophenols and simple allylic halides into complex tricyclic heterocycles through ligand-controlled C–H annulative reactivity. Mechanistically, the initially formed σ-alkyl palladium species undergoes translocation via regioselective protonation, driven by relief of steric congestion in a five-membered palladacycle, enabling remote carbon–carbon bond formation through a cross-dehydrogenative coupling process. Tris­(*o*-methoxyphenyl)­phosphine ligand proved critical in suppressing side reactions and enabling high selectivity among electronically and sterically similar C–H bonds. The reaction exhibits broad scope, delivering over 40 heterocyclic products in high yields with excellent regiocontrol. Product diversification and preliminary mechanistic studies further highlight the synthetic utility of this platform. We anticipate that this transformation will provide a foundation for developing new migratory alkyl–alkyl coupling strategies and expand the scope of palladium-catalyzed distal functionalization.

## Supplementary Material



## Notes

General information; substrate structures; experimental procedures including mechanistic studies; characterization of substrates and products; crystallographic analysis of **13j**; catalog of ^1^H NMR and ^13^C NMR spectra (PDF)
