# Supplementary material for: Alkyl-to-Alkyl Palladium Migration Enables Remote C–C Bond Formation
Source: J Am Chem Soc. 2026 Aug 24;148(35):38395–403. doi: 10.1021/jacs.6c12509 (PMC13564419; doi:10.1021/jacs.6c12509)

# Alkyl-to-Alkyl Palladium Migration Enables Remote C–C Bond Formation.

Yangjin Kuang[†,1], Daniel Zhou[†,1], and Martin Tomanik[1,*]

[1]Department of Chemistry, New York University, New York, New York, 10003, United States.

Supporting Information

**Table of Contents:**

General Information ........ S2
Substrate Structures ........ S3
Experimental Section ........ S4
- Synthetic Procedure for the Preparation of Substrates **11j** ........ S4
- General Procedure A: Synthesis of 2-Bromoaniline Sulfonamides **11k–11m** ........ S5
- Synthetic Procedure for the Preparation of Substrates **11n** ........ S7
- General Procedure B: Synthesis of Amine and Amide-Containing 2-Bromoaniline Sulfonamides **11q–11v** ........ S10
- General Procedure C: Synthesis of 2-Bromoaniline Sulfonamides **11x–11aa** ........ S14
- Synthetic procedure for the preparation of substrates 2-Bromophenol **11ag** ........ S17
- Synthetic Procedure for the Preparation of Allylic Bromides **12ai–12ak** ........ S18
- General Procedure D: Synthesis of 2-Bromoaniline sulfonamides **11an–11as** ........ S25
- Synthetic procedure for diversification of the heterocyclic products ........ S28
- Synthetic Procedure for the Preparation of Substrate **25–D** and **27–D** ........ S33
- General Procedure E: Alkyl-To-Alkyl Palladium Migration-Enabled Annulation ........ S41
- Reaction Optimization Tables ........ S42
- List of Unsuccessful or Limited Reactivity Substrates ........ S45
- Mechanistic Studies ........ S46

Characterization of Products ........ S57
Crystallographic Analysis of **13j** ........ S76
References ........ S78
Catalogue of $^{1}$H NMR and $^{13}$C NMR Spectra ........ S80

**General Information.**

Unless otherwise stated, all reagents were purchased from commercial suppliers and used without further purification. Anhydrous solvents were obtained from the solvent purification system produced by *JC* Meyer Solvent Systems. Analytical thin-layer chromatography (TLC) was performed on Merck Millipore precoated (0.25 mm thickness) silica gel plates with F254 fluorescent indicator. TLC plates were visualized by exposure to ultraviolet light (UV) and/or submersion in aqueous potassium permanganate solution ($KMnO_4$), ceric ammonium molybdate solution (CAM), *para*-anisaldehyde (PAA) and followed by brief heating on a hot plate (120 °C, 10–15 s). Flash-column chromatography was performed employing silica gel (32-63 μm particle size) supplied by Dynamic Adsorbents. Proton nuclear magnetic resonance spectra ($^1$H NMR) were recorded on a Bruker instrument (400, or 500 MHz). Chemical shifts are expressed in parts per million (ppm, δ scale) downfield from tetramethylsilane and are referenced to residual protium in the NMR solvent ($CHCl_3$, δ 7.26; $CD_3OD$, δ 3.31). Data are represented as follows: chemical shift, multiplicity (s = singlet, d = doublet, t = triplet, q = quartet, m = multiplet and/or multiple resonances, b = broad, app = apparent), coupling constant, *J*, in Hertz (Hz) AND integration. Proton-decoupled carbon nuclear magnetic resonance spectra ($^{13}$C NMR) were recorded on a Bruker instrument (101 or 126 MHz). Chemical shifts are expressed in parts per million (ppm, δ scale) downfield from tetramethylsilane and are referenced to the carbon resonances of the solvent ($CDCl_3$, δ 77.2; $CD_3OD$, δ 49.0). High-resolution mass spectra (HRMS) were recorded on an Agilent Mass spectrometer using ESI-TOF (electrospray ionization-time of flight).

## Substrate Structures.

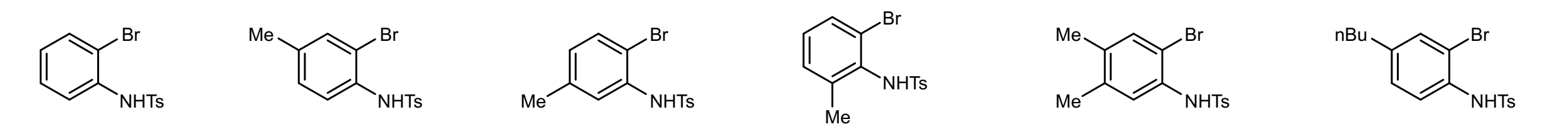

**11a** **11b** **11c** **11d** **11e** **11f**

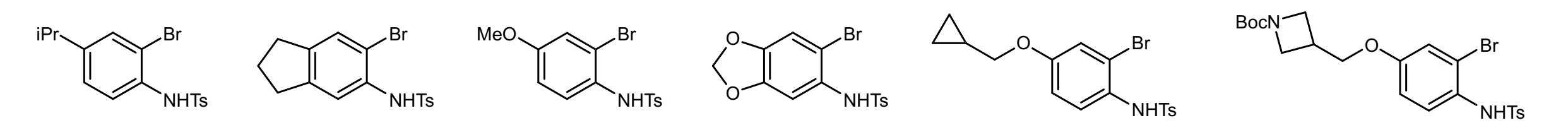

**11g** **11h** **11i** **11j** **11k** **11l**

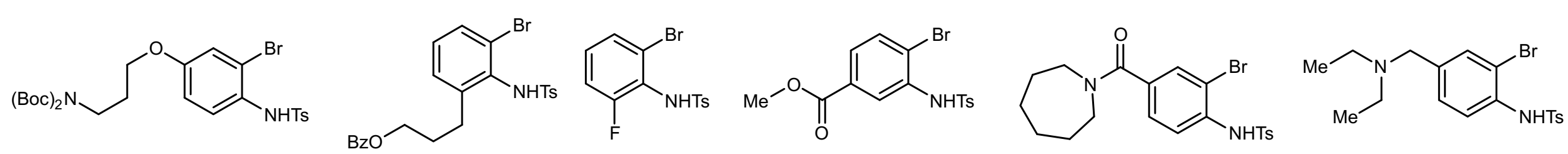

**11m** **11n** **11o** **11p** **11q** **11r**

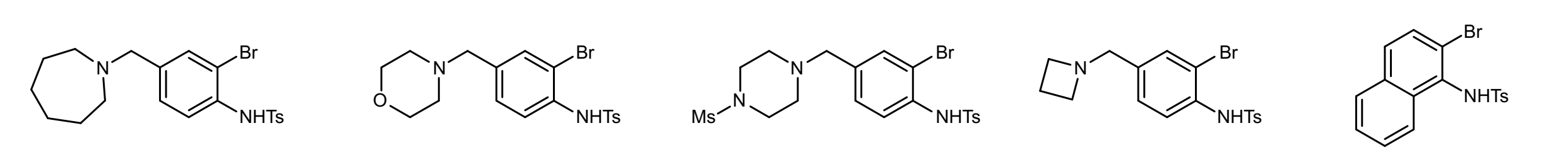

**11s** **11t** **11u** **11v** **11w**

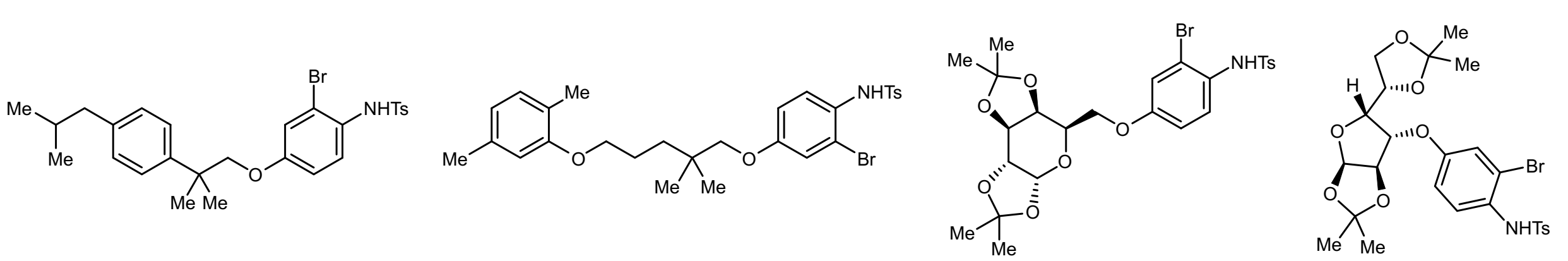

**11x** **11y** **11z** **11aa**

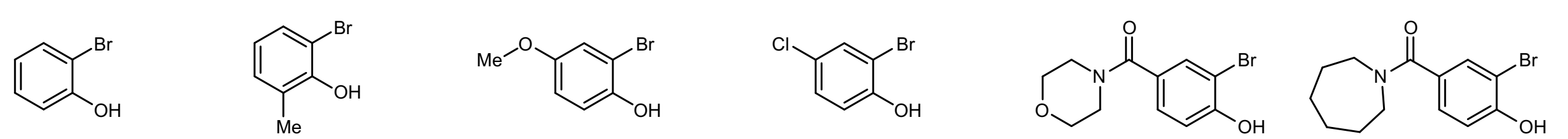

**11ab** **11ac** **11ad** **11ae** **11af** **11ag**

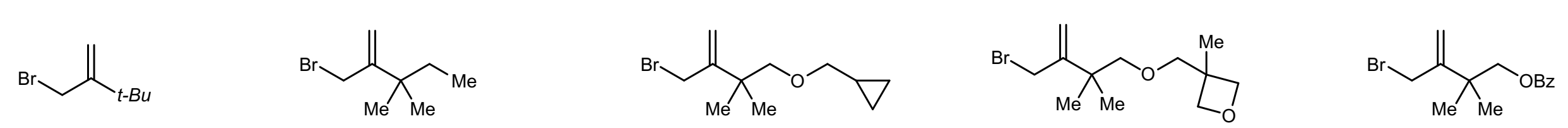

**12a** **12ah** **12ai** **12aj** **12ak**

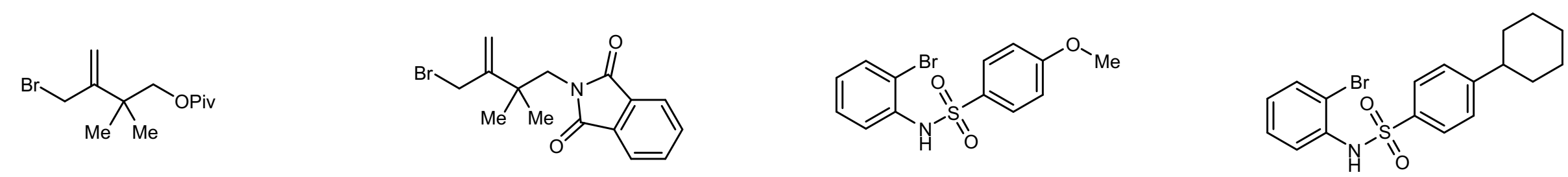

**12al** **12am** **11an** **11ao**

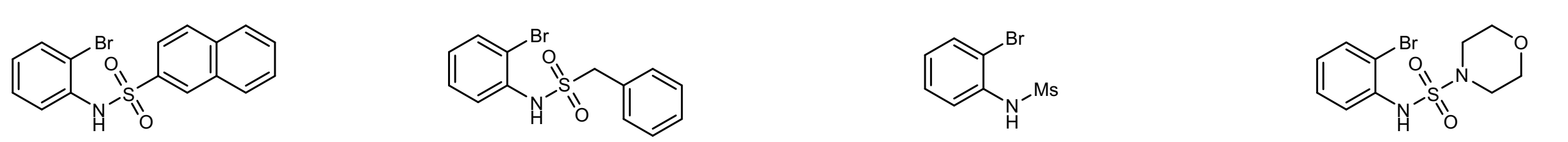

**11ap** **11aq** **11ar** **11as**

**Experimental Section.**

The following substrates: **11a–11i**, **11o**, **11p**, **11w**, **11ab–11af**, **12a**, **12ah**, **12al**, **12am** were obtained from commercial sources or were prepared according to published procedures.[1–7]

**Synthetic procedure for the preparation of substrates 11j:**

*Synthesis of 1,3-dioxolane substrate **11j** through tosyl group installation on S1:*

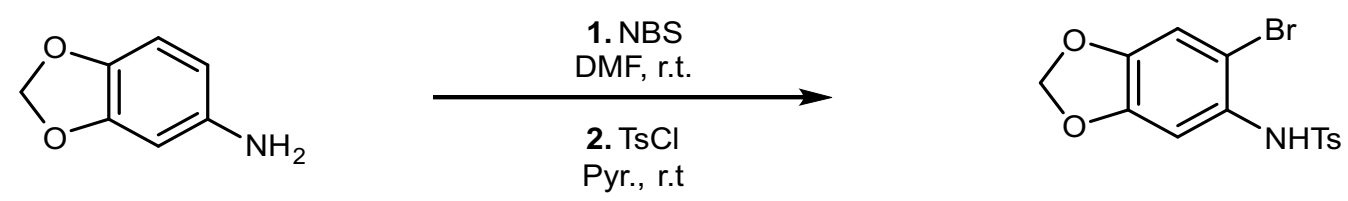

**S1** **11j**

*N*-Bromosuccinimide (649 mg, 3.65 mmol, 1.00 equiv) was added in one portion to a solution of 5-Aminoindan **S1** (500 mg, 3.65 mmol, 1.00 equiv) in dry *N,N*-Dimethylformamide (3.65 mL, 1.0M). The reaction was stirred at r.t. for 1.5 h. The product mixture was then diluted sequentially with ethyl acetate (20 mL) and water (20 mL). The resulting biphasic mixture was transferred to a separatory funnel and the layers that formed were separated. The aqueous layer was extracted with ethyl acetate (3 × 20 mL). The organic layers were combined and the combined organic layers were washed with saturated aqueous sodium chloride solution (30 mL). The washed organic layer was dried over sodium sulfate. The dried solution was filtered and the filtrate was concentrated. The residue obtained was used directly in the next step without further purification. *Para*-toluenesulfonyl chloride (730 mg, 3.83 mmol, 1.05 equiv) was added to a solution of the residue obtained from the previous step (nominally, 1.00 equiv) in pyridine (5.21 mL, 0.70M) at r.t. The reaction mixture was stirred for 16 hours. Pyridine was removed under a stream of nitrogen. The residue obtained was dissolved in dichloromethane and transferred to a separatory funnel. The organic solution was washed with 1 M hydrochloric acid (30 mL). The aqueous layer was extracted with dichloromethane (3 × 30 mL). The organic layers were combined and washed with saturated aqueous sodium chloride solution. The washed organic layer was dried over sodium sulfate. The dried solution was filtered, and the filtrate was concentrated. The residue obtained was purified by flash-column chromatography (eluting with 10% ethyl acetate–hexanes initially) to provide the corresponding substrate **11j** (972 mg, 72%)

$^{1}$H NMR (400 MHz, $CDCl_3$) δ 7.60 (d, *J* = 8.4 Hz, 2H), 7.25 – 7.18 (m, 3H), 6.81 (s, 1H), 6.66 (s, 1H), 5.98 (s, 2H), 2.39 (s, 3H). $^{13}$C NMR (101 MHz, $CDCl_3$) δ 148.1, 146.2, 144.3, 135.9, 129.8, 128.6, 127.53, 111.7, 107.8, 105.7, 102.3, 21.7. HRMS (ESI-TOF) m/z: $[M + H]^+$ calcd for $C_{14}H_{13}BrNO_4S$, 369.9743; found, 369.9758.

**General Procedure** A**: Synthesis of 2-bromoaniline sulfonamides 11k, 11l and 11m.**

*Part 1) Synthesis of the 2-bromoanilines* ***S4*** *via alkylation.*

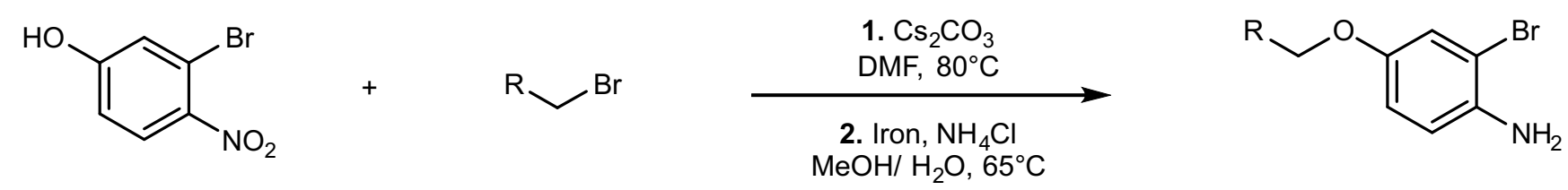

**S2** **S3** **S4**

The alkyl bromide electrophile **S3** (1.50 equiv) and cesium carbonate (2.00 equiv) were sequentially added to a solution of 3-bromo-4-nitrophenol **S2** (1.00 equiv) in *N,N*-dimethylformamide (0.50M) at r.t. The reaction mixture was stirred overnight at 80 °C. The product mixture was diluted sequentially with water and diethyl ether. The resulting biphasic mixture was transferred to a separatory funnel and the layers that formed were separated. The aqueous layer was extracted with diethyl ether. The organic layers were combined, and the combined organic layers were washed with saturated aqueous ammonium chloride solution, water and saturated aqueous sodium chloride solution. The washed organic layer was dried over sodium sulfate. The dried solution was filtered and the filtrate was concentrated. The residue obtained was used directly in the next step without any further purification. Iron powder (4.00 equiv), and ammonium chloride (6.00 equiv) was added to a solution of corresponding nitroarene (1.00 equiv) in methanol/water 2:1 (0.20M). The reaction mixture was then placed to an oil bath that had been preheated to 65 °C and stirred for 16 hours at 65 °C. The reaction mixture was cooled and then filtered through a pad of Celite, and the filtrate was concentrated under reduced pressure. The residue obtained was diluted sequentially with water and dichloromethane. The aqueous layer was extracted with dichloromethane. The organic layers were combined. The combined organic layer was dried over sodium sulfate. The dried solution was filtered and the filtrate was concentrated. The amine residue **S4** obtained was used directly in the next step without any further purification.

*Part 2) Synthesis of the substrate* **11k, 11l and 11m** *through tosyl group installation on* ***S4***:

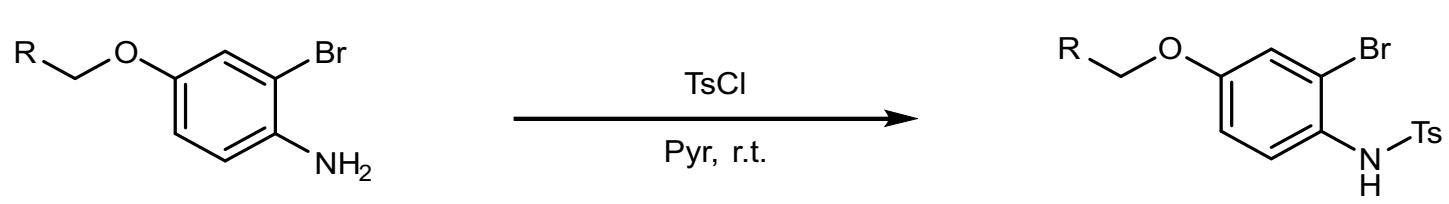

**S4** **11k, 11l and 11m.**

*Para*-toluenesulfonyl chloride (1.05 equiv) was added to a solution of the corresponding aniline intermediate **S4** (1.00 equiv) in pyridine (0.70 M) at r.t. The reaction mixture was stirred for 16 hours. Pyridine was removed under a stream of nitrogen. The residue obtained was dissolved in dichloromethane and transferred to a separatory funnel. The organic solution was washed with 1M hydrochloric acid. The aqueous layer was extracted with dichloromethane. The organic layers were combined and washed with saturated aqueous sodium chloride solution. The washed organic layer was dried over sodium sulfate. The dried solution was filtered, and the filtrate was concentrated. The residue obtained was purified by flash-column chromatography (eluting with 10% ethyl acetate–hexanes initially) to provide the corresponding aryl sulfonamide substrates **11k**, **11l** and **11m**.

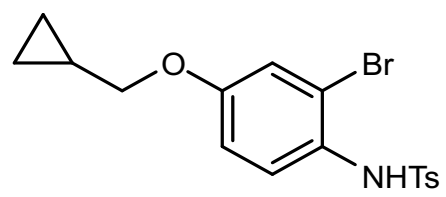

**11k**

N-(2-bromo-4-(cyclopropylmethoxy)phenyl)-4-methylbenzenesulfonamide (**11k**). Prepared according to General Procedure A. Using 3-bromo-4-nitrophenol (520 mg, 2.39 mmol) afforded the final product (392 mg, 0.99 mmol, 42% over three steps). $^1$H NMR (400 MHz, $CDCl_3$) δ 7.61 – 7.52 (m, 3H), 7.19 (d, *J* = 8.0 Hz, 2H), 6.93 (d, *J* = 2.8 Hz, 1H), 6.84 (dd, *J* = 9.0, 2.8 Hz, 1H), 6.60 (s, 1H), 3.73 (d, *J* = 6.9 Hz, 2H), 2.38 (s, 3H), 1.24 – 1.17 (m, 1H), 0.67 – 0.60 (m, 2H), 0.35 – 0.27 (m, 2H). $^{13}$C NMR (101 MHz, $CDCl_3$) δ 157.4, 144.1, 136.0, 129.7, 127.6, 127.5, 125.9, 118.4, 118.2, 115.0, 73.4, 21.7, 10.2, 3.3. HRMS (ESI-TOF) m/z: $[M + H]^+$ calcd for $C_{17}H_{19}BrNO_3S$, 396.0264; found, 396.0276.

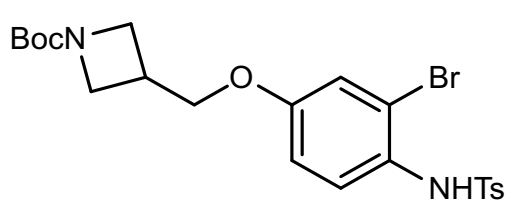

**11l**

Tert-butyl 3-((3-bromo-4-((4-methylphenyl)sulfonamido)phenoxy)methyl)azetidine-1-carboxylate **(11l).** Prepared according to General Procedure A. Using 3-bromo-4-nitrophenol (436 mg, 2.00 mmol) afforded the final product (363 mg, 0.71 mmol, 36% over three steps). $^1$H NMR (400 MHz, $CDCl_3$) δ 7.63 – 7.53 (m, 3H), 7.20 (d, *J* = 8.1 Hz, 2H), 6.95 (d, *J* = 2.8 Hz, 1H), 6.84 (dd, *J* = 8.9, 2.8 Hz, 1H), 6.62 (s, 1H), 4.09 – 4.03 (m, 2H), 4.01 (d, *J* = 6.6 Hz, 2H), 3.80 – 3.70 (m, 2H), 3.00 – 2.84 (m, 1H), 2.39 (s, 3H), 1.44 (s, 9H). $^{13}$C NMR (101 MHz, $CDCl_3$) δ 157.0, 156.5, 144.21, 136.0, 129.7, 128.1, 127.5, 125.9, 118.4, 118.1, 114.9, 79.7, 70.0, 51.7, 28.5, 28.3, 21.8. HRMS (ESI-TOF) m/z: $[M + H]^+$ calcd for $C_{22}H_{28}BrN_2O_5S$, 511.0897; found, 511.0914.

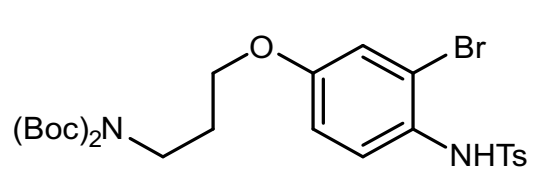

**11m**

Tert-butyl (3-(3-bromo-4-((4-methylphenyl)sulfonamido)phenoxy)propyl)(tert-butoxycarbonyl)carbamate **(11m).** Prepared according to General Procedure A. Using 3-bromo-4-nitrophenol (872 mg, 4.00 mmol) afforded the final product (2.10 g 3.50 mmol, 66% over three steps). $^1$H NMR (400 MHz, $CDCl_3$) δ 7.57 – 7.47 (m, 3H), 7.15 (d, *J* = 8.1 Hz, 2H), 6.88 (d, *J* = 2.8 Hz, 1H), 6.78 (dd, *J* = 8.9, 2.8 Hz, 1H), 6.68 (s, 1H), 3.88 (t, *J* = 6.1 Hz, 2H), 3.72 (t, *J* = 6.9 Hz, 2H), 2.34 (s, 3H), 2.04 – 1.94 (m, 2H), 1.44 (s, 18H). $^{13}$C NMR (101 MHz, $CDCl_3$) δ 157.2, 152.6, 144.0, 135.9, 129.5, 127.5, 127.4, 125.9, 118.2, 118.2, 114.7, 82.4, 66.2, 43.6, 28.7, 28.1, 21.6. HRMS (ESI-TOF) m/z: $[M + H]^+$ calcd for $C_{26}H_{36}BrN_2O_7S$, 599.1421; found, 599.1440.

**Synthetic Procedure for the Preparation of the Substrates 11n:**

*Part 1. Synthesis of α,β-unsaturated ester **S7** via palladium-catalyzed cross-coupling:*

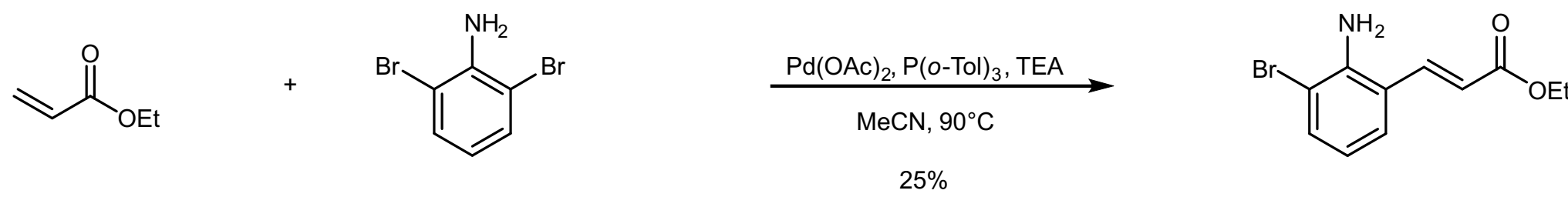


S5 S6 S7

Palladium (II) acetate (168 mg, 0.75 mmol, 5.00 mol%) and tri(*o*-tolyl)phosphine (931 mg, 3.00 mmol, 20.0 mol%) were added to a 100 mL two-neck round-bottom flask equipped with a condenser and septum containing 2,5-dibromoaniline **S6** (3.74 g, 15.0 mmol, 1.00 equiv). The flask was evacuated and backfilled with nitrogen three times. Acetonitrile (30 mL, 0.50M) was added under nitrogen, followed by ethyl acrylate **S5** (1.60 mL, 15.0 mmol, 1.00 equiv) and triethylamine (12.5 mL, 90 mmol, 6.00 equiv). The reaction mixture was placed in a preheated 90 °C oil bath and stirred for 14 h. The reaction mixture was concentrated under reduced pressure. The residue was dissolved in dichloromethane (30 mL) and diluted with saturated aqueous ammonium chloride solution (30 mL). The resulting biphasic mixture was transferred to a separatory funnel and the layers that formed were separated. The aqueous layer was extracted with dichloromethane (3 × 30 mL). The organic layers were combined and dried over sodium sulfate. The dried solution was filtered, and the filtrate was concentrated under reduced pressure. The residue obtained was purified by flash-column chromatography (eluting with 80% dichloromethane–hexanes initially) to afford the crude *α,β*-unsaturated ester **S7** as amorphous solids (1.01 g, 25% yield).

For a spectroscopically pure sample, the *α,β*-unsaturated ester **S7** could be recrystallized from a mixture dichloromethane–hexanes (1:20 v/v) at −20 °C.

$^{1}$H NMR (400 MHz, $CDCl_3$) δ 7.78 (d, *J* = 15.8 Hz, 1H), 7.44 (dd, *J* = 7.9, 1.5 Hz, 1H), 7.31 (dd, *J* = 7.7, 1.4 Hz, 1H), 6.63 (t, *J* = 7.8 Hz, 1H), 6.35 (d, *J* = 15.8 Hz, 1H), 4.29 (s, 2H), 4.26 (q, *J* = 7.1 Hz, 2H), 1.33 (t, *J* = 7.1 Hz, 3H). $^{13}$C NMR (101 MHz, $CDCl_3$) δ 167.0, 142.9, 139.8, 134.3, 127.4, 121.4, 120.0, 119.3, 111.0, 60.8, 14.5. HRMS (ESI-TOF) m/z: $[M + H]^+$ calcd for $C_{11}H_{13}BrNO_2$, 270.0124; found, 270.0138.

*Part 2) Synthesis of the ester **S8** via a 1,4-reduction and tosylation of α,β-unsaturated Ester **S7**:*

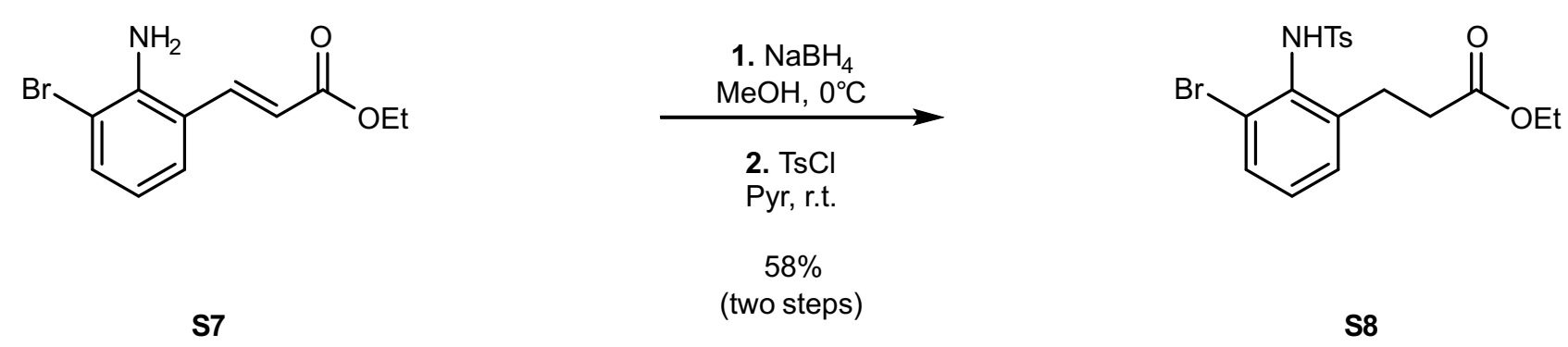

Sodium borohydride (441 mg, 11.7 mmol, 5.0 equiv) was added in one portion to a solution of *α,β*-unsaturated ester **S7** (629 mg, 2.33 mmol, 1.00 equiv) in methanol (46.6 mL, 0.05M) at 0 °C. The reaction mixture was stirred at 0 °C for 15 min. The reaction mixture was diluted sequentially with water (50 mL) and dichloromethane (50 mL) and quenched with 1M hydrochloric acid (10 mL). The resulting biphasic mixture was transferred to a separatory funnel and the layers that formed were separated. The aqueous layer was extracted with dichloromethane (3 × 50 mL). The organic layers were combined and dried over sodium sulfate. The dried solution was filtered, and the filtrate was concentrated under reduced pressure. The residue obtained was used directly in the next step without further purification.

*Para*-Toluenesulfonyl chloride (449 mg, 2.36 mmol, 1.01 equiv) was added to the residue obtained in the previous step (nominally, 1.0 equiv) in pyridine (3.33 mL, 0.70 M) at r.t. The reaction mixture was stirred for 16 hours at r.t. The pyridine was then removed via a steady stream of nitrogen passing through the reaction mixture. The mixture was then diluted sequentially with 1M hydrochloric acid (20 mL), and dichloromethane (20 mL). The resulting biphasic mixture was transferred to a separatory funnel and the layers that formed were separated. The aqueous layer was extracted with dichloromethane (3 × 20 mL). The organic layers were combined and dried over sodium sulfate. The dried solution was filtered and the filtrate was concentrated. The residue obtained was purified by flash-column chromatography eluting with (20% ethyl acetate–hexanes initially) to provide the corresponding sulfonamide **S8** as amorphous solids (707 mg, 57% yield over two steps).

$^1$H NMR (400 MHz, $CDCl_3$) δ 7.59 (d, 2H), 7.34 (dd, *J* = 7.9, 1.5 Hz, 1H), 7.25 – 7.19 (m, 3H), 7.07 (t, *J* = 7.8 Hz, 1H), 6.75 (s, 1H), 4.09 (q, *J* = 7.1 Hz, 2H), 3.20 (t, *J* = 7.2 Hz, 2H), 2.65 (t, *J* = 7.2 Hz, 2H), 2.42 (s, 3H), 1.21 (t, *J* = 7.1 Hz, 3H). $^{13}$C NMR (101 MHz, $CDCl_3$) δ 173.3, 144.1, 143.0, 137.1, 133.1, 131.4, 129.8, 129.6, 129.1, 127.9, 124.4, 60.8, 35.1, 27.6, 21.8, 14.3. HRMS (ESI-TOF) m/z: $[M + H]^+$ calcd for $C_{18}H_{21}BrNO_4S$, 426.0369; found, 426.0379.

*Part 3) Synthesis of the substrate **11n** via a reduction and benzoyl protection sequence of **S8**:*

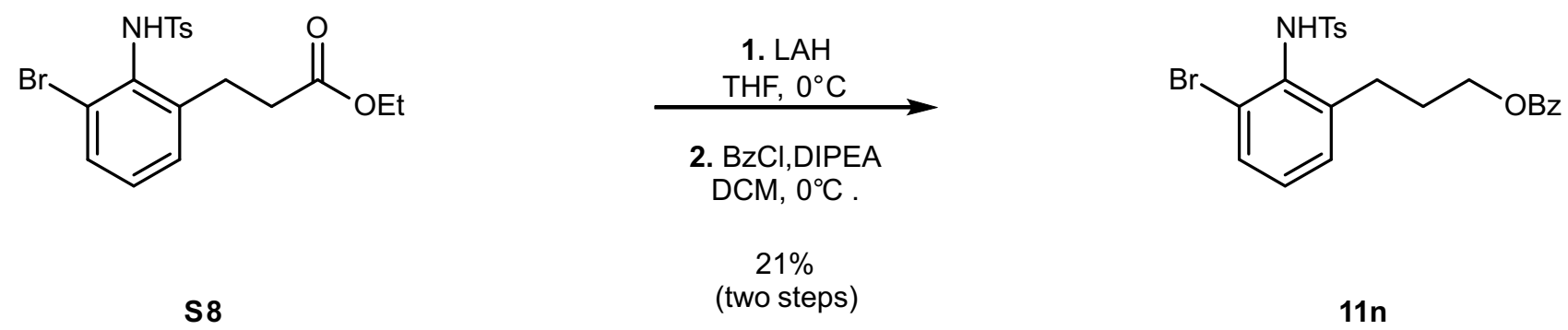


**S8** **11n**

Lithium aluminum hydride (1.64 mL, 3.28 mmol, 2.00 equiv, 2.0 M in tetrahydrofuran) was added dropwise to a solution of the ester **S8** (700 mg, 1.64 mmol, 1.00 equiv) in tetrahydrofuran (16.5 mL, 0.10M) at 0 °C under a nitrogen atmosphere. The reaction mixture was stirred at 0 °C for 30 min. The cold product mixture was then carefully quenched by a protocol developed by Fieser.[8] Water (124 µL), 5M aqueous NaOH (124 µL), and water (372 µL) were added sequentially and dropwise to the reaction vessel. The mixture was stirred for 5 min, anhydrous magnesium sulfate was added. The resulting suspension was filtered through a pad of Celite, washing with diethyl ether. The filtrate was concentrated under reduced pressure to afford the corresponding alcohol intermediate, which was used directly in the next step without any further purification.

Benzoyl chloride (190 µL, 1.64 mmol, 1.00 equiv) was added dropwise to a solution of the residue obtained in the previous step (nominally, 1.00 equiv) and *N,N*-diisopropylethylamine (600 µL, 3.44 mmol, 2.10 equiv) dissolved in dichloromethane (16.4 mL, 0.10M) at 0 °C. The reaction mixture was stirred at 0 °C for 30 min. The product mixture was diluted sequentially with saturated aqueous ammonium chloride solution (20 mL). The resulting biphasic mixture was transferred to a separatory funnel and the layers that formed were separated. The aqueous layer was extracted with dichloromethane (3 × 20 mL). The organic layers were combined and dried over sodium sulfate. The dried solution was filtered and the filtrate was concentrated. The residue obtained was purified by flash-column chromatography eluting with (5% ethyl acetate–hexanes initially) to provide the corresponding benzoylated sulfonamide **11n** as amorphous solid (171 mg, 21% yield over 2 steps).

$^{1}$H NMR (400 MHz, $CDCl_3$) δ 8.07 – 8.03 (m, 2H), 7.58 – 7.51 (m, 3H), 7.47 – 7.43 (m, 2H), 7.32 – 7.28 (m, 2H), 7.22 – 7.19 (m, 2H), 7.08 (t, $J$ = 7.8 Hz, 1H), 6.24 (s, 1H), 4.31 (t, $J$ = 6.4 Hz, 2H), 3.18 (t, $J$ = 6.7 Hz, 2H), 2.41 (s, 3H), 2.16 – 2.08 (m, 2H). $^{13}$C NMR (101 MHz, $CDCl_3$) δ 166.8, 144.3, 143.92, 136.8, 133.0, 132.7, 130.9, 130.5, 130.0, 129.8, 129.7, 129.1, 128.5, 127.9, 124.0, 64.6, 29.5, 29.5, 21.8. HRMS (ESI-TOF) m/z: $[M + H]^+$ calcd for $C_{23}H_{23}BrNO_4S$, 488.0526; found, 488.0533.

**General Procedure B: Synthesis of amine- and amide-containing 2-bromoaniline sulfonamides 11q–11v:**

*Part 1) Synthesis of tosyl-protected amide-containing substrates* ***S11*** *via an amide bond formation/tosylation sequence:*

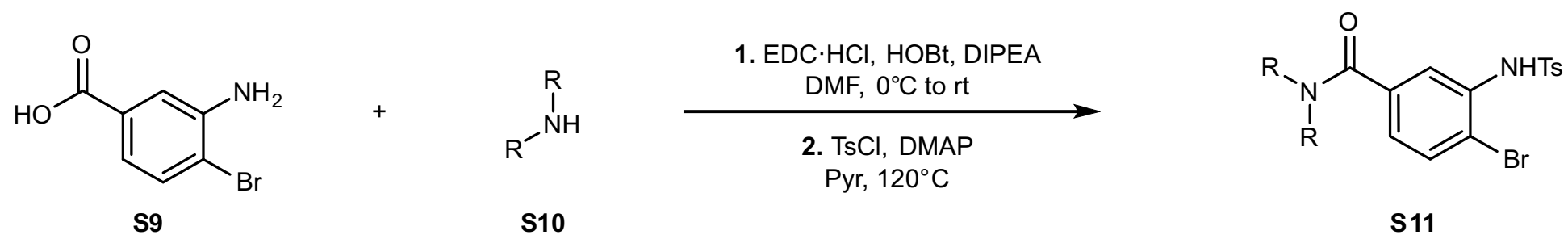


1-Hydroxybenzotriazole hydrate (1.01 equiv), the corresponding secondary amine coupling partner **S10** (1.00 equiv), and EDC·HCl (1.02 equiv) were added in sequence to a solution of 3-amino-4-bromobenzoic acid **S9** (1.50 equiv) in N,N-dimethylformamide (0.80M) at 0 °C. *N,N*-Diisopropylethylamine (2.20 equiv) was then added dropwise at 0 °C. The reaction mixture was allowed to warm to r.t. and stirred for 16 hours. The product mixture was diluted with ethyl acetate, transferred to a separatory funnel, and the organic phase was washed sequentially with 10% aqueous citric acid, saturated aqueous ammonium chloride solution, water, 5M aqueous sodium hydroxide solution, and brine. The washed organic layer was dried over sodium sulfate. The dried solution was filtered, and the filtrate was concentrated. The residue obtained was used directly in the next step without any further purification.

*Para*-Toluenesulfonyl chloride (1.00 equiv) and 4-dimethylaminopyridine (0.10 equiv) were added to a solution of the residue obtained in the previous step (nominally, 1.00 equiv) in pyridine (0.70 M) at r.t. The reaction mixture was then placed to an oil bath that had been preheated to 120 °C and stirred for 16 hours at 120 °C. Subsequently, the reaction mixture was allowed to cool to r.t. over 1 hour and pyridine was removed under a steady stream of nitrogen. The residue obtained was dissolved in dichloromethane and transferred to a separatory funnel. The organic solution was washed with 1 M hydrochloric acid. The aqueous layer was extracted with dichloromethane. The combined organic layer was dried over sodium sulfate. The dried solution was filtered, and the filtrate was concentrated. The residue obtained was eluted over a short plug of silica gel to provide the tosylated product **S11** as amorphous solids. The residue obtained was used directly in the next step without any further purification.

Note: Purification of the substrate such as **11q** could be performed through a recrystallization from a mixture dichloromethane–hexanes (1:20 v/v) at −20 °C. However, we found that the unpurified reaction mixtures could be used directly in the next step without any further purification.

*Part 2) Synthesis of the amine-containing 2-bromoaniline sulfonamides **11r–11v** via amide moiety reduction:*

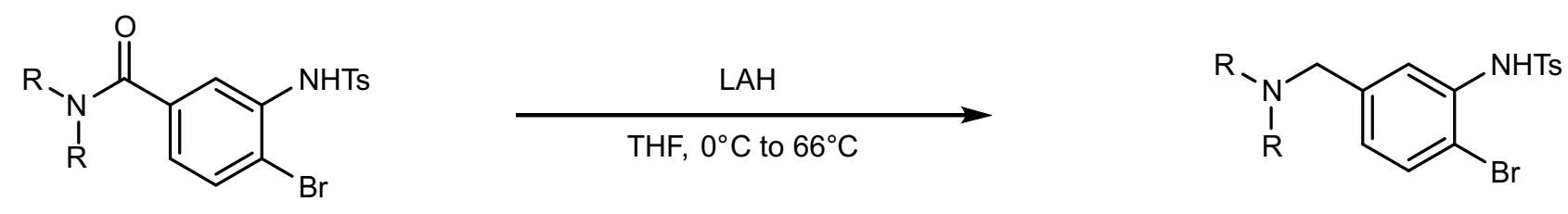


A solution of lithium aluminum hydride in tetrahydrofuran (2.00 M, 1.50 equiv) was added dropwise to a solution of the residue **S11** obtained in the previous step (nominally, 1.00 equiv) in tetrahydrofuran (0.50M) at 0 °C under a nitrogen atmosphere. The reaction mixture was then placed to an oil bath that had been preheated to 66 °C and stirred for 12 hours at 66 °C. The reaction mixture was cooled to 0 °C and carefully quenched using the Fieser work up[8]. For each gram of lithium aluminum hydride used, the reaction was quenched sequentially with Water (1 mL), 5M aqueous NaOH (1 mL), and Water (3 mL). The mixture was stirred for 5 min, anhydrous magnesium sulfate was added. The resulting suspension was filtered through a pad of Celite, washing with diethyl ether. The filtrate was concentrated under reduced pressure. The residue obtained was purified by flash-column chromatography eluting with (5% methanol–dichloromethane initially) to provide the amines **11r – 11v** as amorphous solids.

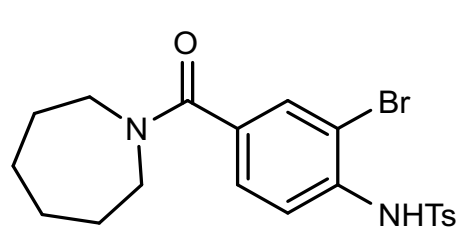


**11q**

N-(5-(azepane-1-carbonyl)-2-bromophenyl)-4-methylbenzenesulfonamide (**11q**). Prepared according to General Procedure **B**. $^{1}$H NMR (400 MHz, $CDCl_3$) δ 7.69 – 7.59 (m, 3H), 7.44 (d, *J* = 8.2 Hz, 1H), 7.25 – 7.16 (m, 2H), 7.08 (s, 1H), 7.00 (dd, *J* = 8.2, 2.0 Hz, 1H), 3.64 (t, 2H), 3.31 (t, 2H), 2.37 (s, 3H), 1.87 – 1.78 (m, 2H), 1.68 – 1.55 (m, 6H). $^{13}$C NMR (101 MHz, $CDCl_3$) δ 169.5, 144.5, 137.8, 135.7, 134.6, 133.0, 129.8, 127.3, 124.6, 120.0, 116.0, 49.7, 46.5, 29.4, 27.8, 27.2, 26.5, 21.6. HRMS (ESI-TOF) m/z: $[M + H]^+$ calcd for $C_{20}H_{24}BrN_2O_3S$, 451.0686; found, 451.0697.

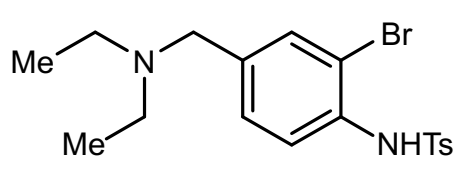


**11r**

N-(2-bromo-5-((diethylamino)methyl)phenyl)-4-methylbenzenesulfonamide (**11r**). Prepared according to General Procedure **B**. $^{1}$H NMR (400 MHz, $CDCl_3$) δ 7.69 – 7.60 (m, 3H), 7.32 (d, *J* = 8.2 Hz, 1H), 7.20 (d, *J* = 8.1 Hz, 2H), 7.03 – 6.94 (m, 1H), 3.50 (s, 2H), 2.49 (q, *J* = 7.1 Hz, 4H), 2.37 (s, 3H), 1.03 (t, *J* = 7.1 Hz, 6H). $^{13}$C NMR (101 MHz, $CDCl_3$) δ 144.3, 136.0, 134.4, 132.3,

129.7, 127.6, 126.8, 122.7, 57.0, 46.9, 21.7, 11.9. HRMS (ESI-TOF) m/z: $[M + H]^+$ calcd for $C_{18}H_{24}BrN_2O_2S$, 411.0737; found, 411.0747.

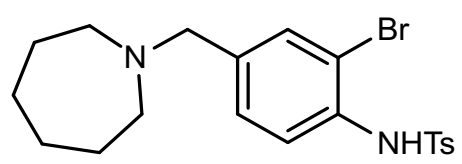


**11s**

N-(5-(azepan-1-ylmethyl)-2-bromophenyl)-4-methylbenzenesulfonamide (**11s**). Prepared according to General Procedure **B**. $^1$H NMR (400 MHz, $CDCl_3$) δ 7.72 – 7.57 (m, 3H), 7.32 (d, *J* = 8.2 Hz, 1H), 7.20 (d, *J* = 8.1 Hz, 2H), 6.96 (dd, *J* = 8.2, 2.0 Hz, 1H), 3.56 (s, 2H), 2.55 (t, *J* = 4.5 Hz, 4H), 2.37 (s, 3H), 1.61 (s, 8H). $^{13}$C NMR (101 MHz, $CDCl_3$) δ 144.3, 141.6, 136.0, 134.5, 132.2, 129.7, 127.6, 126.7, 122.6, 113.4, 62.2, 55.7, 28.5, 27.1, 21.7. HRMS (ESI-TOF) m/z: $[M + H]^+$ calcd for $C_{20}H_{26}BrN_2O_2S$, 437.0893; found, 437.0904.

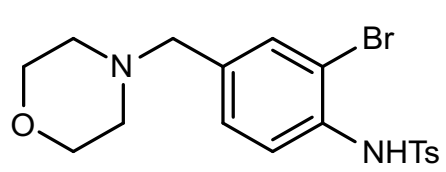


**11t**

N-(2-bromo-5-(morpholinomethyl)phenyl)-4-methylbenzenesulfonamide (**11t**). Prepared according to General Procedure **B**. $^1$H NMR (400 MHz, $CDCl_3$) δ 7.67 – 7.60 (m, 3H), 7.33 (d, *J* = 8.2 Hz, 1H), 7.20 (d, *J* = 8.1 Hz, 2H), 7.01 (s, 1H), 6.93 (dd, *J* = 8.1, 2.0 Hz, 1H), 3.71 – 3.66 (m, 4H), 3.42 (s, 2H), 2.40 – 2.34 (m, 7H). $^{13}$C NMR (101 MHz, $CDCl_3$) δ 144.3, 139.1, 135.9, 134.6, 132.4, 129.7, 127.5, 126.9, 122.8, 113.9, 67.0, 62.5, 53.5, 21.7. HRMS (ESI-TOF) m/z: $[M + H]^+$ calcd for $C_{18}H_{22}BrN_2O_3S$, 425.0529; found, 425.0536.

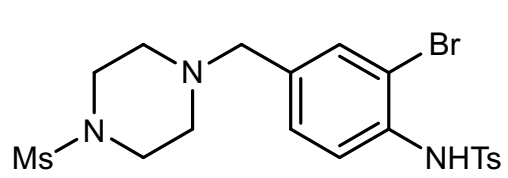


**11u**

N-(2-bromo-5-((4-(methylsulfonyl)piperazin-1-yl)methyl)phenyl)-4-methylbenzenesulfonamide (**11u**). Prepared according to General Procedure **B**. $^1$H NMR (400 MHz, $CDCl_3$) δ 7.68 – 7.59 (m, 3H), 7.34 (d, *J* = 8.2 Hz, 1H), 7.21 (d, *J* = 8.0 Hz, 2H), 6.97 (s, 1H), 6.90 (dd, *J* = 8.2, 2.1 Hz, 1H), 3.49 (s, 2H), 3.24 (t, *J* = 4.8 Hz, 4H), 2.79 (s, 3H), 2.50 (t, *J* = 4.9 Hz, 4H), 2.38 (s, 3H). $^{13}$C NMR (101 MHz, $CDCl_3$) δ 144.4, 138.7, 135.8, 134.7, 132.4, 129.7, 127.3, 126.7, 122.8, 114.2, 61.5, 52.1, 45.9, 34.4, 21.6. HRMS (ESI-TOF) m/z: $[M + H]^+$ calcd for $C_{19}H_{25}BrN_3O_4S_2$, 502.0465; found, 502.0475.

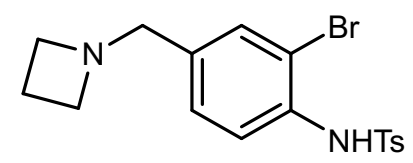

**11v**

N-(5-(azetidin-1-ylmethyl)-2-bromophenyl)-4-methylbenzenesulfonamide (**11v**). Prepared according to General Procedure **B**. $^{1}$H NMR (400 MHz, $CDCl_3$) δ 7.60 (d, *J* = 8.2 Hz, 2H), 7.52 (d, *J* = 2.1 Hz, 1H), 7.30 (d, *J* = 8.2 Hz, 1H), 7.17 (d, *J* = 8.1 Hz, 2H), 6.90 (dd, *J* = 8.2, 2.1 Hz, 1H), 6.74 (s, 1H), 3.50 (s, 2H), 3.21 (t, *J* = 7.1 Hz, 4H), 2.34 (s, 3H), 2.12 – 2.03 (m, 2H). $^{13}$C NMR (101 MHz, $CDCl_3$) δ 144.1, 139.0, 134.6, 132.5, 129.6, 127.4, 126.7, 123.4, 114.7, 77.4, 62.7, 55.1, 21.6, 17.7. HRMS (ESI-TOF) m/z: $[M + H]^+$ calcd for $C_{17}H_{20}BrN_2O_2S$, 395.0424; found, 395.0435.

**General Procedure C: Synthesis of 2-bromoaniline sulfonamides 11x–11aa.**

*Part 1) Synthesis of the 2-bromoanilines* ***S14*** *via nucleophilic aromatic substitution:*

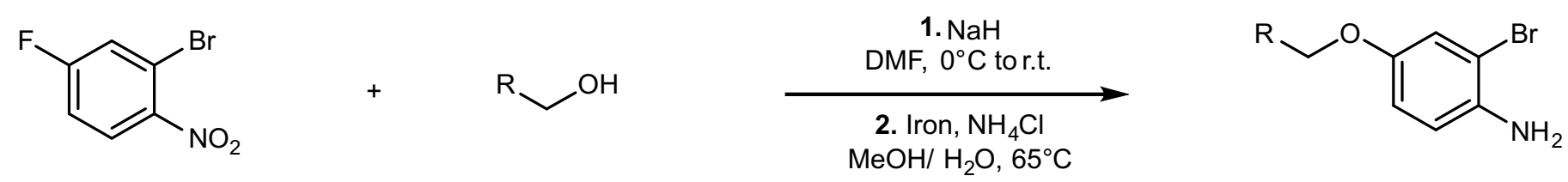


S12 S13 S14

Sodium hydride (1.50 equiv, 60% dispersion in mineral oil, washed three times with pentane prior to use) was added in one portion to a solution of the corresponding alcohol substrate **S13** (1.20 equiv) in tetrahydrofuran (0.10M) at 0 °C. The reaction mixture was stirred for 1 hour at 0 °C. Then, 2-bromo-4-fluoro-1-nitrobenzene **S12** (1.00 equiv) was added, and the reaction mixture was slowly warmed to room temperature and stirred for 16 hours. The product mixture was diluted sequentially with water and ethyl acetate. The resulting biphasic mixture was transferred to a separatory funnel and the layers that formed were separated. The aqueous layer was extracted with ethyl acetate. The organic layers were combined, and the combined organic layers were washed with saturated aqueous sodium chloride solution. The washed organic layer was dried over sodium sulfate. The dried solution was filtered and the filtrate was concentrated. The residue obtained was plugged through a short pad of silica eluting with (1% ethyl acetate–hexanes initially), afford the corresponding nitroarene intermediate, which was used directly in the next step without any further purification.

Iron powder (4.00 equiv), and ammonium chloride (6.00 equiv) were added to a solution of corresponding residue obtained in the previous step (nominally, 1.00 equiv) dissolved in a mixture of methanol/water (0.20M, 2:1 v/v). The reaction mixture was then placed to an oil bath that had been preheated to 65 °C and stirred for 16 hours at 65 °C. The reaction mixture was cooled and the mixture was then filtered through a pad of Celite, and the filtrate was concentrated under reduced pressure. The residue obtained was diluted sequentially with water and dichloromethane. The aqueous layer was extracted with dichloromethane. The organic layers were combined. The combined organic layer was dried over sodium sulfate. The dried solution was filtered and the filtrate was concentrated to afford the corresponding aniline residue **S14**, which was used directly in the next step without any further purification.

*Part 2) Synthesis of the 2-bromoaniline sulfonamides substrates* ***11x–11aa****:*

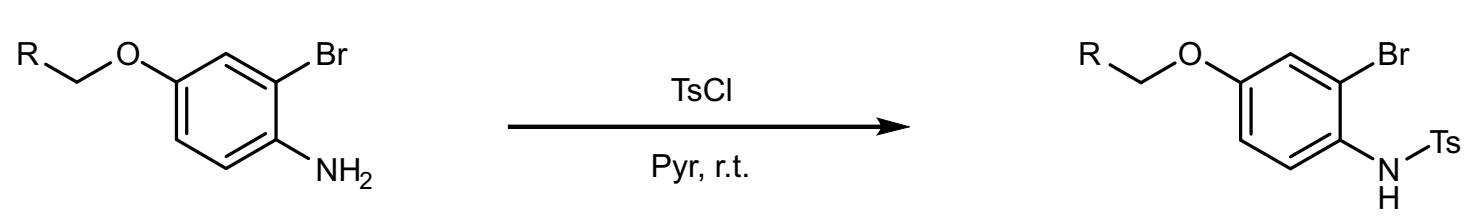


S14 11x - 11aa

*Para*-toluenesulfonyl chloride (1.05 equiv) was added in one portion to a solution of the residue obtained in the previous step (nominally, 1.00 equiv) in pyridine (0.70M) at r.t. The reaction

mixture was stirred for 16 hours. Pyridine was removed under a steady stream of nitrogen for 1h. The residue obtained was dissolved in dichloromethane and transferred to a separatory funnel. The organic solution was washed with 1M hydrochloric acid. The aqueous layer was extracted with dichloromethane. The organic layers were combined and washed with saturated aqueous sodium chloride solution. The washed organic layer was dried over sodium sulfate. The dried solution was filtered, and the filtrate was concentrated. The residue obtained was purified by flash-column chromatography eluting with (15% ethyl acetate–hexanes initially) to provide the corresponding aryl sulfonamide substrates **11x–11aa**.

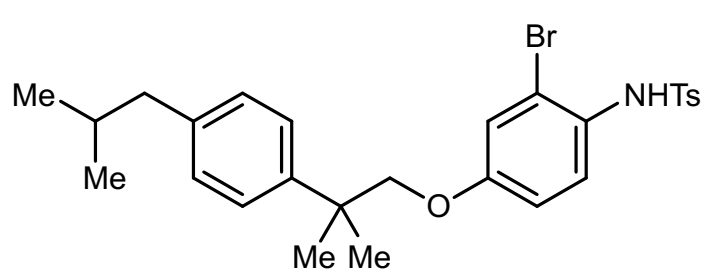


**11x**

N-(2-bromo-4-(2-(4-isobutylphenyl)-2-methylpropoxy)phenyl)-4-methylbenzenesulfonamide **(11x).** Prepared according to General Procedure C. Using 2-bromo-4-fluoro-1-nitrobenzene (660 mg, 3.00 mmol) afforded the final product (114 mg, 0.215 mmol, 7.2% over three steps). $^{1}$H NMR (400 MHz, $CDCl_3$) δ 7.58 – 7.53 (m, 3H), 7.29 (d, *J* = 8.3 Hz, 2H), 7.21 – 7.16 (m, 2H), 7.12 – 7.08 (m, 2H), 6.91 (d, *J* = 2.8 Hz, 1H), 6.82 (dd, *J* = 8.9, 2.8 Hz, 1H), 6.59 (s, 1H), 3.83 (s, 2H), 2.45 (d, *J* = 7.2 Hz, 2H), 2.38 (s, 3H), 1.90 – 1.80 (m, 1H), 1.42 (s, 6H), 0.91 (d, *J* = 6.6 Hz, 6H). $^{13}$C NMR (101 MHz, $CDCl_3$) δ 157.7, 144.11, 143.7, 139.8, 136.0, 129.7, 129.1, 127.6, 127.5, 125.8, 125.8, 118.5, 118.0, 115.1, 78.01, 45.1, 38.7, 30.3, 25.9, 22.6, 21.7. HRMS (ESI-TOF) m/z: $[M + H]^+$ calcd for $C_{27}H_{33}BrNO_3S$, 530.1359; found, 530.1367.

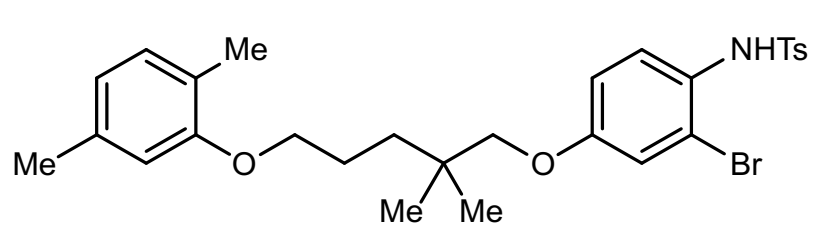


**11y**

N-(2-bromo-4-((5-(2,5-dimethylphenoxy)-2,2-dimethylpentyl)oxy)phenyl)-4-methylbenzenesulfonamide **(11y).** Prepared according to General Procedure C. Using 2-bromo-4-fluoro-1-nitrobenzene (660 mg, 3.00 mmol) afforded the final product (288 mg, 0.514 mmol, 17% over three steps). $^{1}$H NMR (400 MHz, $CDCl_3$) δ 7.62 – 7.54 (m, 3H), 7.20 (d, 2H), 7.00 (dd, *J* = 7.4, 0.9 Hz, 1H), 6.95 (d, *J* = 2.8 Hz, 1H), 6.85 (dd, *J* = 9.0, 2.8 Hz, 1H), 6.68 – 6.64 (m, 1H), 6.61 (s, 2H), 3.92 (t, *J* = 6.4 Hz, 2H), 3.58 (s, 2H), 2.38 (s, 3H), 2.30 (s, 3H), 2.17 (s, 3H), 1.84 – 1.72 (m, 2H), 1.56 – 1.51 (m, 2H), 1.02 (s, 6H). $^{13}$C NMR (101 MHz, $CDCl_3$) δ 157.8, 157.1, 144.1, 136.6, 136.0, 130.4, 129.7, 127.5, 127.5, 125.9, 123.7, 120.8, 118.3, 118.1, 114.9, 112.1, 68.5, 35.4, 34.3, 24.5, 24.3, 21.7, 21.5, 15.9. HRMS (ESI-TOF) m/z: $[M + H]^+$ calcd for $C_{28}H_{35}BrNO_4S$, 560.1465; found, 560.1470.

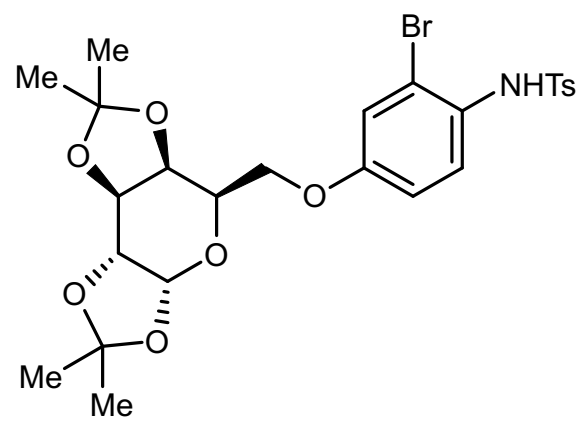


**11z**

N-(2-bromo-4-(((3aR,5R,5aS,8aS,8bR)-2,2,7,7-tetramethyltetrahydro-5H-bis([1,3]dioxolo)[4,5-b:4',5'-d]pyran-5-yl)methoxy)phenyl)-4-methylbenzenesulfonamide **(11z).** Prepared according to General Procedure C. Using 2-bromo-4-fluoro-1-nitrobenzene (880 mg, 4.00 mmol) afforded the final product (1.44 g, 2.46 mmol, 62% over three steps). $^{1}$H NMR (400 MHz, $CDCl_3$) δ 7.60 – 7.50 (m, 3H), 7.18 (d, *J* = 8.1 Hz, 2H), 6.98 (d, *J* = 2.8 Hz, 1H), 6.88 (dd, *J* = 9.0, 2.8 Hz, 1H), 6.64 (s, 1H), 5.55 (d, *J* = 5.0 Hz, 1H), 4.64 (dd, *J* = 7.9, 2.5 Hz, 1H), 4.37 – 4.27 (m, 2H), 4.14 – 3.99 (m, 3H), 2.37 (s, 3H), 1.50 (s, 3H), 1.45 (s, 3H), 1.34 (d, *J* = 3.1 Hz, 6H). $^{13}$C NMR (101 MHz, $CDCl_3$) δ 156.9, 144.1, 135.9, 129.7, 127.9, 127.5, 125.7, 118.7, 118.0, 115.2, 109.7, 108.9, 96.5, 71.1, 70.7, 70.6, 67.4, 66.3, 26.2, 26.1, 25.0, 24.6, 21.7. HRMS (ESI-TOF) m/z: $[M + H]^+$ calcd for $C_{25}H_{31}BrNO_8S$, 584.0949; found, 584.0959.

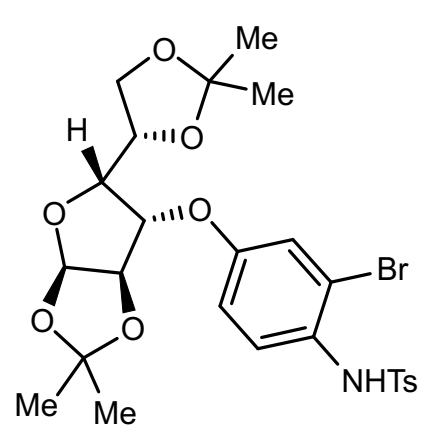


**11aa**

N-(2-bromo-4-(((3aR,5R,6S,6aR)-5-(2,2-dimethyl-1,3-dioxolan-4-yl)-2,2-dimethyltetrahydrofuro[2,3-d][1,3]dioxol-6-yl)oxy)phenyl)-4-methylbenzenesulfonamide **(11aa).** Prepared according to General Procedure C. Using 2-bromo-4-fluoro-1-nitrobenzene (500 mg, 2.27 mmol) afforded the final product (230 mg, 0.350 mmol, 15% over three steps). $^{1}$H NMR (400 MHz, $CDCl_3$) δ 7.63 – 7.57 (m, 3H), 7.22 (d, 2H), 7.07 (d, *J* = 2.8 Hz, 1H), 6.93 (dd, *J* = 8.9, 2.8 Hz, 1H), 6.70 (s, 1H), 5.90 (d, *J* = 3.8 Hz, 1H), 4.62 (d, *J* = 3.0 Hz, 1H), 4.52 (d, *J* = 3.8 Hz, 1H), 4.38 – 4.32 (m, 1H), 4.23 (dd, *J* = 8.1, 3.0 Hz, 1H), 4.12 (dd, *J* = 8.7, 6.1 Hz, 1H), 4.06 (dd, *J* = 8.7, 5.2 Hz, 1H), 2.39 (s, 3H), 1.53 (s, 3H), 1.42 (s, 3H), 1.30 (d, *J* = 7.6 Hz, 6H). $^{13}$C NMR (101 MHz, $CDCl_3$) δ 155.1, 144.3, 136.1, 129.8, 128.9, 127.5, 125.4, 119.9, 117.7, 115.9, 112.4, 109.5, 105.3, 82.3, 80.8, 80.5, 72.1, 67.4, 27.0, 26.8, 26.4, 25.4, 21.7. HRMS (ESI-TOF) m/z: $[M + H]^+$ calcd for $C_{25}H_{31}BrNO_8S$, 584.0949; found, 584.0966.

**Synthetic procedure for the preparation of substrates 11ag:**

*Synthesis of the amide-containing ortho-bromophenol **11ag**:*

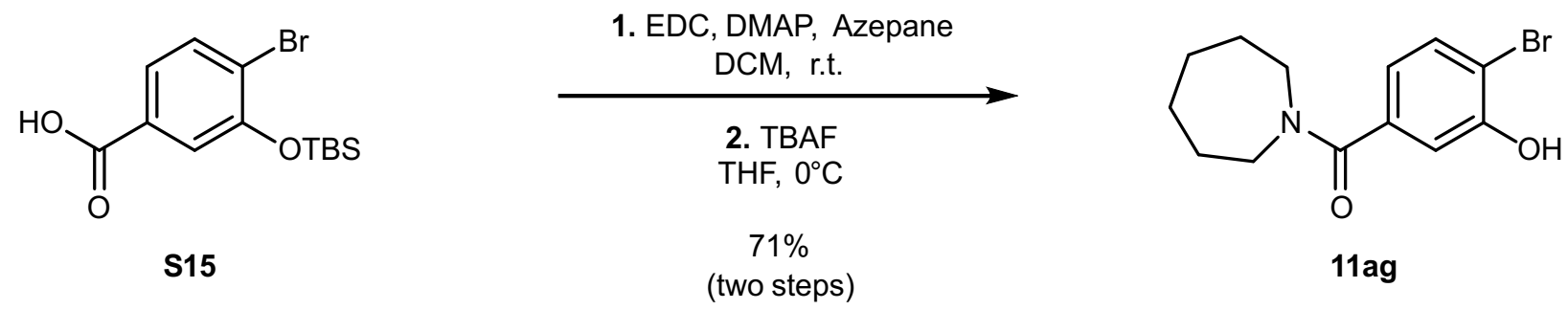


(4-Dimethylamino)pyridine (733 mg, 6.00 mmol, 2.00 equiv), Azepane (507 µL, 4.50 mmol, 1.50 equiv), and EDC (1.15 g, 6.00 mmol, 2.00 equiv) were added in sequence to a solution of the tert-butyldimethylsilyl protected phenol **S15**[3] (994 mg, 3.00 mmol, 1.00 equiv) in dichloromethane (15.0 mL, 0.20M) at r.t. The reaction mixture was stirred at r.t. for 12 hours. The product mixture was then diluted sequentially with dichloromethane (30 mL), water (20 mL), and saturated aqueous ammonium chloride solution (20 mL). The resulting biphasic mixture was transferred to a separatory funnel and the layers that formed were separated. The aqueous layer was extracted with dichloromethane (3 × 30 mL). The organic layers were combined and the combined organic layers were washed with saturated aqueous sodium chloride solution (100 mL). The washed organic layer was dried over sodium sulfate. The dried solution was filtered and the filtrate was concentrated. The residue obtained was used directly in the next step without any further purification. A solution of tetrabutylammonium fluoride (TBAF, 1.00M in THF, 6.00 mL, 6.00 mmol, 2.00 equiv) was added dropwise via syringe over 10 min to the residue obtained in the previous step (nominally, 1.0 equiv) in tetrahydrofuran (15.0 mL, 0.2M) at 0 °C. The reaction mixture was stirred for 5 hours at 0 °C. The cold product mixture was diluted sequentially with saturated aqueous ammonium chloride solution, saturated aqueous sodium chloride solution (20 mL), and ethyl acetate (20 mL). The diluted product mixture was then allowed to warm to r.t. over 20 min. The warmed biphasic mixture was transferred to a separatory funnel and the layers that formed were separated. The aqueous layer was extracted with ethyl acetate (3 × 30 mL). The organic layers were combined, and the combined organic layers were washed with saturated aqueous sodium chloride solution (100 mL). The washed organic layer was dried over sodium sulfate. The dried solution was filtered and the filtrate was concentrated. The residue obtained was purified by flash-column chromatography (eluting with 20% ethyl acetate–hexanes initially) to provide the ortho-bromophenol **11ag** (635 mg, 71% yield) as a colorless oil.

$^{1}$H NMR (400 MHz, $CDCl_3$) δ 7.44 (d, *J* = 8.1 Hz, 1H), 7.36 (s, 1H), 7.02 (d, *J* = 1.9 Hz, 1H), 6.74 (dd, *J* = 8.1, 1.9 Hz, 1H), 3.70 – 3.63 (m, 2H), 3.40 – 3.32 (m, 2H), 1.87 – 1.79 (m, 2H), 1.64 – 1.55 (m, 6H). $^{13}$C NMR (101 MHz, $CDCl_3$) δ 171.0, 153.3, 137.5, 132.6, 119.3, 115.2, 111.5, 50.0, 46.7, 29.6, 27.9, 27.2, 26.6. HRMS (ESI-TOF) m/z: $[M + H]^+$ calcd for $C_{13}H_{17}BrNO_2$, 298.0437; found, 298.0443.

**Synthetic Procedure for the Preparation of Allylic Bromides 12ai, 12aj, and 12ak.**

*Synthesis of the cyclopropane-containing allylic bromide* ***12ai****:*

*Part 1) Synthesis of cyclopropane* ***S18*** *via alkylation:*

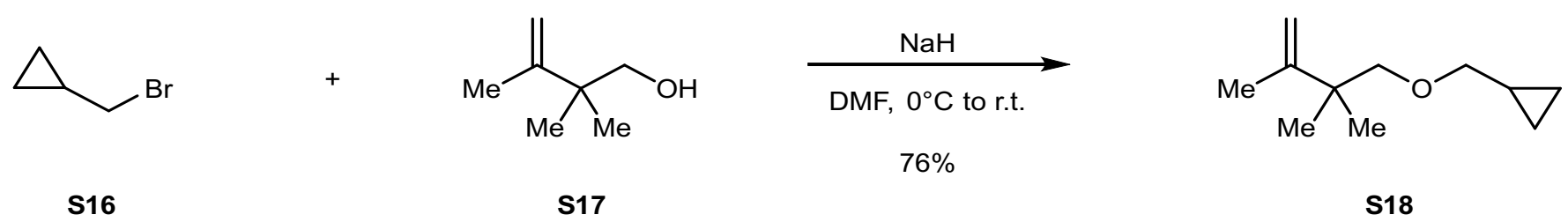


Sodium hydride (911 mg, 22.8 mmol, 2.0 equiv, 60% dispersion in mineral oil) was added in one portion to a solution of the known alcohol **S17**[3] (1.30 g, 11.4 mmol, 1.0 equiv) in *N,N*-dimethylformamide (37.9 mL, 0.30M) at 0 °C. The resulting mixture was stirred for 20 min at 0 °C. (Bromomethyl)cyclopropane **S16** (2.21 mL, 22.8 mmol, 2.0 equiv) was then added dropwise via syringe at 0 °C. The reaction mixture was allowed to slowly warm to r.t. and stirred for 16 hours. The reaction mixture was carefully quenched with water (40 mL) and diluted with diethyl ether (40 mL). The resulting biphasic mixture was transferred to a separatory funnel and the layers that formed were separated. The aqueous layer was extracted with diethyl ether (3 × 40 mL). The organic layers were combined, and the combined organic layers were washed with saturated aqueous ammonium chloride solution (40 mL), water (40 mL), and saturated aqueous sodium chloride solution (40 mL). The washed organic layer was dried over anhydrous sodium sulfate. The dried solution was filtered and the filtrate was concentrated under reduced pressure. The residue obtained was purified by flash-column chromatography eluting with (5% ethyl acetate–hexanes initially) to provide cyclopropane **S18** as a colorless liquid (1.45 g, 76% yield).

$^{1}$H NMR (400 MHz, $CDCl_3$) δ 4.85 – 4.74 (m, 2H), 3.29 (s, 2H), 3.28 (d, *J* = 6.7 Hz, 2H), 1.82 – 1.71 (t, 3H), 1.07 (s, 6H), 1.02 (m, *J* = 6.7, 1.8 Hz, 1H), 0.57 – 0.43 (m, 2H), 0.18 (m, *J* = 6.1, 4.5 Hz, 2H). $^{13}$C NMR (101 MHz, $CDCl_3$) δ 151.1, 110.0, 78.4, 76.0, 40.6, 24.7, 19.9, 10.6, 3.0. HRMS (ESI-TOF) m/z: $[M + H]^+$ calcd for $C_{11}H_{21}O$, 169.1587; found, 169.1581.

*Part 2) Synthesis of the cyclopropane-containing allylic alcohol* ***S19****:*

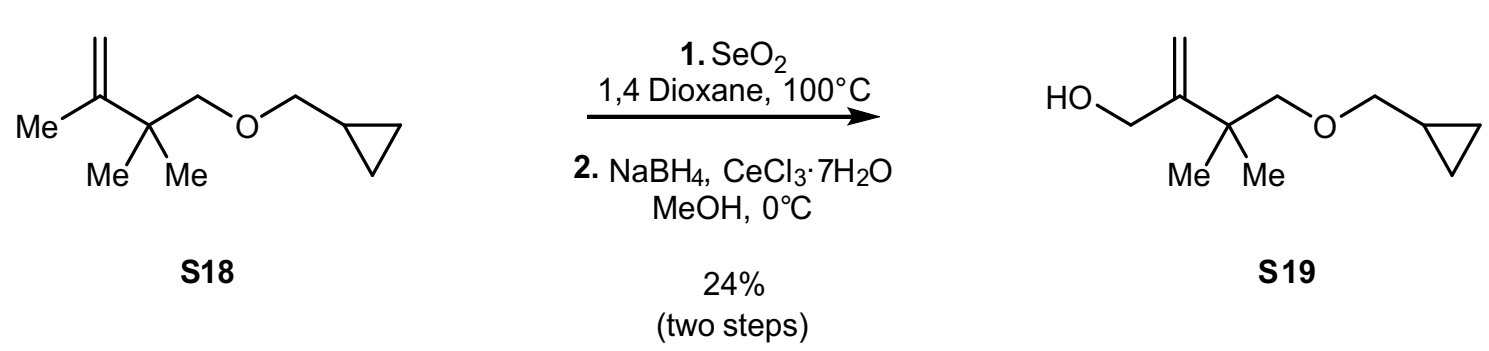


Selenium dioxide (956 mg, 8.62 mmol, 1.0 equiv) was added to a solution of the cyclopropane **S18** (1.45 g, 8.62 mmol, 1.0 equiv) in 1,4-dioxane (28.7 mL, 0.30M). The reaction mixture was then placed to an oil bath that had been preheated to 100 °C and stirred for 1 hour at 100 °C. The reaction was cooled to room temperature. The cooled reaction mixture was filtered through a pad of Celite, and the filtrate was diluted sequentially with diethyl ether (40 mL). The organic layer was washed with water (3 × 40 mL), saturated aqueous sodium bicarbonate solution (40 mL) and saturated aqueous sodium chloride solution (40 mL). The washed organic layer was dried over anhydrous sodium sulfate. The dried solution was filtered and the filtrate was concentrated under

reduced pressure to afford a residue, which was used directly in the next step without further purification.

Cerium(III) chloride heptahydrate (3.85 g, 10.3 mmol, 1.2 equiv) was added to a solution of the residue obtained in the previous step (nominally, 1.00 equiv) dissolved in methanol (24.6 mL, 0.35M) at 0 °C. After stirring the reaction mixture for 5 min at 0 °C, sodium borohydride (329 mg, 8.71 mmol, 1.01 equiv) was added in two portions over 5 min intervals. The reaction mixture was then stirred at 0 °C for 15 min and then quenched with water (5 mL) and 1 M hydrochloric acid (15 mL). The product mixture was transferred to a separatory funnel. The layers were separated and the aqueous layer was extracted with diethyl ether (3 × 30 mL). The organic layers were combined, washed with saturated aqueous sodium chloride solution (40 mL). The washed organic layer was dried over anhydrous sodium sulfate. The dried solution was filtered and the filtrate was concentrated under reduced pressure. The residue obtained was purified by flash-column chromatography eluting with (15% ethyl acetate–hexanes initially) provide cyclopropane alcohol **S19** as a colorless liquid (350 mg, 24% yield over two steps).

$^1$H NMR (400 MHz, $CDCl_3$) δ 4.99 (q, *J* = 1.0 Hz, 1H), 4.88 (d, *J* = 1.2 Hz, 1H), 3.93 (d, *J* = 0.9 Hz, 2H), 3.12 (s, 2H), 3.10 (d, *J* = 6.9 Hz, 2H), 0.95 (s, 6H), 0.90 – 0.81 (m, 1H), 0.37 – 0.31 (m, 2H), 0.01 (m, *J* = 6.0, 4.5 Hz, 2H). $^{13}$C NMR (101 MHz, $CDCl_3$) δ 154.3, 113.4, 80.1, 76.2, 64.7, 39.3, 25.2, 10.2, 3.0. HRMS (ESI-TOF) m/z: $[M + H]^+$ calcd for $C_{11}H_{21}O_2$, 185.1536; found, 185.1537.

*Part 3) Synthesis of cyclopropane-containing bromide **12ai**:*

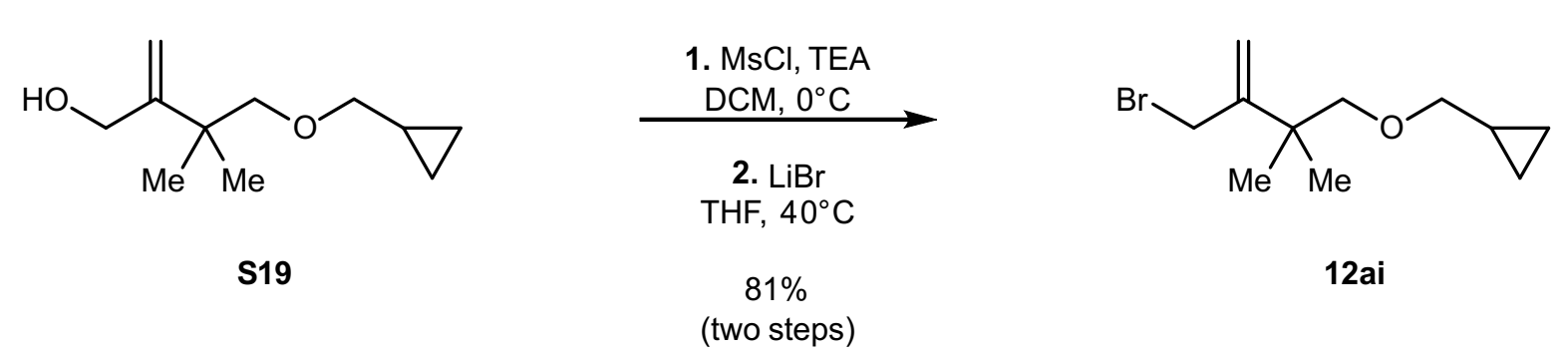

S19 12ai

Triethylamine (371 µL, 2.66 mmol, 1.4 equiv) was added dropwise via syringe to a solution of alcohol **S19** (350 mg, 1.90 mmol, 1.0 equiv) and methanesulfonyl chloride (178 µL, 2.28 mmol, 1.2 equiv) in dichloromethane (9.50 mL, 0.20M) at 0 °C. The reaction mixture was stirred for 1 hour at 0 °C. The reaction mixture was diluted sequentially with water (10 mL) and diethyl ether (40 mL) and transferred to a separatory funnel. The layers were separated. The organic layer was washed with 1M hydrochloric acid solution (10 mL), saturated aqueous sodium bicarbonate solution (10 mL), and saturated aqueous sodium chloride solution (10 mL), dried over sodium

sulfate, filtered, and concentrated to afford the crude mesylate intermediate, which was used directly in the next step without further purification.

Lithium bromide (173 mg, 2.00 mmol, 1.05 equiv) was added to a solution of the crude mesylate residue obtained in the previous step (nominally, 1.00 equiv) in tetrahydrofuran (2.71 mL, 0.70M). The reaction mixture was then placed to an oil bath that had been preheated to 40 °C and stirred for 30 min at 40 °C. The reaction mixture was cooled. The cooled reaction mixture was diluted with water (10 mL) and diethyl ether (20 mL) and transferred to a separatory funnel. The layers were separated. The organic layer was washed with 1M hydrochloric acid solution (10 mL), saturated aqueous sodium bicarbonate solution (10 mL), and saturated aqueous sodium chloride solution (10 mL), dried over sodium sulfate, filtered, and concentrated. The residue obtained was purified by flash-column chromatography eluting with (5% ethyl acetate–hexanes initially) to provide bromide **12ai** as a light-yellow oil (380 mg, 81% yield over two steps).

$^{1}$H NMR (400 MHz, $CDCl_3$) δ 5.37 (d, *J* = 0.9 Hz, 1H), 5.23 (s, 1H), 4.12 (s, 2H), 3.34 (s, 2H), 3.27 (d, *J* = 6.7 Hz, 2H), 1.18 (s, 6H), 1.02 (m, *J* = 8.2, 6.7, 4.9, 1.8 Hz, 1H), 0.60 – 0.41 (m, 2H), 0.18 (m, *J* = 6.0, 4.5 Hz, 2H). $^{13}$C NMR (101 MHz, $CDCl_3$) δ 151.0, 117.8, 79.0, 76.00, 40.4, 33.8, 25.5, 10.6, 3.0. HRMS (ESI-TOF) m/z: $[M + H]^+$ calcd for $C_{11}H_{20}BrO$, 247.0692; found, 247.0695.

*Synthesis of the oxetane-containing allylic bromide **12aj**:*

*Part 1) Synthesis of oxetane **S21** via alkylation:*

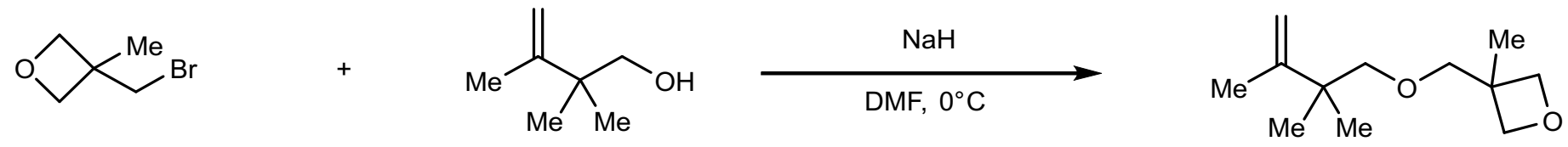


**S20** **S17** **S21**

Sodium hydride (222 mg, 5.55 mmol, 2.0 equiv, 60% dispersion in mineral oil) was added in one portion to a solution of Alcohol **S17** (317 mg, 2.78 mmol, 1.0 equiv) in *N,N*-dimethylformamide (13.9 mL, 0.20M) at 0 °C. The resulting mixture was stirred for 30 min at 0 °C. 3-(Bromomethyl)-3-methyloxetane **S20** (1.15 g, 6.94 mmol, 2.5 equiv) was then added dropwise via syringe at 0 °C. The reaction mixture was allowed to slowly warm to r.t. and stirred for 16 hours. The reaction mixture was carefully quenched with water (15 mL) and diluted with diethyl ether (30 mL). The resulting biphasic mixture was transferred to a separatory funnel and the layers that formed were separated. The aqueous layer was extracted with diethyl ether (3 × 30 mL). The organic layers were combined, and the combined organic layers were washed with saturated aqueous ammonium chloride solution (60 mL), water (60 mL), and saturated aqueous sodium chloride solution (60 mL). The washed organic layer was dried over anhydrous sodium sulfate. The dried solution was filtered and the filtrate was concentrated under reduced pressure. The residue obtained was eluted over a short plug of silica gel to provide the corresponding oxetane **S21** as a colorless liquid, which was used directly in the next step without any further purification.

*Part 2) Synthesis of the oxetane-containing allylic alcohol **S22**:*

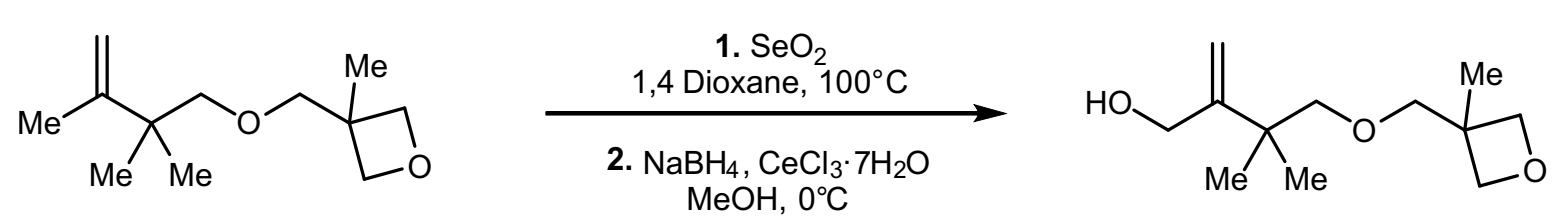


**S21** 47% (three steps) **S22**

Selenium dioxide (308 mg, 2.78 mmol, 1.00 equiv) was added to a solution of the oxetane residue **S21** obtained in the previous step (nominally, 1.00 equiv) in 1,4-dioxane (6.87 mL, 0.30M). The reaction mixture was then placed to an oil bath that had been preheated to 100 °C and stirred for 1 hour at 100 °C. The reaction was cooled to room temperature. The cooled reaction mixture was filtered through a pad of Celite, and the filtrate was diluted sequentially with diethyl ether (30 mL). The organic layer was washed with water (3 × 30 mL), saturated aqueous sodium bicarbonate solution (30 mL), and saturated aqueous sodium chloride solution (30 mL). The washed organic layer was dried over anhydrous sodium sulfate. The dried solution was filtered and the filtrate was

concentrated under reduced pressure to afford the crude aldehyde intermediate which was used directly in the next step without further purification.

Cerium(III) chloride heptahydrate (1.24 g, 3.34 mmol, 1.2 equiv) was added to a solution of the aldehyde residue obtained in the previous step (nominally, 1.00 equiv) in methanol (5.29 mL, 0.35M) at 0 °C. After 5 min, sodium borohydride (106 mg, 2.81 mmol, 1.01 equiv) was added in two portions over 5 min. The reaction mixture was stirred at 0 °C for 15 min. The reaction mixture was quenched with water (5 mL) followed by 1 M hydrochloric acid (10 mL) and transferred to a separatory funnel. The layers were separated and the aqueous layer was extracted with diethyl ether (3 × 20 mL). The organic layers were combined and washed with saturated aqueous sodium chloride solution (20 mL). The washed organic layer was dried over anhydrous sodium sulfate. The dried solution was filtered and the filtrate was concentrated under reduced pressure. The residue obtained was purified by flash-column chromatography (eluting with 15% ethyl acetate–hexanes initially) on silica gel to provide alcohol **S22** as a colorless liquid (280 mg, 47% yield over three steps).

$^{1}$H NMR (400 MHz, $CDCl_3$) δ 5.16 (q, *J* = 1.2 Hz, 1H), 5.05 (d, *J* = 1.1 Hz, 1H), 4.48 (d, *J* = 5.8 Hz, 2H), 4.35 (d, *J* = 5.8 Hz, 2H), 4.13 (d, *J* = 1.1 Hz, 2H), 3.49 (s, 2H), 3.34 (s, 2H), 1.29 (s, 3H), 1.13 (s, 6H). $^{13}$C NMR (101 MHz, $CDCl_3$) δ 154.3, 112.2, 81.0, 80.2, 76.9, 64.4, 40.2, 39.5, 25.1, 21.5. HRMS (ESI-TOF) m/z: $[M + H]^+$ calcd for $C_{12}H_{23}O_3$, 215.1642; found, 215.1648.

*Part 3) Synthesis of the Oxetane Bromide* ***12aj****:*

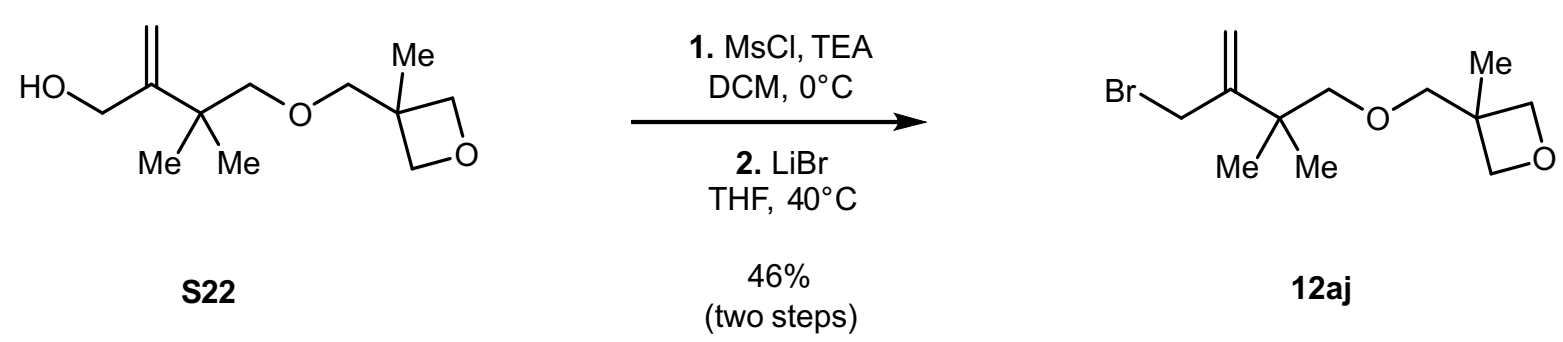


Triethylamine (178 mg, 1.76 mmol, 1.4 equiv) was added dropwise to a stirred solution of alcohol **S22** (270 mg, 1.26 mmol, 1.0 equiv) and methanesulfonyl chloride (173 mg, 1.51 mmol, 1.2 equiv) in dichloromethane (6.30 mL, 0.2 M) at 0 °C. The reaction mixture was stirred at 0 °C for 30 min and then allowed to warm to r.t. and stirred for 1 h. The reaction mixture was diluted with dichloromethane (20 mL) and quenched with water (20 mL). The resulting biphasic mixture was transferred to a separatory funnel and the layers were separated. The organic layer was washed with 1M hydrochloric acid solution (10 mL), saturated aqueous sodium bicarbonate solution (10 mL), and saturated aqueous sodium chloride solution (10 mL). The washed organic layer was dried over anhydrous sodium sulfate. The dried solution was filtered and the filtrate was concentrated under reduced pressure to afford the mesylate intermediate which was used directly in the next step without further purification.

Lithium bromide (131 mg, 1.51 mmol, 1.2 equiv) was added to a solution of the residue obtained in the previous step (nominally, 1.00 equiv) in tetrahydrofuran (1.80 mL, 0.70M). The reaction mixture was heated 40 °C and stirred for 30 min. The cooled reaction mixture was diluted with water (10 mL) and diethyl ether (20 mL) and transferred to a separatory funnel. The layers were separated. The organic layer was washed with 1M hydrochloric acid solution (10 mL), saturated aqueous sodium bicarbonate solution (10 mL), and saturated aqueous sodium chloride solution (10 mL), dried over sodium sulfate, filtered, and concentrated. The residue obtained was purified by flash-column chromatography (eluting with 2% ethyl acetate–hexanes initially) to provide the oxetane bromide **12aj** as a light-yellow liquid. (161 mg, 46% yield over two steps).

$^{1}$H NMR (400 MHz, $CDCl_3$) δ 5.36 (d, *J* = 0.8 Hz, 1H), 5.21 (s, 1H), 4.51 (d, *J* = 5.7 Hz, 2H), 4.33 (d, *J* = 5.7 Hz, 2H), 4.09 (s, 2H), 3.45 (s, 2H), 3.36 (s, 2H), 1.30 (s, 3H), 1.18 (s, 6H). $^{13}$C NMR (101 MHz, $CDCl_3$) δ 150.87, 117.88, 80.27, 80.06, 76.61, 40.46, 40.23, 33.67, 25.27, 21.59. HRMS (ESI-TOF) m/z: $[M + H]^+$ calcd for $C_{12}H_{22}BrO_2$, 277.0798; found, 277.0811.

*Synthesis of the benzoyl-containing allylic bromide **12ak**:*

*Part 1) Synthesis of the benzoyl protected alcohol **S23**:*

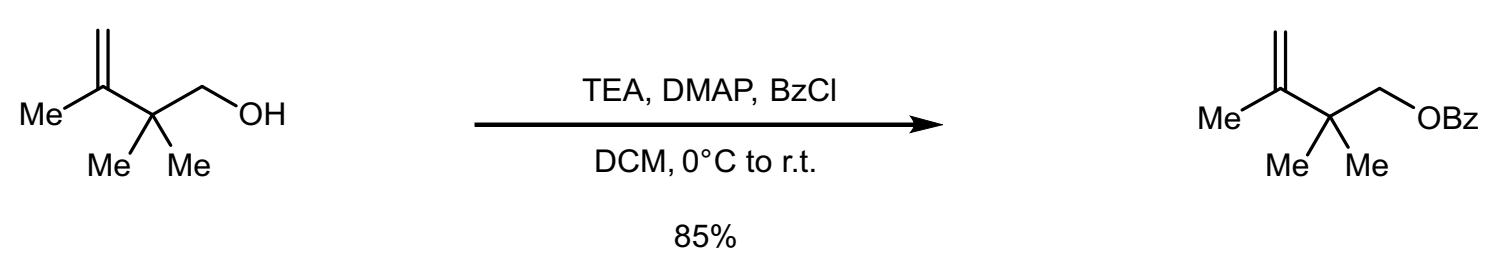


**S17** **S23**

Triethylamine (1.3 mL, 9.6 mmol, 1.1 equiv), 4-dimethylaminopyridine (320 mg, 2.6 mmol, 0.3 equiv), and benzoyl chloride (1.1 mL, 9.6 mmol, 1.1 equiv) were added in sequence to a solution of alcohol **S17** (1.0 g, 8.8 mmol, 1.0 equiv) in dichloromethane (17 mL, 0.50M) at 0 °C. The reaction mixture was slowly warmed to r.t. and stirred for 16 hours. The product mixture was quenched with saturated aqueous ammonium chloride solution (40 mL). The resulting biphasic mixture was transferred to a separatory funnel and the layers that formed were separated. The aqueous layer was extracted with dichloromethane (3 × 40 mL). The combined organic layer was washed with 1M hydrochloric acid (100 mL) and was dried over sodium sulfate. The dried solution was filtered, and the filtrate was concentrated. The residue obtained was purified by flash-column chromatography (eluting with 5% ethyl acetate–hexanes initially) to provide the benzoyl protected alcohol **S23** (1.62 g, 85% yield).

$^{1}$H NMR (400 MHz, $CDCl_3$) δ 8.12 – 7.95 (m, 2H), 7.59 – 7.53 (m, 1H), 7.48 – 7.40 (m, 2H), 4.87 (s, 2H), 4.20 (s, 2H), 1.81 (s, 3H), 1.19 (s, 6H). $^{13}$C NMR (101 MHz, $CDCl_3$) δ 171.2, 156.2, 144.4,

138.7, 138.4, 133.9, 131.1, 129.9, 127.5, 123.3, 114.4, 62.7, 51.5, 51.2, 50.1, 25.6, 23.7, 21.7, 20.7. HRMS (ESI-TOF) m/z: $[M + H]^+$ calcd for $C_{14}H_{19}O_2$, 219.1380; found, 219.1381.

*Part 2) Synthesis of the benzoyl-containing allylic bromide **12ak**:*

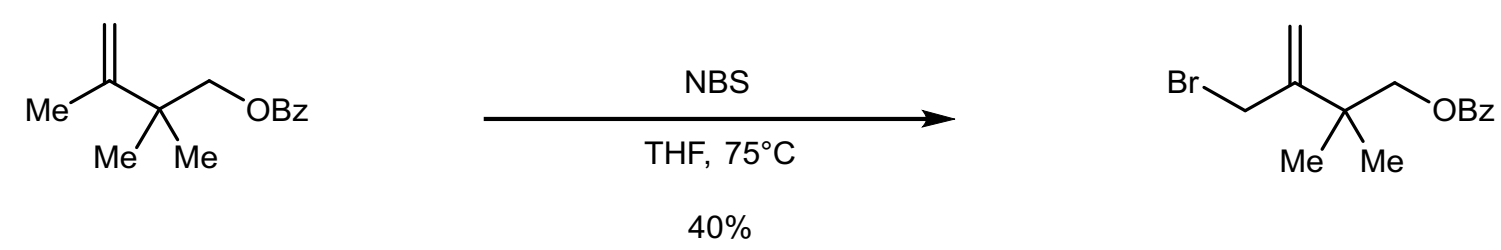


S23 12ak

*N*-bromosuccinimide (1.39 g, 7.81 mmol, 1.05 equiv) was added to a solution of alkene **S23** (1.62 g, 7.44 mmol, 1.0 equiv) in anhydrous tetrahydrofuran (37.2 mL, 0.2 M). The reaction mixture was then placed to an oil bath that had been preheated to 75 °C and stirred for 3 hours at 75 °C. The reaction mixture was cooled. The product mixture was diluted with ethyl acetate and water. The resulting biphasic mixture was transferred to a separatory funnel and the layers that formed were separated. The aqueous layer was extracted with ethyl acetate (3 × 40 mL). The organic layers were combined, and the combined organic layers were washed with saturated aqueous sodium chloride solution (100 mL). The washed organic layer was dried over sodium sulfate. The dried solution was filtered and the filtrate was concentrated. The residue obtained was purified by flash-column chromatography (eluting with 2% ethyl acetate–hexanes initially) to provide the allylic bromide substrates **12ak** (884 mg, 40% yield).

$^1$H NMR (400 MHz, CDCl3) δ 8.07 – 7.98 (m, 2H), 7.60 – 7.53 (m, 1H), 7.49 – 7.40 (m, 2H), 5.45 (s, 1H), 5.32 (s, 1H), 4.27 (s, 2H), 4.10 (s, 2H), 1.31 (s, 6H). $^{13}$C NMR (101 MHz, $CDCl_3$) δ 166.5, 149.4, 132.9, 130.4, 129.5, 128.3, 110.8, 71.5, 39.4, 24.3, 19.7. HRMS (ESI-TOF) m/z: $[M + H]^+$ calcd for $C_{14}H_{18}BrO_2$, 297.0485; found, 297.0497.

**General Procedure D: Synthesis of 2-bromoaniline sulfonamides 11an–11as.**

*Synthesis of **11an–11as** via sulfonyl group installation on the corresponding 2-bromoaniline:*

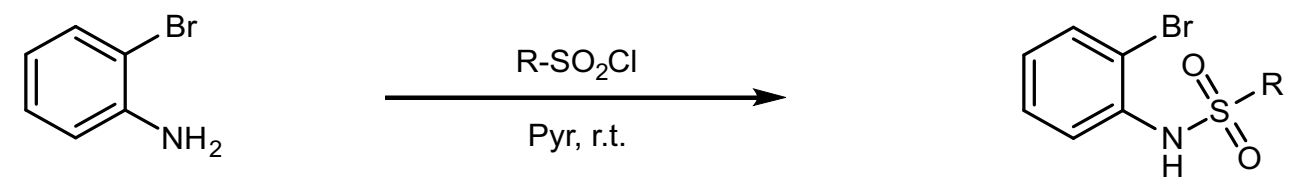


**S24** **11an - 11as**

To a solution of 2-bromoaniline **S24** (1.0 equiv) in pyridine (0.7 M) was added the corresponding commercially available sulfonyl chloride (1.1 equiv) at r.t. The resulting reaction mixture was stirred for 16 hours at r.t. Pyridine was then removed under a stream of nitrogen for 2h at r.t. The residue obtained was dissolved in dichloromethane and transferred to a separatory funnel. The organic solution was washed with 1M hydrochloric acid. The aqueous layer was extracted with dichloromethane. The organic layers were combined and dried over sodium sulfate. The dried solution was filtered, and the filtrate was concentrated under reduced pressure. The residue obtained was purified by flash-column chromatography (eluting with 2% ethyl acetate–hexanes initially) to provide the corresponding sulfonyl-protected bromoaniline substrates **11an–11as**.

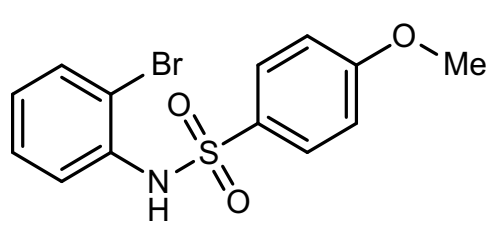


**11an**

N-(2-Bromophenyl)-4-methoxybenzenesulfonamide **(11an).** Prepared according to General Procedure D. Using 2-bromoaniline (455 mg, 2.64 mmol, 1.0 equiv) afforded the title compound as a white solid (530 mg, 1.55 mmol, 59% yield). $^{1}$H NMR (400 MHz, $CDCl_3$) δ 7.73 – 7.64 (m, 3H), 7.41 (dd, $J$ = 8.0, 1.4 Hz, 1H), 7.30 – 7.25 (m, 1H), 7.00 – 6.94 (m, 1H), 6.94 (s, 1H), 6.90 – 6.85 (m, 2H), 3.83 (s, 3H). $^{13}$C NMR (101 MHz, $CDCl_3$) δ 163.5, 135.0, 132.7, 130.5, 129.7, 128.7, 126.4, 122.8, 115.9, 114.3, 55.7. HRMS (ESI-TOF) m/z: $[M + H]^+$ calcd for $C_{13}H_{13}BrNO_3S$, 341.9794; found, 341.9807.

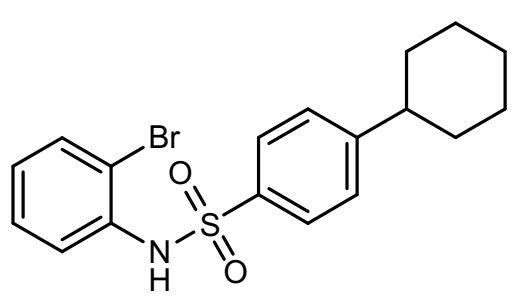


**11ao**

N-(2-Bromophenyl)-4-cyclohexylbenzenesulfonamide (**11ao**). Prepared according to General Procedure D. Using 2-bromoaniline (422 mg, 2.45 mmol, 1.0 equiv) afforded the title compound as a white solid (770 mg, 1.95 mmol, 80% yield). $^{1}$H NMR (400 MHz, $CDCl_3$) δ 7.70 – 7.64 (m, 3H), 7.40 (dd, $J$ = 8.1, 1.4 Hz, 1H), 7.28 – 7.23 (m, 3H), 6.98 (s, 1H), 6.97 – 6.92 (m, 1H), 2.60 – 2.46 (m, 1H), 1.86 – 1.79 (m, 4H), 1.78 – 1.70 (m, 1H), 1.41 – 1.31 (m, 4H), 1.29 – 1.16 (m, 1H).

$^{13}$C NMR (101 MHz, $CDCl_3$) δ 154.2, 136.2, 134.9, 132.7, 128.6, 127.6, 127.5, 126.3, 122.6, 115.8, 44.6, 34.1, 26.7, 26.0. HRMS (ESI-TOF) m/z: $[M + H]^+$ calcd for $C_{18}H_{21}BrNO_2S$, 394.0471; found, 394.0479.

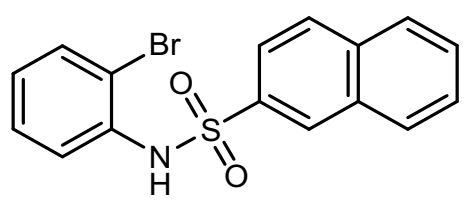


**11ap**

N-(2-Bromophenyl) naphthalene-2-sulfonamide (**11ap**). Prepared according to General Procedure D. Using 2-bromoaniline (625 mg, 3.63 mmol, 1.0 equiv) afforded the title compound as a white solid (1.00 g, 2.76 mmol, 76% yield). $^1$H NMR (400 MHz, $CDCl_3$) δ 8.38 (d, $J$ = 1.9 Hz, 1H), 7.85 (m, $J$ = 11.0, 8.2, 1.8 Hz, 3H), 7.75 (m, $J$ = 8.8, 1.8 Hz, 2H), 7.58 (m, $J$ = 19.2, 8.2, 6.9, 1.4 Hz, 2H), 7.34 (dd, $J$ = 8.1, 1.5 Hz, 1H), 7.27 – 7.24 (m, 1H), 7.18 (s, 1H), 6.92 (td, $J$ = 7.7, 1.6 Hz, 1H). $^{13}$C NMR (101 MHz, $CDCl_3$) δ 135.8, 135.1, 134.6, 132.7, 132.0, 129.5, 129.3, 129.1, 129.1, 128.6, 128.0, 127.6, 126.5, 122.7, 122.2, 115.8. HRMS (ESI-TOF) m/z: $[M + H]^+$ calcd for $C_{16}H_{13}BrNO_2S$, 361.9845; found, 361.9858.

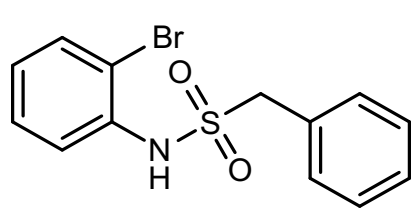


**11aq**

N-(2-Bromophenyl)-1-phenylmethanesulfonamide (**11aq**). Prepared according to General Procedure D. Using 2-bromoaniline (418 mg, 2.43 mmol, 1.0 equiv) afforded the title compound as a white solid (215 mg, 0.66 mmol, 27% yield). $^1$H NMR (400 MHz, $CDCl_3$) δ 7.66 (dd, $J$ = 8.3, 1.5 Hz, 1H), 7.55 (dd, $J$ = 8.0, 1.5 Hz, 1H), 7.38 – 7.27 (m, 4H), 7.25 – 7.18 (m, 2H), 7.05 – 6.98 (m, 1H), 6.71 (s, 1H), 4.38 (s, 2H). $^{13}$C NMR (101 MHz, $CDCl_3$) δ 135.4, 133.1, 130.9, 129.2, 129.1, 129.1, 128.0, 125.7, 119.8, 114.0, 58.2. HRMS (ESI-TOF) m/z: $[M + H]^+$ calcd for $C_{13}H_{13}BrNO_2S$, 325.9845; found, 325.9859.

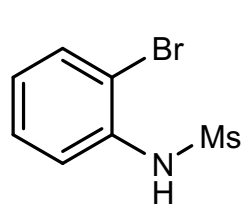


**11ar**

N-(2-Bromophenyl) methane-sulfonamide (**11ar**). Prepared according to General Procedure D. Using 2-bromoaniline (1.00 g, 5.81 mmol, 1.0 equiv) afforded the title compound as a white solid (1.10 g, 4.40 mmol, 76% yield). $^1$H NMR (400 MHz, $CDCl_3$) δ 7.59 (ddd, $J$ = 23.0, 8.1, 1.5 Hz, 2H), 7.35 – 7.28 (m, 1H), 7.05 (m, $J$ = 7.6, 1.6 Hz, 1H), 6.92 (s, 1H), 3.00 (s, 3H). $^{13}$C NMR (101

MHz, $CDCl_3$) δ 134.8, 132.9, 128.9, 126.8, 123.0, 115.8, 40.0. HRMS (ESI-TOF) m/z: $[M + H]^+$ calcd for $C_7H_9BrNO_2S$, 249.9532; found, 249.9544.

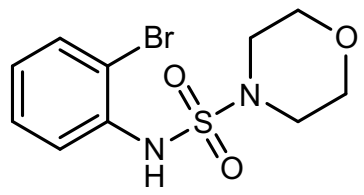

**11as**

N-(2-Bromophenyl) morpholine-4-sulfonamide (**11as**). Prepared according to General Procedure D, with slight modification. DMAP (0.10 equiv) was added, and the resulting reaction mixture was heated at 120 °C and stirred for 16 hours. Using 2-bromoaniline (200 mg, 1.16 mmol, 1.0 equiv) afforded the title compound as a yellow oil (153 mg, 0.477 mmol, 41% yield). $^1$H NMR (400 MHz, $CDCl_3$) δ 7.66 (dd, *J* = 8.2, 1.5 Hz, 1H), 7.54 (dd, *J* = 8.1, 1.5 Hz, 1H), 7.33 – 7.27 (m, 1H), 7.06 – 6.96 (m, 1H), 6.83 (s, 1H), 3.65 – 3.60 (m, 4H), 3.25 – 3.20 (m, 4H). $^{13}$C NMR (101 MHz, $CDCl_3$) δ 135.4, 132.9, 128.7, 125.9, 121.5, 114.3, 66.2, 46.5. HRMS (ESI-TOF) m/z: $[M + H]^+$ calcd for $C_{10}H_{14}BrN_2O_3S$, 320.9903; found, 320.9915.

**Synthetic procedure for diversification of the heterocyclic product.**

*Synthesis of the C10 hydroxylated product **18** via Pd C–H hydroxylation:*

*Part 1) Synthesis of the carboxylic acid **17** via methyl ester hydrolysis:*

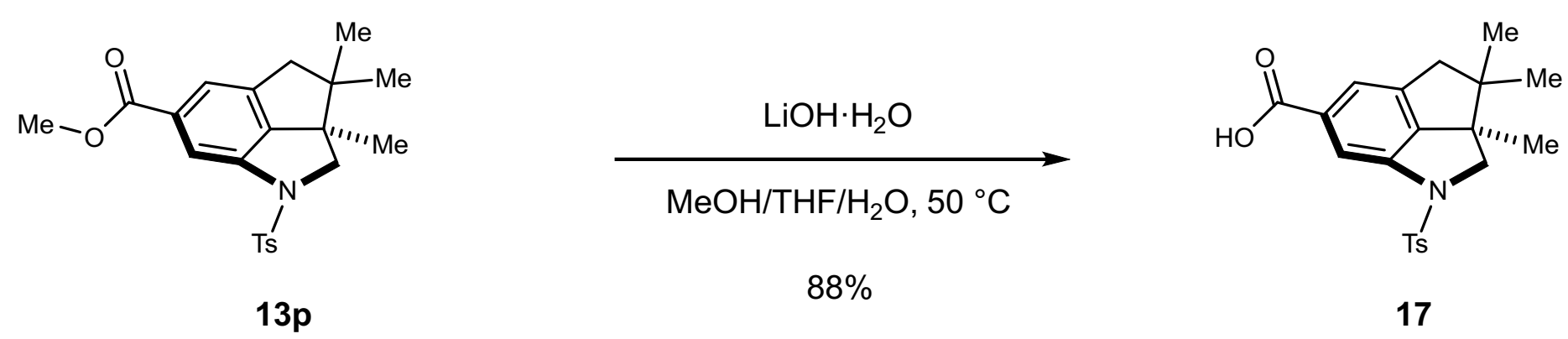


Lithium hydroxide monohydrate (21 mg, 0.51 mmol, 3.0 equiv) was added to a solution of methyl ester **13p** (68 mg, 0.17 mmol, 1.0 equiv) in methanol-tetrahydrofuran-water (5.6 mL, v/v/v= 6:3:1). The flask was placed in an oil bath preheated to 60 °C and stirred for 24 hours. Upon complete conversion of ester as indicated by TLC analysis, the reaction mixture was cooled and diluted with water (30 mL). The mixture was transferred to a separatory funnel, acidified to pH ~ 2 with 1 M hydrochloric acid, and extracted with ethyl acetate (3 × 10 mL). The combined organic layers were washed with brine (20 mL) and dried over anhydrous sodium sulfate. The dried solution was filtered, and the filtrate was concentrated. The residue obtained was purified by flash-column chromatography (eluting with 2% ethyl acetate–hexanes initially) to afford the carboxylic acid **17** (56 mg, 88%) as a white foam.

$^1$H NMR (500 MHz, $CDCl_3$) δ 7.95 (s, 1H), 7.74 (d, *J* = 7.9 Hz, 2H), 7.59 (s, 1H), 7.28 – 7.26 (m, 2H), 3.82 (d, *J* = 10.1 Hz, 1H), 3.72 (d, *J* = 10.1 Hz, 1H), 3.28 (d, *J* = 15.4 Hz, 1H), 2.47 (d, *J* = 15.5 Hz, 1H), 2.38 (s, 3H), 1.17 (s, 3H), 1.12 (s, 3H), 0.60 (s, 3H). $^{13}$C NMR (101 MHz, $CDCl_3$) δ 171.2, 156.2, 144.4, 138.7, 138.4, 133.9, 131.1, 129.9, 127.5, 123.3, 114.4, 62.7, 51.5, 51.2, 50.1, 25.6, 23.7, 21.7, 20.7. HRMS (ESI-TOF) m/z: [M + H]+ calcd for $C_{21}H_{24}NO_4S$, 386.1421; found, 386.1418.

*Part 2) Synthesis of the C10 hydroxylated product **18** via Pd-Catalyzed C-H Hydroxylation of **17**:*

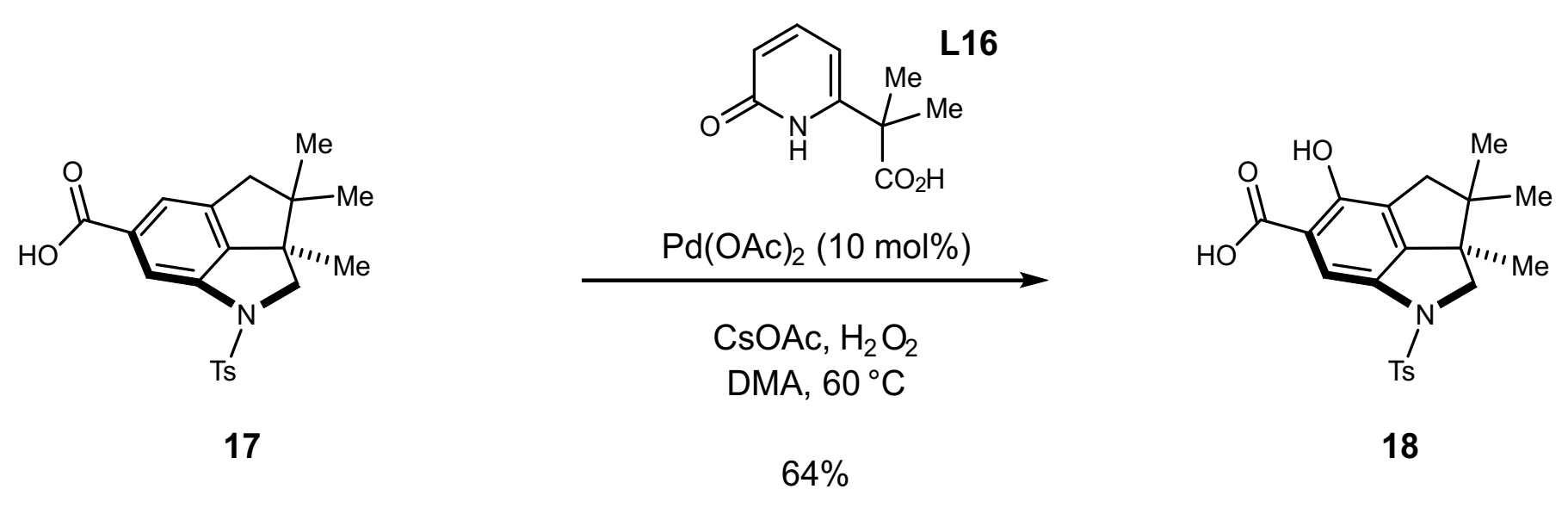


Hydrogen peroxide (50% aqueous solution, 9.54 µL, 156 µmol, 3.00 equiv) was added to a solution of carboxylic acid **17** (20.0 mg, 51.9 µmol, 1.00 equiv), palladium(II) acetate (582 µg, 2.59 µmol, 0.05 equiv), CarboxPyridone ligand **L16**[9] (0.94 mg, 5.19 µmol, 0.10 equiv), and cesium acetate (14.9 mg, 77.8 µmol, 1.50 equiv) in *N,N*-dimethylacetamide (0.53 mL, 0.10 M) at r.t. The reaction

mixture was stirred for 5 min at r.t. and subsequently placed to an oil bath that had been preheated to 60 °C. The reaction was stirred at 60 °C for 24 hours. The reaction mixture was then cooled to r.t. and quenched by addition of saturated aqueous sodium sulfite solution until complete consumption of hydrogen peroxide. The mixture was diluted sequentially with methanol (1 mL) and water (1 mL), and acidified with formic acid (0.5 mL). The resulting mixture was filtered through a pad of Celite, washing with ethyl acetate. The filtrate was transferred to a separatory funnel and the layers that formed were separated. The aqueous layer was extracted with ethyl acetate (3 × 5mL). The organic layers were combined, and the combined organic layers were dried over sodium sulfate. The dried solution was filtered and the filtrate was concentrated. The residue obtained was purified by flash-column chromatography (eluting with 35% ethyl acetate–hexanes initially) to provide **18** as amorphous solids (26 mg, 64% yield).

$^{1}$H NMR (400 MHz, CDCl3) δ 10.76 (s, 1H), 7.79 – 7.71 (m, 3H), 7.29 – 7.26 (m, 2H), 3.77 (d, *J* = 10.2 Hz, 1H), 3.64 (d, *J* = 10.1 Hz, 1H), 3.13 (d, *J* = 15.6 Hz, 1H), 2.62 (d, *J* = 15.7 Hz, 1H), 2.39 (s, 3H), 1.16 (s, 3H), 1.03 (s, 3H), 0.66 (s, 3H). $^{13}$C NMR (101 MHz, $CDCl_3$) δ 174.2, 161.2, 157.8, 144.3, 133.9, 130.0, 129.9, 127.5, 125.0, 114.9, 111.0, 61.9, 52.1, 51.2, 47.2, 25.7, 23.6, 21.7, 20.7. HRMS (ESI-TOF) m/z: [M + H]+ calcd for $C_{21}H_{24}NO_5S$, 402.1370; found, 402.1379.

*Synthesis of **20** via nitrogen atom deletion reaction:*

*Part 1) Synthesis of the free amine **19** via acid-mediated deprotection:*

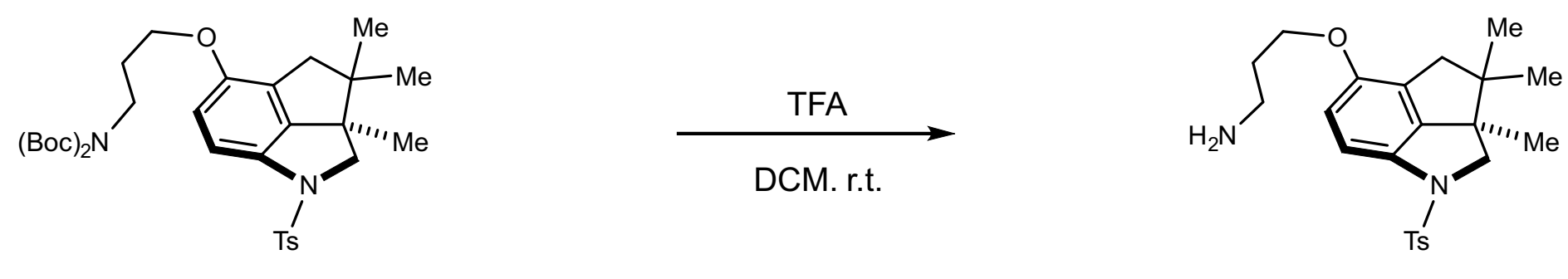


Trifluoroacetic acid (501 µL, 6.50 mmol, 65.0 equiv) was added to a solution of **13m** (62 mg, 0.10 mmol, 1.00 equiv) in dichloromethane (1.0 mL, 0.10M). The reaction was allowed to stir at r.t. for 12 hours. The product mixture was then diluted sequentially with dichloromethane, water, and saturated sodium bicarbonate solution. The resulting biphasic mixture was transferred to a separatory funnel and the layers that formed were separated. The aqueous layer was extracted with dichloromethane (2 × 10 mL). The organic layers were combined and the combined organic layers were washed with saturated aqueous sodium chloride solution (10 mL). The washed organic layer was dried over sodium sulfate. The dried solution was filtered, and the filtrate was concentrated. The residue obtained was used directly without any further purification.

*Part 2) Synthesis of **20** via nitrogen-atom deletion with Levin's reagent:*

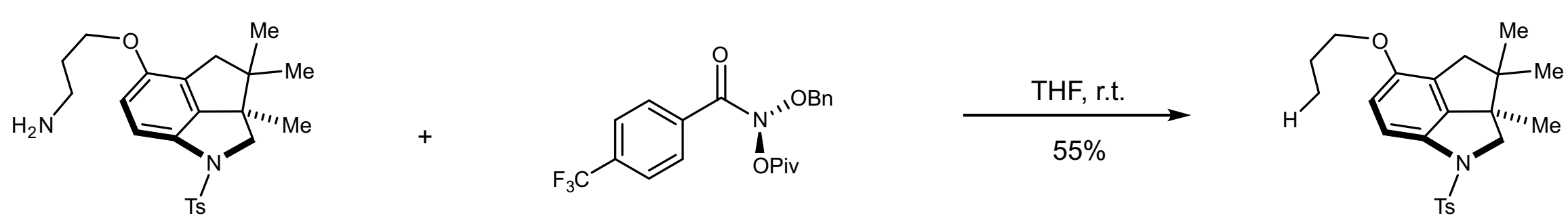


The amine residue **19** obtained from the previous step (nominally, 1.00 equiv) was added to a solution of the Levin's reagent **21**[10] (46 mg, 0.12 mmol, 1.20 equiv) in tetrahydrofuran (1.21 mL, 0.08 M) at r.t. under a nitrogen atmosphere, following a modified reported procedure[10]. The reaction mixture was stirred at r.t. for 1 hour. The reaction mixture was diluted sequentially with saturated aqueous sodium bicarbonate solution (10 mL) and dichloromethane (10 mL). The resulting biphasic mixture was transferred to a separatory funnel and the layers that formed were separated. The aqueous layer was extracted with dichloromethane (10 mL). The organic layers were combined, and dried over sodium sulfate. The dried solution was filtered and the filtrate was concentrated. The residue obtained was purified by flash-column chromatography (eluting with 5% ethyl acetate–hexanes) initially to provide the deamination product **20** as amorphous solids (22.0 mg, 55% yield).

$^{1}$H NMR (400 MHz, $CDCl_3$) δ 7.70 (d, 2H), 7.23 (d, *J* = 8.0 Hz, 2H), 7.11 (d, *J* = 8.2 Hz, 1H), 6.54 (d, *J* = 8.3 Hz, 1H), 3.96 – 3.81 (m, 2H), 3.79 (d, *J* = 10.3 Hz, 1H), 3.59 (d, *J* = 10.3 Hz, 1H), 3.14 (d, *J* = 15.3 Hz, 1H), 2.55 (d, *J* = 15.4 Hz, 1H), 2.37 (s, 3H), 1.77 – 1.69 (m, 2H), 1.11 (s, 3H), 0.99 (t, *J* = 7.4 Hz, 3H), 0.96 (s, 3H), 0.62 (s, 3H). $^{13}$C NMR (101 MHz, $CDCl_3$) δ 153.3, 152.5,

143.9, 134.4, 129.7, 127.5, 125.3, 114.2, 113.9, 71.2, 62.6, 51.0, 48.6, 25.8, 23.7, 23.0, 22.8, 21.7, 21.2, 10.6. HRMS (ESI-TOF) m/z: $[M + H]^+$ calcd for $C_{23}H_{30}NO_3S$, 400.1941; found, 400.1945.

*Synthesis of the C10 aminated product **22** via a palladium-catalyzed C–N cross-coupling:*

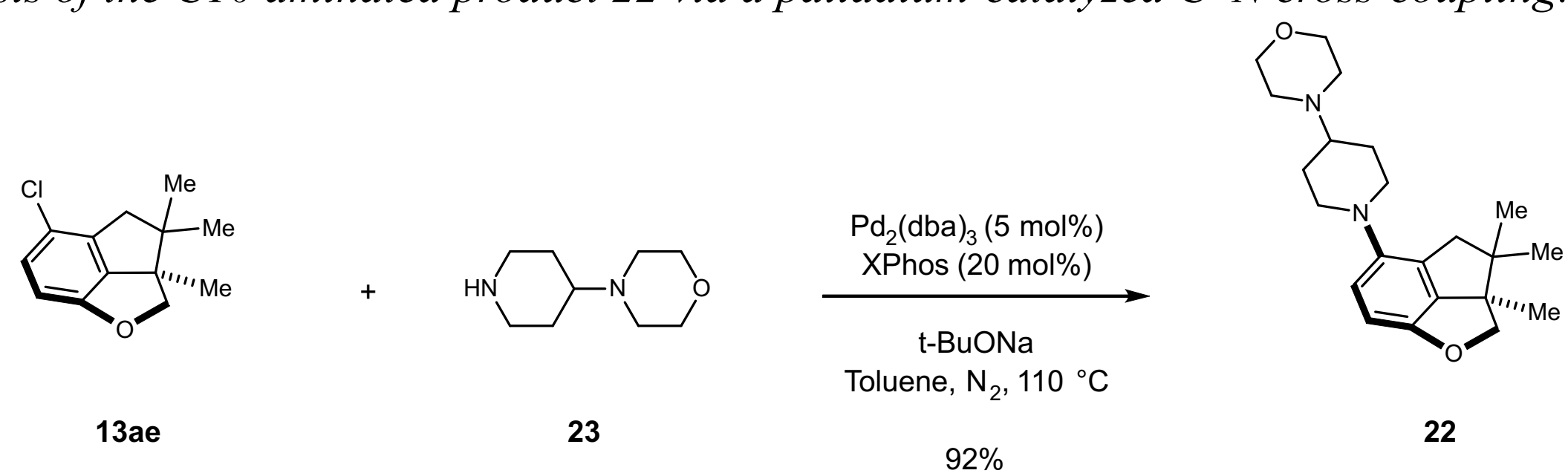


**13ae** **23** **22**

A screw-capped 16 × 125 mm culture tube was sequentially charged under air with the aryl chloride **13ae** (22 mg, 0.10 mmol, 1.0 equiv), the amine **23** (34 mg, 0.20 mmol, 2.0 equiv), $Pd_2(dba)_3$ (4.6 mg, 5.0 μmol, 0.05 equiv), XPhos ligand (9.5 mg, 20.0 μmol, 0.20 equiv), and sodium *tert*-butoxide (19 mg, 0.20 mmol, 2.0 equiv)[11]. The reaction vessel was then sealed using a screw cap with a teflon coated septa. The reaction vessel was then placed under nitrogen atmosphere by using a vacuum manifold with a process of evacuating the headspace for 1.0 minute and venting with nitrogen for five times. Next, toluene (1.0 mL) was added via a syringe to the reaction mixture at r.t. The reaction vessel was then placed into an oil bath that had been preheated to 110 °C. The reaction mixture was allowed to stir for 12 hours at 110 °C. After being allowed to cool to room temperature, the product mixture was diluted with ethyl acetate (10 mL) and water (10 mL). The resulting biphasic mixture was transferred to a separatory funnel and the layers that formed were separated. The aqueous layer was extracted with ethyl acetate (3 × 10 mL). The organic layers were combined, and the combined organic layers were washed with saturated aqueous sodium chloride solution (10 mL). The washed organic layer was dried over sodium sulfate. The dried solution was filtered and the filtrate was concentrated. The residue obtained was purified by flash-column chromatography (eluting with 20% ethyl acetate–hexanes initially) to provide the tricyclic products **22** as amorphous solids (33.0 mg, 92%).

$^1H$ NMR (400 MHz, $CDCl_3$) δ 6.43 (d, $J$ = 8.1 Hz, 1H), 6.35 (d, $J$ = 8.1 Hz, 1H), 4.55 (d, $J$ = 8.7 Hz, 1H), 4.25 (d, $J$ = 8.7 Hz, 1H), 3.77 (s, 4H), 3.48 – 3.36 (m, 2H), 3.32 (d, $J$ = 14.9 Hz, 1H), 2.70 – 2.54 (m, 7H), 1.98 – 1.85 (m, 2H), 1.71 – 1.61 (m, 2H), 1.38 (s, 3H), 1.20 (s, 3H), 0.87 (s, 3H). $^{13}C$ NMR (101 MHz, $CDCl_3$) δ 151.5, 149.6, 144.2, 130.5, 114.6, 107.5, 85.0, 67.2, 62.2, 53.2, 52.8, 51.9, 51.7, 49.7, 48.8, 29.7, 28.4, 28.2, 26.2, 24.1, 20.7. HRMS (ESI-TOF) m/z: [M + H]+ calcd for $C_{22}H_{33}N_2O_2$, 357.2537; found, 357.2543

*Synthesis of the free amine product **24** via an acid-mediated deprotection reaction:*

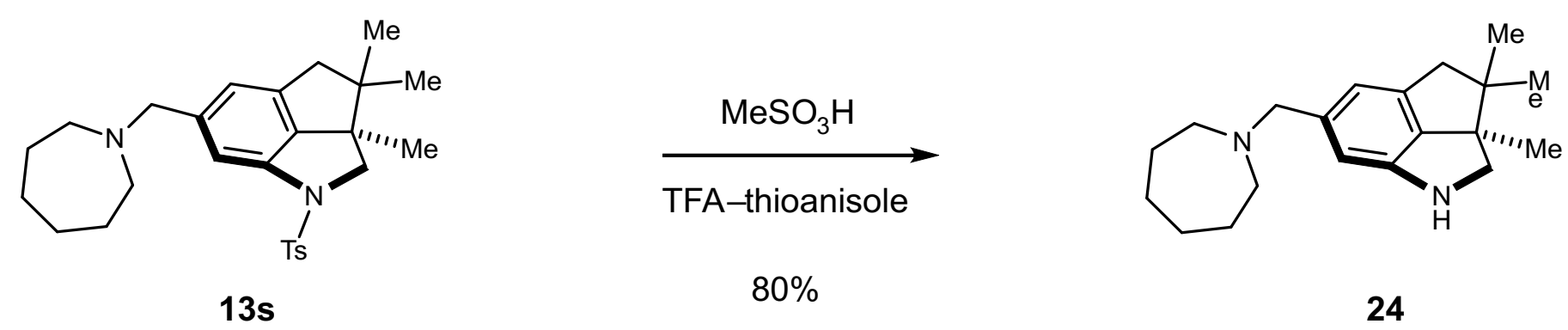

Methanesulfonic acid (13.0 μL, 0.20 mmol) was added in portion to a solution of **13s** (45 mg, 0.10 mmol) in trifluoroacetic acid/thioanisole (1.0 mL, 9:1 v/v) at r.t. The reaction was allowed to stir at r.t. for 24 hours[12]. After removal of volatiles under reduced pressure, a saturated aqueous solution of $NaHCO_3$ was added. The mixture was extracted with dichloromethane (3×10 mL). The combined organic extracts were dried over $Na_2SO_4$, filtered, and concentrated in vacuo. The dried solution was filtered, and the filtrate was concentrated. The residue obtained was purified by flash-column chromatography (eluting with 20% ethyl acetate–hexanes initially) to provide the amine substrate **24** as an oil (24.0 mg, 80%).

$^1$H NMR (400 MHz, $CDCl_3$) δ 6.49 (s, 1H), 6.41 (s, 1H), 3.74 (d, $J$ = 8.9 Hz, 1H), 3.56 (s, 2H), 3.33 (d, $J$ = 14.3 Hz, 1H), 3.16 (d, $J$ = 8.9 Hz, 1H), 2.65 (s, 4H), 2.40 (d, $J$ = 14.8 Hz, 1H), 1.61 (s, 6H), 1.31 (s, 3H), 1.19 (s, 3H), 0.83 (s, 3H). $^{13}$C NMR (101 MHz, $CDCl_3$) δ 148.3, 138.4, 116.6, 108.0, 63.5, 60.8, 55.5, 52.3, 51.8, 51.4, 27.7, 27.3, 26.8, 24.1, 19.7. HRMS (ESI-TOF) m/z: [M + H]+ calcd for $C_{20}H_{31}N_2$, 299.2482; found, 299.2480.

**Synthetic Procedure for the Preparation of Substrate 25–D and 27–D:**

*Synthesis of the deuterated linear substrate **25–D**:*

*Part 1) Ethyl ester reduction and benzoylation of **S25**:*

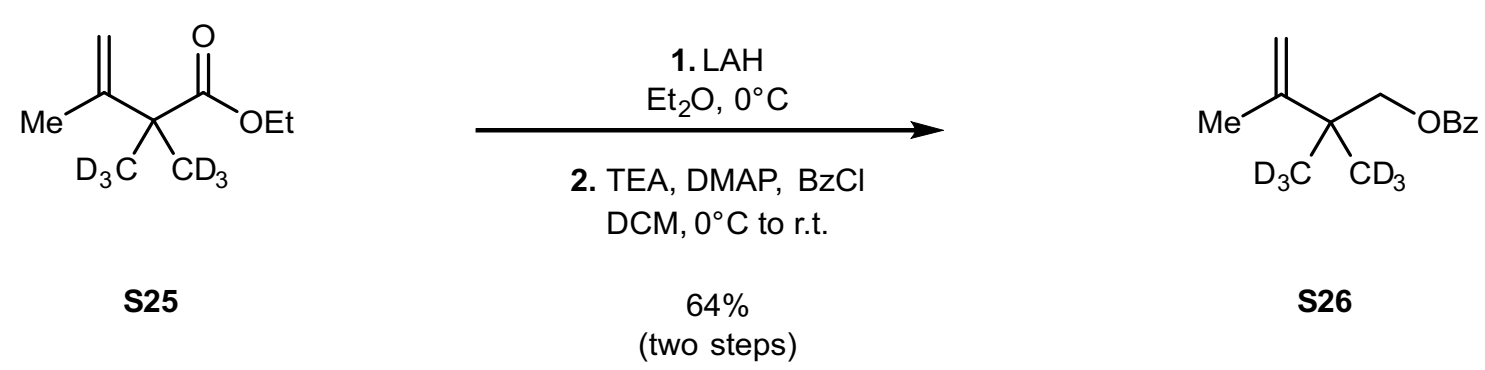


Lithium aluminum hydride (6.24 mL, 12.5 mmol, 1.50 equiv, 2.0 M in tetrahydrofuran) was added dropwise to a solution of the known ester **S25**[3] (1.35g, 8.26 mmol, 1.00 equiv) in diethyl ether (41.6 mL, 0.20M) at 0 °C under a nitrogen atmosphere. The reaction mixture was stirred at 0 °C for 30 min. The cold product mixture was then carefully quenched by a protocol developed by Fieser.[8] Water (474 µL), 5M aqueous NaOH (474 µL), and water (1.42 mL) were added sequentially and dropwise to the reaction vessel. The mixture was stirred for 5 min at room temperature, then anhydrous magnesium sulfate was added in one portion. The resulting suspension was filtered through a pad of Celite, washing with diethyl ether. The filtrate was concentrated under reduced pressure to afford the corresponding the alcohol intermediate, which was used directly in the next step without any further purification.

Triethylamine (1.28 mL, 9.15 mmol, 1.10 equiv), 4-dimethylaminopyridine (305 mg, 2.50 mmol, 0.30 equiv), and benzoyl chloride (1.06 mL, 9.15 mmol, 1.10 equiv) were added in sequence to the alcohol residue obtained in the previous step (nominally, 1.00 equiv) in dichloromethane (16.6 mL, 0.50M) at 0 °C. The reaction mixture was slowly warmed to r.t. and allowed to stir 16 hours at r.t. The product mixture was then quenched with saturated aqueous ammonium chloride solution (40 mL). The resulting biphasic mixture was transferred to a separatory funnel and the layers that formed were separated. The aqueous layer was extracted with dichloromethane (3 × 40 mL). The combined organic layer was washed with 1M hydrochloric acid (100 mL) and was dried over sodium sulfate. The dried solution was filtered, and the filtrate was concentrated. The residue obtained was purified by flash-column chromatography (eluting with 5% ethyl acetate–hexanes initially) to provide the product **S26** as a colorless oil (654 mg, 64% yield over two steps).

$^{1}$H NMR (400 MHz, $CDCl_3$) δ 7.95 – 7.80 (m, 2H), 7.44 – 7.36 (m, 1H), 7.32 – 7.23 (m, 2H), 4.75 – 4.68 (m, 2H), 4.04 (s, 2H), 1.71 – 1.59 (m, 3H). $^{13}$C NMR (101 MHz, $CDCl_3$) δ 166.7, 149.5, 133.0, 130.6, 129.7, 128.5, 110.9, 71.5, 23.5, 19.8. HRMS (ESI-TOF) m/z: $[M + H]^+$ calcd for $C_{14}H_{13}D_6O_2$, 225.1756; found, 225.1760.

*Part 2) Synthesis of deuterated allylic alcohol **S27**:*

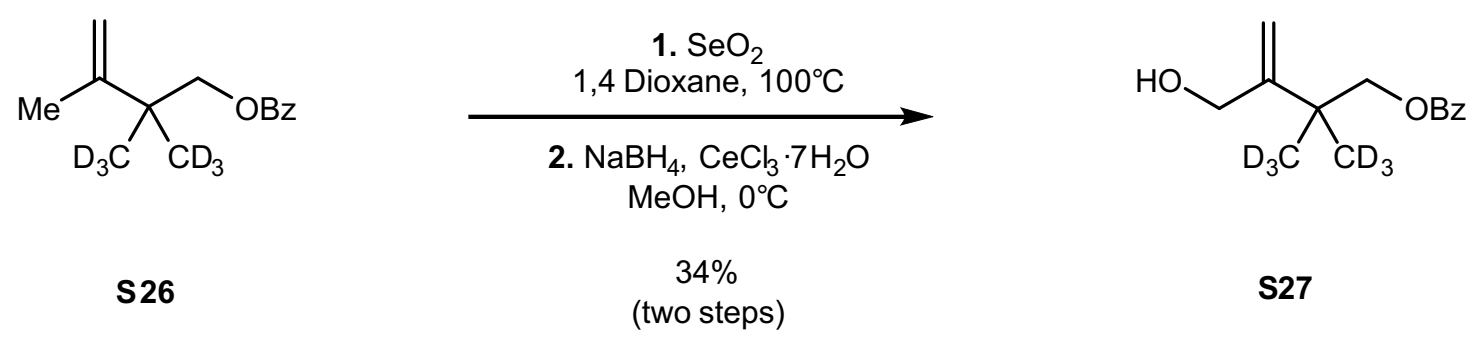


Selenium dioxide (604 mg, 5.44 mmol, 1.00 equiv) was added to a solution of benzoyl protected alcohol **S26** (654 mg, 5.44 mmol, 1.00 equiv) in 1,4-dioxane (18.1 mL, 0.30M). The reaction mixture was then placed to an oil bath that had been preheated to 100 °C and stirred for 1 hour at 100 °C. The reaction was cooled to room temperature. The cooled reaction mixture was filtered through a pad of Celite, and the filtrate was diluted sequentially with diethyl ether (40 mL). The organic layer was washed with saturated aqueous sodium bicarbonate solution (40 mL). The washed organic layer was dried over anhydrous sodium sulfate. The dried solution was filtered and the filtrate was concentrated under reduced pressure to afford a thick residue containing the corresponding aldehyde intermediate, which was used directly in the next step without further purification.

Cerium(III) chloride heptahydrate (2.43 g, 6.53 mmol, 1.20 equiv) was added to a solution of the residue obtained in the previous step (nominally, 1.00 equiv) in methanol (15.5 mL, 0.35M) at 0 °C. Sodium borohydride (208 mg, 5.49 mmol, 1.01 equiv) was then added portion wise at 0 °C. The reaction mixture was stirred at 0 °C until complete consumption of the starting material. The cold product mixture was diluted sequentially with water (40 mL), 1 M hydrochloric acid (20 mL), and dichloromethane (40 mL). The resulting biphasic mixture was transferred to a separatory funnel and the layers that formed were separated. The aqueous layer was extracted with dichloromethane (3 × 40 mL). The combined organic layer was dried over sodium sulfate. The dried solution was filtered and the filtrate was concentrated. The residue obtained was purified by flash-column chromatography (eluting with 15% ethyl acetate–hexanes initially) to provide the allylic alcohol **S27** (440 mg, 34% yield over two steps) as a colorless oil.

$^1$H NMR (400 MHz, $CDCl_3$) δ 8.07 – 7.95 (m, 2H), 7.59 – 7.53 (m, 1H), 7.47 – 7.41 (m, 2H), 5.26 – 5.20 (m, 1H), 5.08 (d, $J$ = 1.0 Hz, 1H), 4.26 (s, 2H), 4.25 – 4.23 (m, 2H). $^{13}$C NMR (101 MHz, $CDCl_3$) δ 166.8, 152.9, 133.1, 130.4, 129.7, 128.6, 111.7, 71.9, 64.0, 38.7, 23.7. HRMS (ESI-TOF) m/z: $[M + H]^+$ calcd for $C_{14}H_{13}D_6O_3$, 241.1706; found, 241.1717.

*Part 3) Synthesis of deuterated allylic bromide **S28** via bromination of the alcohol **S27**:*

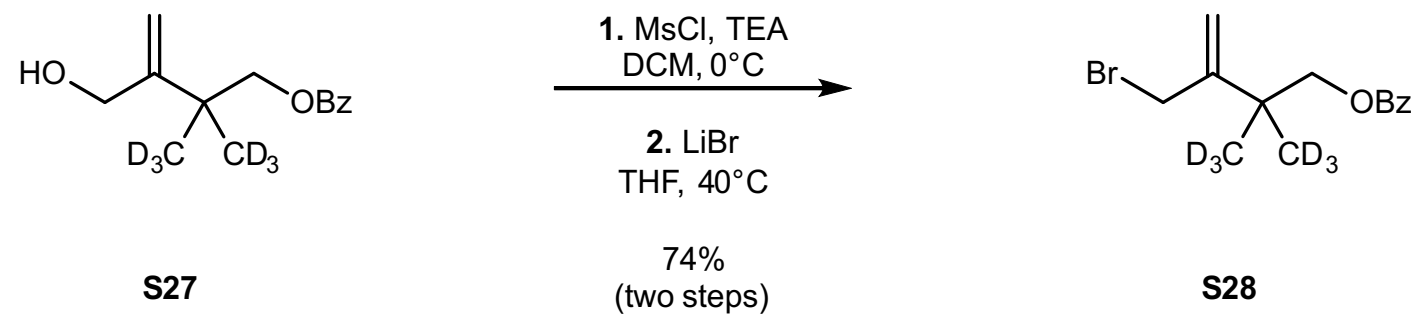

Triethylamine (357 μL, 2.56 mmol, 1.4 equiv) was added dropwise to a stirred solution of the allylic alcohol **S27** (440 mg, 1.83 mmol, 1.0 equiv) and methanesulfonyl chloride (171 μL, 2.20 mmol, 1.2 equiv) in dichloromethane (9.15 mL, 0.2 M) at 0 °C. The reaction mixture was stirred at 0 °C for 30 min. The reaction mixture was then sequentially diluted with diethyl ether (40 mL) and with water (20 mL). The resulting biphasic mixture was transferred to a separatory funnel and the layers were separated. The organic layer was washed with 1 M hydrochloric acid (20 mL), water (20 mL), and saturated aqueous sodium chloride solution (20 mL). The washed organic layer was dried over anhydrous sodium sulfate. The dried solution was filtered and the filtrate was concentrated under reduced pressure to afford a residue containing the corresponding mesylate, which was used directly in the next step without further purification.

Lithium bromide (167 mg, 1.92 mmol, 1.05 equiv) was added in one portion to a solution of the residue obtained in the previous step (nominally, 1.0 equiv) in tetrahydrofuran (2.61 mL, 0.7 M). The reaction mixture was heated at 40 °C and stirred for 30 min. The cooled reaction mixture was diluted with water (10 mL) and diethyl ether (20 mL) and transferred to a separatory funnel. The layers were separated. The organic layer was washed with 1M hydrochloric acid solution (10 mL), saturated aqueous sodium bicarbonate solution (10 mL), and saturated aqueous sodium chloride solution (10 mL), dried over sodium sulfate, filtered, and concentrated. The residue obtained was purified by flash-column chromatography (eluting with 5% ethyl acetate–hexanes initially) to provide the deuterated allylic bromide **S28** as a light-yellow liquid (410 mg, 1.35 mmol, 74% yield over two steps).

$^{1}$H NMR (500 MHz, $CDCl_3$) δ 8.06 – 7.99 (m, 2H), 7.60 – 7.52 (m, 1H), 7.48 – 7.40 (m, 2H), 5.45 (s, 1H), 5.31 (s, 1H), 4.26 (s, 2H), 4.10 (s, 2H). $^{13}$C NMR (126 MHz, $CDCl_3$) δ 166.5, 149.5, 133.1, 130.4, 129.7, 128.6, 118.9, 71.7, 39.4, 32.7, 24.1. HRMS (ESI-TOF) m/z: $[M + H]^+$ calcd for $C_{14}H_{12}D_6BrO_2$, 303.0862; found, 303.0877.

*Part 4) Synthesis of the deuterated linear substrate **25–D**:*

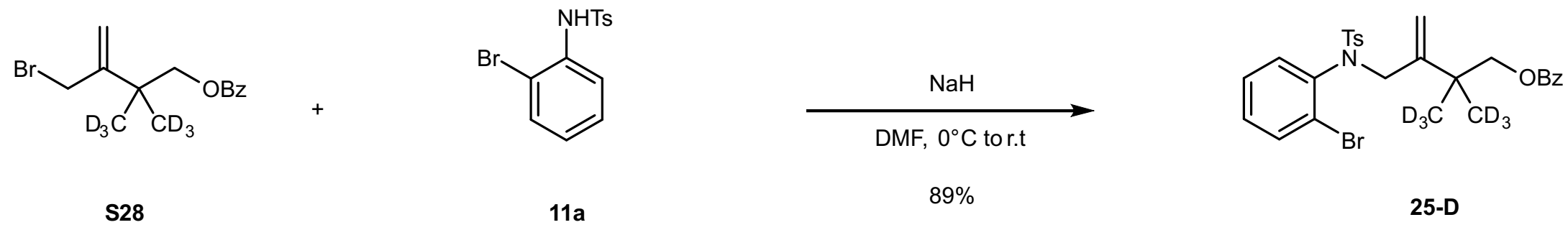


Sodium hydride (15.8 mg, 396 μmol, 1.20 equiv, 60% dispersion in mineral oil) was added to a solution of 2-bromosulfonamide **11a** (108 mg, 330 μmol, 1.00 equiv) in *N,N*-dimethylformamide (3.30 mL, 0.10 M) at 0 °C. The mixture was stirred at 0 °C for 1 hour. The deuterated allylic bromide **S28** (100 mg, 330 μmol, 1.00 equiv) was then added in one portion to reaction mixture at 0 °C. The reaction mixture was stirred for 16 hours at 0 °C. The product mixture was subsequently diluted sequentially with water (10 mL) and ethyl acetate (10 mL). The resulting biphasic mixture was transferred to a separatory funnel and the layers that formed were separated. The aqueous layer was extracted with ethyl acetate (3 × 10 mL). The organic layers were combined, and the combined organic layers were washed with saturated aqueous ammonium chloride solution (30 mL), water (30 mL), and saturated aqueous sodium chloride solution (30 mL). The washed organic layer was dried over sodium sulfate. The dried solution was filtered and the filtrate was concentrated. The residue obtained was purified by flash-column chromatography (eluting with 5% ethyl acetate–hexanes initially) to provide the deuterated linear substrate **25–D** (161 mg, 89.0% yield) as amorphous solids.

$^1$H NMR (400 MHz, $CDCl_3$) δ 7.89 (d, 2H), 7.57 – 7.52 (m, 3H), 7.45 – 7.37 (m, 3H), 7.31 – 7.26 (m, 1H), 7.22 (d, *J* = 8.1 Hz, 2H), 7.16 (td, *J* = 7.7, 1.5 Hz, 1H), 7.02 (td, *J* = 7.7, 1.7 Hz, 1H), 5.44 – 5.39 (m, 1H), 5.21 – 5.15 (m, 1H), 4.48 – 4.31 (m, 2H), 4.06 (s, 2H), 2.41 (s, 3H). $^{13}$C NMR (101 MHz, $CDCl_3$) δ 166.4, 146.9, 143.7, 137.7, 136.7, 134.2, 133.7, 133.0, 130.2, 129.7, 129.7, 129.6, 128.4, 128.0, 127.8, 124.7, 114.4, 77.4, 71.2, 51.4, 38.8, 21.7. HRMS (ESI-TOF) m/z: $[M + H]^+$ calcd for $C_{27}H_{23}D_6BrNO_4S$, 548.1372; found, 548.1381.

*Synthesis of the deuterated linear substrate **27–D**:*

*Part 1) Synthesis of deuterated 1-Bromo-2-nitrobenzene **S29-D**:*

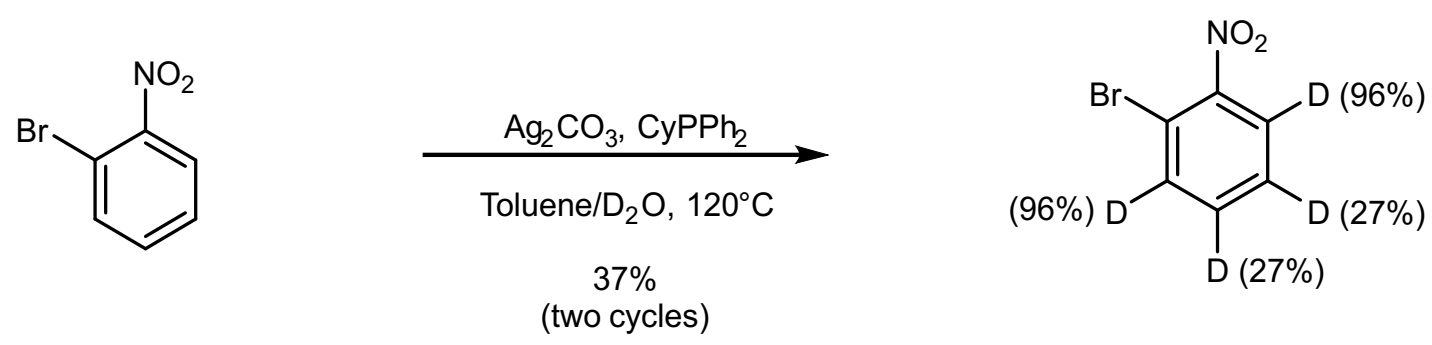


Silver carbonate (328 mg, 1.19 mmol, 0.2 equiv), and cyclohexyl(diphenyl)phosphine (638 mg, 2.38 mmol, 0.4 equiv) were added to a solution of 1-bromo-2-nitrobenzene **S29** (1.20 g, 5.94 mmol, 1.0 equiv) in toluene (60.0 μL) and deuterium oxide (2.15 mL, 119 mmol, 20 equiv). The reaction mixture was stirred at 120 °C for 12 h.[13] After completion of the reaction, the mixture was cooled to room temperature and transferred to an Erlenmeyer flask using diethyl ether. The mixture was diluted with diethyl ether (30 mL). The mixture was dried over anhydrous sodium sulfate, filtered, and concentrated under reduced pressure. Purification by flash-column chromatography (eluting with 5% ethyl acetate–hexanes initially) afforded 1-bromo-2-nitrobenzene–D as amorphous solids (833 mg, 4.04 mmol, 68% yield).

The material obtained in this way was subjected to a second round of deuteration. Specifically, 1-bromo-2-nitrobenzene–D (833 mg, 4.12 mmol, 1.0 equiv), silver carbonate (227 mg, 0.825 mmol, 0.2 equiv), and cyclohexyl(diphenyl)phosphine (443 mg, 1.65 mmol, 0.4 equiv) were dissolved in toluene (40.0 μL) and deuterium oxide (1.49 mL, 82.5 mmol, 20 equiv). The reaction mixture was stirred at 120 °C for 12 h. After completion of the reaction, the mixture was cooled to room temperature and transferred to an Erlenmeyer flask using diethyl ether. The mixture was diluted with diethyl ether (30 mL). The mixture was dried over anhydrous sodium sulfate, filtered, and concentrated under reduced pressure. Purification by flash-column chromatography (eluting with 5% ethyl acetate–hexanes initially) to provide the 1-bromo-2-nitrobenzene–D **S29-D** as amorphous solids (450 mg, 2.18 mmol, 37% yield over two deuteration cycles).

Note: We found that the deuteration at C6 and C3 are 96%, and the average deuteration of C4 and C5 is 27%. This was determined by $^{1}$H NMR of 0.1 mmol of **S29–D** in $CDCl_3$ with an internal standard of 0.1 mmol mesitylene (see $^{1}$H NMR spectrum provided below)

$^{1}$H NMR (400 MHz, $CDCl_3$) δ 7.84 (m, $J$ = 6.0, 2.8, 1.5 Hz, 0H), 7.78 – 7.69 (m, 0H), 7.53 – 7.39 (m, 1H). $^{13}$C NMR (101 MHz, $CDCl_3$) δ 150.0, 135.2, 135.2, 134.9, 134.7, 133.2, 133.1, 128.3, 128.2, 125.8, 125.7, 125.5, 125.2, 114.5. HRMS (ESI-TOF) m/z: $[M + H]^+$ calcd for $C_6HD_4BrNO_2$, 205.9749; found, 205.9745.

$^{1}$H NMR (400 MHz, $CDCl_3$) of 1-bromo-2-nitrobenzene–$D_4$ (**S29-D**) with mesitylene as internal standard.

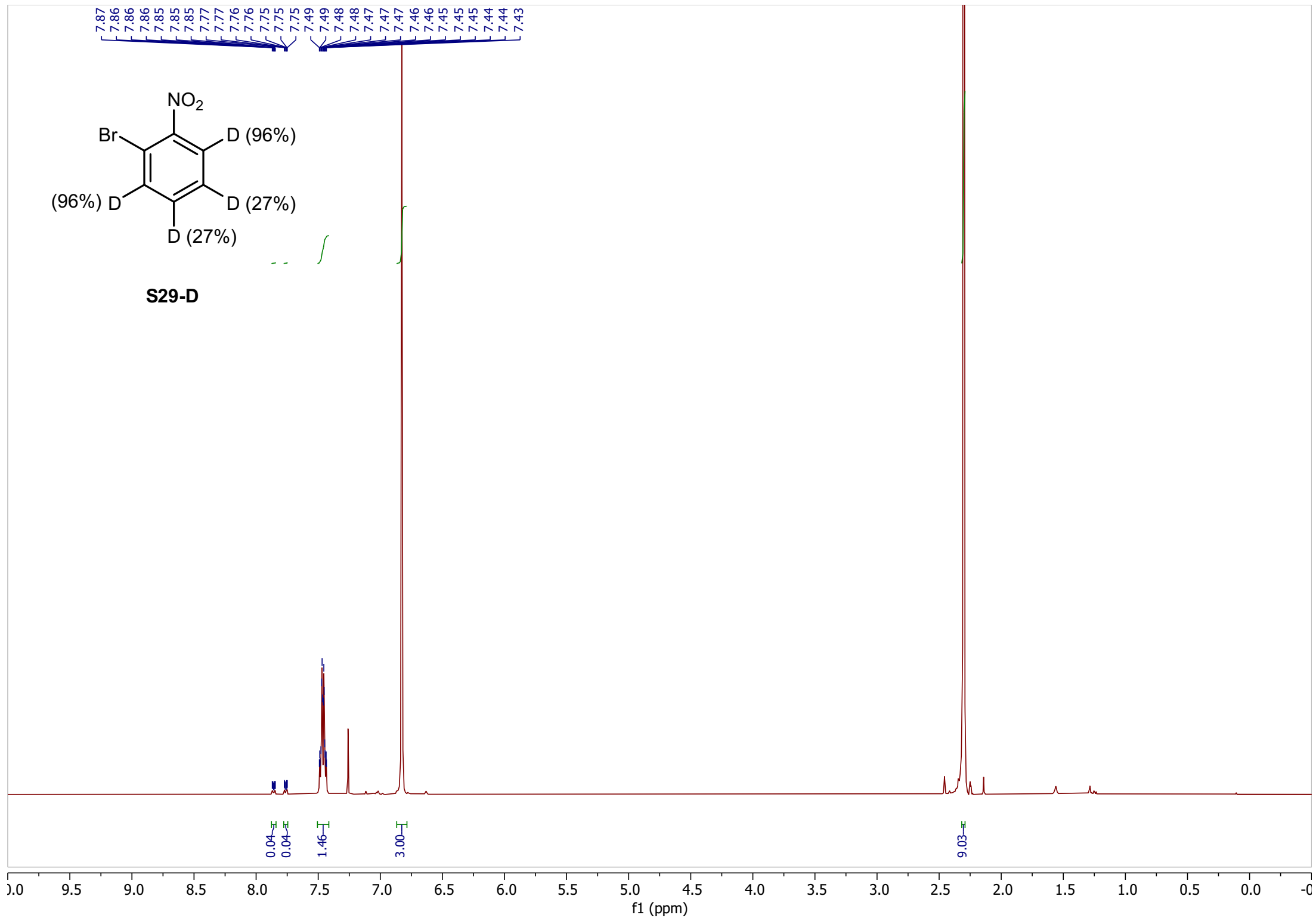

*Part 2) Synthesis of the tosyl-protected aniline **S30–D**:*

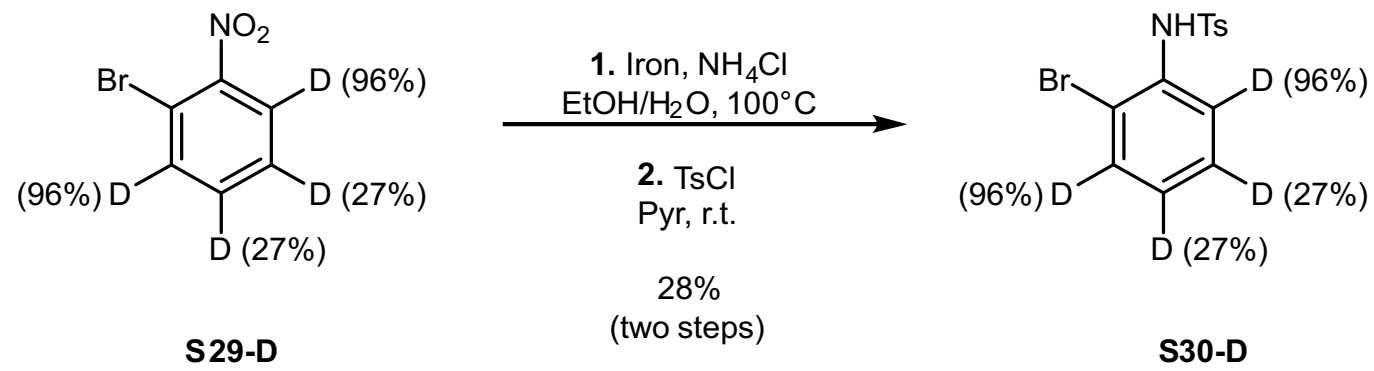


Iron powder (438 mg, 7.84 mmol, 4.0 equiv), and ammonium chloride (210 mg, 3.92 mmol, 2.0 equiv) was sequentially added to a solution of **S29-D** (400 mg, 1.96 mmol, 1.0 equiv), in ethanol (27.5 mL, 470 mmol, 240 equiv) and water (2.83 mL, 157 mmol, 80 equiv). The reaction mixture was then placed to an oil bath that had been preheated to 100 °C and stirred for 2 hours at 100 °C. The reaction mixture was cooled and then filtered through a pad of Celite, and the filtrate was concentrated under reduced pressure. The residue obtained was diluted sequentially with water and dichloromethane. The aqueous layer was extracted with dichloromethane. The organic layers were combined. The combined organic layer was dried over sodium sulfate. The dried solution was filtered and the filtrate was concentrated. The amine residue obtained was used directly in the next step without any further purification.

*Para*-toluenesulfonyl chloride (411 mg, 2.16 mmol, 1.1 equiv) was added to a solution of the residue obtained in the previous step (nominally, 1.0 equiv) in pyridine (2.80 mL, 0.70M) at r.t. The reaction mixture was stirred for 16 hours at r.t. Pyridine was removed under a stream of nitrogen for 2 hours at r.t. The residue obtained was dissolved in dichloromethane and transferred to a separatory funnel. The organic solution was washed with 1 M hydrochloric acid (20 mL). The aqueous layer was extracted with dichloromethane (3 × 20 mL). The combined organic layer was dried over sodium sulfate. The dried solution was filtered, and the filtrate was concentrated. The residue obtained was purified by flash-column chromatography (eluting with 10% ethyl acetate–hexanes initially) to provide the tosyl-protected aniline **S30–D** as amorphous solids (180 mg, 28% yield over two steps).

$^{1}$H NMR (400 MHz, $CD_3OD$) δ 7.66 – 7.55 (m, 2H), 7.52 – 7.48 (m, 0H), 7.48 – 7.45 (m, 0H), 7.28 (d, $J$ = 7.9 Hz, 3H), 7.09 – 7.03 (m, 1H), 2.39 (s, 3H). $^{13}$C NMR (101 MHz, $CD_3OD$) δ 145.2, 138.5, 136.5, 134.1, 133.9, 133.6, 130.6, 129.1, 129.0, 128.4, 128.4, 128.3, 127.9, 127.6, 119.7, 21.5. HRMS (ESI-TOF) m/z: $[M + H]^+$ calcd for $C_{13}H_9D_4BrNO_2S$, 330.0096; found, 330.0082.

*Part 3) Synthesis of the deuterated linear substrate **27–D** via alkylation of **S30–D** :*

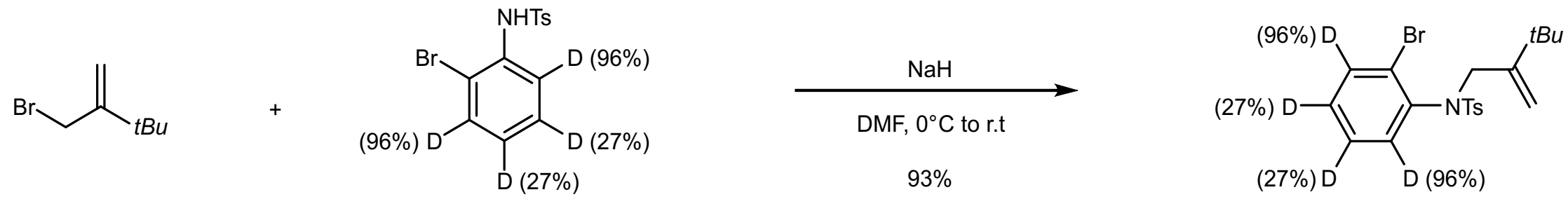


12a S30-D 27-D

Sodium hydride (10.2 mg, 254 μmol, 1.20 equiv, 60% dispersion in mineral oil) was added in one portion to a solution of tosyl-protected aniline **S30–D** (70.0 mg, 212 μmol, 1.00 equiv) in *N,N*-dimethylformamide (2.12 mL, 0.10M) at 0 °C. The mixture was stirred at 0 °C for 1 hour. Then, the *tert*-butyl bromide **12a** (37.5 mg, 212 μmol, 1.00 equiv) was added to the reaction vessel at 0 °C. The reaction mixture was stirred for 16 hours at 0 °C. The product mixture was diluted sequentially with water (10 mL) and ethyl acetate (10 mL). The resulting biphasic mixture was transferred to a separatory funnel and the layers that formed were separated. The aqueous layer was extracted with ethyl acetate (3 × 10 mL). The organic layers were combined, and the combined organic layers were washed with saturated aqueous ammonium chloride solution (30 mL), water (30 mL) and saturated aqueous sodium chloride solution (30 mL). The washed organic layer was dried over sodium sulfate. The dried solution was filtered and the filtrate was concentrated. The residue obtained was purified by flash-column chromatography (eluting with 10% ethyl acetate–hexanes initially) to provide deuterated linear substrate **27–D** (84.2 mg, 197 μmol, 93% yield) as amorphous solids.

Note: Our analysis of the deuteration levels within **27–D** did not change over the course of the reduction and tosylation sequence. Specifically, the deuteration at C6 and C3 are 96%, and the deuteration of C4 and C5 is 27%.

$^{1}$H NMR (400 MHz, $CDCl_3$) δ 7.64 – 7.53 (m, 2H), 7.54 – 7.51 (m, 0H), 7.34 – 7.30 (m, 0H), 7.29 – 7.26 (m, 1H), 7.26 – 7.23 (m, 2H), 7.18 – 7.12 (m, 1H), 5.23 – 5.17 (m, 1H), 5.06 – 5.01 (m, 1H), 4.60 – 4.11 (m, 2H), 2.42 (s, 3H), 0.95 (s, 9H). $^{13}$C NMR (101 MHz, $CDCl_3$) δ 151.1, 143.4, 137.7, 136.9, 133.7, 133.6, 129.4, 129.4, 129.3, 127.9, 127.5, 127.4, 124.5, 110.8, 51.3, 35.4, 28.7, 21.6. HRMS (ESI-TOF) m/z: $[M + H]^+$ calcd for $C_{20}H_{21}D_4BrNO_2S$, 426.1035; found, 426.1027.

## General Procedure E: Alkyl-To-Alkyl Palladium Migration-Enabled Annulation:

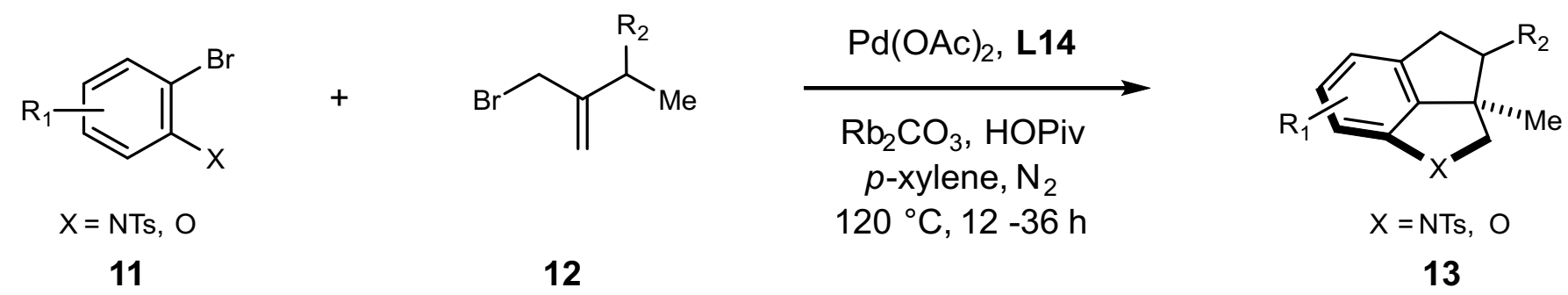


A screw-capped 16 × 125 mm culture tube was sequentially charged under air with the tosyl protected aniline or phenol **11** (0.20 mmol, 1.0 equiv), $Pd(OAc)_2$ (2.25 mg, 10 μmol, 0.05 equiv), **L14** ligand (21.1 mg, 60.0 μmol, 0.30 equiv), rubidium carbonate (139 mg, 0.60 mmol, 3.0 equiv), and pivalic acid (6.13 mg, 60.0 μmol, 0.30 equiv). The reaction vessel was then sealed using a screw cap with a teflon coated septa. The reaction vessel was then placed under nitrogen atmosphere by using a vacuum manifold with a process of evacuating the headspace for 1.0 minute and venting with nitrogen for five times. Next, the bromide **12** (0.26 mmol, 1.3 equiv) was dissolved in *para*-xylene (2.0 mL) and it was added via a syringe to the reaction mixture at r.t. The reaction vessel was then placed into an oil bath that had been preheated to 120 °C. The reaction mixture was allowed to stir for 12 or 36 hours at 120 °C. After being allowed to cool to room temperature, the product mixture was diluted with DCM and filtered through a pad of Celite. The filtrate was concentrated and the residue obtained was purified by flash-column chromatography (eluting with 5% ethyl acetate–hexanes initially) or preparative thin-layer chromatography to provide the products **13a–13as**.

## Reaction Optimization Tables.

*Optimization tables for discovery of the alkyl-to-alkyl palladium migration enables remote C–C bond formation:*

**Table S1.** Ligand Investigation Part I.[a,b]

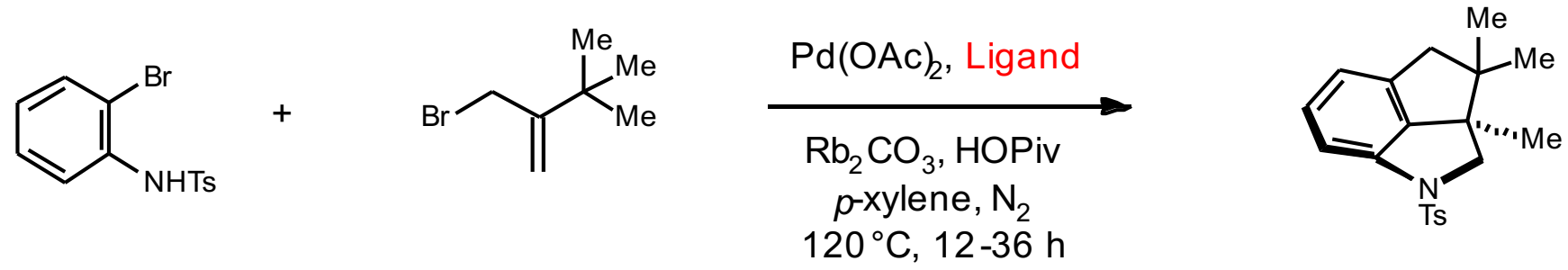


**11a** **12a** **13a**

*Ligands:*

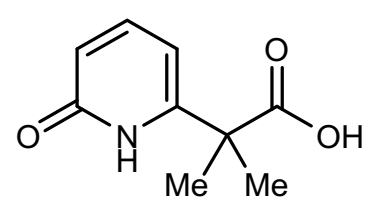


n.d.

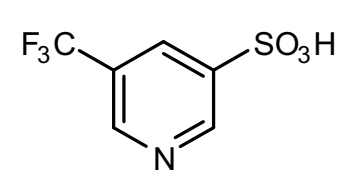


n.d.

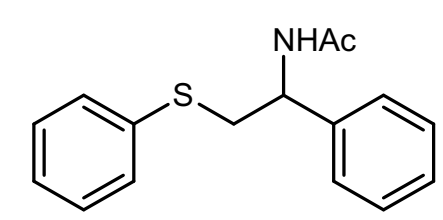


n.d.

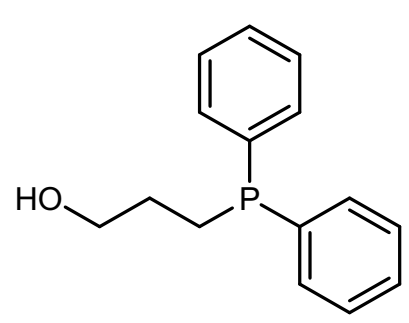


4%

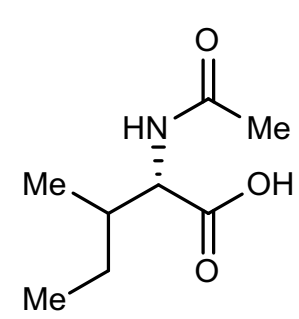


n.d.

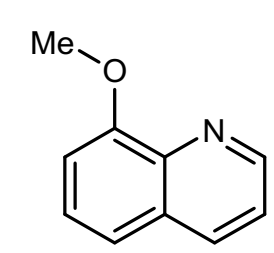


n.d.

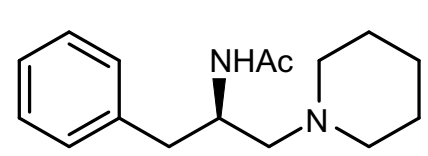


n.d.

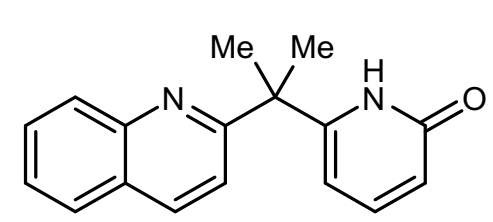


n.d.

[a]Conditions A: **11a** (0.20 mmol), **12a** (0.26 mmol), $Pd(OAc)_2$ (5 mol%), Ligand (30 mol%), $Rb_2CO_3$ (3.0 equiv), HOPiv (30 mol%), *p*-xylene (2.0 mL), $N_2$, 120 °C, 12-36 h. [b]Yields were determined by $^1H$ NMR analysis of an unpurified product mixture using $CH_2Br_2$ as an internal standard.

**Table S2.** Ligand Investigation Part II.[a,b]

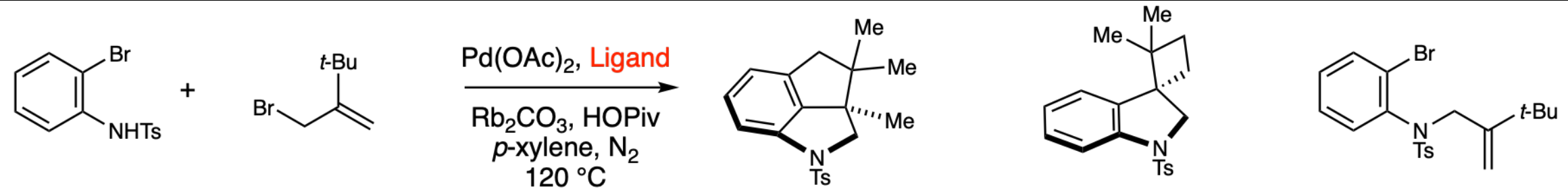


**11a** **12a** **13a** **S42** **36**

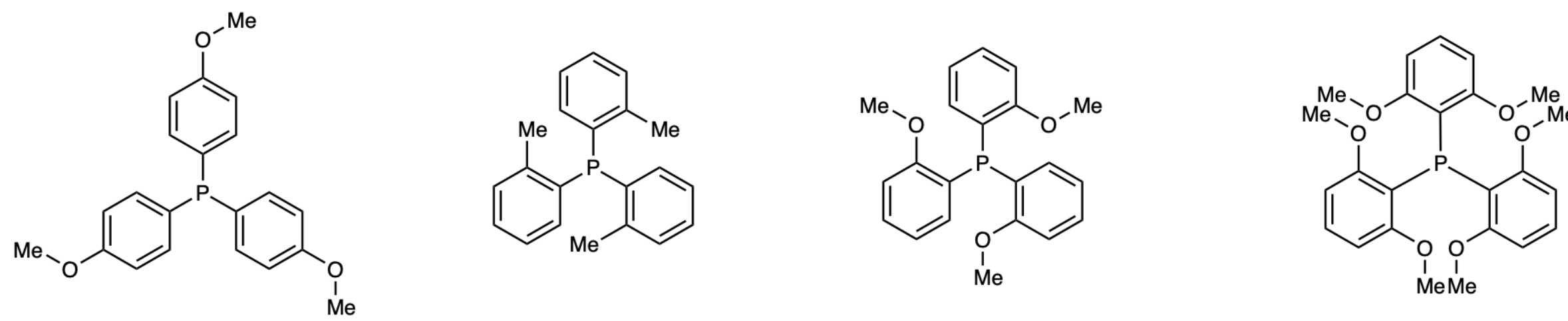


| **L9** | **L13** | **L14** | **L15** |
|---|---|---|---|
| **S42**: 42% | **S42**: 20% | **S42**: 4% | **S42**: 7% |
| **36**: n.d. | **36**: 48% | **36**: n.d. | **36**: 64% |
| **13a**: 48% | **13a**: n.d. | **13a**: 86% | **13a**: 21% |

[a]Conditions A: **11a** (0.20 mmol), **12a** (0.26 mmol), $Pd(OAc)_2$ (5 mol%), Ligand (30 mol%), $Rb_2CO_3$ (3.0 equiv), HOPiv (30 mol%), *p*-xylene (2.0 mL), $N_2$, 120 °C, 12-36 h. [b]Yields were determined by $^1H$ NMR analysis of an unpurified product mixture using $CH_2Br_2$ as an internal standard.

**Table S3.** Palladium Source Investigation.[a,b]

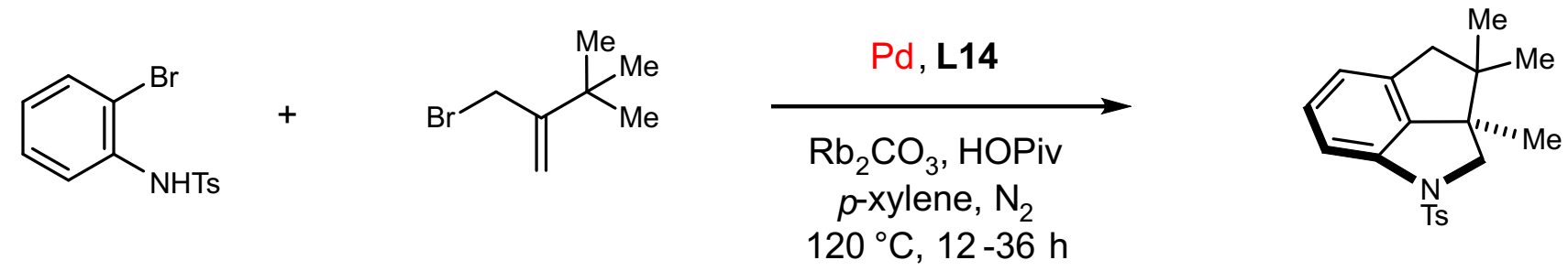


**11a** **12a** **13a**

| Entry | Pd Source | **13a** (%) |
|---|---|---|
| 1. | $Pd_2(dba)_3$ | 71 |
| 2. | $PdCl_2$ | 18 |
| 3. | $Pd(OAc)_2$ | 86 |
| 4. | $Pd(PPh_3)_4$ | 40 |
| 5. | $PdCl_2(MeCN)_2$ | 28 |

[a]Conditions A: **11a** (0.20 mmol), **12a** (0.26 mmol), Pd (5 mol%), **L14** (30 mol%), $Rb_2CO_3$ (3.0 equiv), HOPiv (30 mol%), *p*-xylene (2.0 mL), $N_2$, 120 °C, 12-36 h. [b]Yields were determined by $^1H$ NMR analysis of an unpurified product mixture using $CH_2Br_2$ as an internal standard.

**Table S4.** Investigation of Palladium Catalyst Loading.[a,b]

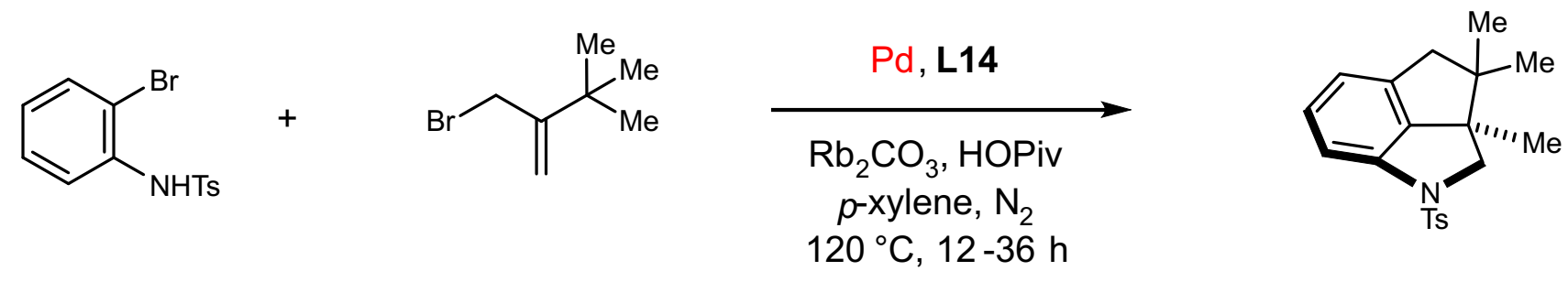


| Entry | Equivalents of $Pd(OAc)_2$ | **13a** (%) |
|---|---|---|
| 1. | 2 mol% | 34 |
| 2. | 5 mol% | 86 |
| 3. | 10 mol% | 48 |
| 4. | 20 mol% | 40 |

[a]Conditions A: **11a** (0.20 mmol), **12a** (0.26 mmol), $Pd(OAc)_2$ (x mol%), **L14** (30 mol%), $Rb_2CO_3$ (3.0 equiv), HOPiv (30 mol%), *p*-xylene (2.0 mL), $N_2$, 120 °C, 12-36 h. [b]Yields were determined by $^1$H NMR analysis of an unpurified product mixture using $CH_2Br_2$ as an internal standard.

**Table S5.** Solvent Investigation Continued.[a,b,c]

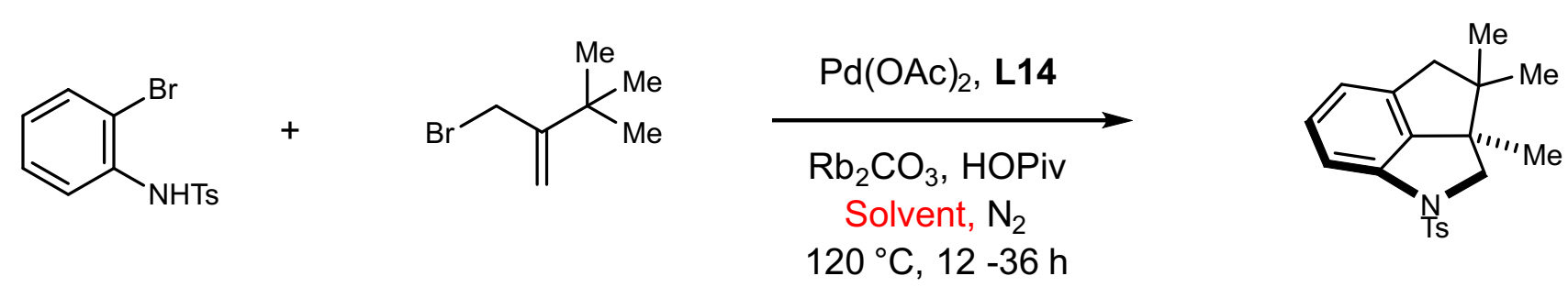


| Entry | Solvent | **13a** (%) |
|---|---|---|
| 1. | DMSO | 15 |
| 2. | chlorobenzene | 40 |
| 3. | *m*-xylene | 46 |
| 4. | *p*-xylene | 86 |
| 5. | trifluorotoluene | 13 |
| 6.[c] | DCE | 0 |
| 7.[c] | HFIP | 0 |

[a]Conditions A: **11a** (0.20 mmol), **12a** (0.26 mmol), $Pd(OAc)_2$ (5 mol%), **L14** (30 mol%), $Rb_2CO_3$ (3.0 equiv), HOPiv (30 mol%), Solvent (2.0 mL), $N_2$, 120 °C, 12-36 h. [b]Yields were determined by $^1$H NMR analysis of an unpurified product mixture using $CH_2Br_2$ as an internal standard. [c]Reaction temperature lowered to 100 °C.

## List of Unsuccessful or Limited Reactivity Substrates.[a,b]

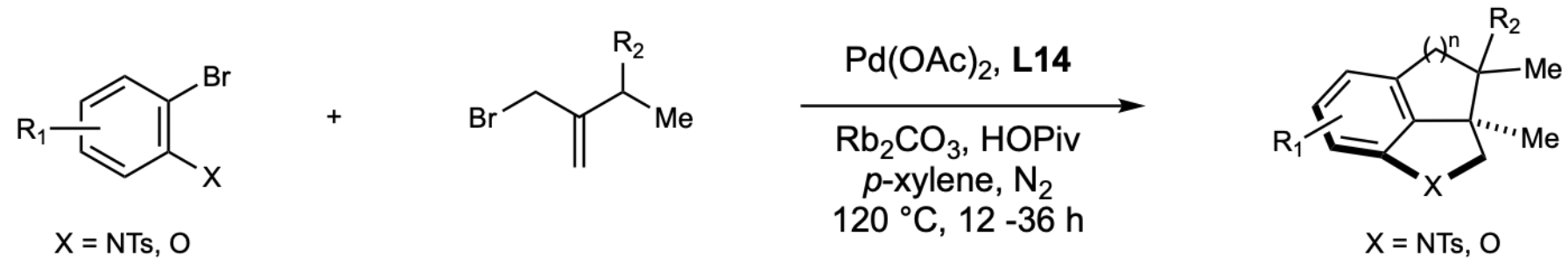


11 12 13

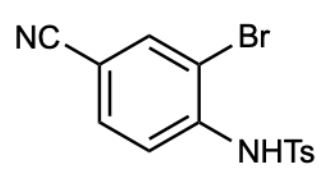


**S31**
n.d.

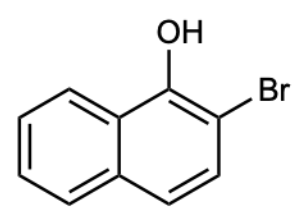


**S32**
12%

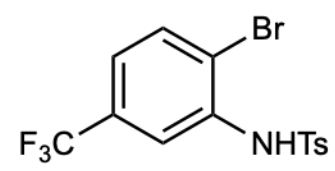


**S33**
n.d.

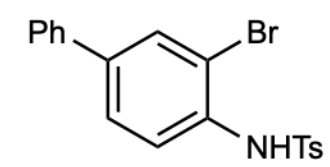


**S34**
n.d.

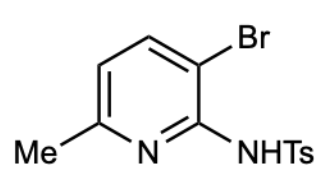


**S35**
20%

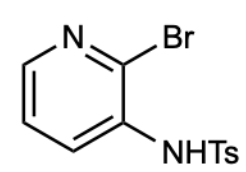


**S36**
n.d.

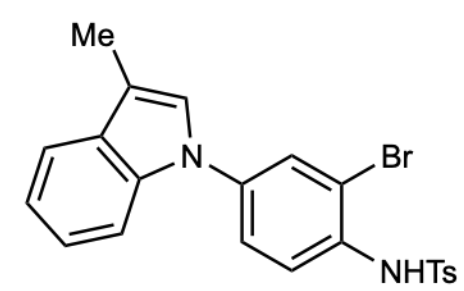


**S37**
n.d.

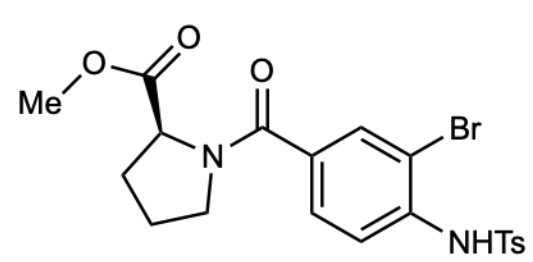


**S38**
17%

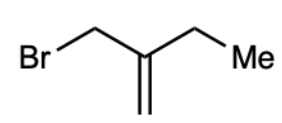


**S39**
n.d.

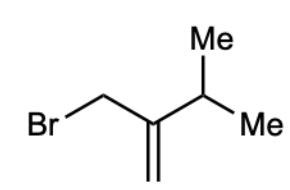


**S40**
n.d.

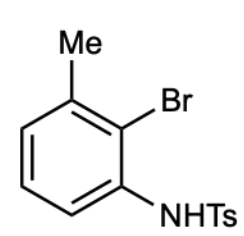


**S41**
n.d.

[a]Conditions A: **11a** (0.20 mmol), **12a** (0.26 mmol), $Pd(OAc)_2$ (5 mol%), **L14** (30 mol%), $Rb_2CO_3$ (3.0 equiv), HOPiv (30 mol%), *p*-xylene (2.0 mL), $N_2$, 120 °C, 12-36 h. [b]Yields were determined by $^1$H NMR analysis of an unpurified product mixture using $CH_2Br_2$ as an internal standard.

## Preliminary Mechanistic Studies

*Part 1: Intramolecular deuterium-labeling experiment with **25–D**:*

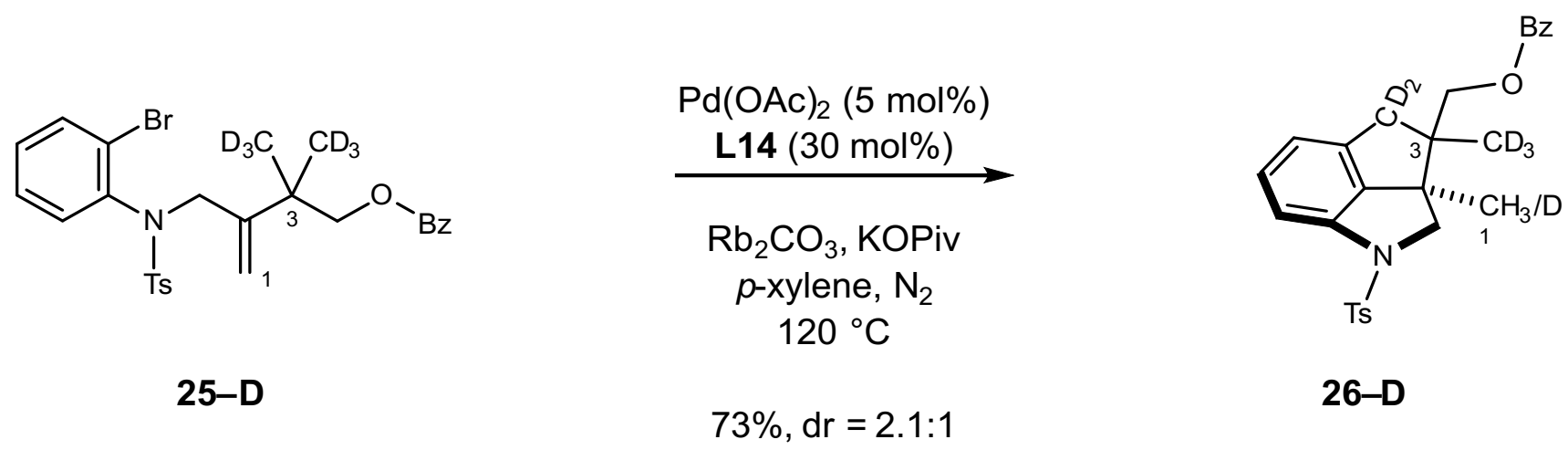


A screw-capped 16 × 125 mm culture tube was sequentially charged under nitrogen atmosphere with the **25–D** (110 mg, 0.20 mmol, 1.0 equiv), $Pd(OAc)_2$ (2.25 mg, 10 µmol, 0.05 equiv), L14 ligand (21.1 mg, 60.0 µmol, 0.30 equiv), rubidium carbonate (139 mg, 0.60 mmol, 3.0 equiv), and anhydrous potassium pivalate (8.41 mg, 60.0 µmol, 0.30 equiv). The reaction vessel was then sealed using a screw cap with a teflon coated septa. The reaction vessel was then placed under nitrogen atmosphere by using a vacuum manifold with a process of evacuating the headspace for 1.0 minute and venting with nitrogen for five times. Next, *para*-xylene (2.0 mL) was added via a syringe to the reaction mixture at r.t. The reaction vessel was then placed into an oil bath that had been preheated to 120 °C. The reaction mixture was allowed to stir for 12 hours at 120 °C. After being allowed to cool to room temperature, the product mixture was diluted with DCM and filtered through a pad of Celite. The filtrate was concentrated and the residue obtained was purified by flash-column chromatography (eluting with 5% ethyl acetate–hexanes) to provide the product **26–D** (68.1 mg, 73% yield) as a colorless oil with a 2.1:1 diastereomeric mixture at the C3 position.

Note: The *para*-xylene used in this experiment was rigorously dried with activated 3Å molecular sieves for 24 hours. Substrate **25–D** was azeotropically dried from toluene (3 × 2 mL) using a vacuum manifold.

*Deuterium labeling analysis of the newly formed C1 methyl group:*
In the obtained $^1$H NMR spectrum of the 2.1:1 mixture of diastereomers, we observed that the deuterium labeling at the C1 position was nearly identical for both diastereomers (see below). According to our proposed mechanistic pathway, only one equivalent of deuterium can migrate from the C3 position to the newly formed C1 methyl group. Therefore:

1) The C1 methyl group of the major diastereomer integrated to 2.60, indicating 40% deuteration.
2) The C1 methyl group of the minor diastereomer integrated to 2.61, indicating 39% deuteration.

*Spectroscopic data for **26–D**:*

**26–D**: Major diastereomer: $^{1}H$ NMR (400 MHz, $CDCl_3$) δ 8.02 – 7.97 (m, 2H), 7.76 – 7.71 (m, 2H), 7.65 – 7.60 (m, 1H), 7.53 – 7.48 (m, 2H), 7.26 – 7.22 (m, 3H), 7.10 (t, *J* = 7.6 Hz, 1H), 6.82 (d, *J* = 7.3 Hz, 1H), 4.43 (d, *J* = 11.1 Hz, 1H), 4.33 (d, *J* = 11.1 Hz, 1H), 3.98 (d, *J* = 10.4 Hz, 1H), 3.79 (d, *J* = 10.3 Hz, 1H), 2.37 (s, 3H), 1.10 (s, 2.60H).

**26–D**: Minor diastereomer: $^{1}H$ NMR (600 MHz, $CDCl_3$) δ 7.60 – 7.57 (m, 1H), 7.44 – 7.42 (m, 2H), 7.26 – 7.24 (m, 3H), 7.09 – 7.06 (m, 1H), 6.93 (d, *J* = 8.1 Hz, 2H), 6.76 (d, *J* = 7.4 Hz, 1H), 4.41 (d, *J* = 1.6 Hz, 1H), 4.03 (d, *J* = 11.1 Hz, 1H), 3.72 (d, *J* = 10.0 Hz, 1H), 3.59 (d, *J* = 11.3 Hz, 1H), 2.25 (s, 3H), 1.26 (s, 2.61H).

$^{1}H$ NMR (400 MHz, $CDCl_3$) for the mixture of diastereomers with the major diastereomer integrated for deuterium labeling on top and the minor one on the bottom (see below).

Major diastereomer:

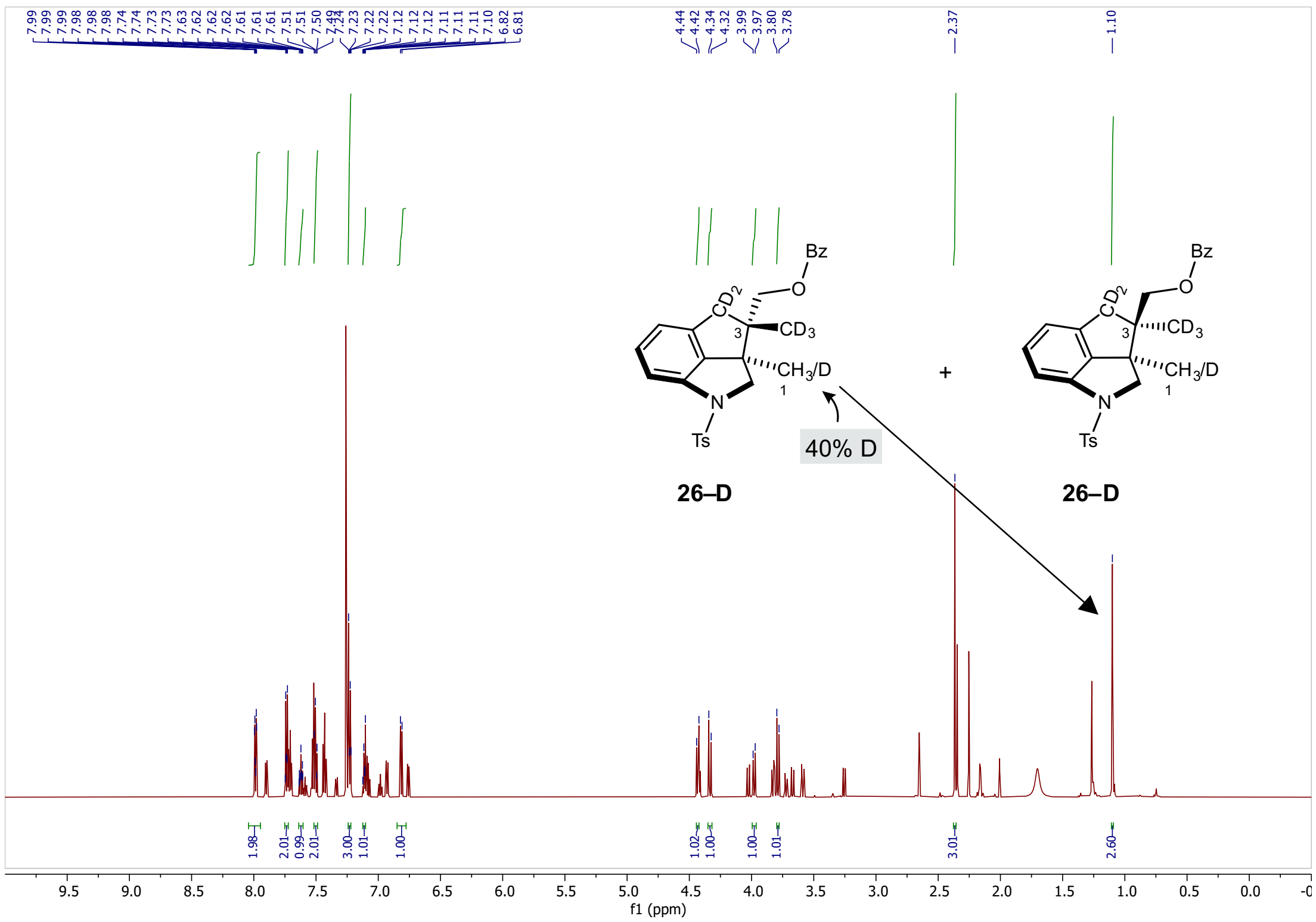


Minor diastereomer:

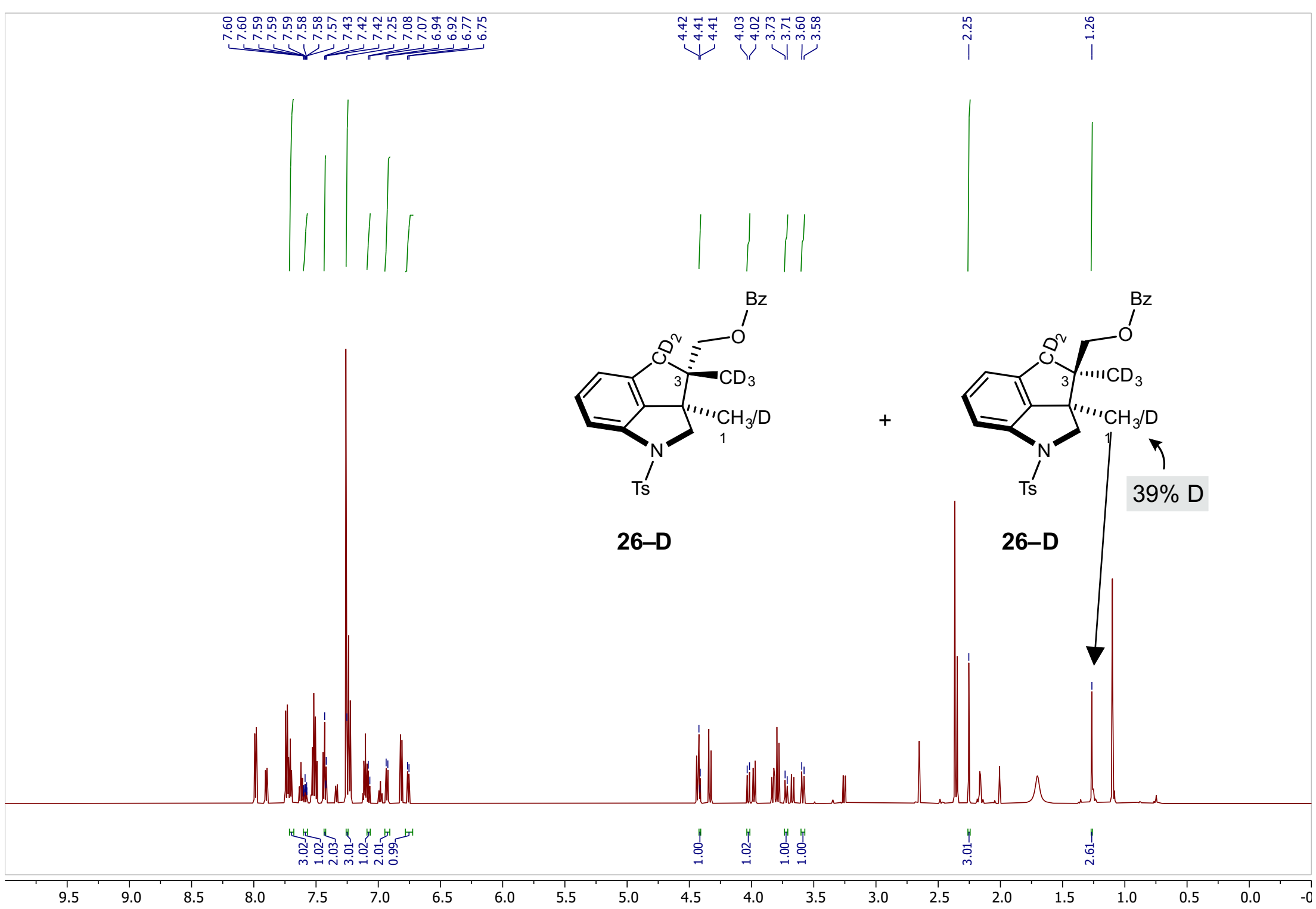

Using preparative thin-layer chromatography (1% methanol in dichloromethane as the eluent), we were able to purify the major diastereomer, and the corresponding $^{1}H$ NMR spectrum is included below.

$^{1}H$ NMR (400 MHz, $CDCl_3$) for the major diastereomer:

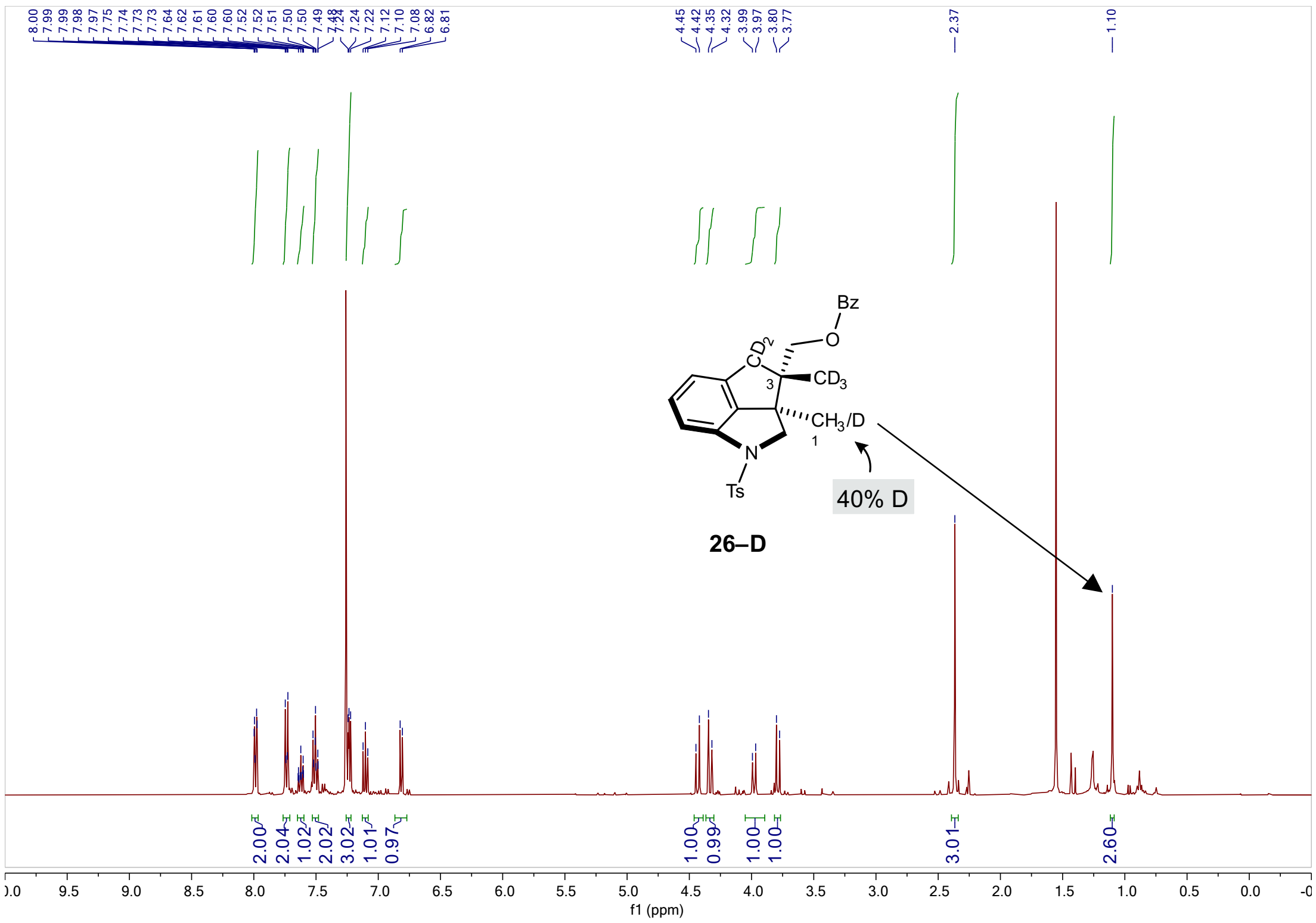

*Part 2: Intramolecular deuterium-labeling experiment with* ***27–D****:*

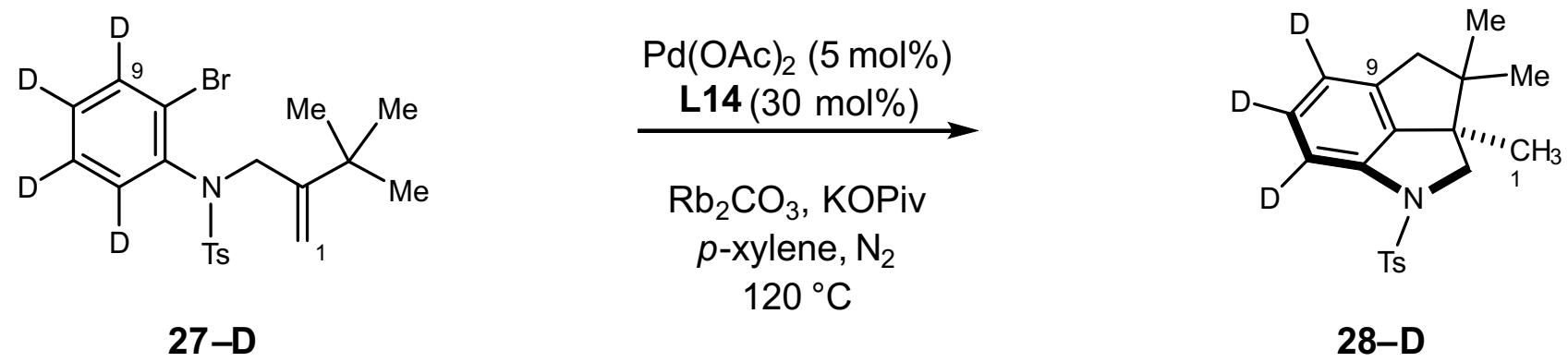


A screw-capped 16 × 125 mm culture tube was sequentially charged under nitrogen atmosphere with the **27–D** (85.3 mg, 0.20 mmol, 1.0 equiv), $Pd(OAc)_2$ (2.25 mg, 10 µmol, 0.05 equiv), L14 ligand (21.1 mg, 60.0 µmol, 0.30 equiv), rubidium carbonate (139 mg, 0.60 mmol, 3.0 equiv), and anhydrous potassium pivalate (8.41 mg, 60.0 µmol, 0.30 equiv). The reaction vessel was then sealed using a screw cap with a teflon coated septa. The reaction vessel was then placed under nitrogen atmosphere by using a vacuum manifold with a process of evacuating the headspace for 1.0 minute and venting with nitrogen for five times. Next, *para*-xylene (2.0 mL) was added via a syringe to the reaction mixture at r.t. The reaction vessel was then placed into an oil bath that had been preheated to 120 °C. The reaction mixture was allowed to stir for 12 hours at 120 °C. After being allowed to cool to room temperature, the product mixture was diluted with DCM and filtered through a pad of Celite. The filtrate was concentrated and the residue obtained was purified by flash-column chromatography (eluting with 5% ethyl acetate–hexanes) to provide the product **28–D** (44.8 mg, 65% yield) as amorphous solids.

Note: The *para*-xylene used in this experiment was rigorously dried with activated 3Å molecular sieves for 24 hours. Substrate **27–D** was azeotropically dried from toluene (3 × 2 mL) using a vacuum manifold.

*Deuterium labeling analysis of the newly formed C1 methyl group:*
In the obtained $^1$H NMR spectrum for the product **28–D**, we did not observe any deuterium labeling at the newly established methyl C1 position (see spectrum below).

$^1$H NMR (400 MHz, $CDCl_3$) δ 7.71 (d, $J$ = 8.4 Hz, 2H), 7.25 – 7.22 (m, 2H), 7.19 (m, 0.04H), 7.06 – 7.03 (m, 0.73H), 6.77 – 6.72 (m, 0.73H), 3.78 (d, $J$ = 10.1 Hz, 1H), 3.64 (d, $J$ = 10.1 Hz, 1H), 3.25 (d, $J$ = 15.2 Hz, 1H), 2.38 (d, $J$ = 15.9 Hz, 4H), 1.13 (s, 3H), 1.05 (s, 3H), 0.57 (s, 3H).

$^{1}H$ NMR (400 MHz, $CDCl_3$) for the indoline heterocycle **28–D**:

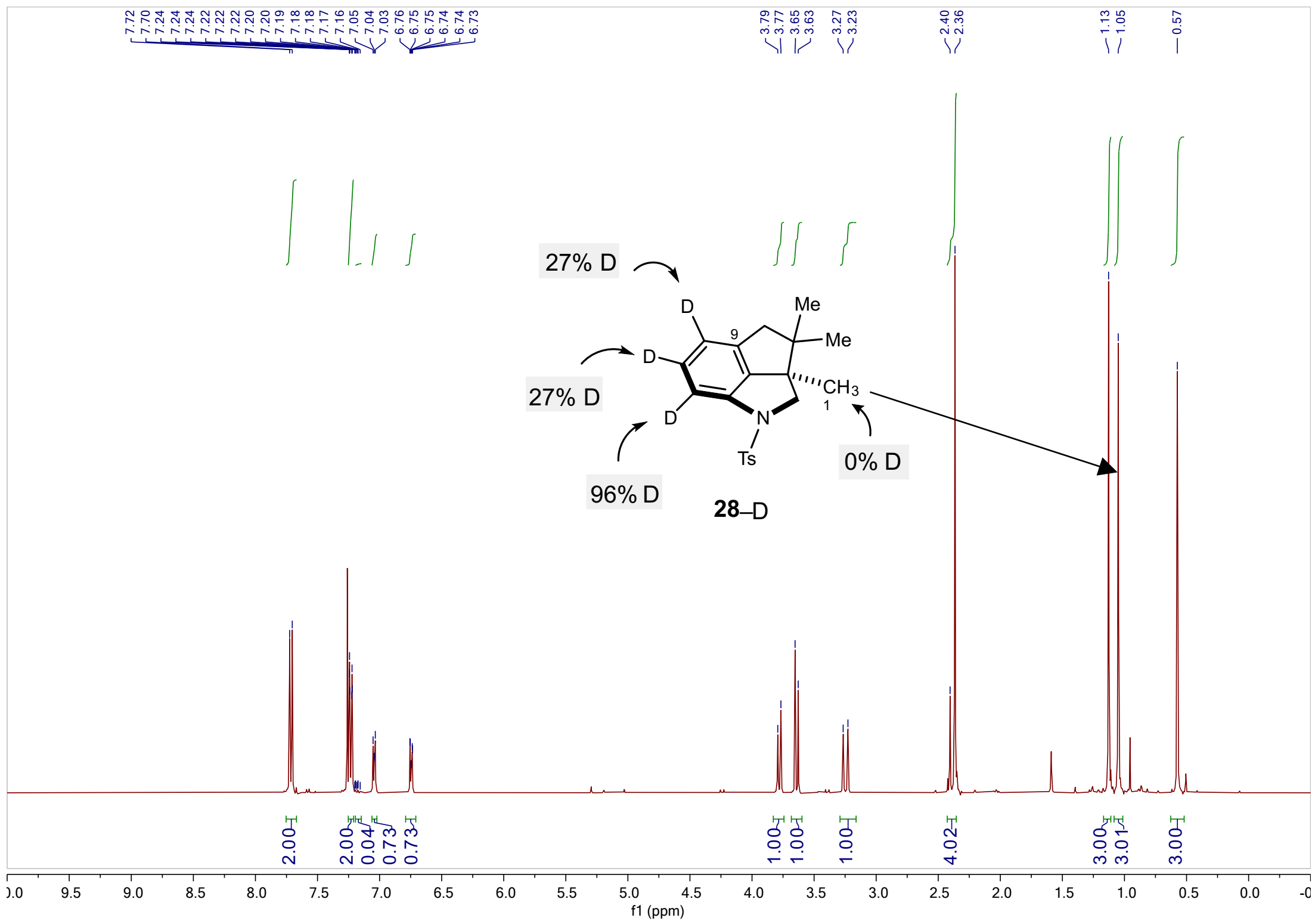

*Part 3: Intermolecular deuterium-labeling experiment with $D_2O$:*

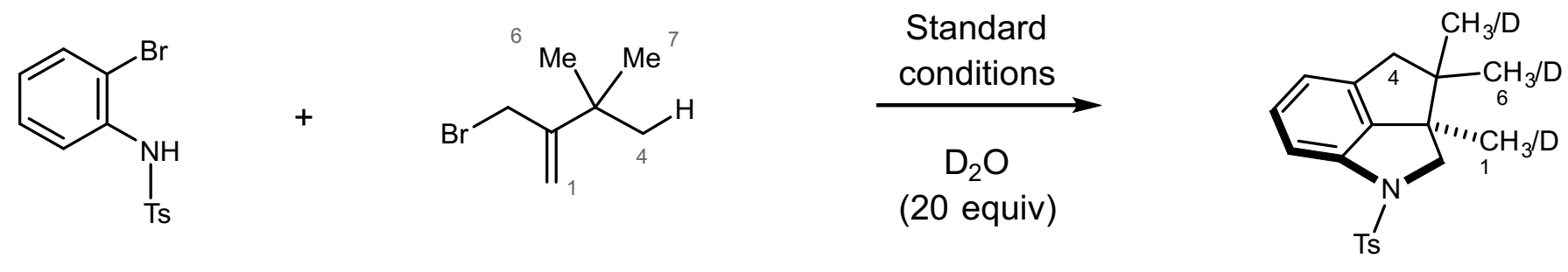


A screw-capped 16 × 125 mm culture tube was sequentially charged under nitrogen atmosphere with the **11a** (65.2 mg, 0.20 mmol, 1.0 equiv), $Pd(OAc)_2$ (2.25 mg, 10 μmol, 0.05 equiv), L14 ligand (21.1 mg, 60.0 μmol, 0.30 equiv), rubidium carbonate (139 mg, 0.60 mmol, 3.0 equiv), and anhydrous potassium pivalate (8.41 mg, 60.0 μmol, 0.30 equiv). The reaction vessel was then sealed using a screw cap with a teflon coated septa. The reaction vessel was then placed under nitrogen atmosphere by using a vacuum manifold with a process of evacuating the headspace for 1.0 minute and venting with nitrogen for five times. Next, the bromide **12a** (46.0 mg, 0.26 mmol, 1.3 equiv) was dissolved in *para*-xylene (2.0 mL) and it was added via a syringe to the reaction mixture at r.t. Then, deuterium oxide (4.00 mmol, 72.4 µL, 20.0 equiv) was added via a syringe to the reaction mixture at r.t. The reaction vessel was then placed into an oil bath that had been preheated to 120 °C. The reaction mixture was allowed to stir for 12 hours at 120 °C. After being allowed to cool to room temperature, the product mixture was diluted with DCM and filtered through a pad of Celite. The filtrate was concentrated and the residue obtained was purified by flash-column chromatography (eluting with 5% ethyl acetate–hexanes) to provide the product **29–D** (47.8 mg, 70% yield) as amorphous solid.

$^1$H NMR (400 MHz, $CDCl_3$) δ 7.71 (d, 2H), 7.23 (d, $J$ = 7.8 Hz, 2H), 7.19 (d, 1H), 7.04 (t, $J$ = 7.6 Hz, 1H), 6.75 (dd, $J$ = 7.2, 1.1 Hz, 1H), 3.78 (d, $J$ = 10.2 Hz, 1H), 3.64 (d, $J$ = 10.2 Hz, 1H), 3.25 (d, $J$ = 15.3 Hz, 0.97H), 2.41 – 2.36 (m, 3.97H), 1.13 (s, 2.43H), 1.05 (s, 1.32H), 0.58 (s, 2.43H).

*Deuterium labeling analysis of the product **29–D**:*

In the obtained $^1$H NMR spectrum (see below) for the product **29–D**, we detected the following deuteration at the indicated positions:

1) The C1 methyl group integrated to 1.32, indicating 56% deuteration.
2) The C6 methyl group integrated to 2.43, indicating 19% deuteration.
3) The C7 methyl group integrated to 2.43, indicating 19% deuteration.
4) Each proton at the C4 methylene position integrated to 0.97, indicating 3% deuteration.

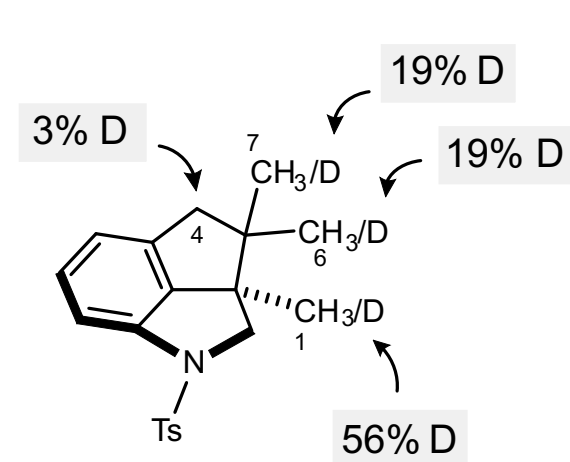


**29–D**

$^{1}$H NMR (400 MHz, $CDCl_3$) for the deuterium-labeled product **29–D**:

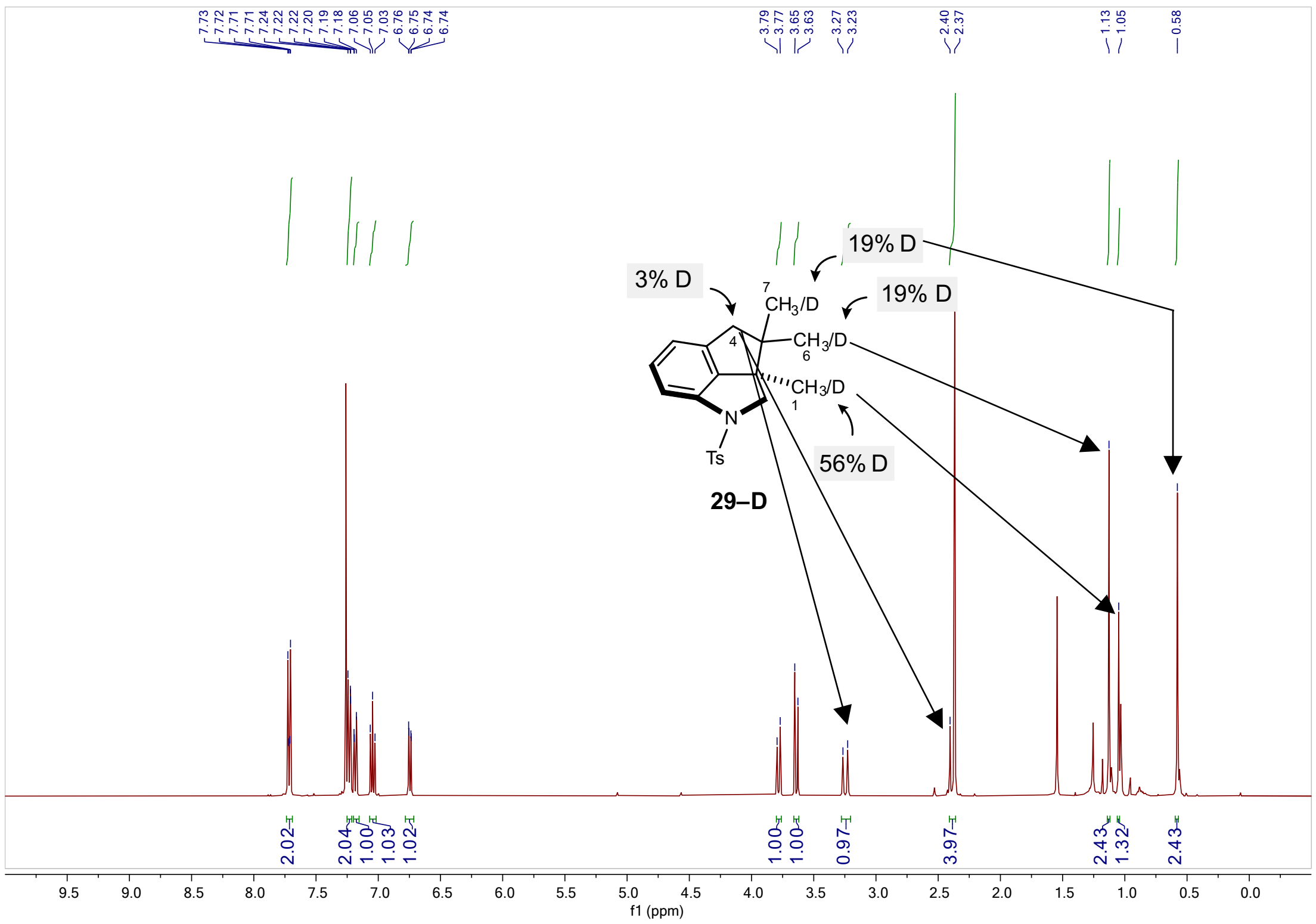

*Part 4: Deuterium-labeling experiment with $D_2O$ using the Product from the Pd Reaction:*

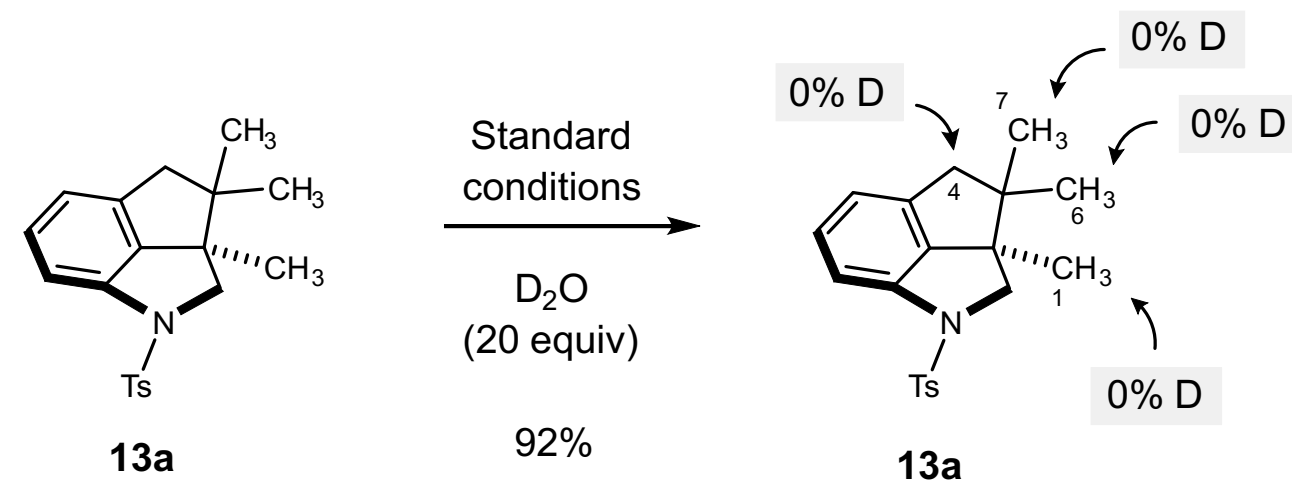


A screw-capped 16 × 125 mm culture tube was sequentially charged under nitrogen atmosphere with the **13a** (68.3 mg, 0.20 mmol, 1.0 equiv), $Pd(OAc)_2$ (2.25 mg, 10 µmol, 0.05 equiv), L14 ligand (21.1 mg, 60.0 µmol, 0.30 equiv), rubidium carbonate (139 mg, 0.60 mmol, 3.0 equiv), and anhydrous potassium pivalate (8.41 mg, 60.0 µmol, 0.30 equiv). The reaction vessel was then sealed using a screw cap with a teflon coated septa. The reaction vessel was then placed under nitrogen atmosphere by using a vacuum manifold with a process of evacuating the headspace for 1.0 minute and venting with nitrogen for five times. Next *para*-xylene (2.0 mL) was added via a syringe to the reaction mixture at r.t. Then, deuterium oxide (4.00 mmol, 72.4 µL, 20.0 equiv) was added via a syringe to the reaction mixture at r.t. The reaction vessel was then placed into an oil bath that had been preheated to 120 °C. The reaction mixture was allowed to stir for 12 hours at 120 °C. After being allowed to cool to room temperature, the product mixture was diluted with DCM and filtered through a pad of Celite. The filtrate was concentrated and the residue obtained was purified by flash-column chromatography (eluting with 5% ethyl acetate–hexanes) to provide the product **13a** (62.8 mg, 92% yield) as amorphous solids.

$^1$H NMR (400 MHz, $CDCl_3$) δ 7.72 (d, $J$ = 8.3 Hz, 2H), 7.23 (d, $J$ = 8.0 Hz, 2H), 7.18 (d, $J$ = 7.8 Hz, 1H), 7.04 (t, $J$ = 7.5 Hz, 1H), 6.75 (d, $J$ = 7.2 Hz, 1H), 3.78 (d, $J$ = 10.2 Hz, 1H), 3.64 (d, $J$ = 10.2 Hz, 1H), 3.25 (d, $J$ = 15.2 Hz, 1H), 2.41 – 2.36 (m, 4H), 1.13 (s, 3H), 1.05 (s, 3H), 0.58 (s, 3H).

*Deuterium labeling analysis of the product **13a**:*

In the obtained $^1$H NMR spectrum for **13a**, we did not observe any deuterium labeling at any of the positions (see spectrum below).

$^1$H NMR (400 MHz, $CDCl_3$) for **13a**:

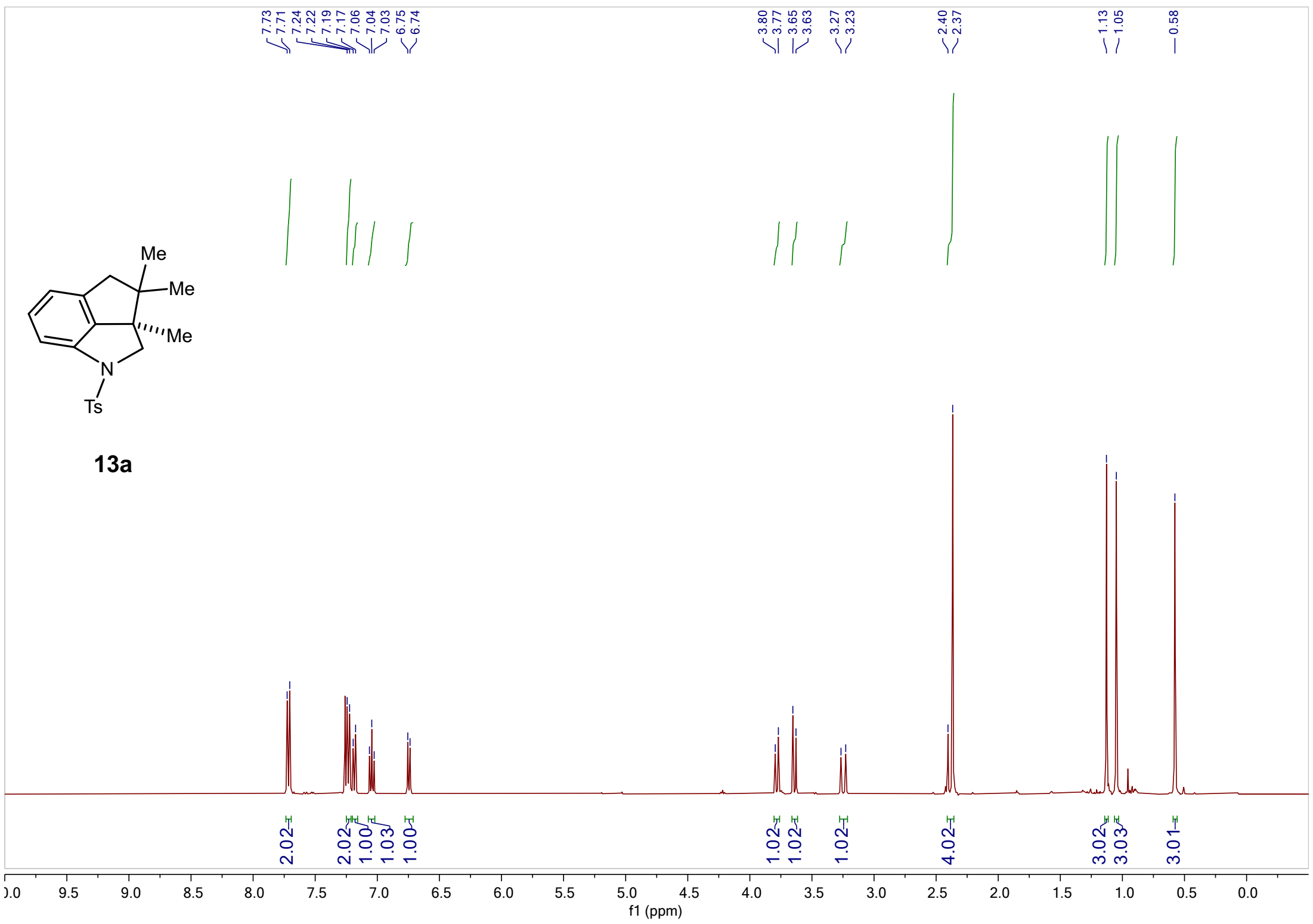

*Part 5: Evaluation of phosphine oxide as the ligand:*

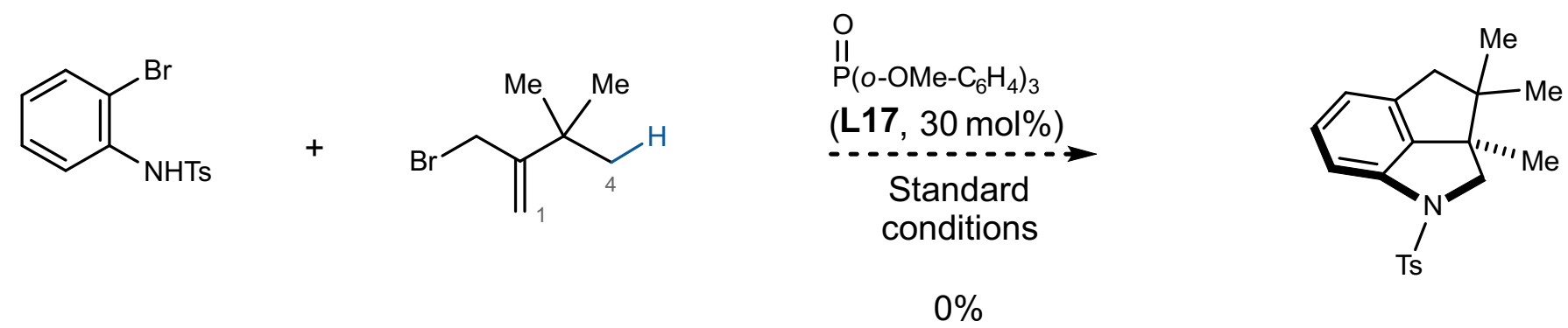


A screw-capped 16 × 125 mm culture tube was sequentially charged under nitrogen atmosphere with the **11a** (65.2 mg, 0.20 mmol, 1.0 equiv), $Pd(OAc)_2$ (2.25 mg, 10 µmol, 0.05 equiv), phosphine monoxide **L17** ligand[14] (22.1 mg, 60.0 µmol, 0.30 equiv), rubidium carbonate (139 mg, 0.60 mmol, 3.0 equiv), and anhydrous potassium pivalate (8.41 mg, 60.0 µmol, 0.30 equiv). The reaction vessel was then sealed using a screw cap with a teflon coated septa. The reaction vessel was then placed under nitrogen atmosphere by using a vacuum manifold with a process of evacuating the headspace for 1.0 minute and venting with nitrogen for five times. Next, the bromide **12a** (46.0 mg, 0.26 mmol, 1.3 equiv) was dissolved in *para*-xylene (2.0 mL) and it was added via a syringe to the reaction mixture at r.t. The reaction vessel was then placed into an oil bath that had been preheated to 120 °C. The reaction mixture was allowed to stir for 12 hours at 120 °C. After being allowed to cool to room temperature, the product mixture was diluted with DCM and filtered through a pad of Celite. The filtrate was concentrated and the residue obtained was subjected to $^1H$ NMR analysis. The analysis of the unpurified spectrum showed that no desired product **13a** was formed with ligand **L17**.

**Characterization of Products.**

Products:

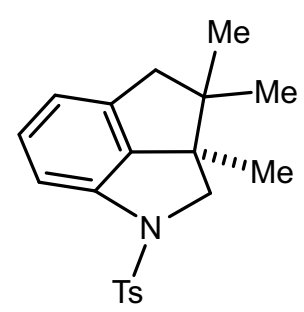


**13a**

2a,3,3-trimethyl-1-tosyl-2,2a,3,4-tetrahydro-1H-cyclopenta[cd]indole (**13a**). Prepared according to general procedure E: $^{1}$H NMR (400 MHz, $CDCl_3$) δ 7.72 (d, *J* = 8.3 Hz, 2H), 7.23 (d, *J* = 8.0 Hz, 2H), 7.18 (d, *J* = 7.8 Hz, 1H), 7.04 (t, *J* = 7.5 Hz, 1H), 6.75 (d, *J* = 7.2 Hz, 1H), 3.78 (d, *J* = 10.2 Hz, 1H), 3.64 (d, *J* = 10.2 Hz, 1H), 3.25 (d, *J* = 15.2 Hz, 1H), 2.41 – 2.36 (m, 4H), 1.13 (s, 3H), 1.05 (s, 3H), 0.58 (s, 3H). $^{13}$C NMR (101 MHz, $CDCl_3$) δ 149.8, 144.0, 139.0, 138.3, 134.4, 129.7, 129.4, 127.4, 120.2, 112.3, 62.7, 51.3, 51.0, 50.5, 25.7, 23.8, 21.7, 21.1. HRMS (ESI-TOF) *m/z*: $[M + H]^+$ calcd for $C_{20}H_{24}NO_2S$, 342.1523; found, 342.1530.

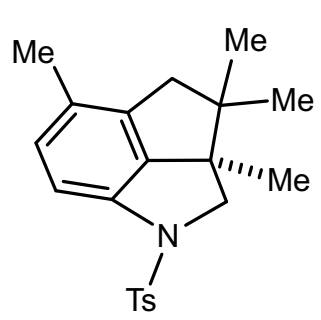


**13b**

2a,3,3,5-tetramethyl-1-tosyl-2,2a,3,4-tetrahydro-1H-cyclopenta[cd]indole (**13b**). Prepared according to general procedure E: $^{1}$H NMR (400 MHz, $CDCl_3$) δ 7.71 (d, *J* = 8.3 Hz, 2H), 7.23 (d, *J* = 8.1 Hz, 2H), 7.08 (d, *J* = 7.7 Hz, 1H), 6.83 (d, *J* = 7.9 Hz, 1H), 3.79 (d, *J* = 10.2 Hz, 1H), 3.62 (d, *J* = 10.2 Hz, 1H), 3.13 (d, *J* = 15.3 Hz, 1H), 2.38 (d, *J* = 15.3 Hz, 1H), 2.37 (s, 3H), 2.13 (s, 3H), 1.12 (s, 3H), 1.00 (s, 3H), 0.59 (s, 3H). $^{13}$C NMR (101 MHz, $CDCl_3$) δ 149.9, 143.8, 137.4, 136.0, 134.5, 130.1, 130.0, 129.7, 127.4, 112.8, 62.9, 51.2, 51.0, 49.2, 25.8, 23.8, 21.7, 21.2, 18.1. HRMS (ESI-TOF) *m/z*: $[M + H]^+$ calcd for $C_{21}H_{26}NO_2S$, 356.1679; found, 356.1686.

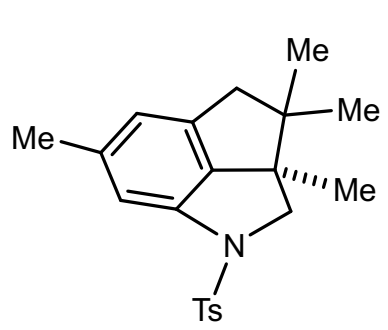


**13c**

2a,3,3,6-tetramethyl-1-tosyl-2,2a,3,4-tetrahydro-1H-cyclopenta[cd]indole (**13c**). Prepared according to general procedure E: $^{1}$H NMR (400 MHz, $CDCl_3$) δ 7.72 (d, *J* = 8.3 Hz, 2H), 7.23 (d,

$J$ = 8.3 Hz, 2H), 7.02 (s, 1H), 6.58 (s, 1H), 3.81 (d, $J$ = 10.3 Hz, 1H), 3.61 (d, $J$ = 10.2 Hz, 1H), 3.21 (d, $J$ = 15.2 Hz, 1H), 2.37 (s, 3H), 2.34 (s, 3H), 2.30 (d, $J$ = 10.4 Hz, 1H), 1.11 (s, 3H), 1.00 (s, 3H), 0.58 (s, 3H). $^{13}$C NMR (101 MHz, $CDCl_3$) δ 147.2, 143.8, 139.7, 138.8, 138.4, 134.5, 129.6, 127.2, 120.8, 113.0, 62.9, 51.4, 50.6, 50.3, 25.6, 23.7, 22.3, 21.5, 21.2. HRMS (ESI-TOF) *m/z*: $[M + H]^+$ calcd for $C_{21}H_{26}NO_2S$, 356.1679; found, 356.1689.

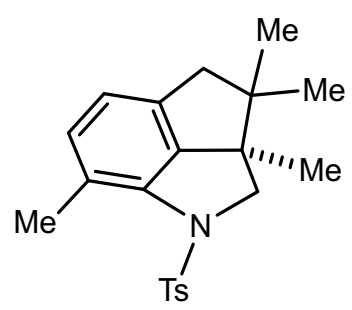


**13d**

2a,3,3,7-tetramethyl-1-tosyl-2,2a,3,4-tetrahydro-1H-cyclopenta[cd]indole (**13d**). Prepared according to general procedure E: $^1$H NMR (400 MHz, $CDCl_3$) δ 7.74 (d, $J$ = 8.4 Hz, 2H), 7.28 (d, $J$ = 8.2 Hz, 2H), 6.86 (d, $J$ = 7.4 Hz, 1H), 6.75 (d, $J$ = 7.4 Hz, 1H), 4.11 (d, $J$ = 11.8 Hz, 1H), 3.80 (d, $J$ = 11.6 Hz, 1H), 3.21 (d, $J$ = 15.0 Hz, 1H), 2.43 (s, 3H), 2.40 (s, 3H), 2.36 (d, $J$ = 5.1 Hz, 1H), 1.09 (s, 3H), 0.77 (s, 3H), 0.73 (s, 3H). $^{13}$C NMR (101 MHz, $CDCl_3$) δ 153.3, 143.6, 137.6, 137.4, 136.8, 132.3, 129.7, 127.5, 127.4, 122.5, 63.9, 51.6, 50.8, 50.3, 29.9, 25.7, 23.9, 21.7, 20.3. HRMS (ESI-TOF) *m/z*: $[M + H]^+$ calcd for $C_{21}H_{26}NO_2S$, 356.1679; found, 356.1689.

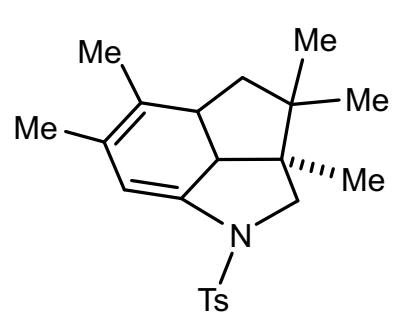


**13e**

2a,3,3,5,6-pentamethyl-1-tosyl-2,2a,2a1,3,4,4a-hexahydro-1H-cyclopenta[cd]indole (**13e**). Prepared according to general procedure E: $^1$H NMR (400 MHz, $CDCl_3$) δ 7.72 (d, $J$ = 8.4 Hz, 2H), 7.24 (d, $J$ = 7.9 Hz, 2H), 7.03 (s, 1H), 3.81 (d, $J$ = 10.3 Hz, 1H), 3.59 (d, $J$ = 10.3 Hz, 1H), 3.14 (d, $J$ = 15.2 Hz, 1H), 2.41 (d, $J$ = 15.2 Hz, 1H), 2.37 (s, 3H), 2.24 (s, 3H), 2.03 (s, 3H), 1.11 (s, 3H), 0.95 (s, 3H), 0.61 (s, 3H). $^{13}$C NMR (101 MHz, $CDCl_3$) δ 147.4, 143.8, 137.7, 137.7, 135.9, 134.7, 129.7, 128.6, 127.4, 114.2, 62.9, 51.1, 49.4, 25.9, 23.9, 21.7, 21.4, 20.7, 15.7. HRMS (ESI-TOF) *m/z*: $[M + H]^+$ calcd for $C_{22}H_{28}NO_2S$, 370.1836; found, 370.1844.

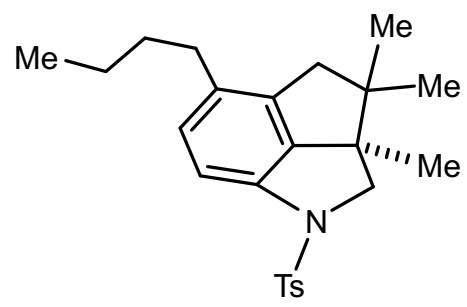

**13f**

5-butyl-2a,3,3-trimethyl-1-tosyl-2,2a,3,4-tetrahydro-1H-cyclopenta[cd]indole (**13f**). Prepared according to general procedure E: $^1$H NMR (500 MHz, $CDCl_3$) δ 7.72 (d, *J* = 8.1 Hz, 2H), 7.23 (d, *J* = 8.0 Hz, 2H), 7.09 (d, *J* = 7.9 Hz, 1H), 6.83 (d, *J* = 7.9 Hz, 1H), 3.79 (d, *J* = 10.3 Hz, 1H), 3.61 (d, *J* = 10.2 Hz, 1H), 3.15 (d, *J* = 13.7 Hz, 1H), 2.44 – 2.39 (m, 3H), 2.37 (s, 3H), 1.50 – 1.44 (m, 2H), 1.32 – 1.27 (m, 2H), 1.12 (s, 3H), 1.01 (s, 3H), 0.90 (t, *J* = 7.3 Hz, 3H), 0.59 (s, 3H). $^{13}$C NMR (126 MHz, $CDCl_3$) δ 149.8, 143.7, 137.0, 136.0, 135.3, 134.5, 129.5, 129.1, 127.3, 112.6, 62.7, 51.0, 50.9, 49.1, 33.3, 32.6, 25.6, 23.7, 22.4, 21.5, 21.1, 14.0. HRMS (ESI-TOF) *m/z*: $[M + H]^+$ calcd for $C_{24}H_{32}NO_2S$, 398.2149; found, 398.2152.

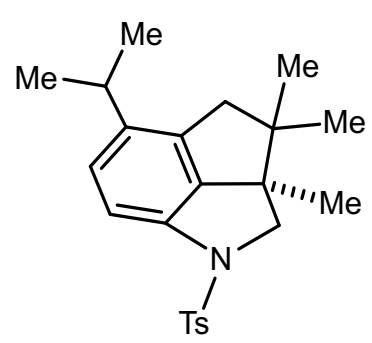

**13g**

5-isopropyl-2a,3,3-trimethyl-1-tosyl-2,2a,3,4-tetrahydro-1H-cyclopenta[cd]indole (**13g**). Prepared according to general procedure E: $^1$H NMR (400 MHz, $CDCl_3$) δ 7.72 (d, *J* = 8.4 Hz, 2H), 7.24 (d, *J* = 7.8 Hz, 2H), 7.11 (d, *J* = 7.9 Hz, 1H), 6.88 (d, *J* = 8.1 Hz, 1H), 3.81 (d, *J* = 10.3 Hz, 1H), 3.61 (d, *J* = 10.3 Hz, 1H), 3.18 (d, *J* = 15.2 Hz, 1H), 2.79 (p, *J* = 6.9 Hz, 1H), 2.49 (d, *J* = 15.2 Hz, 1H), 2.37 (s, 3H), 1.16 (dd, *J* = 7.0, 4.3 Hz, 6H), 1.13 (s, 3H), 0.99 (s, 3H), 0.61 (s, 3H). $^{13}$C NMR (101 MHz, $CDCl_3$) δ 150.0, 143.8, 141.4, 136.1, 136.0, 134.8, 129.7, 127.4, 126.2, 112.8, 62.7, 50.9, 50.8, 49.6, 31.7, 25.7, 23.8, 23.0, 21.7, 21.2, 14.3. HRMS (ESI-TOF) *m/z*: $[M + H]^+$ calcd for $C_{23}H_{30}NO_2S$, 384.1992; found, 384.1993.

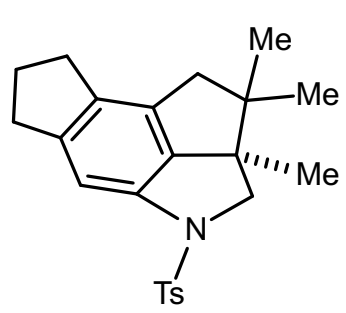

**13h**

2,2,2a-trimethyl-4-tosyl-1,2,2a,3,4,6,7,8-octahydrodicyclopenta[cd,f]indole (**13h**). Prepared according to general procedure E: $^1$H NMR (400 MHz, $CDCl_3$) δ 7.73 (d, *J* = 8.3 Hz, 2H), 7.24 (d, *J* = 8.2 Hz, 2H), 7.06 (s, 1H), 3.86 (d, *J* = 10.4 Hz, 1H), 3.61 (d, *J* = 10.3 Hz, 1H), 3.13 (d, *J* = 15.3 Hz, 1H), 2.88 – 2.83 (m, 2H), 2.72 – 2.58 (m, 2H), 2.37 (s, 3H), 2.34 (d, *J* = 13.8 Hz, 1H),

2.11 – 2.02 (m, 2H), 1.11 (s, 3H), 0.94 (s, 3H), 0.64 (s, 3H). $^{13}$C NMR (101 MHz, $CDCl_3$) δ 148.7, 146.4, 143.7, 136.7, 136.1, 135.0, 134.1, 129.7, 127.4, 109.3, 63.0, 51.1, 50.7, 49.3, 33.3, 30.2, 26.0, 25.9, 23.9, 21.7, 21.5. HRMS (ESI-TOF) *m/z*: $[M + H]^+$ calcd for $C_{23}H_{28}NO_2S$, 382.1836; found, 382.1832.

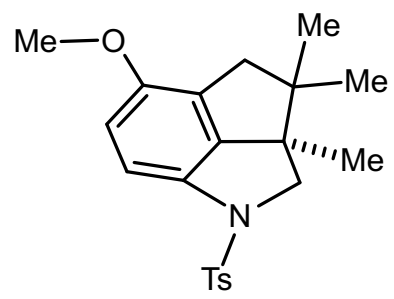


**13i**

5-methoxy-2a,3,3-trimethyl-1-tosyl-2,2a,3,4-tetrahydro-1H-cyclopenta[cd]indole (**13i**). Prepared according to general procedure E: $^1$H NMR (400 MHz, $CDCl_3$) δ 7.71 (d, *J* = 8.3 Hz, 2H), 7.23 (d, *J* = 8.0 Hz, 2H), 7.13 (d, *J* = 8.2 Hz, 1H), 6.54 (d, *J* = 8.3 Hz, 1H), 3.80 (d, *J* = 10.4 Hz, 1H), 3.77 (s, 3H), 3.60 (d, *J* = 10.3 Hz, 1H), 3.14 (d, *J* = 15.4 Hz, 1H), 2.54 (d, *J* = 15.3 Hz, 1H), 2.37 (s, 3H), 1.11 (s, 3H), 0.96 (s, 3H), 0.62 (s, 3H). $^{13}$C NMR (101 MHz, $CDCl_3$) δ 153.7, 152.7, 143.9, 134.4, 131.7, 129.7, 127.5, 124.9, 114.2, 112.3, 62.6, 56.5, 51.3, 51.1, 48.3, 25.8, 23.7, 21.7, 21.2. HRMS (ESI-TOF) *m/z*: $[M + H]^+$ calcd for $C_{21}H_{26}NO_3S$, 372.1628; found, 372.1631.

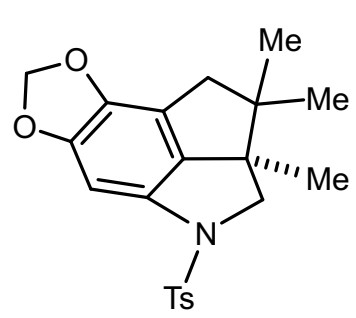


**13j**

2,2,2a-trimethyl-4-tosyl-2,2a,3,4-tetrahydro-1H-cyclopenta[cd][1,3]dioxolo[4,5-f]indole (**13j**). Prepared according to general procedure E: $^1$H NMR (400 MHz, $CDCl_3$) δ 7.65 (d, *J* = 8.3 Hz, 2H), 7.20 (d, *J* = 8.3 Hz, 2H), 6.76 (s, 1H), 5.82 (dd, *J* = 11.5, 1.3 Hz, 2H), 3.79 (d, *J* = 10.7 Hz, 1H), 3.52 (d, *J* = 10.6 Hz, 1H), 3.00 (d, *J* = 15.4 Hz, 1H), 2.40 (d, *J* = 15.4 Hz, 1H), 2.32 (s, 3H), 1.02 (s, 3H), 0.79 (s, 3H), 0.60 (s, 3H). $^{13}$C NMR (101 MHz, $CDCl_3$) δ 148.8, 144.2, 143.8, 140.2, 134.4, 131.2, 129.6, 127.3, 117.7, 101.2, 97.3, 62.8, 51.0, 50.9, 47.3, 25.5, 23.5, 21.6, 21.5. HRMS (ESI-TOF) *m/z*: $[M + H]^+$ calcd for $C_{21}H_{24}NO_4S$, 386.1421; found, 386.1424.

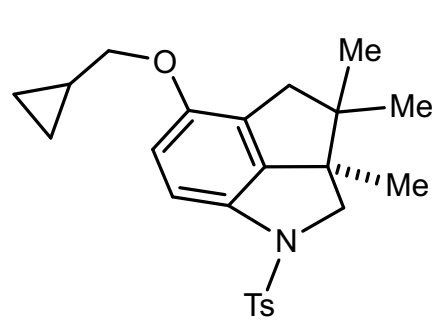


**13k**

5-(cyclopropylmethoxy)-2a,3,3-trimethyl-1-tosyl-2,2a,3,4-tetrahydro-1H-cyclopenta[cd]indole (**13k**). Prepared according to general procedure E: $^1$H NMR (400 MHz, $CDCl_3$) δ 7.70 (d, *J* = 8.4 Hz, 2H), 7.23 (d, *J* = 8.0 Hz, 2H), 7.10 (d, *J* = 8.2 Hz, 1H), 6.54 (d, *J* = 8.3 Hz, 1H), 3.81 – 3.70 (m, 3H), 3.59 (d, *J* = 10.4 Hz, 1H), 3.16 (d, *J* = 15.3 Hz, 1H), 2.56 (d, *J* = 15.3 Hz, 1H), 2.37 (s, 3H), 1.10 (s, 3H), 0.96 (s, 3H), 0.62 (s, 3H), 0.60 – 0.55 (m, 2H), 0.34 – 0.23 (m, 2H). $^{13}$C NMR (101 MHz, $CDCl_3$) δ 153.2, 152.5, 143.9, 134.3, 131.8, 129.6, 127.5, 125.5, 114.5, 114.2, 74.5, 62.5, 51.3, 51.0, 48.7, 25.8, 23.7, 21.7, 21.2, 10.7, 3.2, 3.2. HRMS (ESI-TOF) *m/z*: $[M + H]^+$ calcd for $C_{24}H_{30}NO_3S$, 412.1941; found, 412.1942.

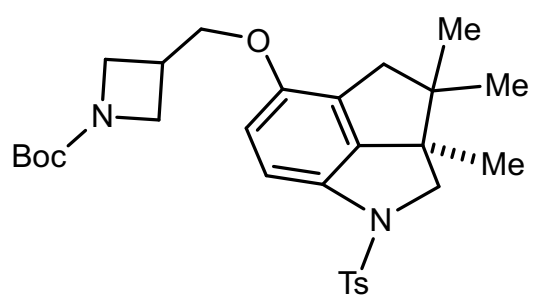


**13l**

tert-butyl 3-(((2a,3,3-trimethyl-1-tosyl-2,2a,3,4-tetrahydro-1H-cyclopenta[cd]indol-5-yl)oxy)methyl)azetidine-1-carboxylate (**13l**). Prepared according to general procedure E: $^1$H NMR (400 MHz, $CDCl_3$) δ 7.70 (d, *J* = 8.3 Hz, 2H), 7.24 (d, *J* = 7.8 Hz, 2H), 7.12 (d, *J* = 8.2 Hz, 1H), 6.54 (d, *J* = 8.3 Hz, 1H), 4.09 – 3.97 (m, 4H), 3.81 – 3.72 (m, 3H), 3.60 (d, *J* = 10.3 Hz, 1H), 3.13 (d, *J* = 15.3 Hz, 1H), 2.92 – 2.83 (m, 1H), 2.52 (d, *J* = 15.3 Hz, 1H), 2.37 (s, 3H), 1.44 (s, 9H), 1.11 (s, 3H), 0.97 (s, 3H), 0.61 (s, 3H). $^{13}$C NMR (101 MHz, $CDCl_3$) δ 156.5, 152.8, 152.7, 143.9, 134.3, 132.2, 129.7, 127.5, 125.5, 114.2, 114.2, 79.6, 71.1, 62.6, 51.3, 51.1, 48.5, 28.6, 28.5, 25.8, 23.6, 21.7, 21.2. HRMS (ESI-TOF) *m/z*: $[M + H]^+$ calcd for $C_{29}H_{39}N_2O_5S$, 527.2574; found, 527.2575.

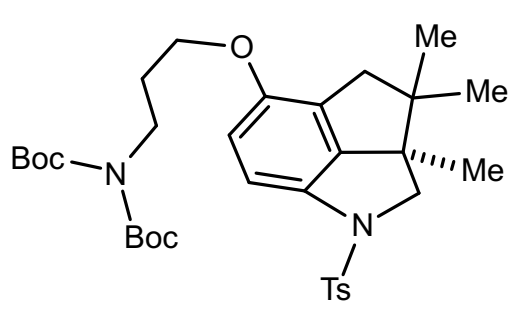


**13m**

tert-butyl (tert-butoxycarbonyl)(3-((2a,3,3-trimethyl-1-tosyl-2,2a,3,4-tetrahydro-1H-cyclopenta[cd]indol-5-yl)oxy)propyl)carbamate (**13m**). Prepared according to general procedure E: $^1$H NMR (400 MHz, $CDCl_3$) δ 7.69 (d, *J* = 8.4 Hz, 2H), 7.22 (d, *J* = 7.8 Hz, 2H), 7.09 (d, *J* = 8.3 Hz, 1H), 6.51 (d, *J* = 8.3 Hz, 1H), 4.01 – 3.87 (m, 2H), 3.79 – 3.72 (m, 3H), 3.59 (d, *J* = 10.4 Hz, 1H), 3.13 (d, *J* = 15.3 Hz, 1H), 2.54 (d, *J* = 15.4 Hz, 1H), 2.36 (s, 3H), 2.02 – 1.95 (m, 2H), 1.47 (s, 18H), 1.10 (s, 3H), 0.95 (s, 3H), 0.61 (s, 3H). $^{13}$C NMR (101 MHz, $CDCl_3$) δ 153.0, 152.7, 152.5, 143.8, 134.3, 131.6, 129.6, 127.5, 125.0, 114.1, 113.6, 82.4, 67.1, 62.5, 51.3, 50.9, 48.6, 43.9, 29.3, 28.2, 25.8, 23.6, 21.6, 21.2. HRMS (ESI-TOF) *m/z*: $[M + Na]^+$ calcd for $C_{33}H_{46}N_2NaO_7S$, 637.2918; found, 637.2919.

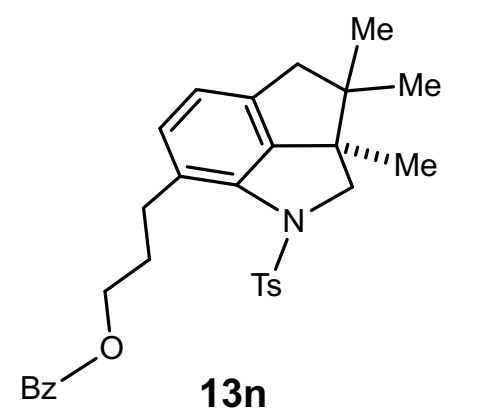


**13n**

3-(2a,3,3-trimethyl-1-tosyl-2,2a,3,4-tetrahydro-1H-cyclopenta[cd]indol-7-yl)propyl benzoate (**13n**). Prepared according to general procedure E: $^{1}$H NMR (400 MHz, $CDCl_3$) δ 8.09 – 8.05 (m, 2H), 7.75 (d, *J* = 8.4 Hz, 2H), 7.57 – 7.52 (m, 1H), 7.43 (t, *J* = 7.5 Hz, 2H), 7.25 (d, *J* = 8.4 Hz, 2H), 6.92 (d, *J* = 7.5 Hz, 1H), 6.81 (d, *J* = 7.6 Hz, 1H), 4.36 – 4.27 (m, 2H), 4.11 (d, *J* = 11.8 Hz, 1H), 3.78 (d, *J* = 11.9 Hz, 1H), 3.21 (d, *J* = 15.0 Hz, 1H), 3.16 – 3.07 (m, 1H), 2.97 – 2.89 (m, 1H), 2.40 – 2.35 (m, 4H), 2.20 – 2.09 (m, 2H), 1.08 (s, 3H), 0.72 (d, *J* = 6.6 Hz, 6H). $^{13}$C NMR (101 MHz, $CDCl_3$) δ 166.8, 153.9, 143.8, 137.6, 137.4, 137.2, 132.9, 131.6, 131.1, 130.7, 129.8, 129.8, 128.4, 127.4, 123.1, 64.9, 64.0, 51.7, 50.9, 50.2, 29.4, 29.4, 25.7, 23.9, 21.7, 20.1. HRMS (ESI-TOF) *m/z*: $[M + H]^+$ calcd for $C_{30}H_{34}NO_4S$, 504.2203; found, 504.2210.

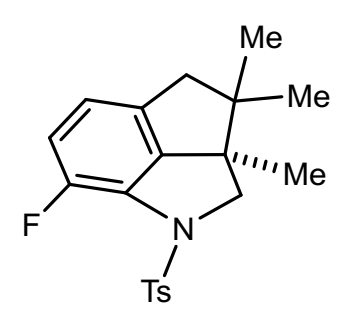


**13o**

7-fluoro-2a,3,3-trimethyl-1-tosyl-2,2a,2a1,3,4,4a-hexahydro-1H-cyclopenta[cd]indole (**13o**). Prepared according to general procedure E: $^{1}$H NMR (500 MHz, $CDCl_3$) δ 7.82 (d, *J* = 8.3 Hz, 2H), 7.29 (d, *J* = 8.0 Hz, 2H), 6.78 – 6.68 (m, 2H), 4.02 (d, *J* = 10.5 Hz, 1H), 3.84 (d, *J* = 10.6 Hz, 1H), 3.19 (d, *J* = 15.2 Hz, 1H), 2.39 (d, *J* = 11.6 Hz, 4H), 1.14 (s, 3H), 1.06 (s, 3H), 0.75 (s, 3H). $^{13}$C NMR (126 MHz, $CDCl_3$) δ 154.6 (d, J = 8.8 Hz), 149.8 (d, J = 253.3 Hz), 143.9, 136.0, 133.9 (d, J = 10.1 Hz), 129.8, 127.6 (d, J = 7.6 Hz), 123.7 (d, J = 10.1 Hz), 122.4 (d, J = 7.6 Hz), 117.4 (d, J = 23.9 Hz), 64.1, 52.0, 51.1, 49.7, 25.8, 23.9, 21.7, 20.5. $^{19}$F NMR (471 MHz, $CDCl_3$) δ -124.8. HRMS (ESI-TOF) *m/z*: $[M + H]^+$ calcd for $C_{20}H_{23}FNO_2S$, 360.1428; found, 360.1437.

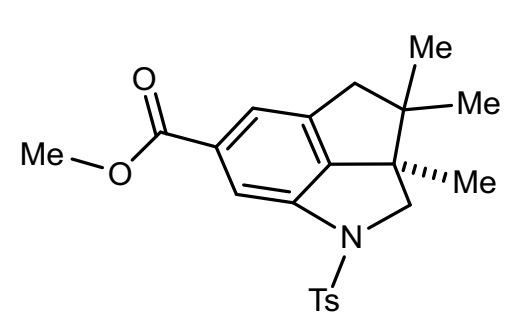


**13p**

methyl 2a,3,3-trimethyl-1-tosyl-2,2a,3,4-tetrahydro-1H-cyclopenta[cd]indole-6-carboxylate (**13p**). Prepared according to general procedure E: $^1$H NMR (400 MHz, $CDCl_3$) δ 7.88 (s, 1H), 7.72 (d, *J* = 8.4 Hz, 2H), 7.52 (s, 1H), 7.25 (d, *J* = 8.4 Hz, 2H), 3.91 (s, 3H), 3.79 (s, 1H), 3.68 (d, *J* = 10.1 Hz, 1H), 3.26 (d, *J* = 15.4 Hz, 1H), 2.43 (d, *J* = 15.4 Hz, 1H), 2.37 (s, 3H), 1.15 (s, 3H), 1.08 (s, 3H), 0.58 (s, 3H). $^{13}$C NMR (101 MHz, $CDCl_3$) δ 167.4, 155.2, 144.3, 138.6, 138.3, 134.1, 132.3, 129.8, 127.4, 122.7, 113.9, 62.7, 52.3, 51.5, 51.1, 50.1, 25.6, 23.7, 21.7, 20.8. HRMS (ESI-TOF) *m/z*: $[M + H]^+$ calcd for $C_{22}H_{26}NO_4S$, 400.1577; found, 400.1581.

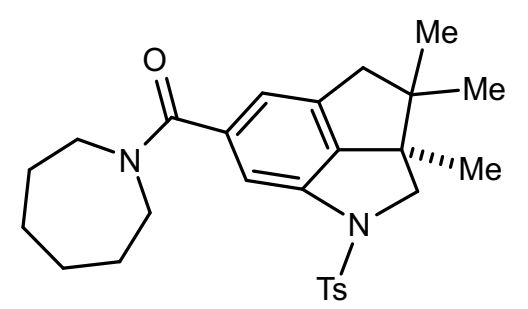


**13q**

azepan-1-yl(2a,3,3-trimethyl-1-tosyl-2,2a,3,4-tetrahydro-1H-cyclopenta[cd]indol-6-yl)methanone (**13q**). Prepared according to general procedure E: $^1$H NMR (400 MHz, $CDCl_3$) δ 7.70 (d, *J* = 8.3 Hz, 2H), 7.24 (d, *J* = 8.0 Hz, 2H), 7.18 (s, 1H), 6.80 (s, 1H), 3.75 (d, *J* = 10.0 Hz, 1H), 3.70 – 3.64 (m, 3H), 3.41 – 3.37 (m, 2H), 3.24 (d, *J* = 15.4 Hz, 1H), 2.40 (d, *J* = 15.4 Hz, 1H), 2.37 (s, 3H), 1.87 – 1.82 (m, 2H), 1.67 – 1.57 (m, 8H), 1.14 (s, 3H), 1.12 (s, 3H), 0.57 (s, 3H). $^{13}$C NMR (101 MHz, $CDCl_3$) δ 171.7, 150.5, 144.2, 138.9, 138.8, 137.8, 133.7, 129.7, 127.3, 119.0, 110.3, 62.8, 51.4, 50.8, 50.2, 49.9, 46.4, 36.6, 29.5, 28.0, 27.4, 26.6, 25.6, 23.7, 21.5, 20.8. HRMS (ESI-TOF) *m/z*: $[M + H]^+$ calcd for $C_{27}H_{35}N_2O_3S$, 467.2363; found, 467.2371.

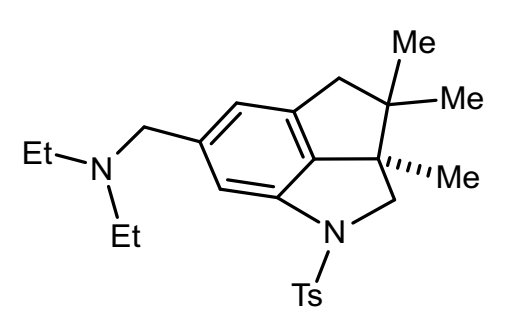


**13r**

N-ethyl-N-((2a,3,3-trimethyl-1-tosyl-2,2a,3,4-tetrahydro-1H-cyclopenta[cd]indol-6-yl)methyl)ethanamine (**13r**). Prepared according to general procedure E: $^1$H NMR (400 MHz, $CDCl_3$) δ 7.71 (d, *J* = 8.3 Hz, 2H), 7.21 (d, *J* = 8.0 Hz, 2H), 7.16 (s, 1H), 6.76 (s, 1H), 3.76 (d, *J* = 10.2 Hz, 1H), 3.64 (d, *J* = 10.1 Hz, 1H), 3.52 (d, *J* = 3.0 Hz, 2H), 3.21 (d, *J* = 15.2 Hz, 1H), 2.53 (qd, *J* = 7.1, 2.6 Hz, 4H), 2.36 (s, 3H), 2.32 (s, 1H), 1.11 (s, 3H), 1.09 – 1.04 (m, 9H), 0.53 (s, 3H). $^{13}$C NMR (101 MHz, $CDCl_3$) δ 148.6, 143.8, 142.1, 138.7, 138.1, 134.1, 129.4, 127.4, 121.0, 113.0, 62.9, 58.1, 51.4, 50.6, 50.2, 46.6, 25.5, 23.7, 21.5, 21.1, 11.7. HRMS (ESI-TOF) *m/z*: $[M + H]^+$ calcd for $C_{25}H_{35}N_2O_2S$, 427.2414; found, 427.2418.

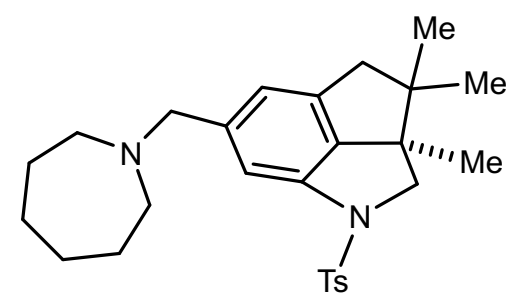

**13s**

6-(azepan-1-ylmethyl)-2a,3,3-trimethyl-1-tosyl-2,2a,3,4-tetrahydro-1H-cyclopenta[cd]indole (**13s**). Prepared according to general procedure E: $^1$H NMR (400 MHz, $CDCl_3$) δ 7.71 (d, *J* = 8.4 Hz, 2H), 7.21 (d, *J* = 7.9 Hz, 3H), 7.18 (s, 1H), 6.75 (s, 1H), 3.76 (d, *J* = 10.2 Hz, 1H), 3.66 – 3.55 (m, 3H), 3.22 (d, *J* = 15.1 Hz, 1H), 2.68 – 2.59 (m, 4H), 2.36 (s, 3H), 2.33 (d, *J* = 15.1 Hz, 1H), 1.64 (s, 8H), 1.11 (s, 3H), 1.06 (s, 3H), 0.53 (s, 3H). $^{13}$C NMR (101 MHz, $CDCl_3$) δ 148.7, 143.9, 142.3, 138.7, 138.3, 134.2, 129.6, 127.5, 121.0, 113.0, 63.4, 63.1, 55.6, 51.6, 50.7, 50.4, 28.2, 27.2, 25.7, 23.8, 21.7, 21.2. HRMS (ESI-TOF) *m/z*: $[M + H]^+$ calcd for $C_{27}H_{37}N_2O_2S$, 453.2570; found, 453.2573.

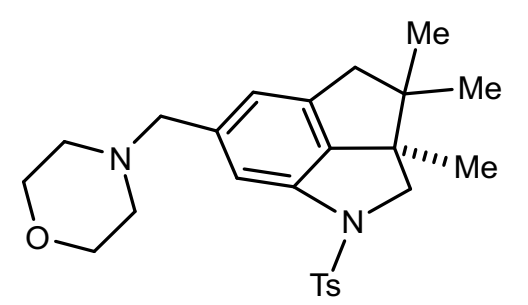

**13t**

4-((2a,3,3-trimethyl-1-tosyl-2,2a,3,4-tetrahydro-1H-cyclopenta[cd]indol-6-yl)methyl)morpholine (**13t**). Prepared according to general procedure E: $^1$H NMR (400 MHz, $CDCl_3$) δ 7.71 (d, *J* = 8.3 Hz, 2H), 7.21 (d, *J* = 8.1 Hz, 2H), 7.17 (s, 1H), 6.72 (s, 1H), 3.78 (s, 1H), 3.74 (s, 4H), 3.65 (d, *J* = 10.1 Hz, 1H), 3.50 (d, *J* = 12.7 Hz, 1H), 3.41 (d, *J* = 11.3 Hz, 1H), 3.22 (d, *J* = 15.2 Hz, 1H), 2.44 (s, 4H), 2.37 (s, 3H), 2.34 (d, *J* = 9.8 Hz, 1H), 1.12 (s, 3H), 1.06 (s, 3H), 0.54 (s, 3H). $^{13}$C NMR (101 MHz, $CDCl_3$) δ 149.2, 144.0, 139.9, 138.9, 138.4, 134.2, 129.6, 127.5, 121.4, 113.4, 67.2, 64.3, 63.1, 53.7, 51.6, 50.7, 50.4, 25.7, 23.8, 21.7, 21.1. HRMS (ESI-TOF) *m/z*: $[M + H]^+$ calcd for $C_{25}H_{33}N_2O_3S$, 441.2207; found, 441.2217.

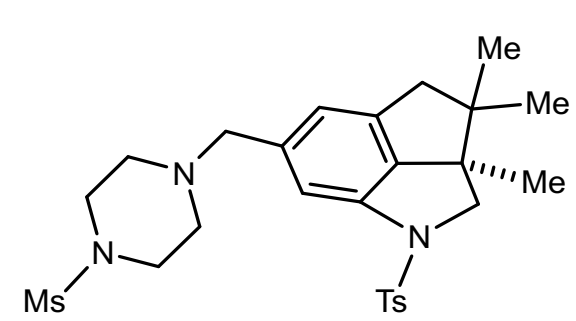

**13u**

2a,3,3-trimethyl-6-((4-(methylsulfonyl)piperazin-1-yl)methyl)-1-tosyl-2,2a,3,4-tetrahydro-1H-cyclopenta[cd]indole (**13u**). Prepared according to general procedure E: $^1$H NMR (400 MHz, $CDCl_3$) δ 7.70 (d, *J* = 8.3 Hz, 2H), 7.24 – 7.18 (m, 3H), 6.68 (s, 1H), 3.78 (d, *J* = 10.2 Hz, 1H), 3.64 (d, *J* = 10.2 Hz, 1H), 3.57 – 3.47 (m, 2H), 3.30 – 3.20 (m, 5H), 2.80 (d, *J* = 1.8 Hz, 3H), 2.56 (s, 4H), 2.38 (s, 3H), 2.35 (d, *J* = 11.4 Hz, 1H), 1.12 (s, 3H), 1.05 (s, 3H), 0.56 (s, 3H). $^{13}$C NMR (101 MHz, $CDCl_3$) δ 149.4, 144.2, 139.6, 139.0, 138.6, 134.3, 129.6, 127.4, 121.3, 113.1, 63.4,

63.0, 52.3, 51.6, 50.8, 50.4, 46.1, 34.3, 25.7, 23.8, 21.7, 21.2. HRMS (ESI-TOF) *m/z*: $[M + H]^+$ calcd for $C_{26}H_{36}N_3O_4S_2$, 518.2142; found, 518.2142.

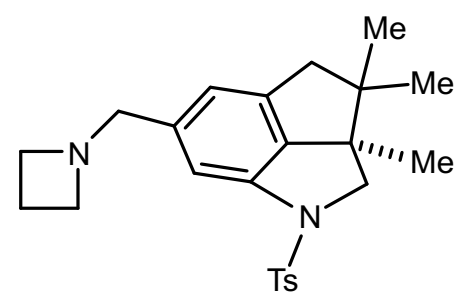

**13v**

6-(azetidin-1-ylmethyl)-2a,3,3-trimethyl-1-tosyl-2,2a,3,4-tetrahydro-1H-cyclopenta[cd]indole (**13v**). Prepared according to general procedure E: $^1$H NMR (400 MHz, $CDCl_3$) δ 7.70 (d, *J* = 8.3 Hz, 2H), 7.24 (d, *J* = 8.0 Hz, 2H), 7.11 (s, 1H), 6.92 (s, 1H), 3.79 (m, 3H), 3.63 (m, 5H), 3.22 (d, *J* = 15.3 Hz, 1H), 2.39 (m, 4H), 2.29 (s, 2H), 1.11 (s, 3H), 1.02 (s, 3H), 0.55 (s, 3H). $^{13}$C NMR (101 MHz, $CDCl_3$) δ 144.2, 138.5, 134.3, 129.8, 127.4, 113.5, 63.0, 51.6, 50.9, 50.4, 25.7, 23.7, 21.7, 21.1. HRMS (ESI-TOF) *m/z*: $[M + H]^+$ calcd for $C_{24}H_{31}N_2O_2S$, 411.2101; found, 411.2104.

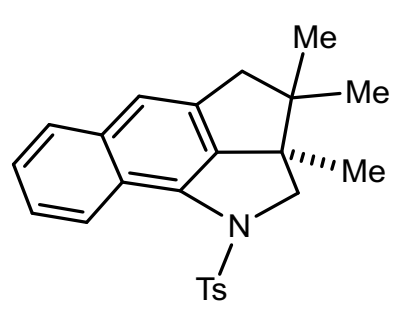

**13w**

2a,3,3-trimethyl-1-tosyl-2,2a,3,4-tetrahydro-1H-benzo[g]cyclopenta[cd]indole (**13w**). Prepared according to general procedure E. $^1$H NMR (400 MHz, $CDCl_3$) δ 8.87 (d, *J* = 8.7 Hz, 1H), 7.79 (d, *J* = 8.2 Hz, 1H), 7.72 (d, *J* = 8.3 Hz, 2H), 7.53 – 7.47 (m, 1H), 7.44 – 7.39 (m, 1H), 7.35 (s, 1H), 7.22 (d, *J* = 7.9 Hz, 2H), 4.33 (d, *J* = 12.0 Hz, 1H), 3.95 (d, *J* = 12.0 Hz, 1H), 3.34 (d, *J* = 15.2 Hz, 1H), 2.52 (d, *J* = 15.1 Hz, 1H), 2.37 (s, 3H), 1.11 (s, 3H), 0.73 (s, 3H), 0.66 (s, 3H). $^{13}$C NMR (101 MHz, $CDCl_3$) δ 151.5, 143.8, 137.7, 137.3, 136.2, 134.1, 129.8, 129.2, 127.8, 126.1, 125.3, 124.8, 123.1, 65.6, 51.6, 50.1, 50.0, 25.5, 23.9, 21.7, 20.8. HRMS (ESI-TOF) *m/z*: $[M + H]^+$ calcd for $C_{24}H_{26}NO_2S$, 392.1679; found, 392.1684.

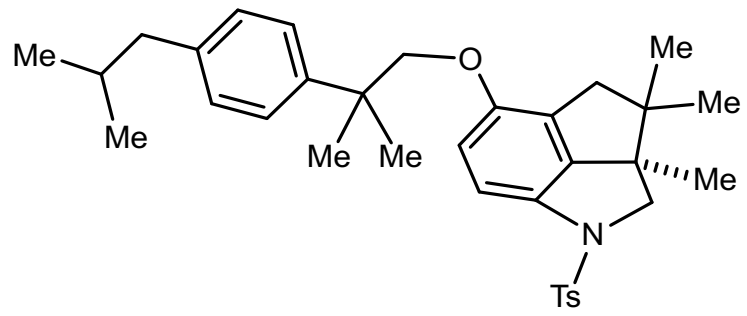

**13x**

5-(2-(4-isobutylphenyl)-2-methylpropoxy)-2a,3,3-trimethyl-1-tosyl-2,2a,3,4-tetrahydro-1H-cyclopenta[cd]indole (**13x**). Prepared according to general procedure E. $^{1}$H NMR (400 MHz, $CDCl_3$) δ 7.69 (d, $J$ = 8.3 Hz, 2H), 7.30 (d, $J$ = 8.3 Hz, 2H), 7.22 (d, $J$ = 8.1 Hz, 2H), 7.10 – 7.06 (m, 3H), 6.51 (d, $J$ = 8.3 Hz, 1H), 3.93 (d, $J$ = 8.8 Hz, 1H), 3.82 – 3.74 (m, 2H), 3.58 (d, $J$ = 10.4 Hz, 1H), 3.09 (d, $J$ = 15.3 Hz, 1H), 2.46 – 2.42 (m, 3H), 2.37 (s, 3H), 1.88 – 1.81 (m, 1H), 1.40 (s, 6H), 1.08 (s, 3H), 0.94 (s, 3H), 0.90 (d, $J$ = 6.6 Hz, 6H), 0.59 (s, 3H). $^{13}$C NMR (101 MHz, $CDCl_3$) δ 153.5, 152.3, 144.1, 143.7, 139.4, 134.2, 131.6, 129.5, 128.9, 127.3, 125.8, 125.5, 114.2, 114.0, 79.2, 62.5, 51.2, 50.9, 48.3, 44.9, 38.8, 30.2, 29.7, 25.8, 25.8, 25.7, 23.5, 22.5, 21.5, 21.1. HRMS (ESI-TOF) $m/z$: $[M + H]^+$ calcd for $C_{34}H_{44}NO_3S$, 546.3037; found, 546.3038.

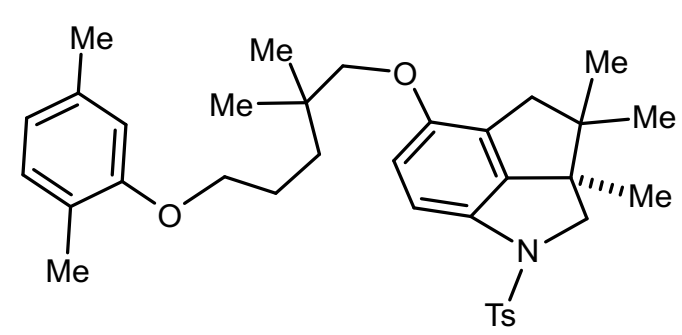


**13y**

5-((5-(2,5-dimethylphenoxy)-2,2-dimethylpentyl)oxy)-2a,3,3-trimethyl-1-tosyl-2,2a,3,4-tetrahydro-1H-cyclopenta[cd]indole (**13y**). Prepared according to general procedure E: $^{1}$H NMR (400 MHz, $CDCl_3$) δ 7.64 (d, $J$ = 8.3 Hz, 2H), 7.16 (d, $J$ = 7.8 Hz, 2H), 7.04 (d, $J$ = 8.3 Hz, 1H), 6.93 (d, $J$ = 7.4 Hz, 1H), 6.61 – 6.56 (m, 1H), 6.54 (d, $J$ = 1.6 Hz, 1H), 6.47 (d, $J$ = 8.3 Hz, 1H), 3.85 (t, $J$ = 6.4 Hz, 2H), 3.73 (d, $J$ = 10.3 Hz, 1H), 3.60 (d, $J$ = 8.7 Hz, 1H), 3.54 – 3.45 (m, 2H), 3.08 (d, $J$ = 15.4 Hz, 1H), 2.47 (d, $J$ = 15.3 Hz, 1H), 2.30 (s, 3H), 2.23 (s, 3H), 2.10 (s, 3H), 1.75 – 1.67 (m, 2H), 1.46 – 1.42 (m, 2H), 1.03 (s, 3H), 0.93 (d, $J$ = 1.6 Hz, 6H), 0.89 (d, $J$ = 2.9 Hz, 3H), 0.55 (s, 3H). $^{13}$C NMR (101 MHz, $CDCl_3$) δ 157.0, 153.6, 152.4, 143.7, 136.5, 134.3, 131.5, 130.3, 129.5, 127.3, 125.2, 123.6, 120.7, 114.0, 113.8, 112.0, 78.0, 68.5, 62.5, 51.2, 50.9, 48.4, 35.4, 34.4, 25.7, 24.4, 24.4, 24.2, 23.6, 21.5, 21.4, 21.1, 15.8. HRMS (ESI-TOF) $m/z$: $[M + H]^+$ calcd for $C_{35}H_{46}NO_4S$, 576.3142; found, 576.3143.

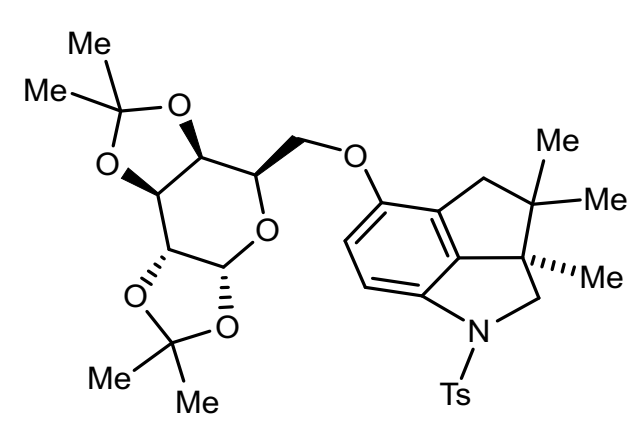


**13z**

2a,3,3-trimethyl-5-(((3aR,5R,5aS,8aS,8bR)-2,2,7,7-tetramethyltetrahydro-5H-bis([1,3]dioxolo)[4,5-b:4',5'-d]pyran-5-yl)methoxy)-1-tosyl-2,2a,3,4-tetrahydro-1H-cyclopenta[cd]indole (**13z**). Prepared according to general procedure E: $^{1}$H NMR (400 MHz, $CDCl_3$) δ 7.71 (d, $J$ = 7.7 Hz, 2H), 7.24 (s, 2H), 7.12 (d, $J$ = 8.3 Hz, 1H), 6.64 (dd, $J$ = 8.3, 2.9 Hz, 1H), 5.92 (dd, $J$ = 6.8, 3.8 Hz, 1H), 4.58 (ddd, $J$ = 20.1, 13.5, 3.8 Hz, 2H), 4.40 (dtd, $J$ = 7.0, 5.9,

1.0 Hz, 1H), 4.26 (dt, *J* = 7.5, 3.2 Hz, 1H), 4.14 – 4.03 (m, 2H), 3.81 (dd, *J* = 10.3, 4.3 Hz, 1H), 3.62 (dd, *J* = 10.3, 1.0 Hz, 1H), 3.14 (dd, *J* = 15.4, 3.5 Hz, 1H), 2.58 (dd, *J* = 18.8, 15.4 Hz, 1H), 2.39 (s, 3H), 1.53 (d, *J* = 6.2 Hz, 3H), 1.42 (d, *J* = 11.0 Hz, 3H), 1.32 – 1.28 (m, 6H), 1.12 (s, 3H), 0.98 (s, 3H), 0.64 (s, 3H). $^{13}$C NMR (101 MHz, $CDCl_3$) δ 152.7, 152.6, 151.0, 150.7, 143.9, 143.9, 134.2, 133.0, 132.4, 129.6, 127.3, 126.7, 125.7, 116.7, 114.6, 114.2, 114.0, 112.1, 112.1, 109.1, 105.2, 82.5, 82.1, 82.0, 81.1, 80.7, 80.6, 72.4, 72.4, 67.1, 67.1, 62.5, 51.2, 51.2, 51.1, 51.0, 48.4, 34.7, 31.6, 26.9, 26.8, 26.8, 26.3, 26.3, 25.7, 25.6, 25.4, 25.3, 25.3, 22.7, 21.6, 21.1, 21.0, 14.1. HRMS (ESI-TOF) *m/z*: $[M + H]^+$ calcd for $C_{32}H_{42}NO_8S$, 600.2626; found, 600.2627.

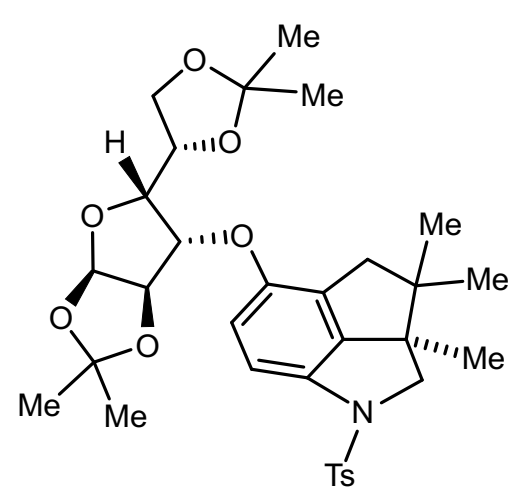


**13aa**

5-(((3aR,5R,6S,6aR)-5-((S)-2,2-dimethyl-1,3-dioxolan-4-yl)-2,2-dimethyltetrahydrofuro[2,3-d][1,3]dioxol-6-yl)oxy)-2a,3,3-trimethyl-1-tosyl-2,2a,3,4-tetrahydro-1H-cyclopenta[cd]indole (**13aa**). Prepared according to general procedure E: $^1$H NMR (400 MHz, $CDCl_3$) δ 7.71 (dd, *J* = 8.3, 2.4 Hz, 2H), 7.25 (d, *J* = 8.2 Hz, 2H), 7.12 (dd, *J* = 8.3, 2.4 Hz, 1H), 6.62 (dd, *J* = 8.3, 4.7 Hz, 1H), 5.59 – 5.54 (m, 1H), 4.68 – 4.62 (m, 1H), 4.37 – 4.31 (m, 2H), 4.22 – 4.01 (m, 4H), 3.82 – 3.74 (m, 1H), 3.62 (d, *J* = 10.3 Hz, 1H), 3.18 (d, *J* = 15.3 Hz, 1H), 2.64 – 2.55 (m, 1H), 2.39 (s, 4H), 1.52 – 1.46 (m, 7H), 1.38 – 1.33 (m, 7H), 1.12 (s, 3H), 0.99 (s, 3H), 0.62 (d, *J* = 4.6 Hz, 3H). $^{13}$C NMR (101 MHz, $CDCl_3$) δ 152.8, 152.7, 152.5, 143.9, 134.2, 134.1, 132.1, 132.0, 129.6, 127.6, 127.5, 125.5, 125.4, 115.2, 114.6, 114.1, 114.1, 109.6, 109.6, 108.8, 108.8, 96.5, 96.5, 71.1, 71.0, 70.8, 70.8, 70.7, 68.5, 68.2, 66.4, 66.4, 62.6, 51.3, 51.0, 48.7, 48.5, 34.8, 31.7, 26.2, 26.2, 26.1, 25.8, 25.4, 25.1, 25.1, 24.6, 24.6, 23.7, 22.8, 21.7, 21.2, 14.3. HRMS (ESI-TOF) *m/z*: $[M + H]^+$ calcd for $C_{32}H_{42}NO_8S$, 600.2626; found, 600.2628.

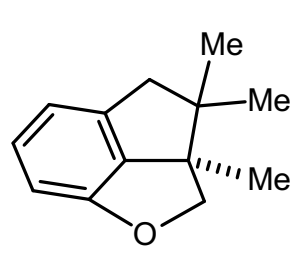


**13ab**

2a,3,3-trimethyl-2,2a,3,4-tetrahydroindeno[7,1-bc]furan (**13ab**). Prepared according to general procedure E: $^1$H NMR (400 MHz, $CDCl_3$) δ 6.96 (t, *J* = 7.5 Hz, 1H), 6.65 (d, *J* = 7.3 Hz, 1H), 6.50 (d, *J* = 7.8 Hz, 1H), 4.60 – 4.56 (m, 1H), 4.32 (d, *J* = 8.7 Hz, 1H), 3.46 (d, *J* = 15.0 Hz, 1H), 2.51 (d, *J* = 15.0 Hz, 1H), 1.39 (s, 3H), 1.21 (s, 3H), 0.85 (s, 3H). $^{13}$C NMR (101 MHz, $CDCl_3$) δ 157.5, 140.3, 129.6, 117.4, 107.4, 85.6, 52.9, 52.6, 52.1, 26.1, 24.2, 20.8. HRMS (ESI-TOF) *m/z*: $[M + H]^+$ calcd for $C_{13}H_{17}O$, 189.1274; found, 189.1281.

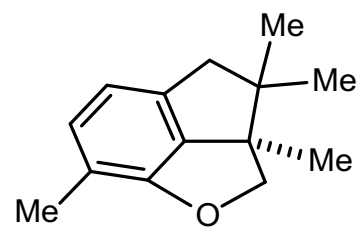

**13ac**

2a,3,3,7-tetramethyl-2,2a,3,4-tetrahydroindeno[7,1-bc]furan (**13ac**). Prepared according to general procedure E: $^{1}$H NMR (400 MHz, $CDCl_3$) δ 6.76 (d, *J* = 7.2 Hz, 1H), 6.56 (d, *J* = 7.2 Hz, 1H), 4.56 (d, *J* = 8.7 Hz, 1H), 4.31 (d, *J* = 8.7 Hz, 1H), 3.42 (d, *J* = 15.0 Hz, 1H), 2.47 (d, *J* = 14.9 Hz, 1H), 2.18 (s, 3H), 1.38 (s, 3H), 1.19 (s, 3H), 0.86 (s, 3H). $^{13}$C NMR (101 MHz, $CDCl_3$) δ 155.5, 147.3, 137.1, 130.8, 117.6, 117.4, 85.2, 52.9, 52.5, 51.8, 26.0, 24.1, 20.6, 14.7. HRMS (ESI-TOF) *m/z*: $[M + H]^+$ calcd for $C_{14}H_{19}O$, 203.1431; found, 203.1432.

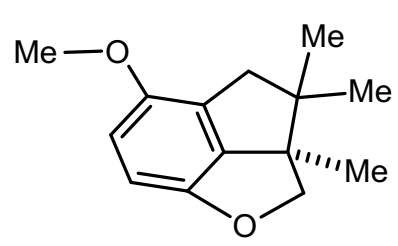

**13ad**

5-methoxy-2a,3,3-trimethyl-2,2a,3,4-tetrahydroindeno[7,1-bc]furan (**13ad**). Prepared according to general procedure E: $^{1}$H NMR (400 MHz, $CDCl_3$) δ 6.24 (s, 1H), 6.12 (s, 1H), 4.61 (d, *J* = 8.8 Hz, 1H), 4.33 (d, *J* = 8.8 Hz, 1H), 3.75 (s, 3H), 3.43 (d, *J* = 15.0 Hz, 1H), 2.42 (d, *J* = 14.9 Hz, 1H), 1.38 (s, 3H), 1.18 (s, 3H), 0.83 (s, 3H). $^{13}$C NMR (101 MHz, $CDCl_3$) δ 162.5, 158.6, 140.7, 139.9, 103.2, 94.6, 86.5, 56.0, 52.9, 52.3, 52.1, 26.0, 24.1, 21.1. HRMS (ESI-TOF) *m/z*: $[M + H]^+$ calcd for $C_{14}H_{19}O_2$, 219.1380; found, 219.1389.

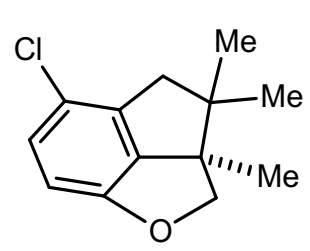

**13ae**

5-chloro-2a,3,3-trimethyl-2,2a,3,4-tetrahydroindeno[7,1-bc]furan (**13ae**). Prepared according to general procedure E: $^{1}$H NMR (400 MHz, $CDCl_3$) δ 6.88 (d, *J* = 8.1 Hz, 1H), 6.46 (d, *J* = 8.1 Hz, 1H), 4.62 (d, *J* = 8.8 Hz, 1H), 4.33 (d, *J* = 8.8 Hz, 1H), 3.40 (d, *J* = 15.4 Hz, 1H), 2.65 (d, *J* = 15.4 Hz, 1H), 1.39 (s, 3H), 1.21 (s, 3H), 0.90 (s, 3H). $^{13}$C NMR (101 MHz, $CDCl_3$) δ 155.9, 149.7, 137.6, 129.3, 122.0, 109.4, 86.2, 53.7, 52.7, 51.0, 26.2, 24.0, 20.7. HRMS (ESI-TOF) *m/z*: $[M + H]^+$ calcd for $C_{13}H_{16}ClO$, 223.0884; found, 223.0890.

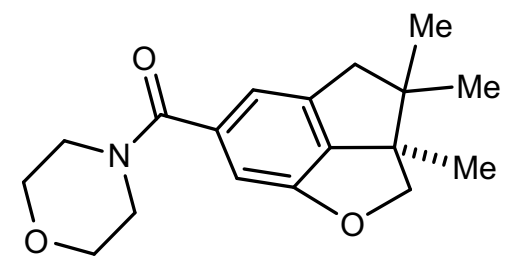


**13af**

morpholino(2a,3,3-trimethyl-2,2a,3,4-tetrahydroindeno[7,1-bc]furan-6-yl)methanone (**13af**). Prepared according to general procedure E: $^1$H NMR (400 MHz, CDCl$_3$) δ 6.74 (s, 1H), 6.55 (s, 1H), 4.61 (d, *J* = 8.8 Hz, 1H), 4.36 (d, *J* = 8.8 Hz, 1H), 3.68 (s, 8H), 3.44 (d, *J* = 15.1 Hz, 1H), 2.53 (d, *J* = 15.1 Hz, 1H), 1.39 (s, 3H), 1.22 (s, 3H), 0.86 (s, 3H). $^{13}$C NMR (101 MHz, CDCl$_3$) δ 171.1, 157.1, 150.1, 140.2, 137.2, 117.1, 106.8, 86.1, 67.1, 52.9, 52.8, 51.9, 26.1, 24.2, 20.7. HRMS (ESI-TOF) *m/z*: [M + H]$^+$ calcd for $C_{18}H_{24}NO_3$, 302.1751; found, 302.1759.

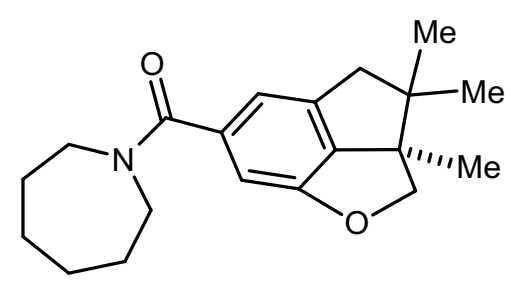


**13ag**

azepan-1-yl(2a,3,3-trimethyl-2,2a,3,4-tetrahydroindeno[7,1-bc]furan-6-yl)methanone (**13ag**). Prepared according to general procedure E: $^1$H NMR (400 MHz, CDCl$_3$) δ 6.69 (s, 1H), 6.52 (s, 1H), 4.60 (d, *J* = 8.8 Hz, 1H), 4.34 (d, *J* = 8.8 Hz, 1H), 3.64 (t, *J* = 5.8 Hz, 2H), 3.44 (d, *J* = 15.1 Hz, 1H), 3.37 (t, *J* = 5.8 Hz, 2H), 2.51 (d, *J* = 15.1 Hz, 1H), 1.81 (s, 2H), 1.62 (s, 6H), 1.39 (s, 3H), 1.21 (s, 3H), 0.86 (s, 3H). $^{13}$C NMR (101 MHz, CDCl$_3$) δ 172.0, 156.9, 148.9, 139.9, 139.2, 116.2, 106.1, 85.9, 52.6, 51.8, 49.8, 46.3, 29.5, 28.0, 27.4, 26.5, 26.0, 24.1, 20.6. HRMS (ESI-TOF) *m/z*: [M + H]$^+$ calcd for $C_{20}H_{28}NO_2$, 314.2115; found, 314.2124.

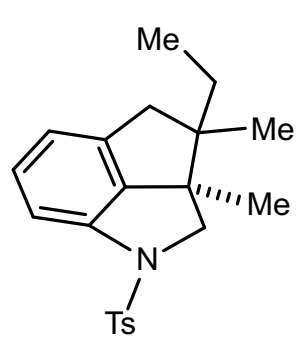


**13ah**

3-ethyl-2a,3-dimethyl-1-tosyl-2,2a,3,4-tetrahydro-1H-cyclopenta[cd]indole (**13ah**). Prepared according to general procedure E: $^1$H NMR (400 MHz, CDCl$_3$) δ 7.76 – 7.68 (m, 3.40H), 7.24 (d, J = 8.1 Hz, 3.40H), 7.21 – 7.14 (m, 1.70H), 7.07 – 7.00 (m, 1.72H), 6.78 – 6.68 (m, 1.70H), 3.82 (t, J = 10.8 Hz, 1.72H), 3.69 – 3.61 (m, 1.72H), 3.18 (d, J = 15.2 Hz, 0.72H), 3.03 (d, J = 15.5 Hz, 1H), 2.63 (d, J = 15.6 Hz, 1H), 2.41 – 2.36 (m, 5.80H), 1.72 – 1.63 (m, 0.72H), 1.50 – 1.42 (m, 0.70H), 1.07 (s, 6H), 1.03 (s, 2.09H), 1.02 – 0.92 (m, 1.35H), 0.81 (t, J = 7.4 Hz, 2.10H), 0.78 – 0.73 (m, 0.72H), 0.56 – 0.51 (m, 5H). $^{13}$C NMR (101 MHz, CDCl$_3$) δ 150.1, 149.8, 144.0, 138.7, 138.6, 138.1, 137.9, 134.4, 134.4, 129.7, 129.7, 129.4, 129.4, 127.5, 127.4, 120.3, 119.9, 112.3,

112.2, 62.9, 62.7, 55.7, 54.5, 51.6, 50.5, 49.7, 45.6, 29.2, 29.1, 21.7, 21.7, 21.1, 20.5, 20.5, 10.1, 9.4. HRMS (ESI-TOF) *m/z*: $[M + H]^+$ calcd for $C_{21}H_{26}NO_2S$, 356.1679; found, 356.1686.

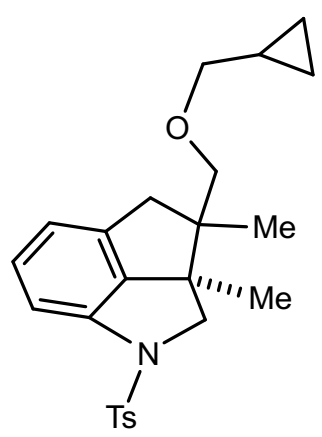


**13ai**

3-((cyclopropylmethoxy)methyl)-2a,3-dimethyl-1-tosyl-2,2a,3,4-tetrahydro-1H-cyclopenta[cd]indole (**13ai**). Prepared according to general procedure E: $^1$H NMR (400 MHz, $CDCl_3$) δ 7.72 (dd, *J* = 8.3, 6.5 Hz, 3H), 7.25 – 7.18 (m, 4.55H), 7.05 (t, *J* = 7.6 Hz, 1.55H), 6.75 (dd, *J* = 7.3, 0.9 Hz, 1H), 6.71 (dd, *J* = 7.3, 1.0 Hz, 0.55H), 3.81 (d, *J* = 10.4 Hz, 1H), 3.75 – 3.69 (m, 2H), 3.60 (d, *J* = 9.0 Hz, 1H), 3.32 – 3.20 (m, 3H), 3.19 – 3.10 (m, 1.55H), 2.83 (dd, *J* = 10.3, 6.6 Hz, 0.54H), 2.74 (s, 1H), 2.74 – 2.69 (m, 0.50H), 2.54 (d, *J* = 15.9 Hz, 0.53H), 2.36 (d, *J* = 2.5 Hz, 4.64H), 2.32 (d, *J* = 15.1 Hz, 1H), 1.19 (s, 1.55H), 1.12 (s, 1.54H), 1.10 (s, 3H), 1.01 (m, 1H), 0.78 (m, 0.53H), 0.64 (s, 3H), 0.54 – 0.49 (m, 2H), 0.42 – 0.38 (m, 1H), 0.21 – 0.16 (m, 2H), 0.04 – -0.01 (m, 1H). $^{13}$C NMR (101 MHz, $CDCl_3$) δ 149.3, 149.2, 143.8, 143.8, 138.3, 138.1, 138.0, 137.6, 134.2, 134.2, 129.5, 129.4, 129.3, 127.4, 127.3, 120.2, 119.4, 112.2, 111.8, 75.5, 75.4, 75.1, 74.3, 64.0, 63.3, 55.3, 55.2, 50.5, 50.1, 46.3, 46.0, 21.5, 21.5, 21.3, 21.2, 20.2, 19.3, 10.5, 10.3, 2.9, 2.9, 2.8, 2.5. HRMS (ESI-TOF) *m/z*: $[M + H]^+$ calcd for $C_{24}H_{30}NO_3S$, 412.1941; found, 412.1949.

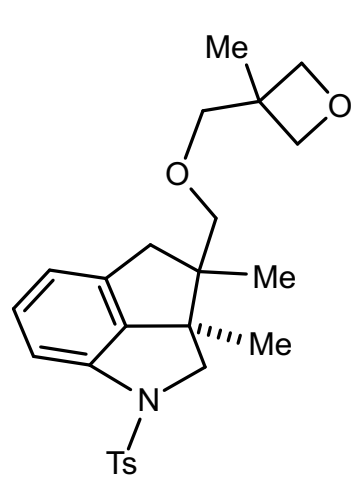


**13aj**

2a,3-dimethyl-3-(((3-methyloxetan-3-yl)methoxy)methyl)-1-tosyl-2,2a,3,4,7,7a-hexahydro-1H-cyclopenta[cd]indole (**13aj**). Prepared according to general procedure E: $^1$H NMR (400 MHz, $CDCl_3$) δ 7.74 – 7.70 (m, 2.41H), 7.26 – 7.22 (m, 2.41H), 7.19 (d, *J* = 7.9 Hz, 1.19H), 7.05 (t, *J* = 7.6 Hz, 1.19H), 6.76 (dd, *J* = 7.2, 1.0 Hz, 1H), 6.72 (dd, *J* = 7.2, 1.0 Hz, 0.18H), 4.48 – 4.45 (m, 2H), 4.39 – 4.36 (m, 2H), 4.34 – 4.31 (m, 0.38H), 4.29 – 4.25 (m, 0.38H), 3.81 (d, *J* = 10.4 Hz, 1H), 3.70 – 3.63 (m, 2.38H), 3.49 (s, 2H), 3.28 (d, *J* = 8.9 Hz, 1H), 3.19 (d, *J* = 15.4 Hz, 1H), 3.05 (d, *J* = 9.0 Hz, 0.18H), 2.92 (d, *J* = 9.0 Hz, 0.18H), 2.87 (d, *J* = 9.0 Hz, 0.19H), 2.80 (d, *J* = 9.0

Hz, 0.17H), 2.54 (d, *J* = 10.9 Hz, 0.21H), 2.38 – 2.32 (m, 4.80H), 1.30 (s, 3H), 1.18 (d, J = 2.2 Hz, 1.21H), 1.10 (s, 0.62H), 1.08 (s, 3H), 0.66 (s, 3H). $^{13}$C NMR (101 MHz, $CDCl_3$) δ 149.2, 149.0, 143.9, 143.8, 138.2, 138.0, 138.0, 137.4, 134.3, 134.3, 129.6, 129.6, 129.5, 129.4, 127.4, 127.3, 120.2, 119.4, 112.3, 112.0, 80.1, 80.1, 79.9, 79.8, 76.5, 75.9, 75.3, 63.9, 63.1, 55.3, 50.5, 50.1, 46.4, 45.9, 40.0, 39.7, 21.5, 21.5, 21.4, 21.3, 21.2, 21.2, 20.3, 19.3. HRMS (ESI-TOF) *m/z*: [M + H]$^+$ calcd for $C_{25}H_{32}NO_4S$, 442.2047; found, 442.2049.

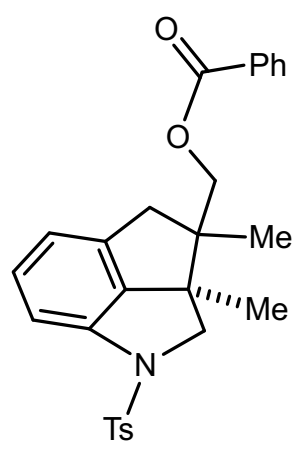


**13ak**

(2a,3-dimethyl-1-tosyl-2,2a,3,4-tetrahydro-1H-cyclopenta[cd]indol-3-yl)methyl benzoate (**13ak**). Prepared according to general procedure E: $^1$H NMR (500 MHz, $CDCl_3$) δ 7.99 (d, *J* = 7.0 Hz, 1.40H), 7.77 – 7.72 (m, 1.42H), 7.72 – 7.69 (m, 2H), 7.64 – 7.57 (m, 1.71H), 7.52 (t, *J* = 7.5 Hz, 3H), 7.43 (t, *J* = 7.7 Hz, 2.10H), 7.26 – 7.20 (m, 3.44H), 7.12 – 7.06 (m, 1.70H), 6.93 (d, *J* = 8.0 Hz, 2H), 6.81 (d, *J* = 7.3 Hz, 0.71H), 6.76 (d, *J* = 7.3 Hz, 1H), 4.44 (d, *J* = 11.2 Hz, 0.70H), 4.34 (d, *J* = 11.1 Hz, 0.71H), 3.99 (d, *J* = 10.4 Hz, 0.70H), 3.81 (q, *J* = 9.8 Hz, 2.71H), 3.73 (d, *J* = 10.0 Hz, 1H), 3.59 (d, *J* = 11.3 Hz, 1H), 3.37 (d, *J* = 15.2 Hz, 0.70H), 3.31 (d, *J* = 15.9 Hz, 1H), 2.71 (d, *J* = 15.9 Hz, 1H), 2.51 (d, *J* = 15.2 Hz, 0.70H), 2.37 (s, 2.10H), 2.25 (s, 3H), 1.27 (s, 6H), 1.10 (s, 2H), 0.79 (s, 2H). $^{13}$C NMR (101 MHz, $CDCl_3$) δ 166.5, 166.0, 148.8, 144.3, 144.2, 138.3, 138.1, 137.6, 137.1, 134.5, 133.4, 133.3, 133.1, 130.0, 130.0, 129.9, 129.8, 129.8, 129.8, 129.6, 129.6, 129.5, 128.8, 128.6, 127.4, 127.2, 120.6, 119.8, 112.9, 112.1, 70.0, 68.8, 63.6, 62.8, 54.4, 54.3, 50.7, 50.3, 46.7, 46.2, 21.7, 21.6, 21.6, 21.1, 20.7, 19.4. HRMS (ESI-TOF) *m/z*: [M + H]$^+$ calcd for $C_{27}H_{28}NO_4S$, 462.1734; found, 462.1742.

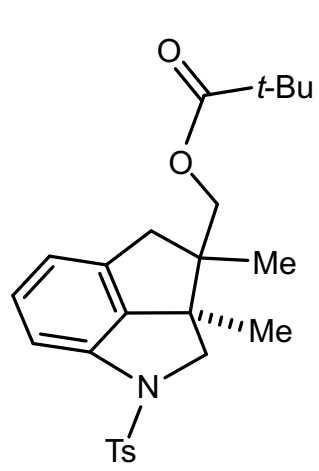


**13al**

(2a,3-dimethyl-1-tosyl-2,2a,3,4-tetrahydro-1H-cyclopenta[cd]indol-3-yl)methyl pivalate (**13al**). Prepared according to general procedure E: $^1$H NMR (500 MHz, $CDCl_3$) δ 7.72 (d, *J* = 8.3 Hz, 2H), 7.68 (d, *J* = 8.3 Hz, 1.20H), 7.26 – 7.17 (m, 4.80H), 7.10 – 7.04 (m, 1.62H), 6.78 (d, *J* = 7.3 Hz, 1H), 6.73 (d, *J* = 7.3 Hz, 0.62H), 4.16 (d, *J* = 11.1 Hz, 1H), 4.05 (d, *J* = 11.1 Hz, 1H), 3.87 (d,

$J$ = 10.4 Hz, 1H), 3.76 (d, $J$ = 10.1 Hz, 0.60H), 3.70 (dd, $J$ = 10.3, 7.2 Hz, 1.62H), 3.60 (d, $J$ = 11.2 Hz, 0.61H), 3.44 (d, $J$ = 11.1 Hz, 0.61H), 3.28 (d, $J$ = 15.2 Hz, 1H), 3.22 (d, $J$ = 15.9 Hz, 0.61H), 2.64 (d, $J$ = 15.9 Hz, 0.61H), 2.41 (d, $J$ = 15.3 Hz, 1H), 2.37 (s, 3H), 2.37 (s, 1.80H), 1.22 (s, 9H), 1.17 (d, $J$ = 6.5 Hz, 3.60H), 1.11 (s, 5.42H), 1.05 (s, 3H), 0.66 (s, 3H). $^{13}$C NMR (101 MHz, $CDCl_3$) δ 178.2, 177.8, 148.5, 148.4, 144.2, 144.0, 138.0, 138.0, 137.2, 137.0, 134.2, 134.0, 129.8, 129.7, 129.7, 129.7, 129.6, 127.2, 127.2, 120.4, 119.6, 112.6, 112.1, 68.7, 68.0, 63.6, 62.7, 54.2, 54.1, 50.5, 50.2, 46.4, 45.9, 38.8, 38.8, 27.3, 27.1, 21.6, 21.5, 21.2, 20.8, 20.5, 19.0. HRMS (ESI-TOF) *m/z*: $[M + H]^+$ calcd for $C_{25}H_{32}NO_4S$, 442.2047; found, 442.2051.

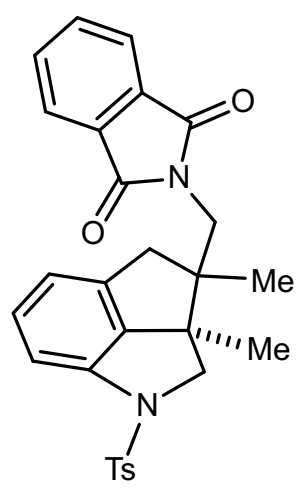


**13am**

2-((2a,3-dimethyl-1-tosyl-2,2a,3,4-tetrahydro-1H-cyclopenta[cd]indol-3-yl)methyl)isoindoline-1,3-dione (**13am**). Prepared according to general procedure E: $^1$H NMR (400 MHz, $CDCl_3$) δ 7.85 (dd, $J$ = 5.5, 3.1 Hz, 2H), 7.80 (dd, $J$ = 5.5, 3.1 Hz, 1.20H), 7.74 (ddd, $J$ = 8.4, 5.6, 2.8 Hz, 6.41H), 7.22 (dd, $J$ = 11.5, 7.9 Hz, 4.81H), 7.16 (t, $J$ = 7.5 Hz, 0.60H), 7.05 (t, $J$ = 7.6 Hz, 1H), 6.91 (d, $J$ = 7.2 Hz, 0.60H), 6.74 (d, $J$ = 7.3 Hz, 1H), 3.97 (t, $J$ = 13.3 Hz, 1.60H), 3.82 – 3.74 (m, 3.65H), 3.66 (d, $J$ = 15.4 Hz, 1H), 3.27 (d, $J$ = 13.4 Hz, 0.60H), 3.00 (d, $J$ = 15.6 Hz, 0.60H), 2.91 (d, $J$ = 13.3 Hz, 0.60H), 2.72 (d, $J$ = 15.6 Hz, 0.60H), 2.39 (d, $J$ = 15.4 Hz, 1H), 2.36 (s, 3H), 2.31 (s, 1.82H), 1.29 (s, 3H), 1.13 (d, $J$ = 3.5 Hz, 3.60H), 0.52 (s, 3H). $^{13}$C NMR (101 MHz, $CDCl_3$) δ 169.1, 168.8, 148.9, 148.8, 144.1, 144.1, 138.2, 138.1, 137.9, 134.4, 134.3, 134.3, 132.0, 130.1, 129.9, 129.8, 129.7, 127.4, 123.6, 123.4, 120.9, 120.3, 112.6, 112.5, 62.4, 61.9, 56.7, 55.3, 51.4, 51.0, 48.6, 45.5, 43.6, 43.5, 21.7, 21.6, 21.5, 20.8, 20.6, 20.2. HRMS (ESI-TOF) *m/z*: $[M + H]^+$ calcd for $C_{28}H_{27}N_2O_4S$, 487.1686; found, 487.1698.

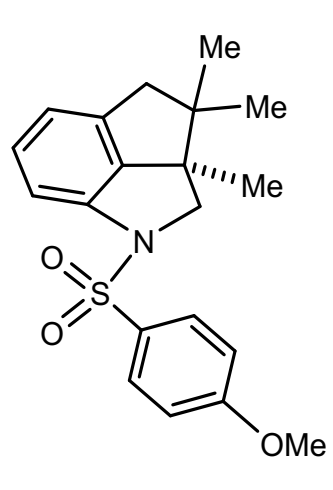


**13an**

1-((4-methoxyphenyl)sulfonyl)-2a,3,3-trimethyl-2,2a,3,4-tetrahydro-1H-cyclopenta[cd]indole (**13an**). Prepared according to general procedure E: $^1$H NMR (400 MHz, $CDCl_3$) δ 7.77 (d, $J$ = 9.0 Hz, 2H), 7.18 (d, $J$ = 7.6 Hz, 1H), 7.04 (t, $J$ = 7.6 Hz, 1H), 6.95 – 6.85 (m, 2H), 6.75 (d, $J$ = 6.1

Hz, 1H), 3.82 (s, 3H), 3.77 (d, $J$ = 10.1 Hz, 1H), 3.63 (d, $J$ = 10.1 Hz, 1H), 3.25 (d, $J$ = 15.3 Hz, 1H), 2.39 (d, $J$ = 15.2 Hz, 1H), 1.13 (s, 3H), 1.07 (s, 3H), 0.58 (s, 3H). $^{13}$C NMR (101 MHz, $CDCl_3$) δ 163.3, 149.8, 139.0, 138.4, 129.5, 129.4, 129.0, 120.2, 114.2, 112.3, 62.7, 55.7, 51.4, 51.0, 50.5, 25.7, 23.8, 21.1. HRMS (ESI-TOF) *m/z*: $[M + H]^+$ calcd for $C_{20}H_{24}NO_3S$, 358.1472; found, 358.1480.

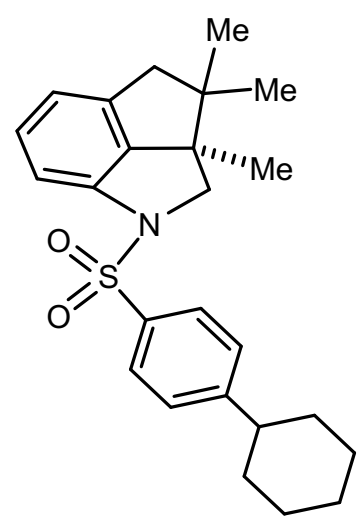


**13ao**

1-((4-cyclohexylphenyl)sulfonyl)-2a,3,3-trimethyl-2,2a,3,4-tetrahydro-1H-cyclopenta[cd]indole (**13ao**). Prepared according to general procedure E: $^1$H NMR (400 MHz, $CDCl_3$) δ 7.73 (d, $J$ = 8.4 Hz, 2H), 7.26 (d, $J$ = 8.3 Hz, 2H), 7.20 (d, $J$ = 7.9 Hz, 1H), 7.05 (t, $J$ = 7.5 Hz, 1H), 6.75 (d, $J$ = 7.2 Hz, 1H), 3.77 (d, $J$ = 10.3 Hz, 1H), 3.62 (d, $J$ = 10.2 Hz, 1H), 3.24 (d, $J$ = 15.4 Hz, 1H), 2.57 – 2.46 (m, 1H), 2.38 (d, $J$ = 15.3 Hz, 1H), 1.85 – 1.77 (m, 4H), 1.40 – 1.19 (m, 6H), 1.12 (s, 3H), 1.01 (s, 3H), 0.54 (s, 3H). $^{13}$C NMR (101 MHz, $CDCl_3$) δ 153.9, 149.9, 139.0, 138.4, 134.5, 129.4, 127.5, 127.5, 120.2, 112.5, 62.6, 51.4, 51.0, 50.5, 44.7, 34.2, 34.2, 26.8, 26.1, 25.5, 23.7, 21.1. HRMS (ESI-TOF) *m/z*: $[M + H]^+$ calcd for $C_{25}H_{32}NO_2S$, 410.2149; found, 410.2151.

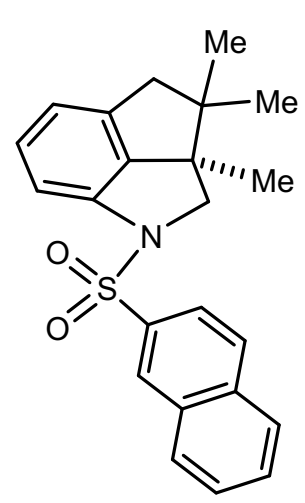


**13ap**

2a,3,3-trimethyl-1-(naphthalen-2-ylsulfonyl)-2,2a,3,4-tetrahydro-1H-cyclopenta[cd]indole (**13ap**). Prepared according to general procedure E: $^1$H NMR (500 MHz, $CDCl_3$) δ 8.44 (s, 1H), 7.95 (d, $J$ = 7.6 Hz, 1H), 7.87 (t, $J$ = 8.6 Hz, 2H), 7.79 (d, $J$ = 8.5 Hz, 1H), 7.64 – 7.57 (m, 2H), 7.28 (d, $J$ = 7.3 Hz, 1H), 7.07 (t, $J$ = 7.6 Hz, 1H), 6.75 (d, $J$ = 7.3 Hz, 1H), 3.88 (d, $J$ = 10.2 Hz, 1H), 3.71 (d, $J$ = 10.2 Hz, 1H), 3.22 (d, $J$ = 15.3 Hz, 1H), 2.37 (d, $J$ = 15.3 Hz, 1H), 1.10 (s, 3H), 1.01 (s, 3H), 0.55 (s, 3H). $^{13}$C NMR (126 MHz, $CDCl_3$) δ 149.8, 139.0, 138.3, 135.1, 134.6, 132.2, 129.5, 129.5, 129.4, 128.9, 128.8, 128.0, 127.7, 122.6, 120.3, 112.4, 62.9, 51.3, 51.1, 50.5, 25.7, 23.8, 21.1. HRMS (ESI-TOF) *m/z*: $[M + H]^+$ calcd for $C_{23}H_{24}NO_2S$, 378.1523; found, 378.1531.

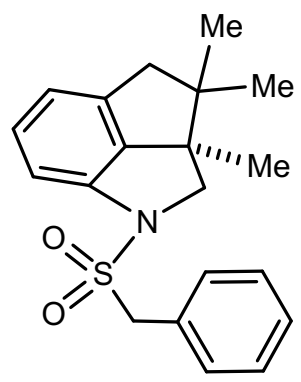

**13aq**

1-(benzylsulfonyl)-2a,3,3-trimethyl-2,2a,3,4-tetrahydro-1H-cyclopenta[cd]indole (**13aq**). Prepared according to general procedure E: $^1$H NMR (400 MHz, $CDCl_3$) δ 7.33 – 7.28 (m, 5H), 7.00 (t, $J$ = 7.5 Hz, 1H), 6.81 – 6.75 (m, 2H), 4.36 (d, $J$ = 2.6 Hz, 2H), 3.66 (d, $J$ = 9.6 Hz, 1H), 3.59 (d, $J$ = 9.6 Hz, 1H), 3.30 (d, $J$ = 15.2 Hz, 1H), 2.47 (d, $J$ = 15.3 Hz, 1H), 1.24 (s, 3H), 1.16 (s, 3H), 0.75 (s, 3H). $^{13}$C NMR (101 MHz, $CDCl_3$) δ 149.0, 139.0, 138.4, 131.0, 129.4, 129.0, 128.9, 128.5, 119.7, 110.3, 64.5, 56.6, 51.5, 50.9, 50.6, 26.3, 23.8, 20.5. HRMS (ESI-TOF) *m/z*: $[M + H]^+$ calcd for $C_{20}H_{24}NO_2S$, 342.1523; found, 342.1533.

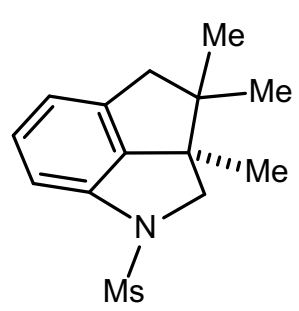

**13ar**

2a,3,3-trimethyl-1-(methylsulfonyl)-2,2a,3,4-tetrahydro-1H-cyclopenta[cd]indole (**13ar**). Prepared according to general procedure E: $^1$H NMR (400 MHz, $CDCl_3$) δ 7.06 (t, $J$ = 7.6 Hz, 1H), 6.96 (d, $J$ = 7.7 Hz, 1H), 6.82 (d, $J$ = 7.3 Hz, 1H), 3.87 (d, $J$ = 9.8 Hz, 1H), 3.75 (d, $J$ = 9.8 Hz, 1H), 3.36 (d, $J$ = 15.2 Hz, 1H), 2.84 (s, 3H), 2.51 (d, $J$ = 15.3 Hz, 1H), 1.36 (s, 3H), 1.24 (s, 3H), 0.87 (s, 3H). $^{13}$C NMR (101 MHz, $CDCl_3$) δ 149.4, 139.3, 138.5, 129.7, 120.3, 110.7, 63.8, 51.6, 51.1, 50.6, 34.6, 26.1, 23.8, 20.8. HRMS (ESI-TOF) *m/z*: $[M + Na]^+$ calcd for $C_{14}H_{19}NNaO_2S$, 288.1029; found, 288.1039.

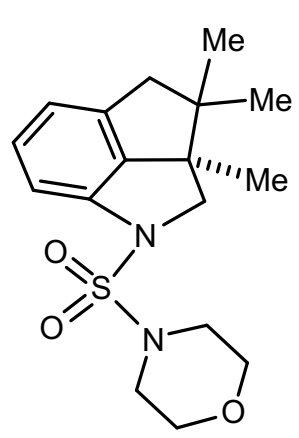

**13as**

4-((2a,3,3-trimethyl-2,2a,3,4-tetrahydro-1H-cyclopenta[cd]indol-1-yl)sulfonyl)morpholine (**13as**). Prepared according to general procedure E: $^1$H NMR (400 MHz, $CDCl_3$) δ 7.06 – 7.00 (m, 1H), 6.95 (d, $J$ = 7.8 Hz, 1H), 6.77 (d, $J$ = 7.3 Hz, 1H), 4.07 (d, $J$ = 9.7 Hz, 1H), 3.77 – 3.70 (m, 4H), 3.65 (d, $J$ = 9.8 Hz, 1H), 3.35 – 3.25 (m, 5H), 2.51 (d, $J$ = 15.3 Hz, 1H), 1.35 (s, 3H), 1.22 (s,

3H), 0.88 (s, 3H). $^{13}C$ NMR (101 MHz, $CDCl_3$) δ 148.6, 139.4, 138.9, 129.4, 119.8, 111.3, 66.4, 64.1, 51.3, 51.1, 50.6, 46.8, 26.1, 23.9, 20.9. HRMS (ESI-TOF) *m/z*: $[M + H]^+$ calcd for $C_{17}H_{25}N_2O_3S$, 337.1581; found, 337.1571.

**Crystallographic Analysis.**

**Crystallographic Analysis for the Indoline 13j.**

Single crystals of the ether **13j** suitable for the X-ray analysis were obtained by the slow evaporation of a solution of compound **13j** in hexane–methanol (4:1 v/v) at 23 °C. Crystals were mounted on a MiTeGen MicroMount with Type B immersion oil (Cargille Labs). Single crystal X-ray intensity data were measured on a Bruker D8 SMART APEXII "three-circle diffractometer" system equipped with an Incoatec "microfocus sealed X-ray tube" (MoK α radiation, λ = 0.71073 Å), a multilayer optics monochromator and a PHOTON-II-C14 detector. Crystal temperature was controlled by an Oxford Cryosystems 700 + Cooler. Full datasets were collected with ω scans at T = 100(2) K. The frames were integrated with the Bruker SAINT software package using a narrow-frame algorithm and the data were corrected for absorption effects using the Multi-Scan method with the SADABS software. The structures were solved by intrinsic phasing methods (SHELXT) and the structure models were completed and refined using the full-matrix least-square methods on F2 (SHELXL). All non-hydrogen atoms were refined with anisotropic displacement parameters, and hydrogen atoms on carbons were placed in idealized positions (C-H = 0.95-1.00 Å) and included using a riding model with Uiso(H) = 1.2 or 1.5 Ueq(non-H). Selected crystallographic parameters are listed in **Table S6**. Crystallographic data of these structures, including cif, res, fcf, and hkl files, have been deposited with the Cambridge Crystallographic Data Centre with deposition numbers 2551161. Copies of these data can be requested, free of charge, from the CCDC website at https://www.ccdc.cam.ac.uk/structures/.

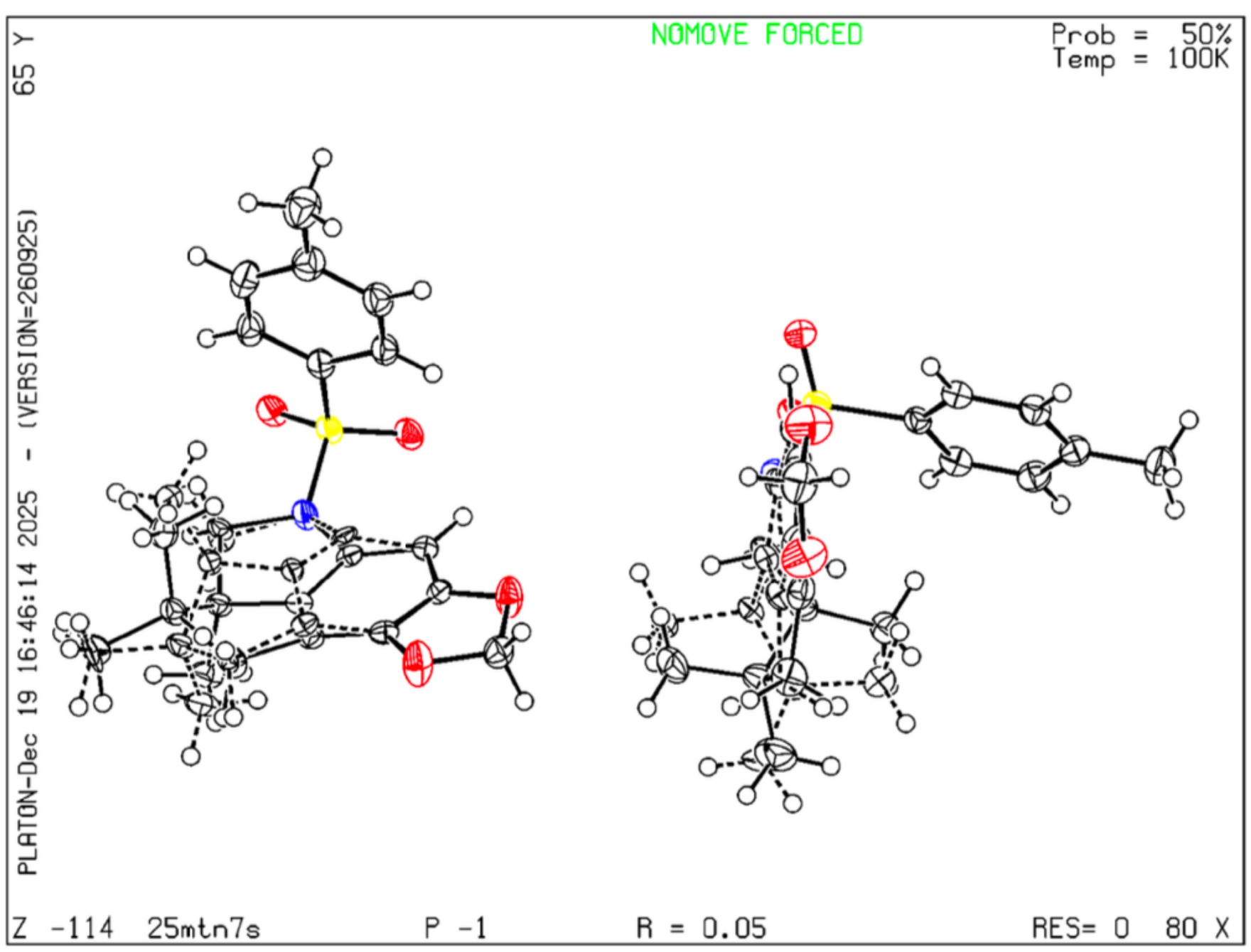


**Figure S1.** The crystal structure of **13j**.

**Table S6**. Crystallographic data and structure refinement for **13j:**

Identification code: 2551161

| | | | |
|---|---|---|---|
| Bond precision: | C–C = 0.0026 Å | | Wavelength=0.71073 |
| Cell: | a = 10. 2850(6) Å | b = 11.6340 (6) Å | c = 16.3302(9) Å |
| | α = 87.171 (2) | β = 76.891(2) | γ = 85.783 (2) |
| Temperature: | 100 K | | |

| | Calculated | Reported |
|---|---|---|
| Volume | 1896.85(17) Å$^3$ | 1896.85(17) |
| Space group | P –1 | P –1 |
| Hall group | –P 1 | –P 1 |
| Moiety formula | C21 H23 N O4 S | |
| Sum formula | C21 H23 N O4 S | C21 H23 N O4 S |
| Mr | 385.46 | 385.46 |
| Dx,g cm-3 | 1.350 | 1.350 |
| Z | 4 | 4 |
| Mu (mm-1) | 0.198 | 0.198 |
| F000 | 816.0 | 816.0 |
| F000' | 816.17 | |
| h,k,lmax | 13,15,21 | 13,15,21 |
| Nref | 9603 | 9573 |
| Tmin,Tmax | 0.927,0.940 | 0.860,0.940 |
| Tmin' | 0.917 | |

Correction method = # Reported T Limits: Tmin=0.860 Tmax=0.940 AbsCorr = MULTI-SCAN

Data completeness = 0.997 Theta(max) = 28.490

R(reflections) = 0.0490( 8601) wR2(reflections) = 0.1270( 9573)

S = 1.064 Npar = 683

## References.


[1] Arisawa, M.; Terada, Y.; Takahashi, K.; Nakagawa, M.; Nishida, A. Development of Isomerization and Cycloisomerization with Use of a Ruthenium Hydride with *N*-Heterocyclic Carbene and Its Application to the Synthesis of Heterocycles. *J. Org. Chem.* **2006**, *71*, 4255–4261.

[2] Xie, S.; Fu, L.; Ding, Y.; Wang, Q.; He, C.; Xu, W.; Wang, Q.; Zhong, Y.; Fan, X.; Yang, M. Electrochemical C–H Mono-/Multi-Bromination Regulation of *N*-Sulfonylanilines on a Cost-Effective Carbon Fiber Electrode and Its Prospective Electroactive Molecule Screening. *J. Org. Chem.* **2024**, *89*, 6759–6769.

[3] Wei, W.-X.; Kuang, Y.; Tomanik, M. Ligand-Controlled Alkylation–Heck–C($sp^3$)–H Annulation Cascade for a Divergent Synthesis of Cyclobutane- and Cyclopropane-Containing Heterocycles. *J. Am. Chem. Soc*. **2025**, 147, 37347–37356.

[4] Chen, D.; Chen, Y.; Ma, Z.; Zou, L.; Li, J.; Liu, Y. One-Pot Synthesis of Indole-3-acetic Acid Derivatives through the Cascade Tsuji–Trost Reaction and Heck Coupling. *J. Org. Chem*. **2018**, *83*, 6805-6814.

[5] Vaith, J.; Rodina, D.; Spaulding, G. C.; Paradine, S. M. Pd-Catalyzed Heteroannulation Using *N*-Arylureas as a Sterically Undemanding Ligand Platform. *J. Am. Chem. Soc.* **2022**, *144*, 6667-6673.

[6] Marcyk, P. T.; Jefferies, L. R.; AbuSalim, D. I.; Pink, M.; Baik, M.-H.; Cook, S. P. Stereoinversion of Unactivated Alcohols by Tethered Sulfonamides. *Angew. Chem. Int. Ed.* **2019**, *58*, 1727-1731.

[7] Majumdar, K. C.; Mondal, S.; De, N. Synthesis of Polycyclic Sultams by Palladium-Catalyzed Intramolecular Cyclization. *Synthesis*, **2009**, *18*, 3127-3135.

[8] Chandra, T.; Zebrowski, J. P. A Safety Guidance Document for Lithium Aluminum Hydride (LAH) Reduction: A Resource for Developing Specific SOPs on LAH Manipulations. *ACS Chem. Health Saf.* **2024**, *31*, 162–171.

[9] Li, Z.; Park, H. S.; Qiao, J. X.; Yeung, K.-S.; Yu, J.-Q. Ligand-Enabled C–H Hydroxylation with Aqueous $H_2O_2$ at Room Temperature. *J. Am. Chem. Soc*. **2022**, *144*, 18109–18116.

[10] Berger, K. J.; Driscoll, J. L.; Yuan, M.; Dherange, B. D.; Gutierrez, O.; Levin, M. D. Direct Deamination of Primary Amines via Isodiazene Intermediates. *J. Am. Chem. Soc.* **2021**, *143*, 17366–17373.

[11] Ruiz-Castillo, P.; Buchwald, S. L. Applications of Palladium-Catalyzed C–N Cross-Coupling Reactions. *Chem. Rev.* **2016**, *116*, 12564–12649.

[12] Ye, Z.; Adhikari, S.; Xia, Y.; Dai, M. Expedient Syntheses of *N*-Heterocycles via Intermolecular Amphoteric Diamination of Allenes. *Nat Commun.* **2018**, *9*, 721.

[13] Yoon, J.-H.; Park, Y.-J.; Ahn, H.-C.; Lee, Y.-S.; Han, J.-H. CN Patent 120718051 A, 2025.

[14] Sinou, D.; Maillard, D.; Pozzi, G. Synthesis of a Family of Triarylphosphanes with Fluorous Phase Affinity. *Eur. J. Org. Chem.* **2002**, *2002*, 269-275.

**Catalogue of $^{1}$H NMR and $^{13}$C NMR Spectra.**

<u>NMR Spectra of Substrates:</u>

$^{1}$H NMR (400 MHz, $CDCl_3$)

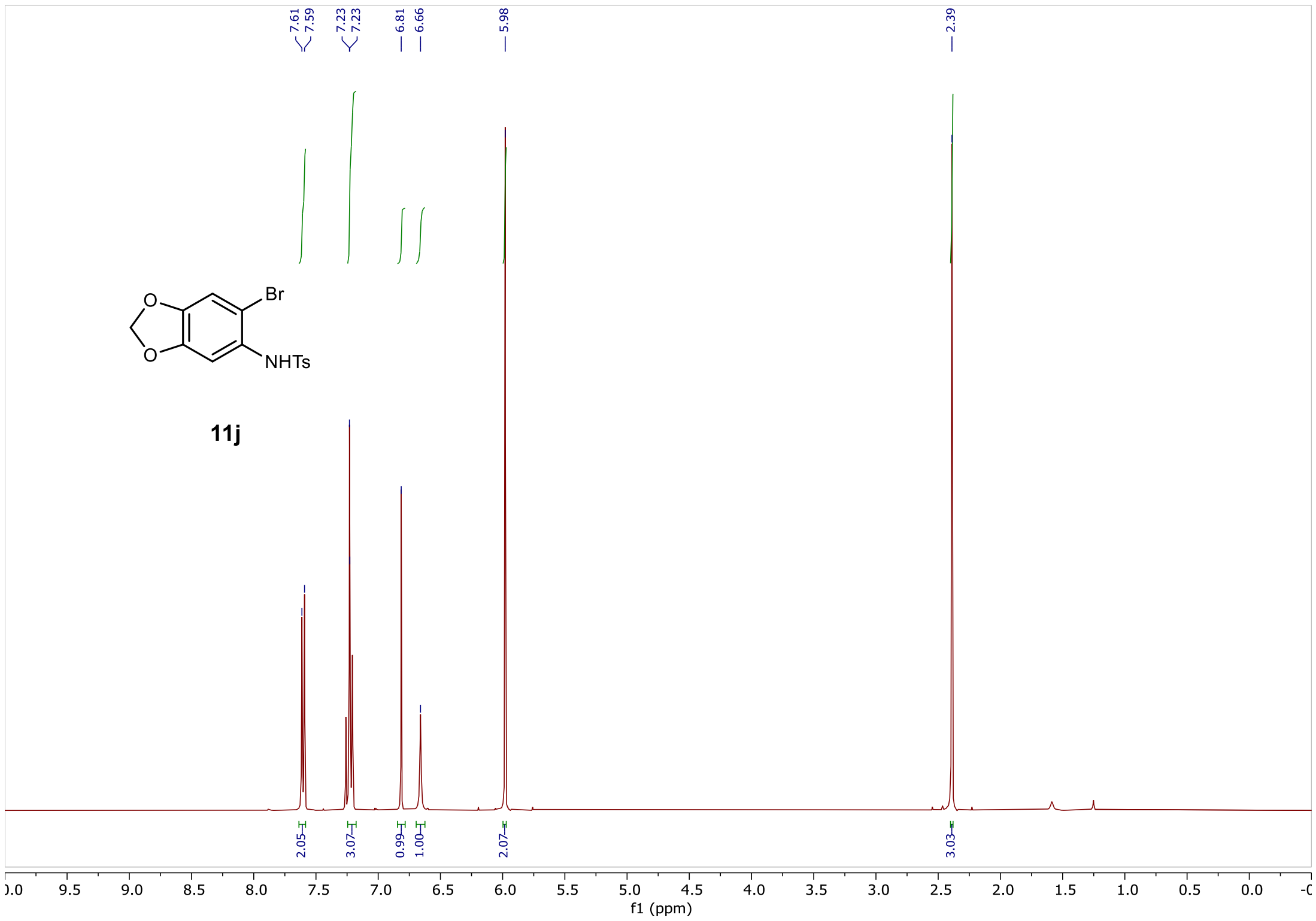


$^{13}$C NMR (101 MHz, $CDCl_3$)

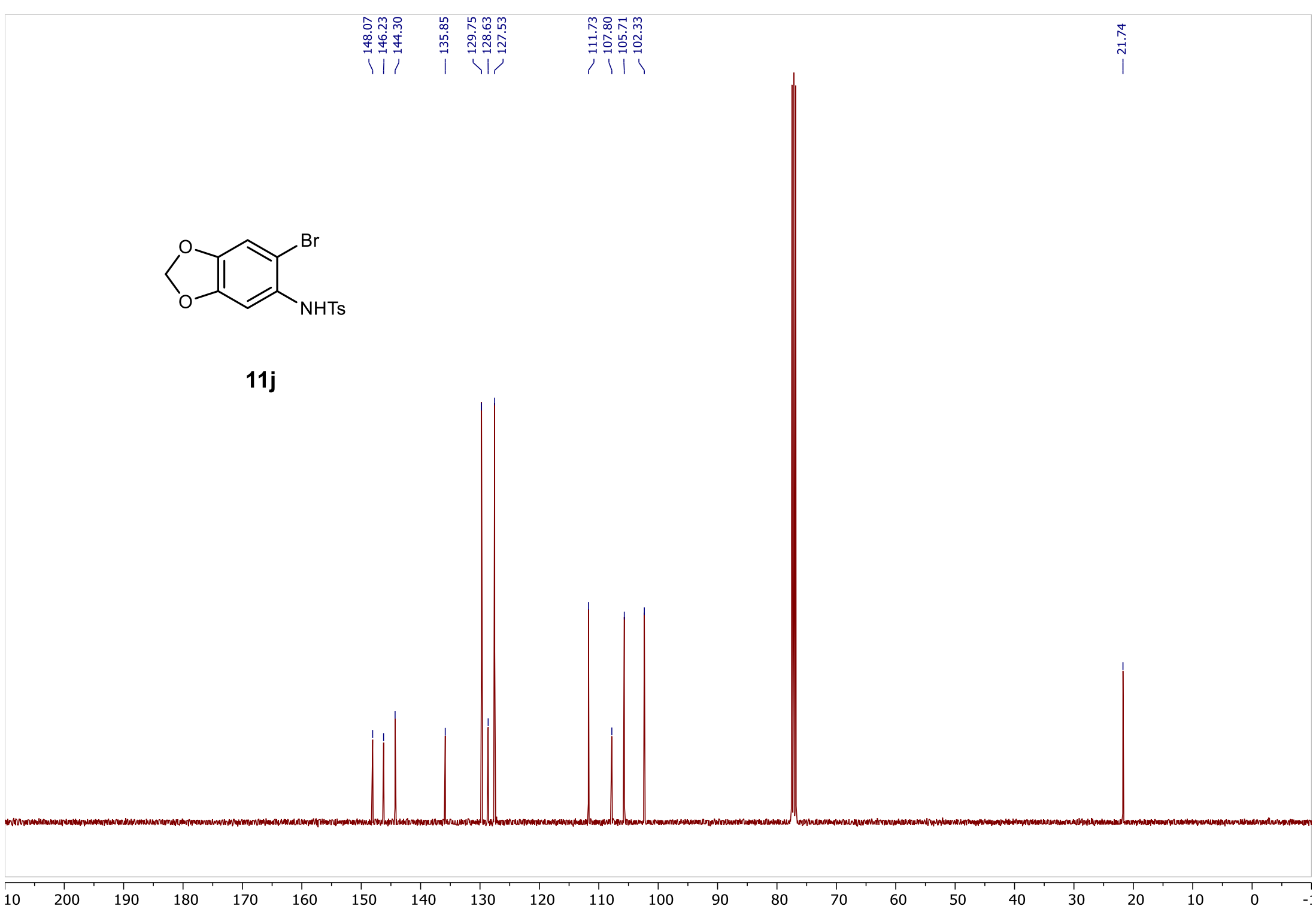

$^1$H NMR (400 MHz, $CDCl_3$)

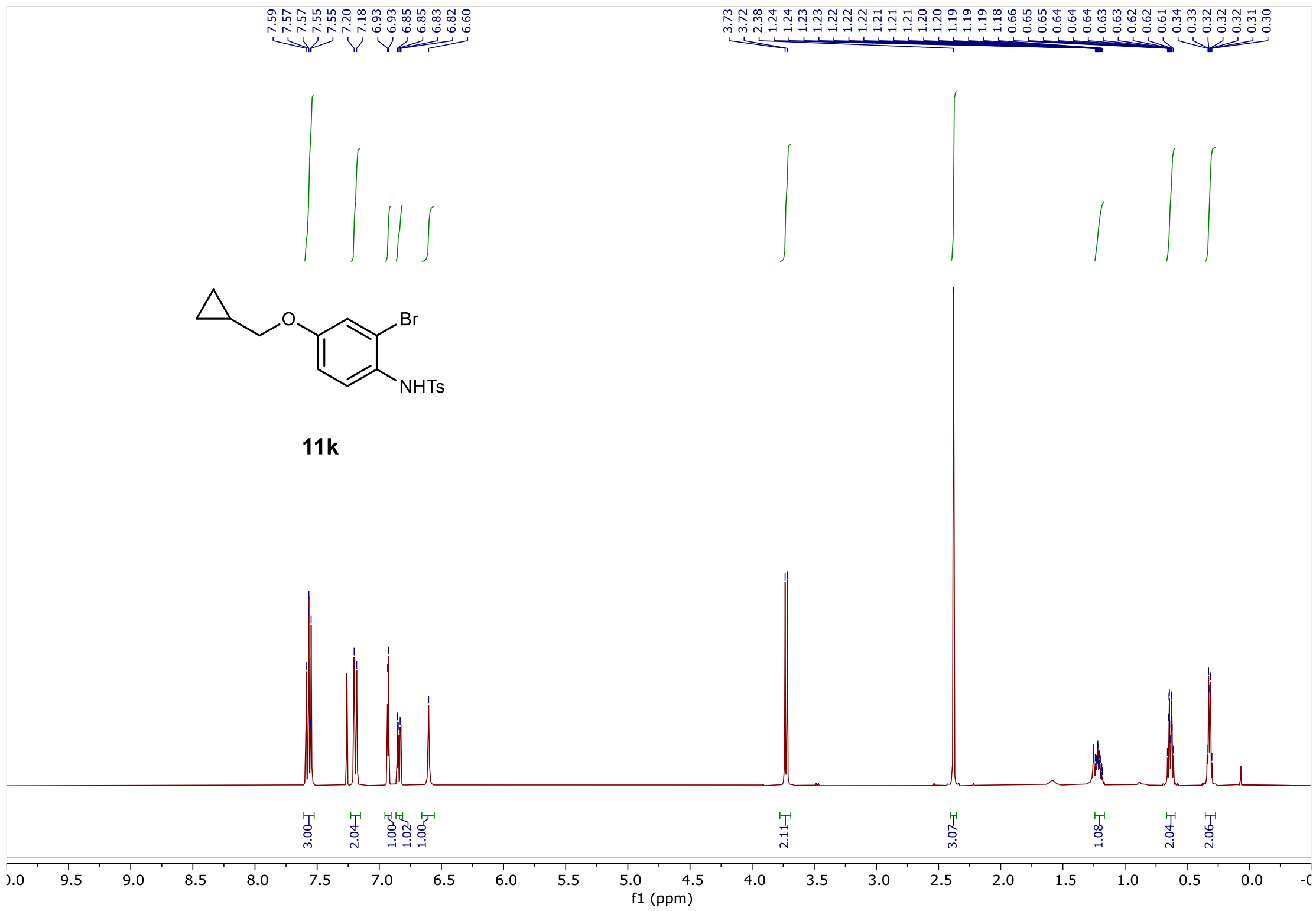


$^{13}$C NMR (101 MHz, $CDCl_3$)

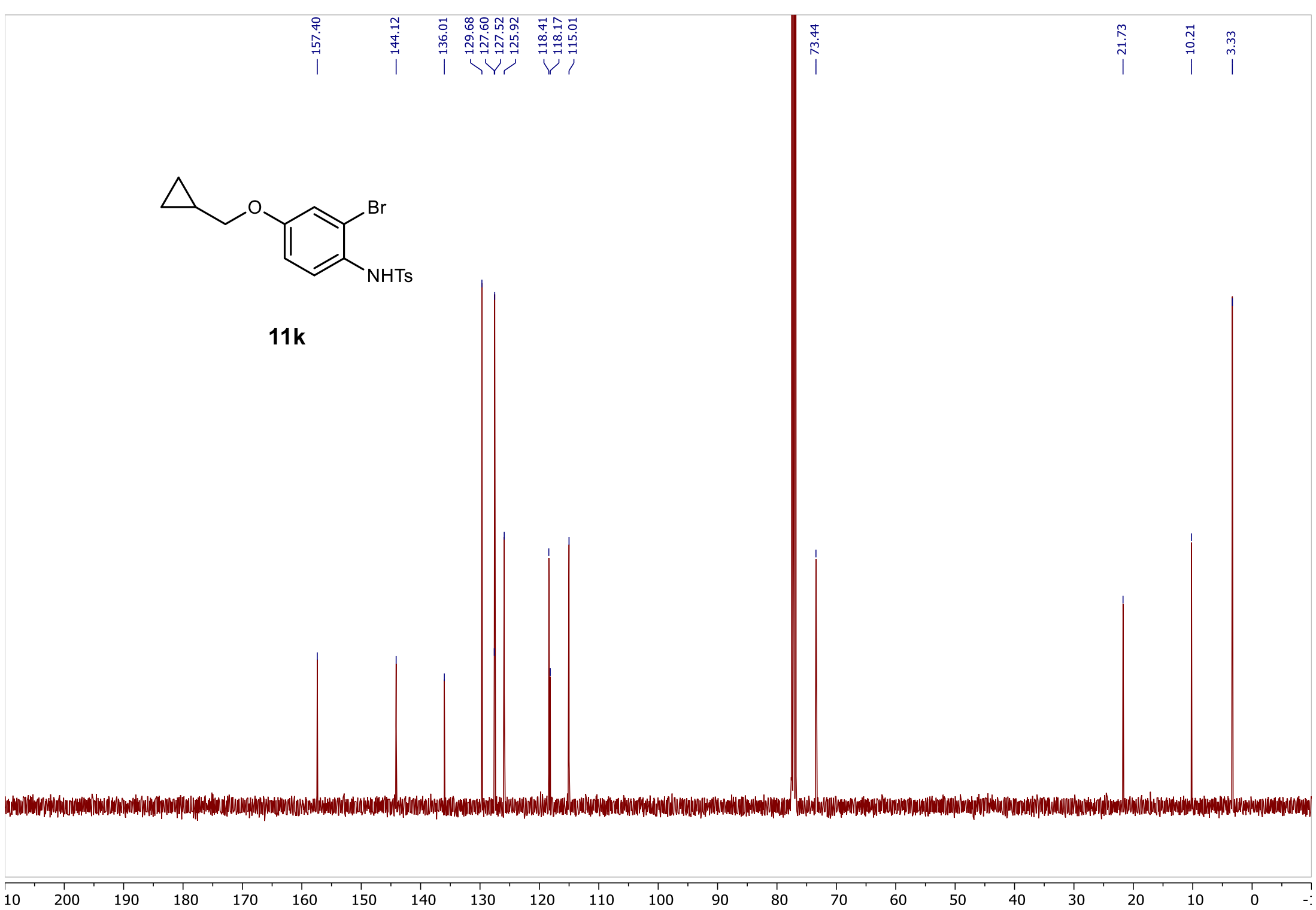

$^{1}H$ NMR (400 MHz, $CDCl_3$)

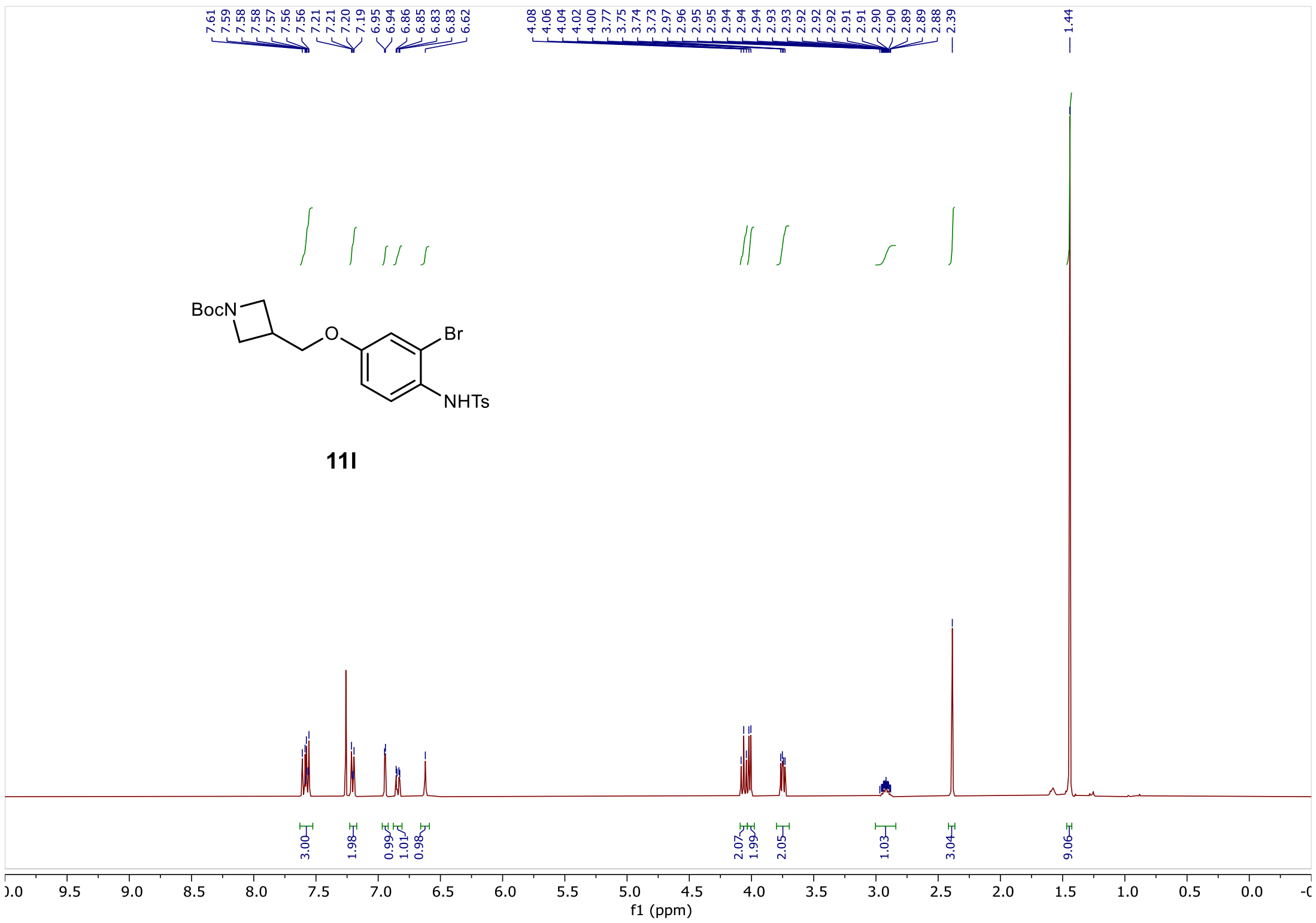

$^{13}C$ NMR (101 MHz, $CDCl_3$)

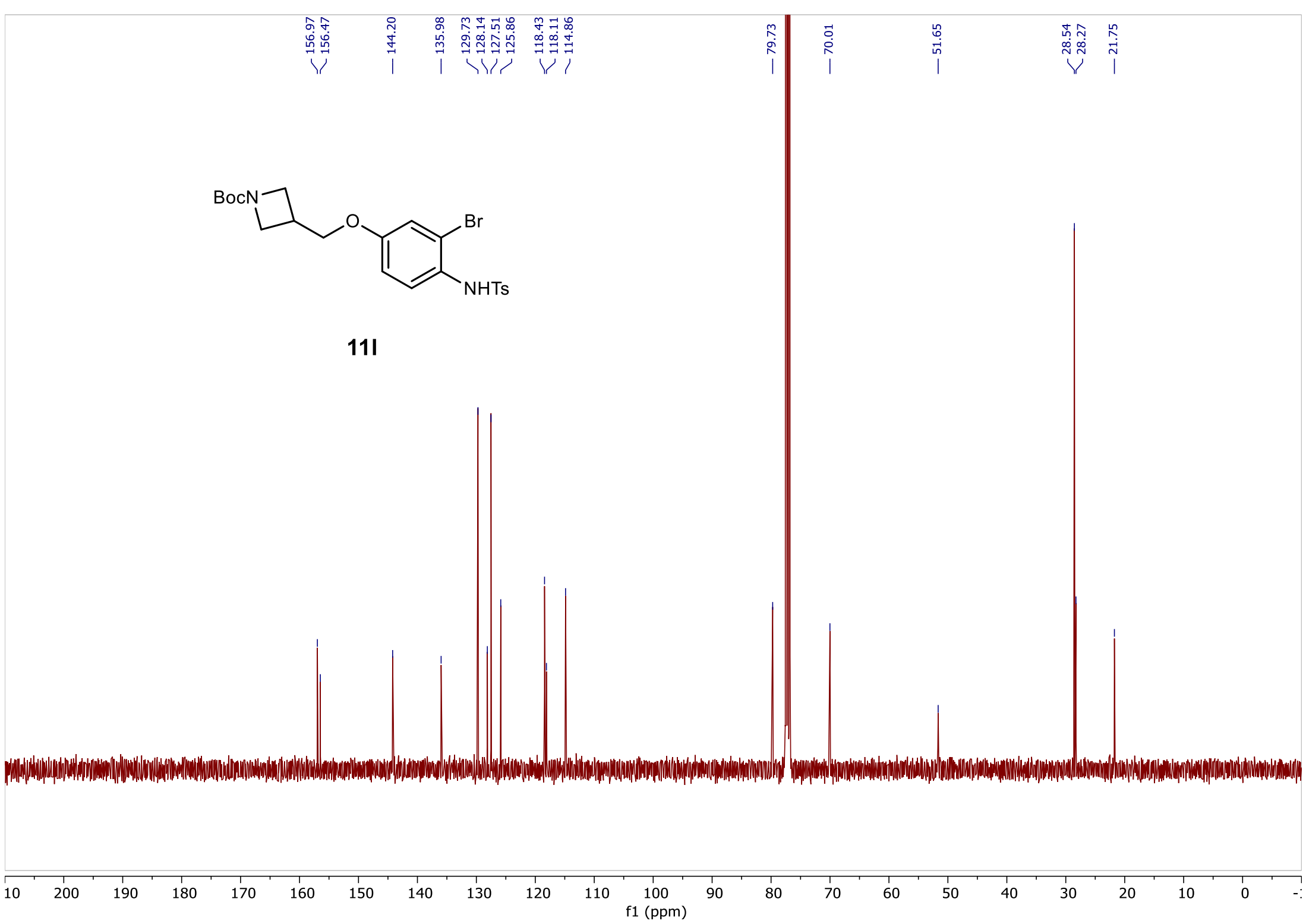

$^{1}H$ NMR (400 MHz, $CDCl_3$)

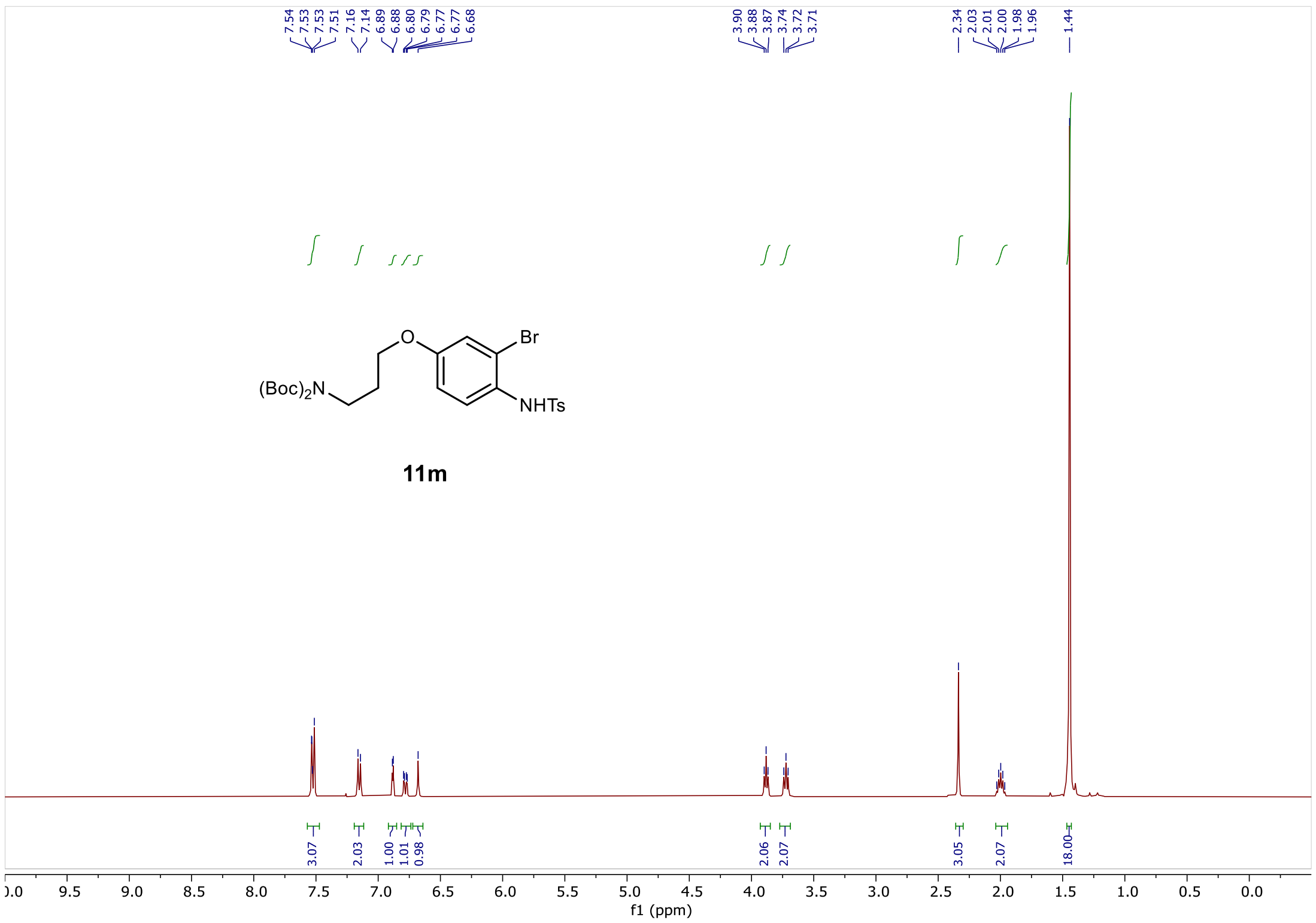


$^{13}C$ NMR (101 MHz, $CDCl_3$)

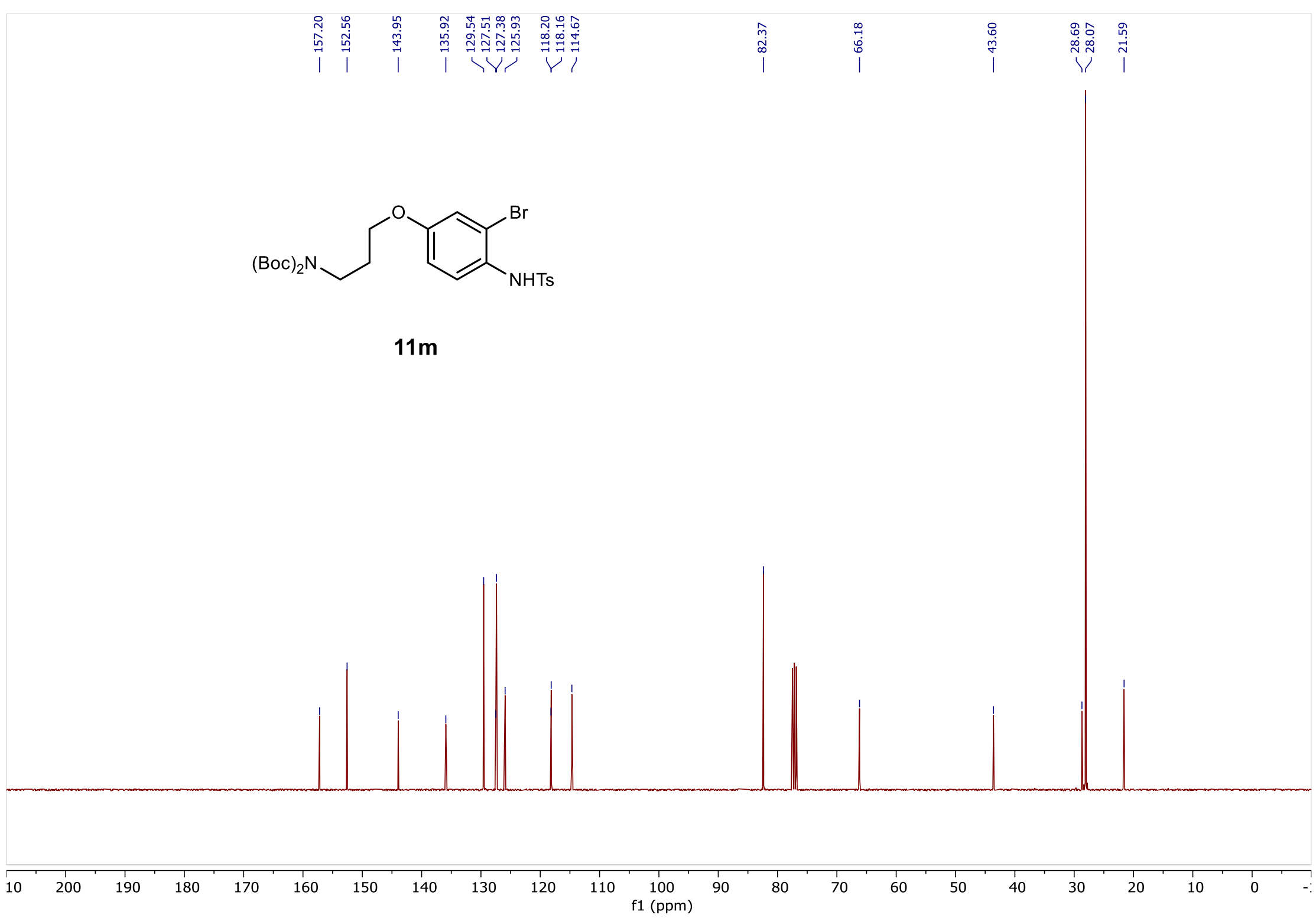

$^{1}H$ NMR (400 MHz, $CDCl_3$)

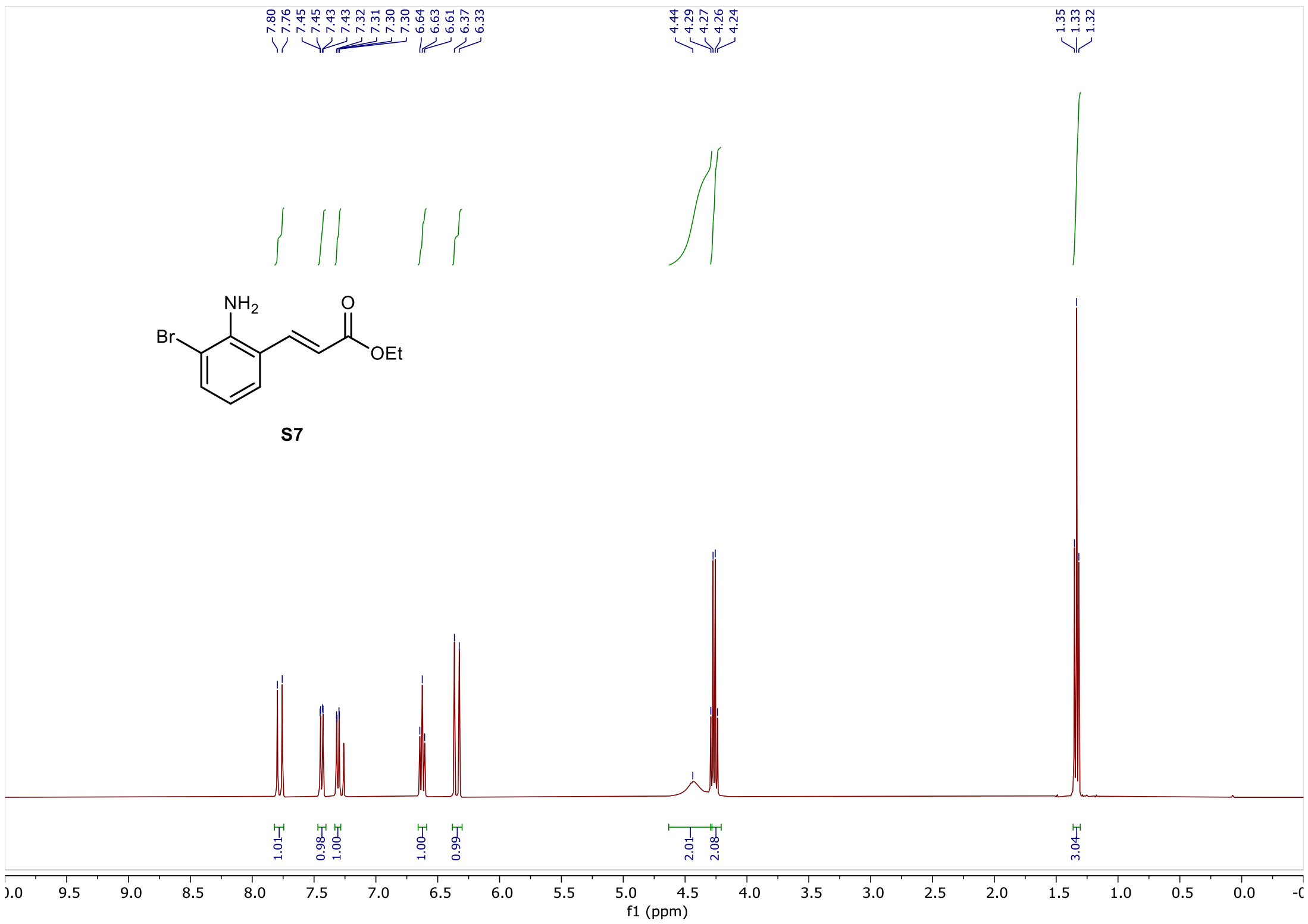


$^{13}C$ NMR (101 MHz, $CDCl_3$)

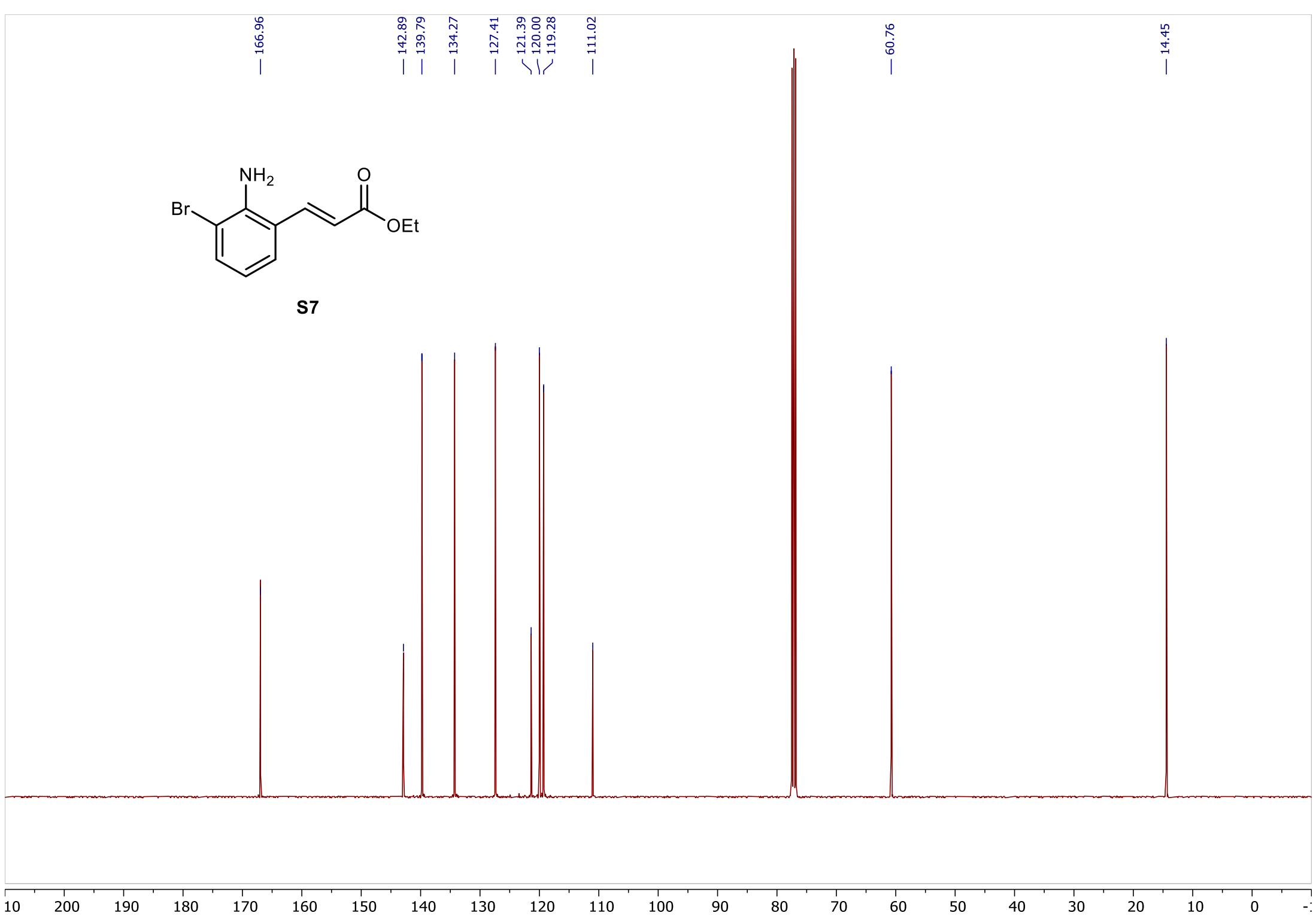

$^{1}H$ NMR (400 MHz, $CDCl_3$)

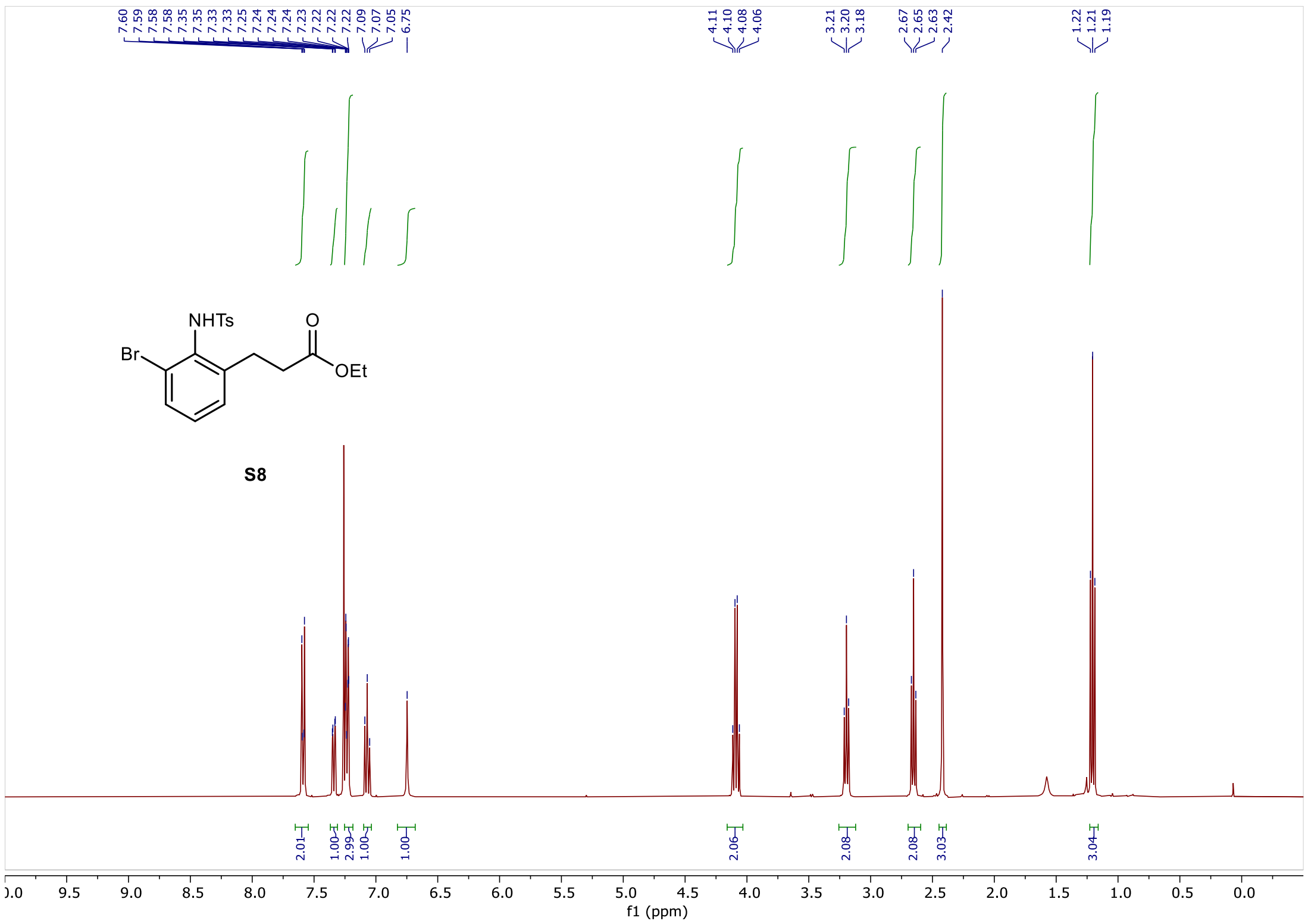

$^{13}C$ NMR (101 MHz, $CDCl_3$)

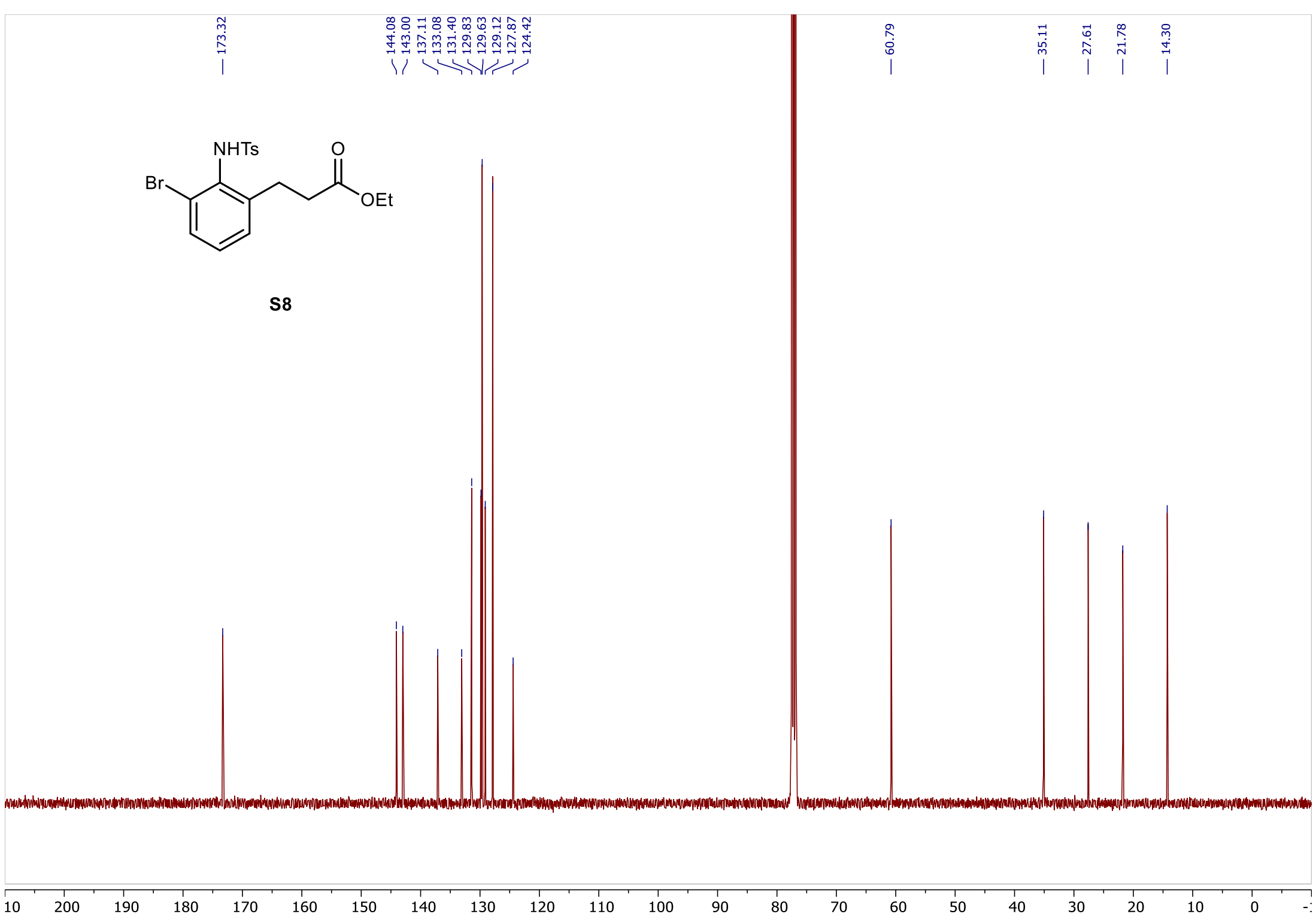

$^{1}H$ NMR (400 MHz, $CDCl_3$)

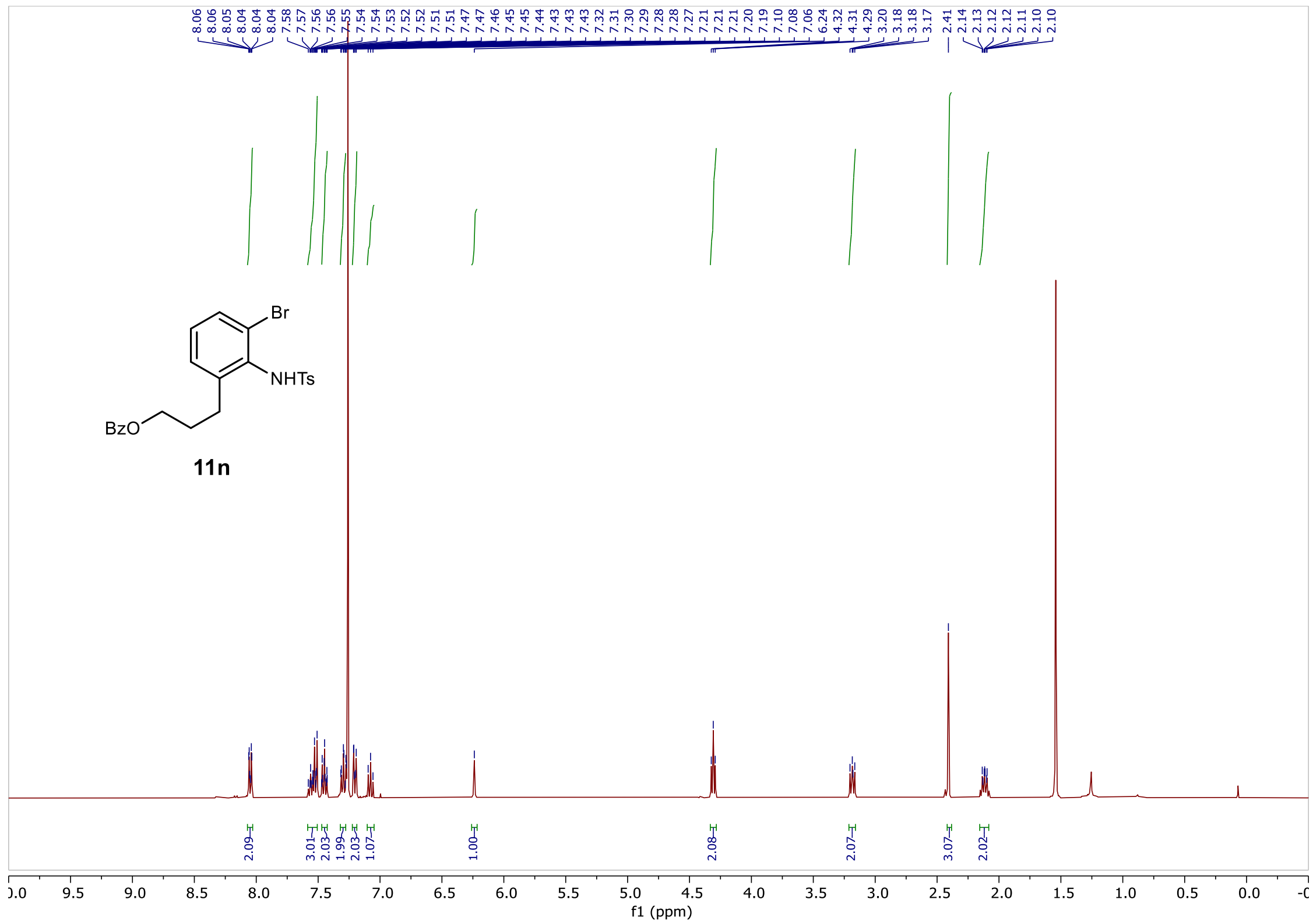

$^{13}C$ NMR (101 MHz, $CDCl_3$)

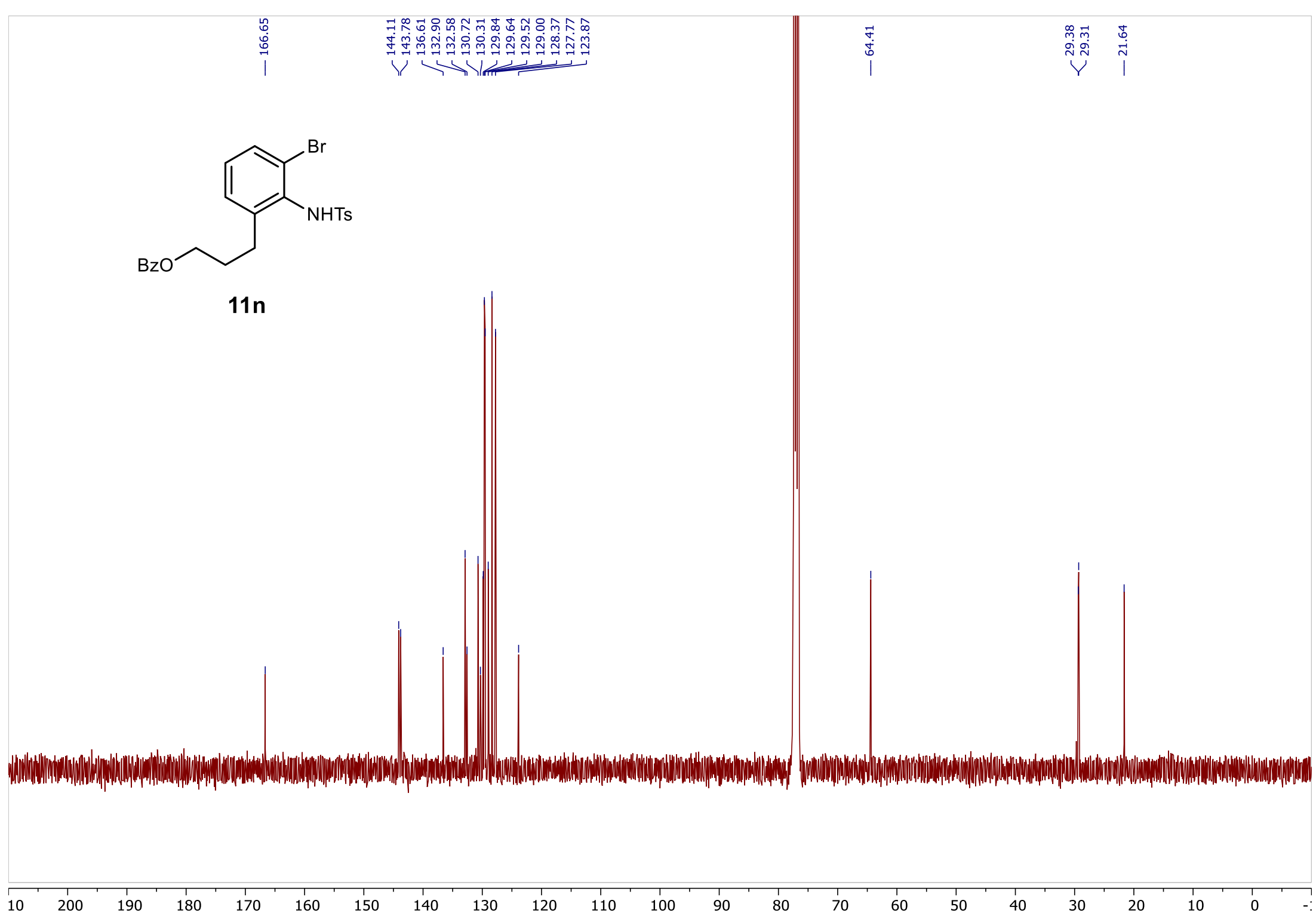

$^1$H NMR (400 MHz, $CDCl_3$)

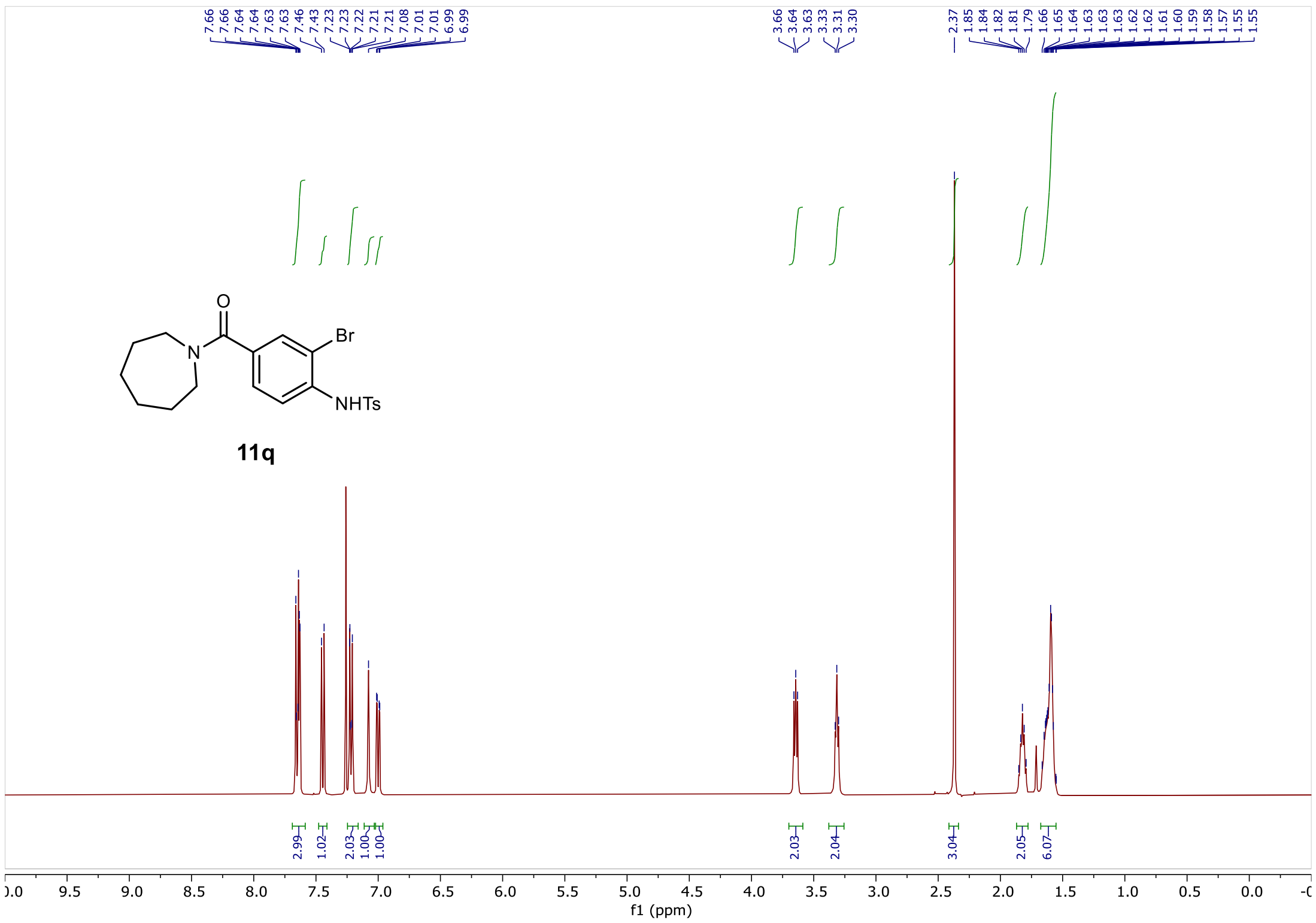


$^{13}$C NMR (101 MHz, $CDCl_3$)

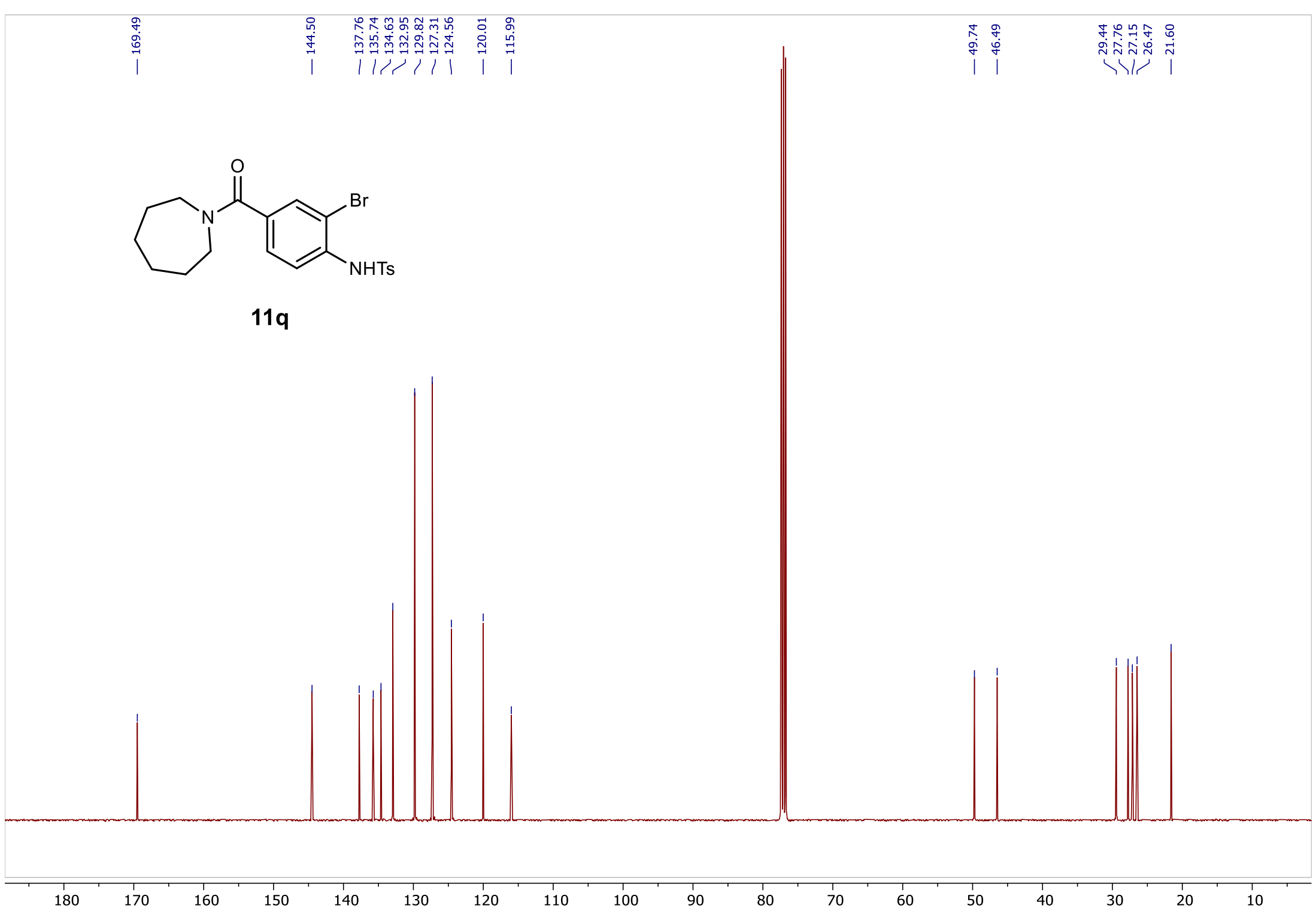

$^{1}$H NMR (400 MHz, $CDCl_3$)

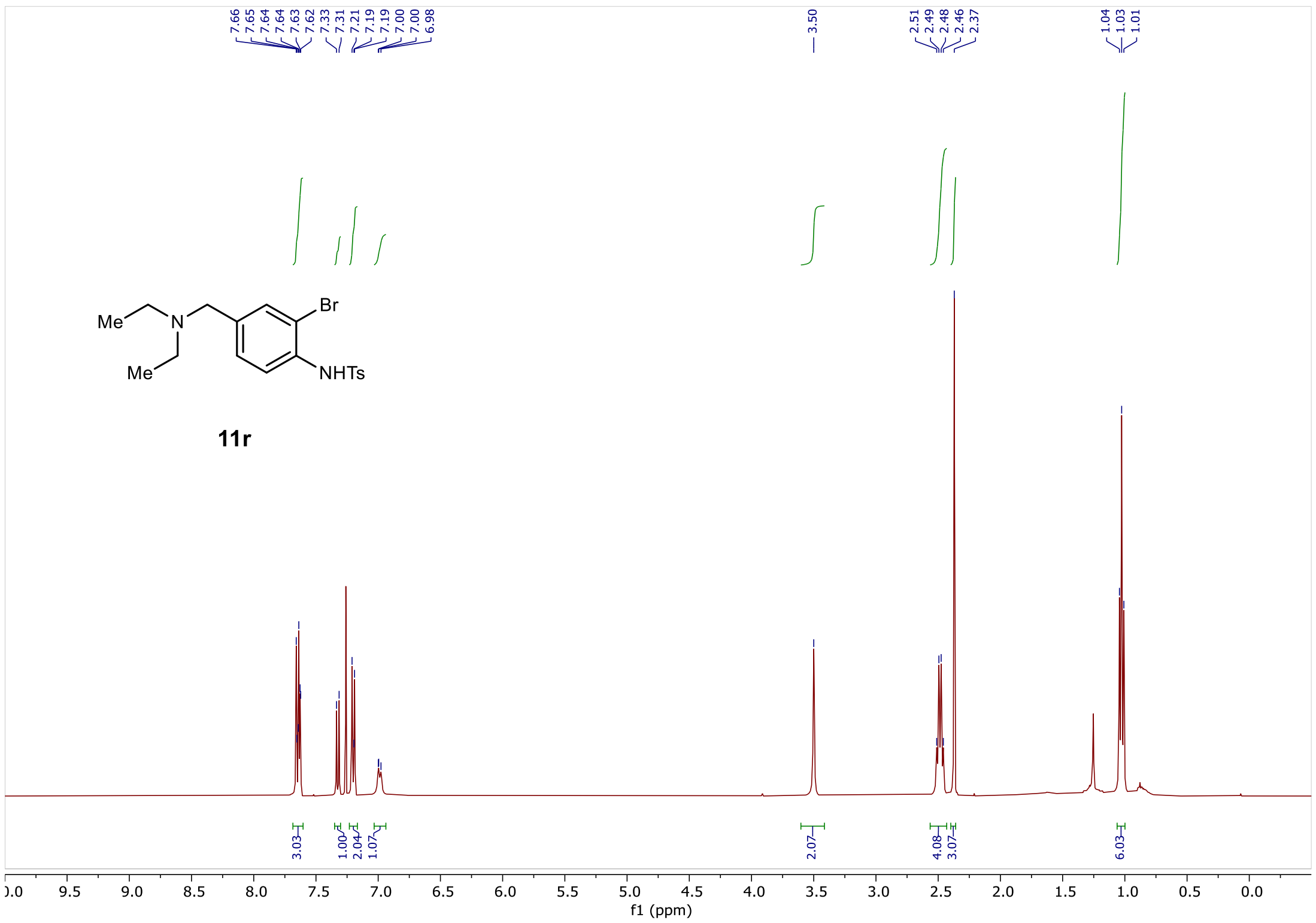

$^{13}$C NMR (101 MHz, $CDCl_3$)

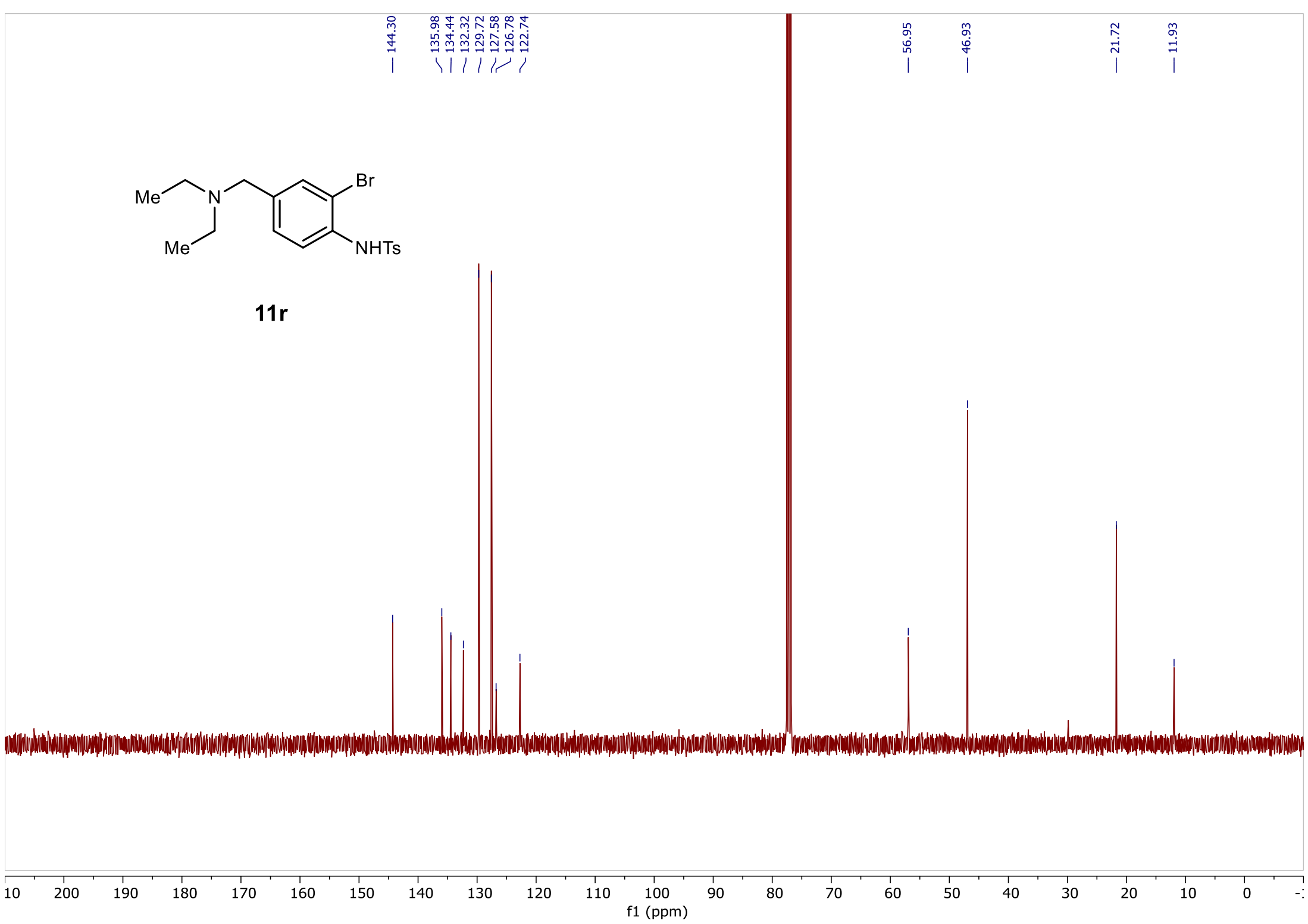

$^{1}$H NMR (400 MHz, $CDCl_3$)

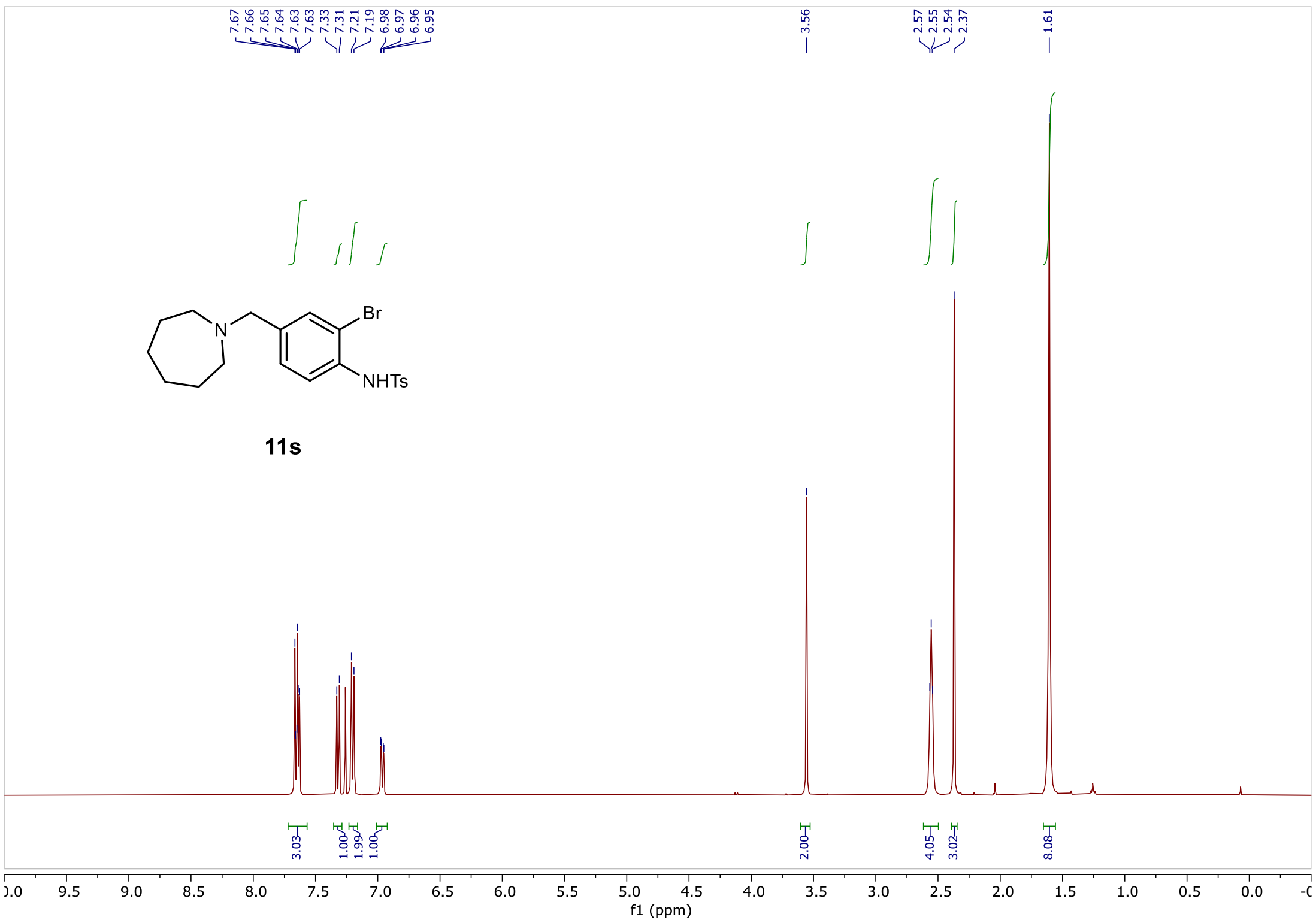


$^{13}$C NMR (101 MHz, $CDCl_3$)

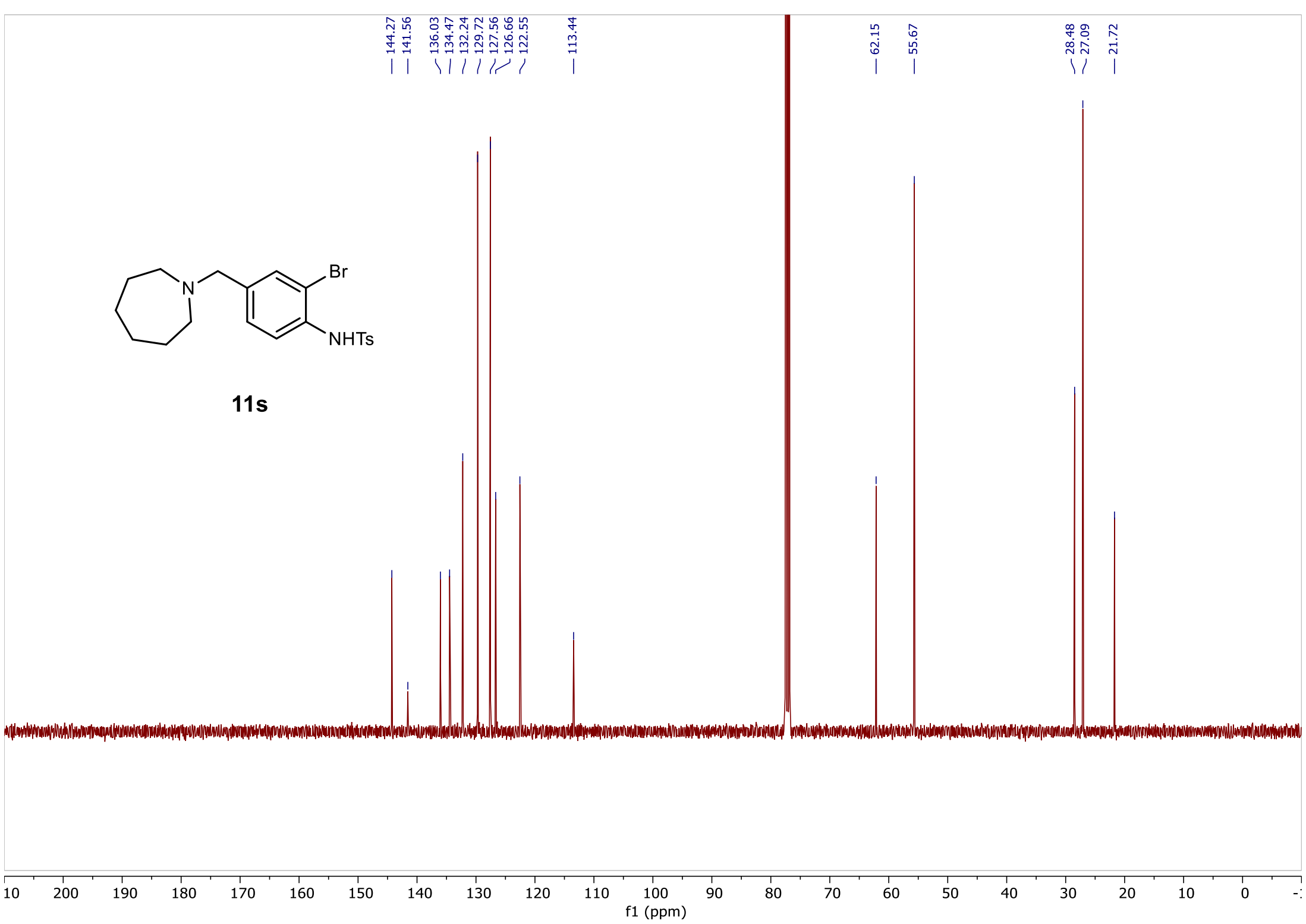

$^{1}$H NMR (400 MHz, $CDCl_3$)

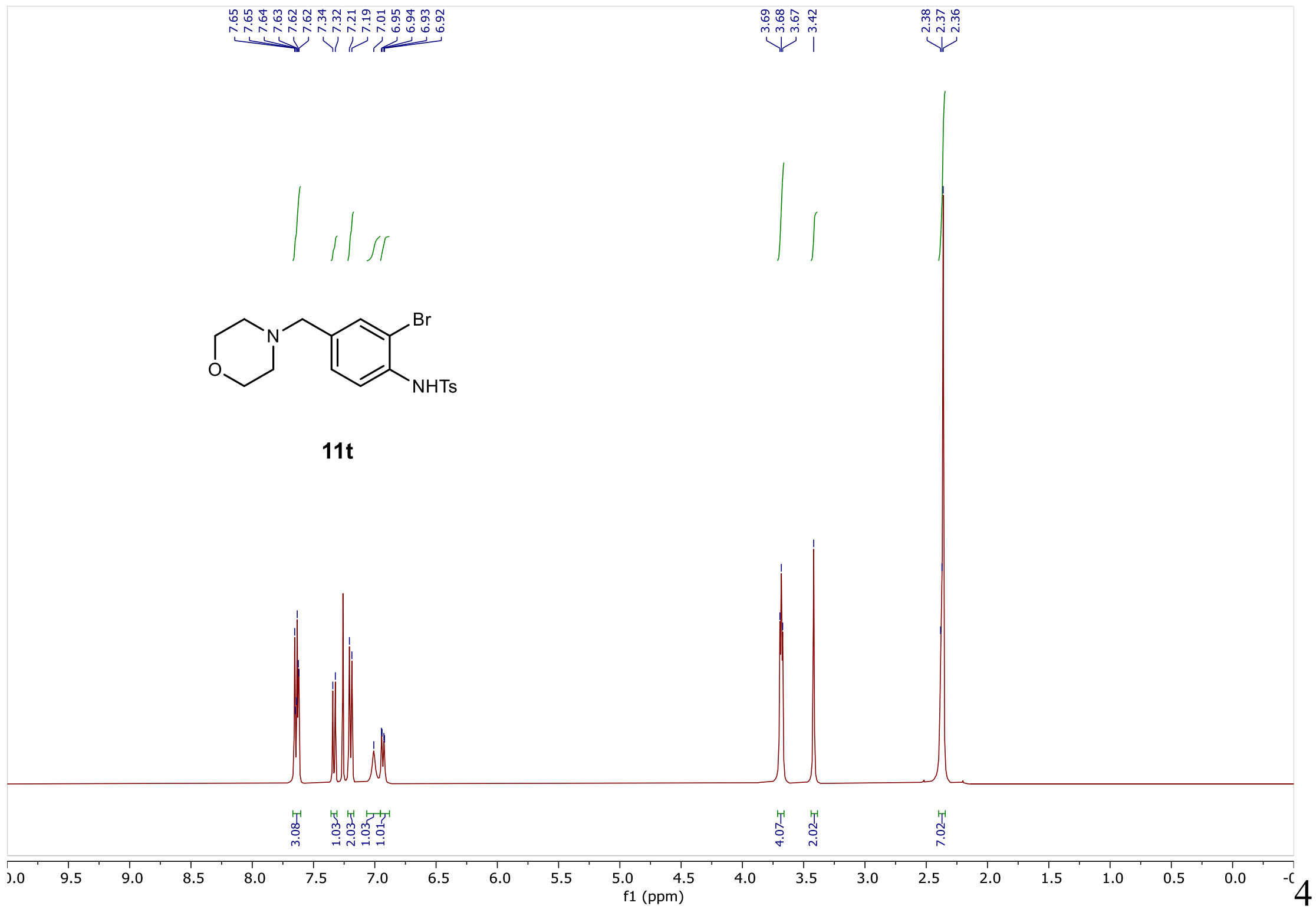


$^{13}$C NMR (101 MHz, $CDCl_3$)

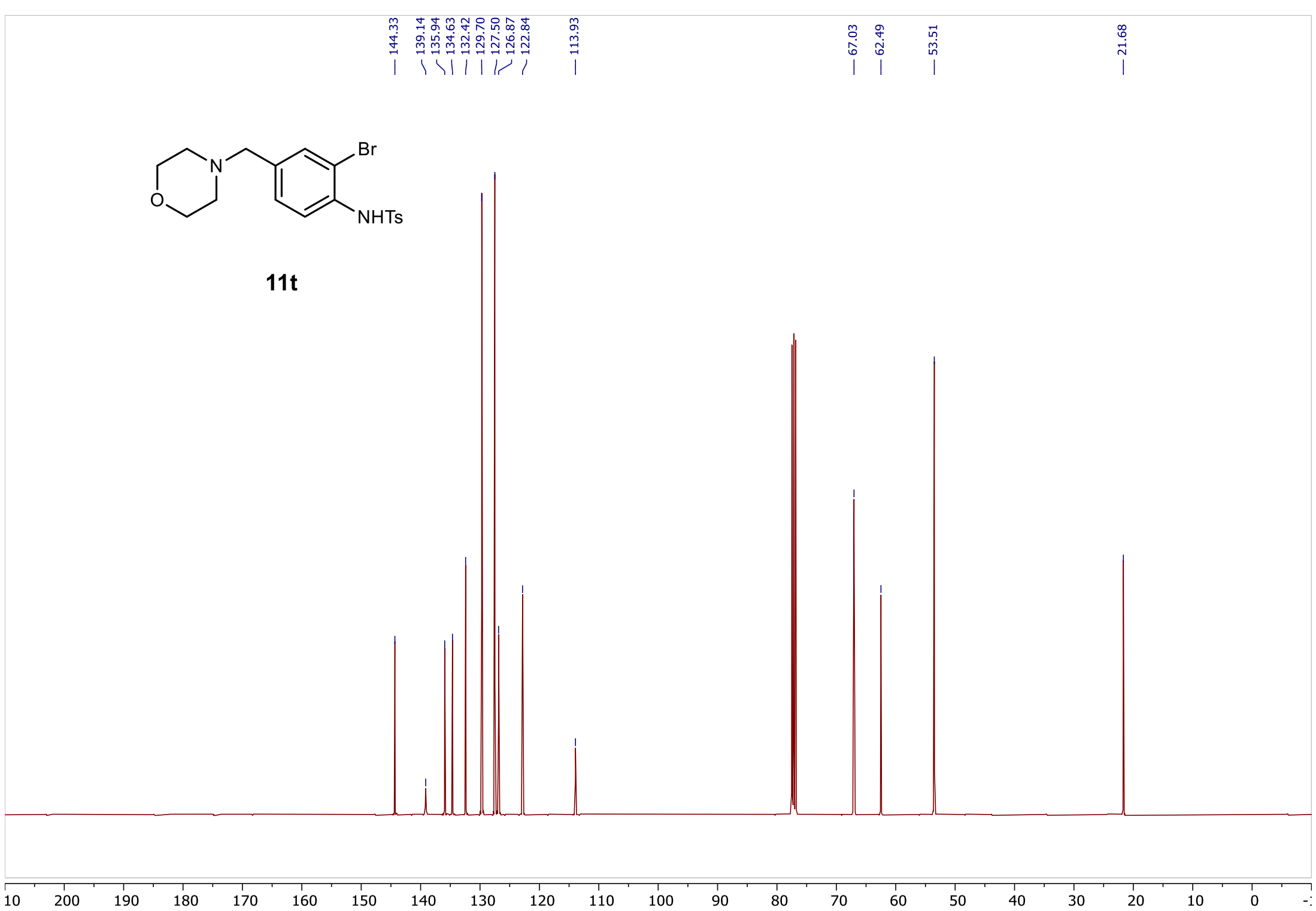

$^{1}H$ NMR (400 MHz, $CDCl_3$)

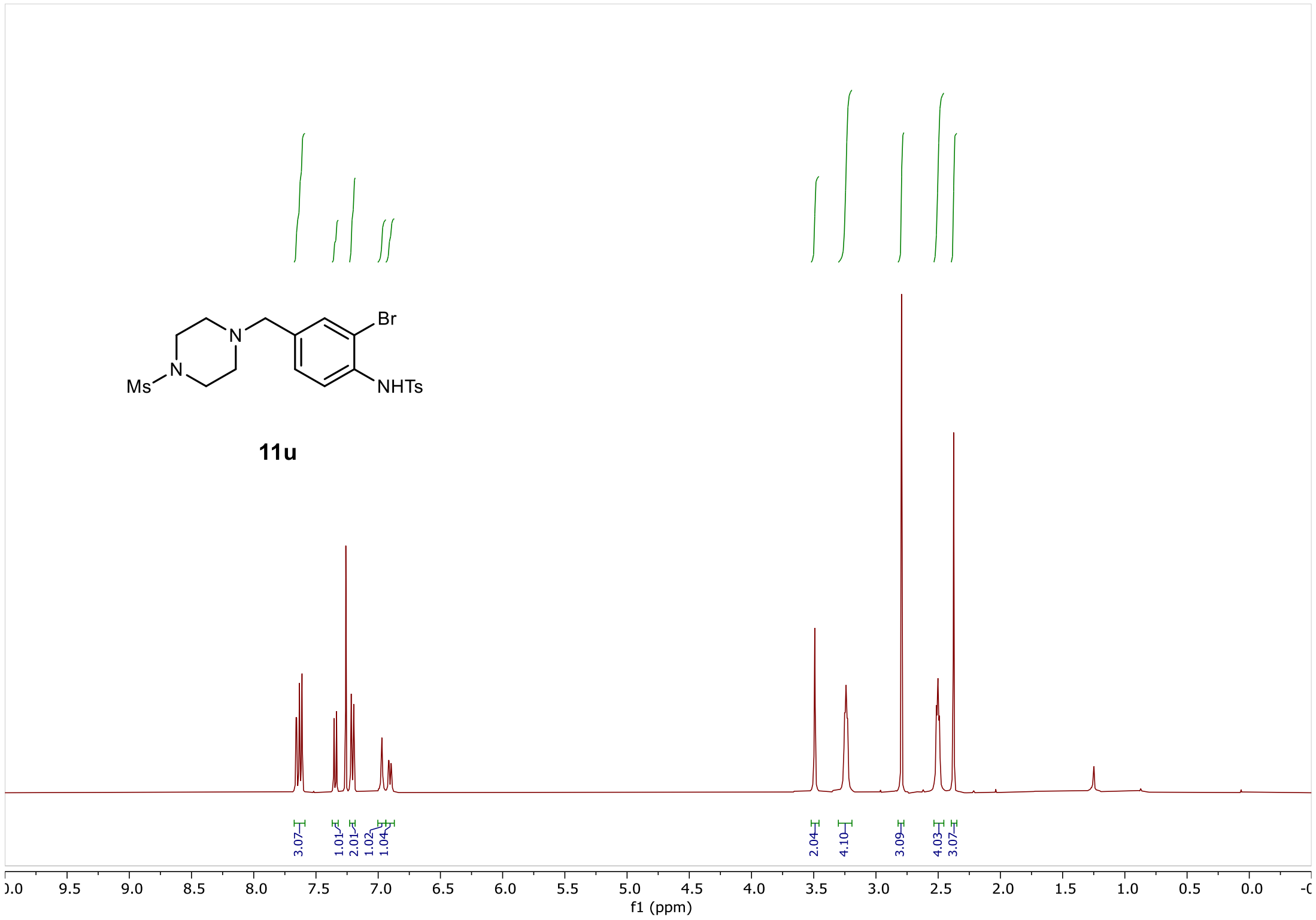


$^{13}C$ NMR (101 MHz, $CDCl_3$)

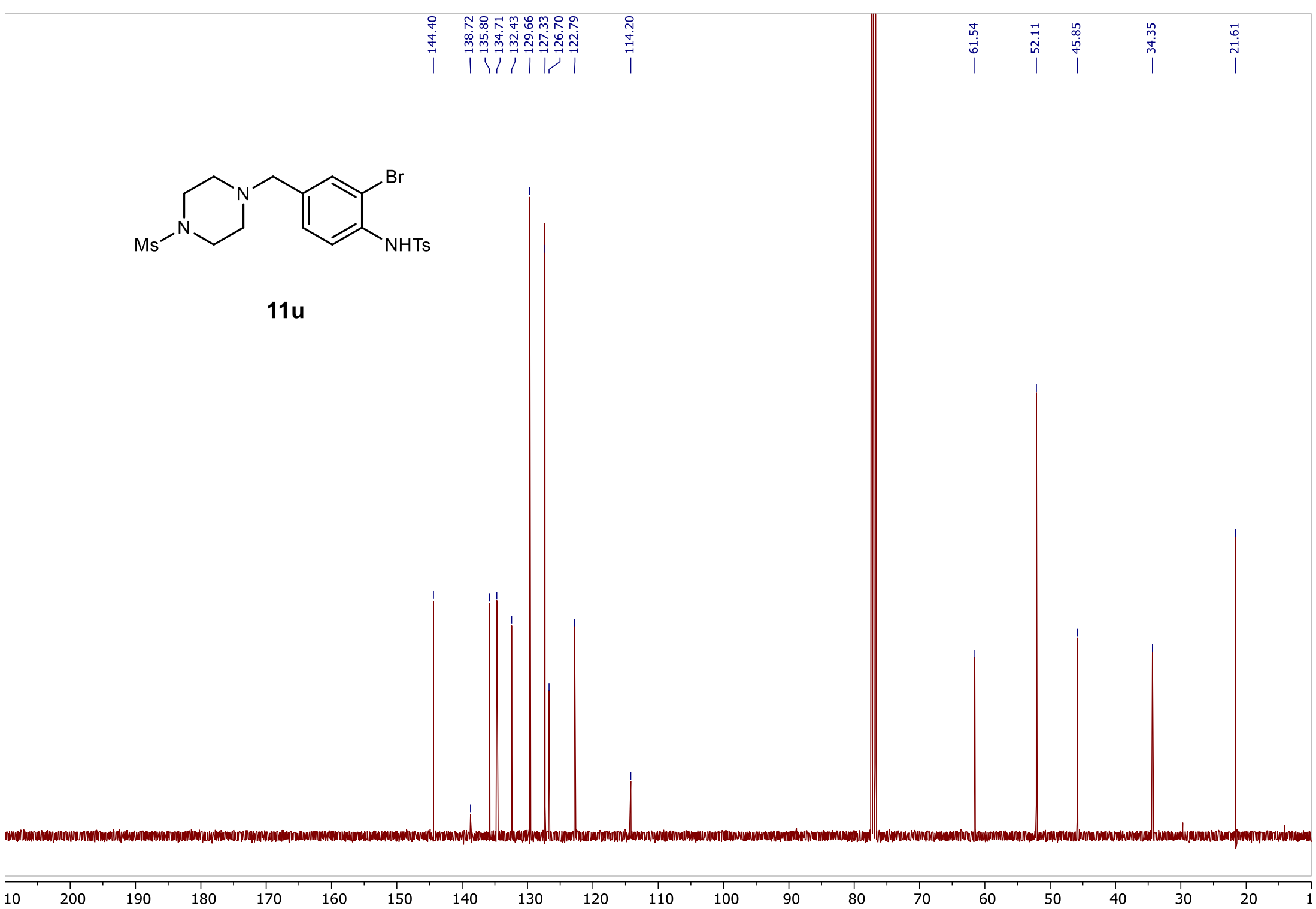

$^{1}H$ NMR (400 MHz, $CDCl_3$)

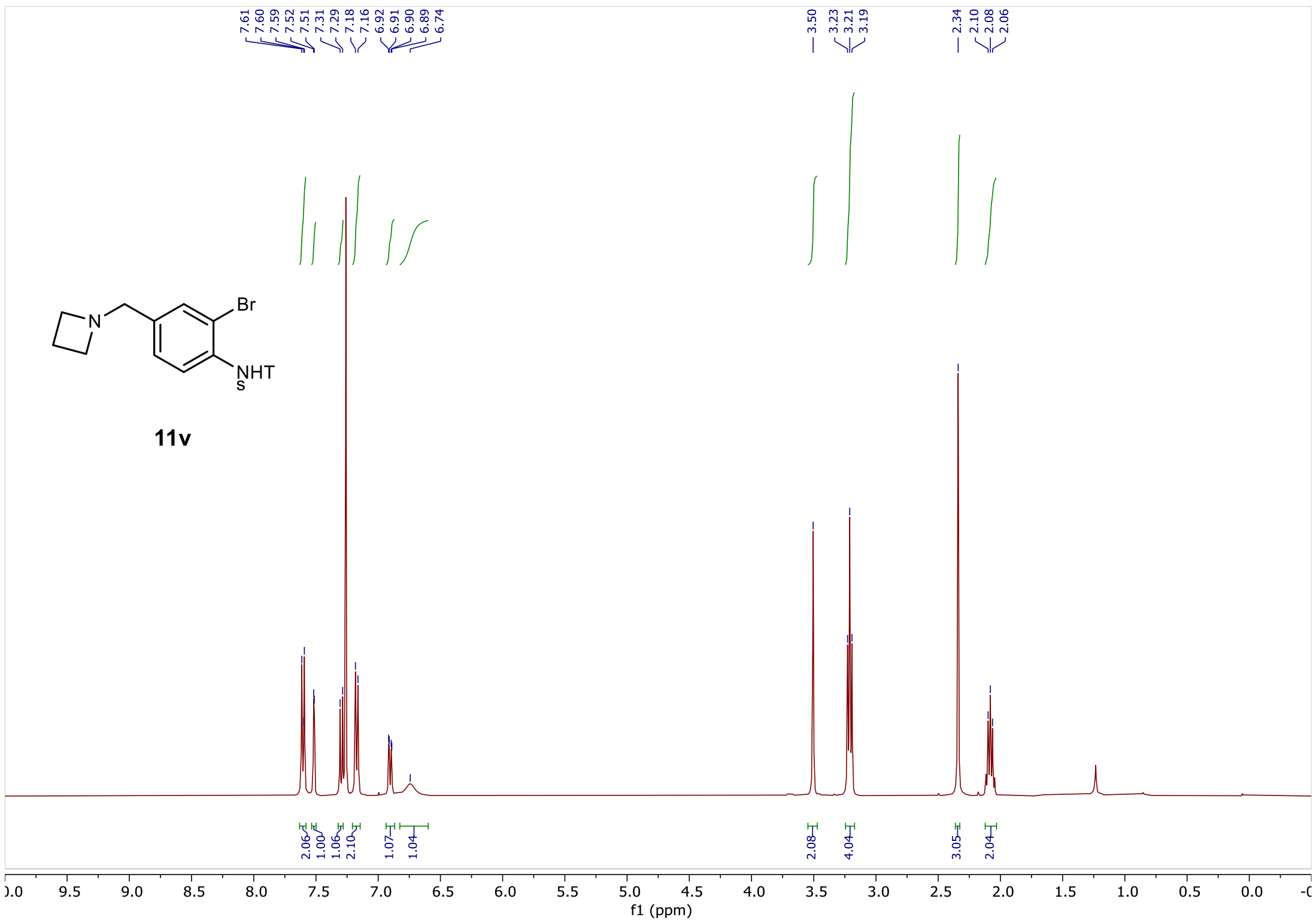

$^{13}C$ NMR (101 MHz, $CDCl_3$)

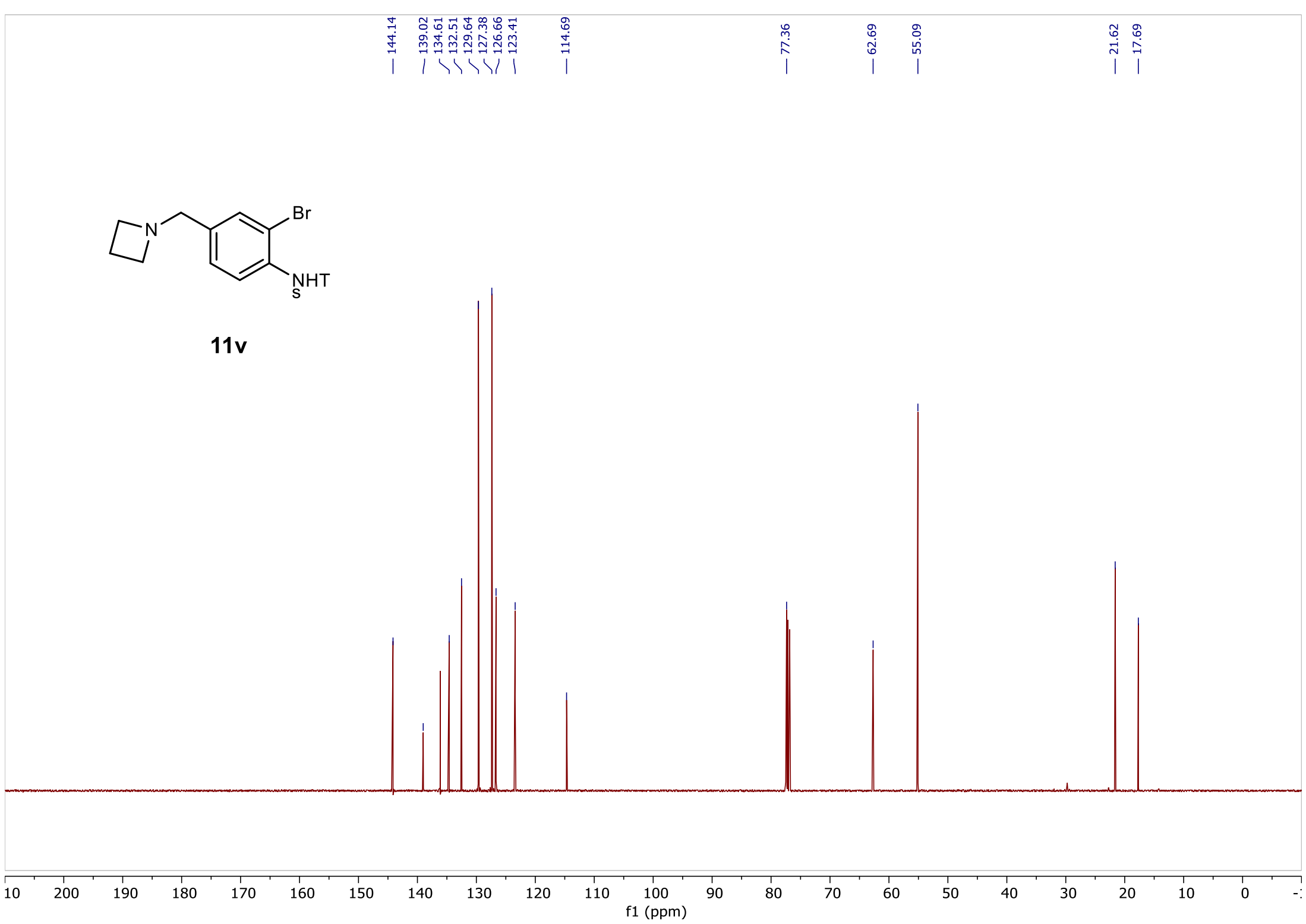

$^{1}H$ NMR (400 MHz, $CDCl_3$)

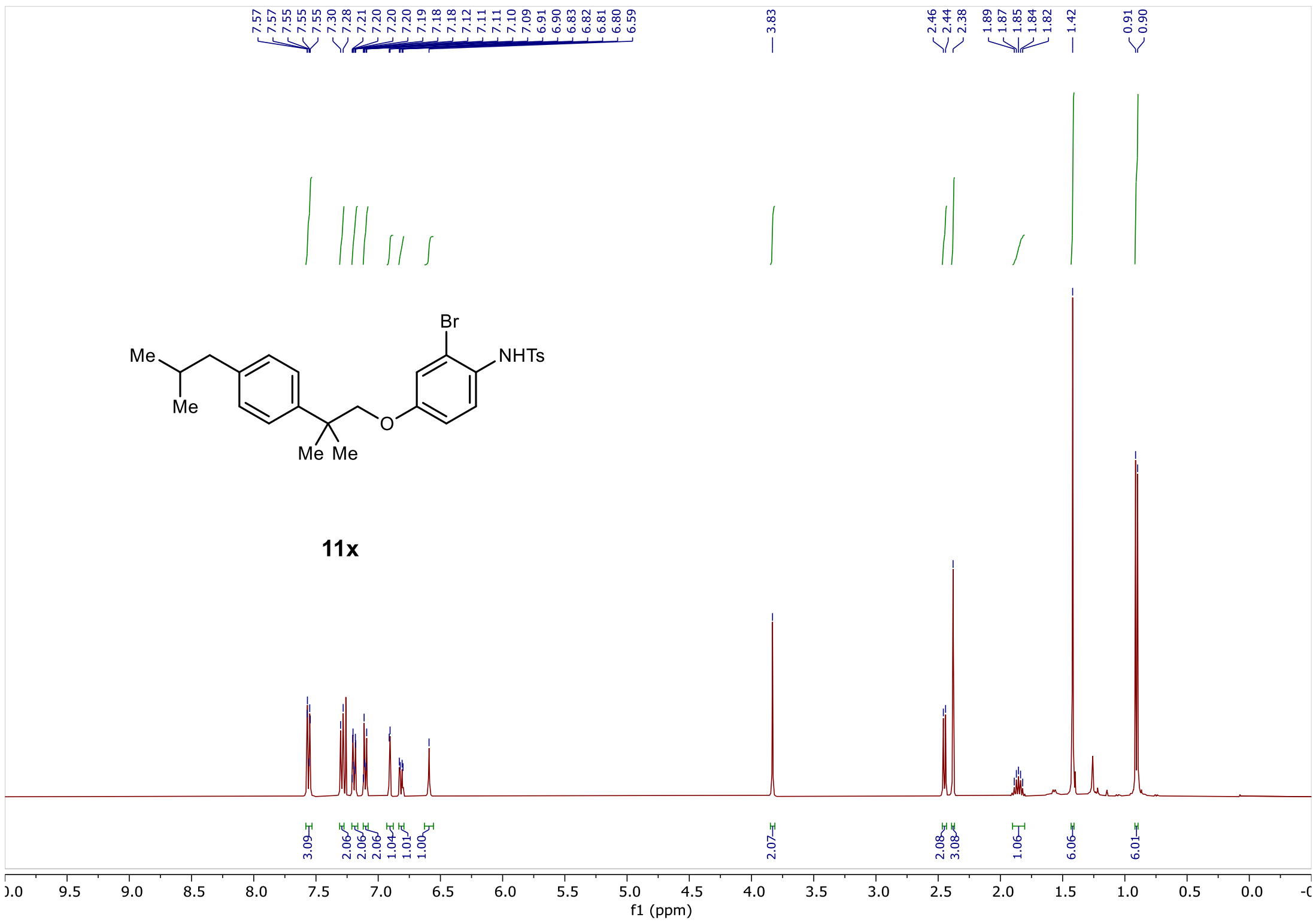


$^{13}C$ NMR (101 MHz, $CDCl_3$)

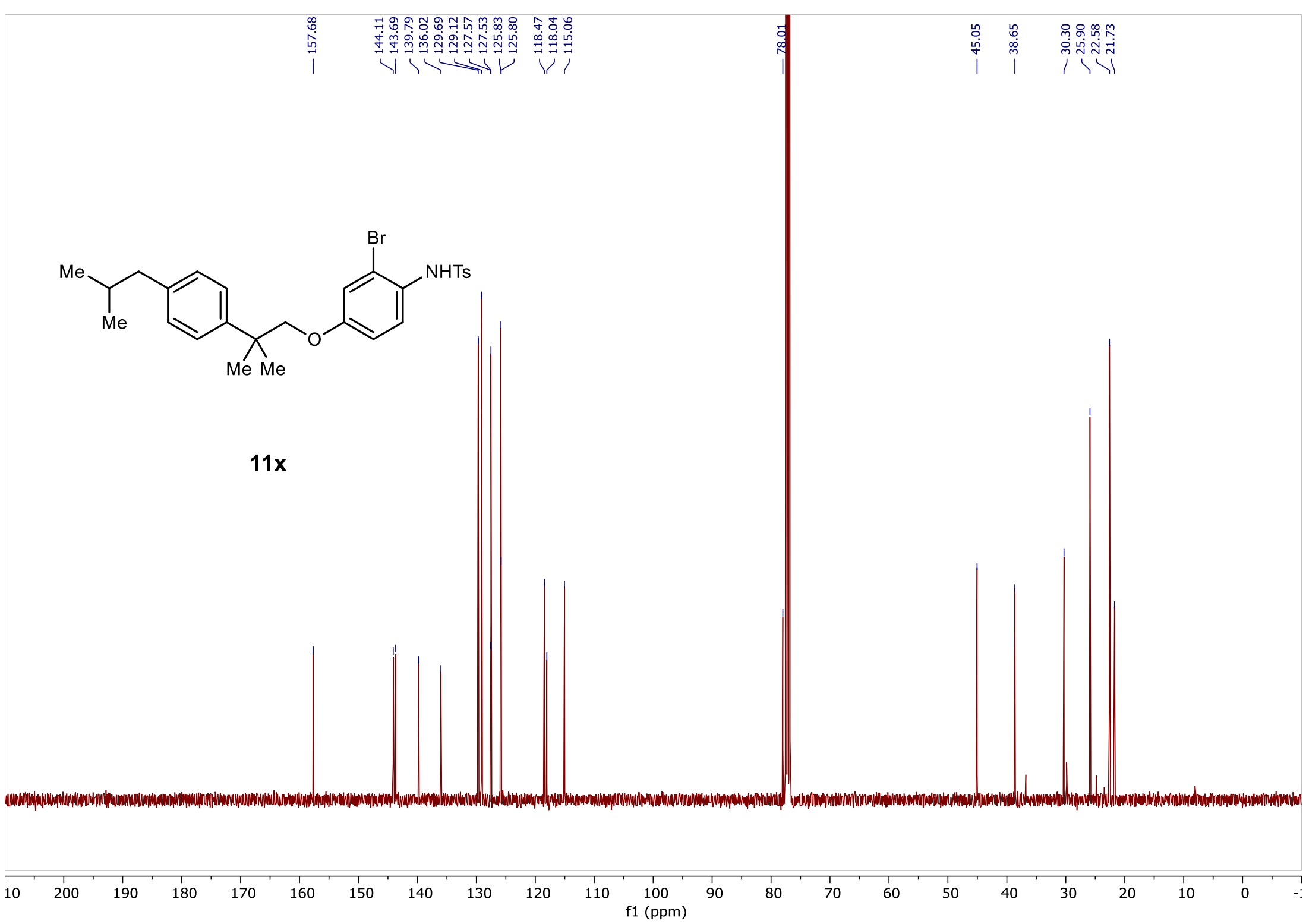

$^{1}H$ NMR (400 MHz, $CDCl_3$)

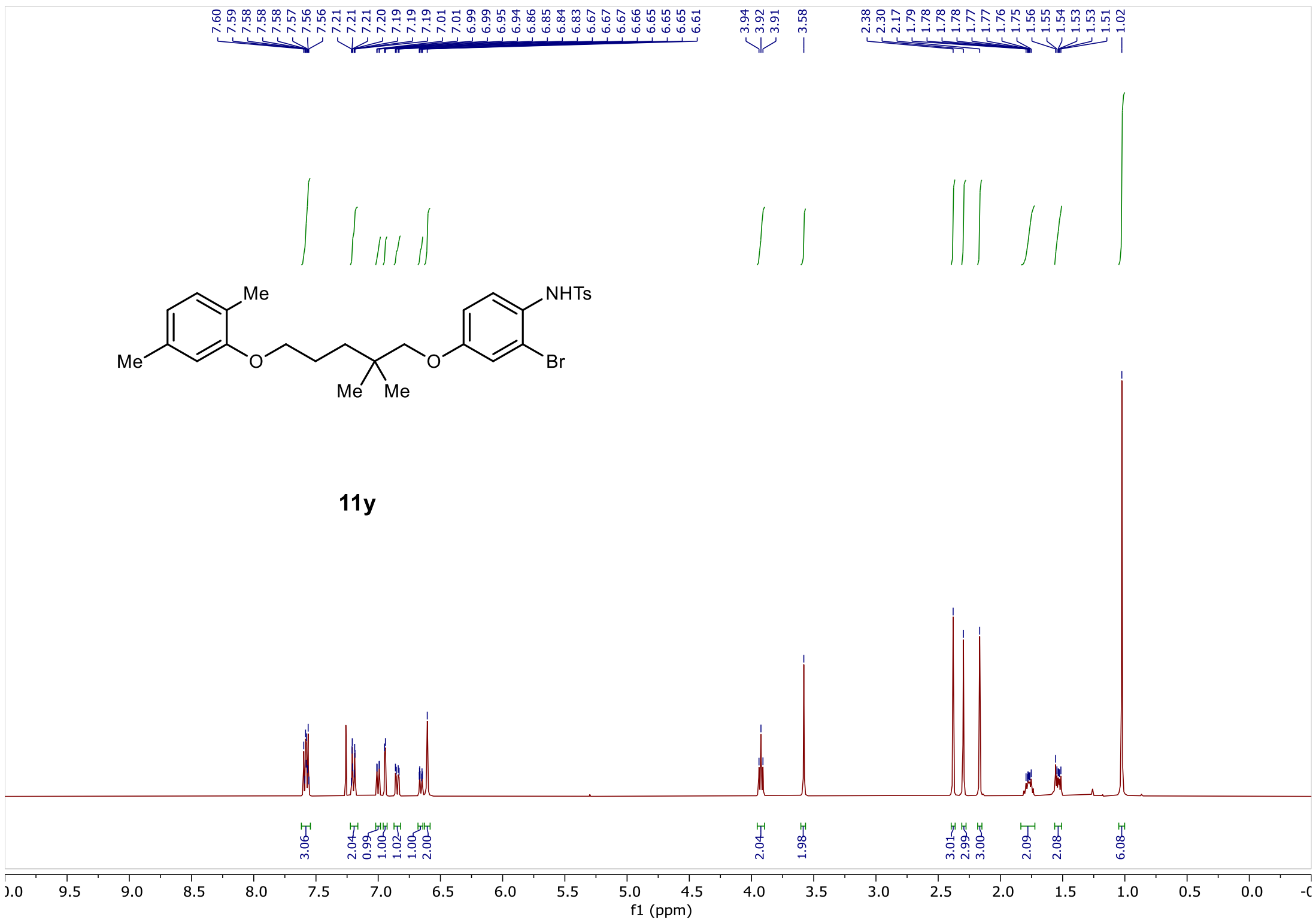

$^{13}C$ NMR (101 MHz, $CDCl_3$)

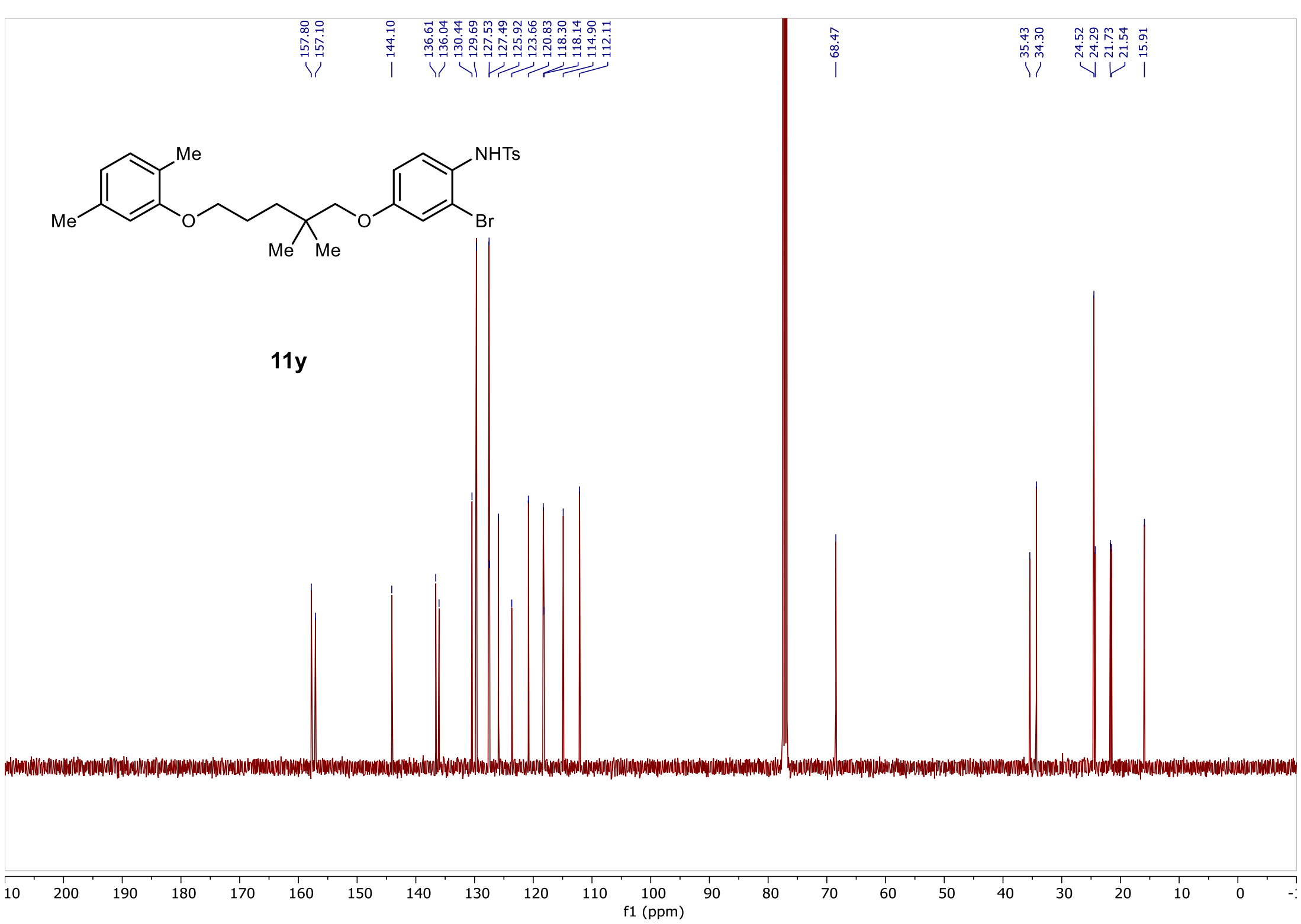

$^1$H NMR (400 MHz, CDCl$_3$)

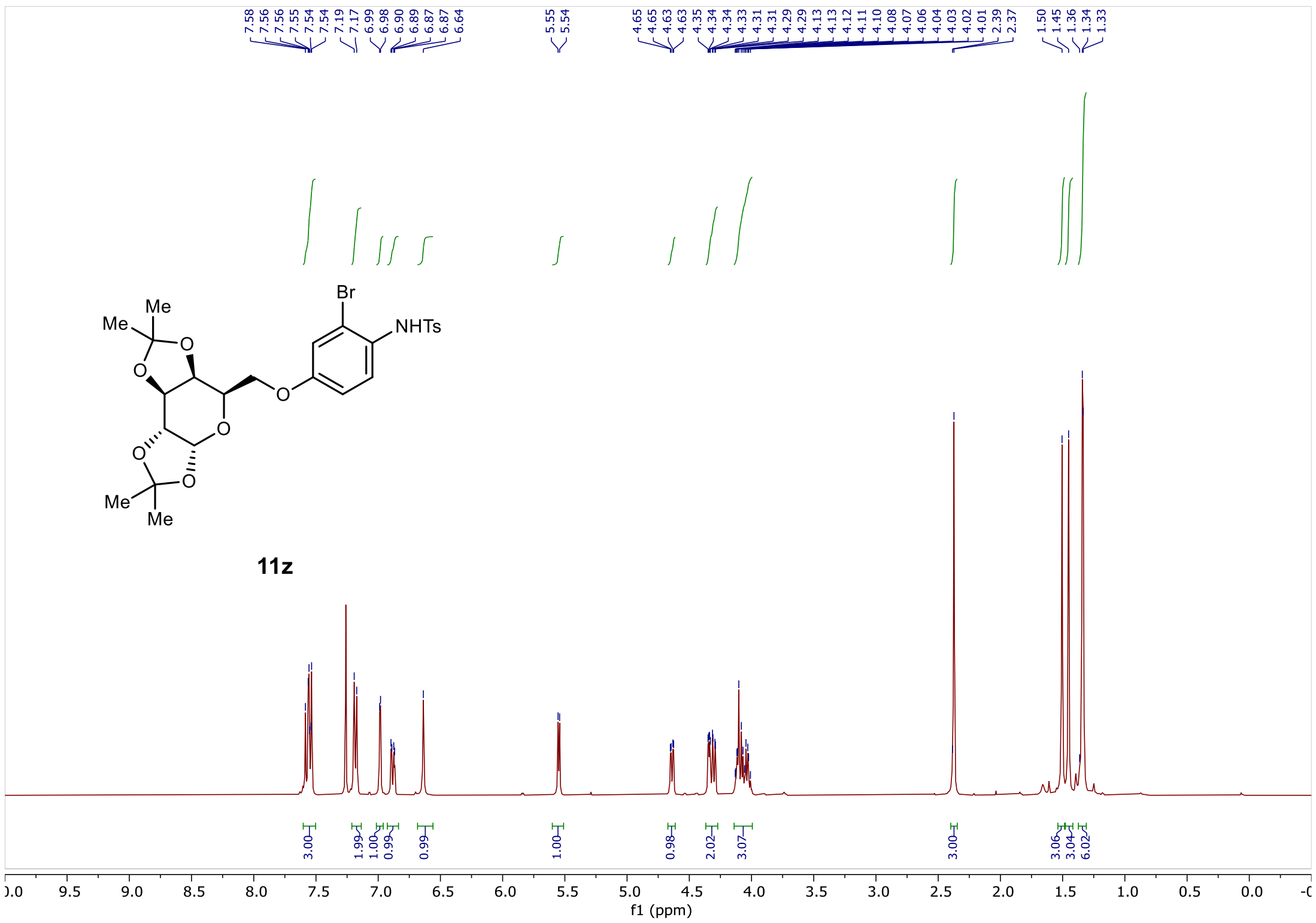

$^{13}$C NMR (101 MHz, CDCl$_3$)

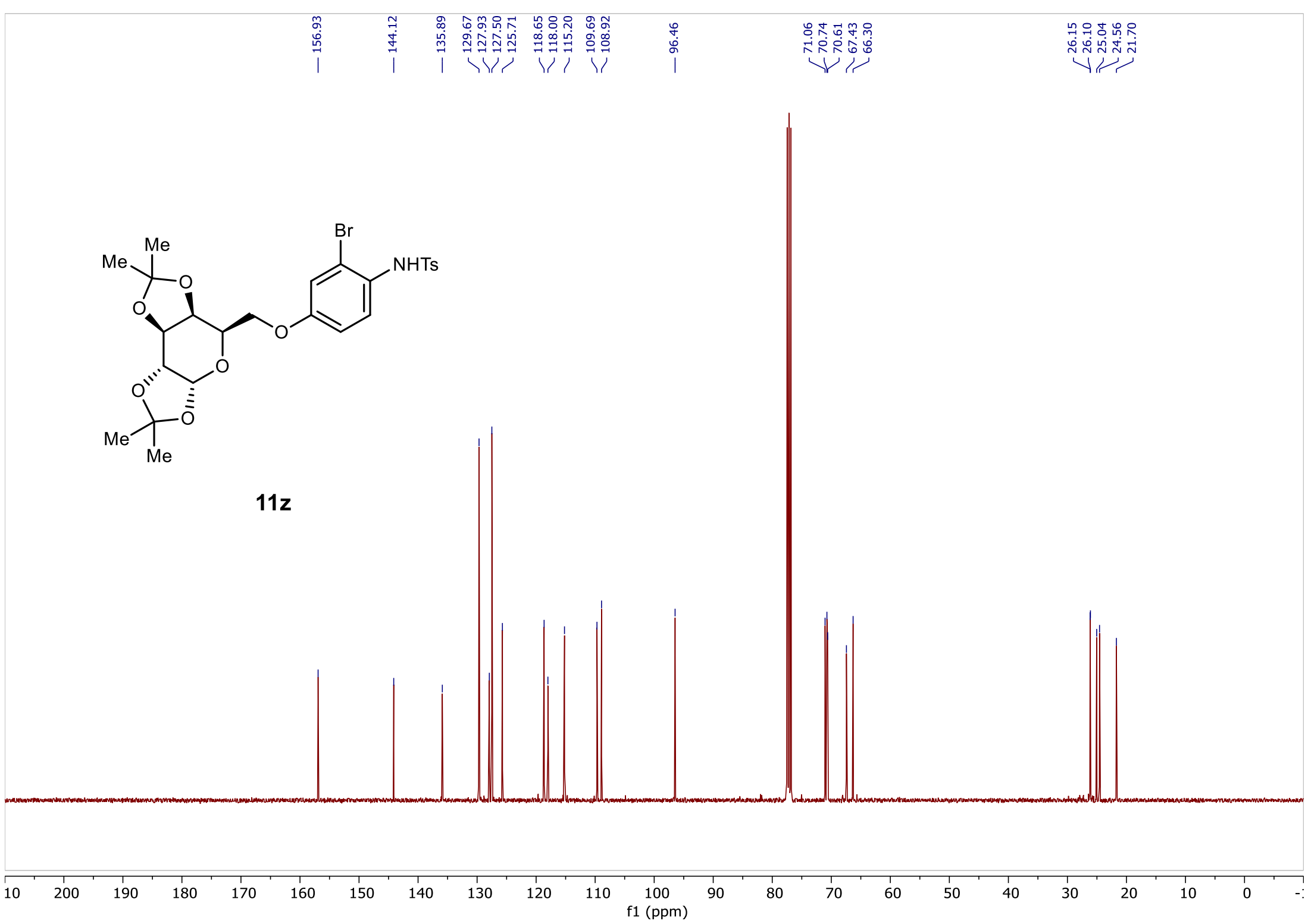

$^{1}H$ NMR (400 MHz, $CDCl_3$)

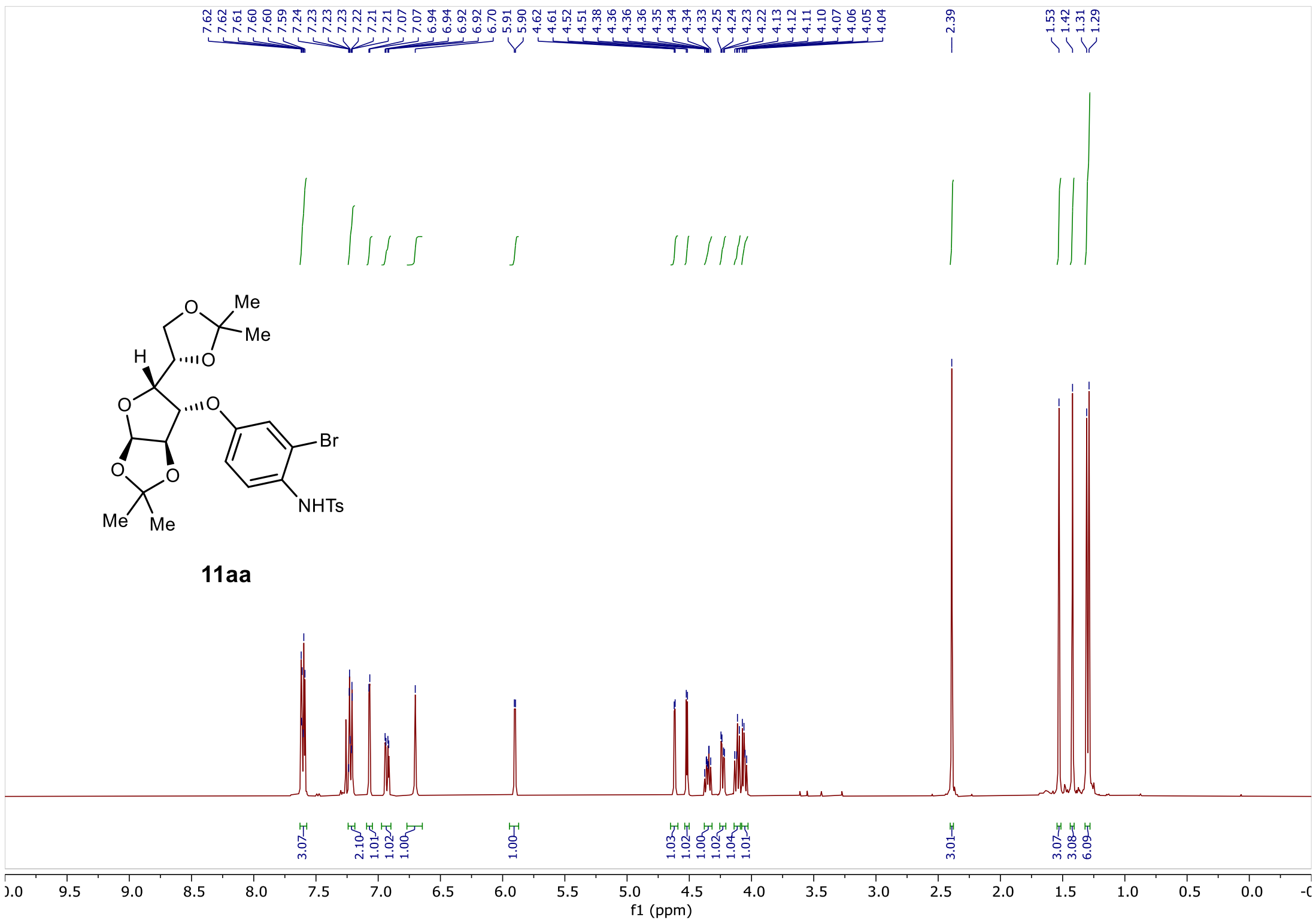


$^{13}C$ NMR (101 MHz, $CDCl_3$)

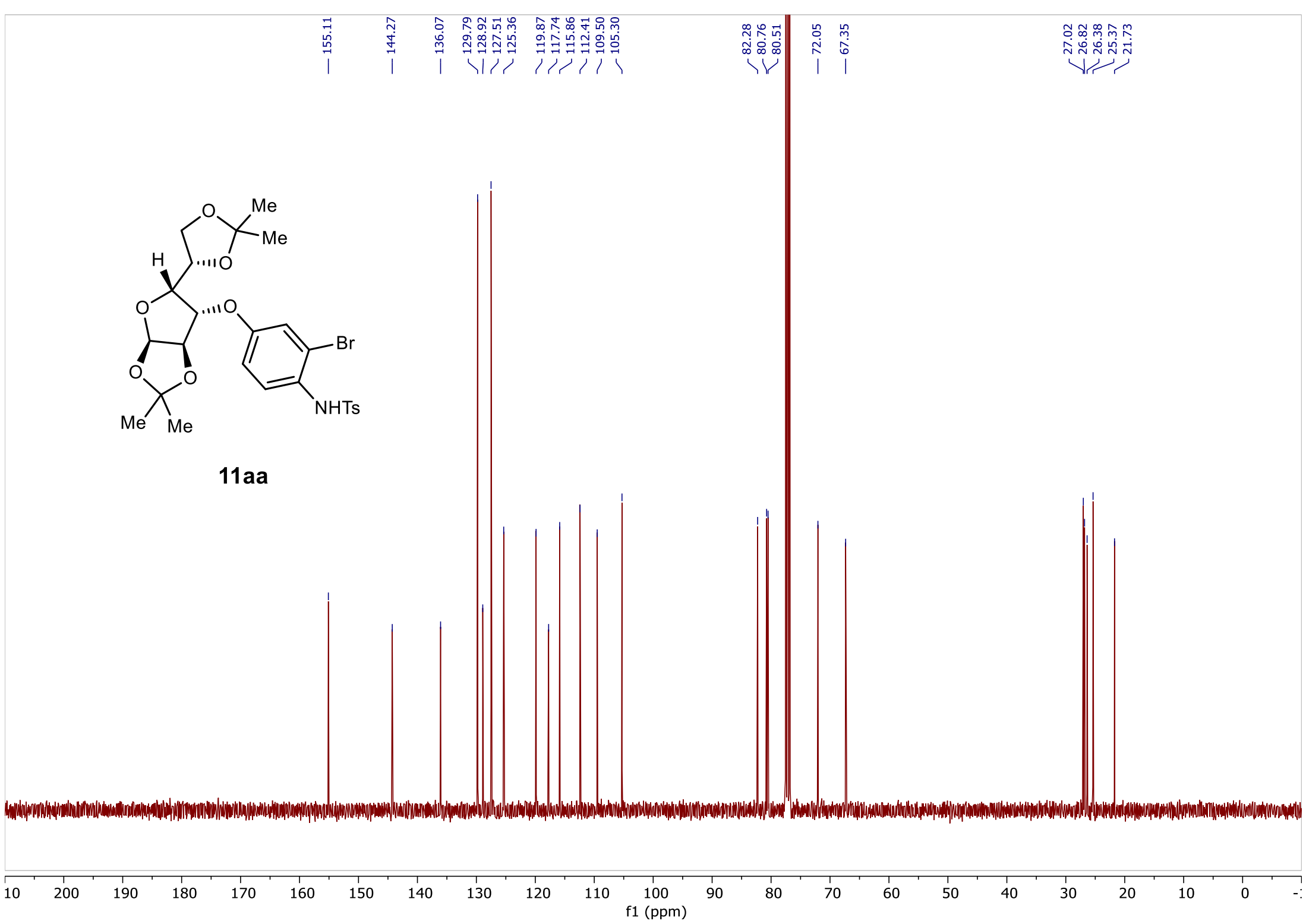

$^1$H NMR (400 MHz, $CDCl_3$)

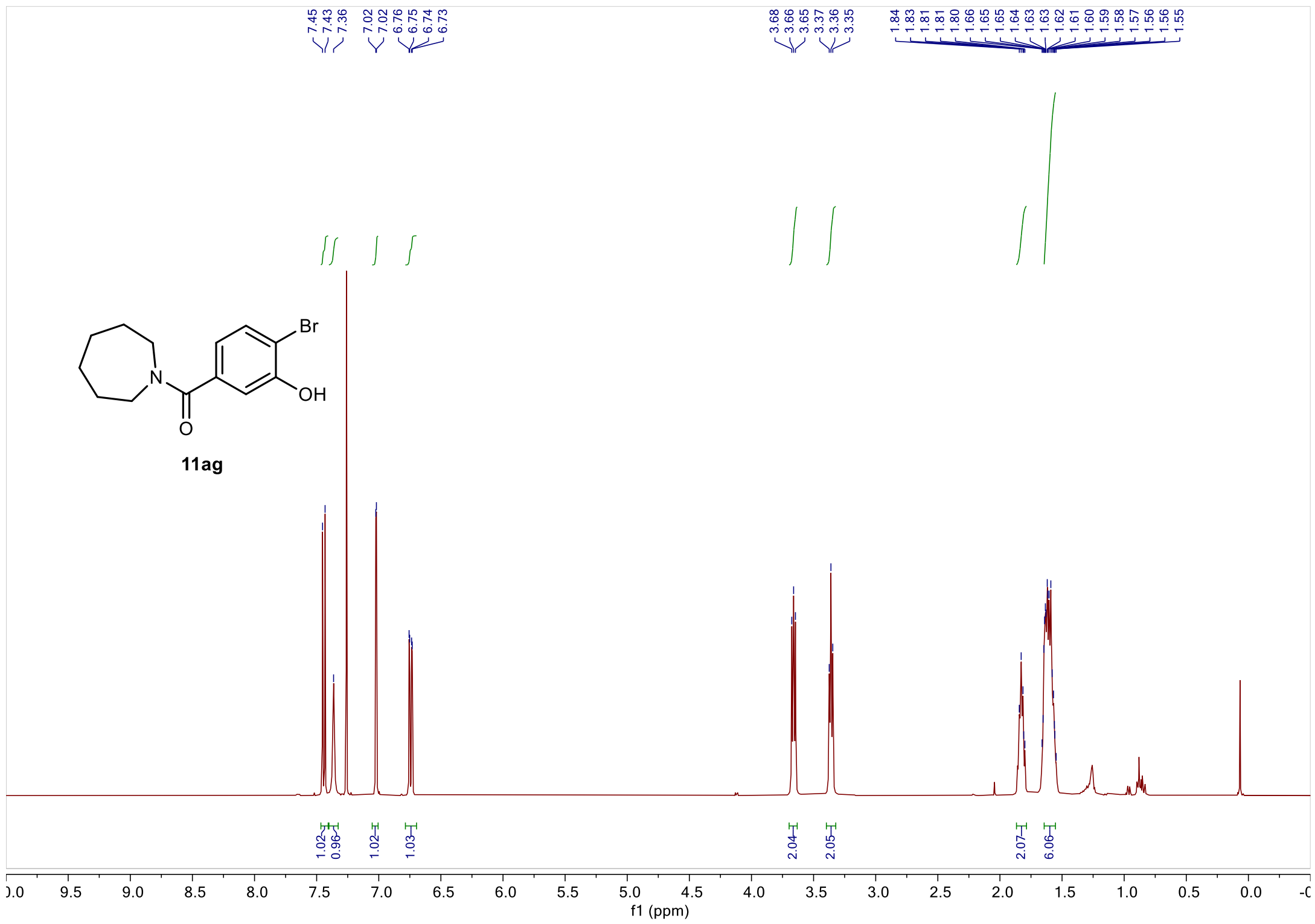

$^{13}$C NMR (101 MHz, $CDCl_3$)

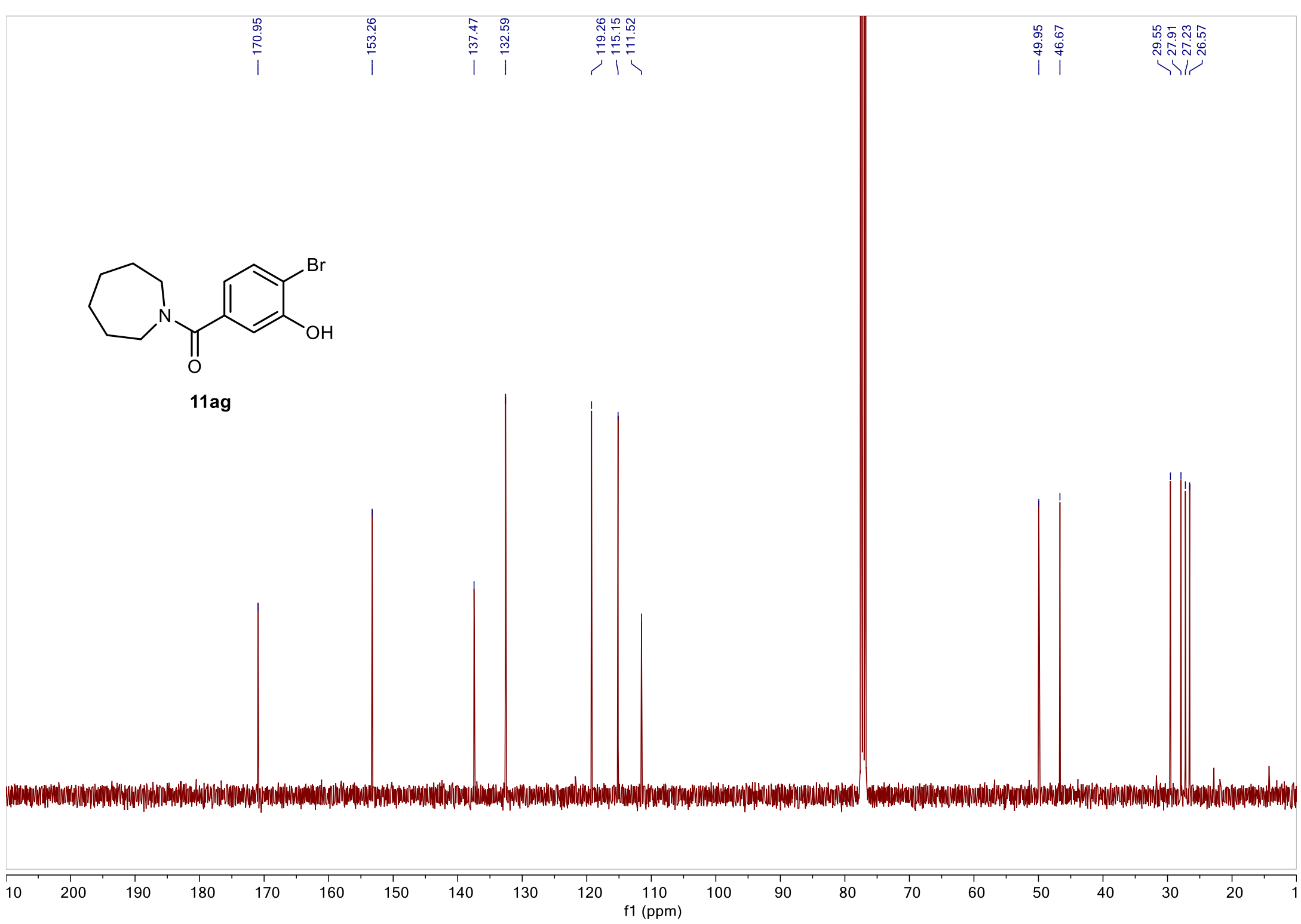

$^{1}$H NMR (400 MHz, $CDCl_3$)

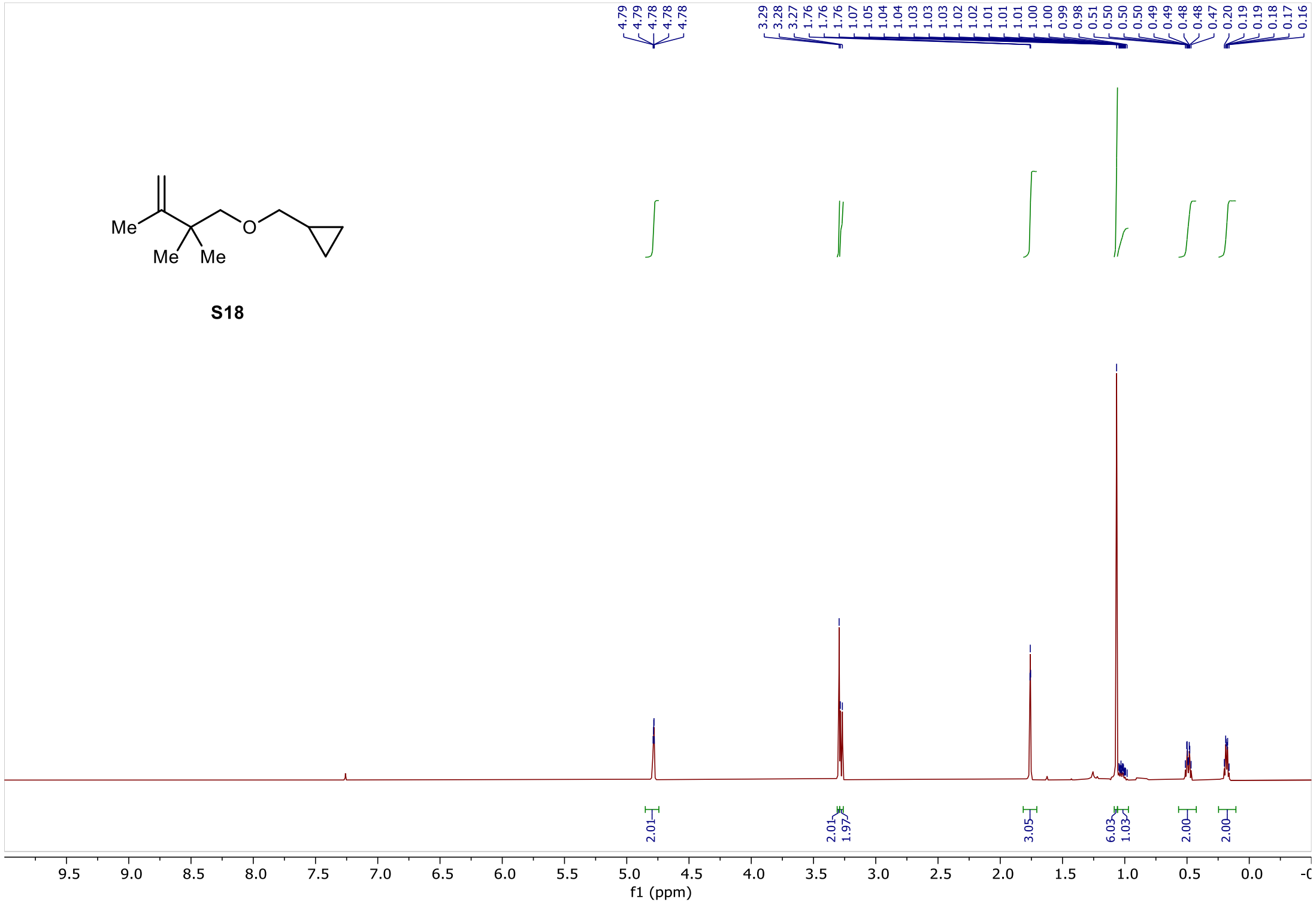


$^{13}$C NMR (101 MHz, $CDCl_3$)

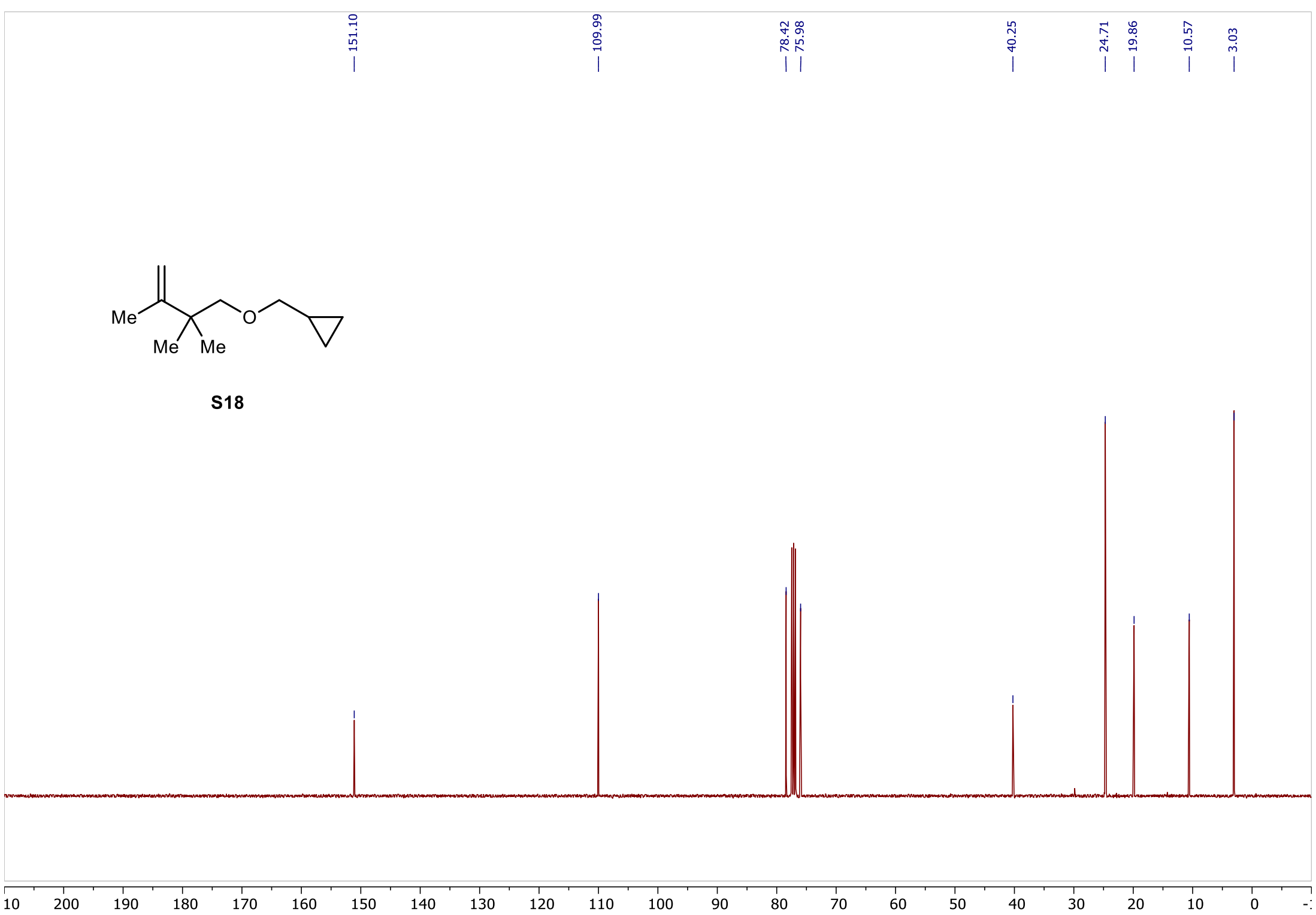

$^{1}H$ NMR (400 MHz, $CDCl_3$)

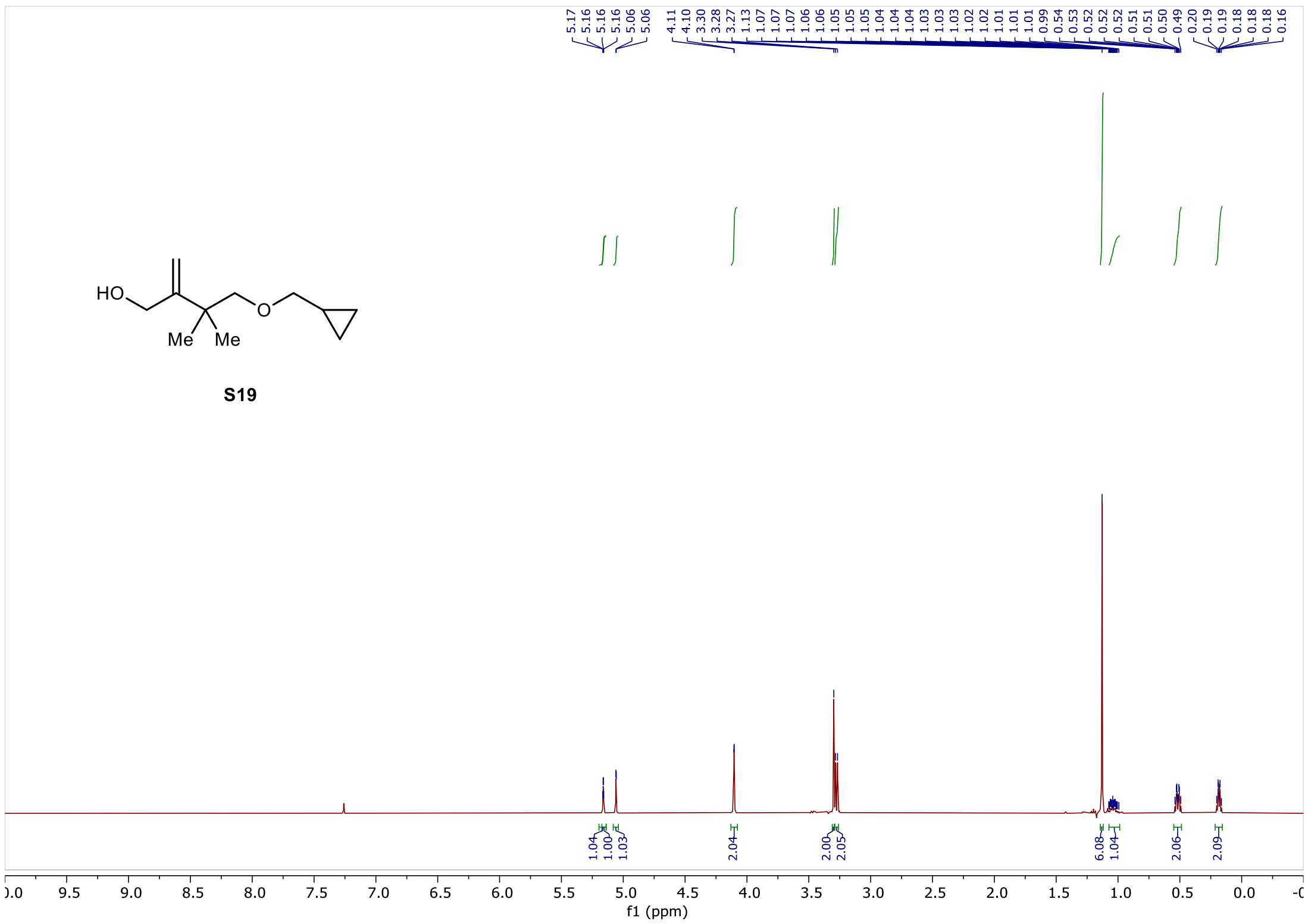

$^{13}C$ NMR (101 MHz, $CDCl_3$)

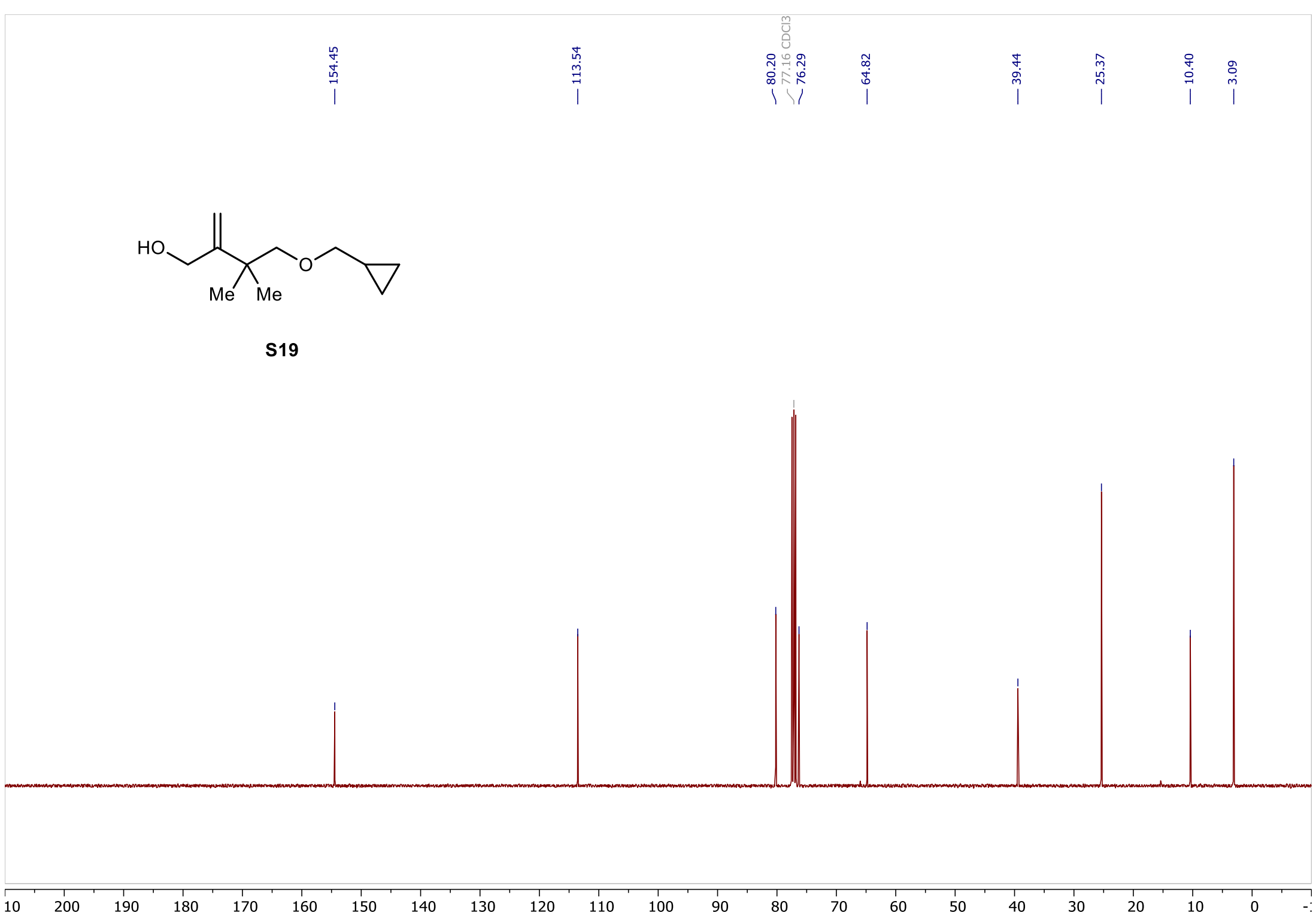

$^{1}H$ NMR (400 MHz, $CDCl_3$)

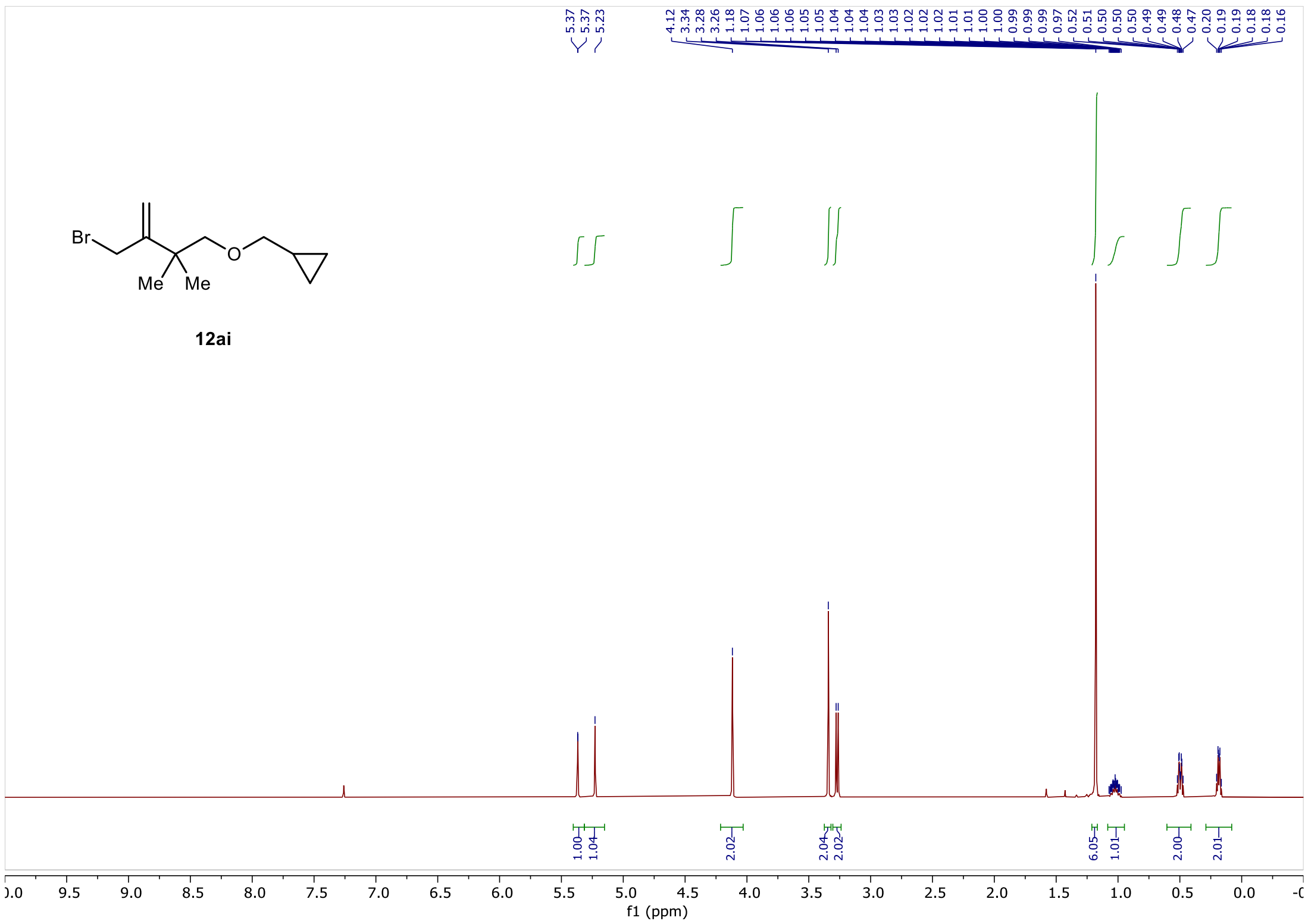


$^{13}C$ NMR (101 MHz, $CDCl_3$)

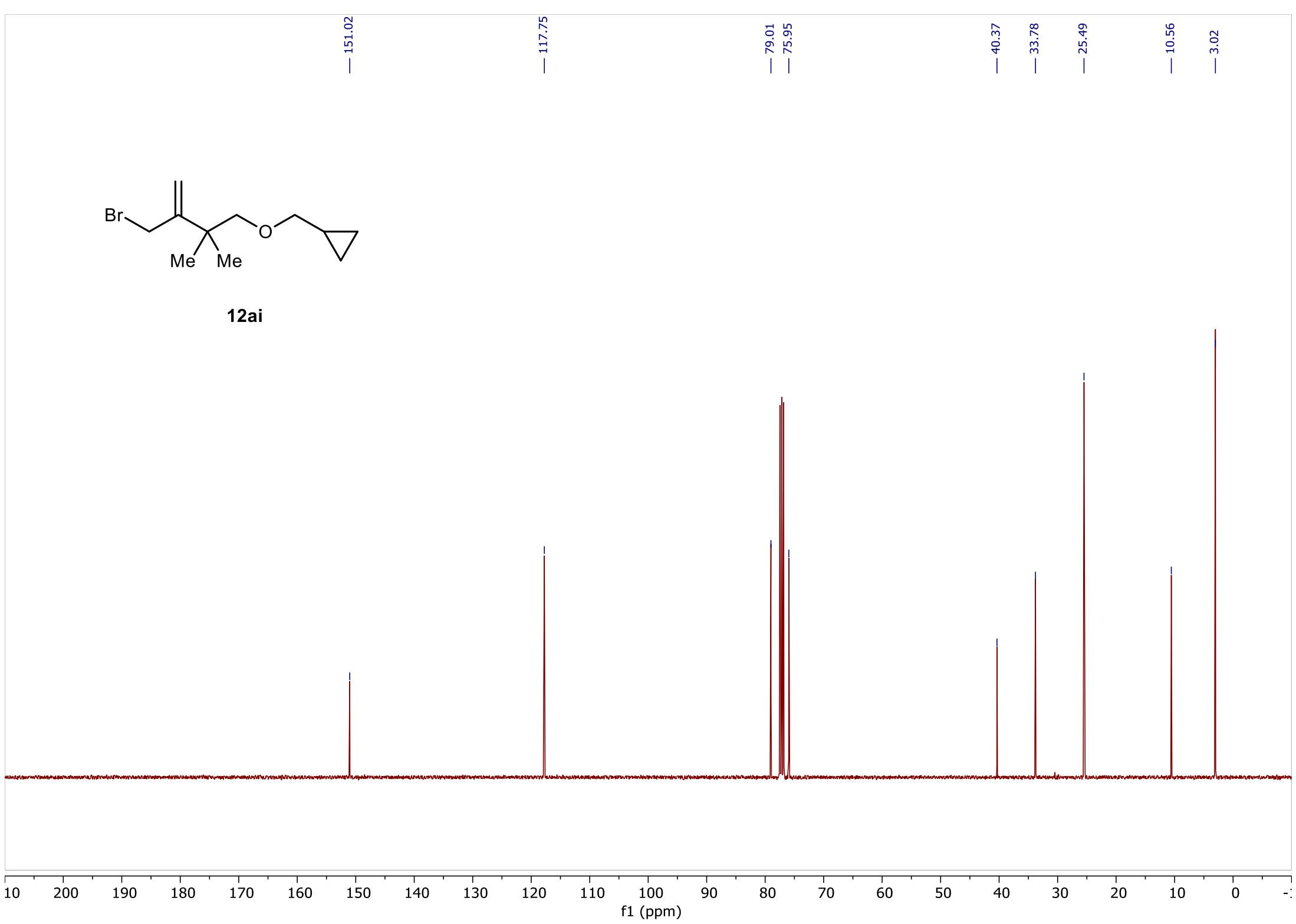

$^{1}H$ NMR (400 MHz, $CDCl_3$)

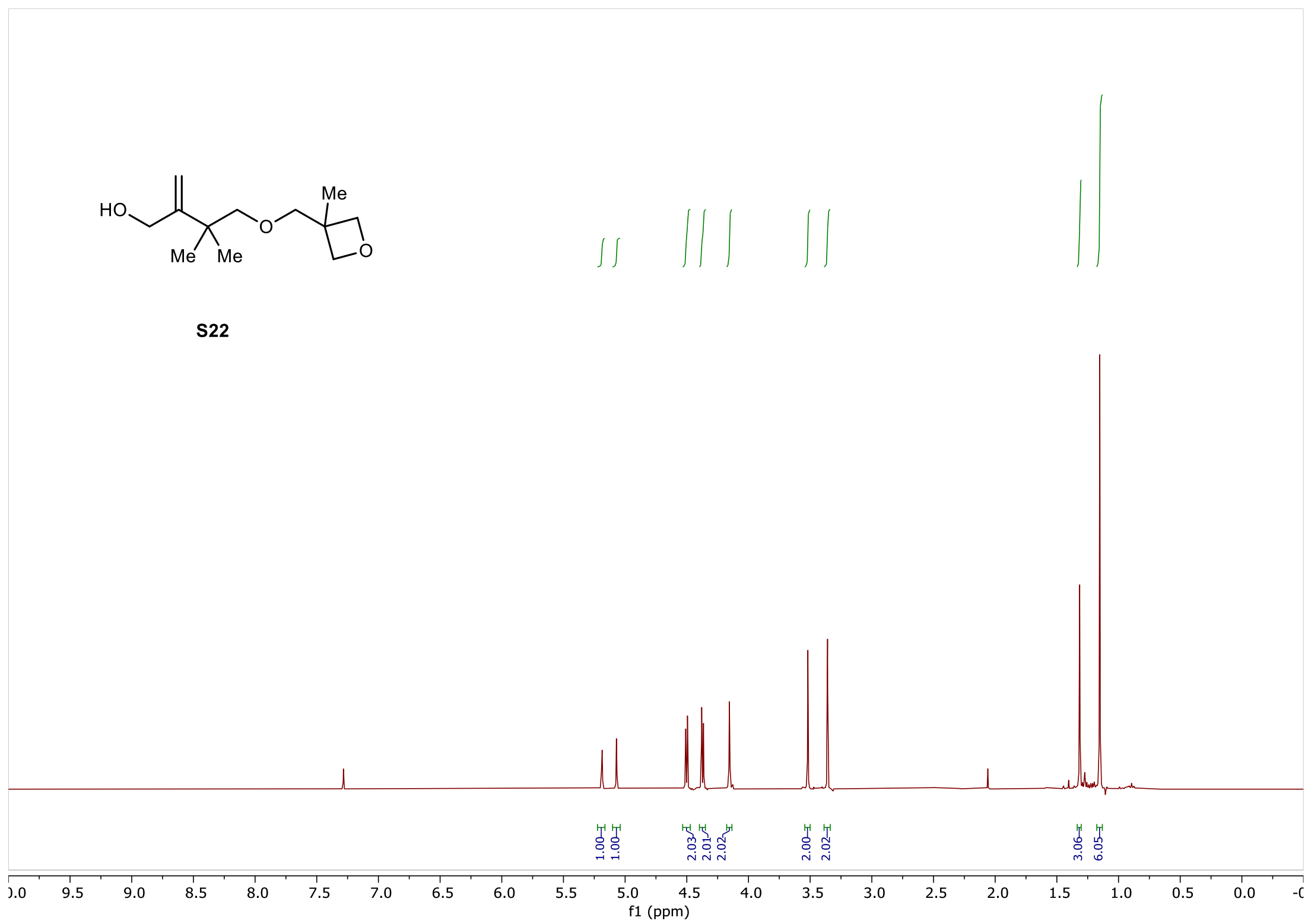


$^{13}C$ NMR (101 MHz, $CDCl_3$)

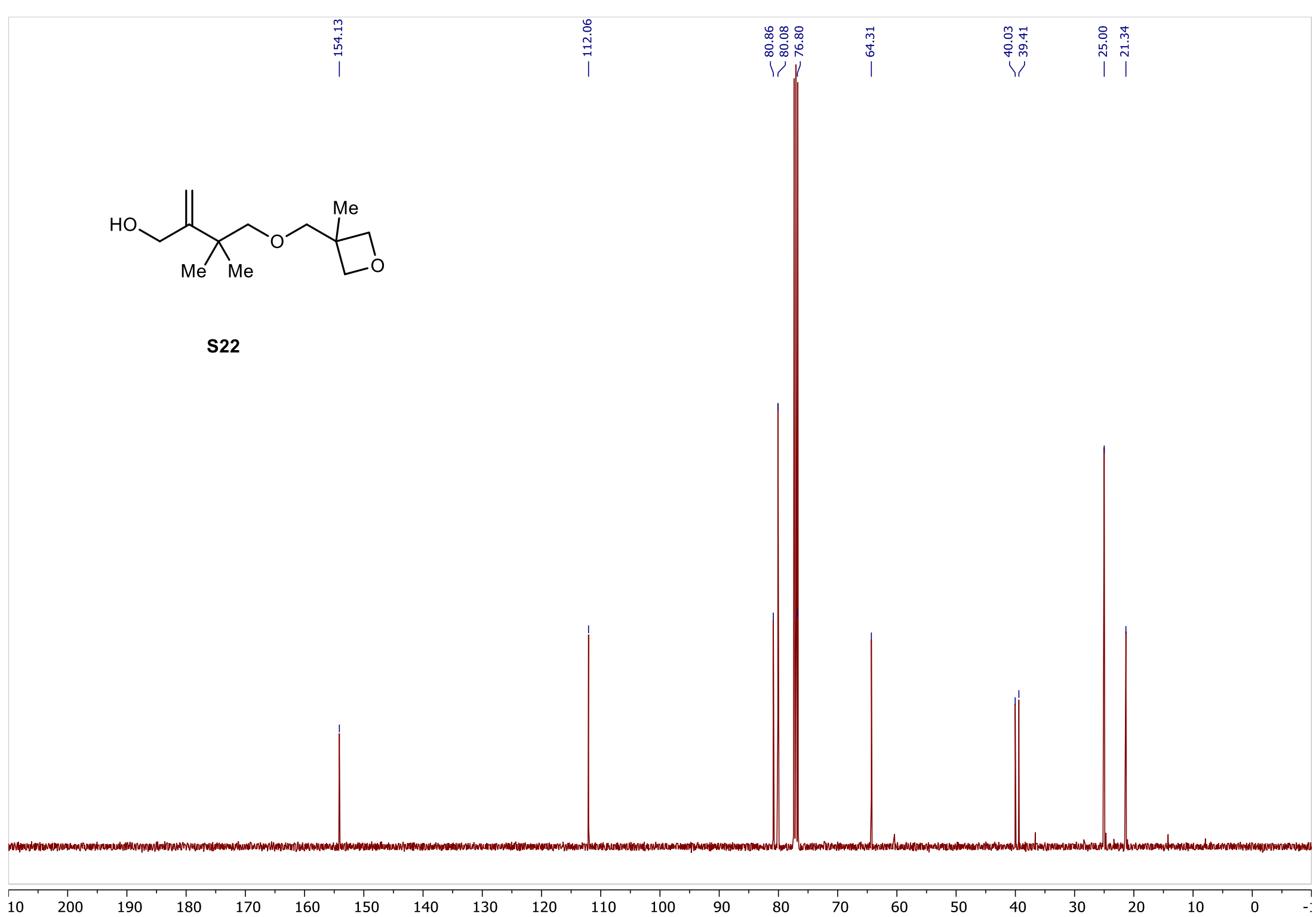

$^1$H NMR (400 MHz, $CDCl_3$)

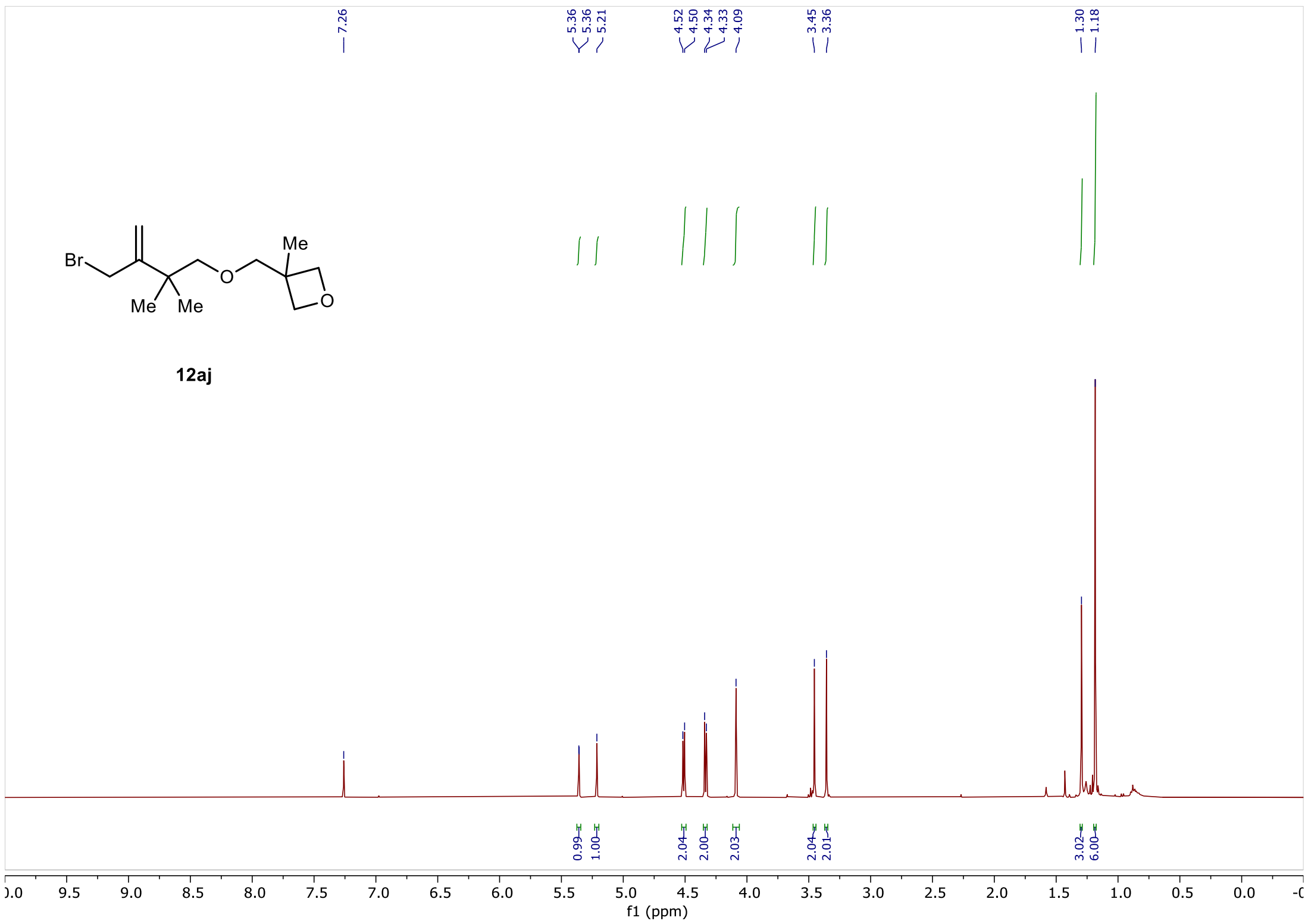


$^{13}$C NMR (101 MHz, $CDCl_3$)

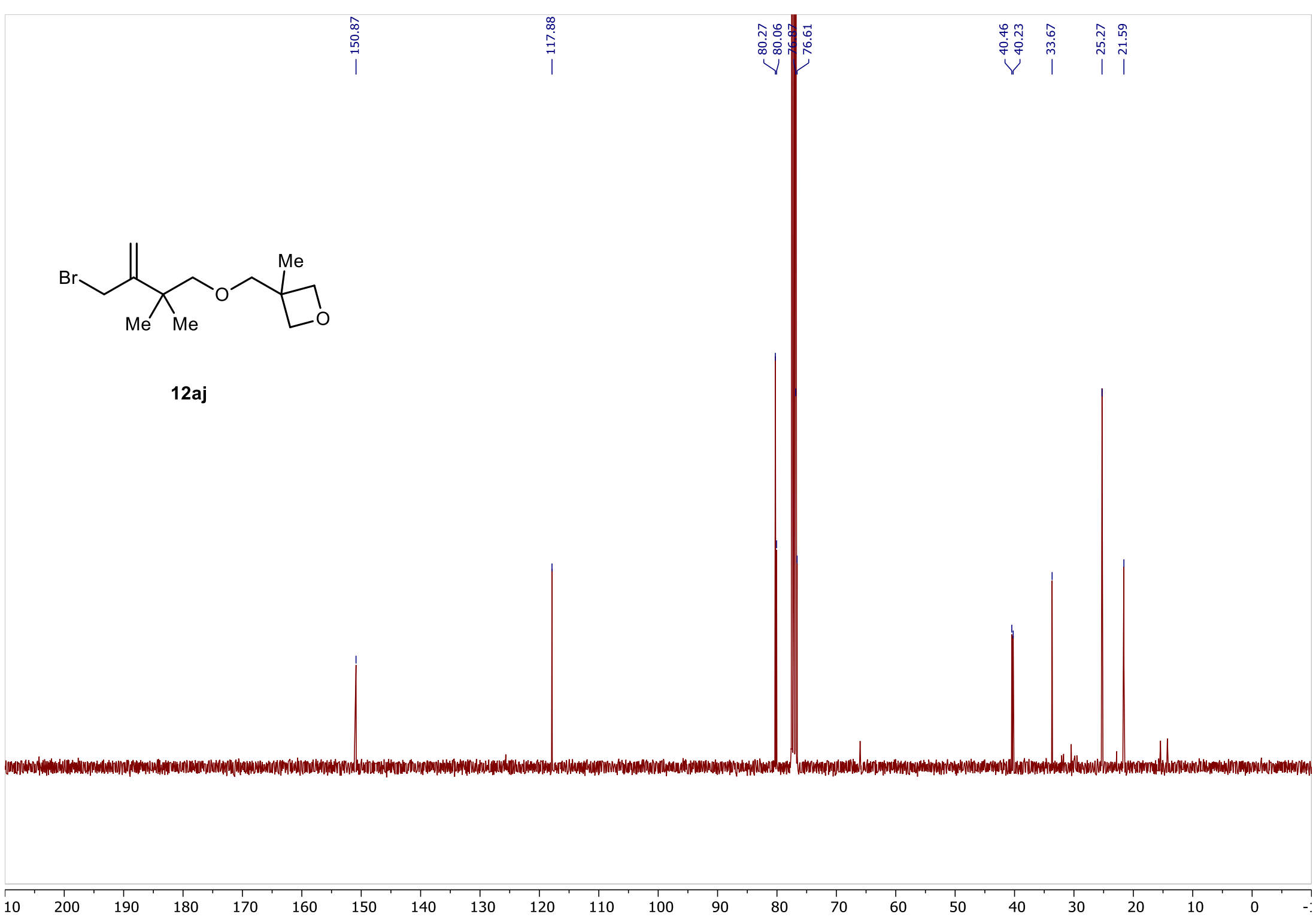

$^{1}H$ NMR (400 MHz, $CDCl_3$)

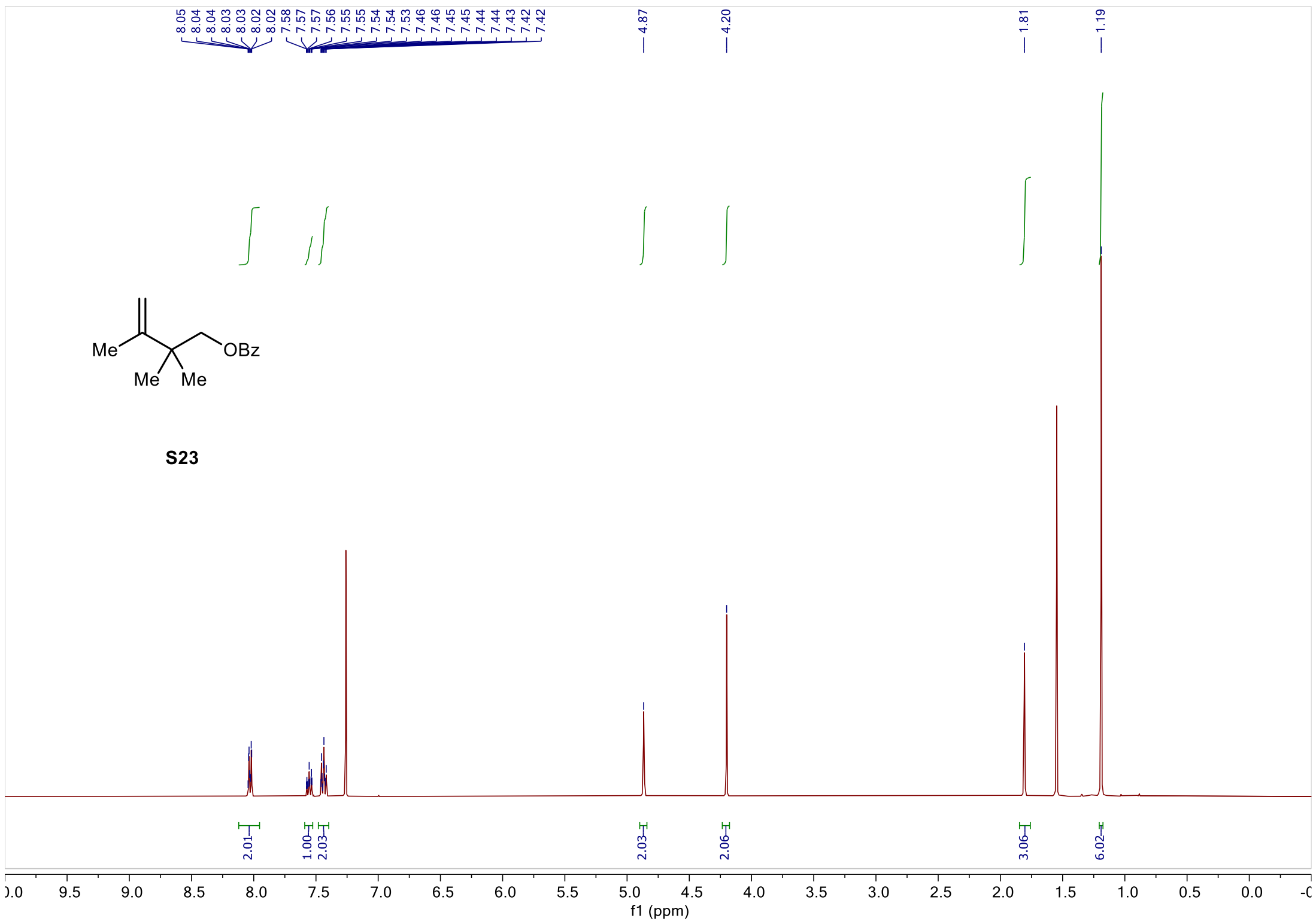

$^{13}C$ NMR (101 MHz, $CDCl_3$)

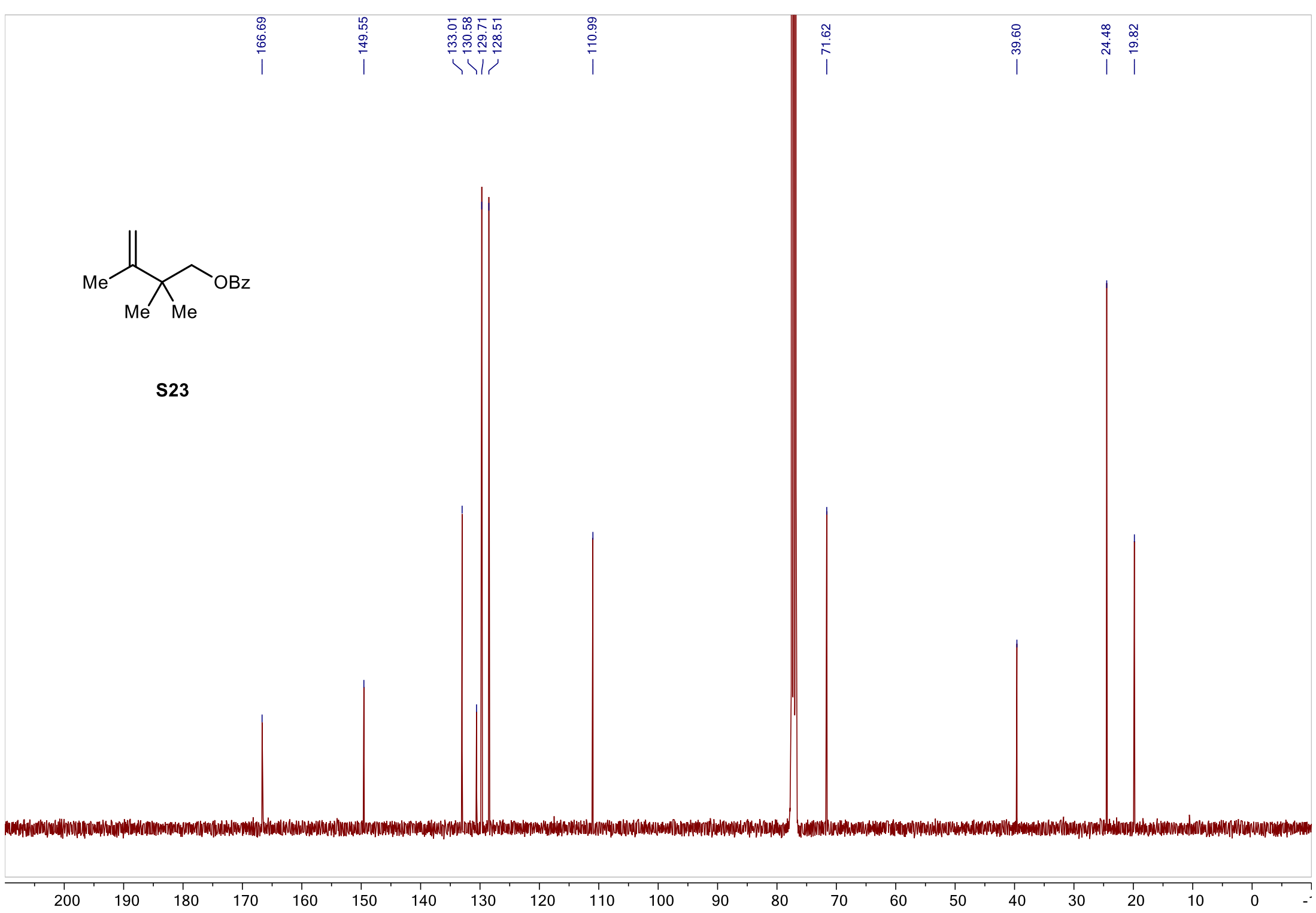

$^{1}H$ NMR (400 MHz, $CDCl_3$)

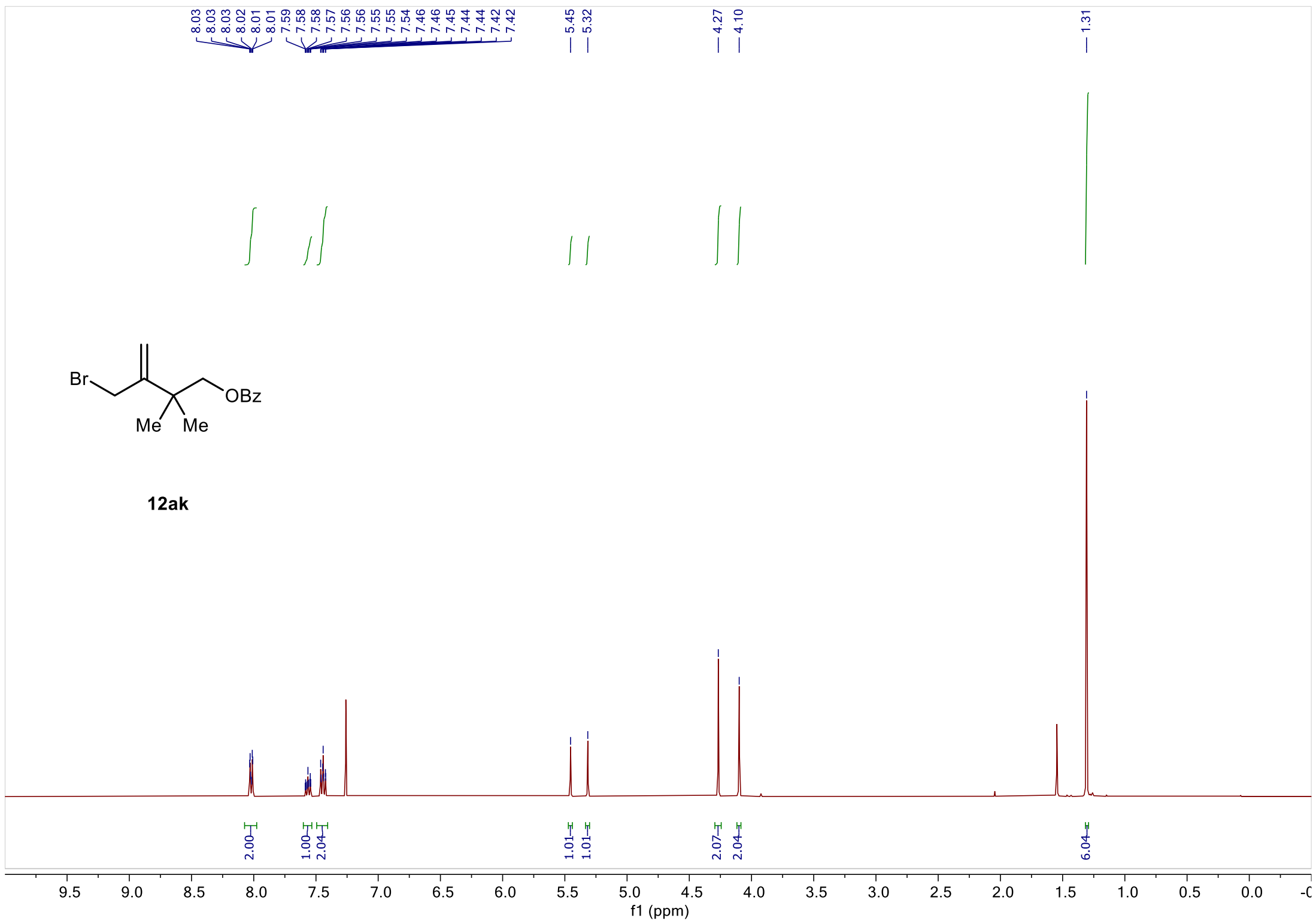


$^{13}C$ NMR (101 MHz, $CDCl_3$)

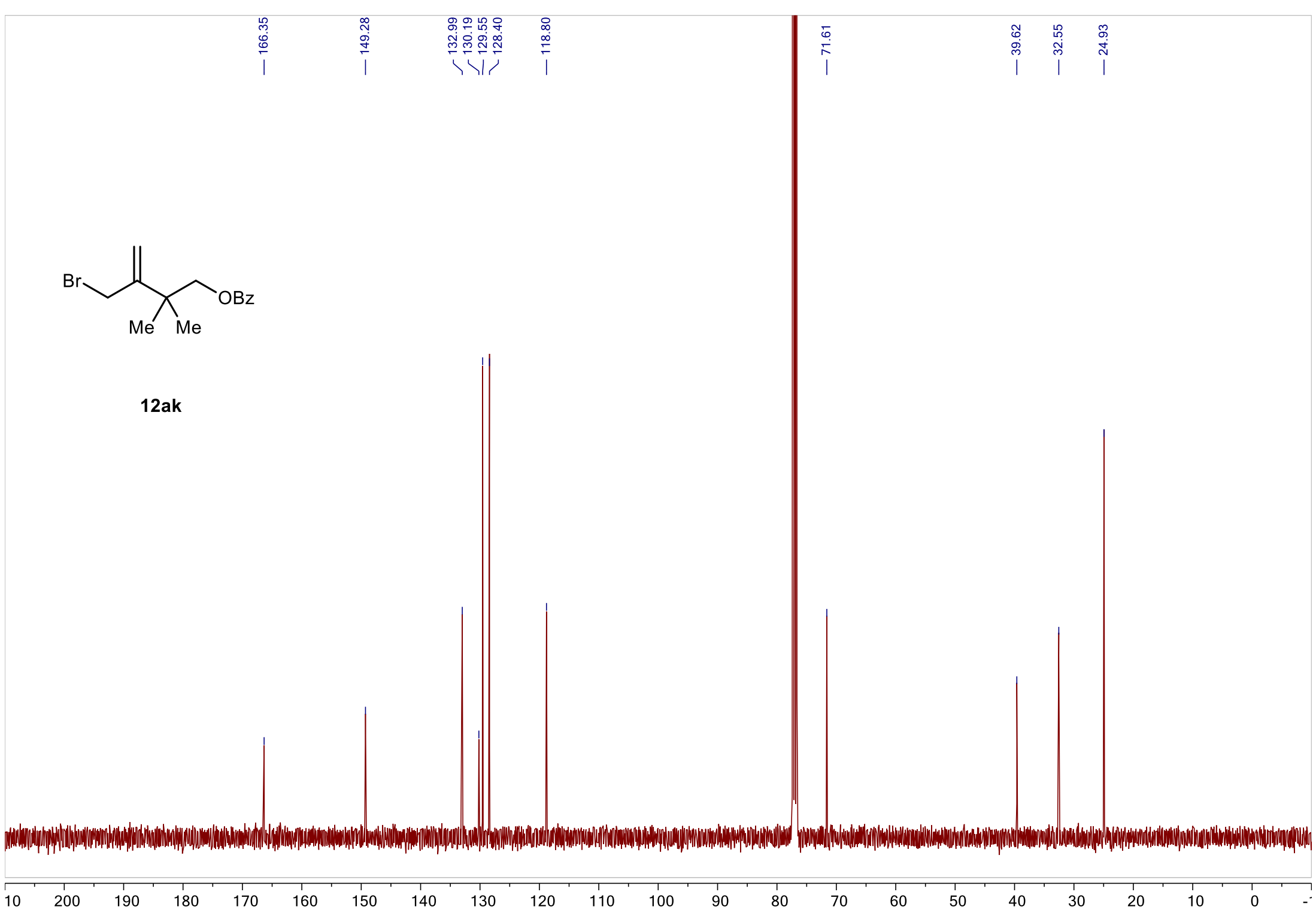

$^{1}H$ NMR (400 MHz, $CDCl_3$)

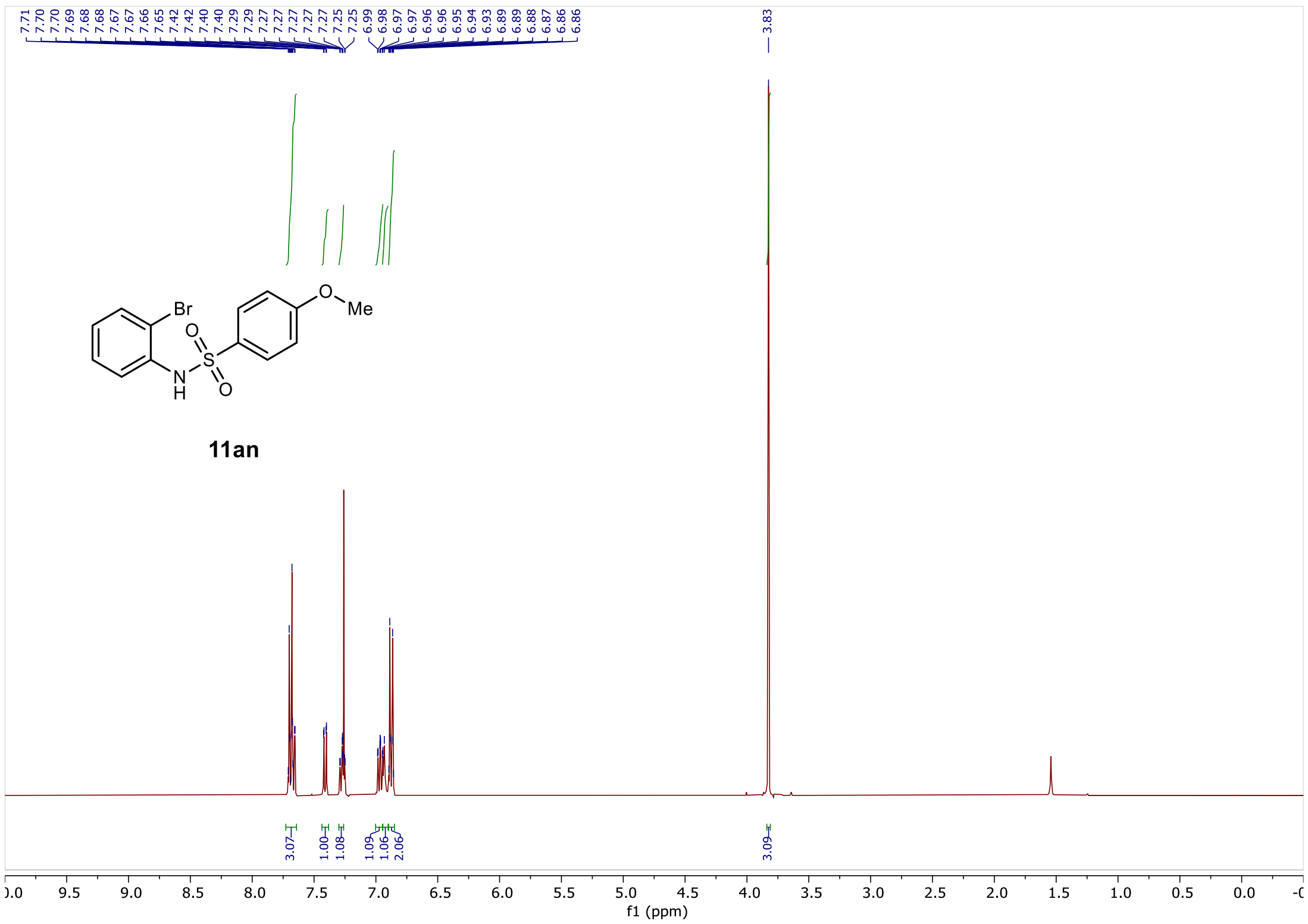

$^{13}C$ NMR (101 MHz, $CDCl_3$)

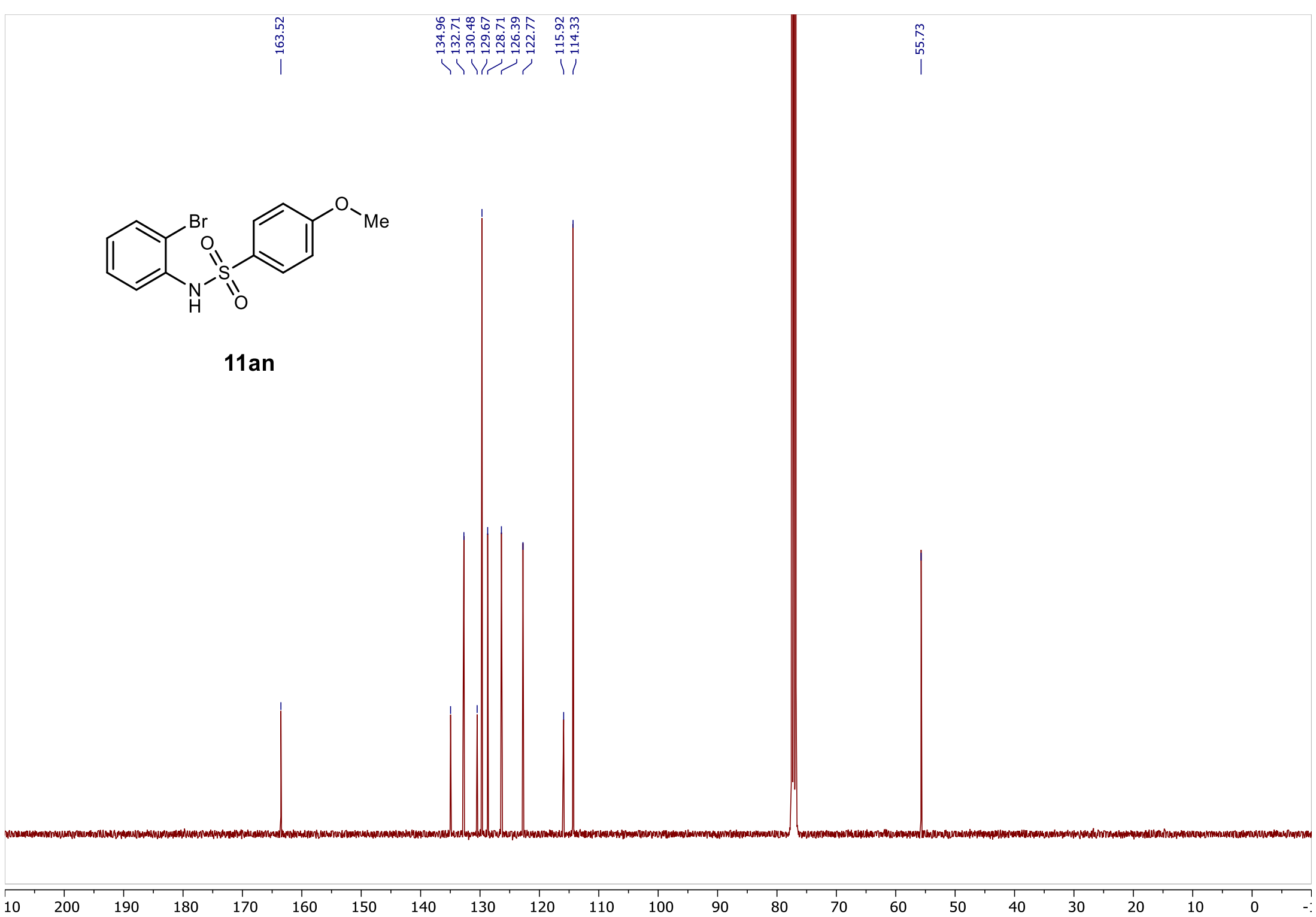

$^{1}H$ NMR (400 MHz, $CDCl_3$)

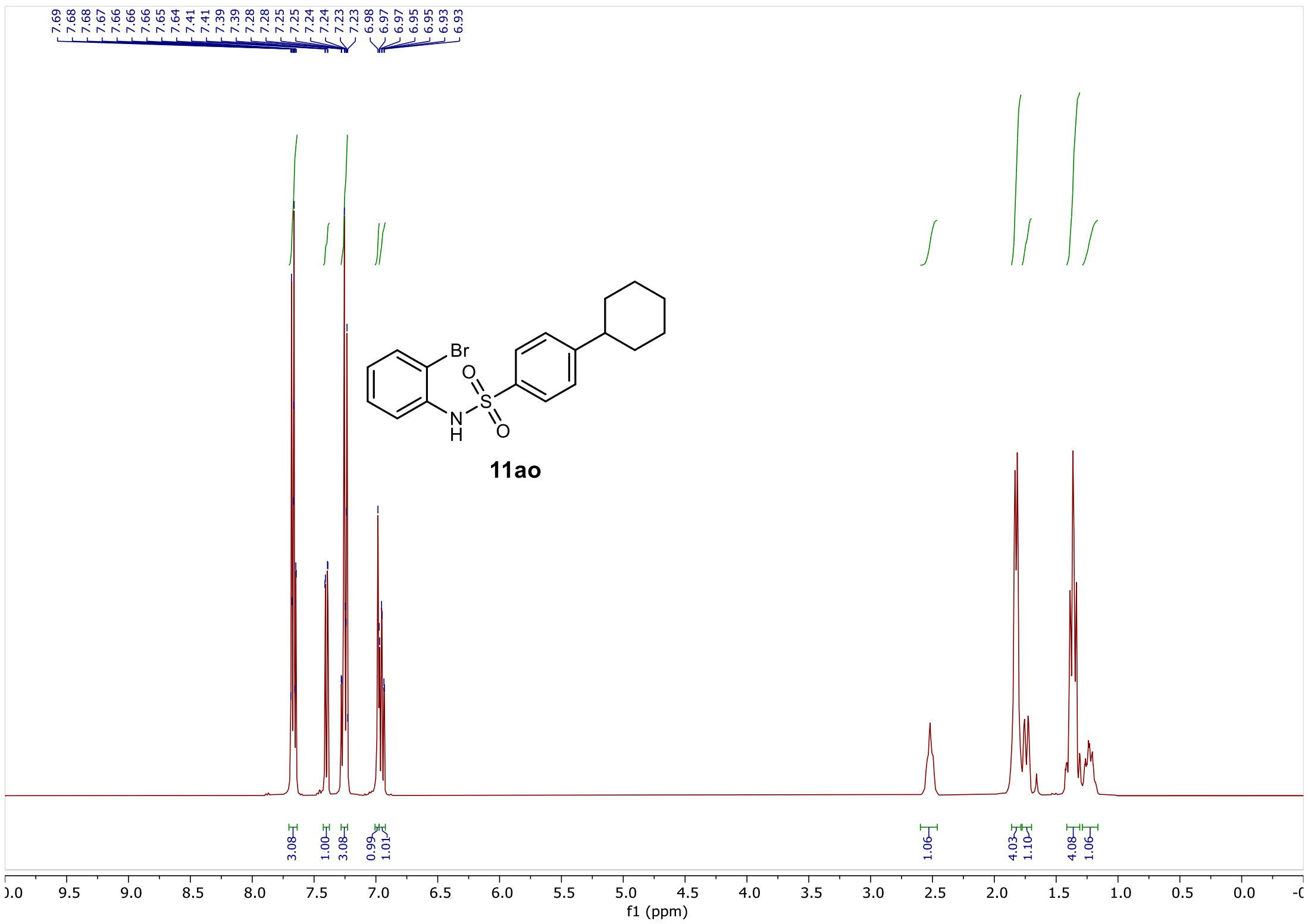


$^{13}C$ NMR (101 MHz, $CDCl_3$)

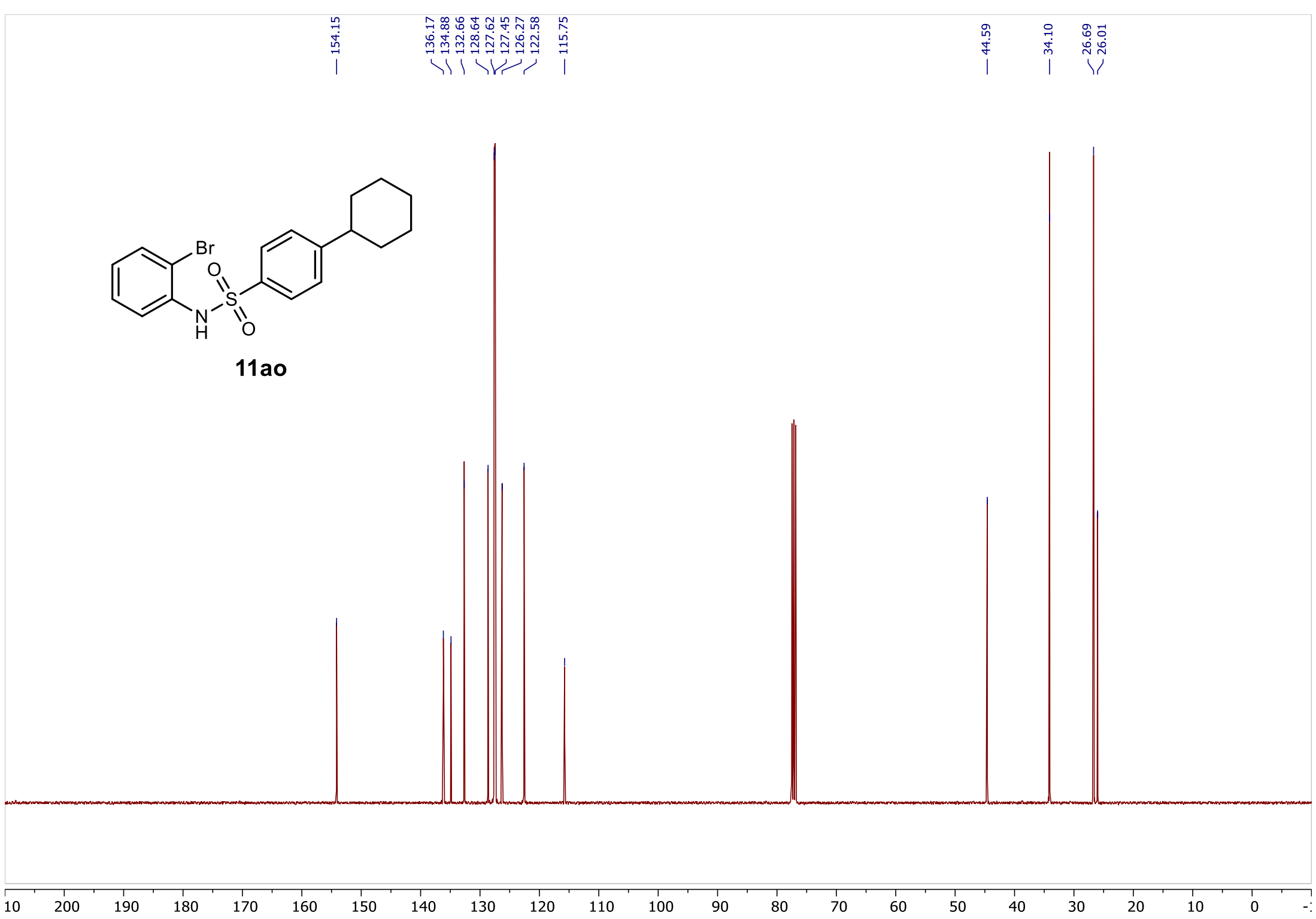

$^{1}H$ NMR (400 MHz, $CDCl_3$)

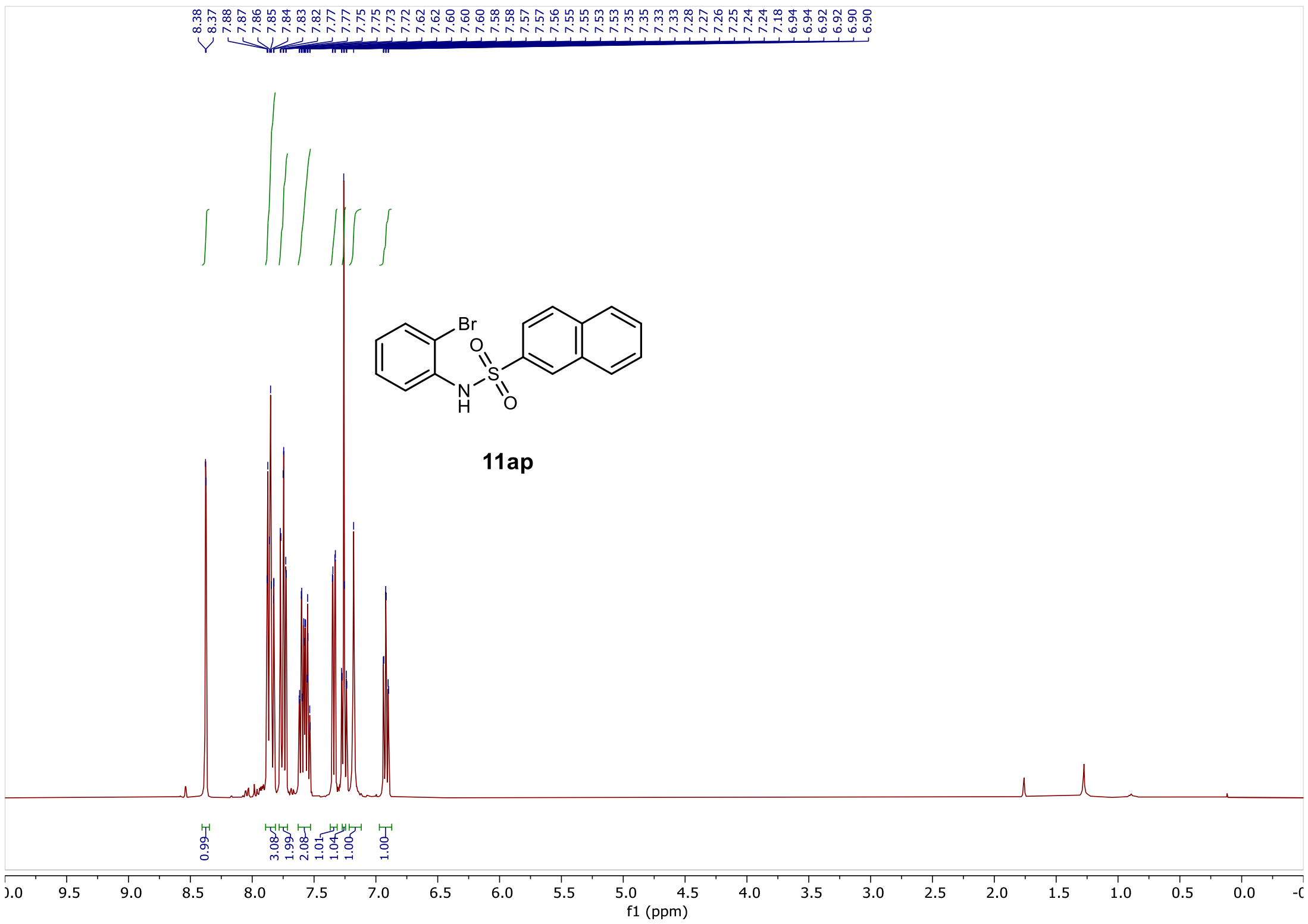


$^{13}C$ NMR (101 MHz, $CDCl_3$)

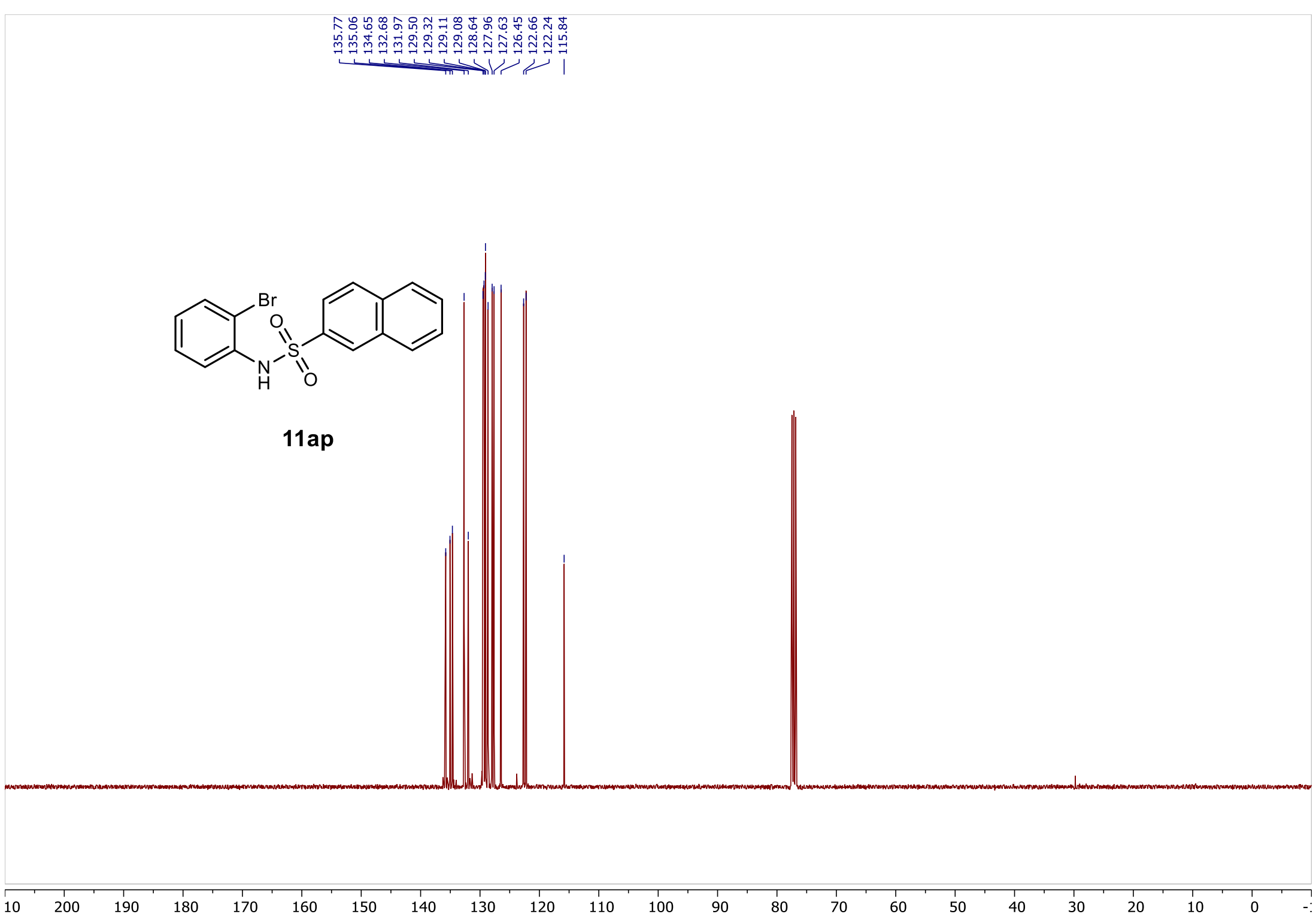

$^{1}$H NMR (400 MHz, $CDCl_3$)

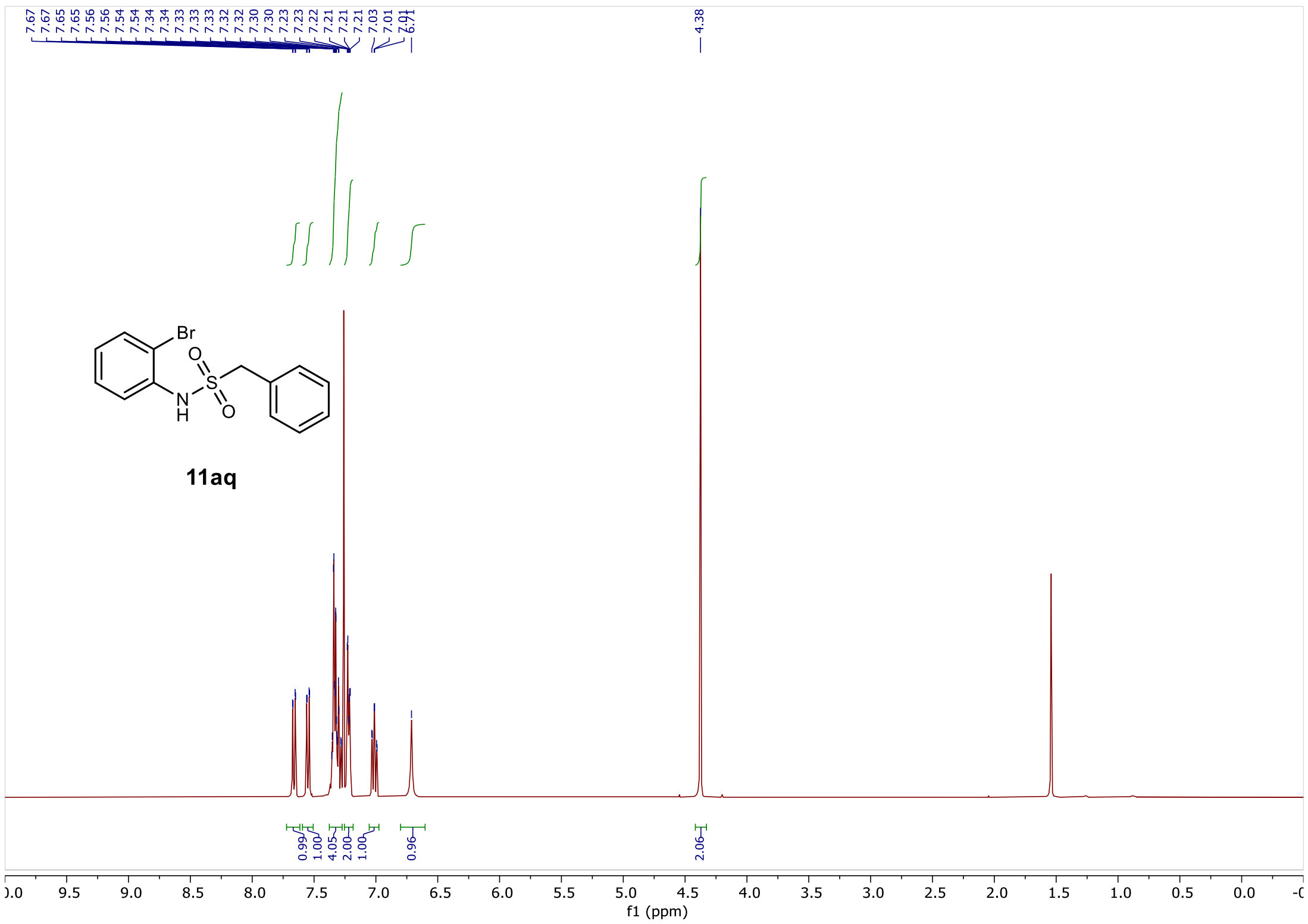

$^{13}$C NMR (101 MHz, $CDCl_3$)

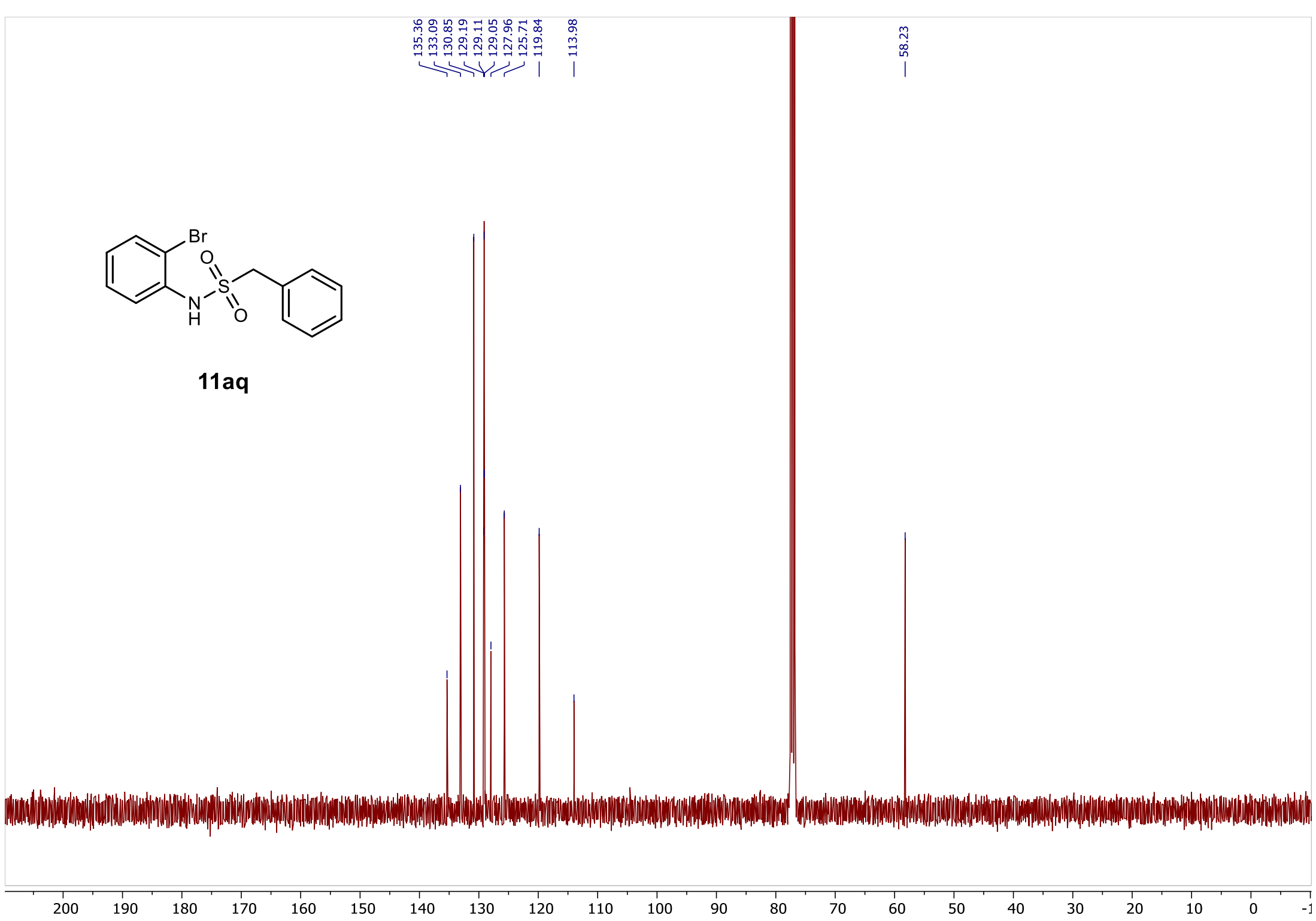

$^{1}$H NMR (400 MHz, $CDCl_3$)

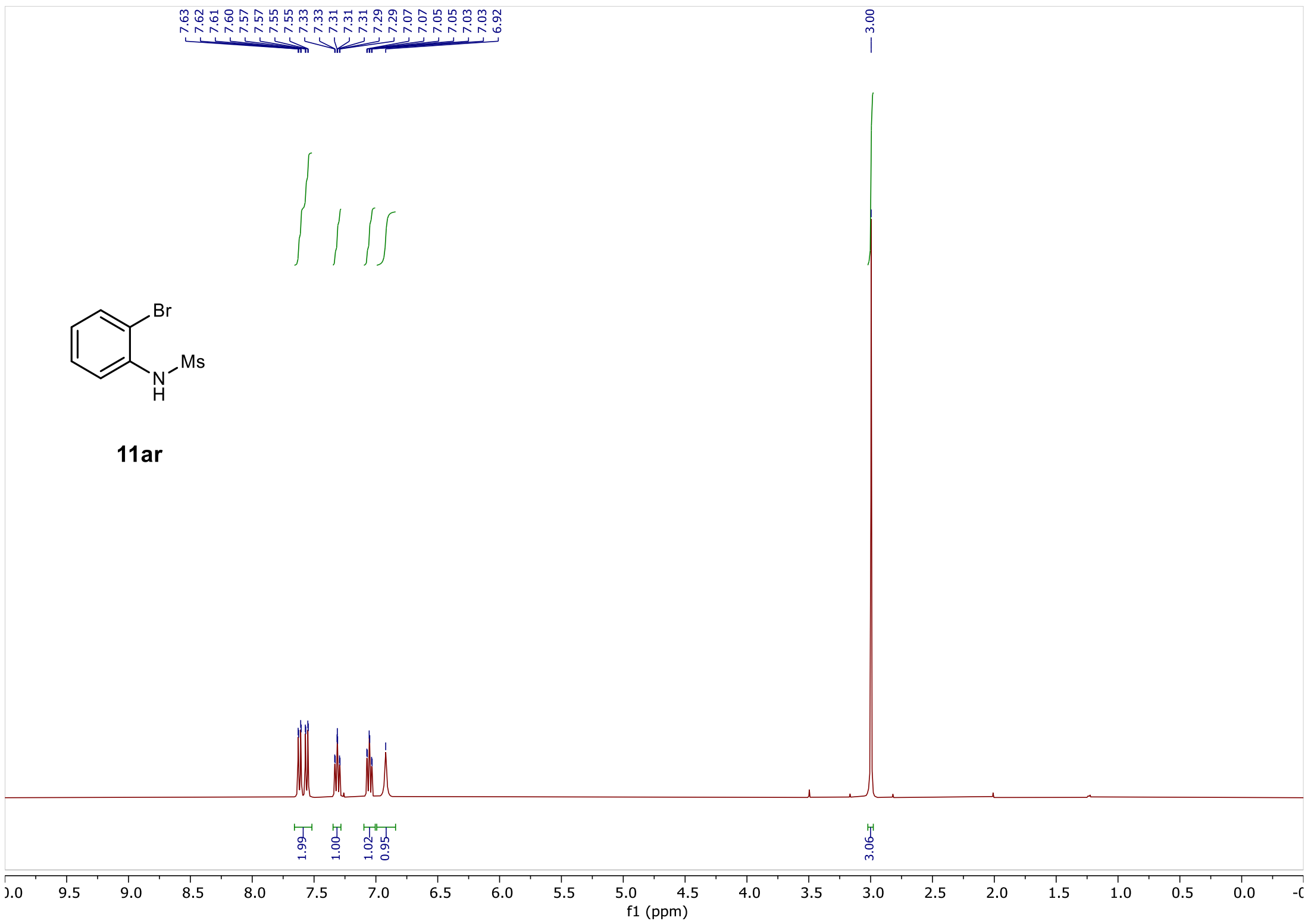


$^{13}$C NMR (101 MHz, $CDCl_3$)

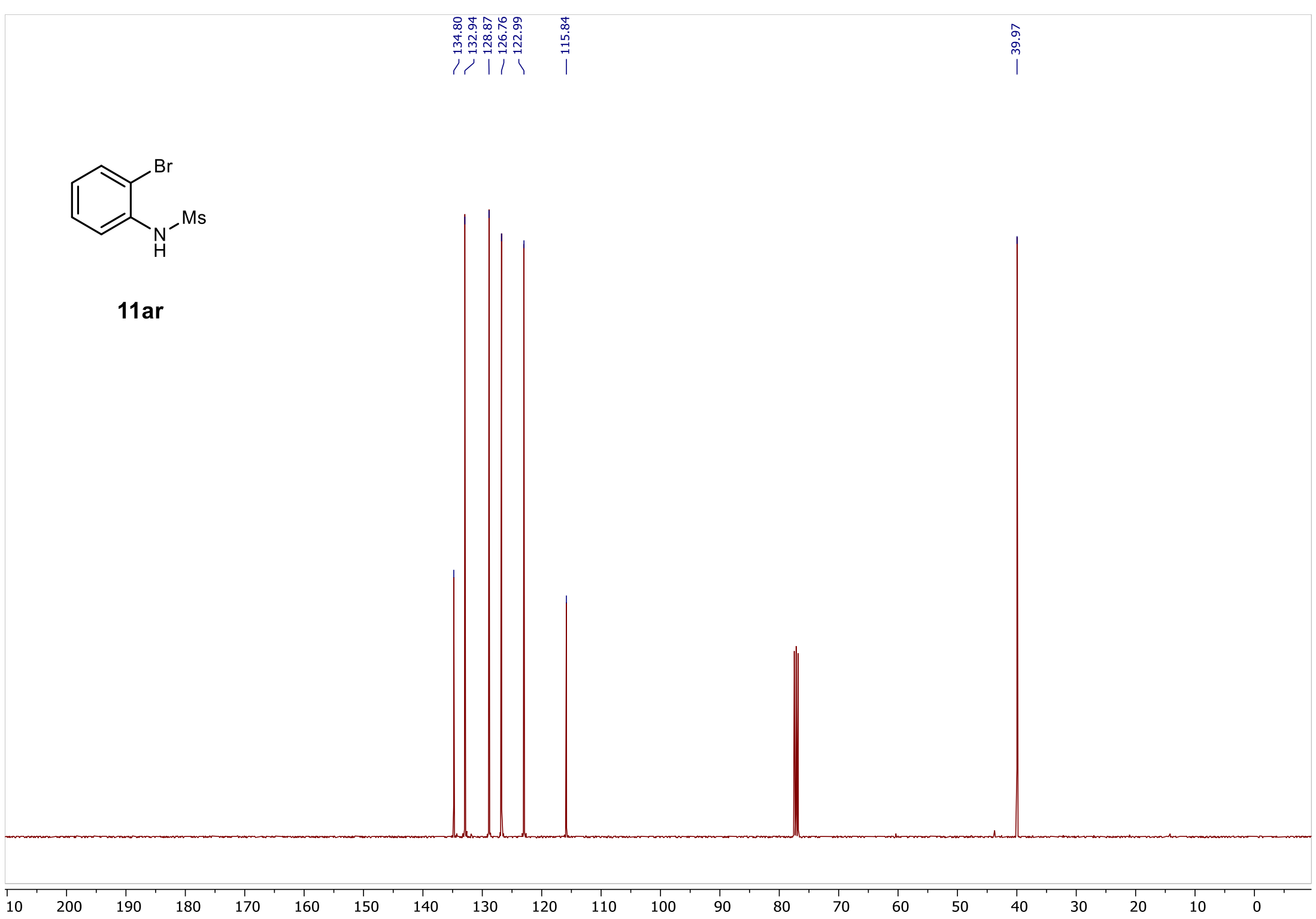

$^{1}$H NMR (400 MHz, $CDCl_3$)

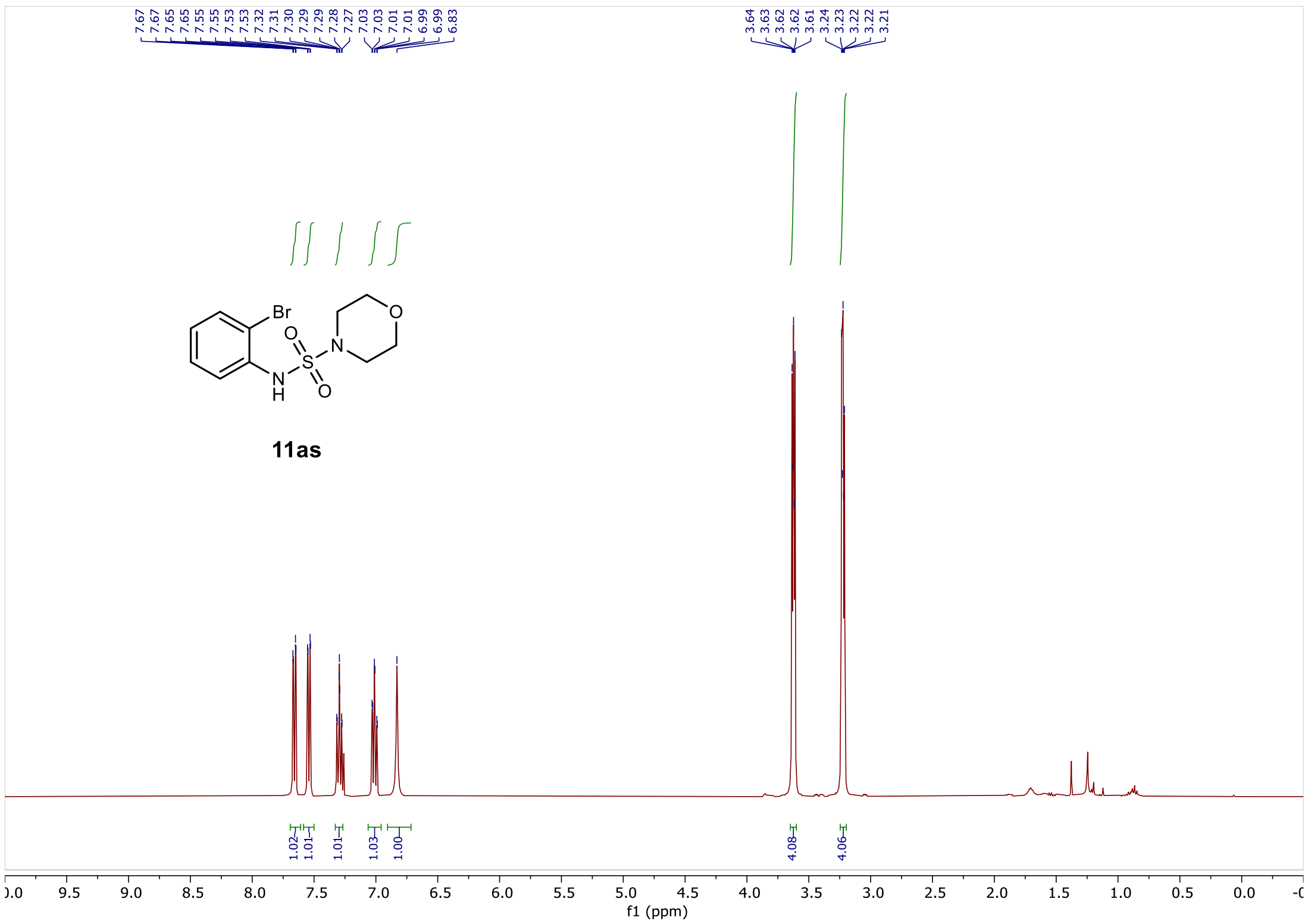

$^{13}$C NMR (101 MHz, $CDCl_3$)

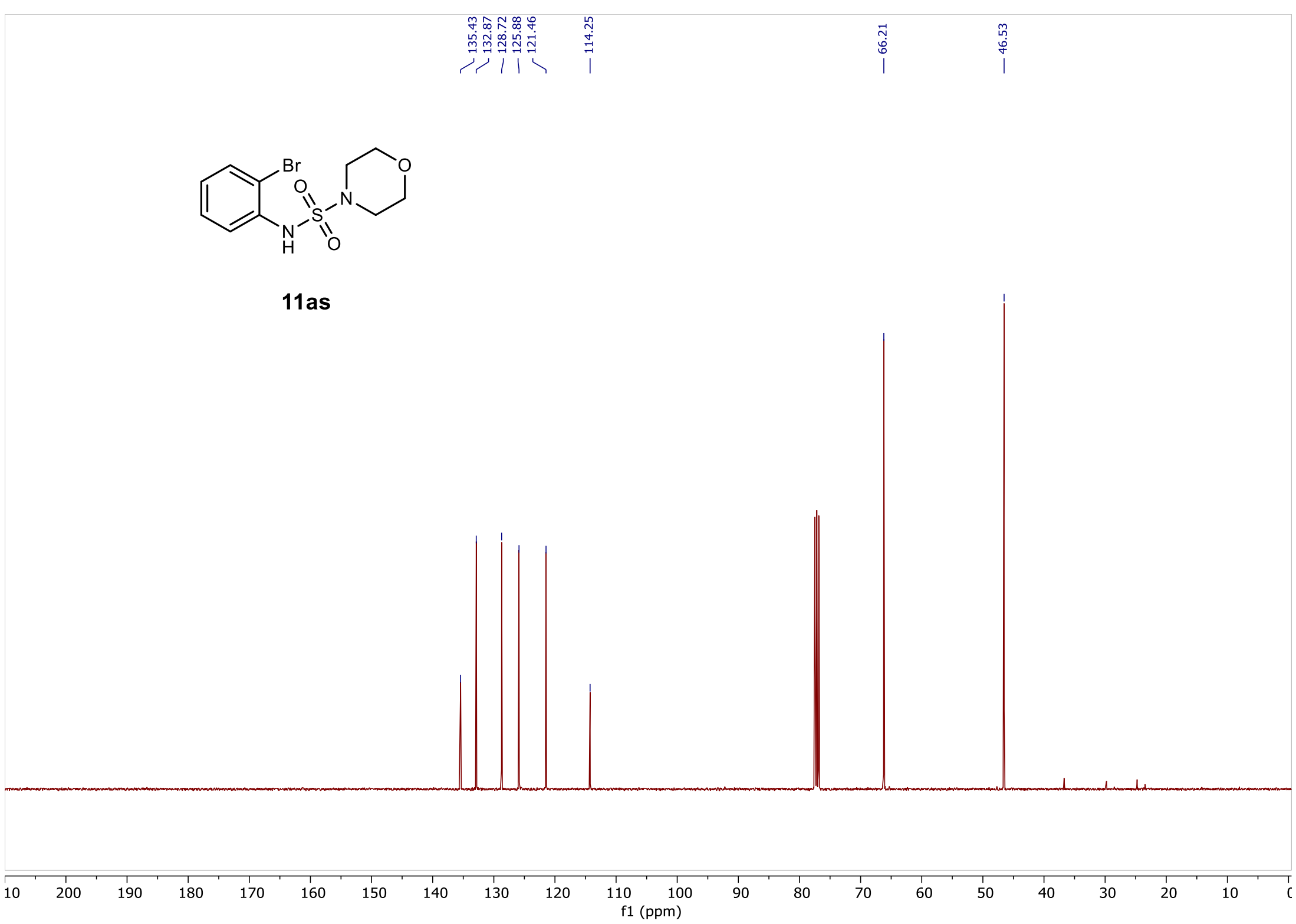

$^1H$ NMR (500 MHz, $CDCl_3$)

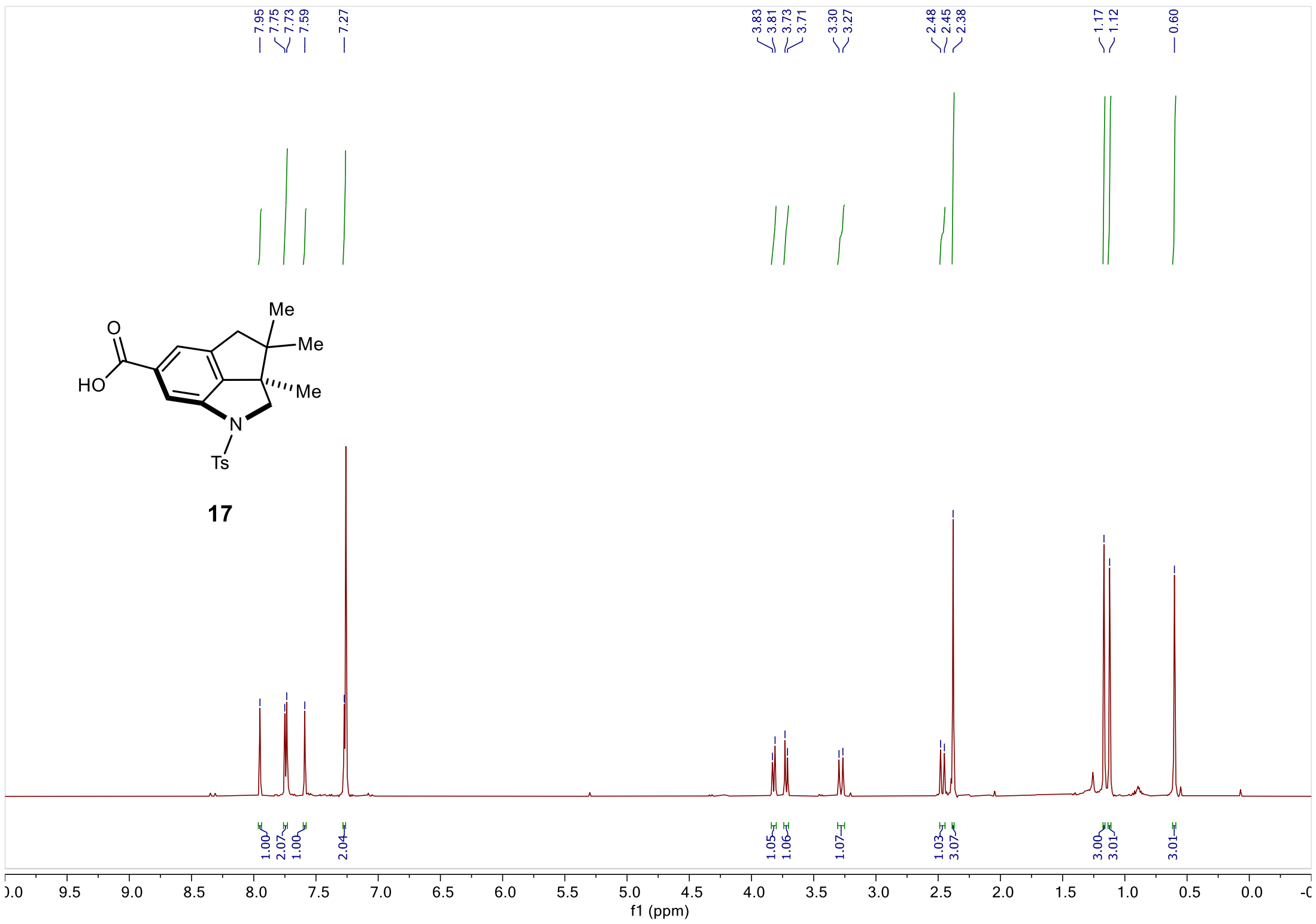

$^{13}C$ NMR (101 MHz, $CDCl_3$)

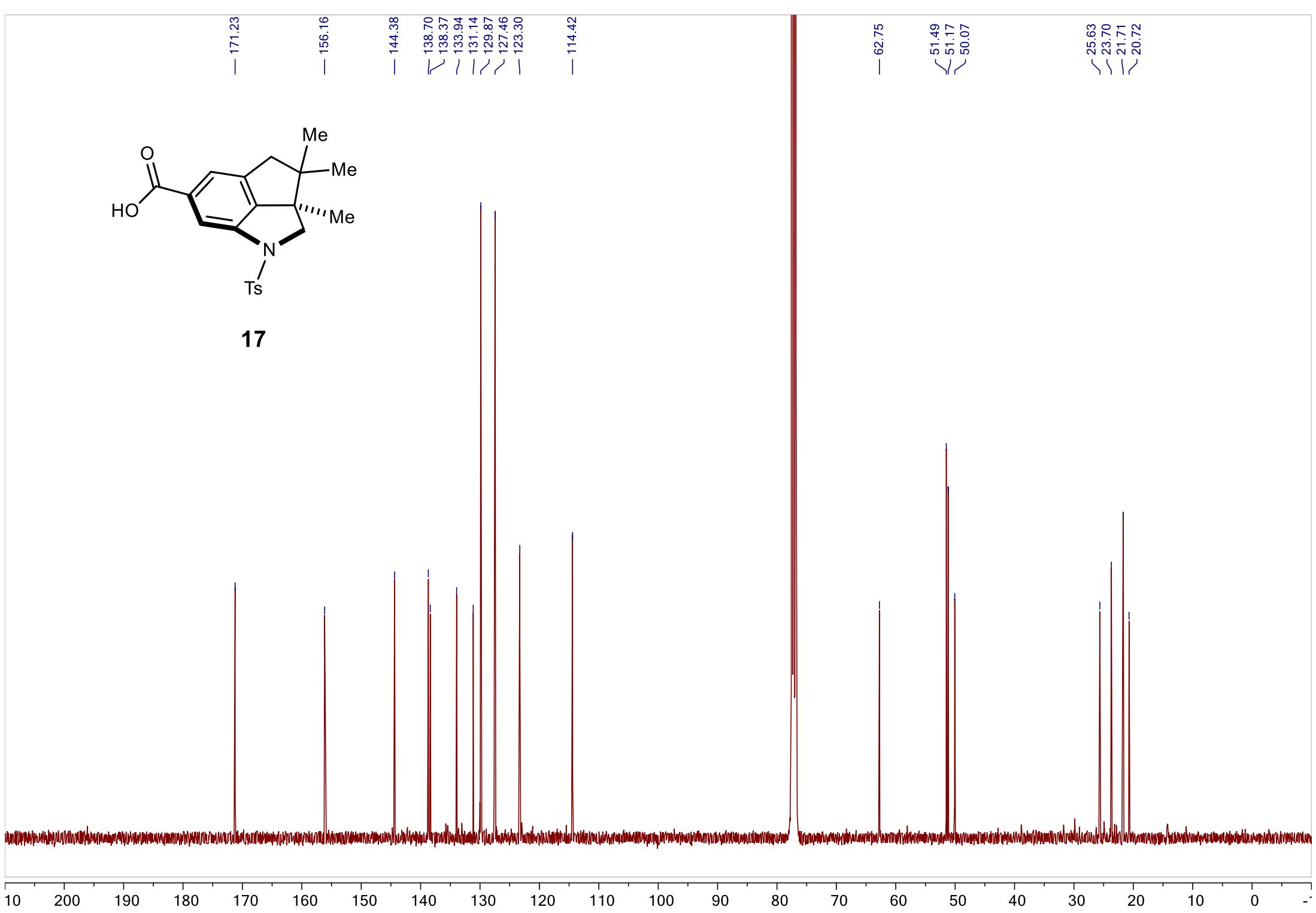

$^{1}H$ NMR (400 MHz, $CDCl_3$)

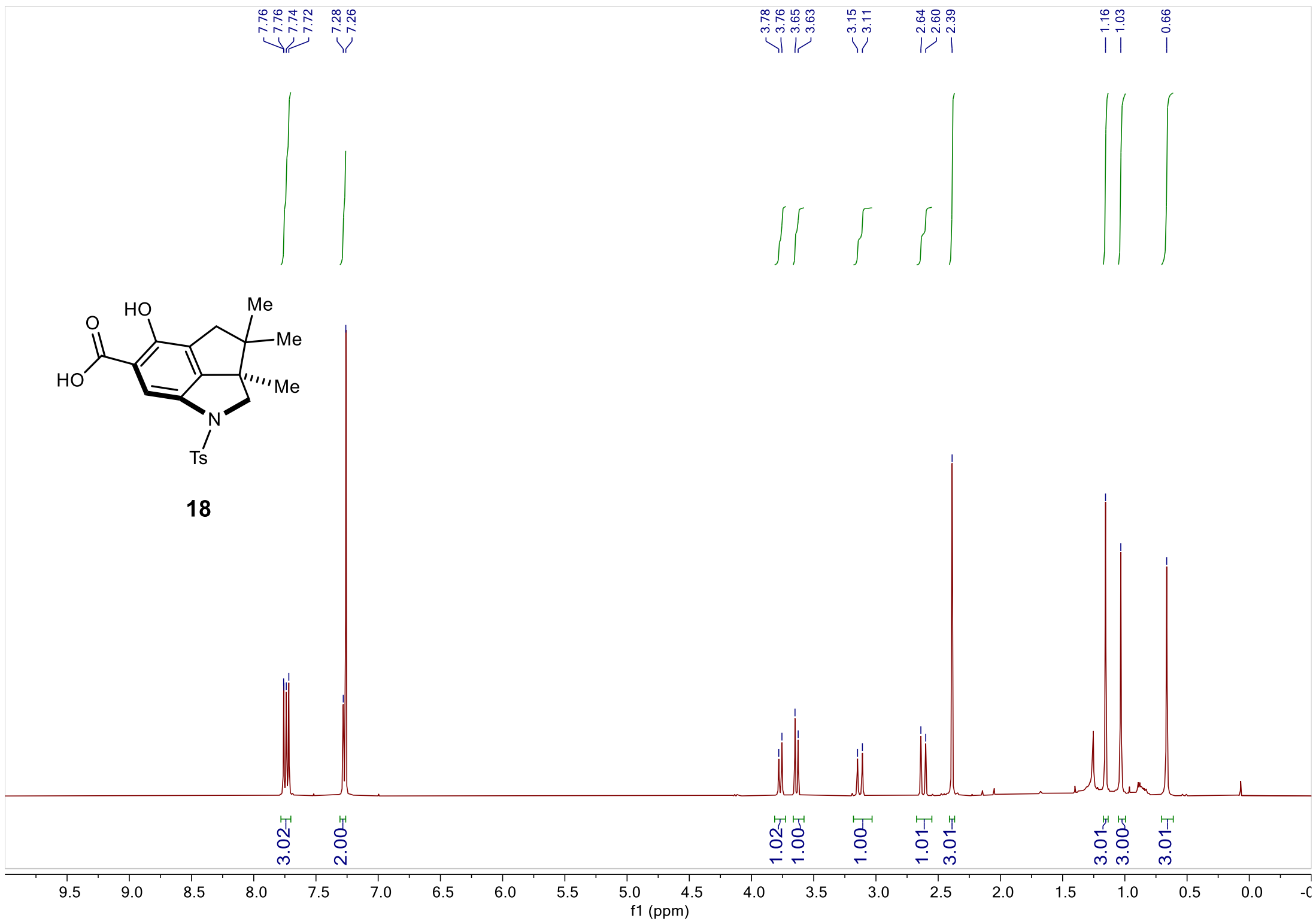

$^{13}C$ NMR (101 MHz, $CDCl_3$)

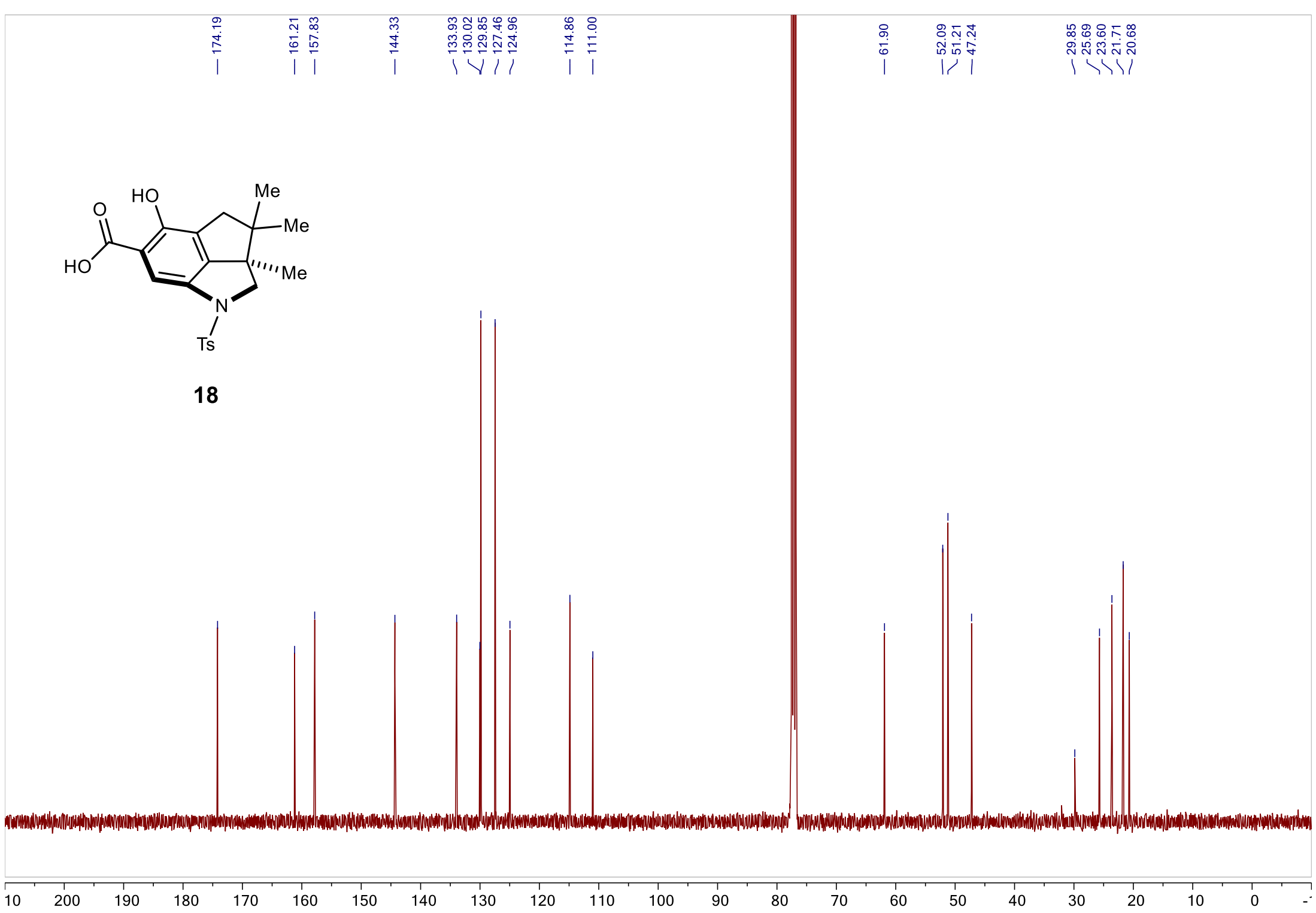

$^{1}H$ NMR (400 MHz, $CDCl_3$)

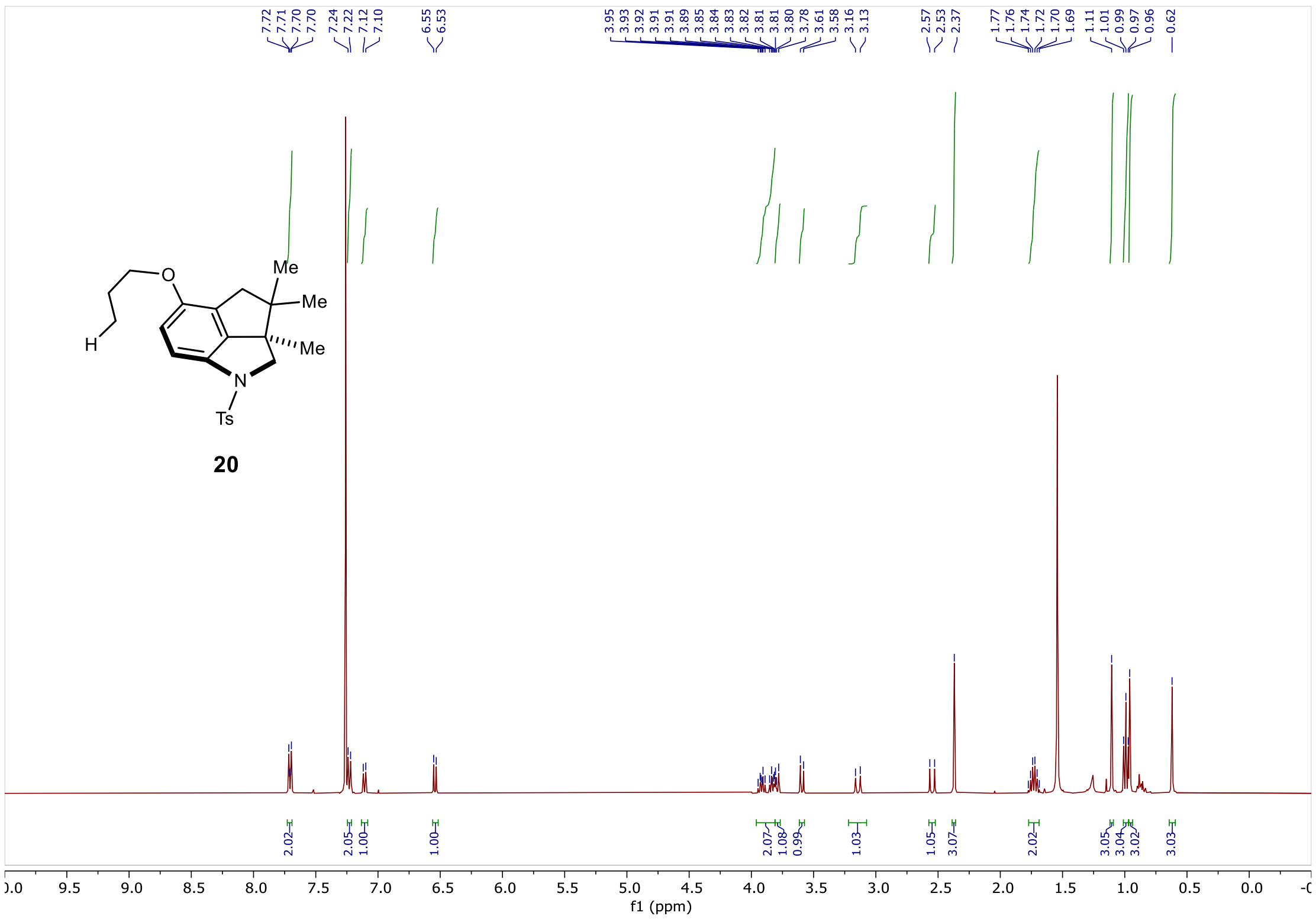

$^{13}C$ NMR (101 MHz, $CDCl_3$)

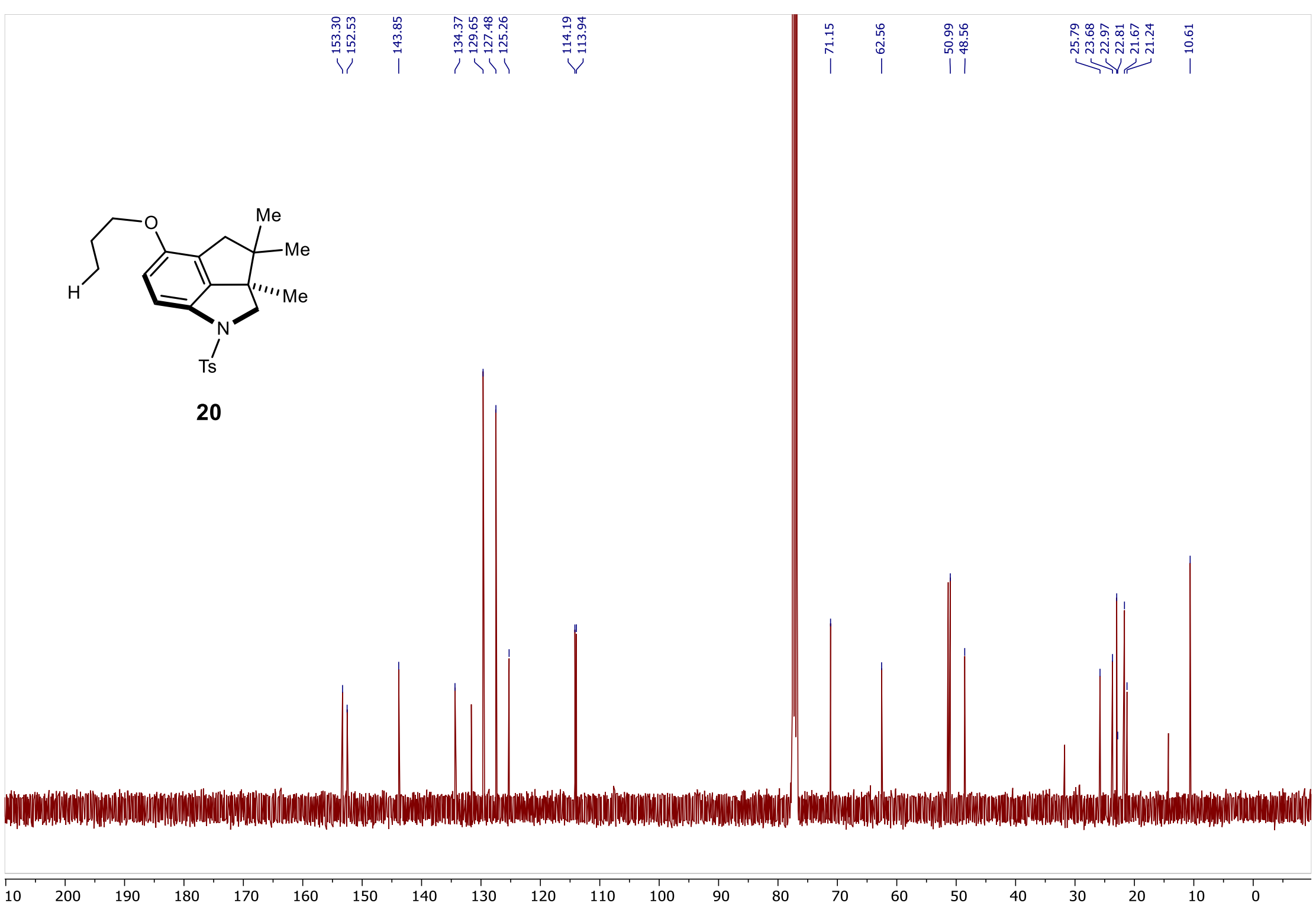

$^{1}H$ NMR (400 MHz, $CDCl_3$)

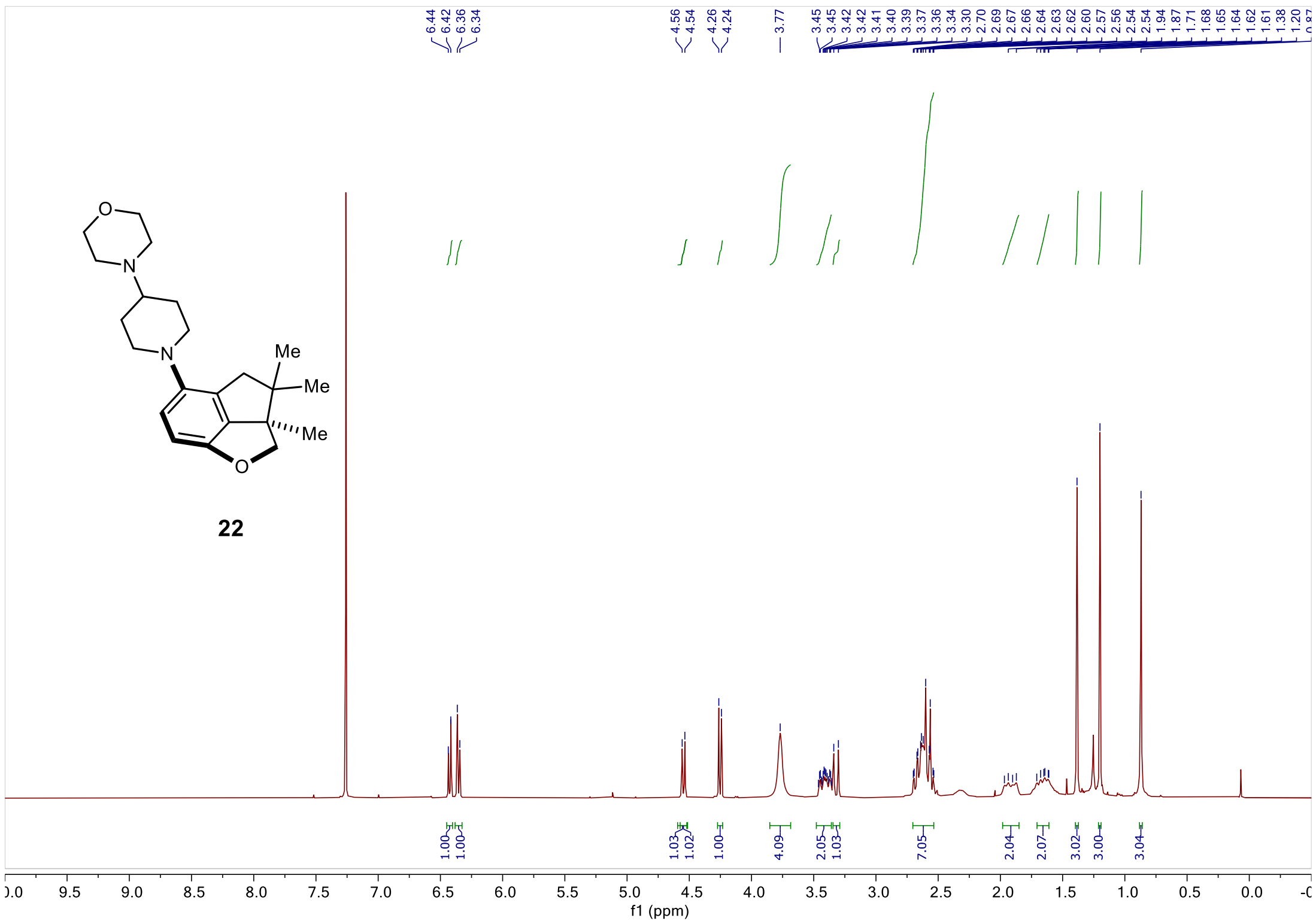


$^{13}C$ NMR (101 MHz, $CDCl_3$)

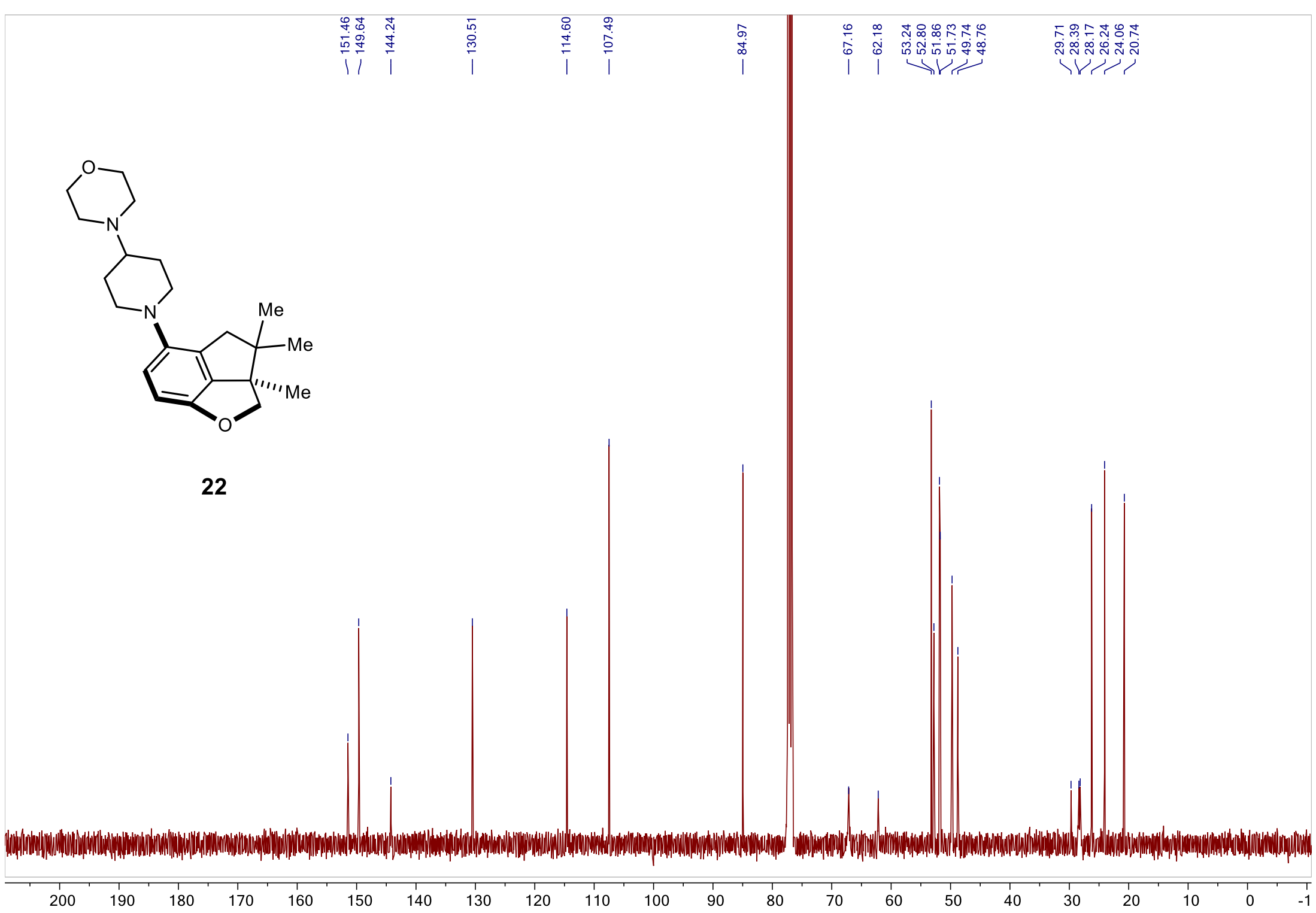

$^{1}H$ NMR (400 MHz, $CDCl_3$)

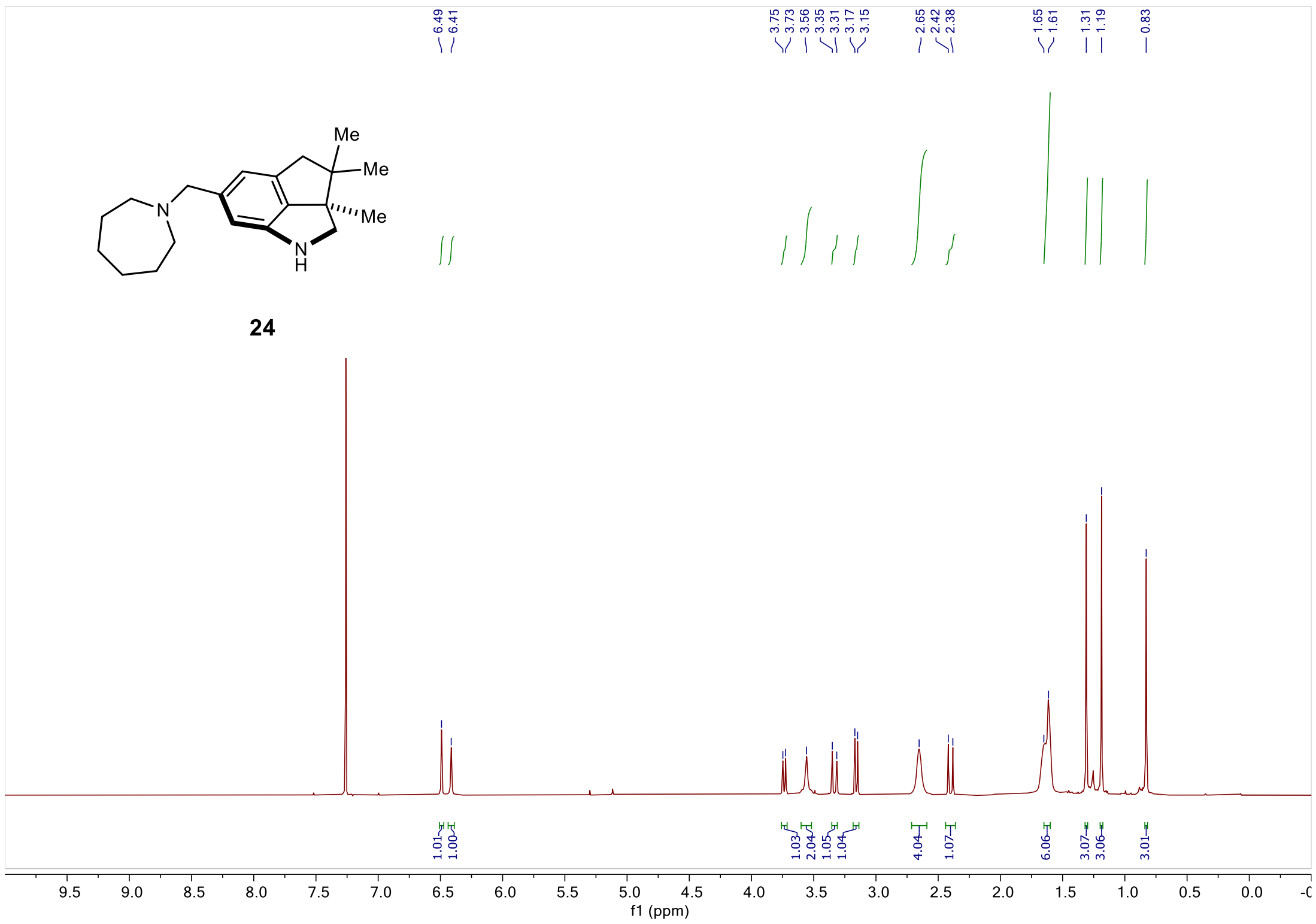


$^{13}C$ NMR (101 MHz, $CDCl_3$)

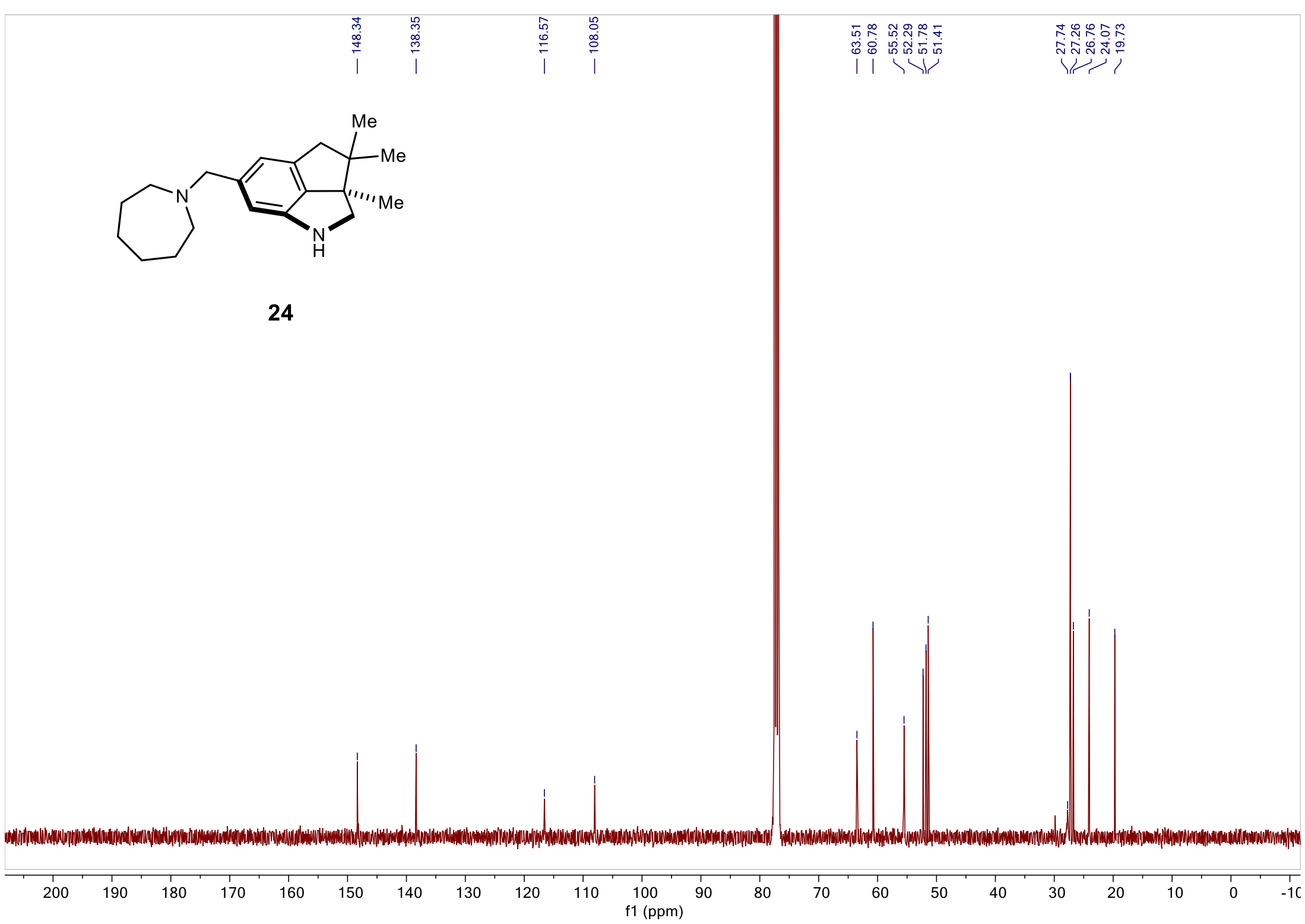

$^1H$ NMR (400 MHz, $CDCl_3$)

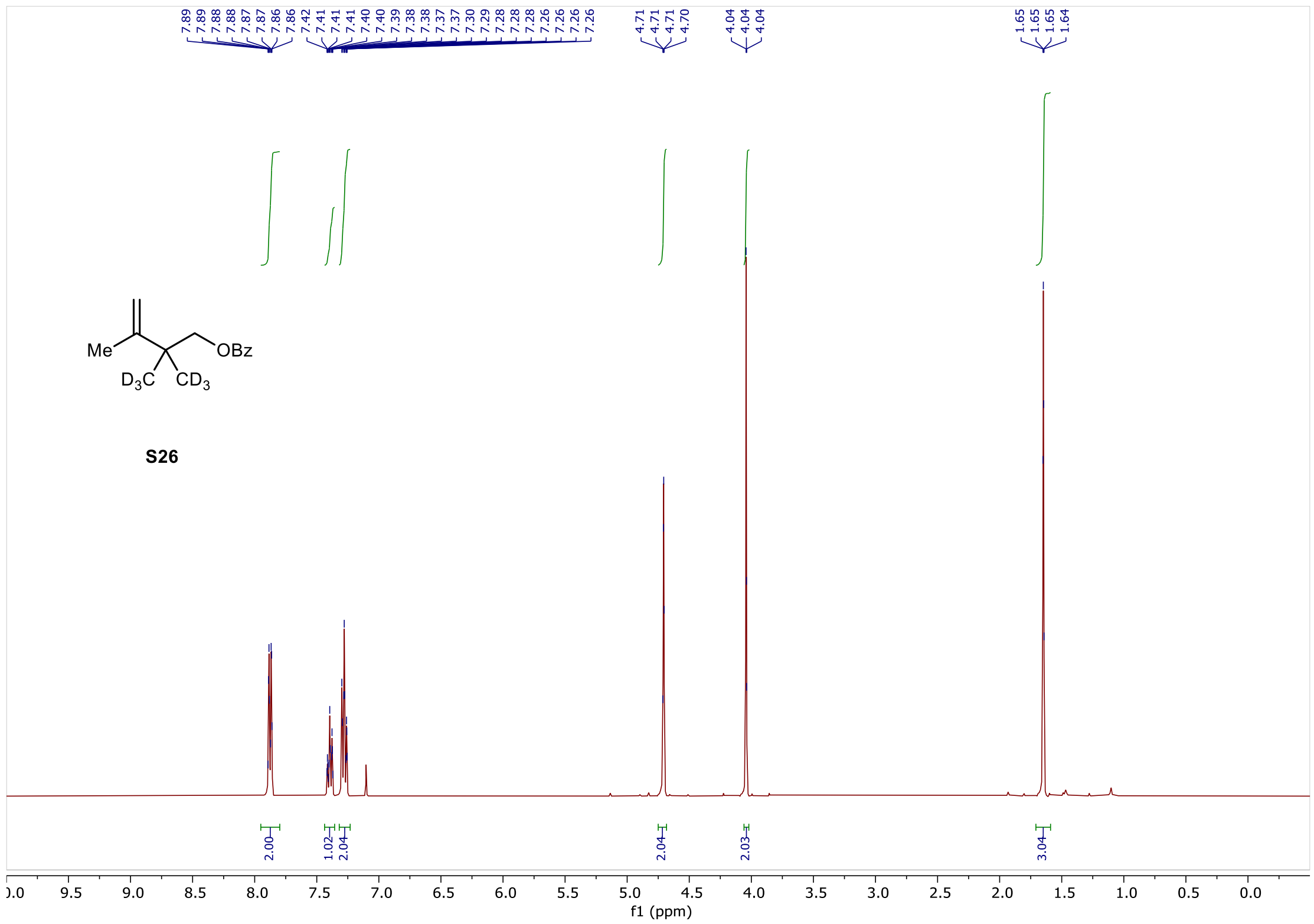

$^{13}C$ NMR (101 MHz, $CDCl_3$)

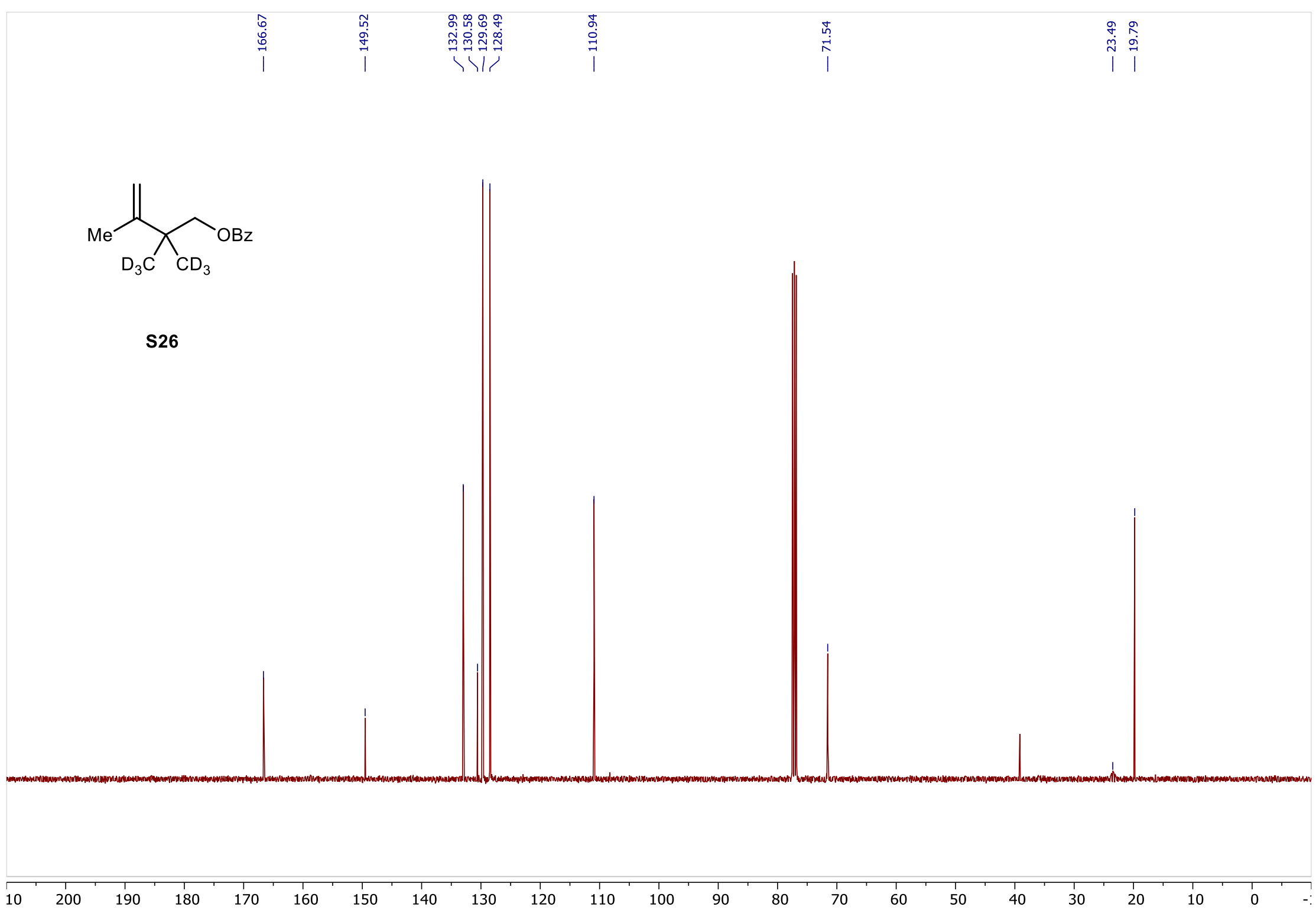

$^{1}H$ NMR (400 MHz, $CDCl_3$)

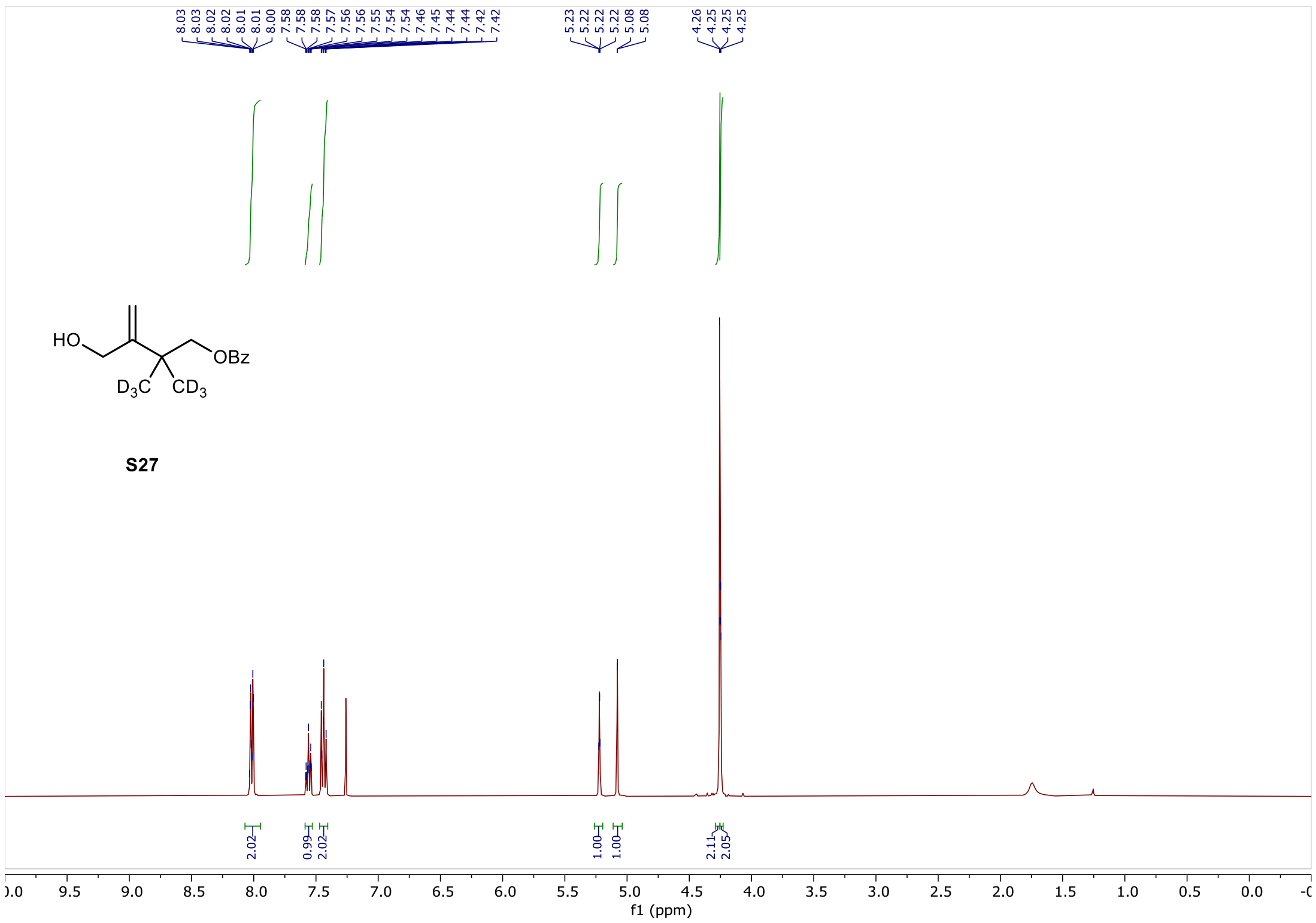


$^{13}C$ NMR (101 MHz, $CDCl_3$)

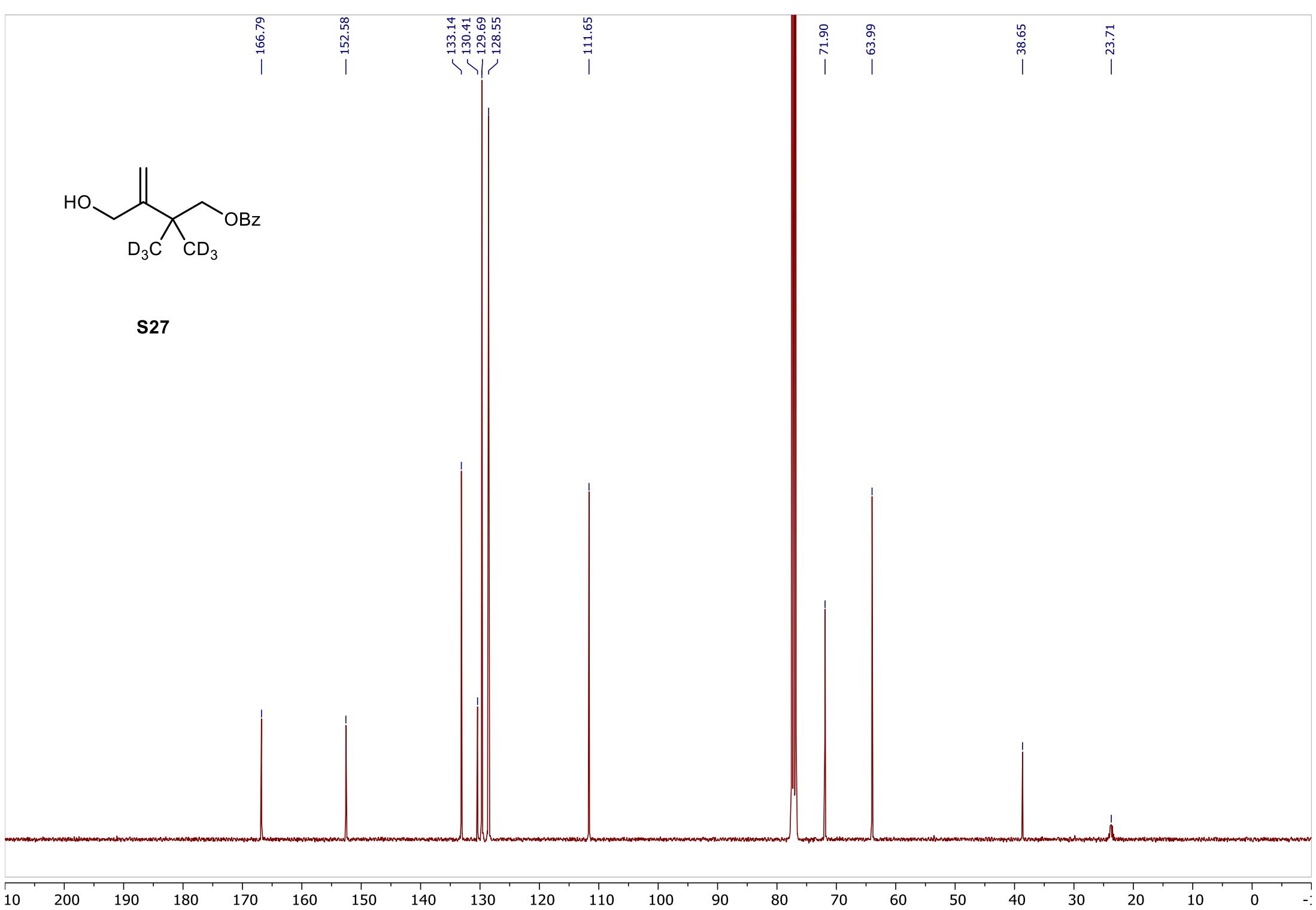

$^{1}$H NMR (500 MHz, $CDCl_3$)

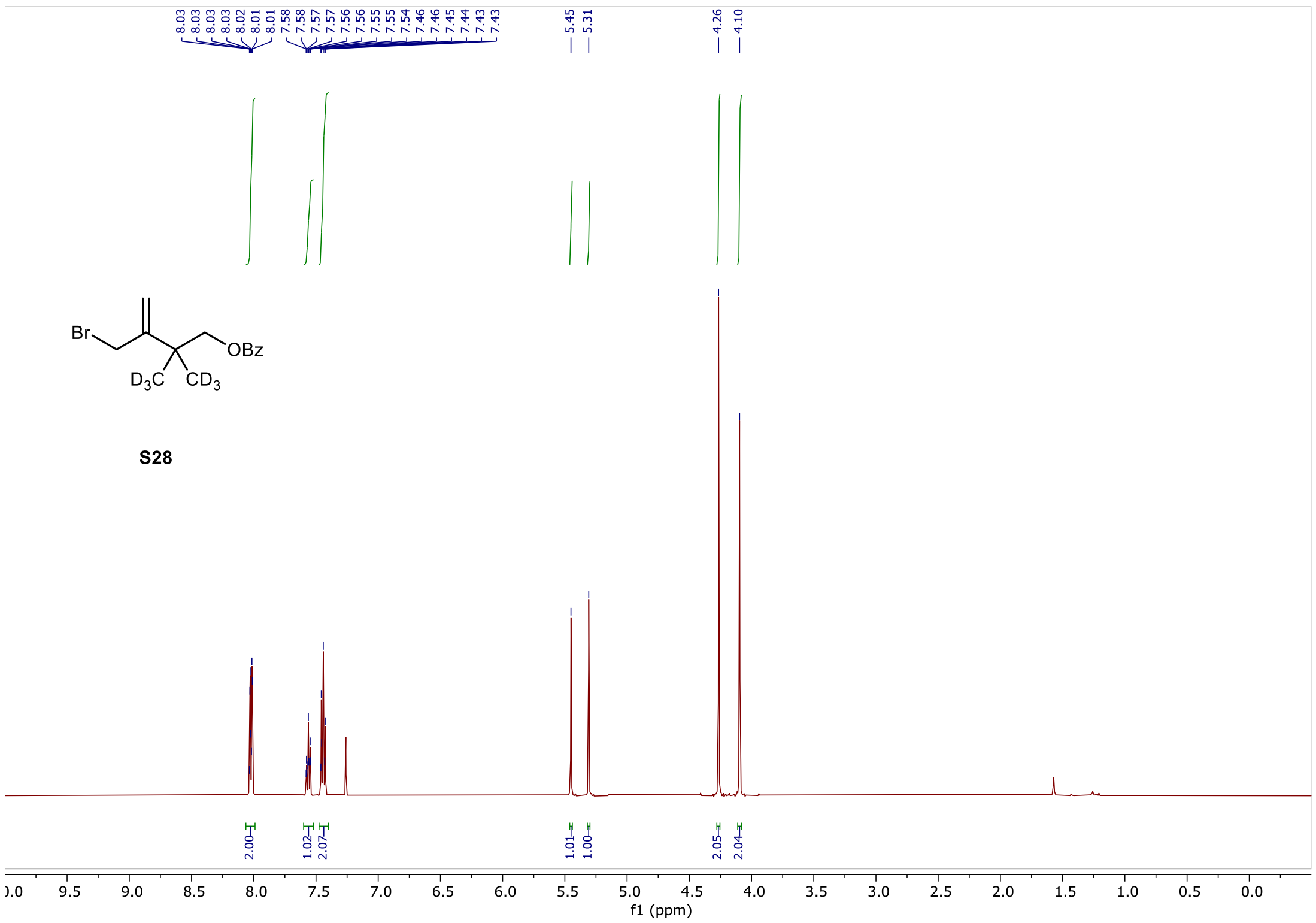

$^{13}$C NMR (126 MHz, $CDCl_3$)

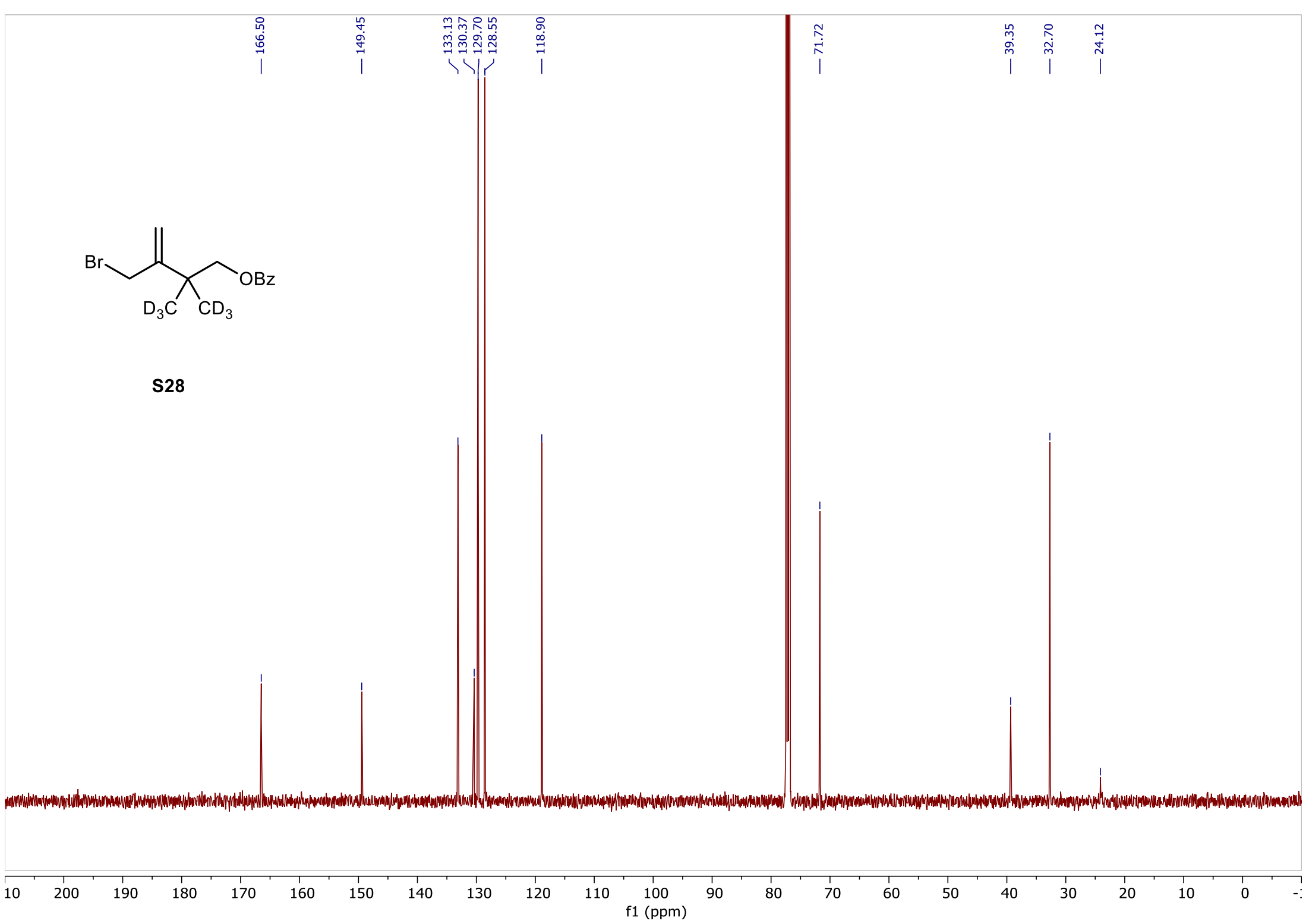

$^{1}H$ NMR (400 MHz, $CDCl_3$)

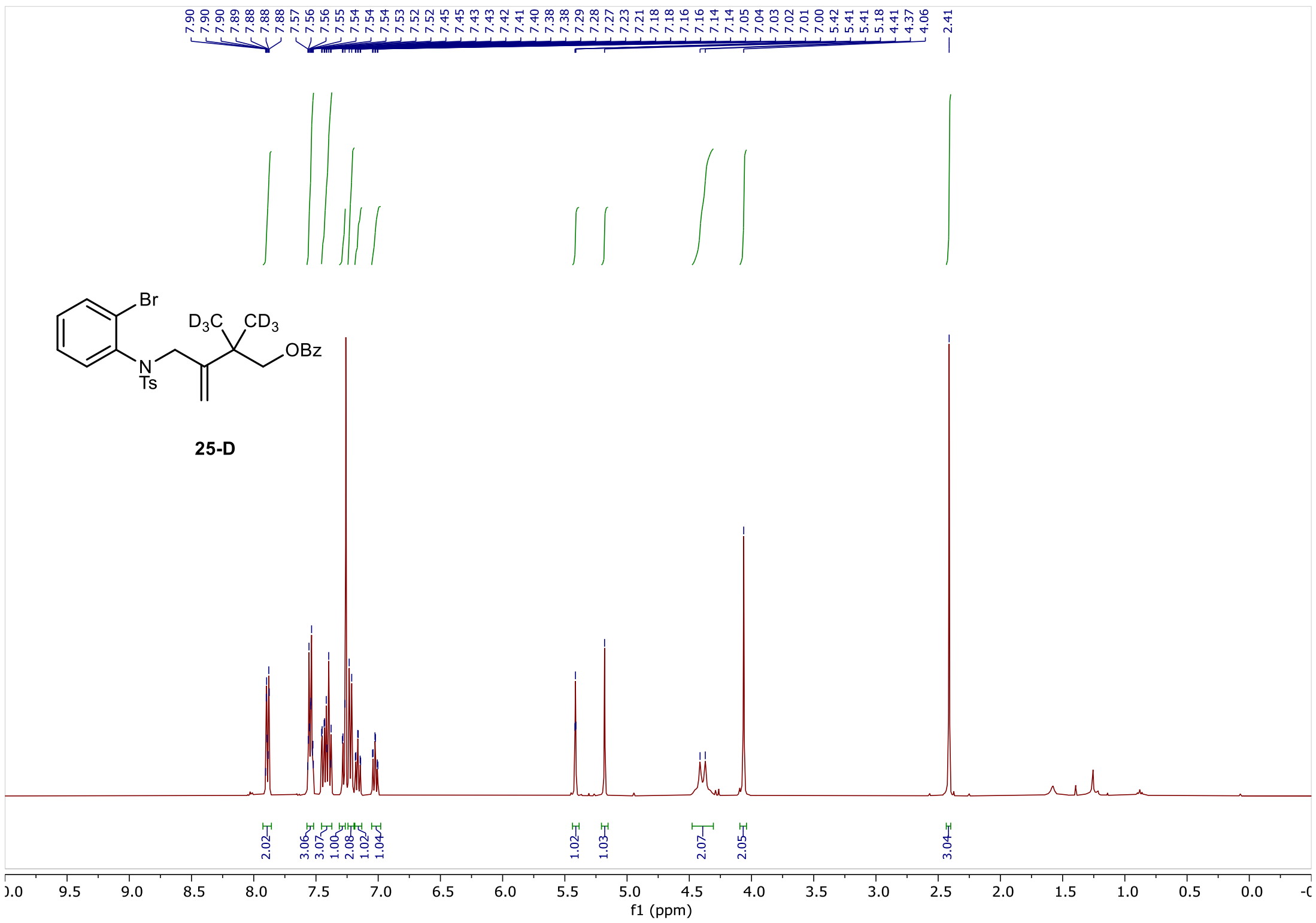

$^{13}C$ NMR (101 MHz, $CDCl_3$)

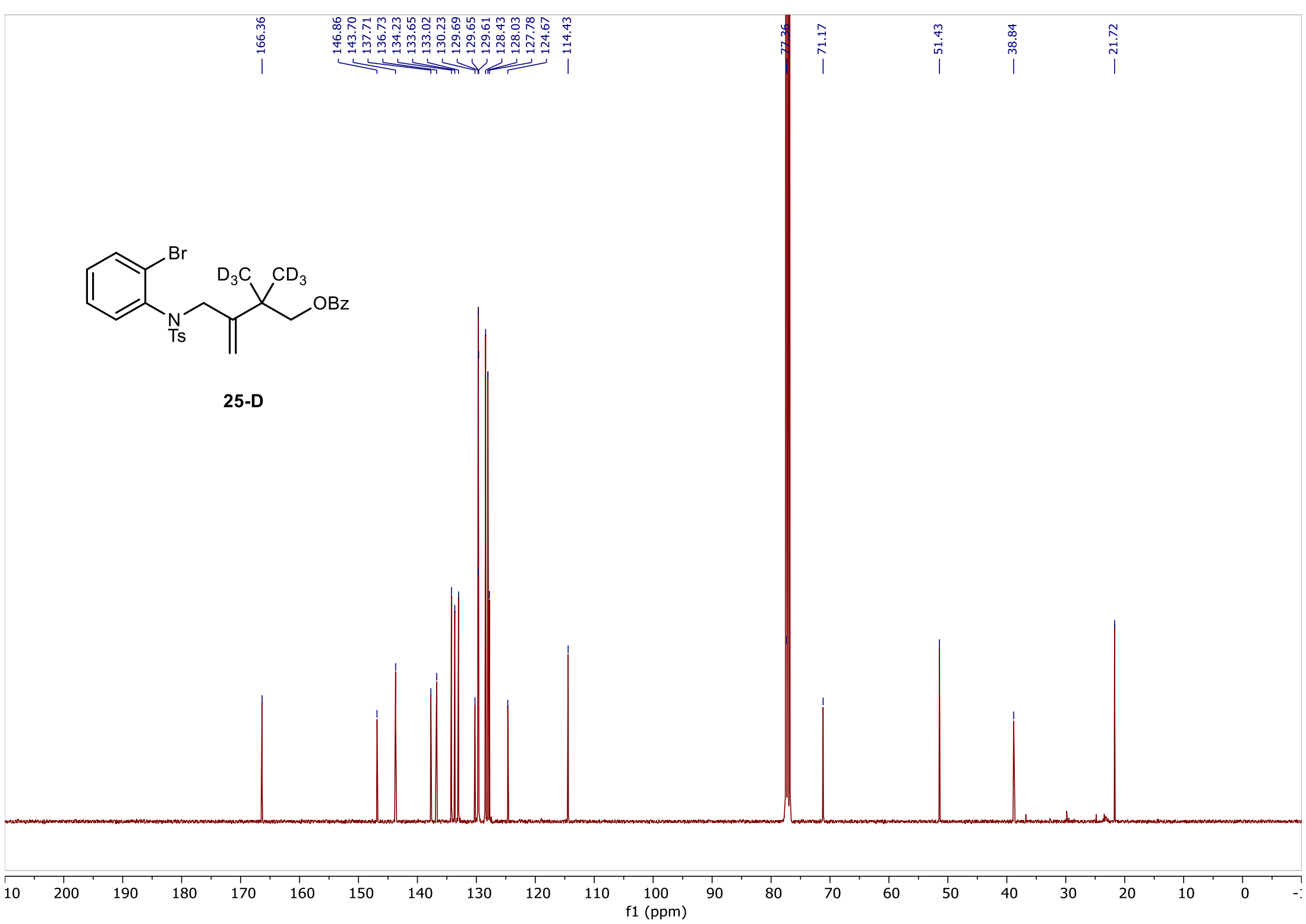

$^{1}H$ NMR (400 MHz, $CDCl_3$)

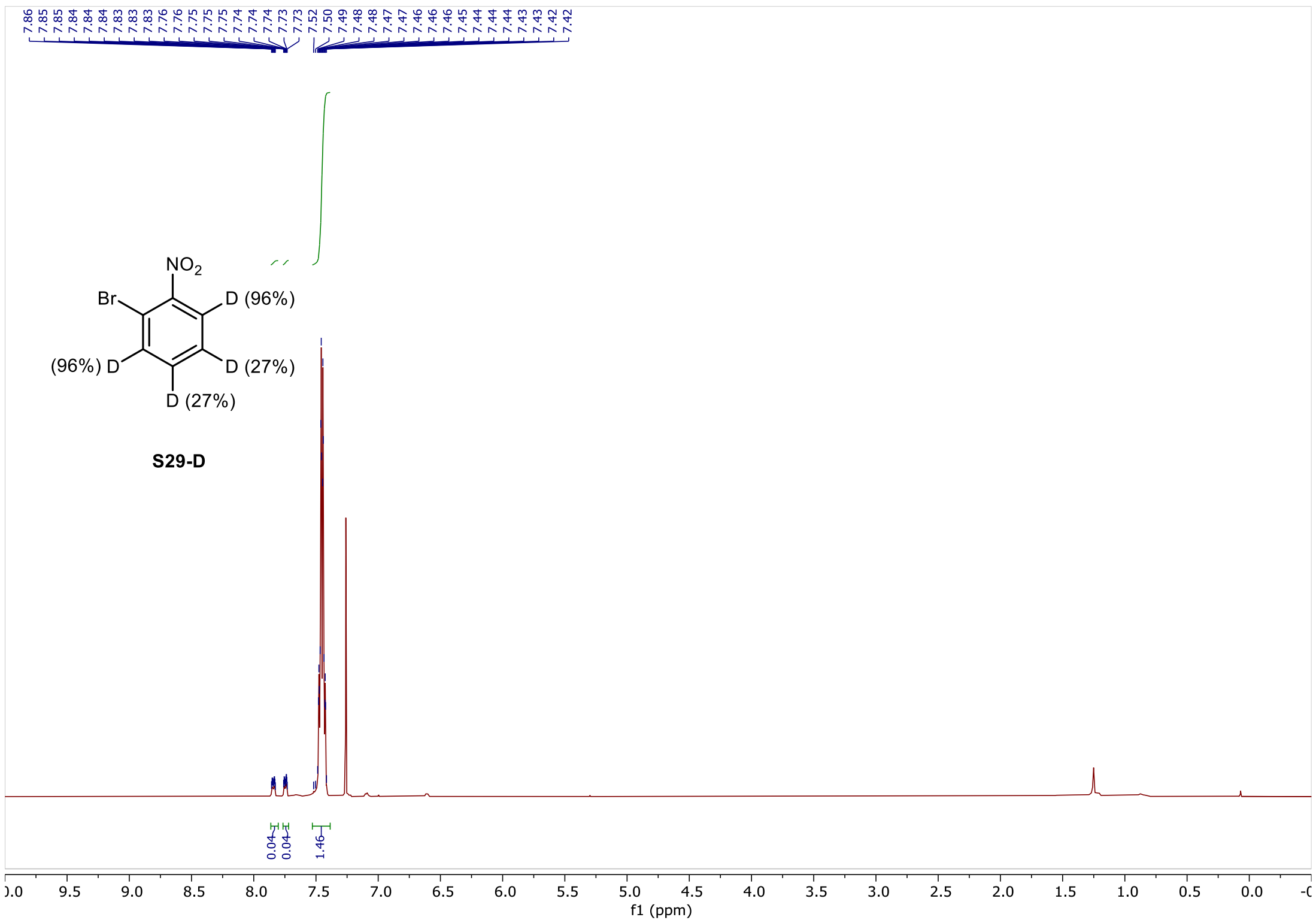

$^{13}C$ NMR (101 MHz, $CDCl_3$)

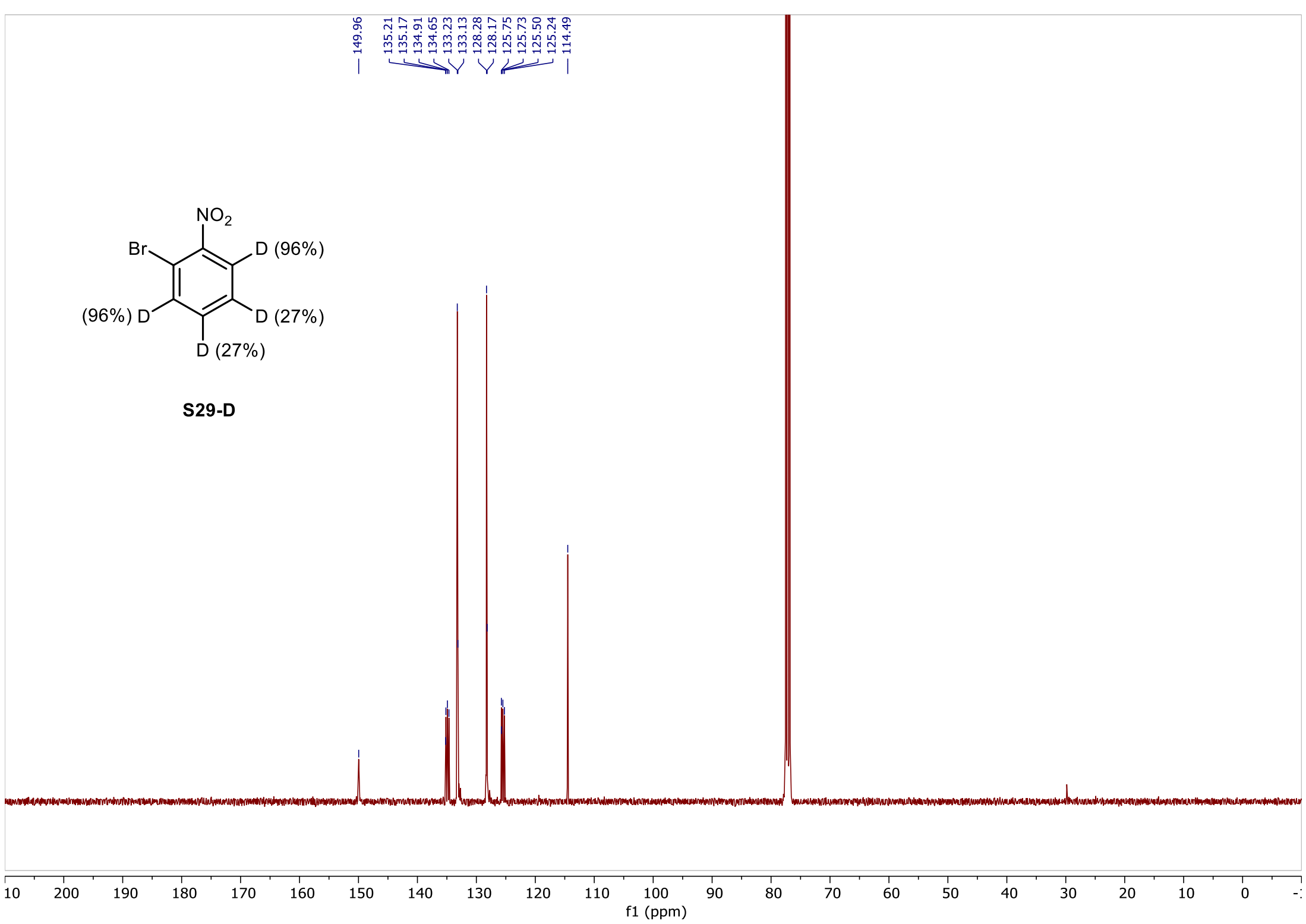

$^1H$ NMR (400 MHz, MeOD)

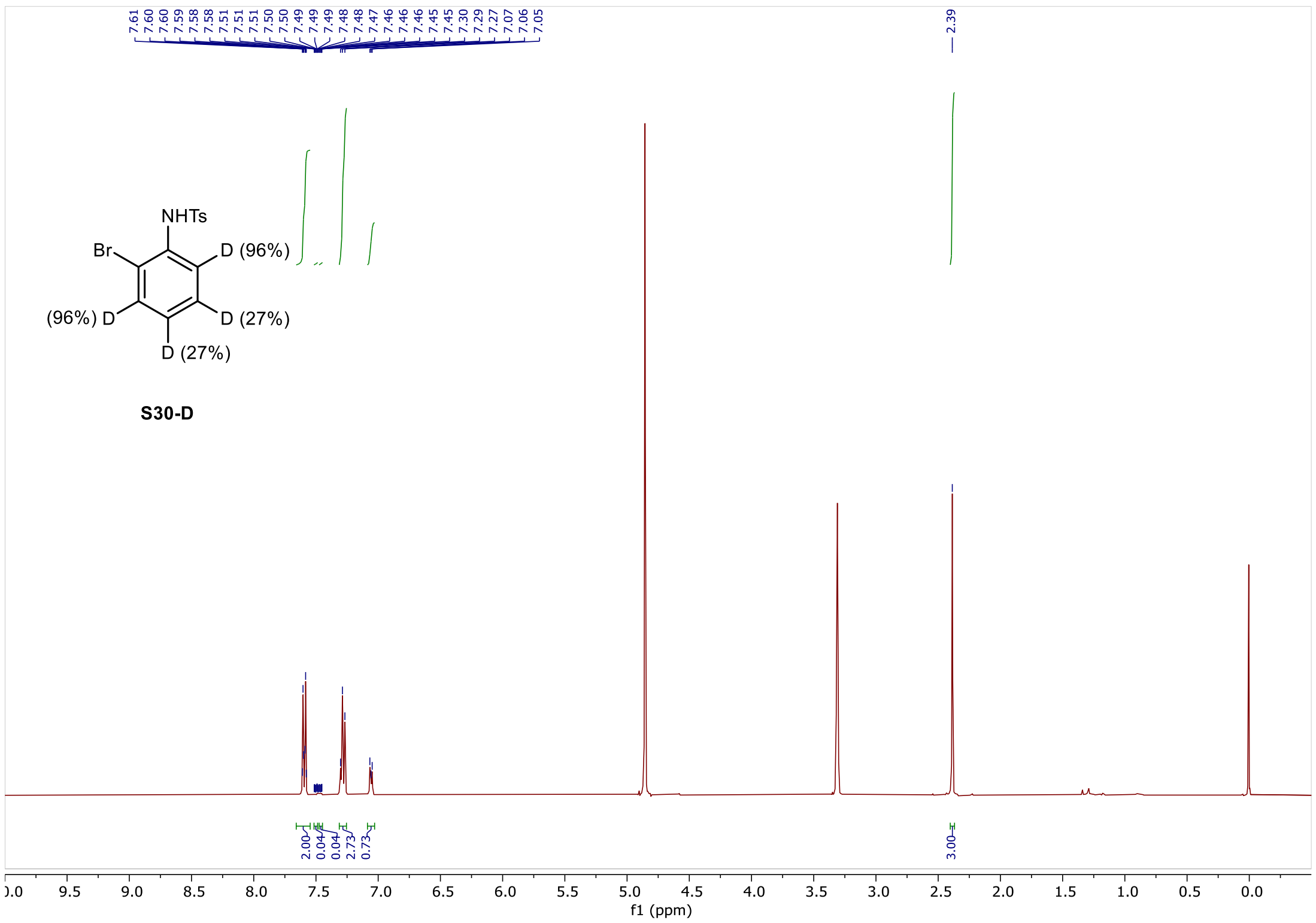


$^{13}C$ NMR (101 MHz, MeOD)

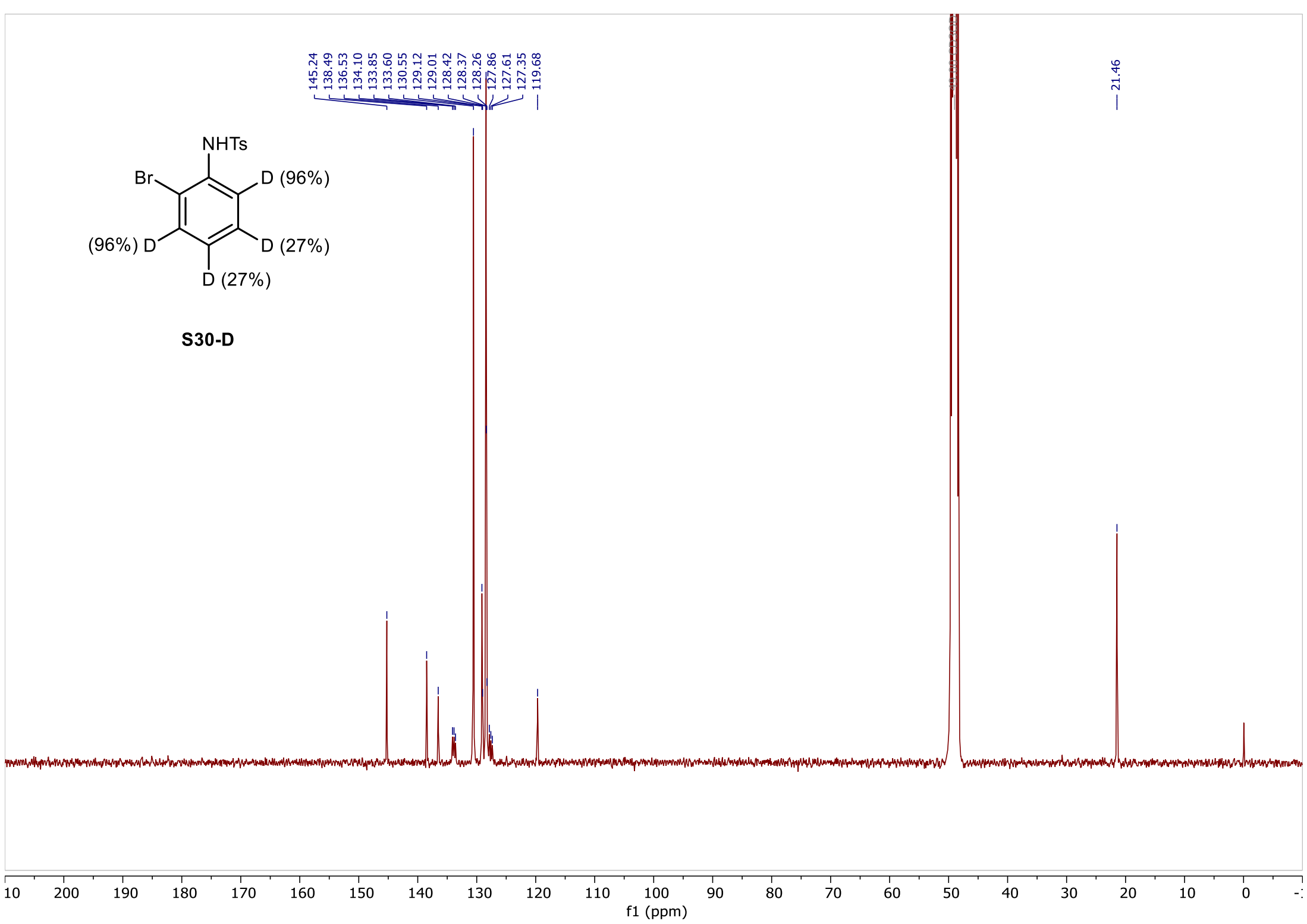

$^{1}$H NMR (400 MHz, $CDCl_3$)

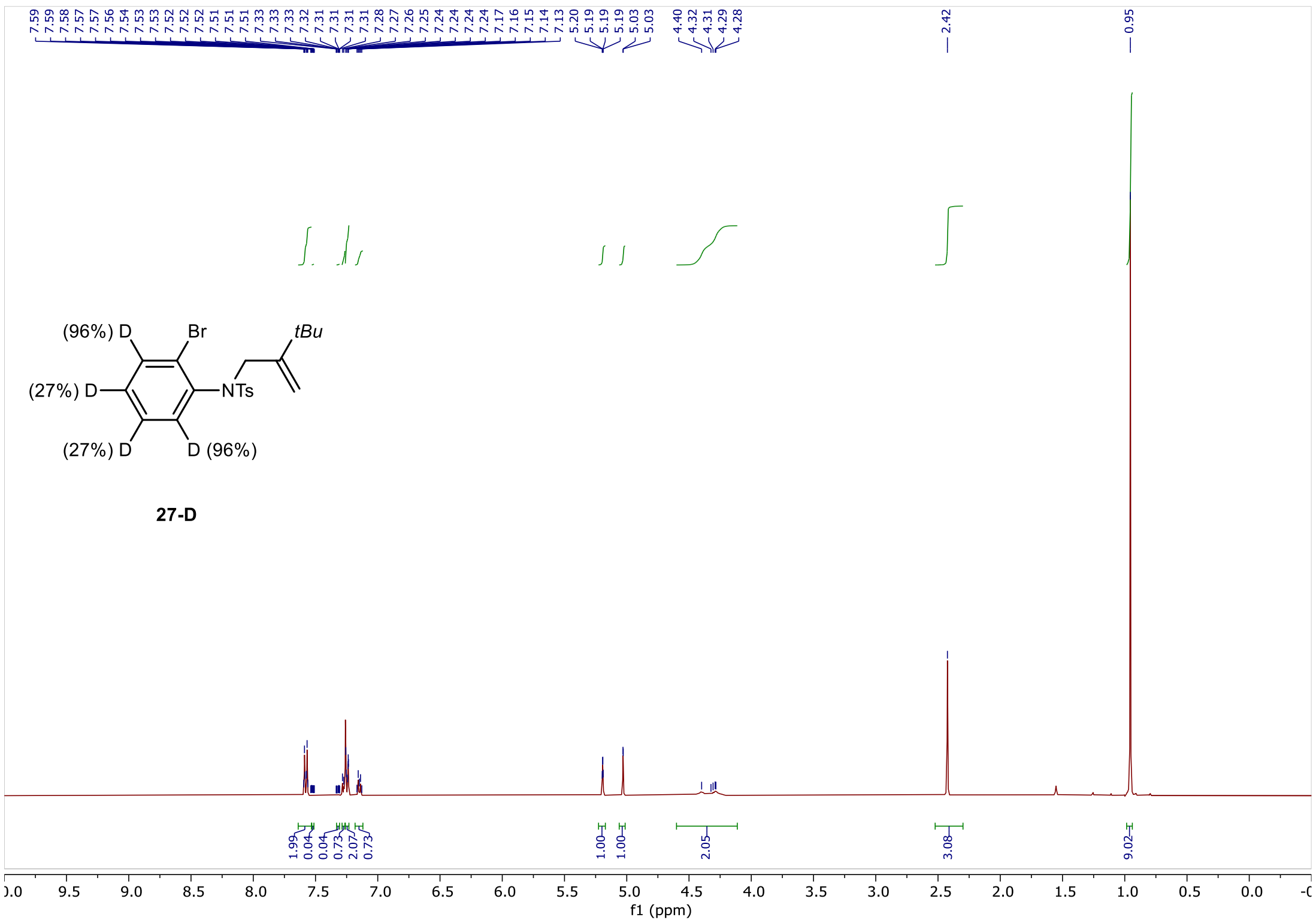


$^{13}$C NMR (101 MHz, $CDCl_3$)

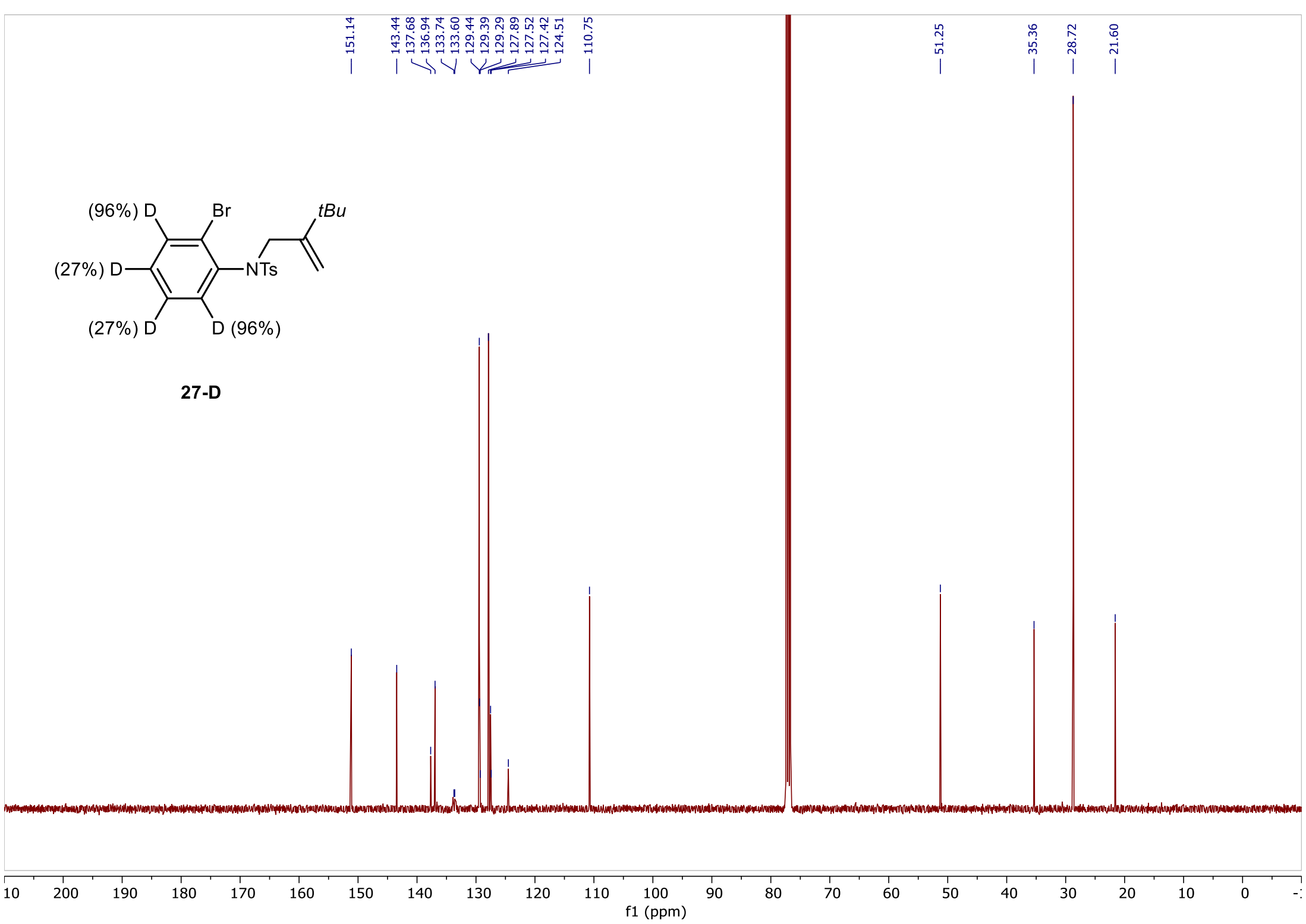

$^{1}H$ NMR (400 MHz, $CDCl_3$)

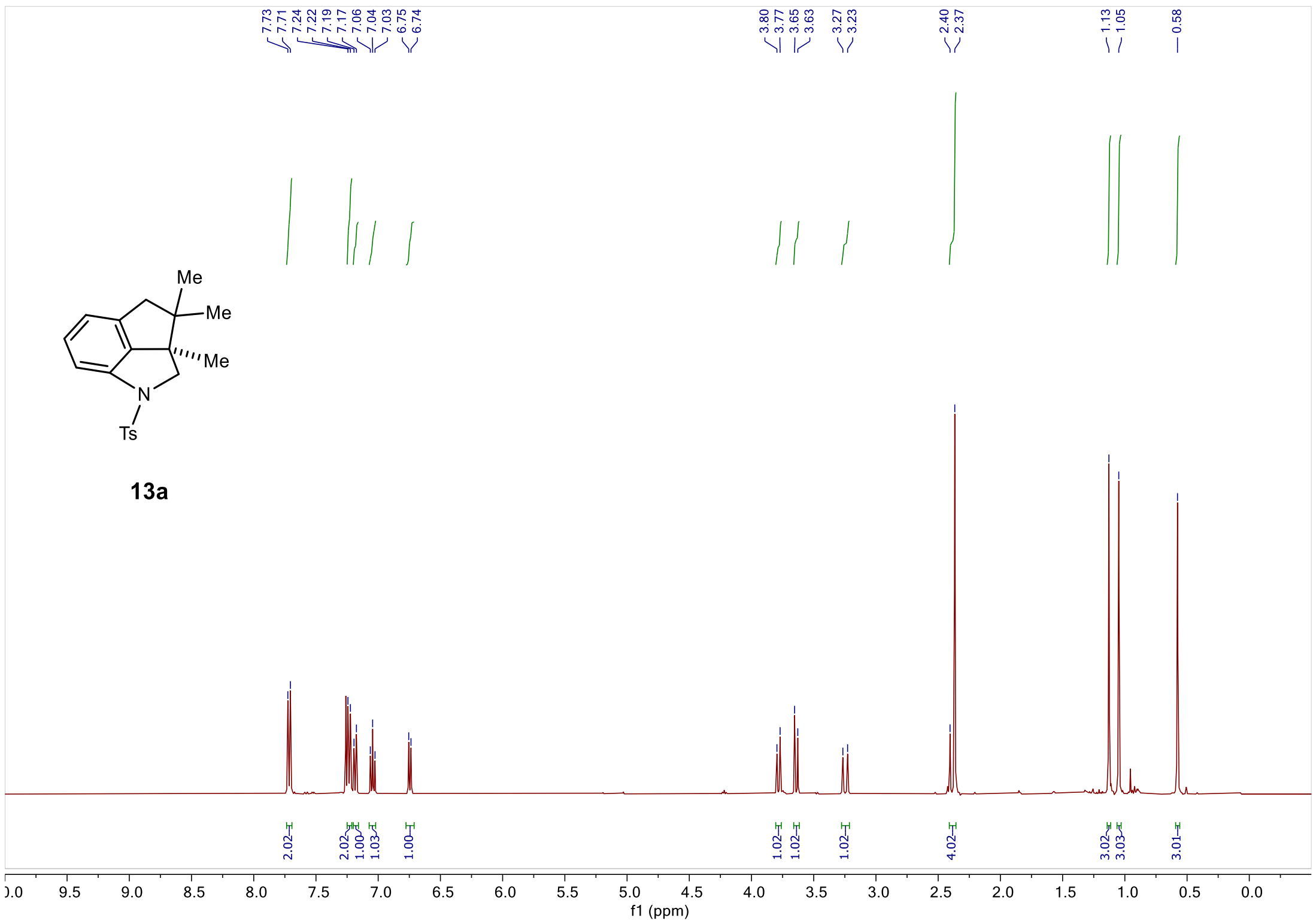

13a

$^{13}C$ NMR (101 MHz, $CDCl_3$)

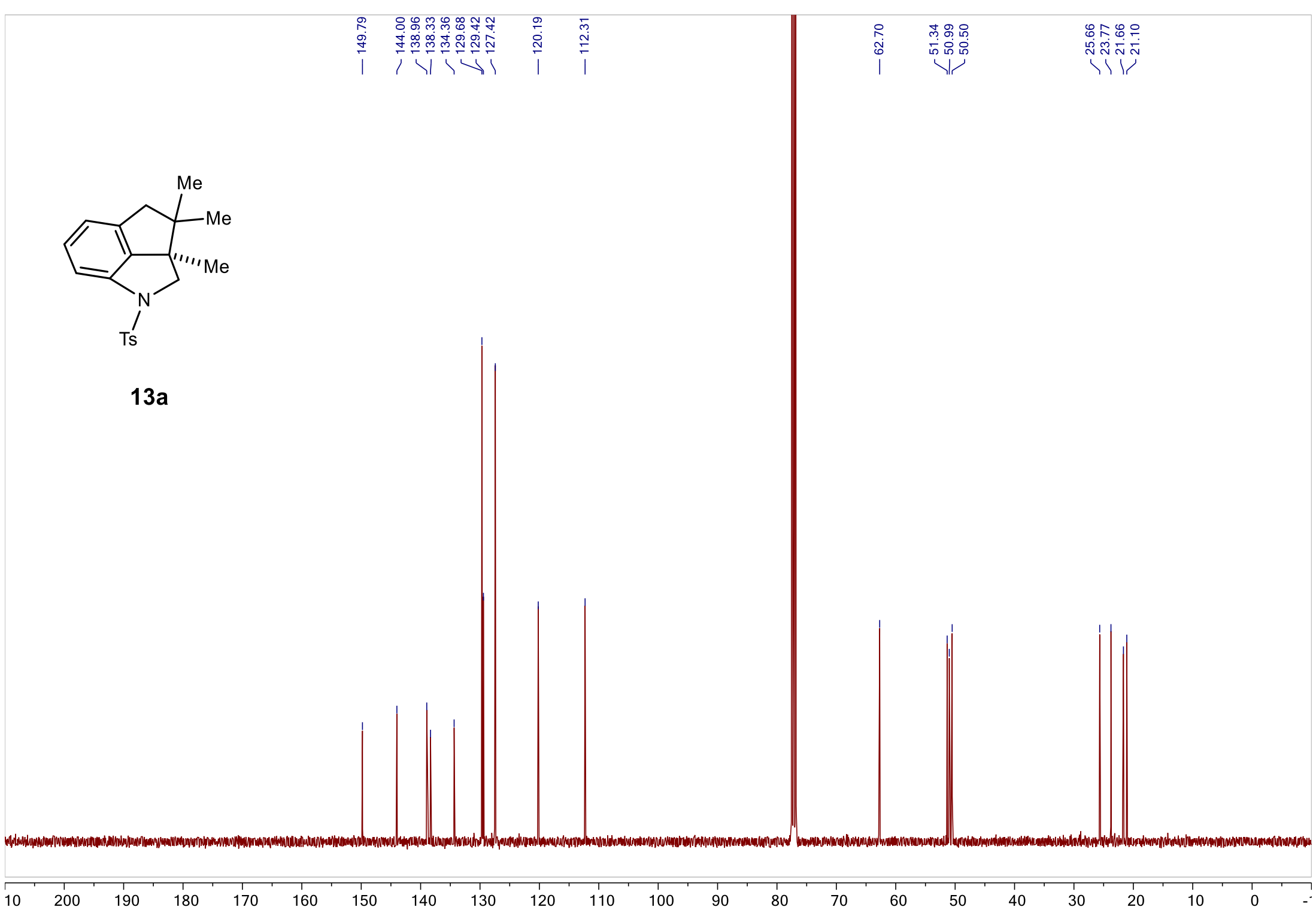

13a

$^{1}H$ NMR (400 MHz, $CDCl_3$)

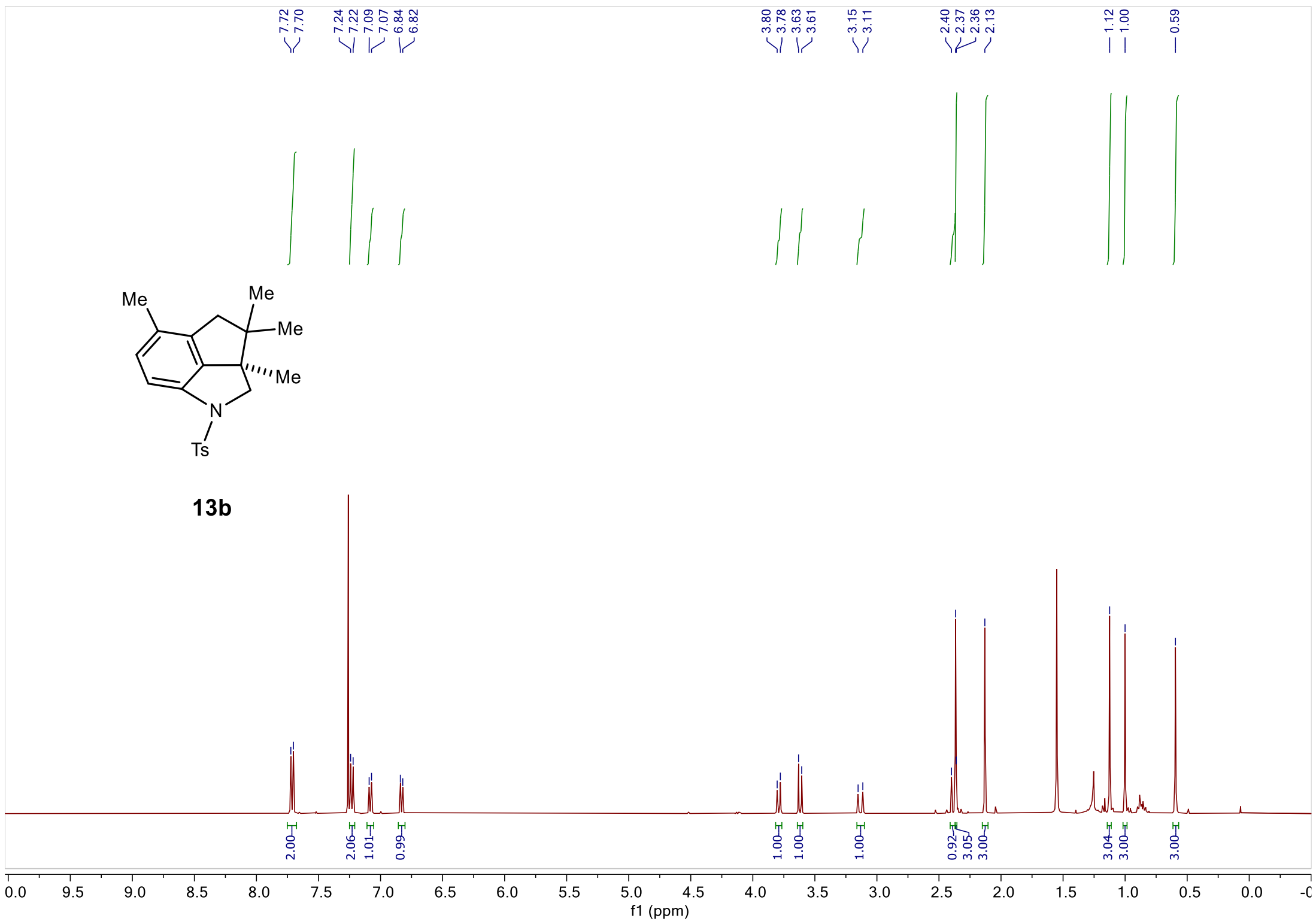


$^{13}C$ NMR (101 MHz, $CDCl_3$)

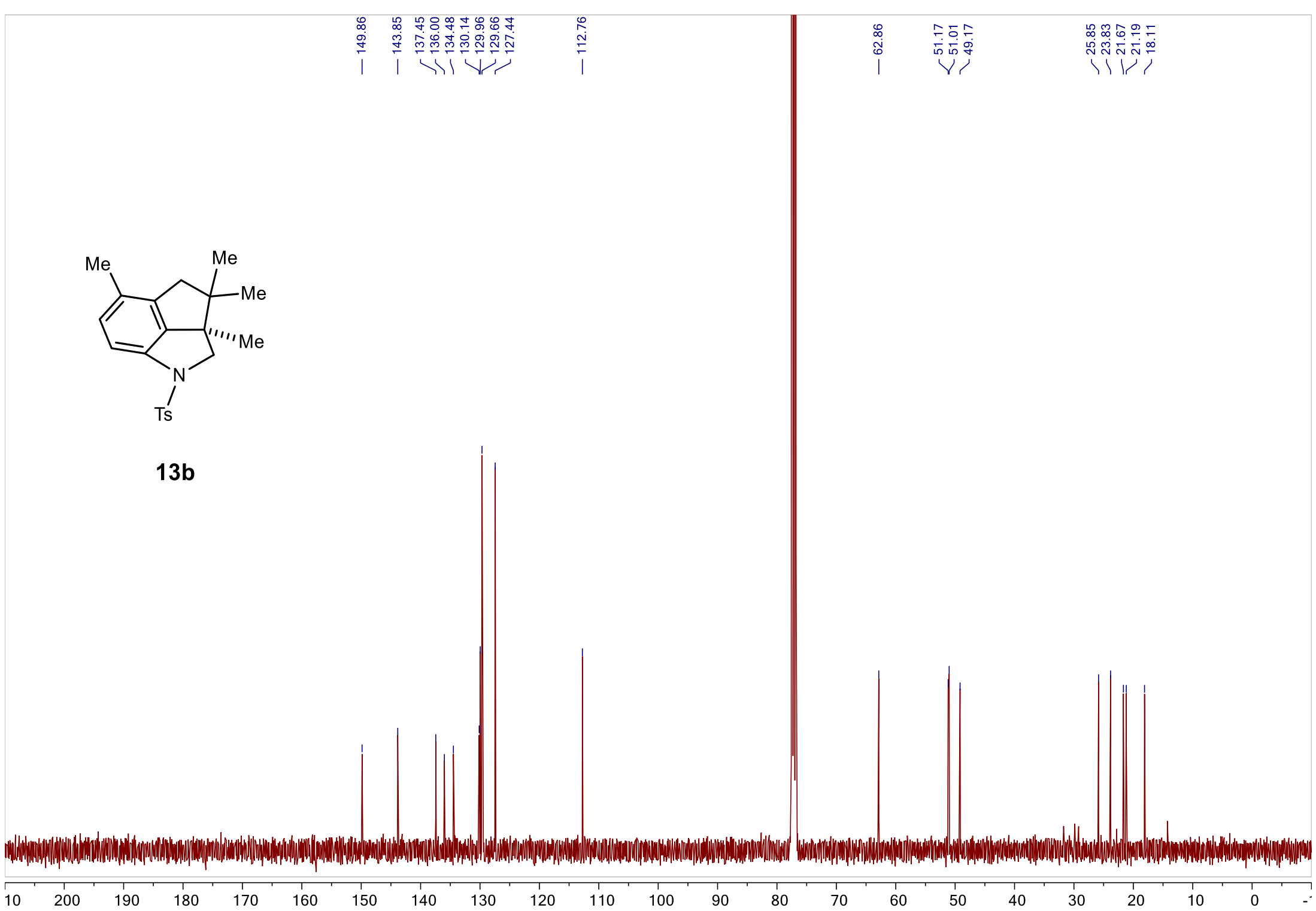

$^{1}H$ NMR (400 MHz, $CDCl_3$)

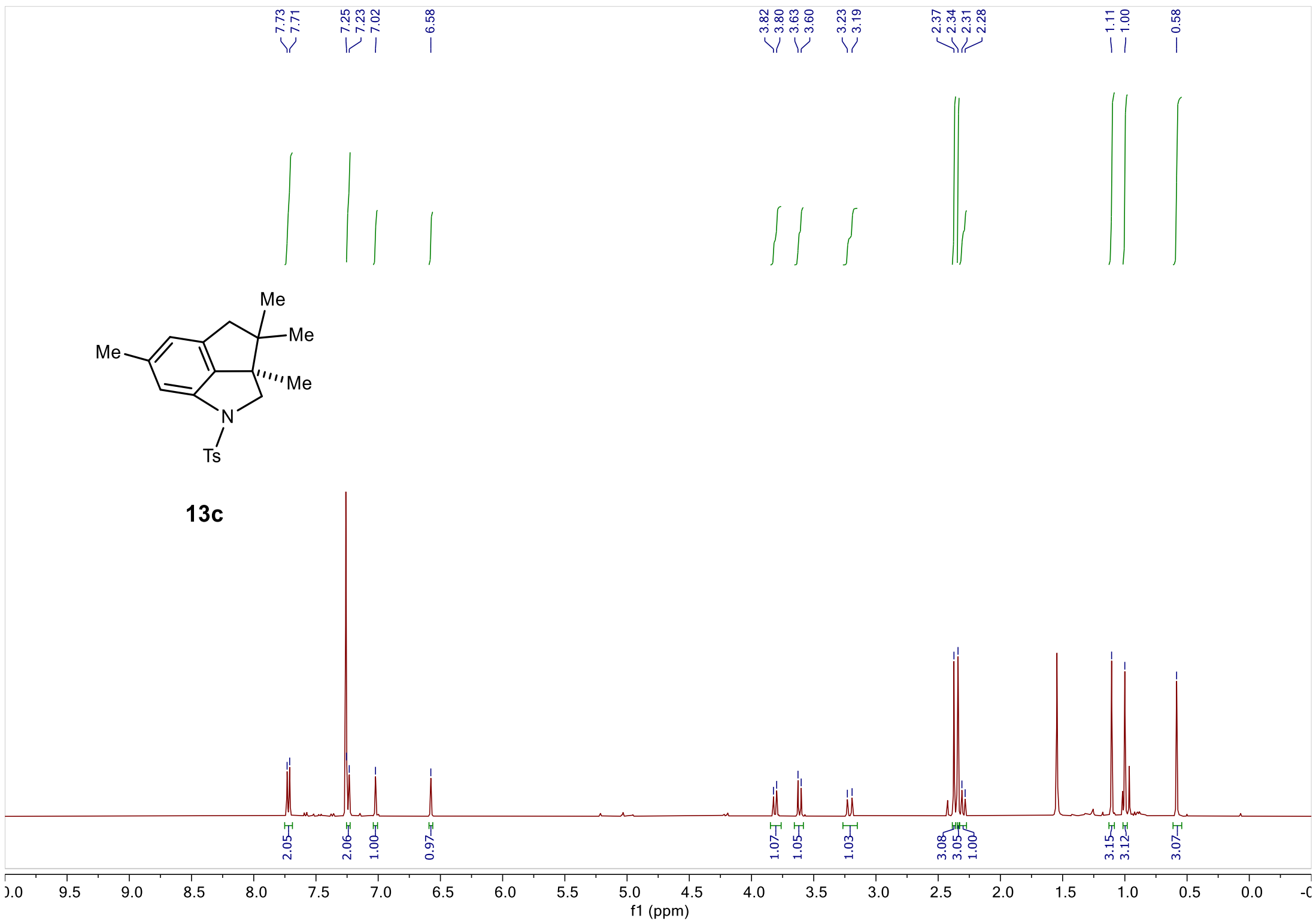

$^{13}C$ NMR (101 MHz, $CDCl_3$)

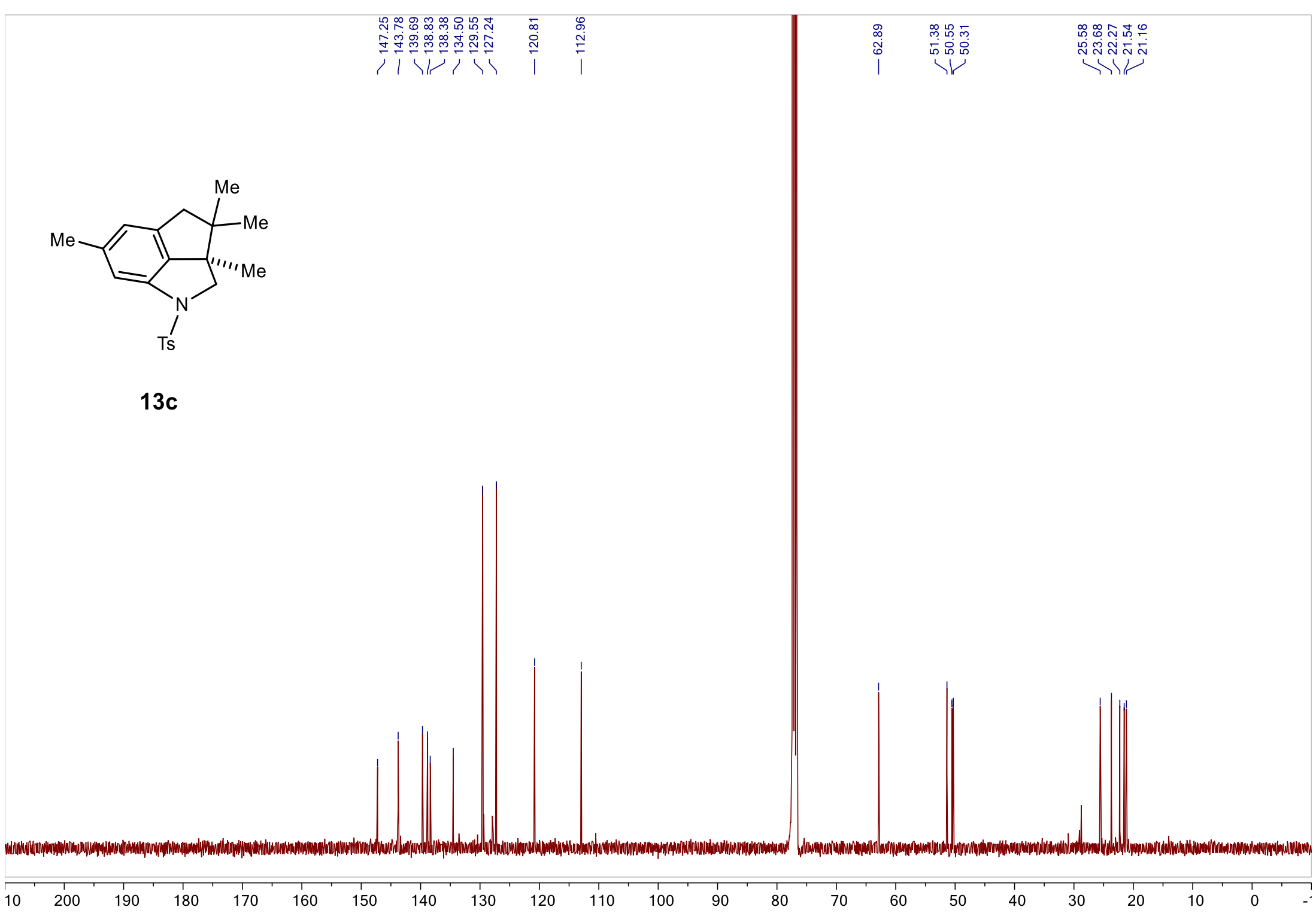

$^{1}H$ NMR (400 MHz, $CDCl_3$)

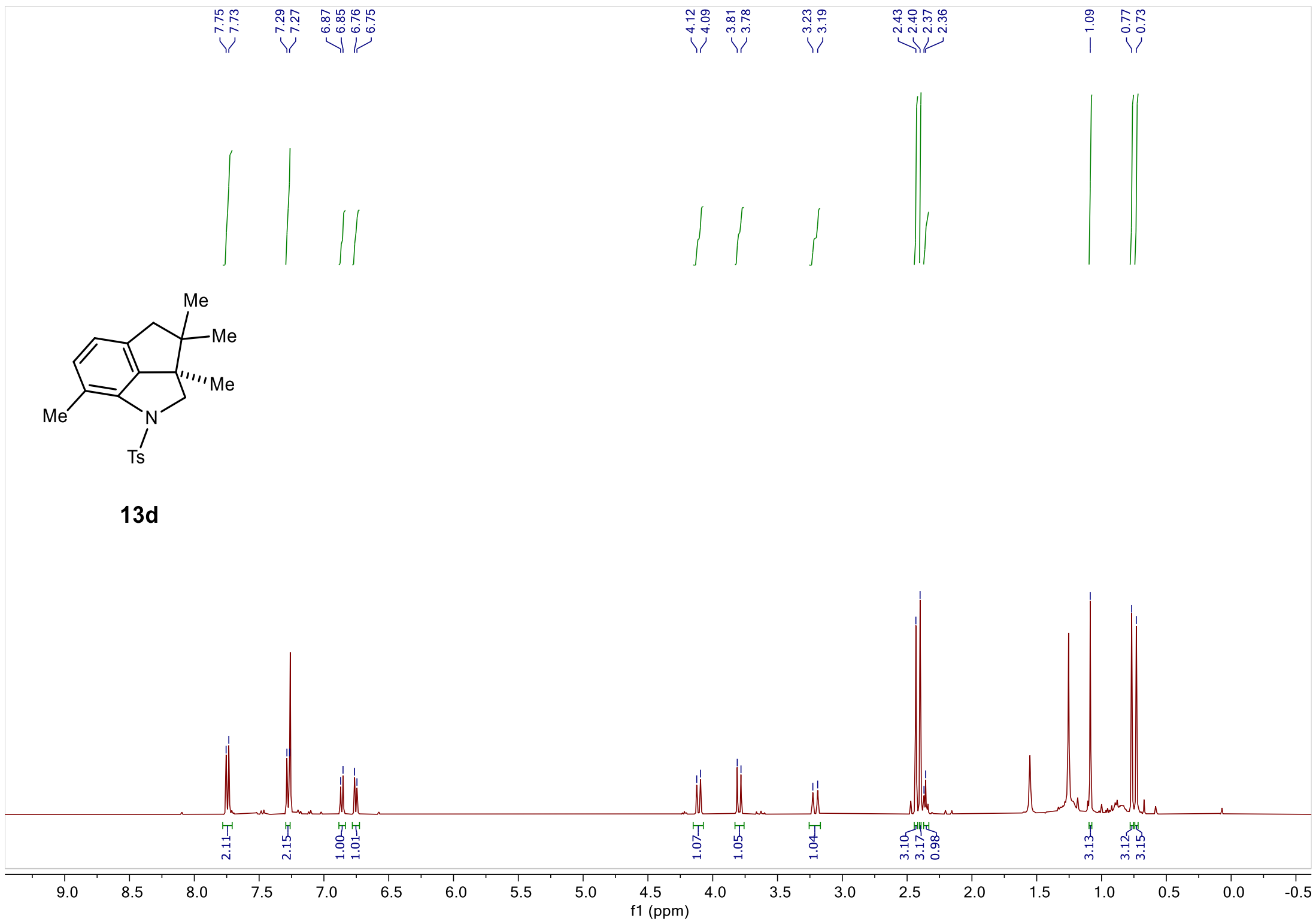


$^{13}C$ NMR (101 MHz, $CDCl_3$)

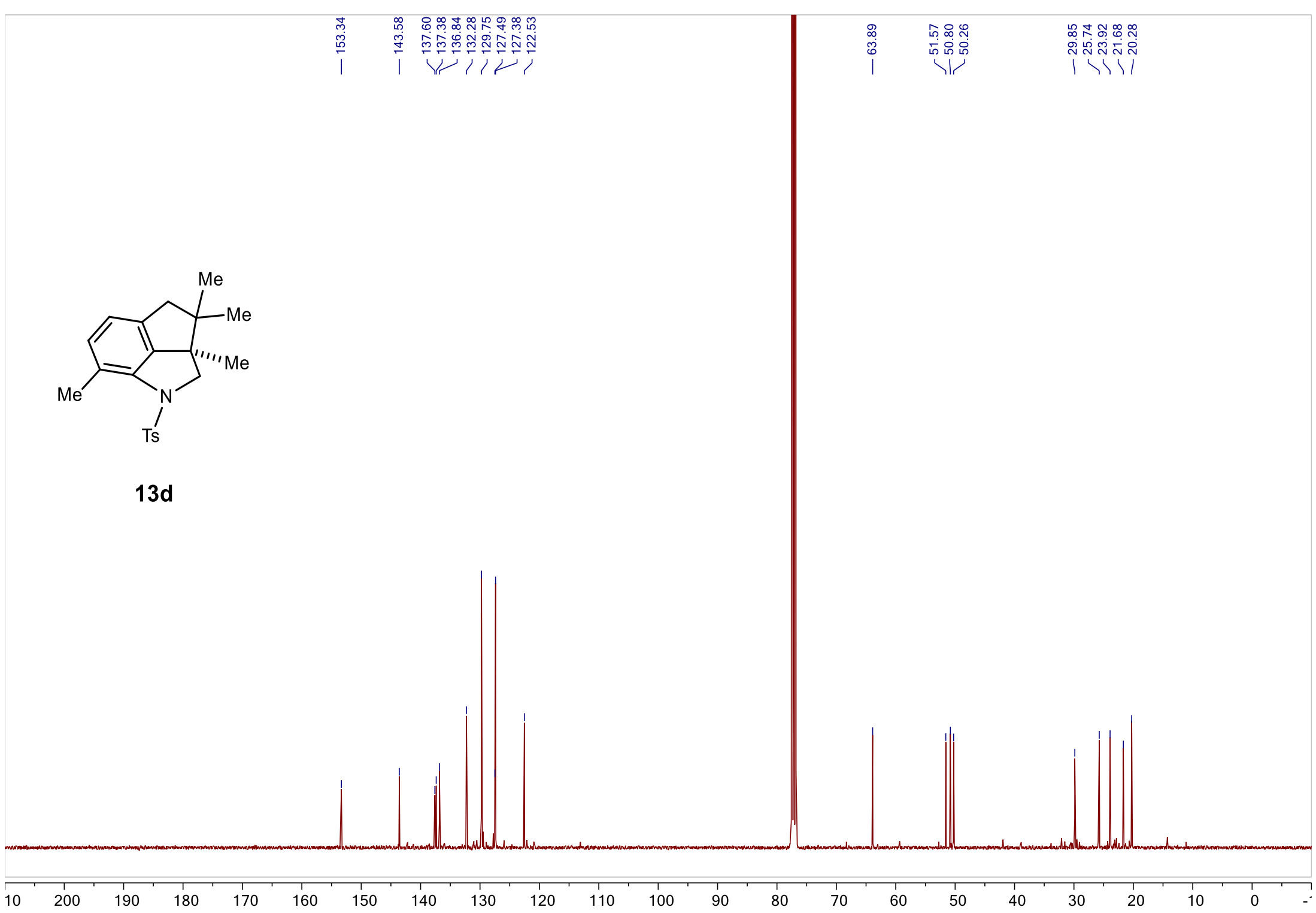

$^{1}H$ NMR (400 MHz, $CDCl_3$)

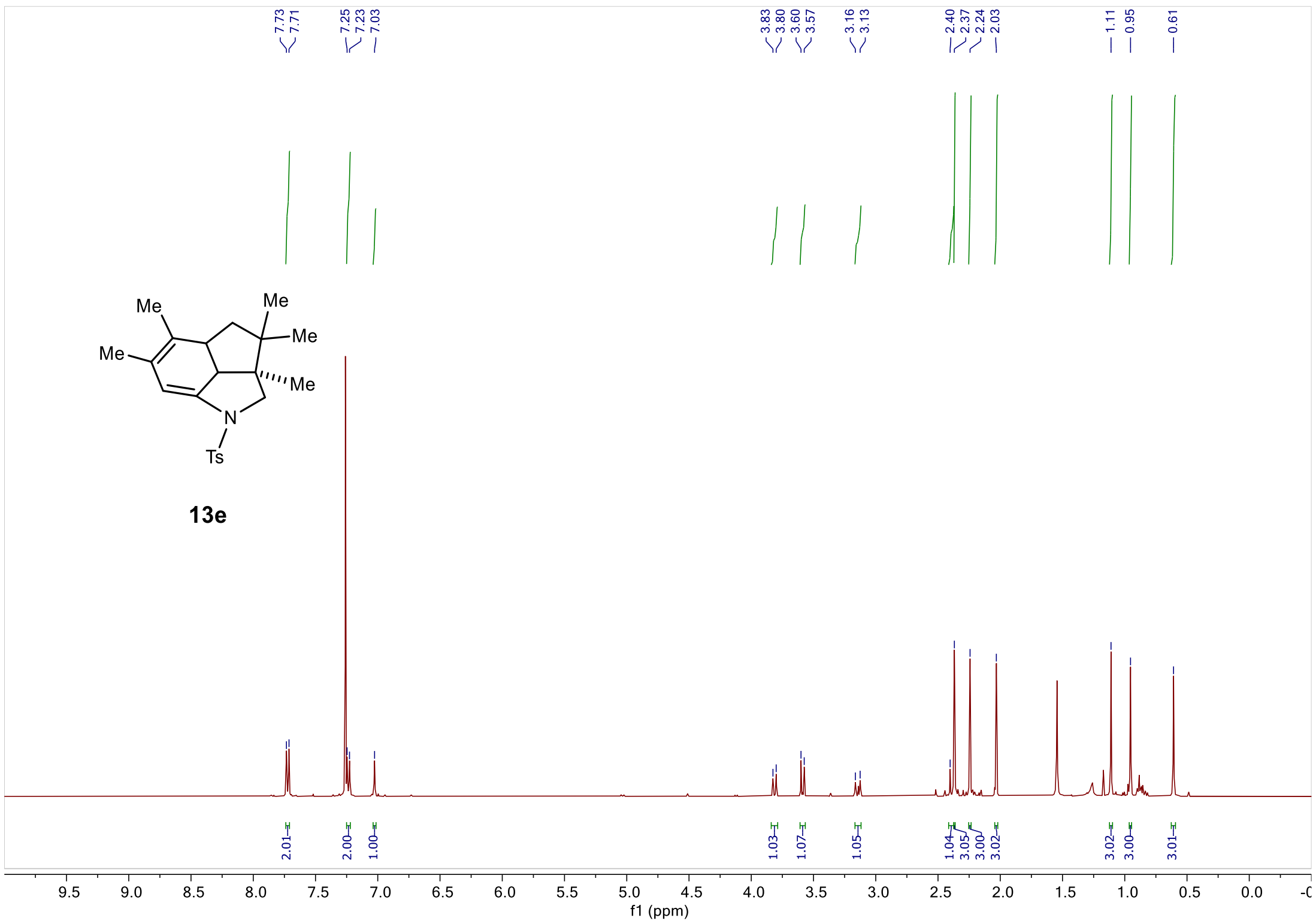


$^{13}C$ NMR (101 MHz, $CDCl_3$)

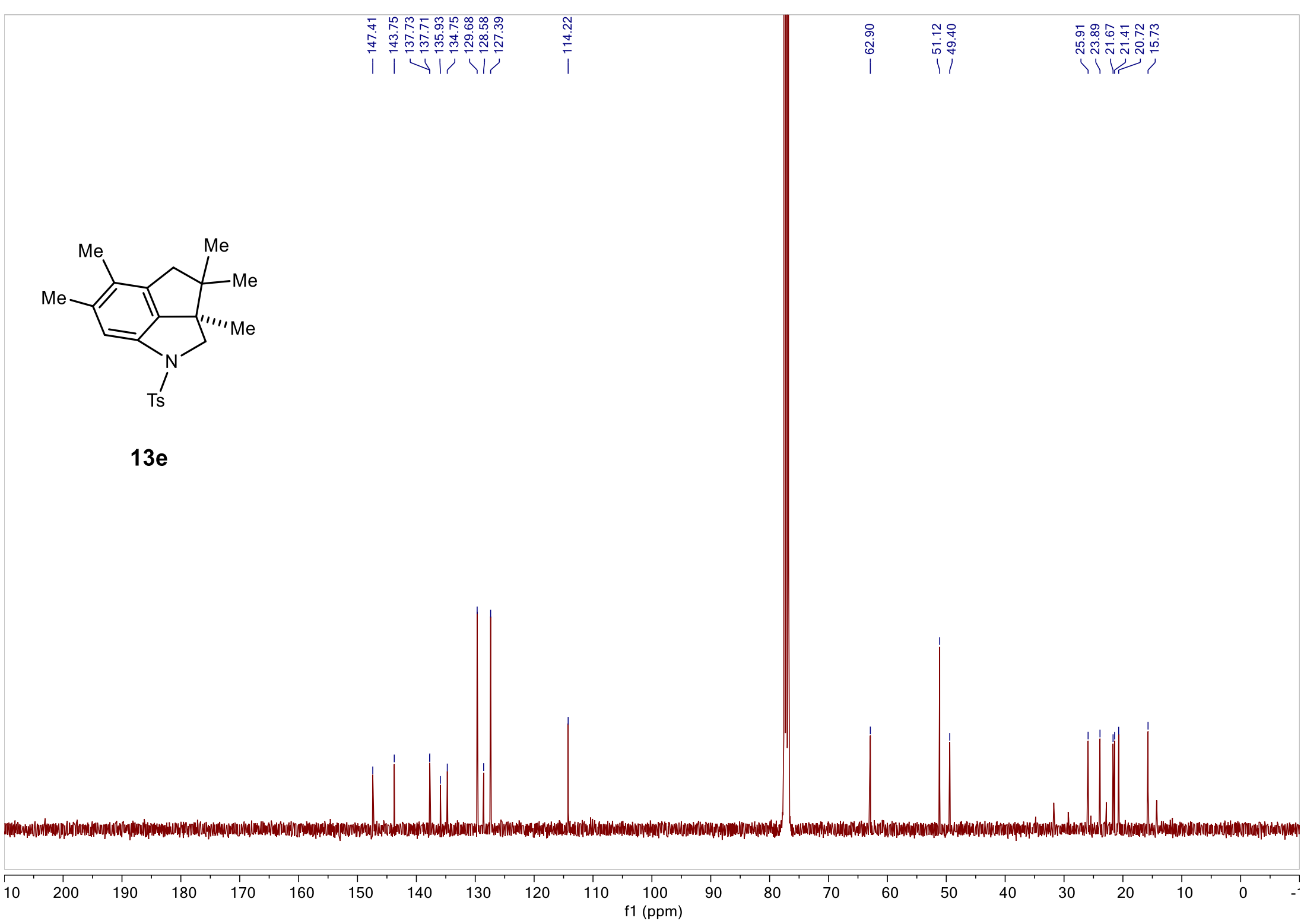

$^{1}H$ NMR (500 MHz, $CDCl_3$)

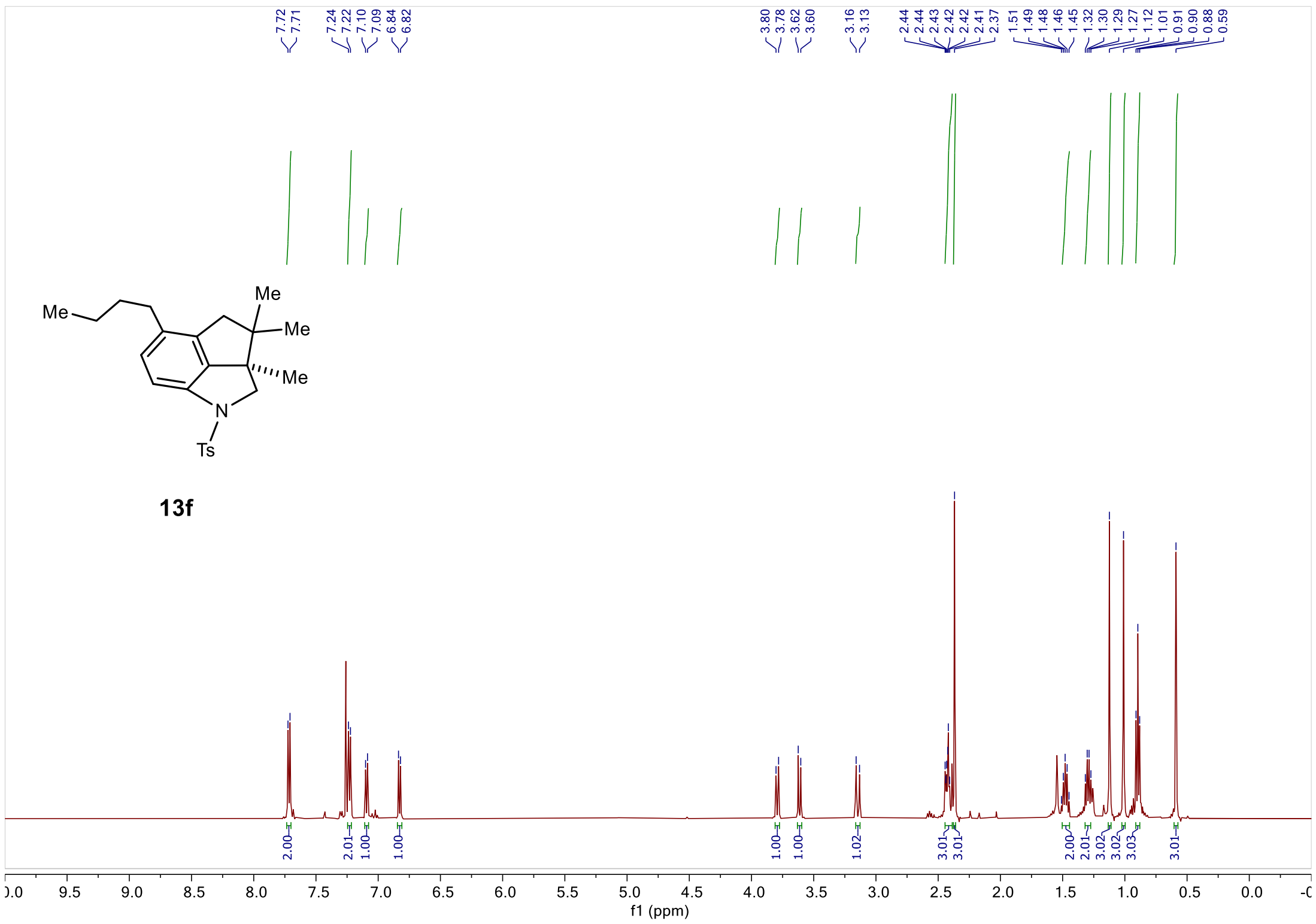

$^{13}C$ NMR (126 MHz, $CDCl_3$)

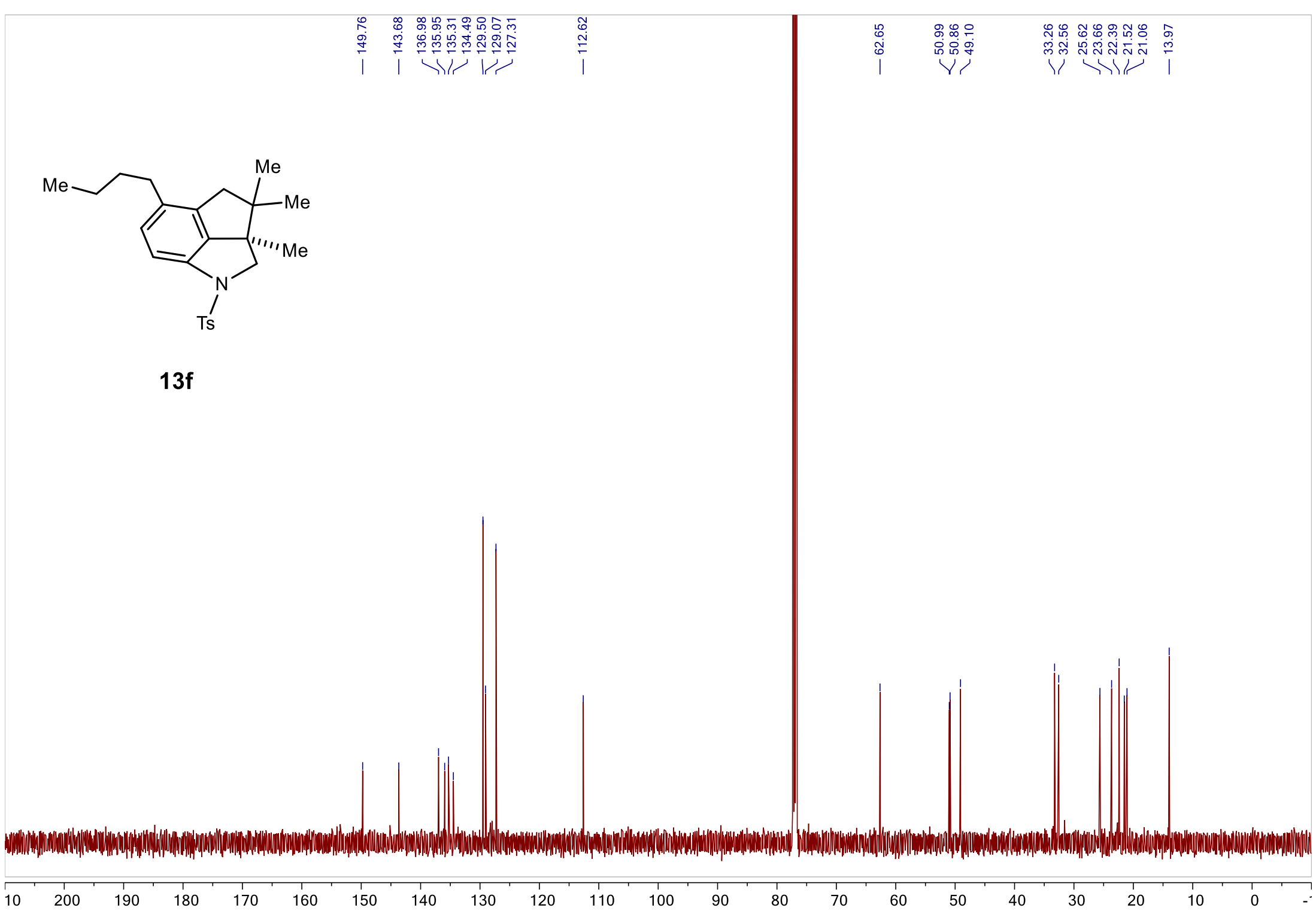

$^{1}H$ NMR (400 MHz, $CDCl_3$)

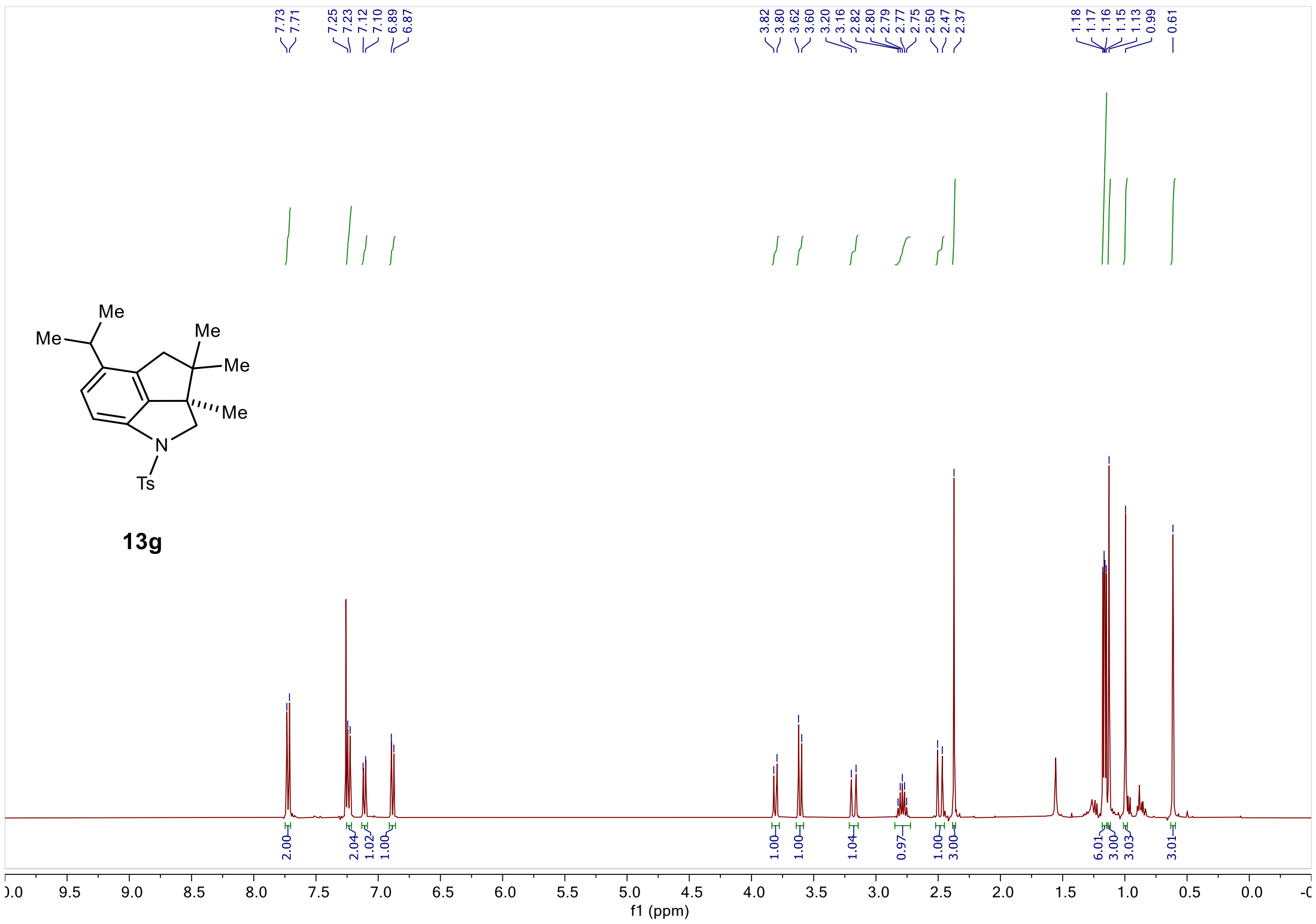

$^{13}C$ NMR (101 MHz, $CDCl_3$)

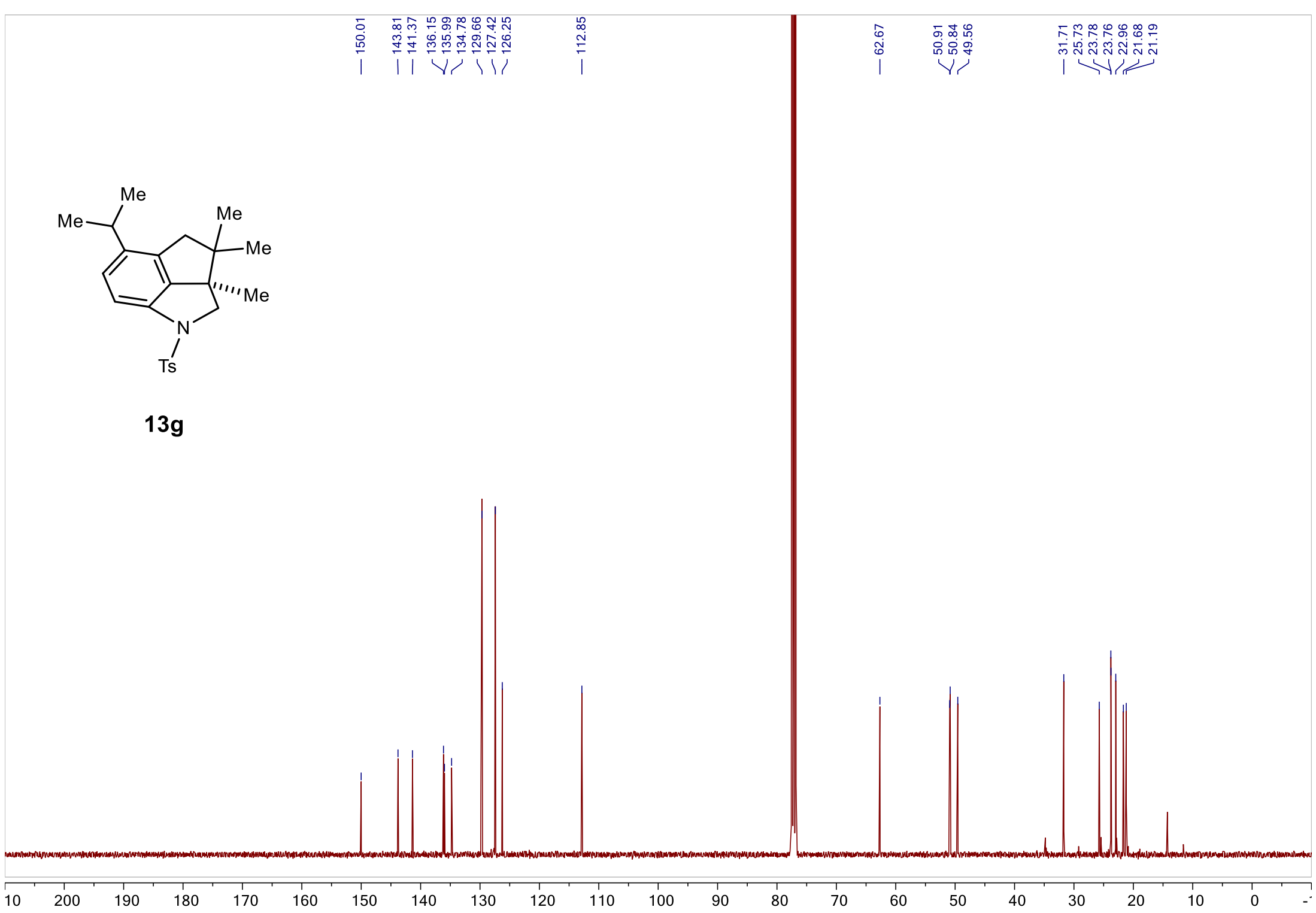

$^{1}H$ NMR (400 MHz, $CDCl_3$)

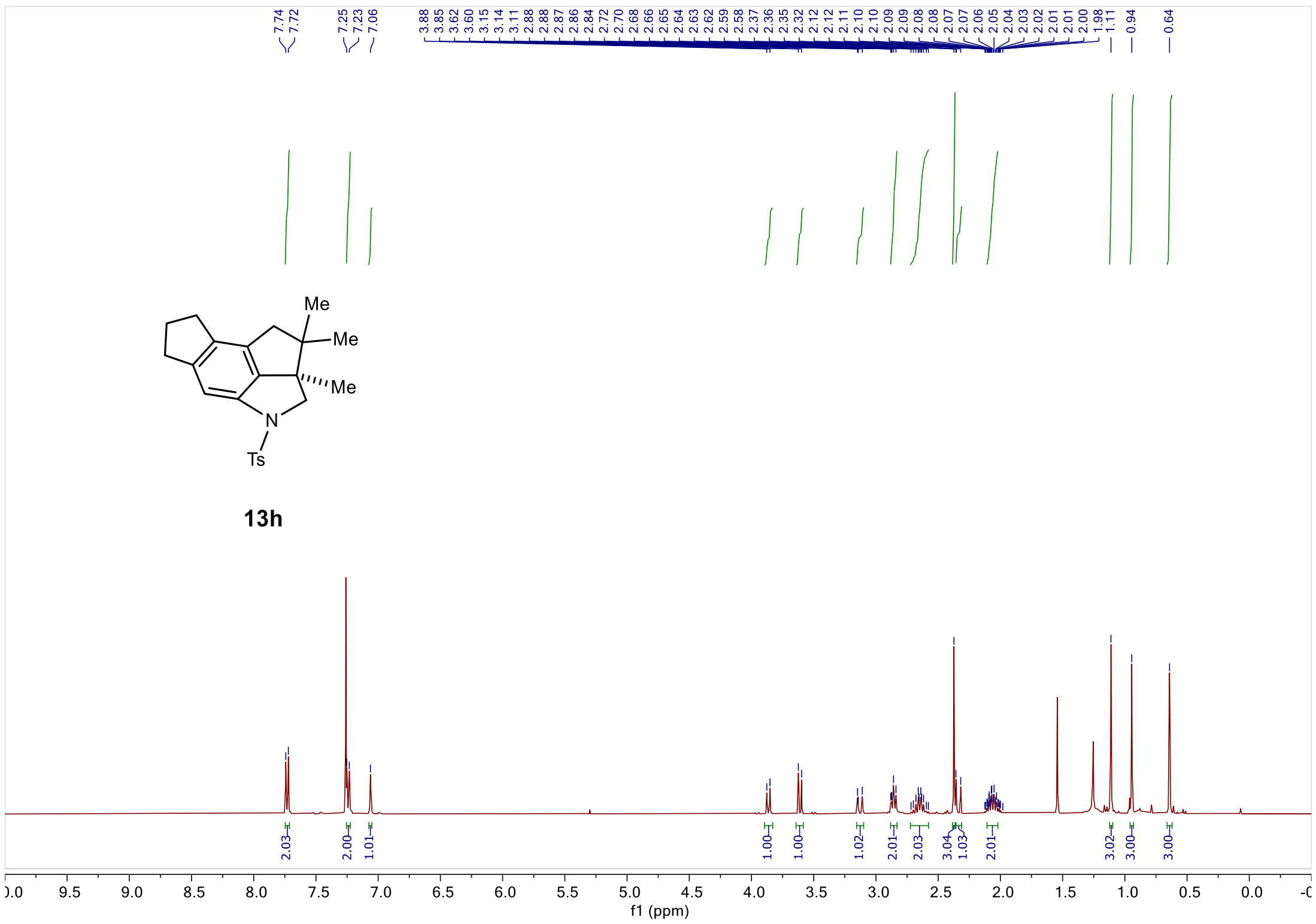

$^{13}C$ NMR (101 MHz, $CDCl_3$)

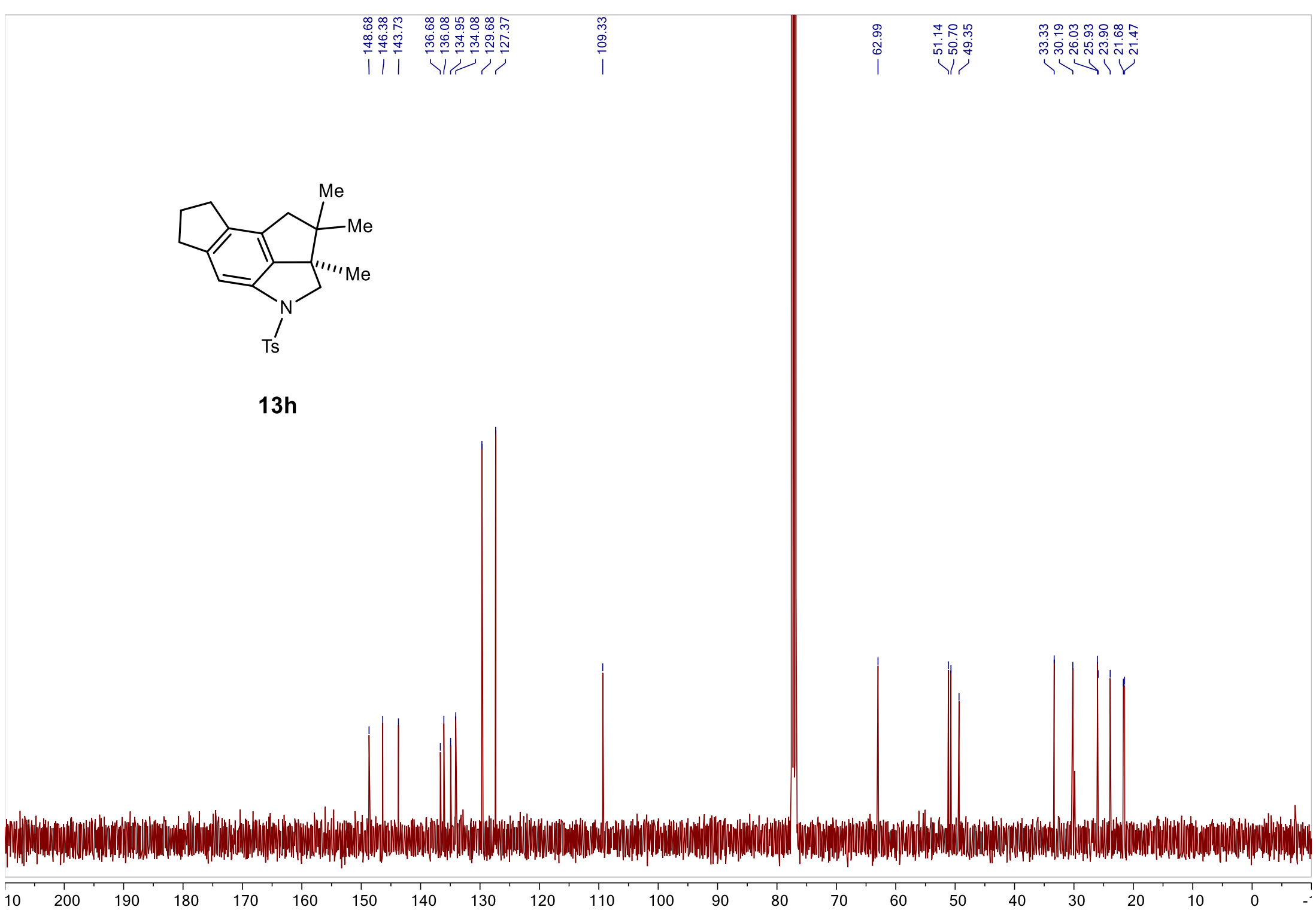

$^{1}H$ NMR (400 MHz, $CDCl_3$)

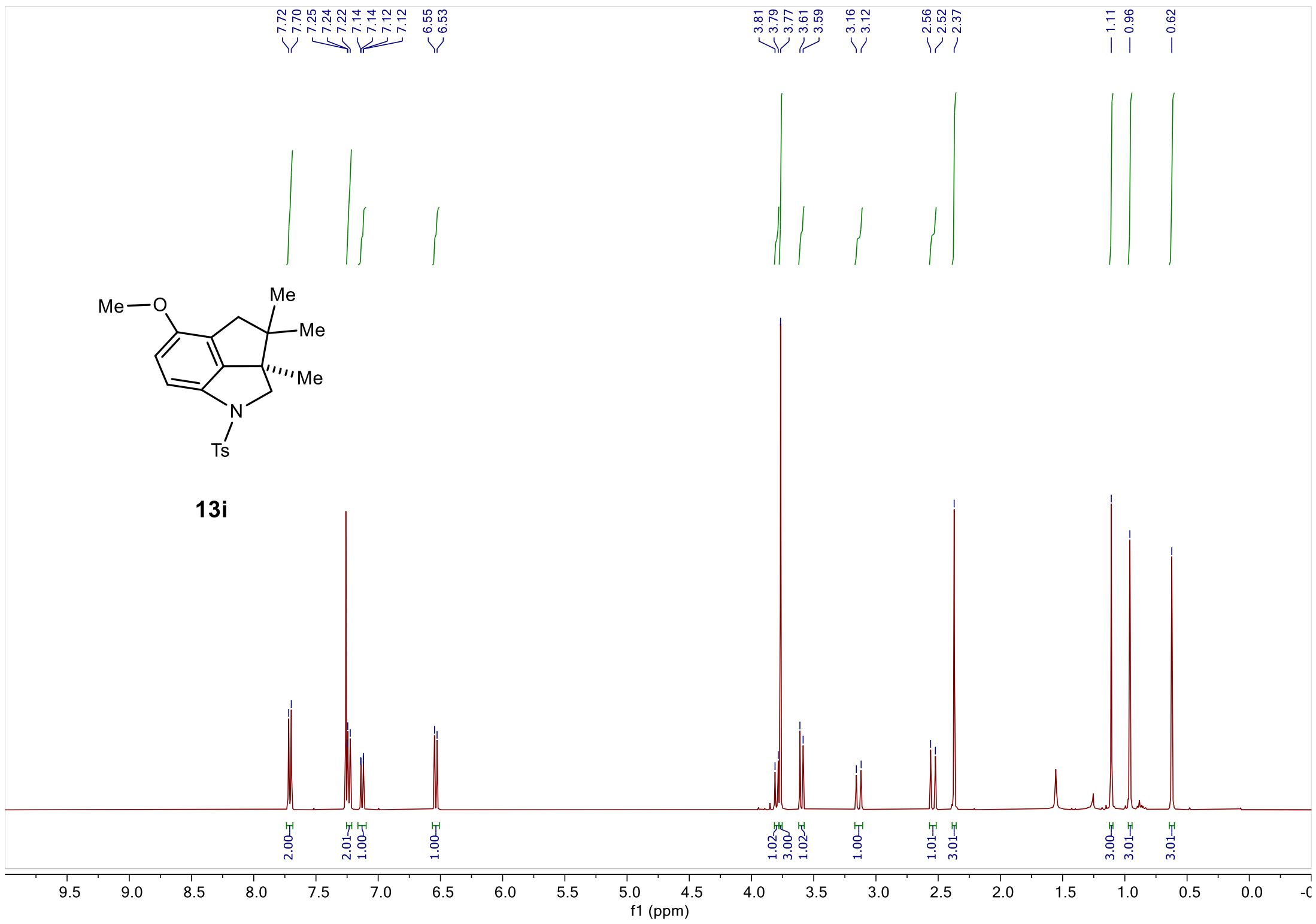

$^{13}C$ NMR (101 MHz, $CDCl_3$)

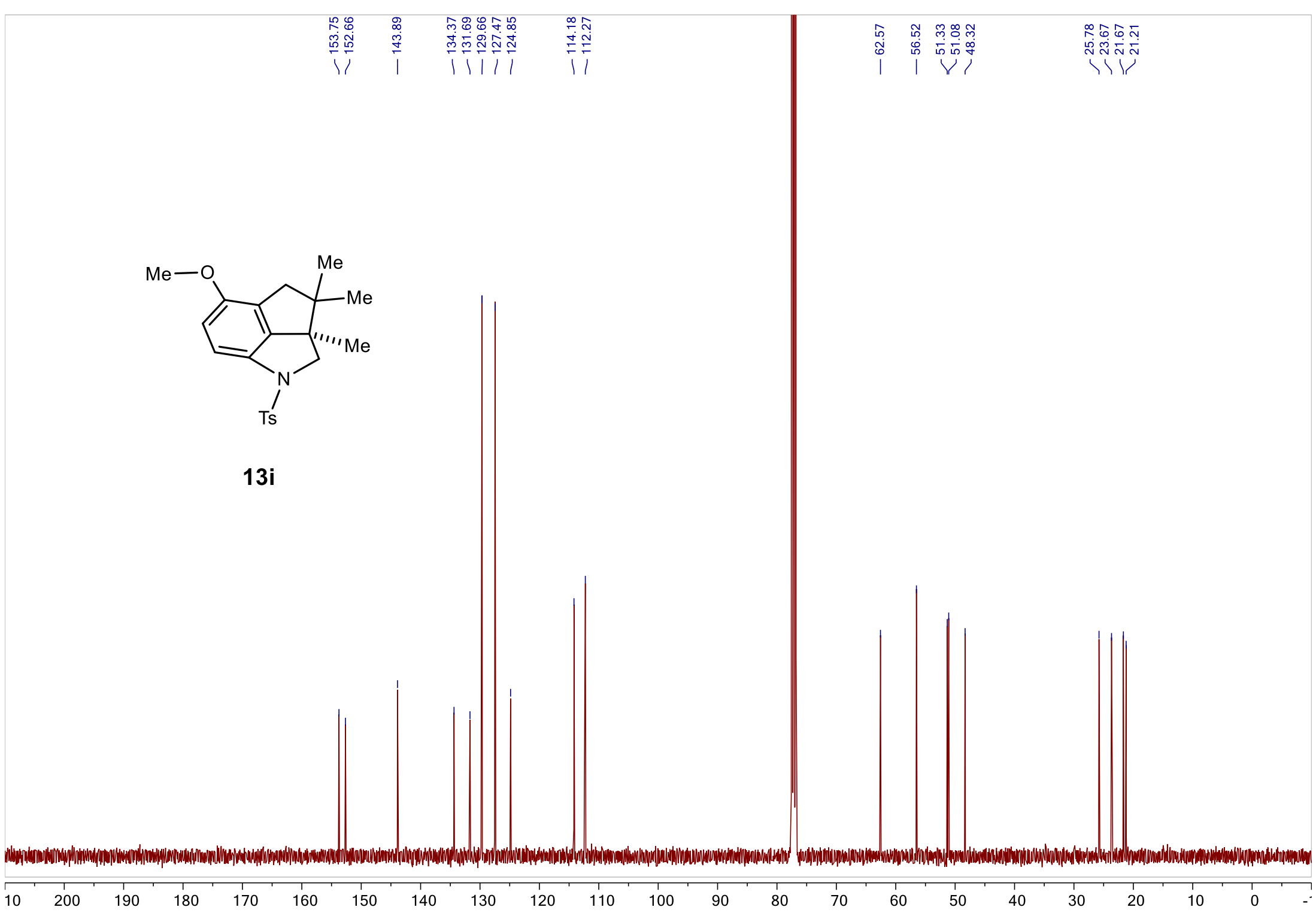

$^{1}$H NMR (400 MHz, $CDCl_3$)

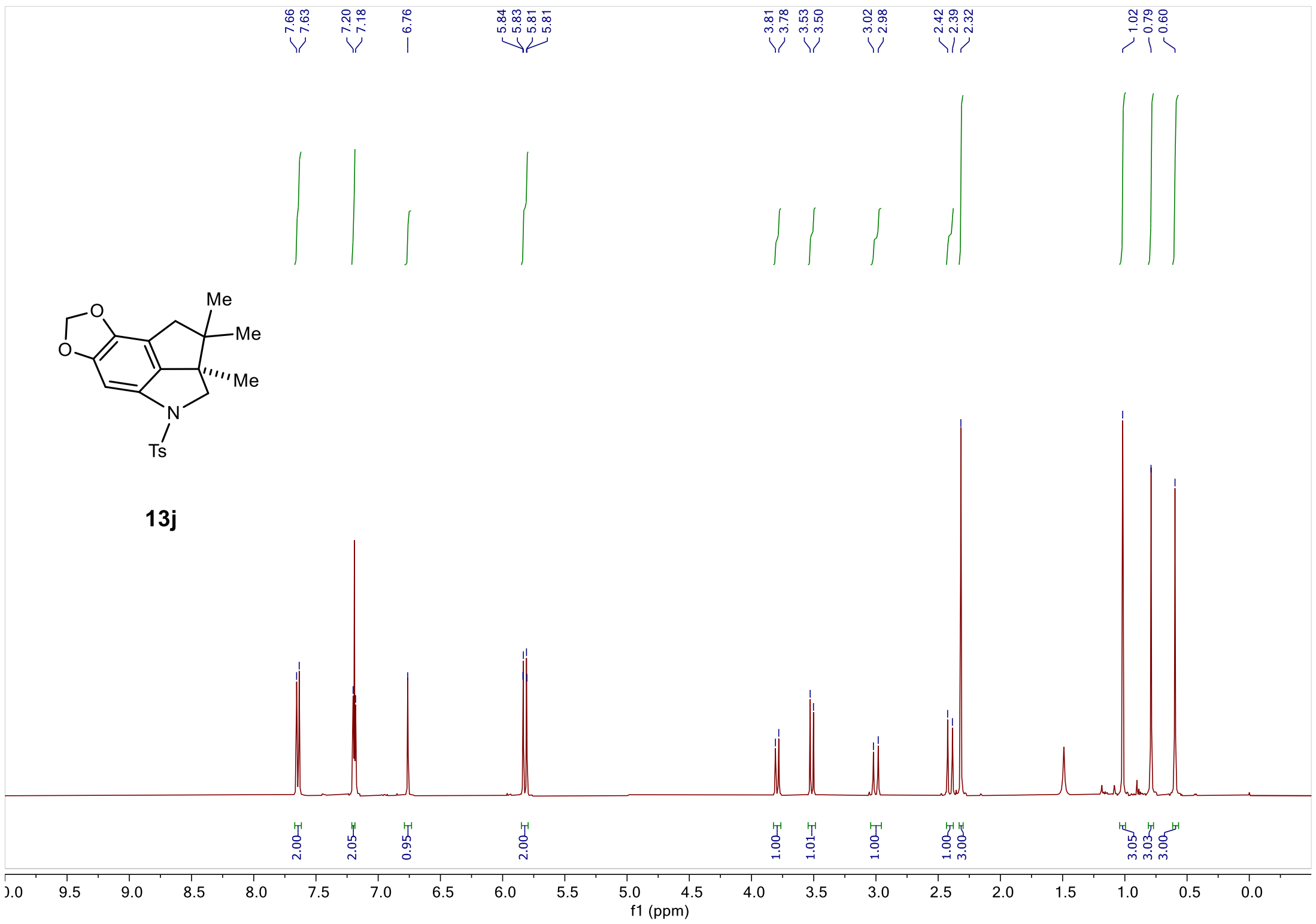


$^{13}$C NMR (101 MHz, $CDCl_3$)

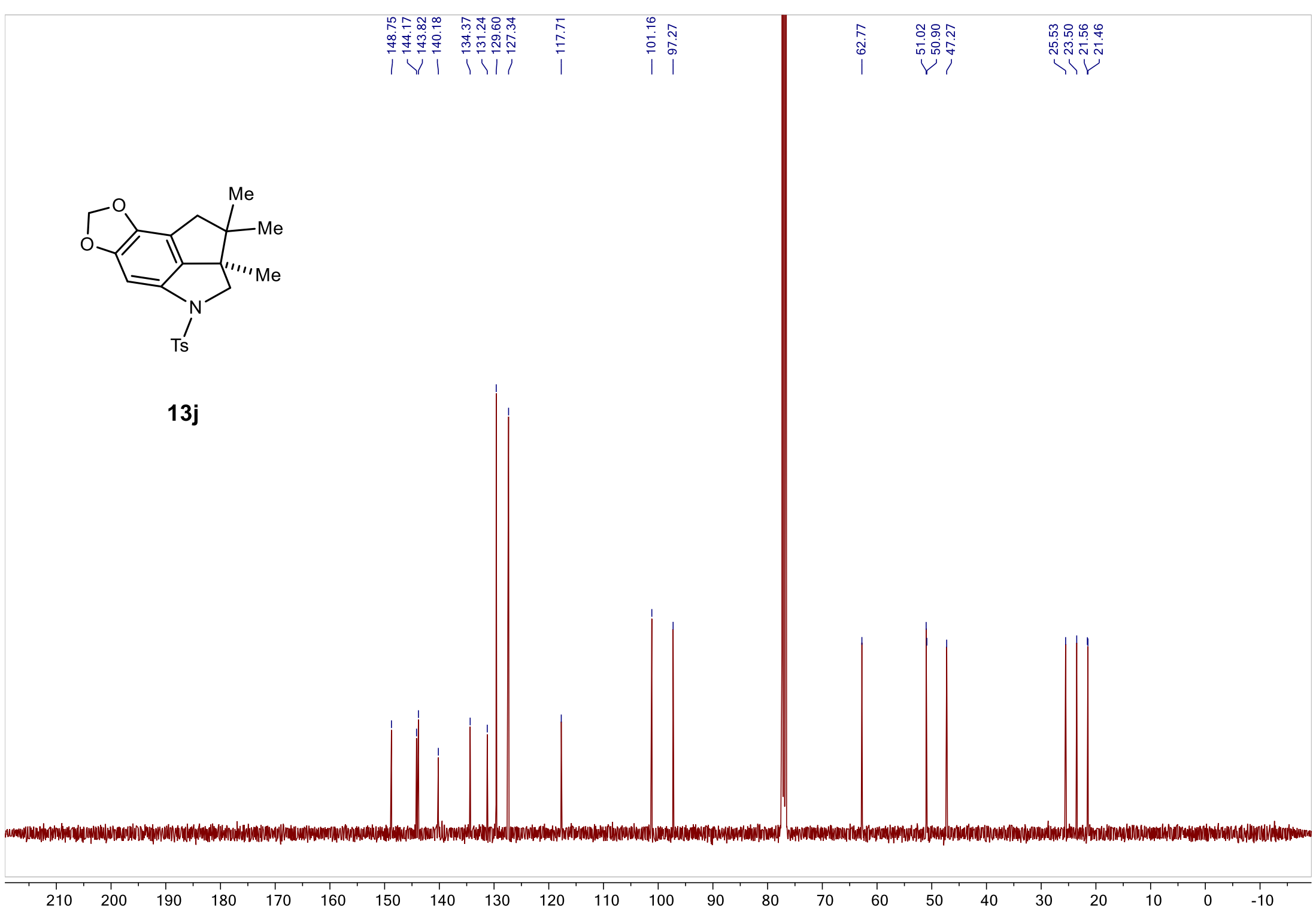

$^{1}$H NMR (400 MHz, $CDCl_3$)

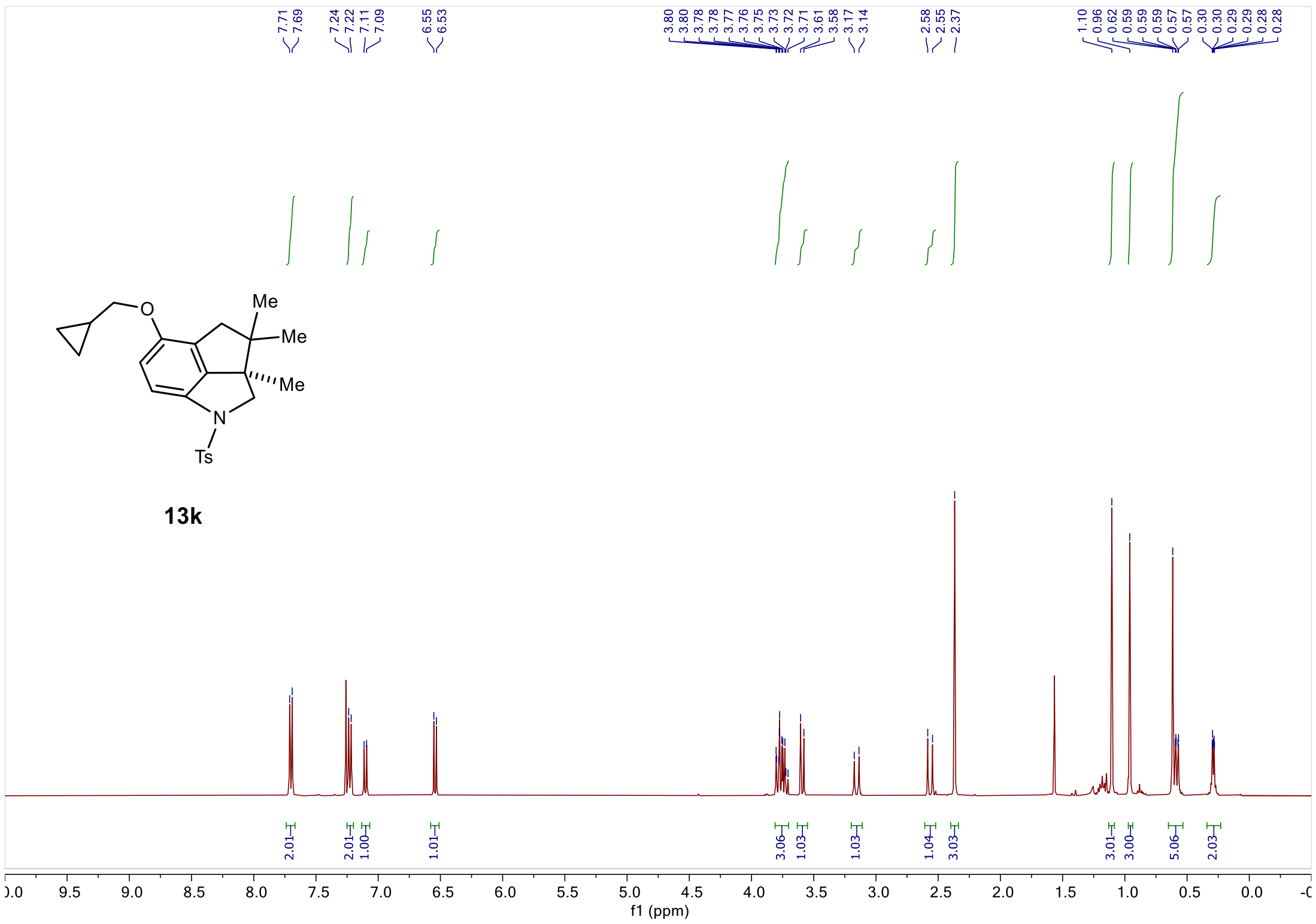

$^{13}$C NMR (101 MHz, $CDCl_3$)

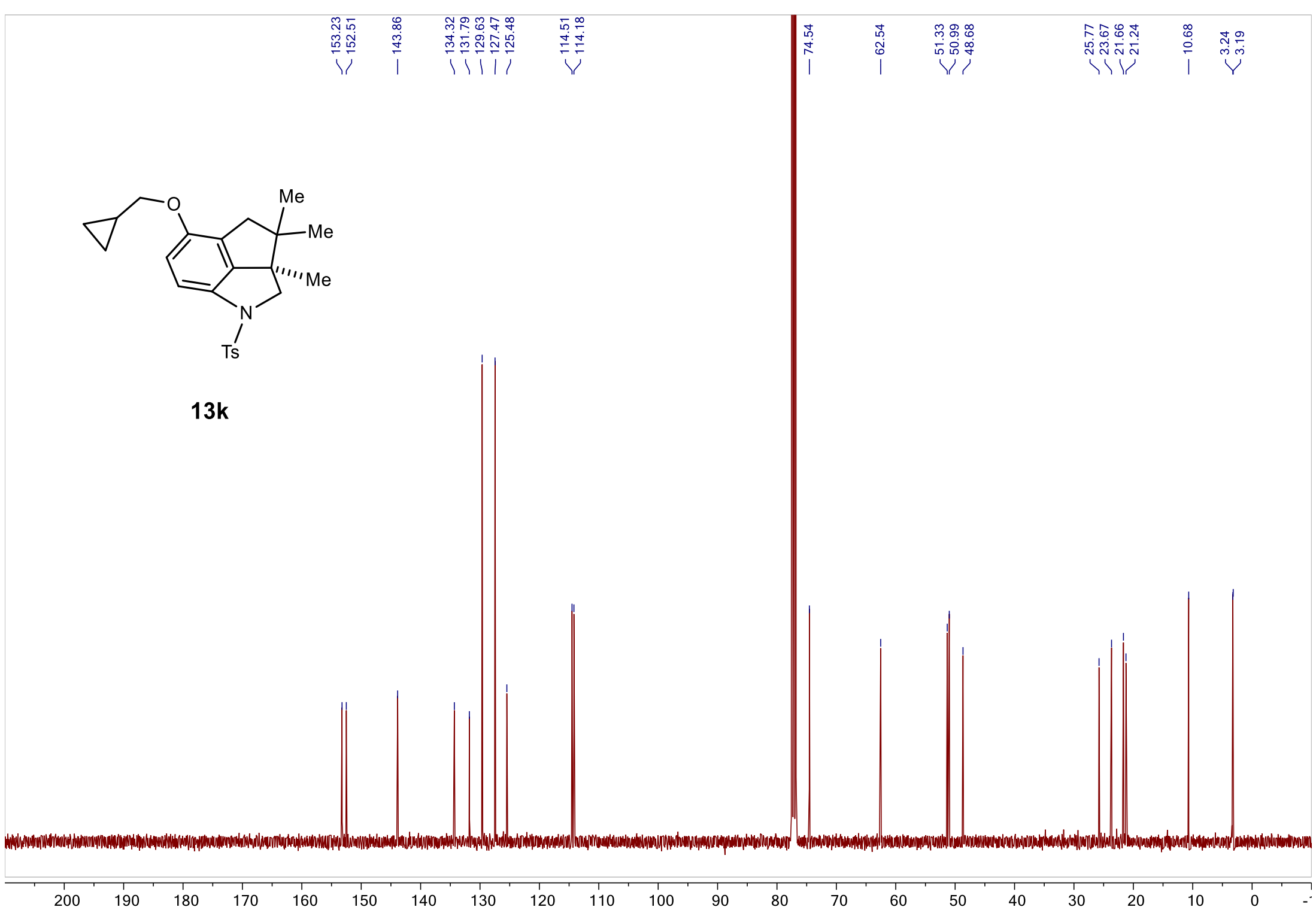

$^{1}H$ NMR (400 MHz, $CDCl_3$)

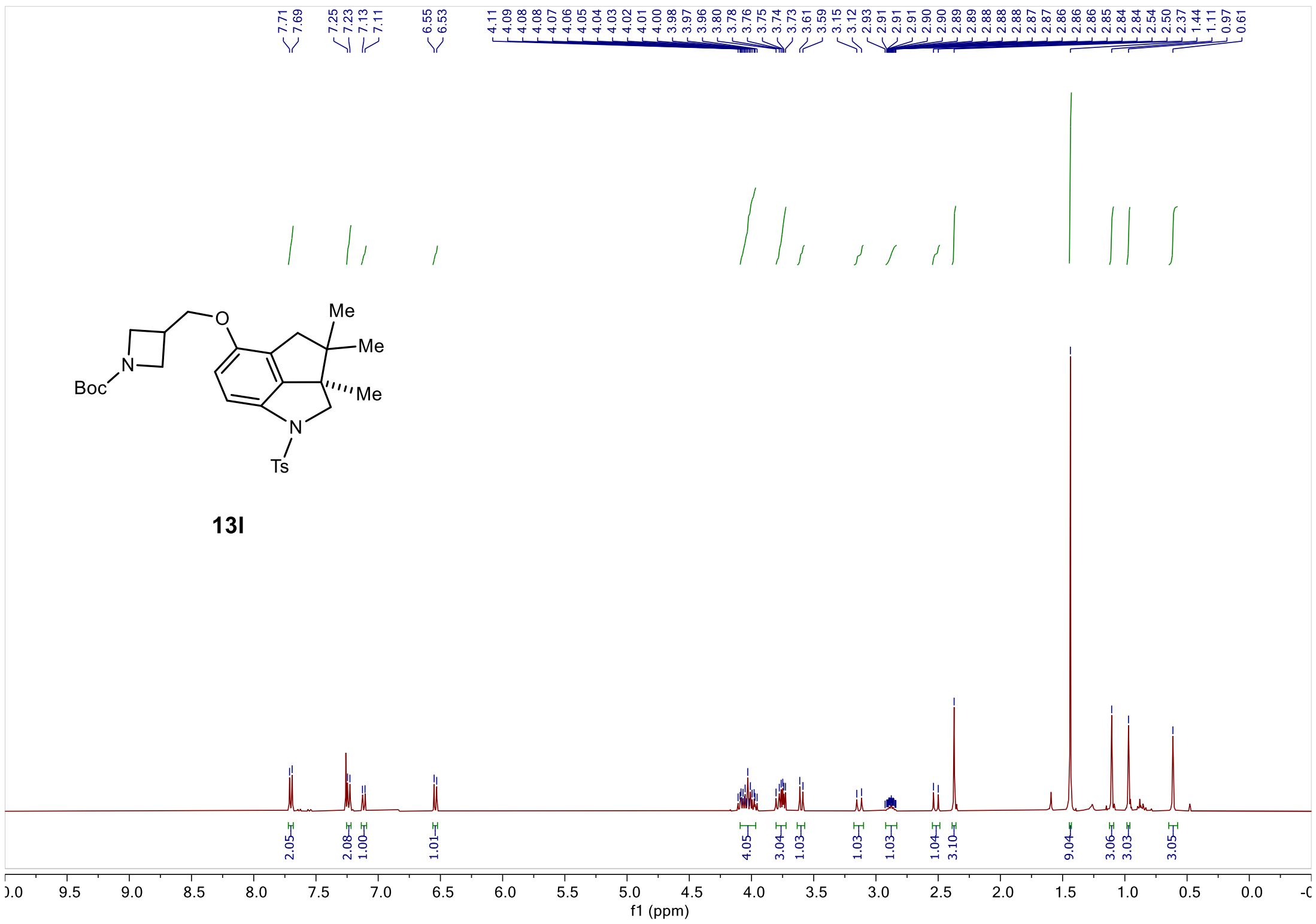


$^{13}C$ NMR (101 MHz, $CDCl_3$)

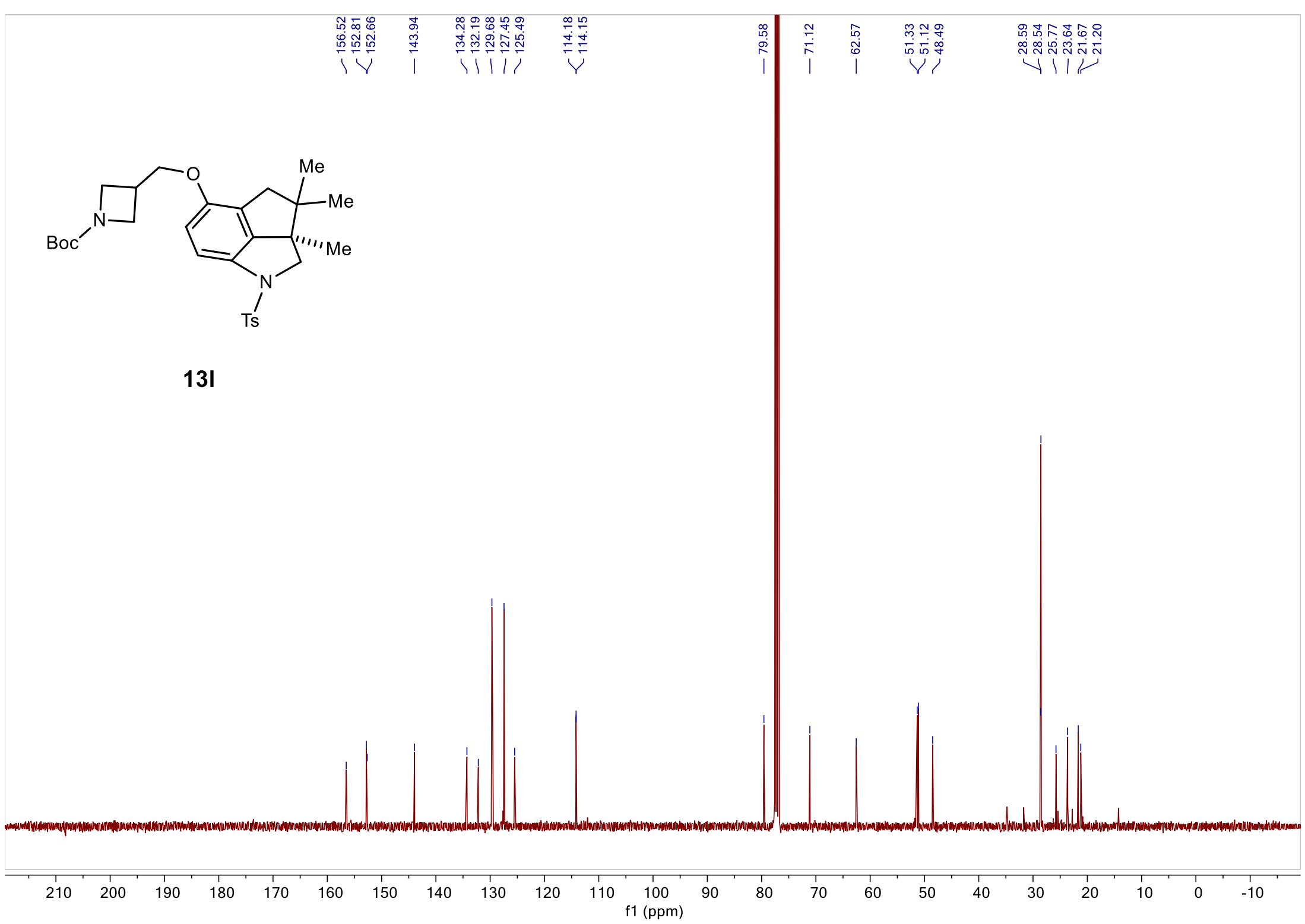

$^{1}H$ NMR (400 MHz, $CDCl_3$)

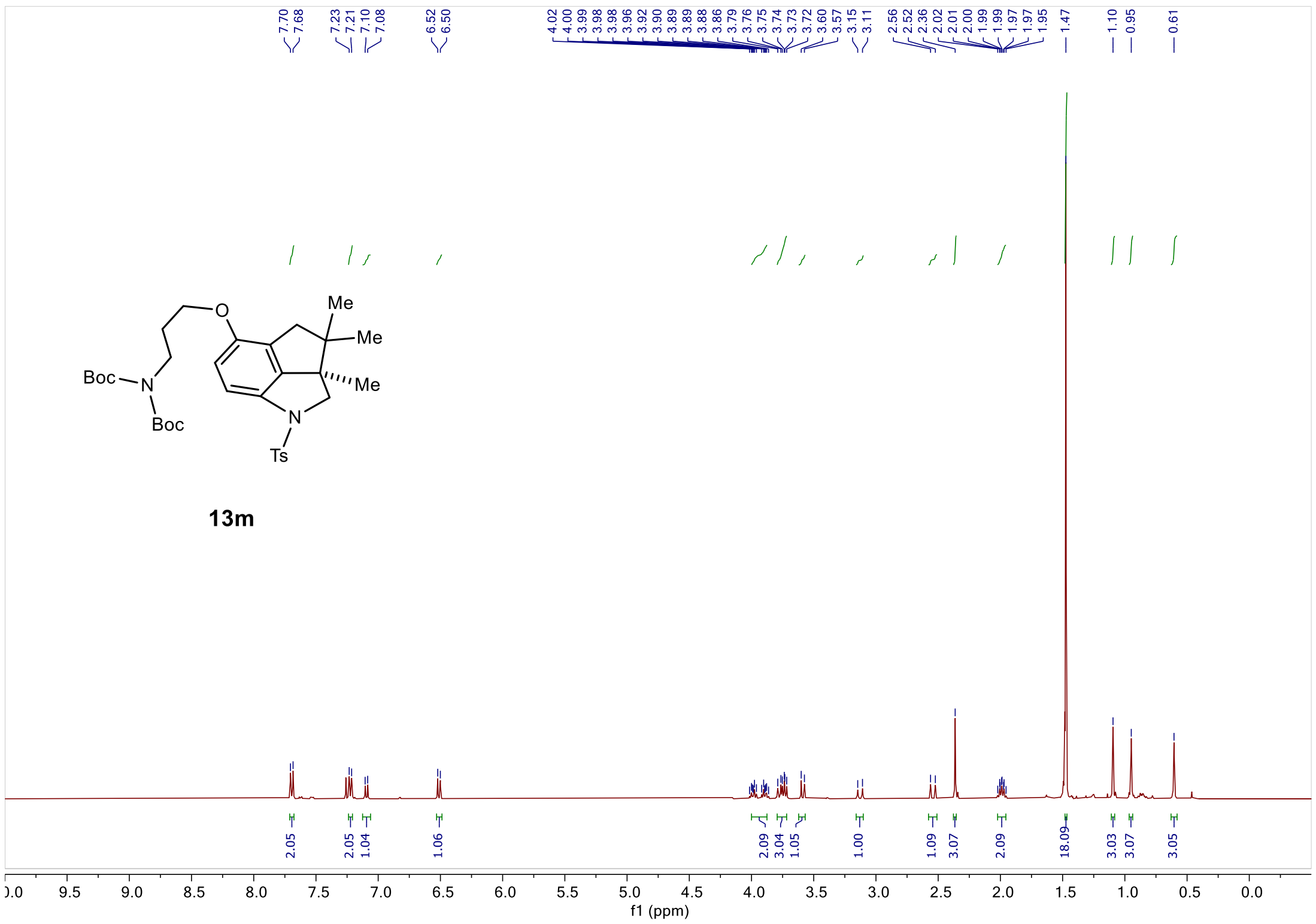


$^{13}C$ NMR (101 MHz, $CDCl_3$)

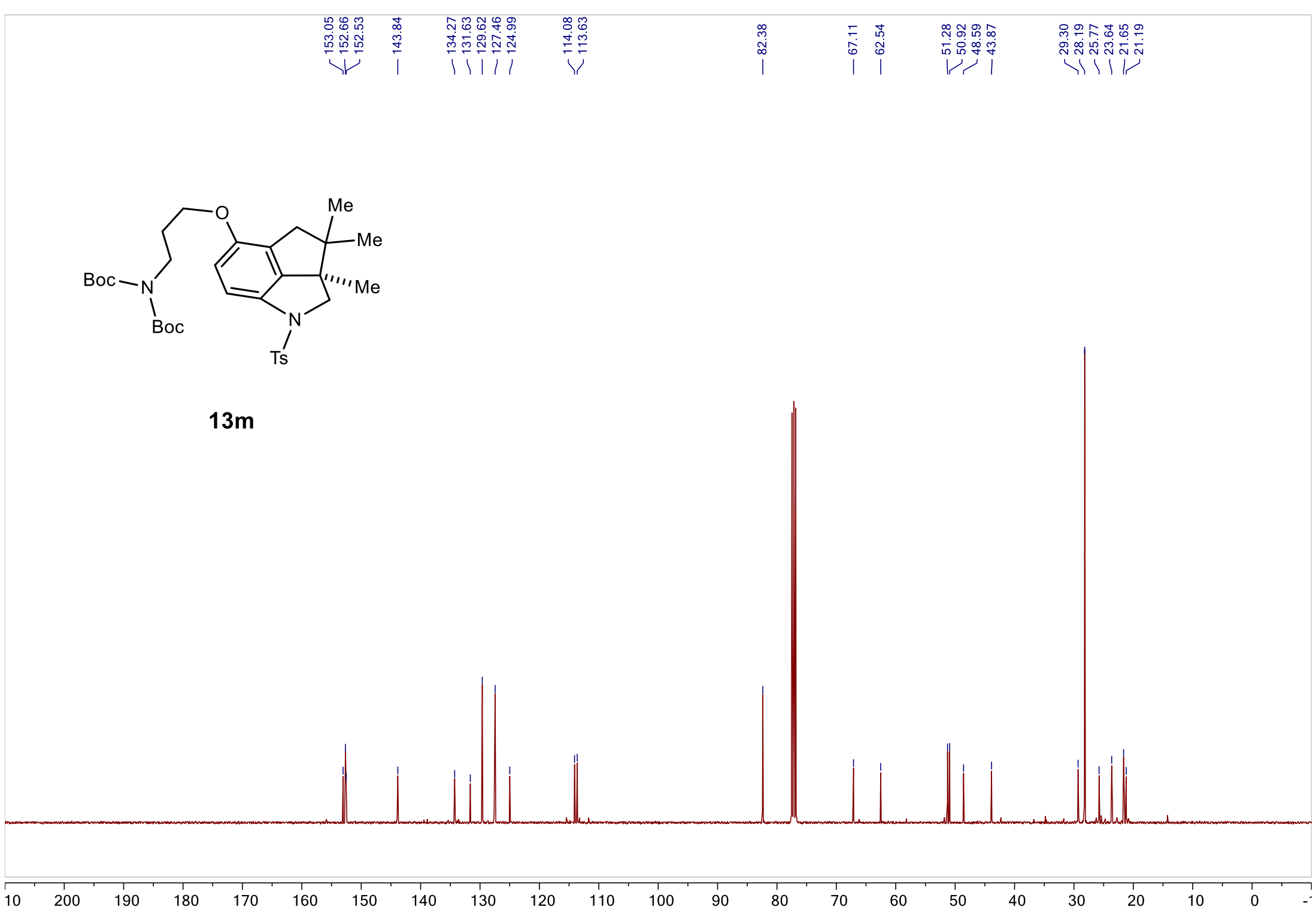

$^{1}$H NMR (400 MHz, $CDCl_3$)

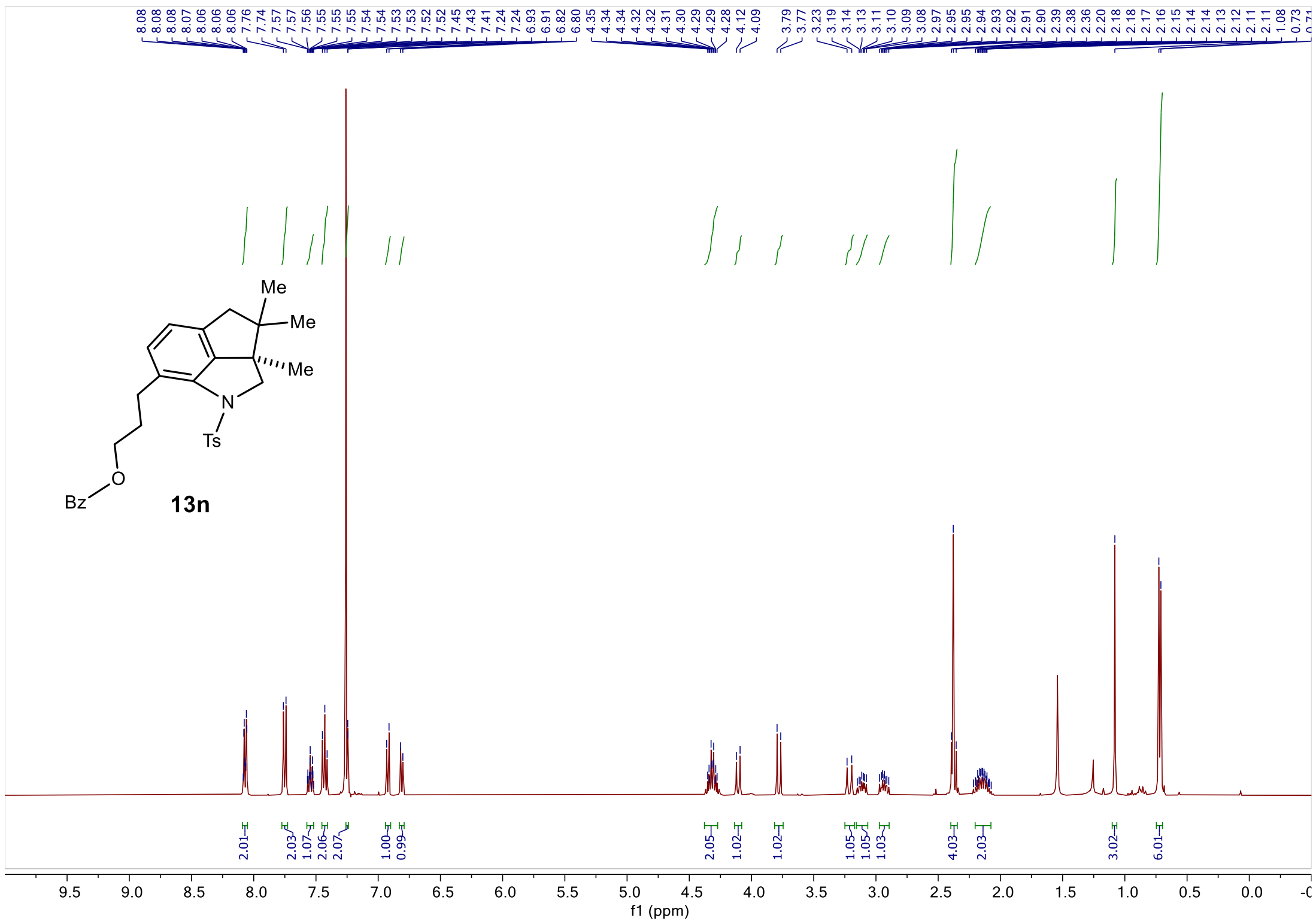


$^{13}$C NMR (101 MHz, $CDCl_3$)

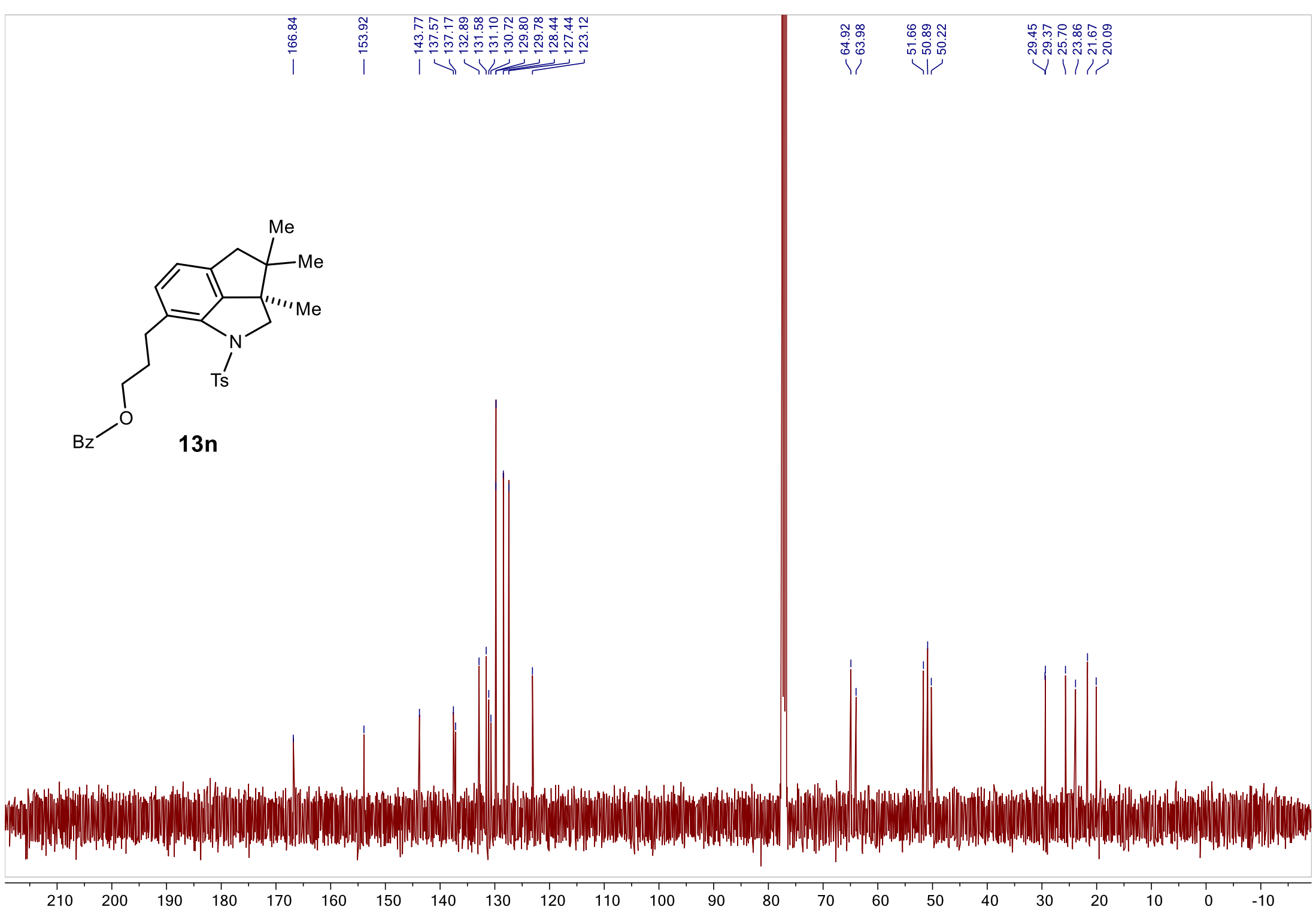

$^{1}H$ NMR (500 MHz, $CDCl_3$)

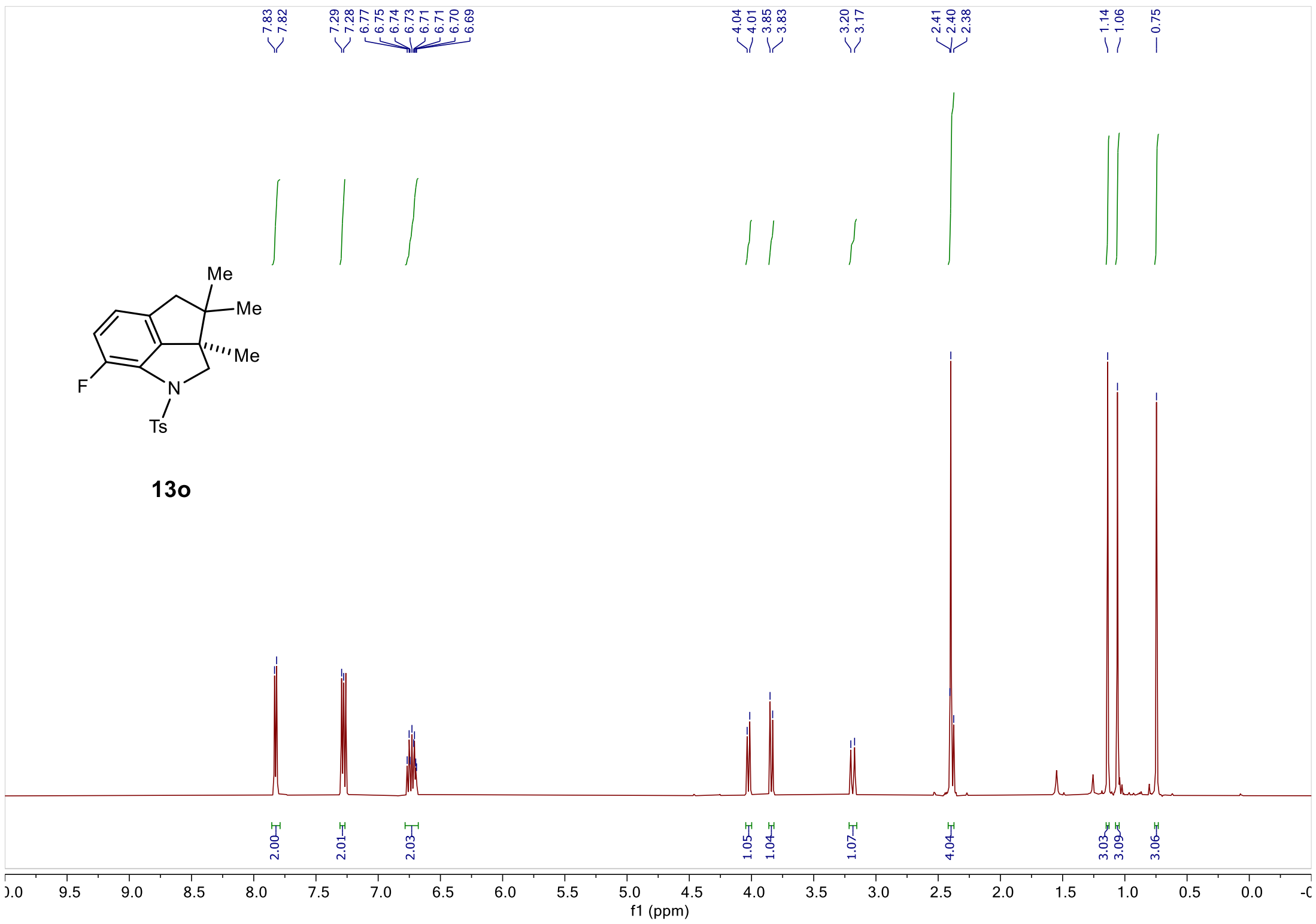

$^{13}C$ NMR (126 MHz, $CDCl_3$)

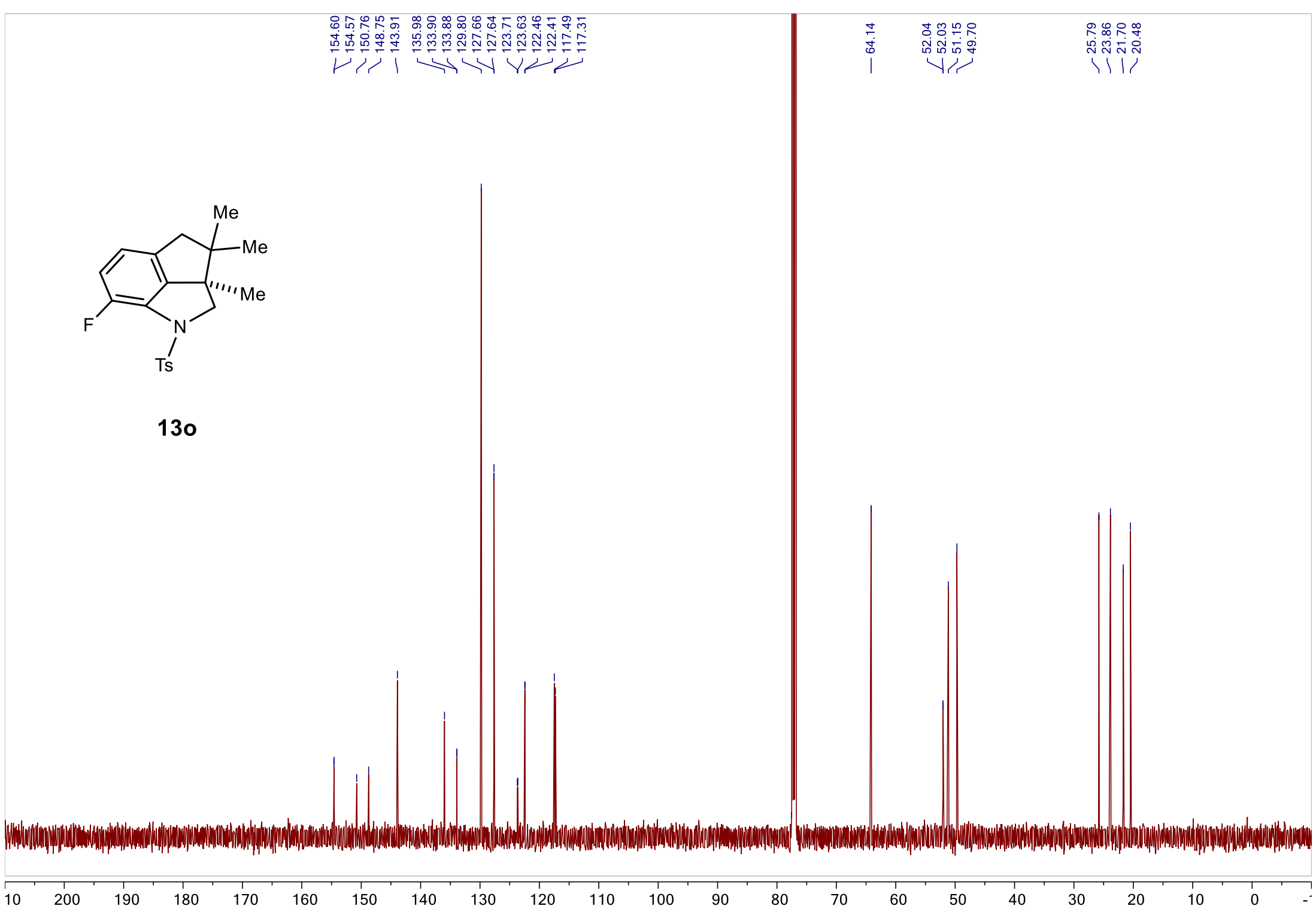

$^{19}F$ NMR (471 MHz, $CDCl_3$)

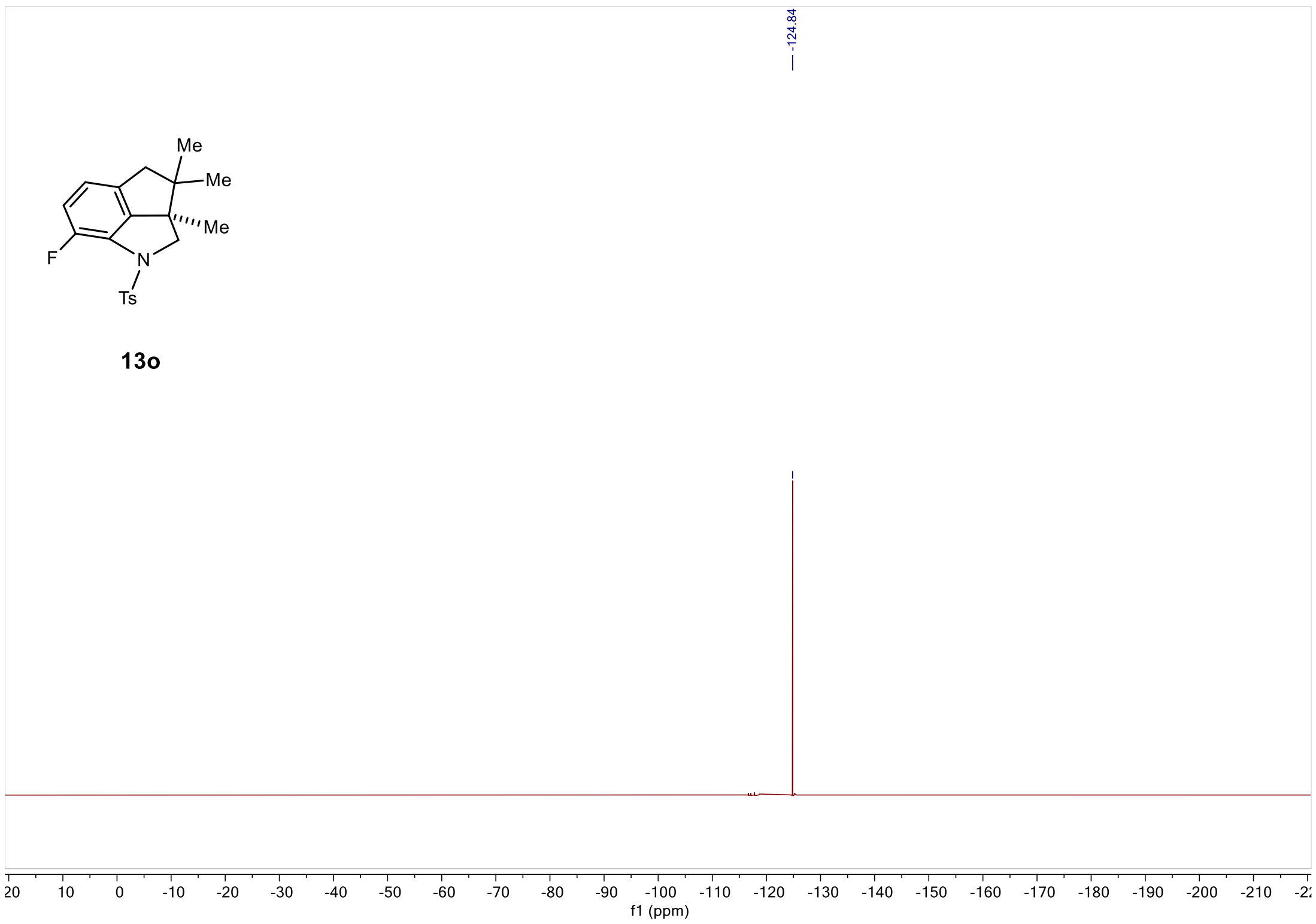

$^{1}H$ NMR (400 MHz, $CDCl_3$)

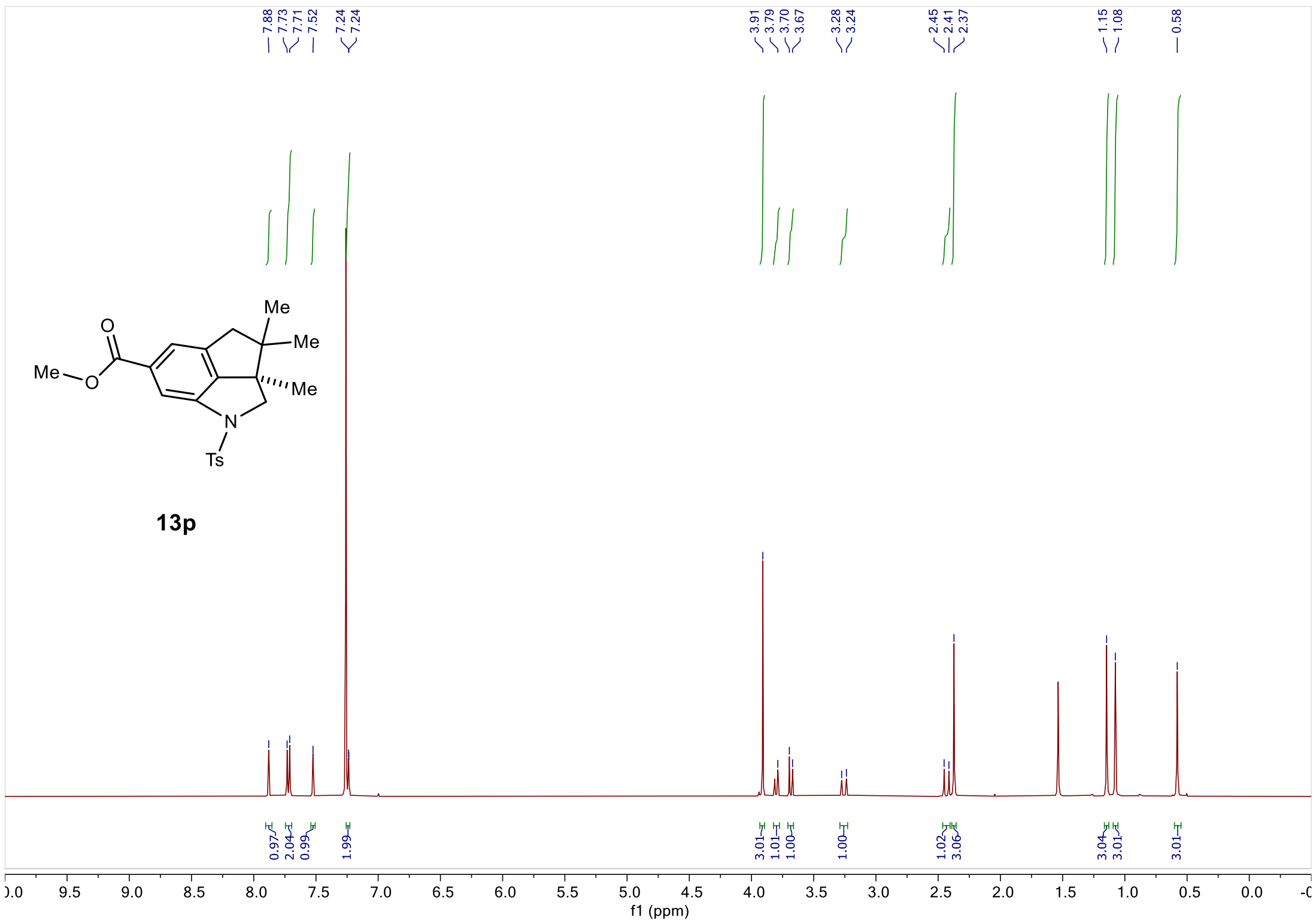


$^{13}C$ NMR (101 MHz, $CDCl_3$)

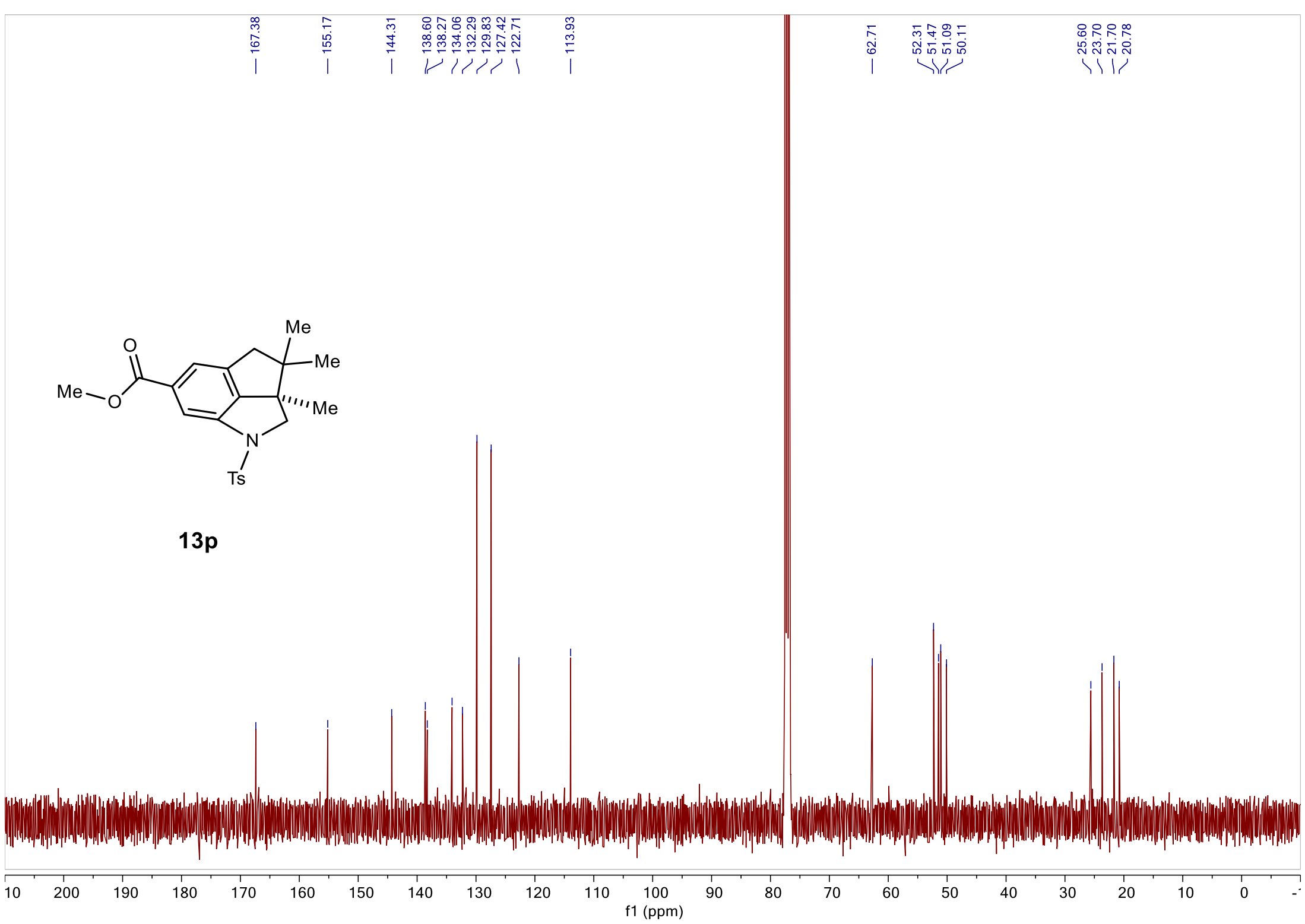

$^{1}H$ NMR (400 MHz, $CDCl_3$)

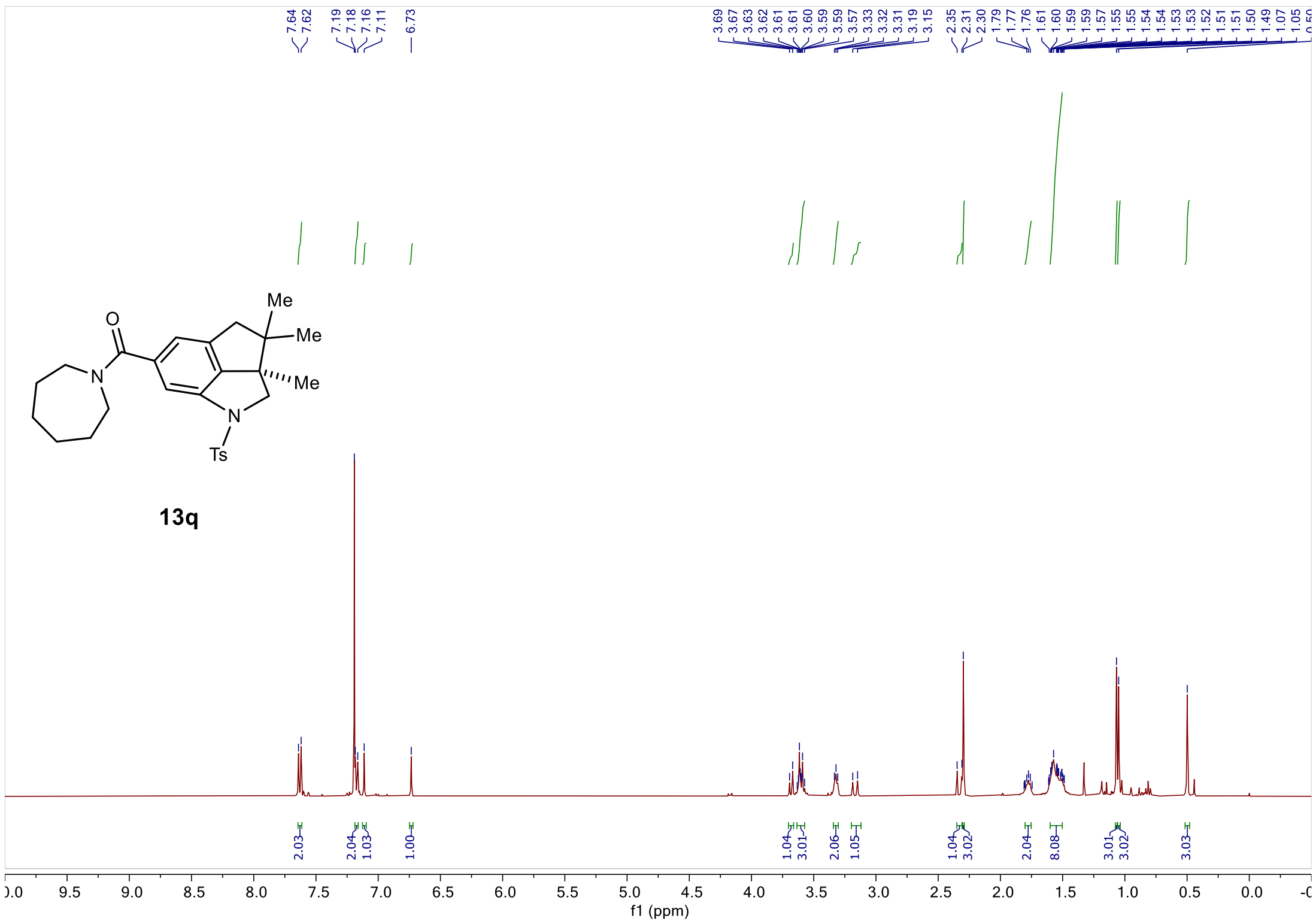


$^{13}C$ NMR (101 MHz, $CDCl_3$)

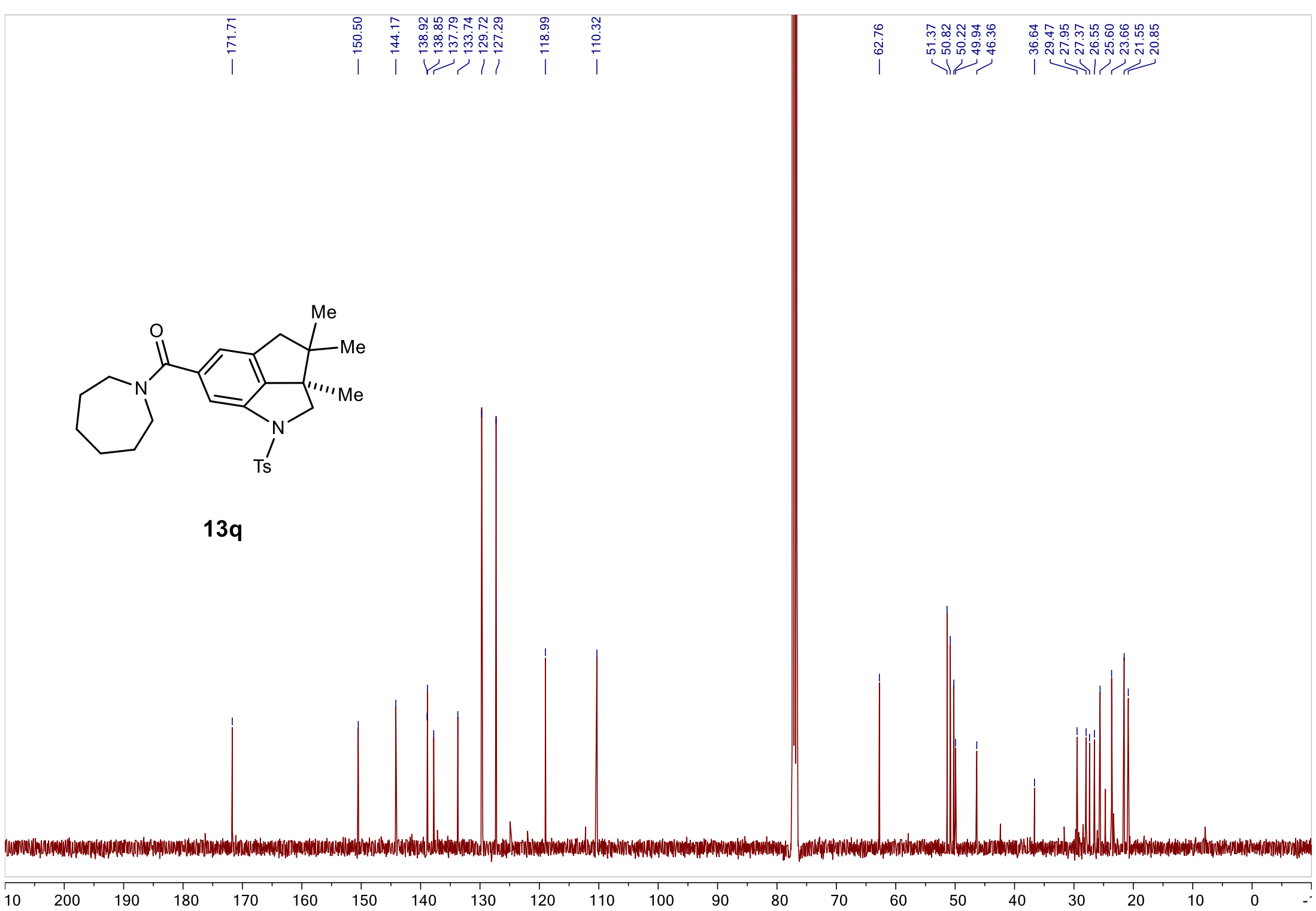

$^1$H NMR (400 MHz, $CDCl_3$)

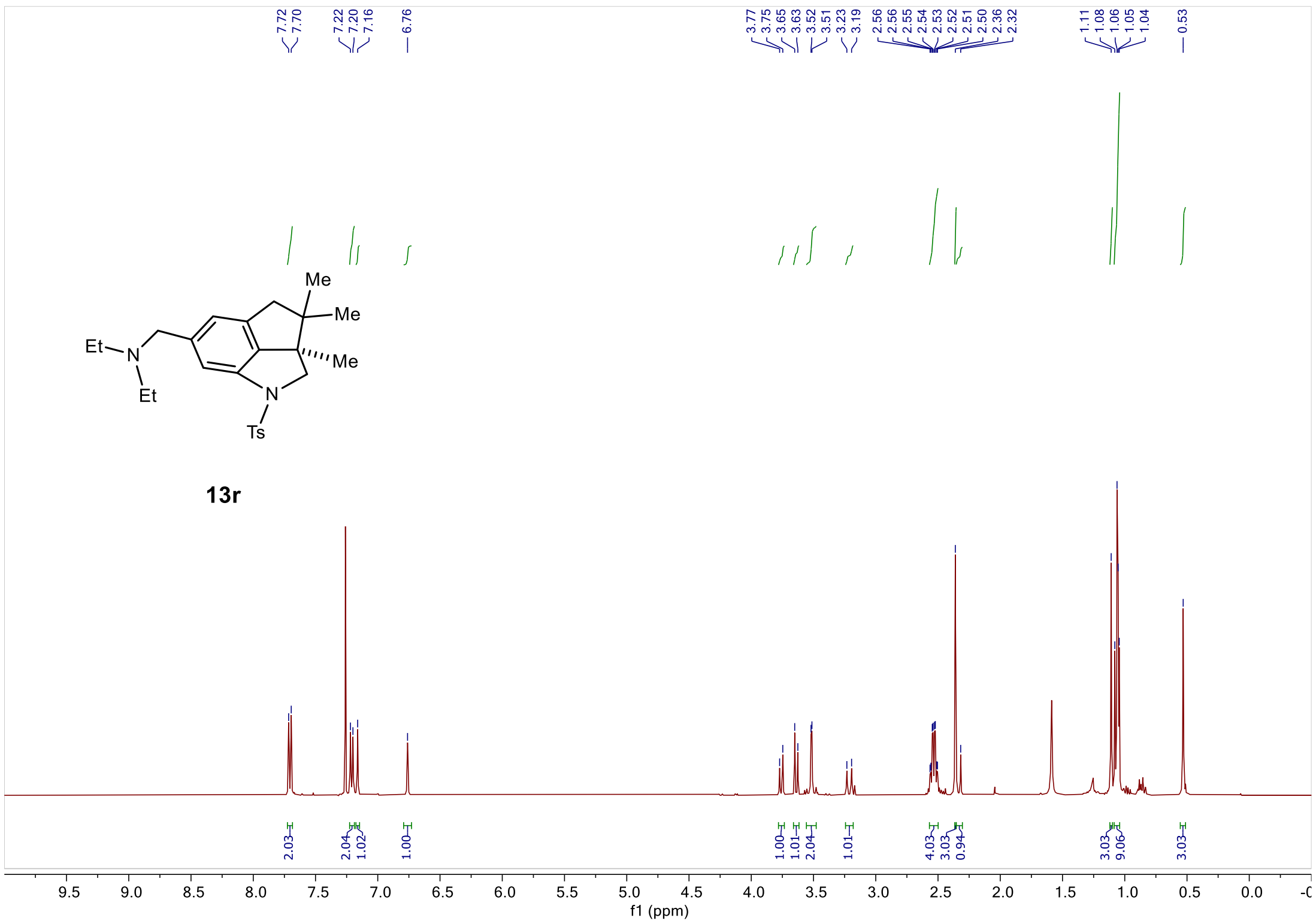


$^{13}$C NMR (101 MHz, $CDCl_3$)

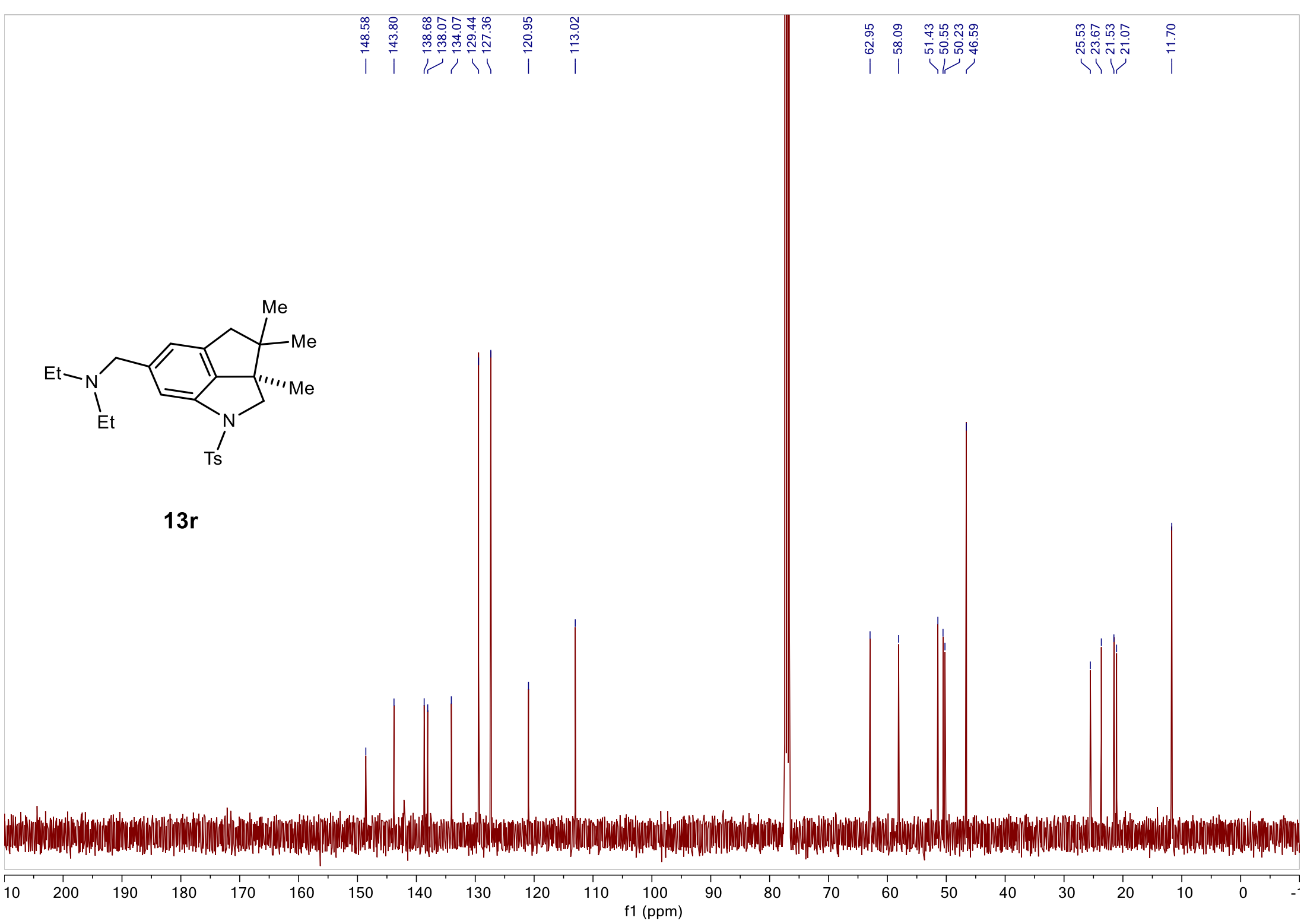

$^{1}H$ NMR (400 MHz, $CDCl_3$)

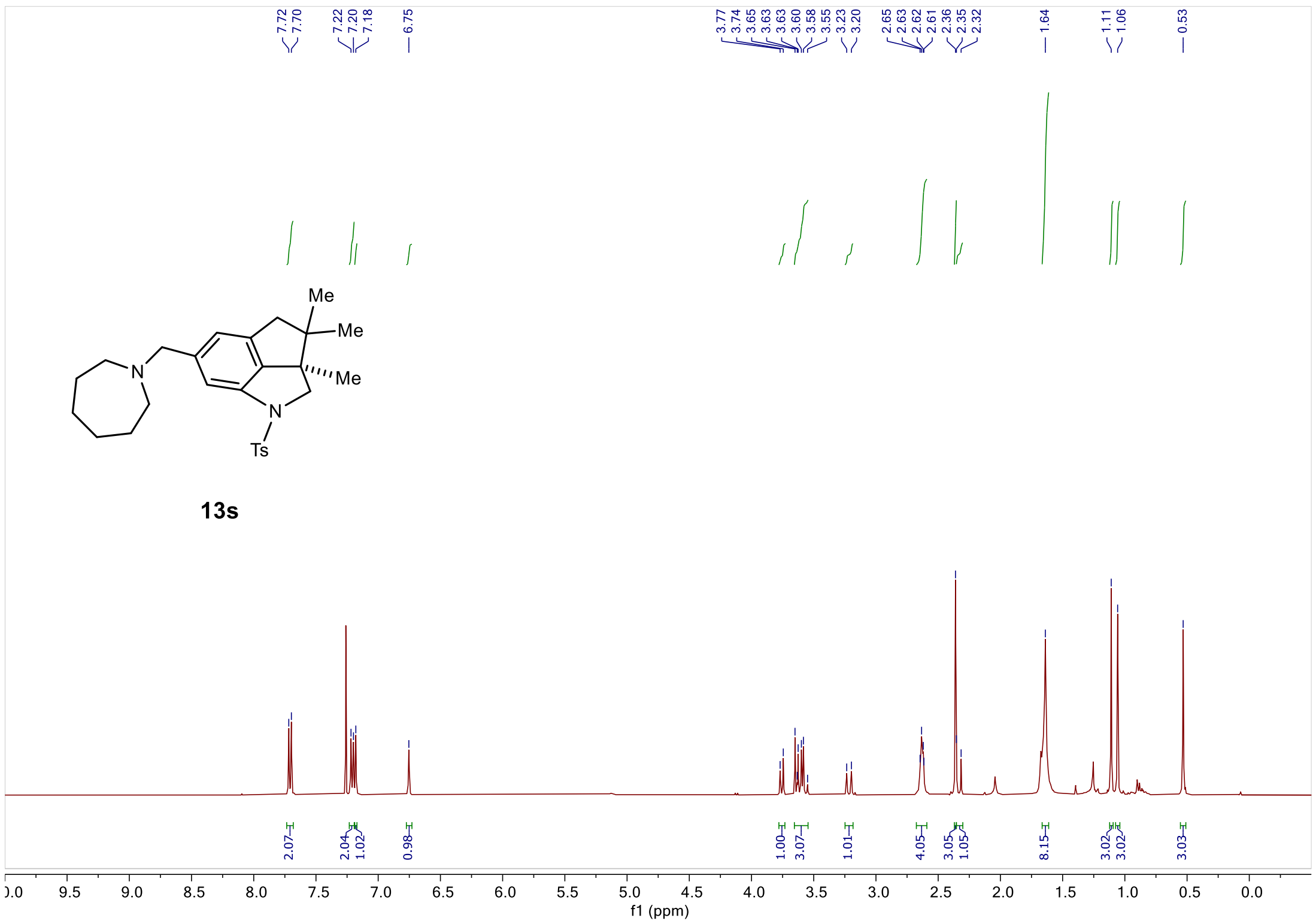

$^{13}C$ NMR (101 MHz, $CDCl_3$)

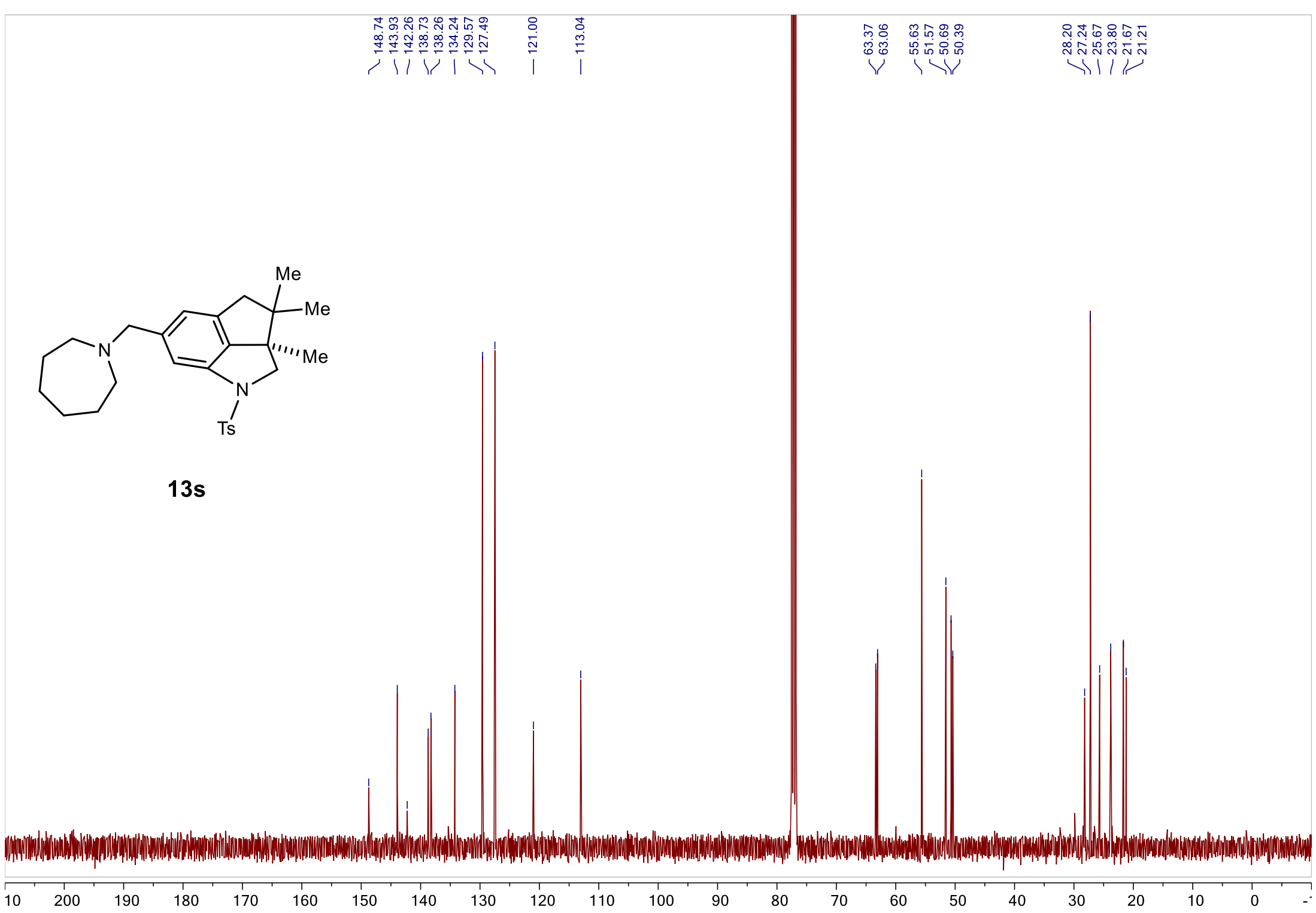

$^1H$ NMR (400 MHz, $CDCl_3$)

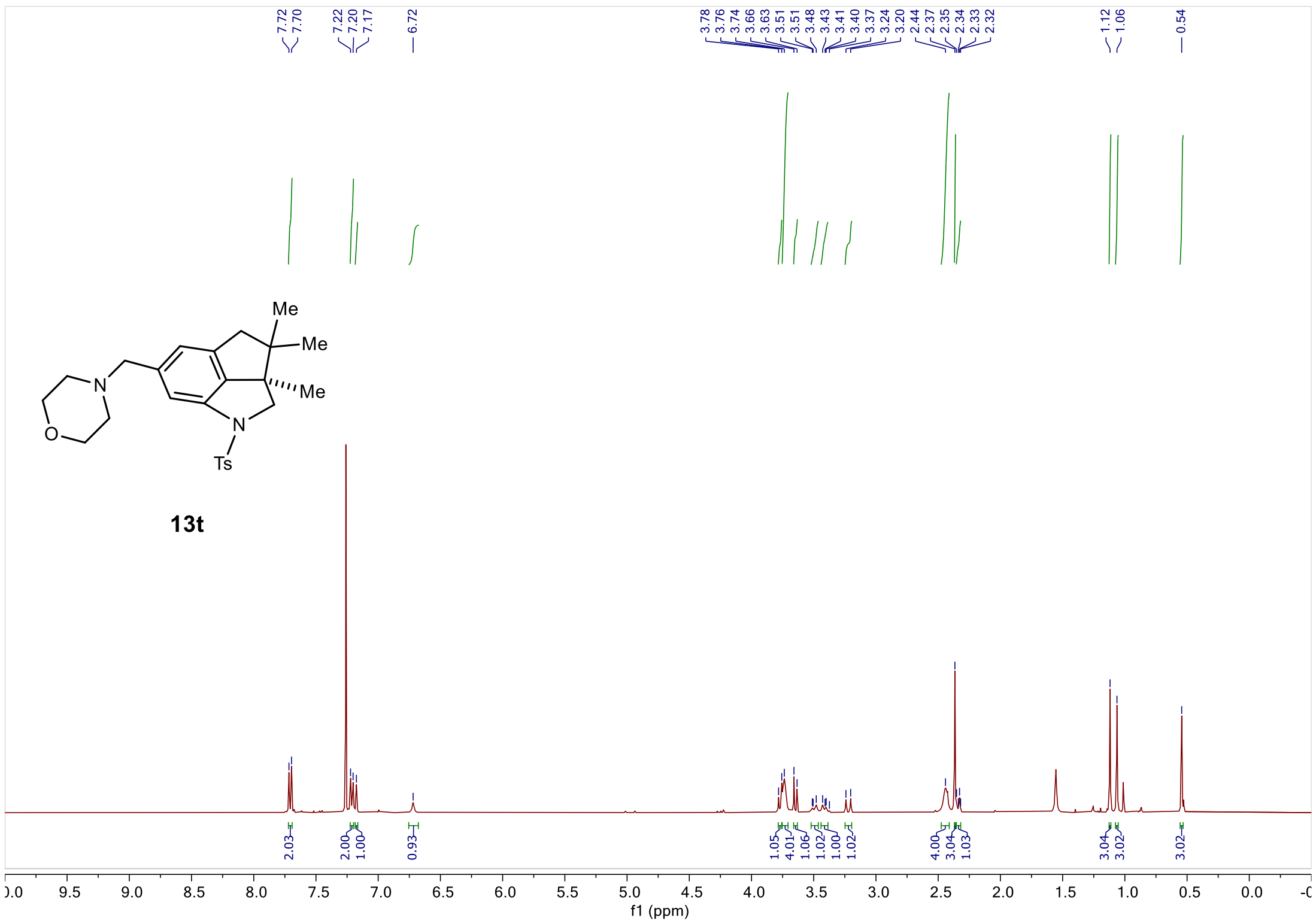

$^{13}C$ NMR (101 MHz, $CDCl_3$)

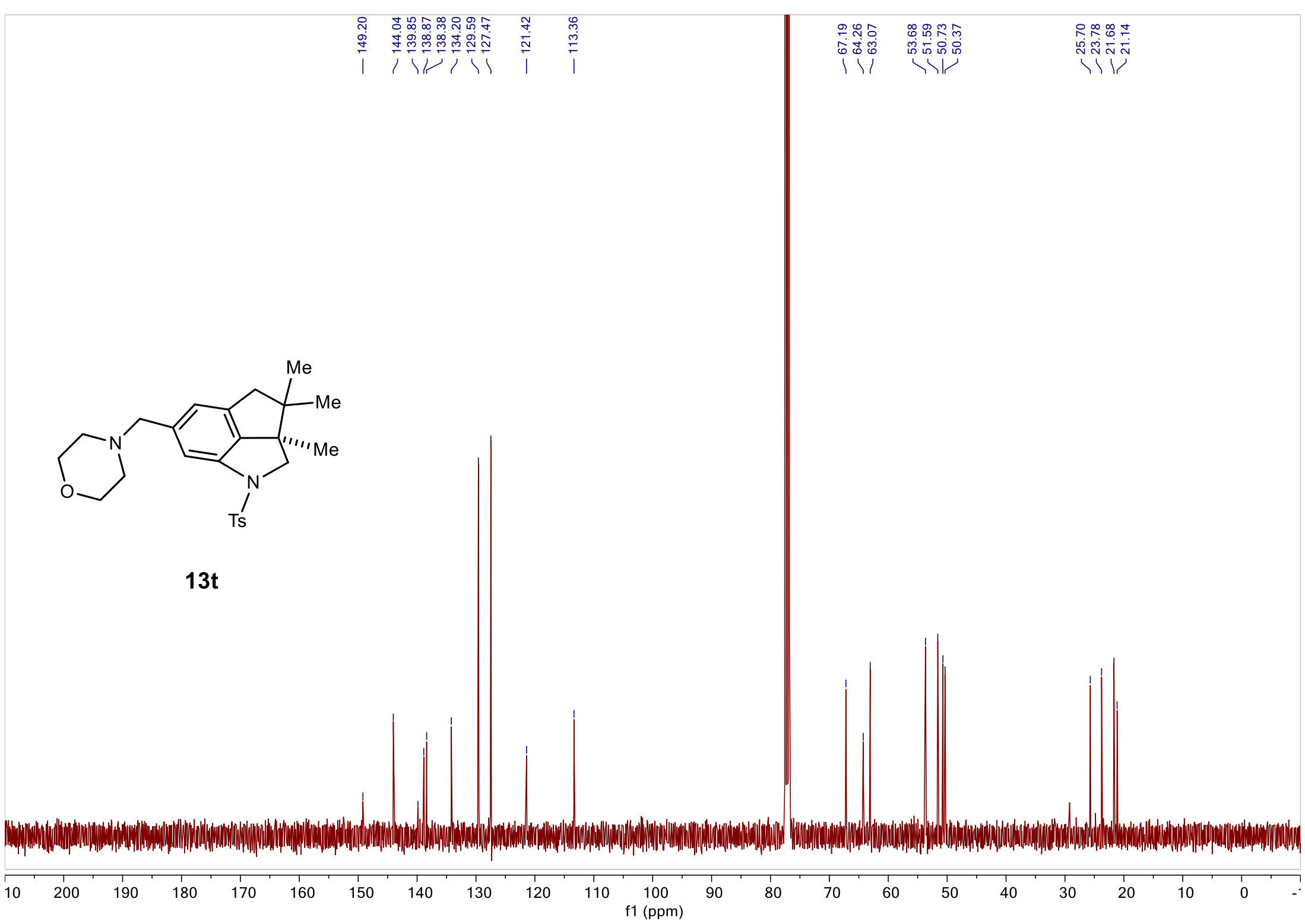

$^{1}H$ NMR (400 MHz, $CDCl_3$)

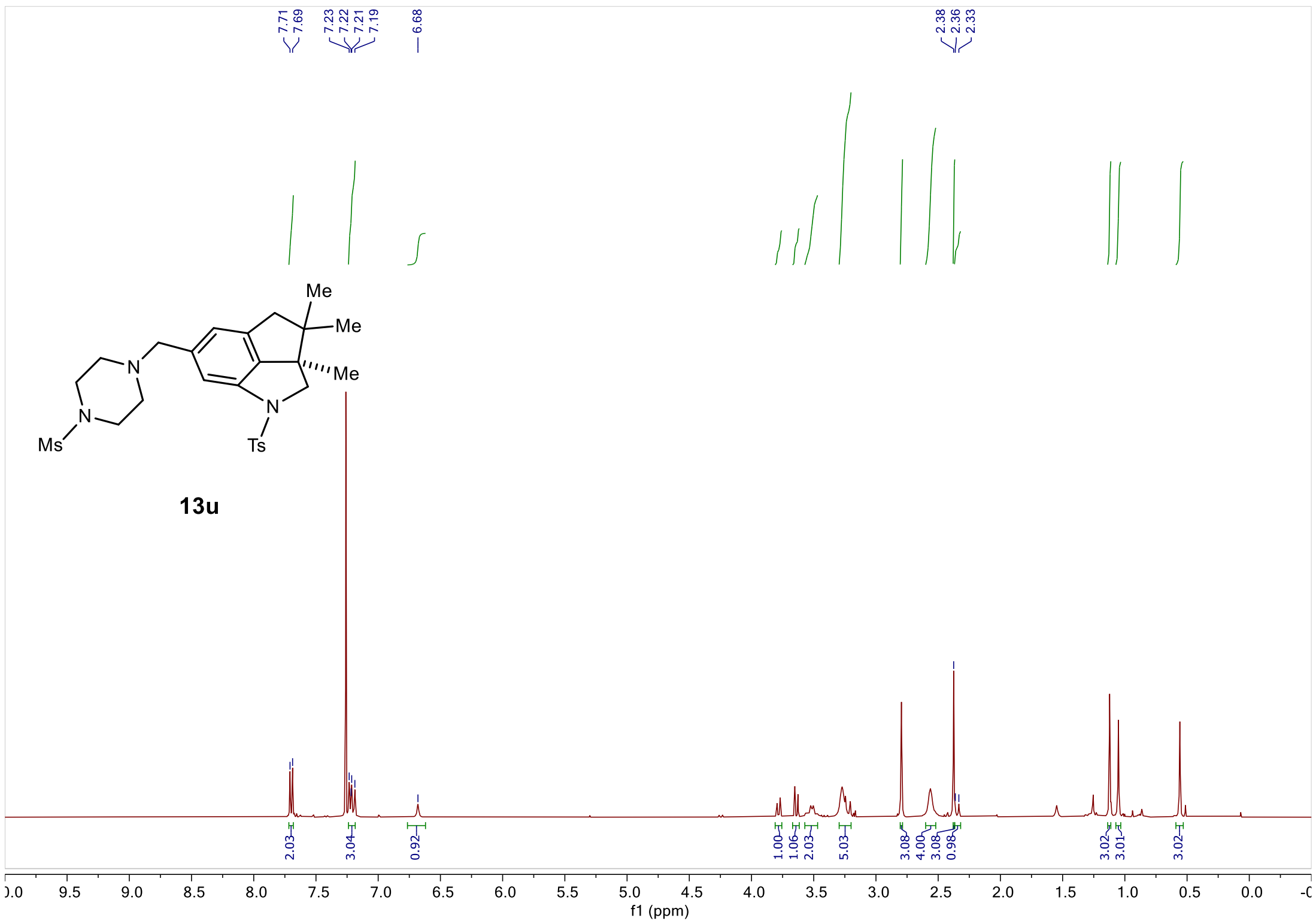


$^{13}C$ NMR (101 MHz, $CDCl_3$)

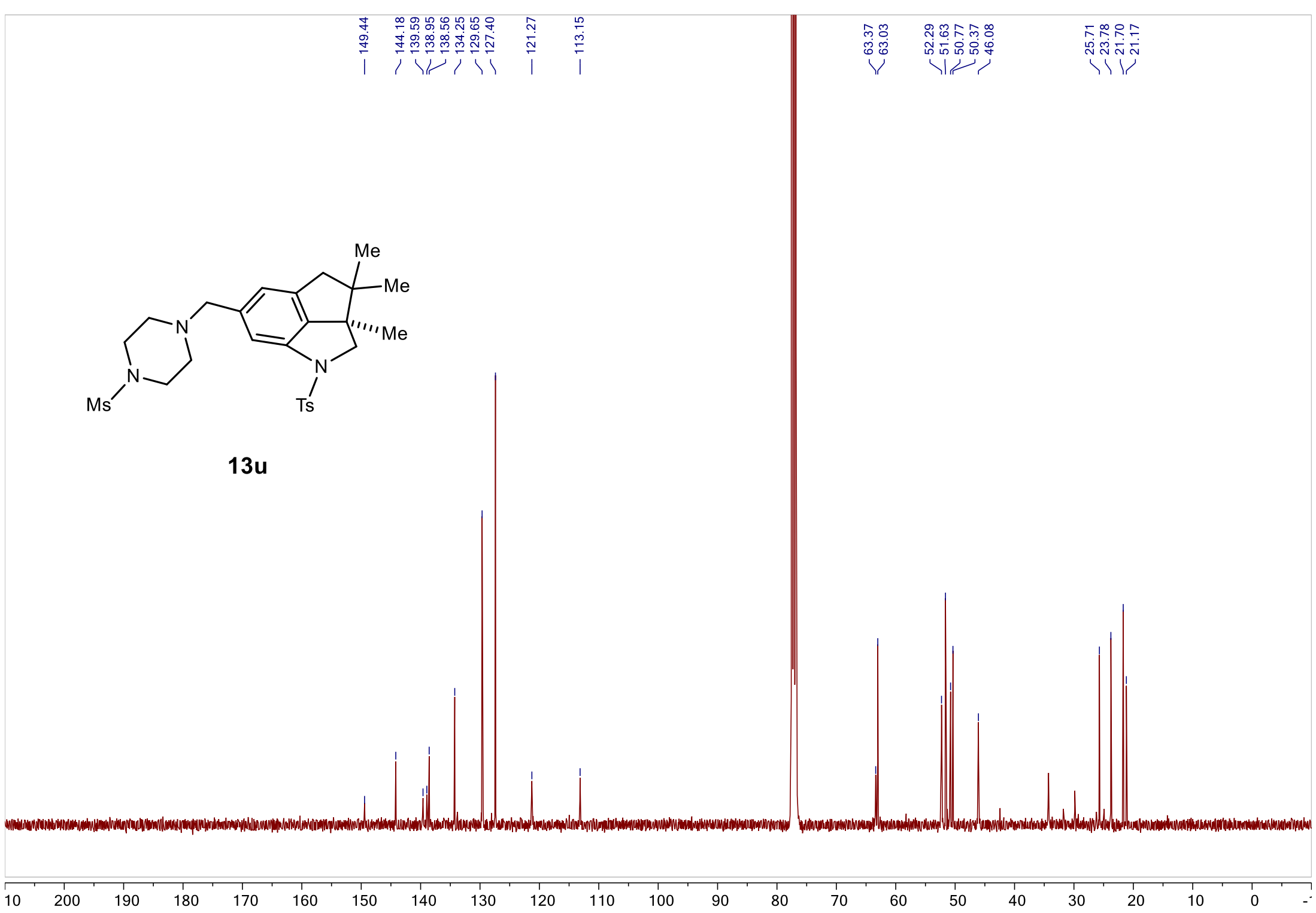

$^{1}H$ NMR (400 MHz, $CDCl_3$)

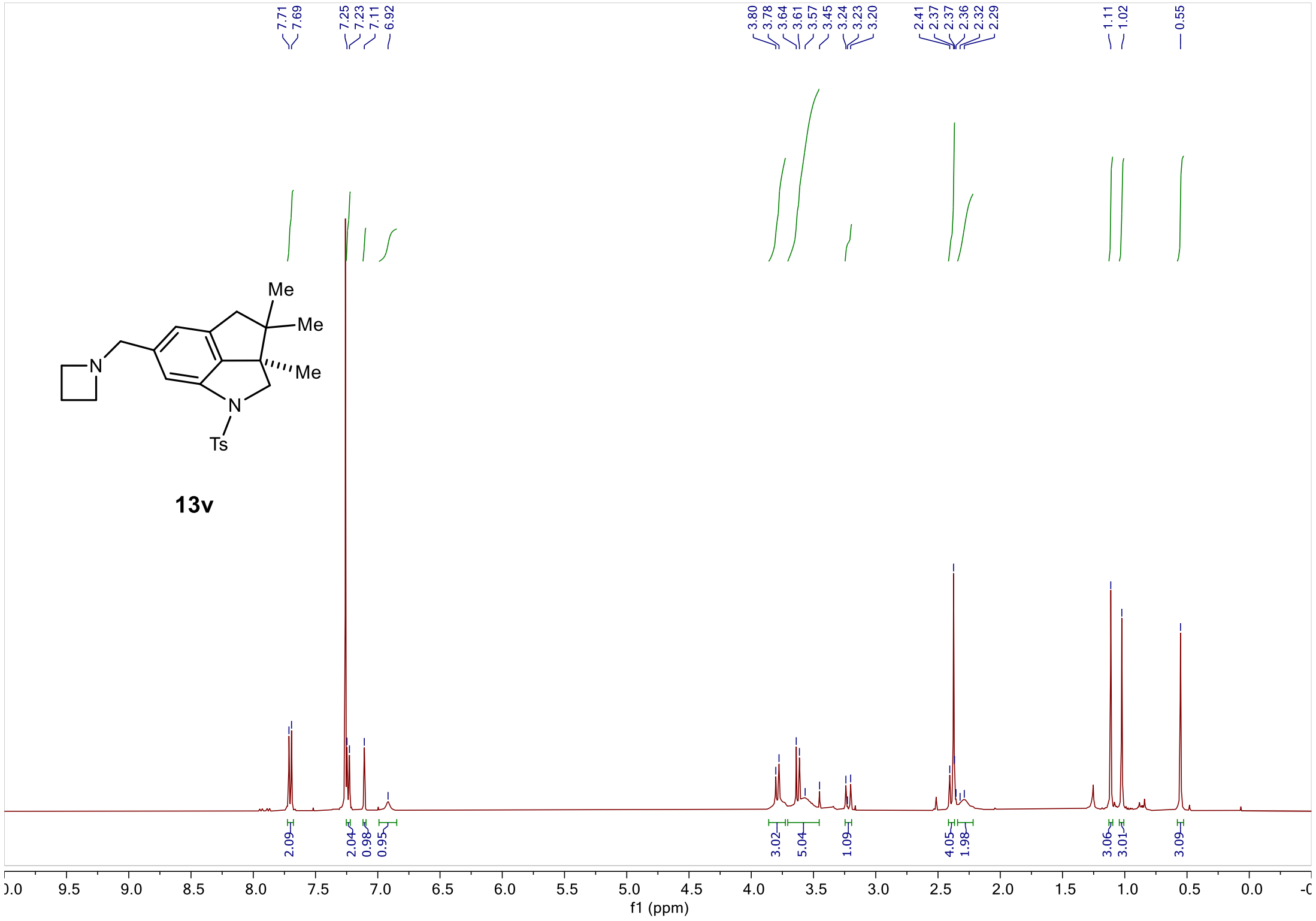


$^{13}C$ NMR (101 MHz, $CDCl_3$)

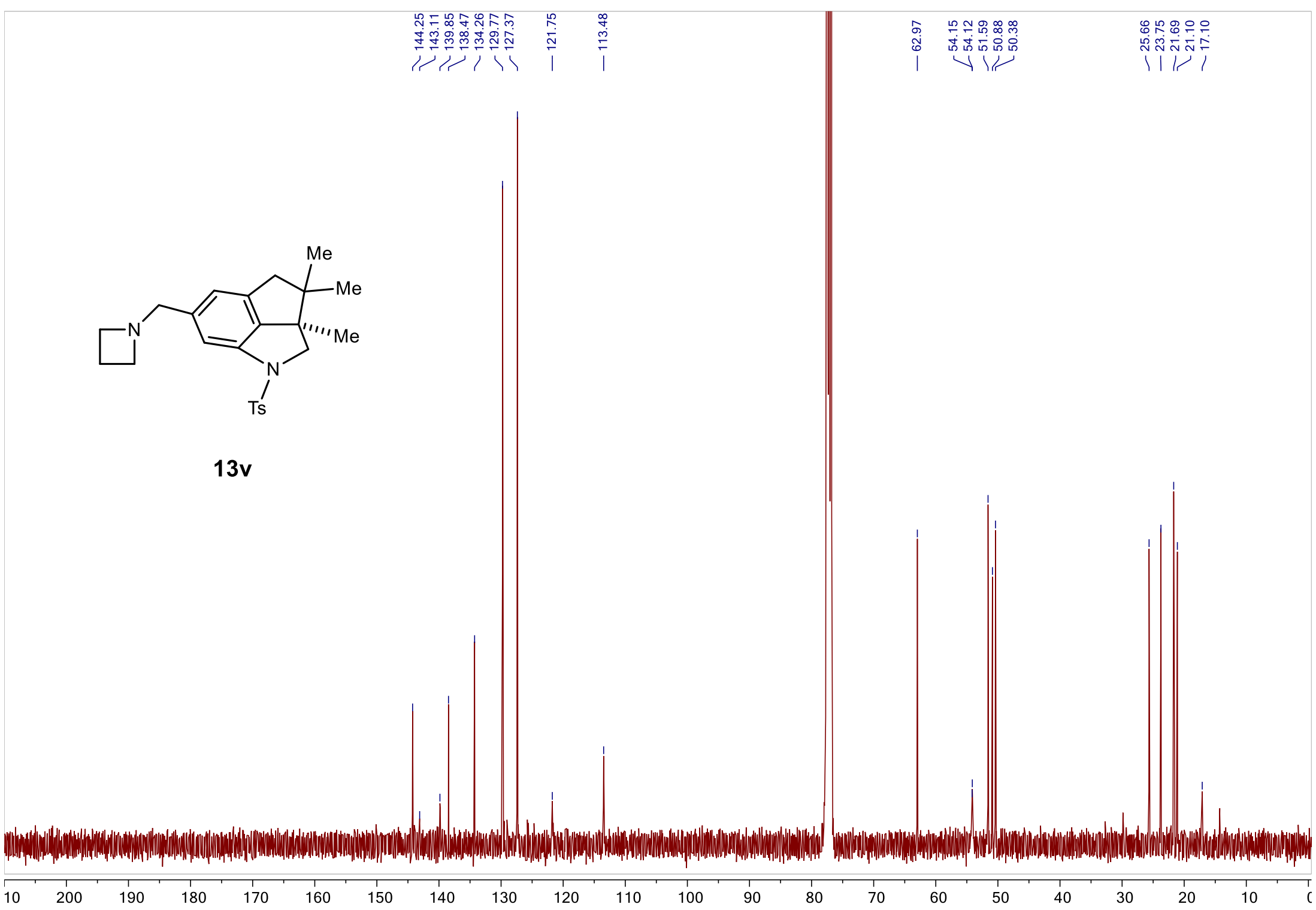

$^{1}H$ NMR (400 MHz, $CDCl_3$)

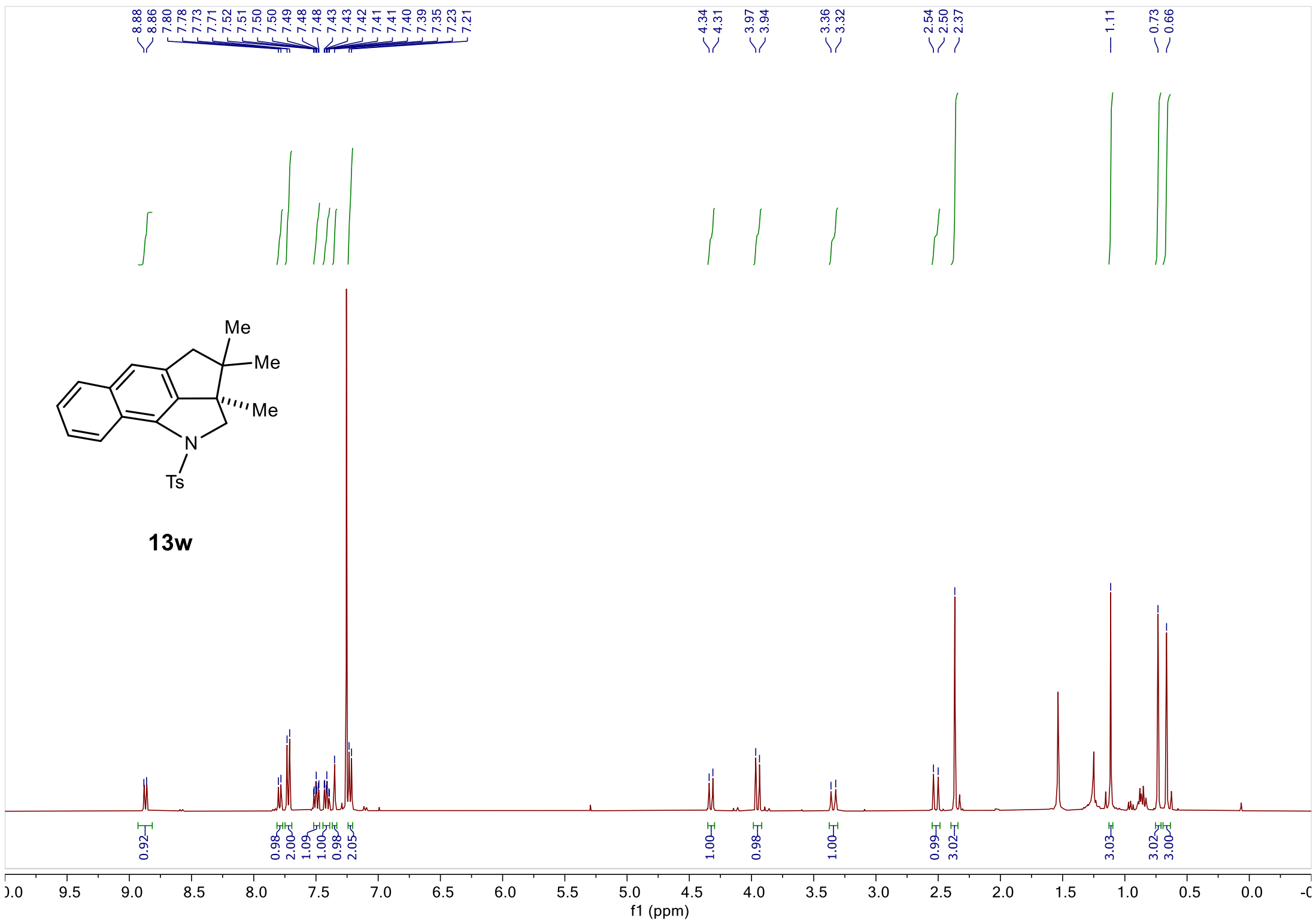


$^{13}C$ NMR (101 MHz, $CDCl_3$)

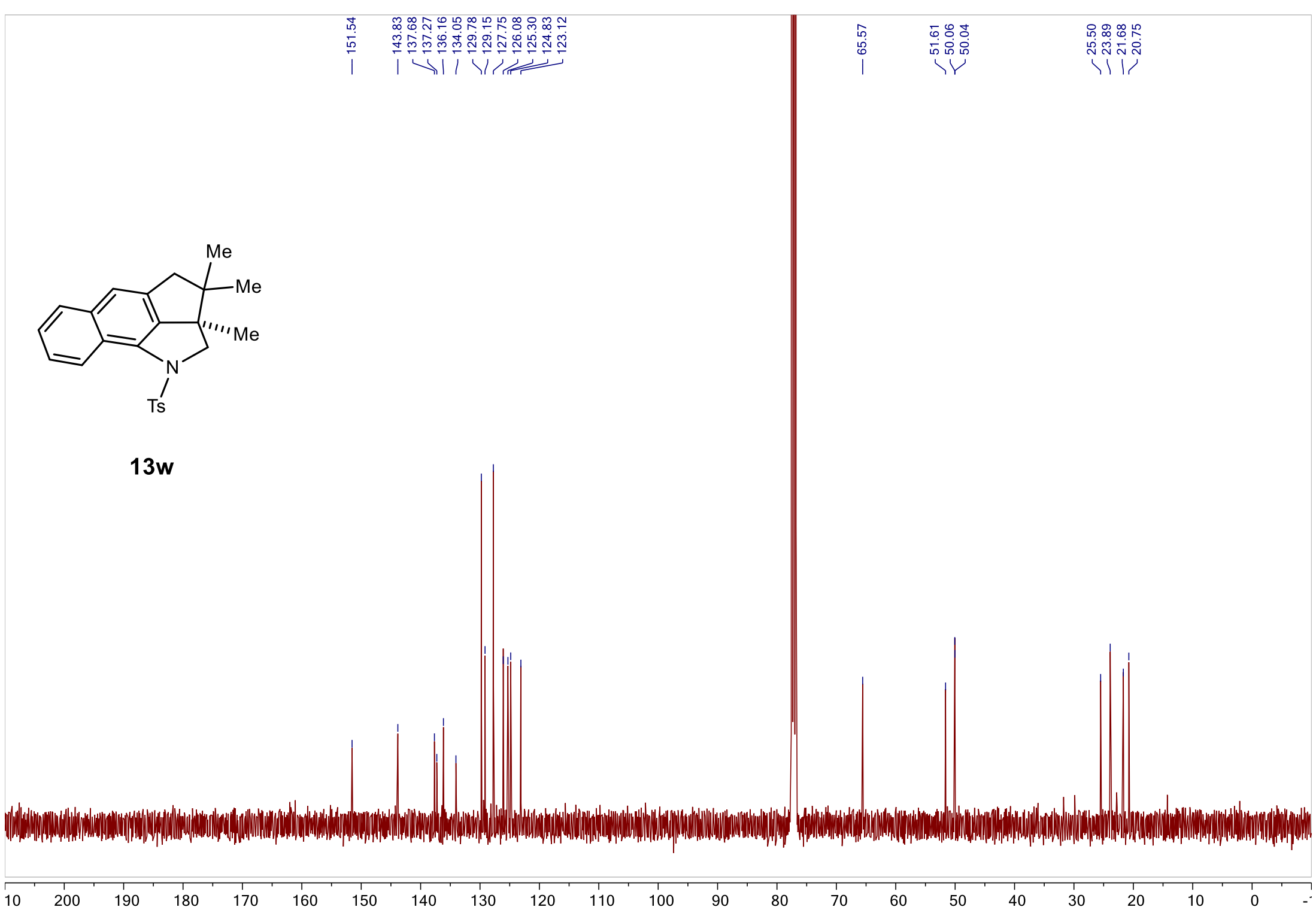

$^{1}H$ NMR (400 MHz, $CDCl_3$)

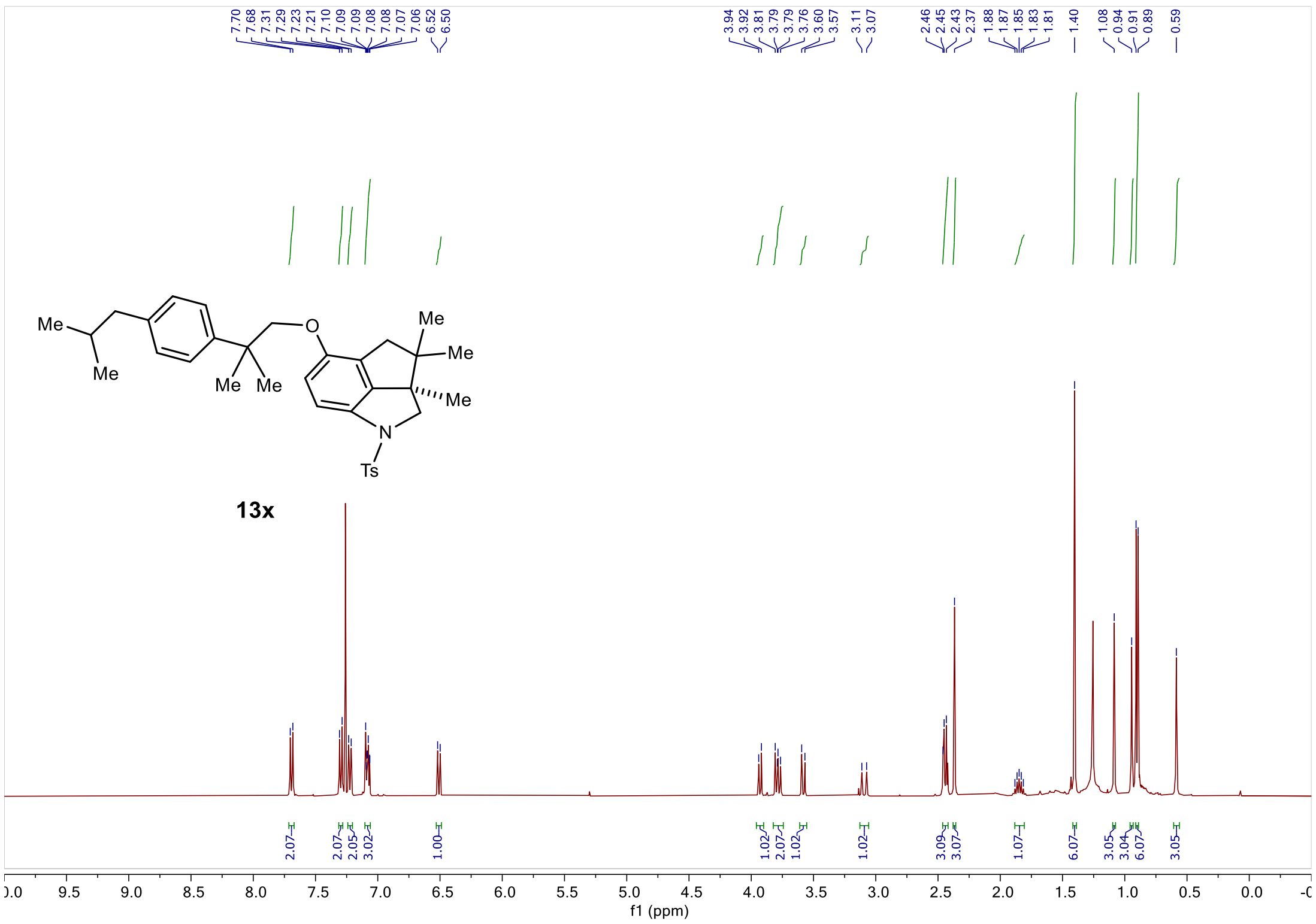

$^{13}C$ NMR (101 MHz, $CDCl_3$)

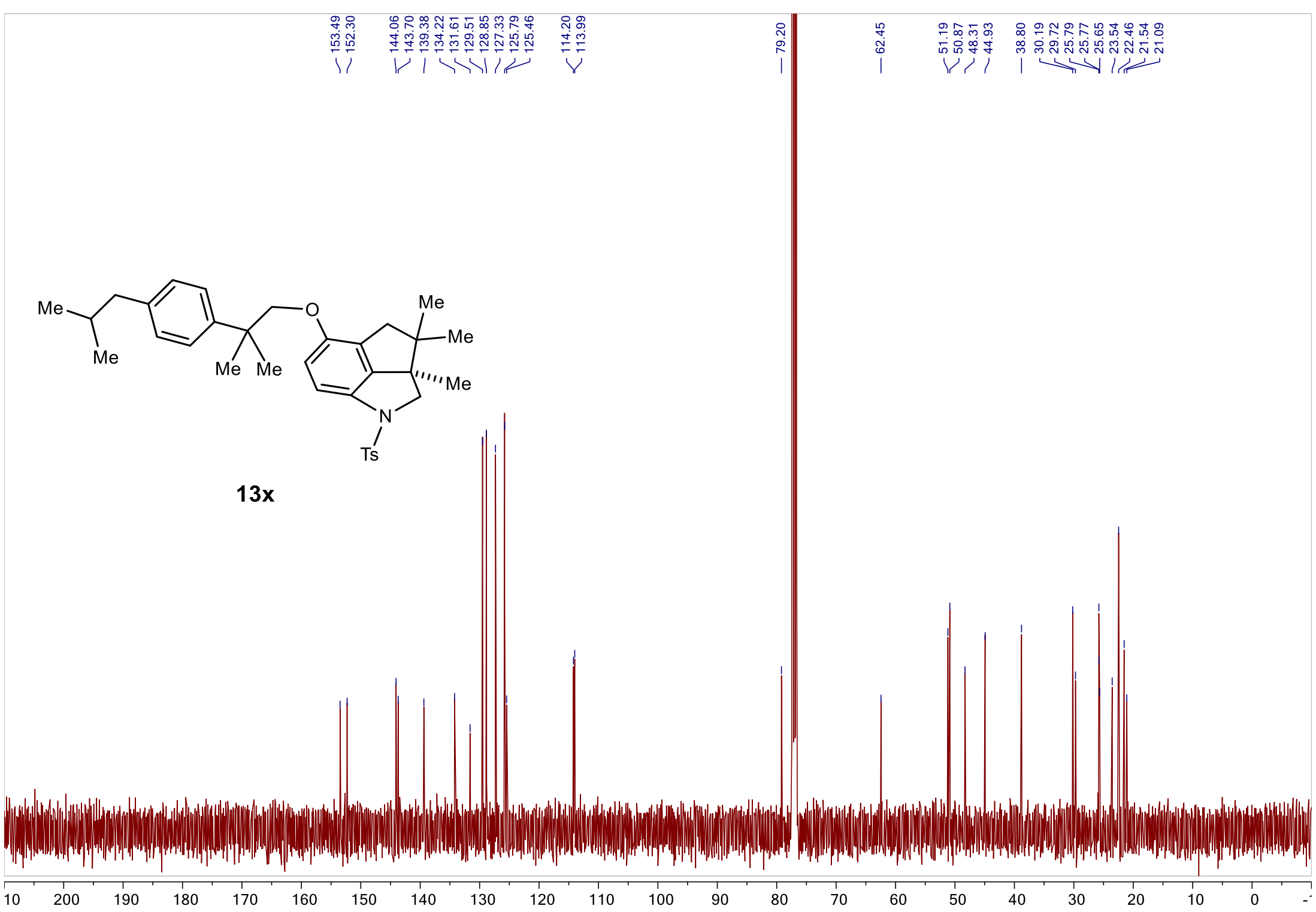

$^{1}H$ NMR (400 MHz, $CDCl_3$)

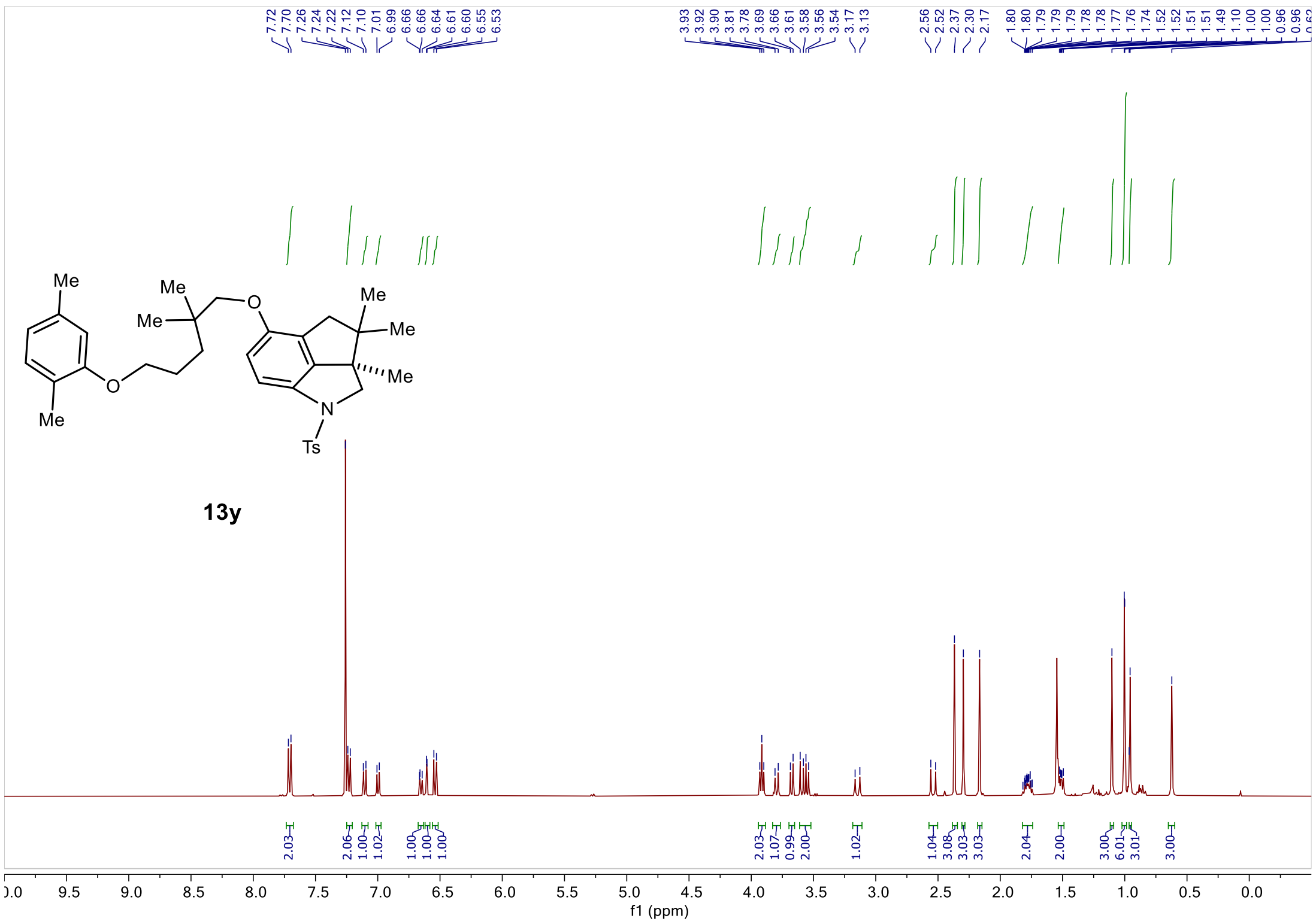


$^{13}C$ NMR (101 MHz, $CDCl_3$)

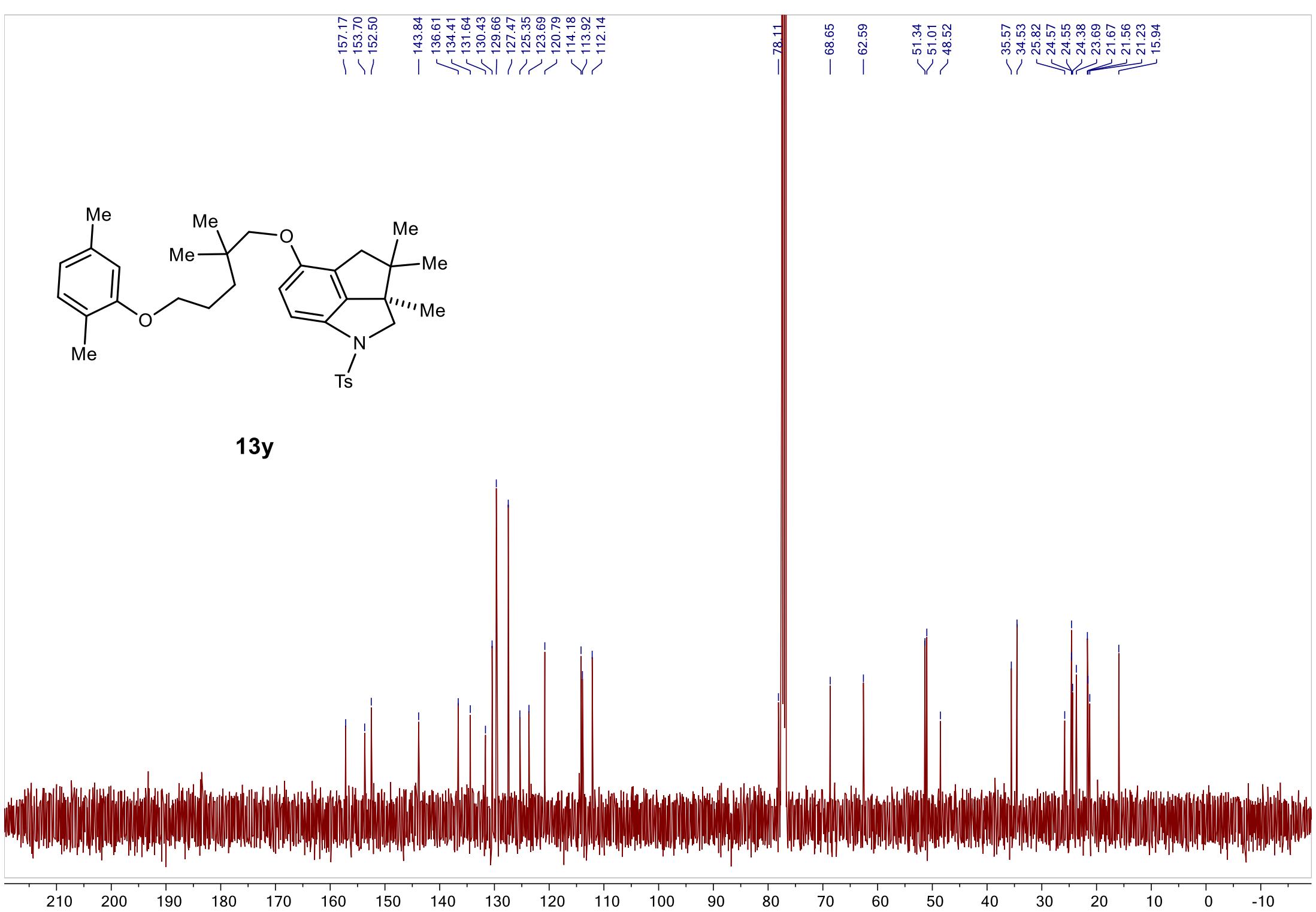

$^{1}H$ NMR (400 MHz, $CDCl_3$)

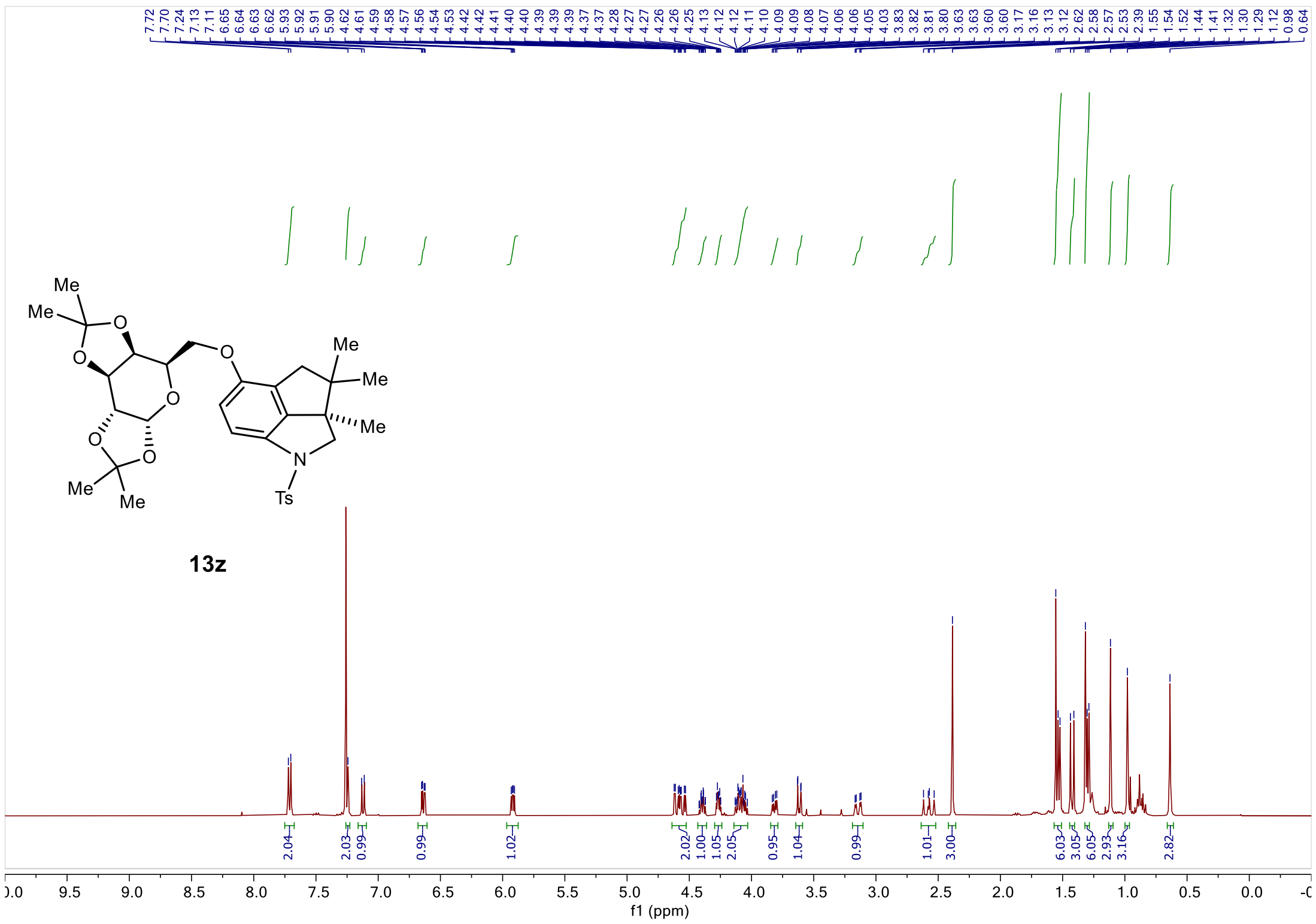


$^{13}C$ NMR (101 MHz, $CDCl_3$)

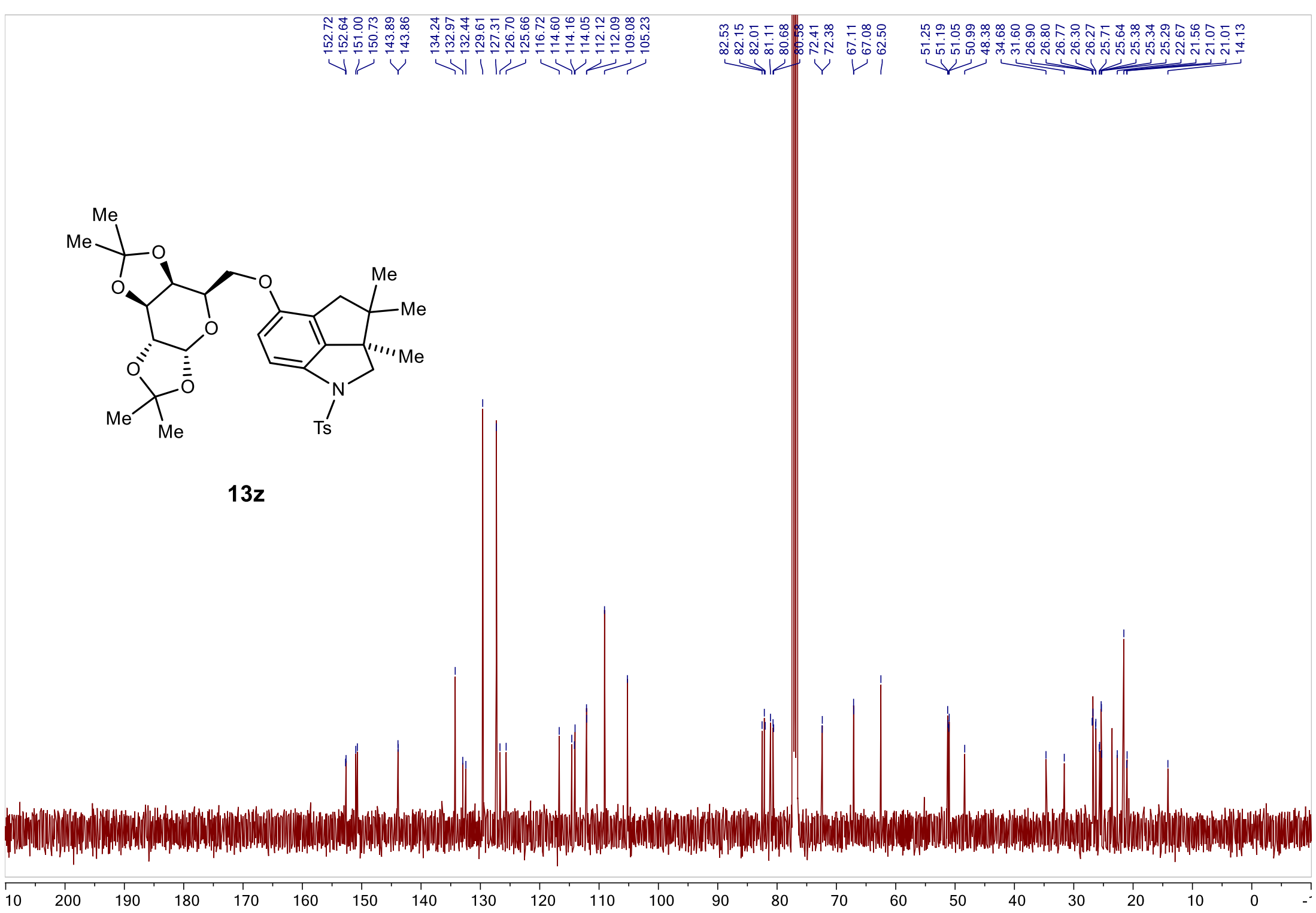

$^1$H NMR (400 MHz, $CDCl_3$)

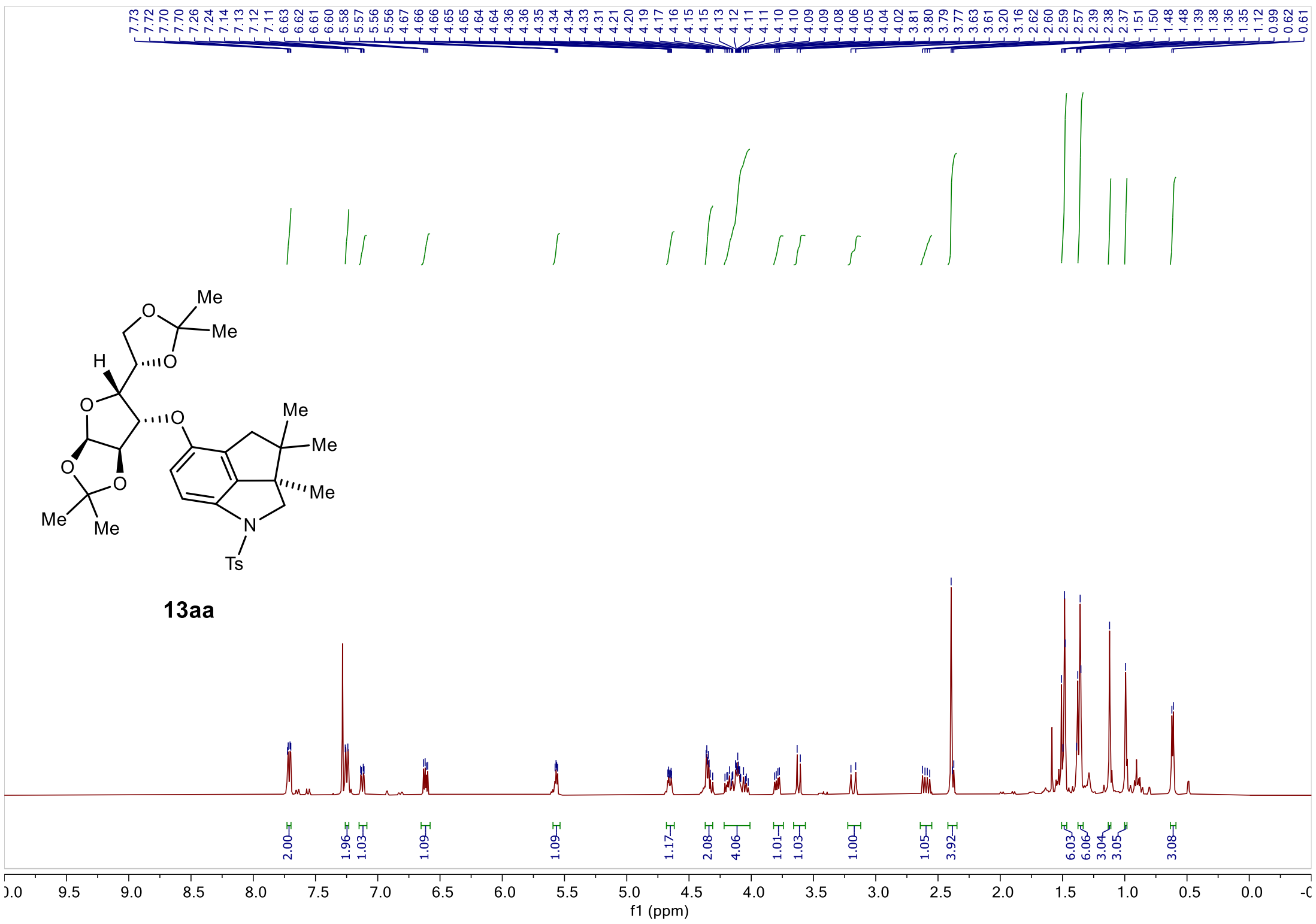

$^{13}$C NMR (101 MHz, $CDCl_3$)

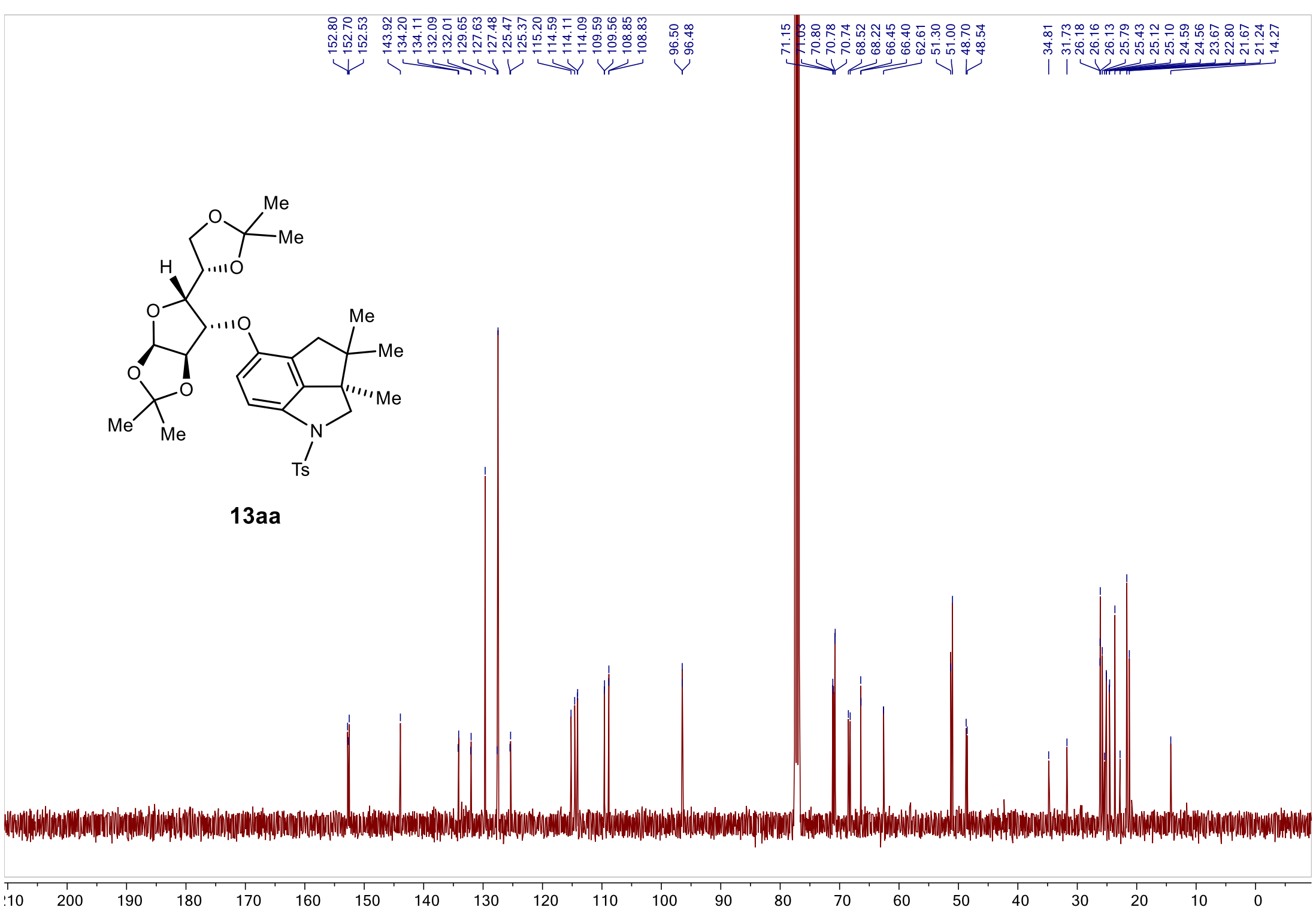

$^{1}H$ NMR (400 MHz, $CDCl_3$)

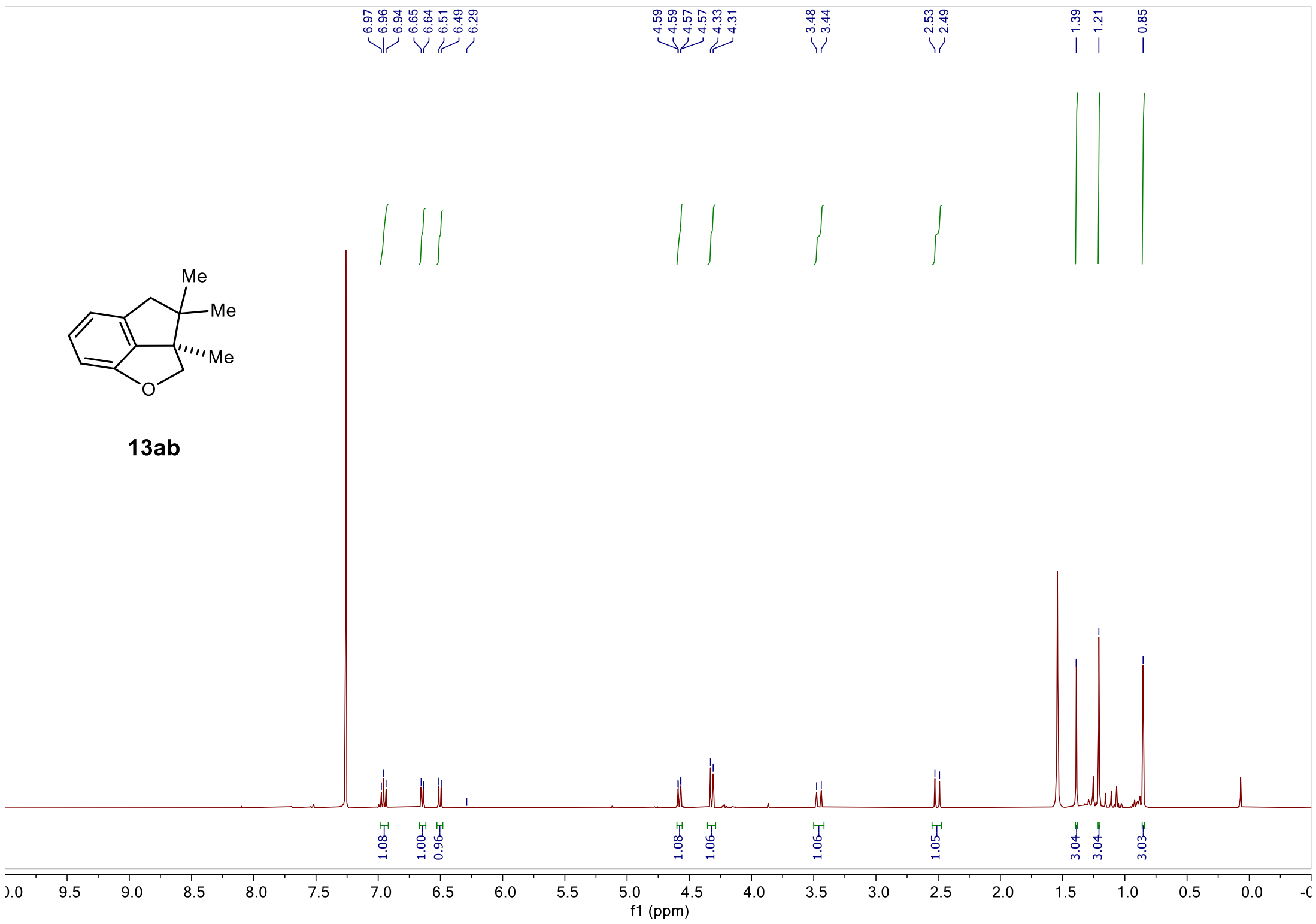


$^{13}C$ NMR (101 MHz, $CDCl_3$)

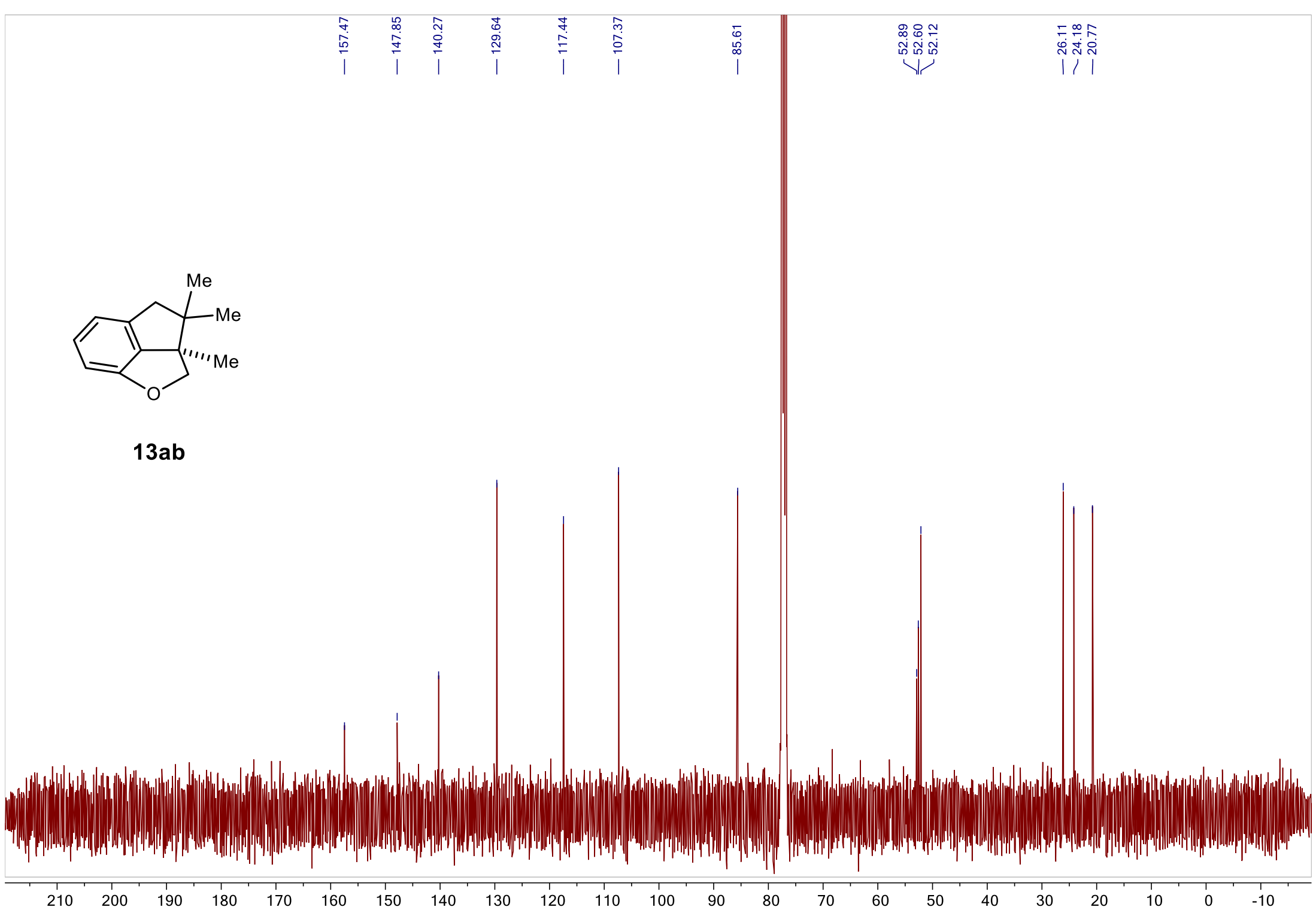

$^{1}H$ NMR (400 MHz, $CDCl_3$)

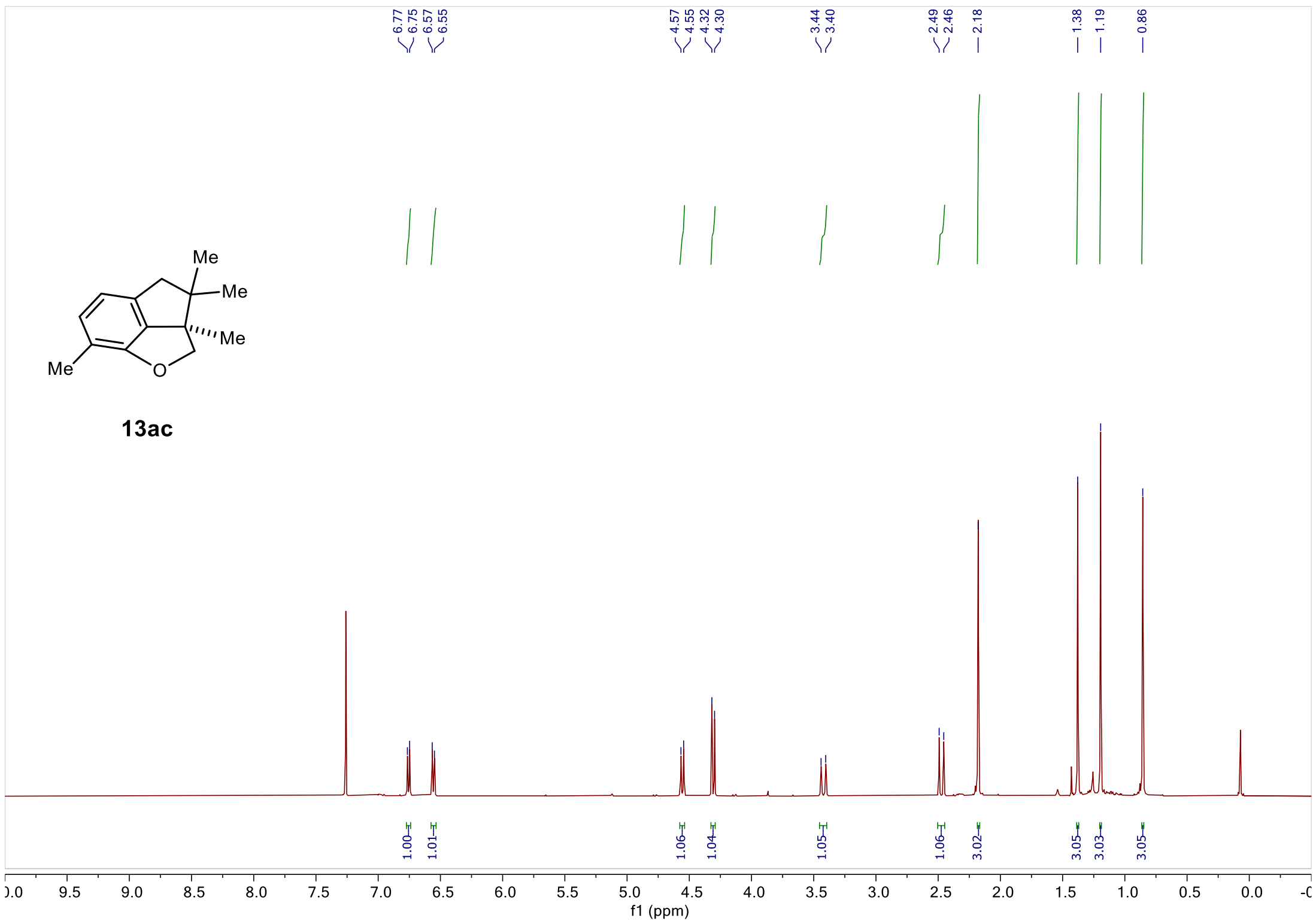

$^{13}C$ NMR (101 MHz, $CDCl_3$)

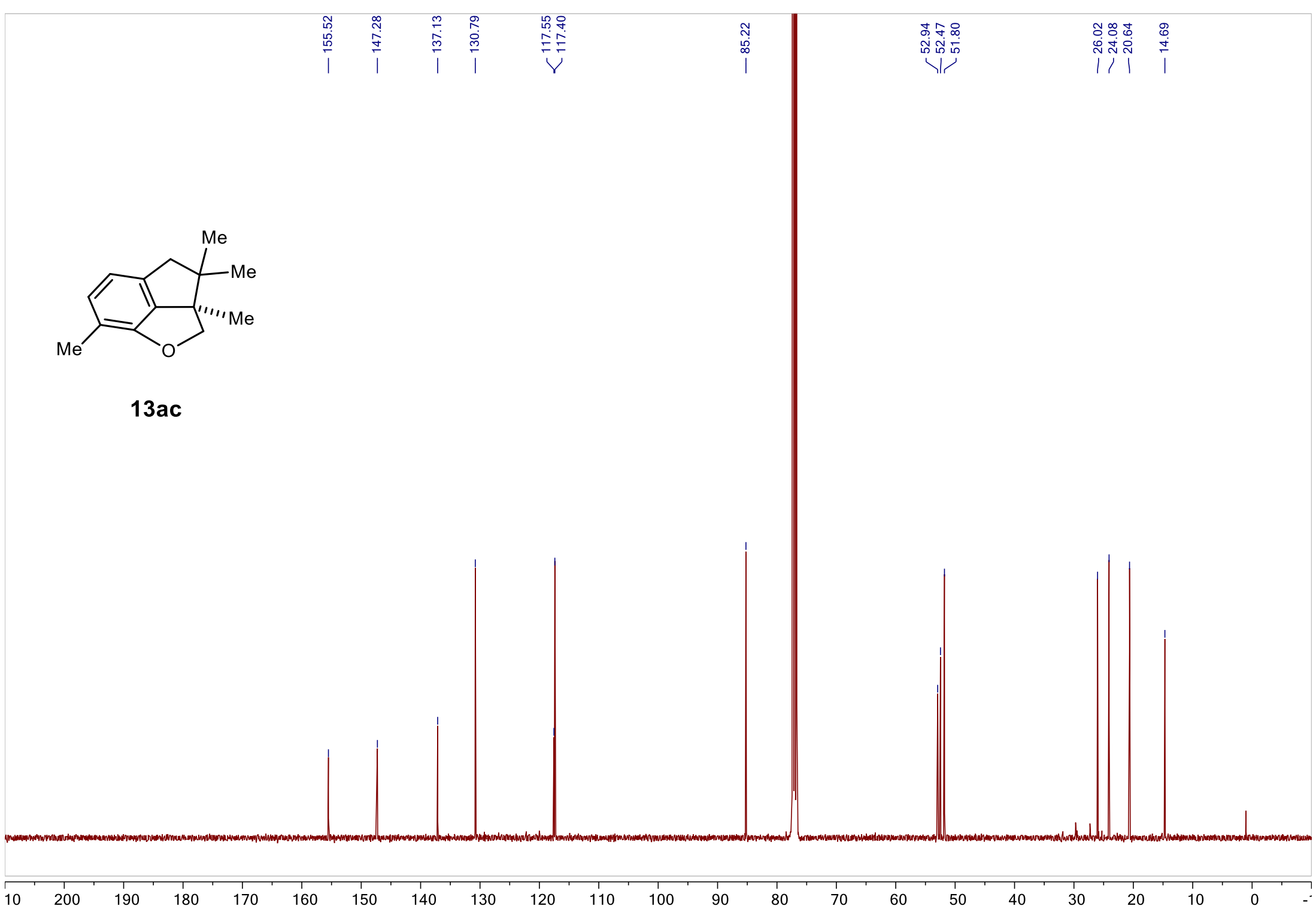

$^{1}H$ NMR (400 MHz, $CDCl_3$)

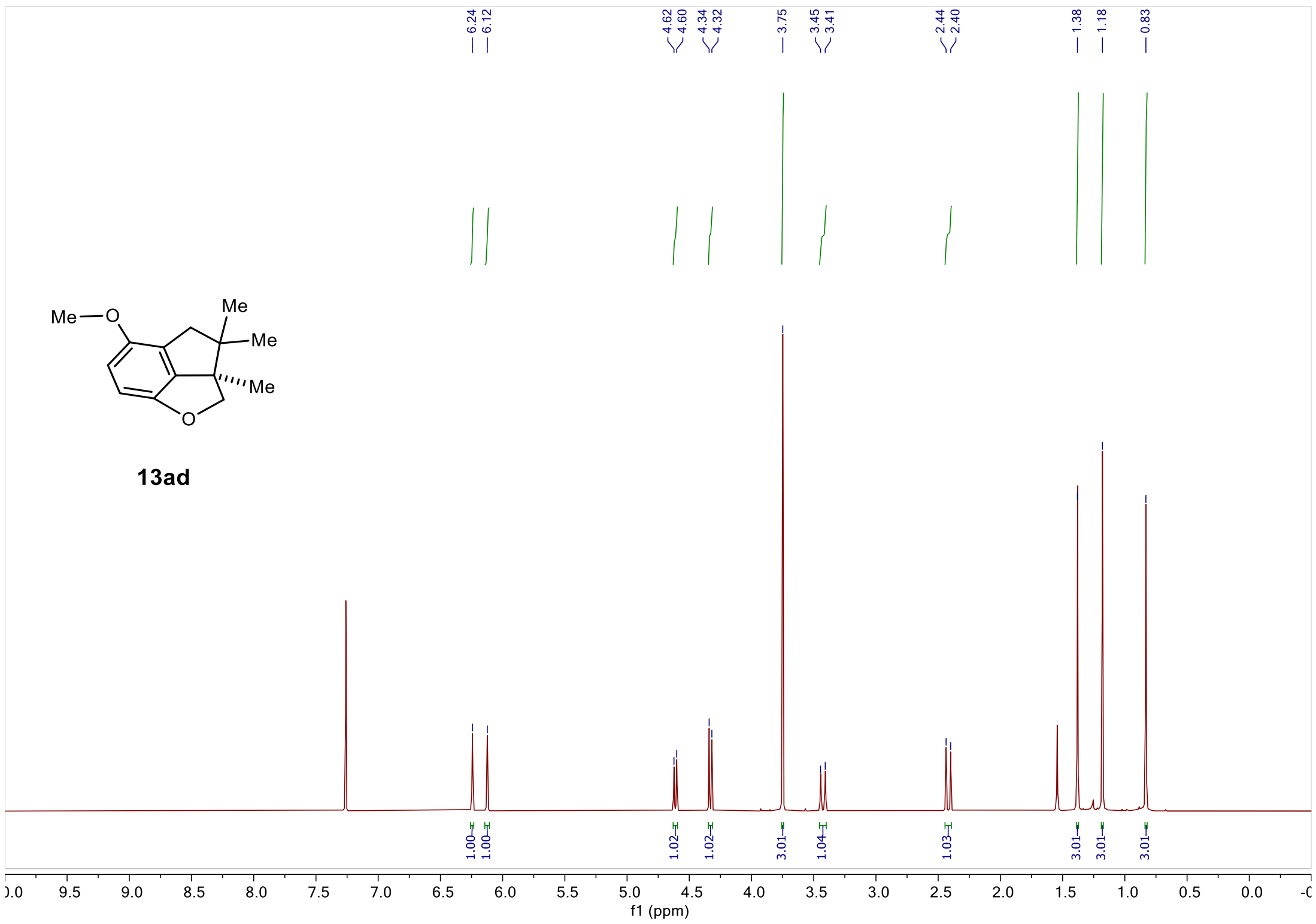


$^{13}C$ NMR (101 MHz, $CDCl_3$)

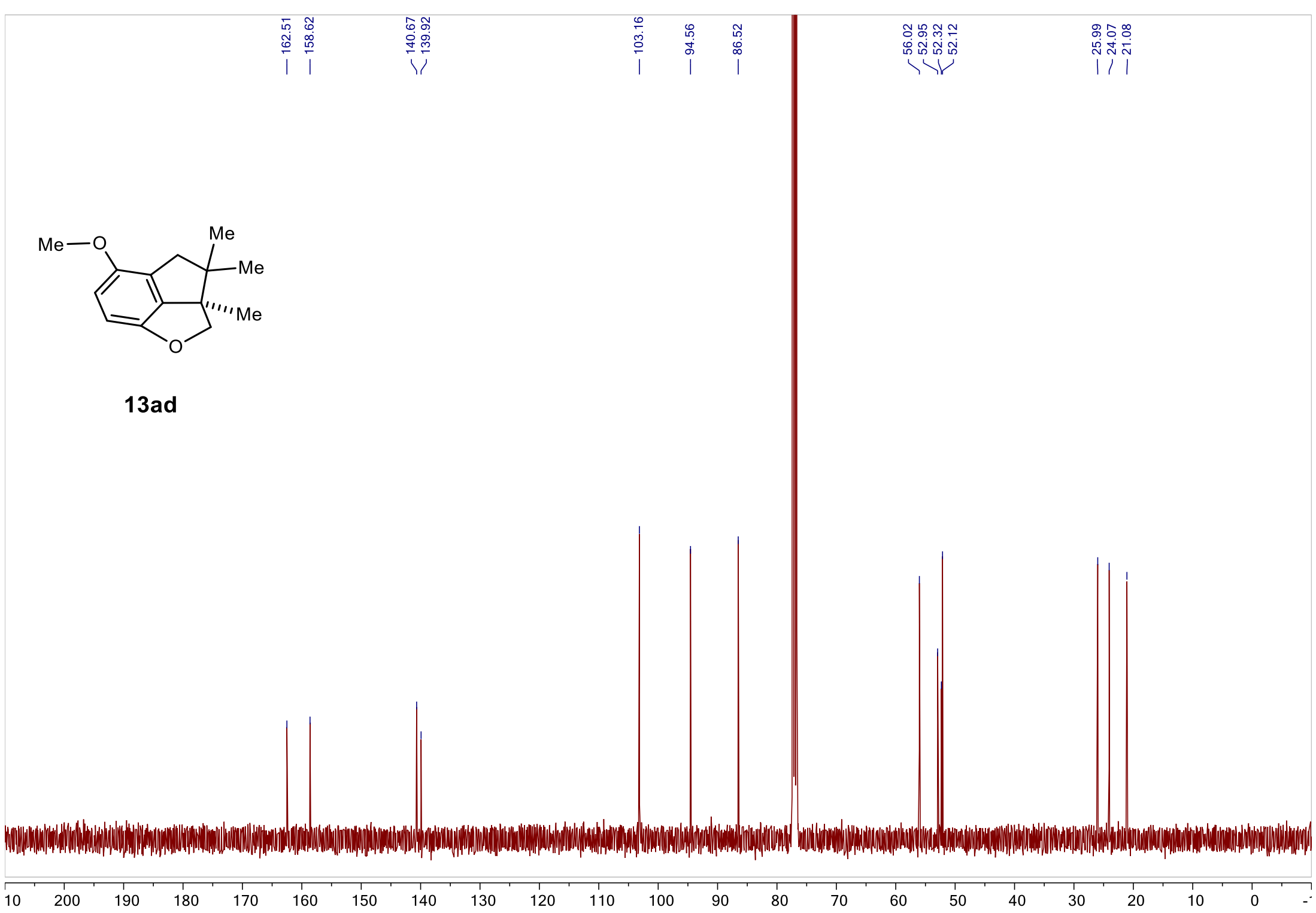

$^{1}H$ NMR (400 MHz, $CDCl_3$)

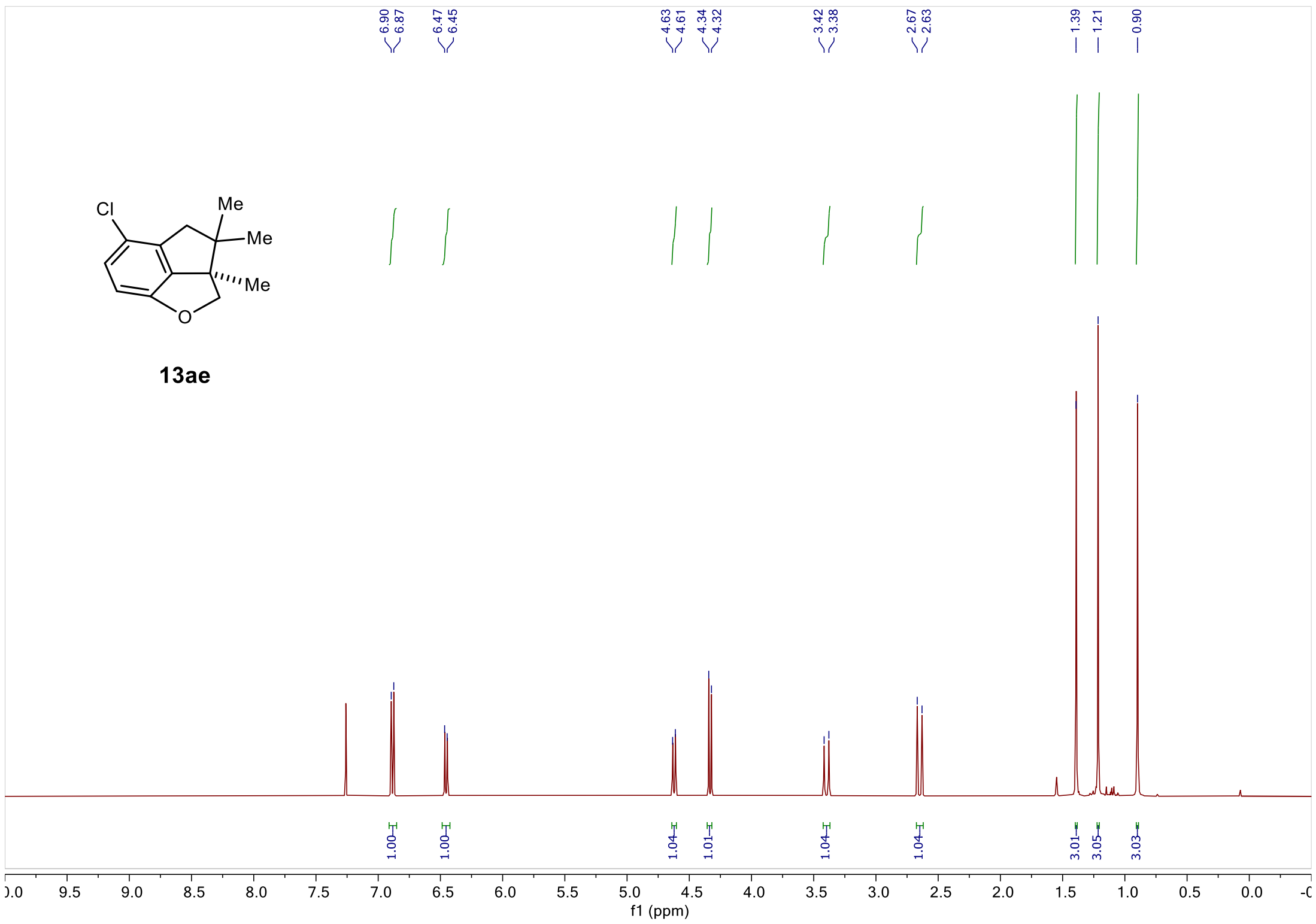


$^{13}C$ NMR (101 MHz, $CDCl_3$)

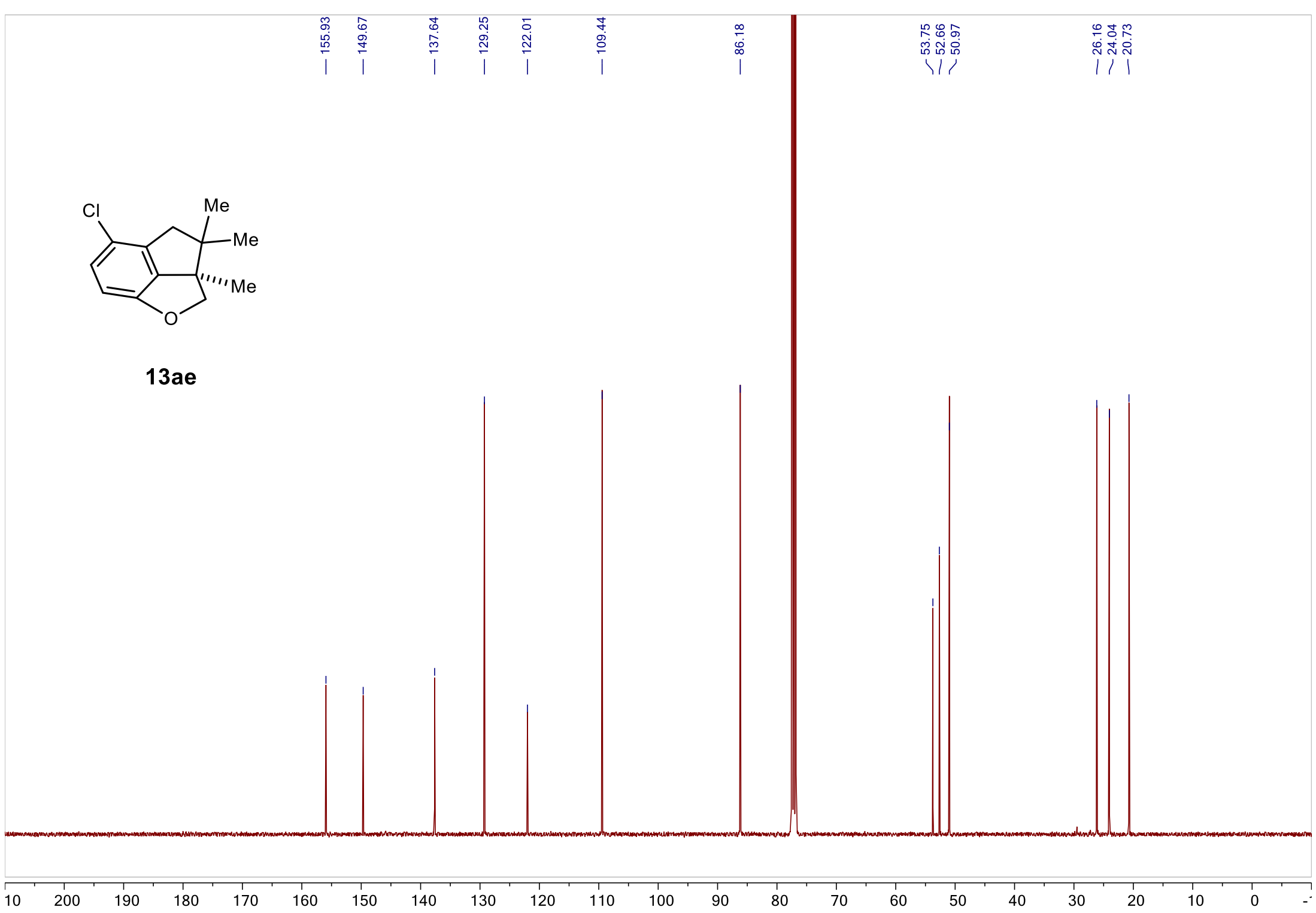

$^{1}H$ NMR (400 MHz, $CDCl_3$)

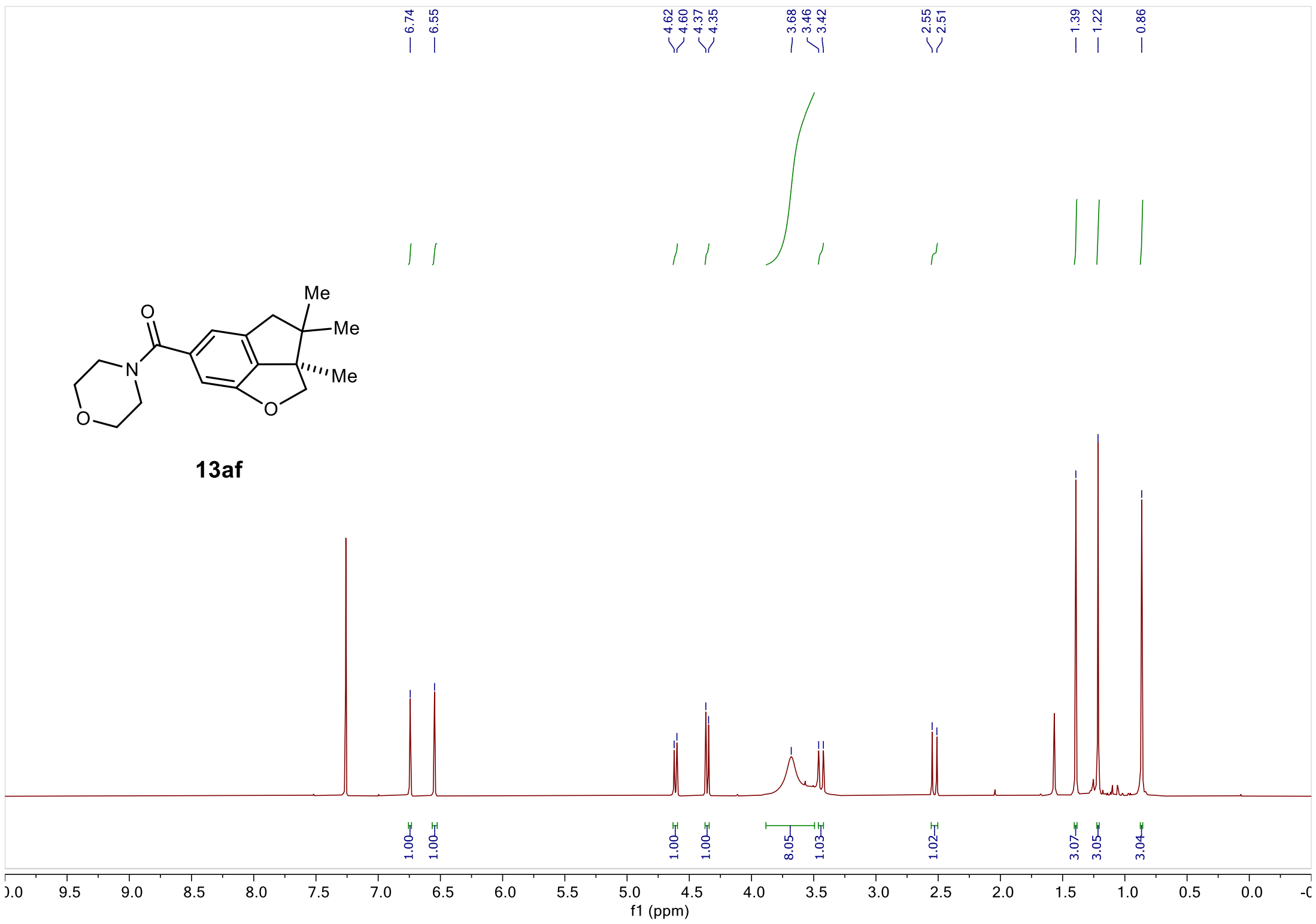


$^{13}C$ NMR (101 MHz, $CDCl_3$)

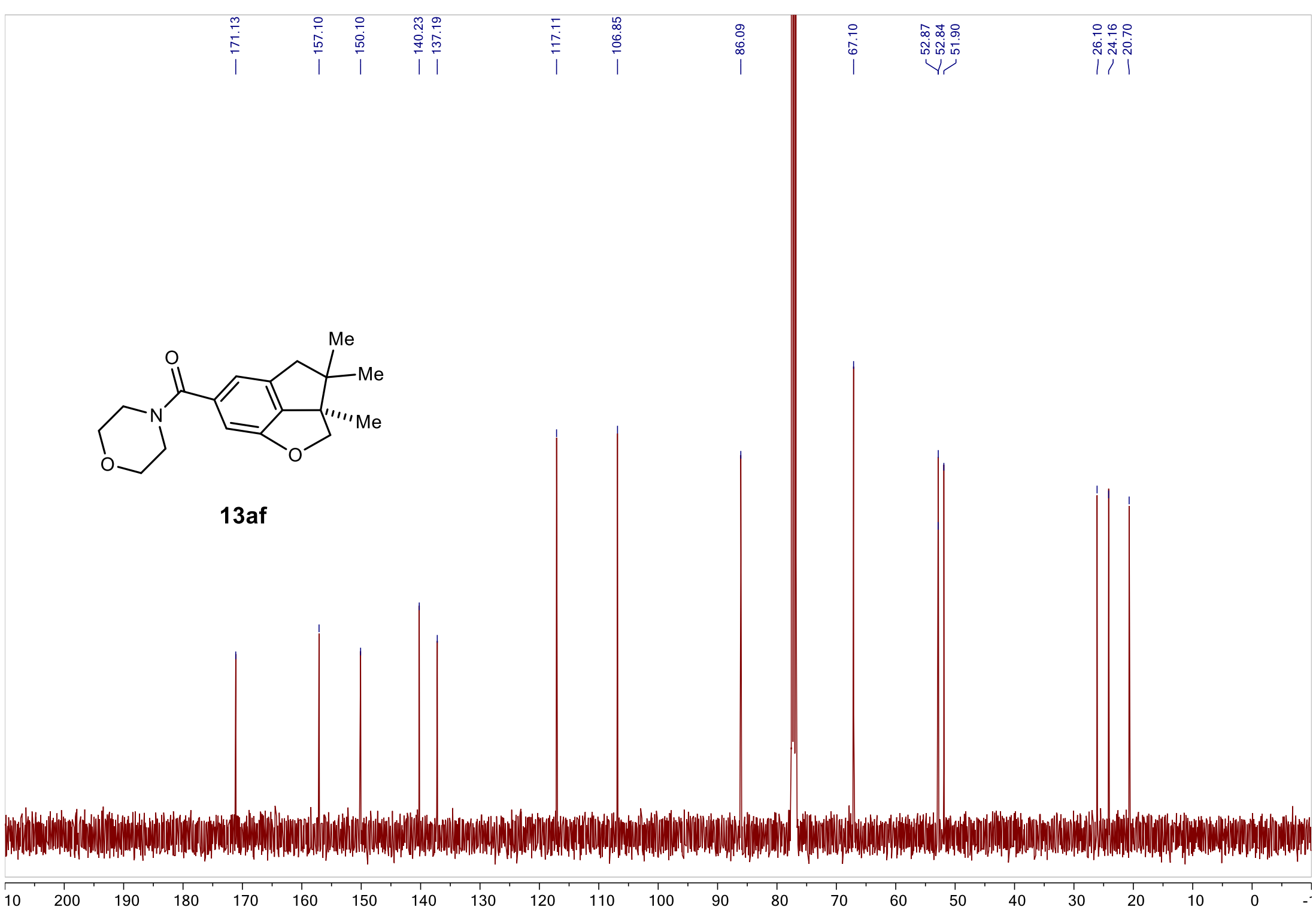

$^{1}$H NMR (400 MHz, $CDCl_3$)

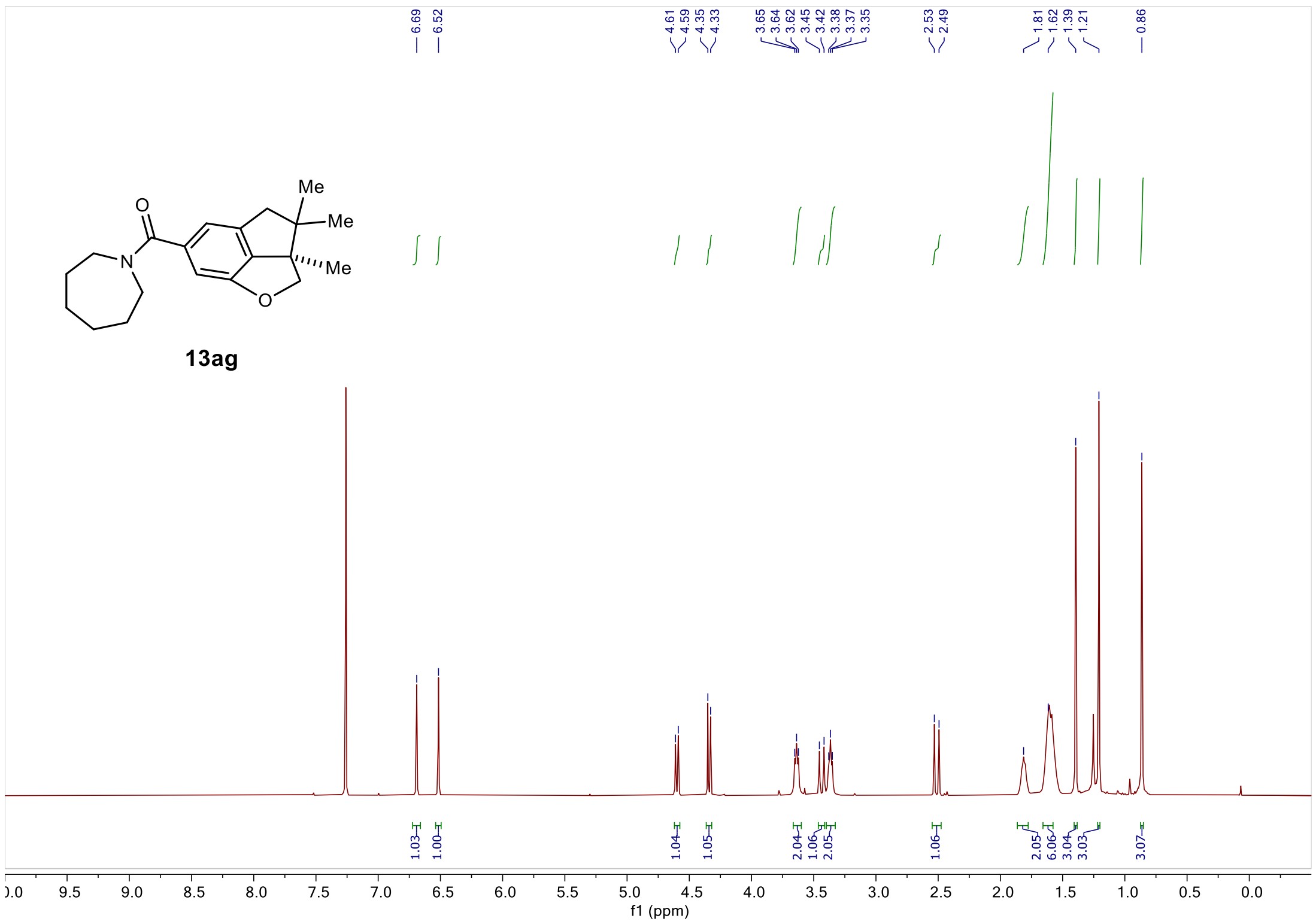


$^{13}$C NMR (101 MHz, $CDCl_3$)

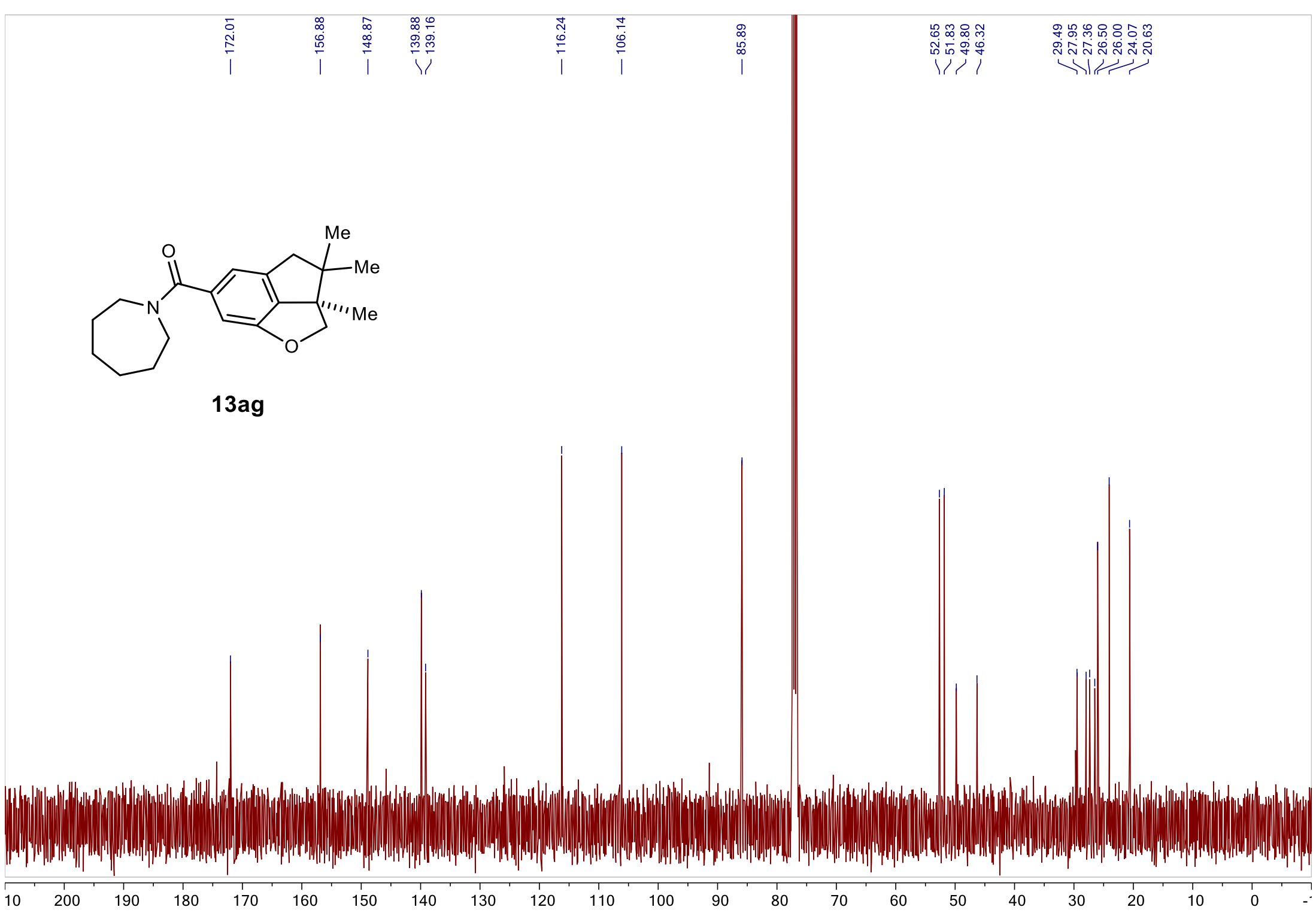

$^{1}$H NMR (400 MHz, $CDCl_3$)

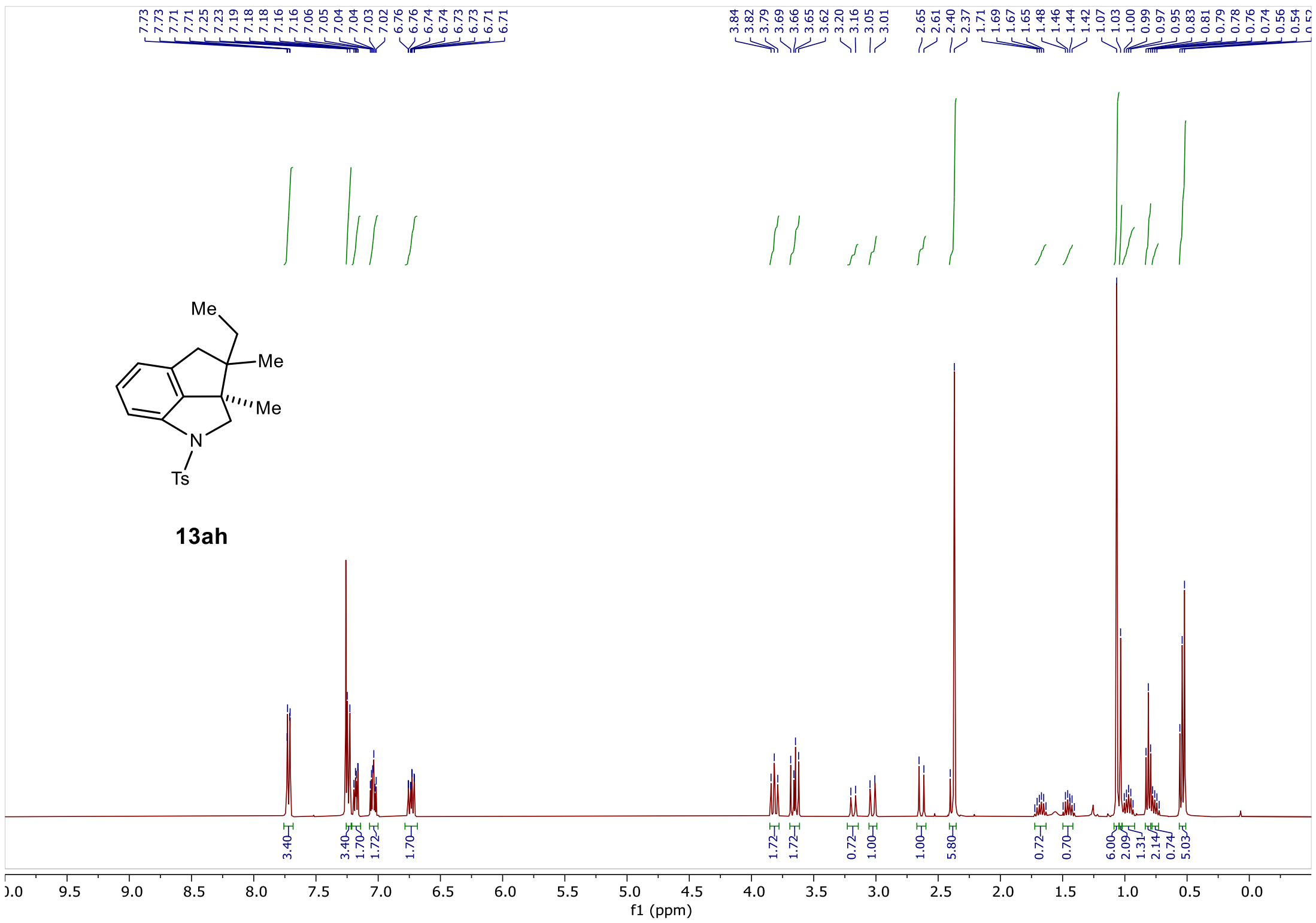

$^{13}$C NMR (101 MHz, $CDCl_3$)

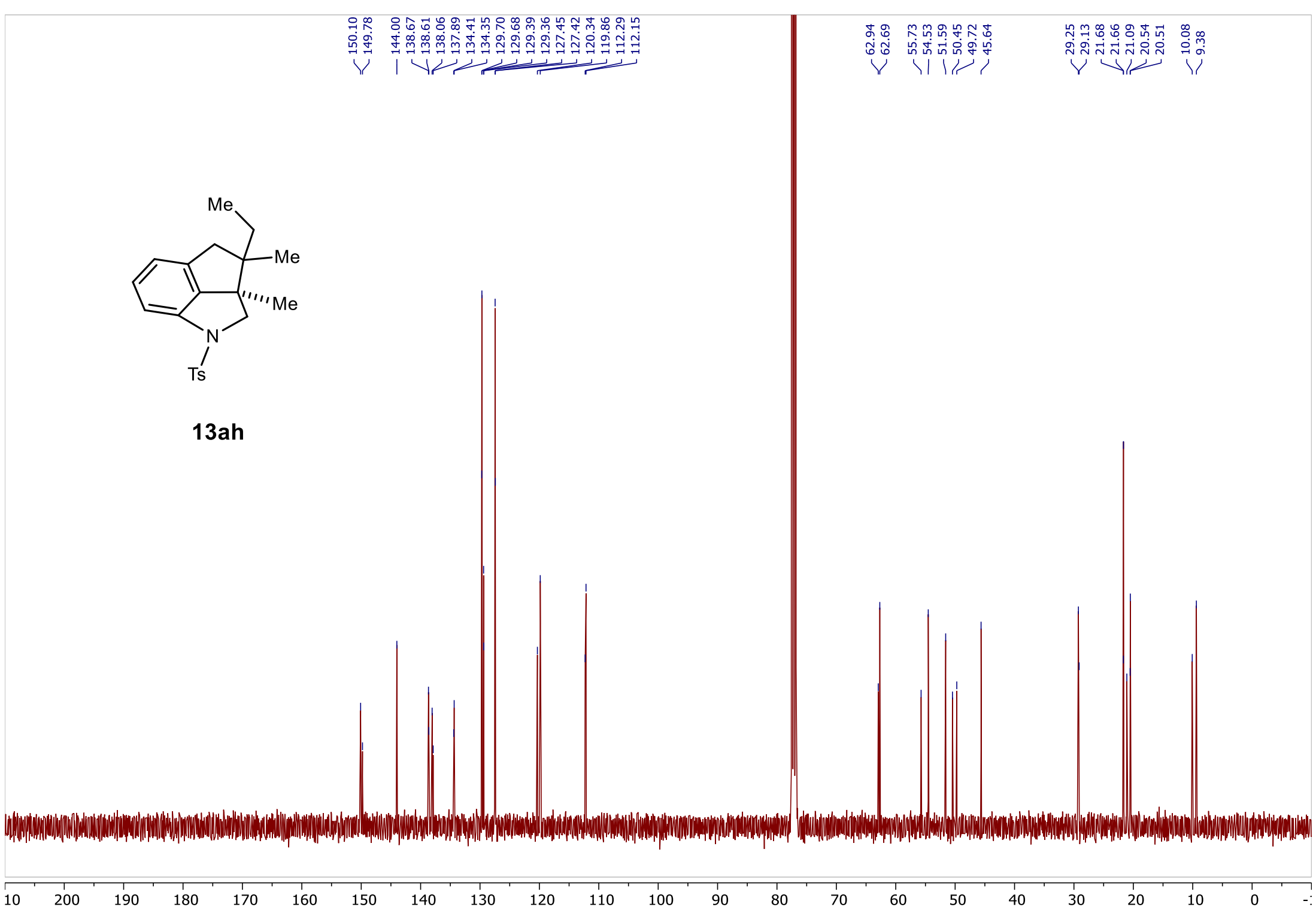

$^{1}H$ NMR (400 MHz, $CDCl_3$)

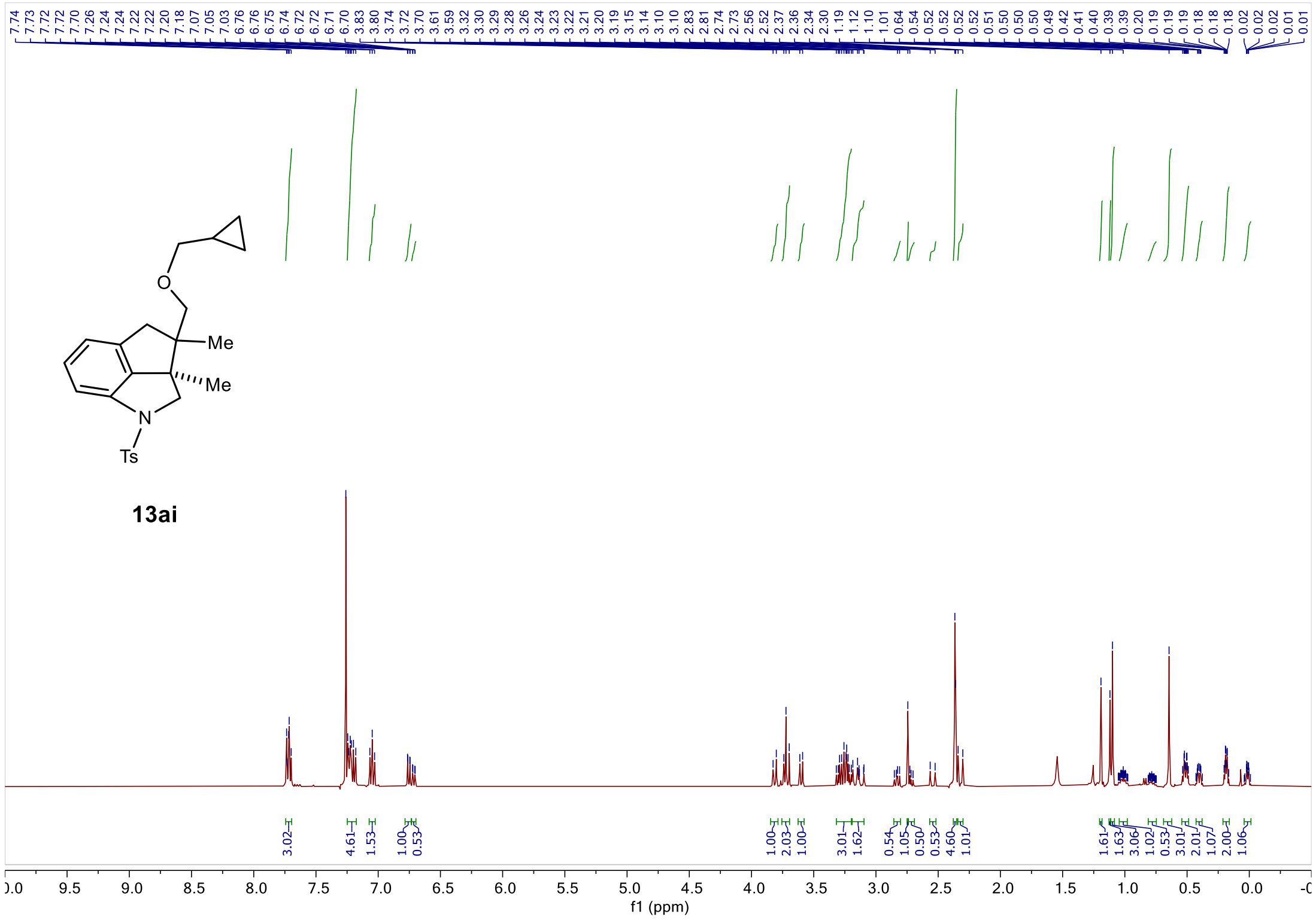

$^{13}C$ NMR (101 MHz, $CDCl_3$)

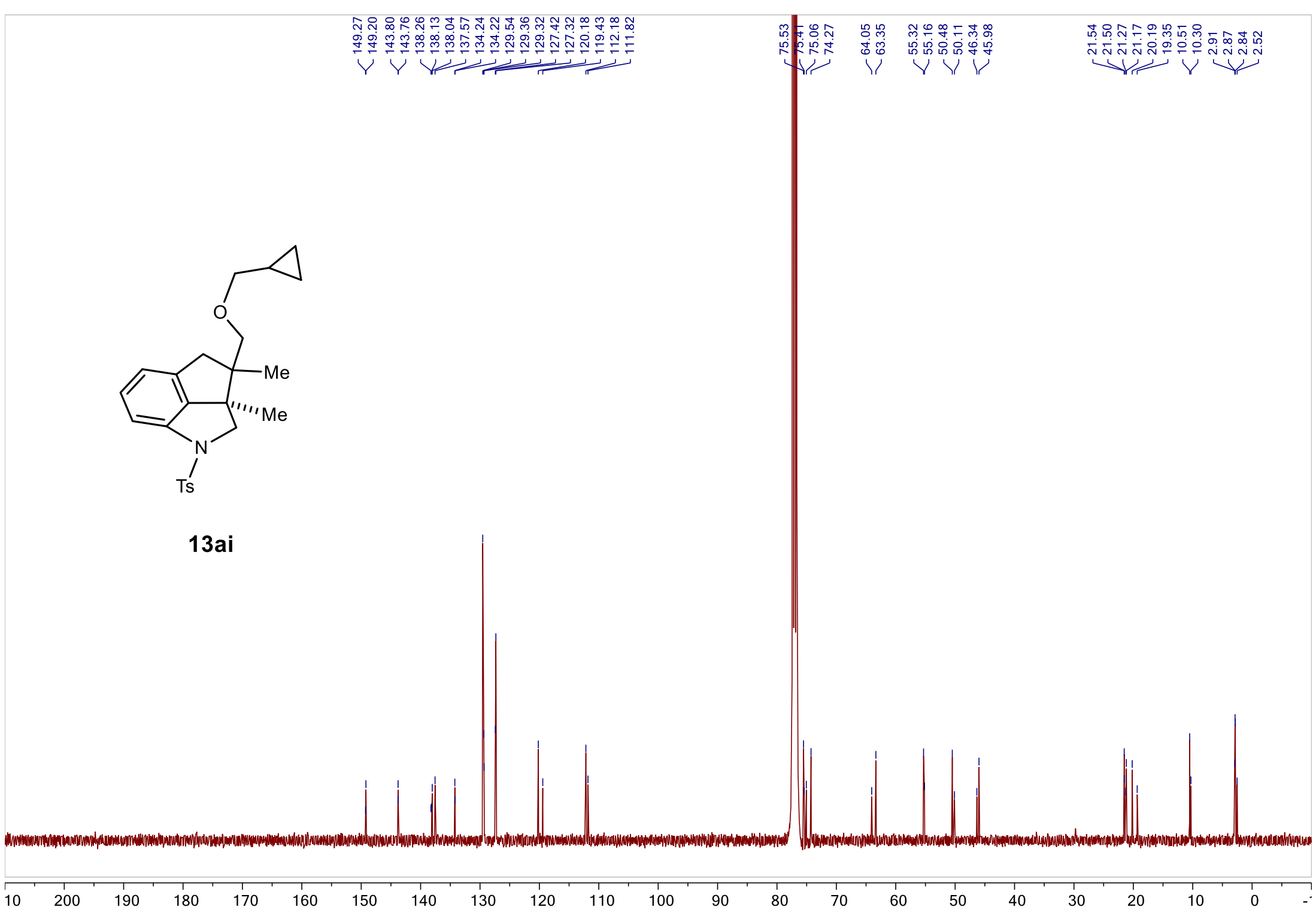

$^{1}H$ NMR (400 MHz, $CDCl_3$)

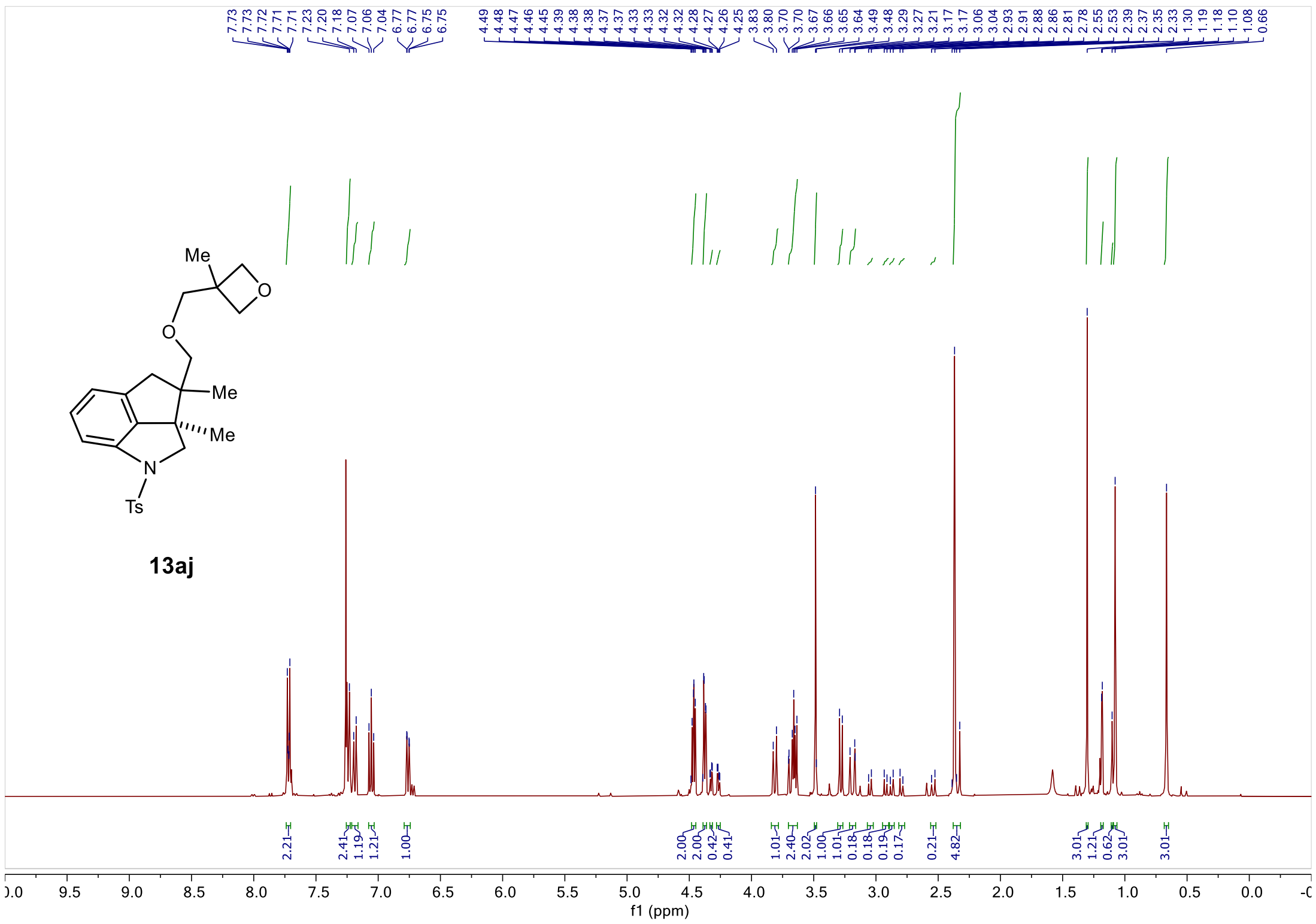


$^{13}C$ NMR (101 MHz, $CDCl_3$)

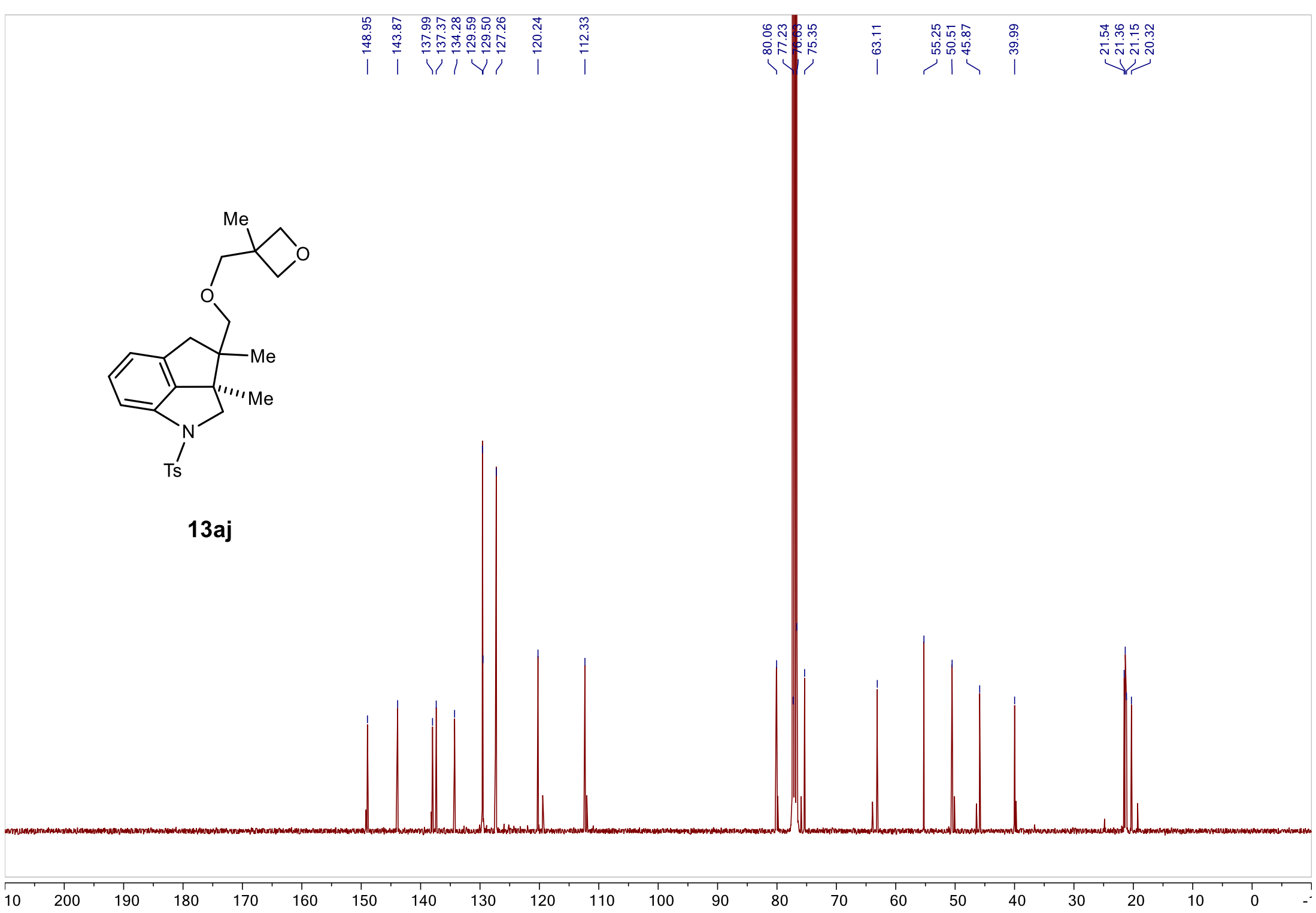

$^{1}H$ NMR (500 MHz, $CDCl_3$)

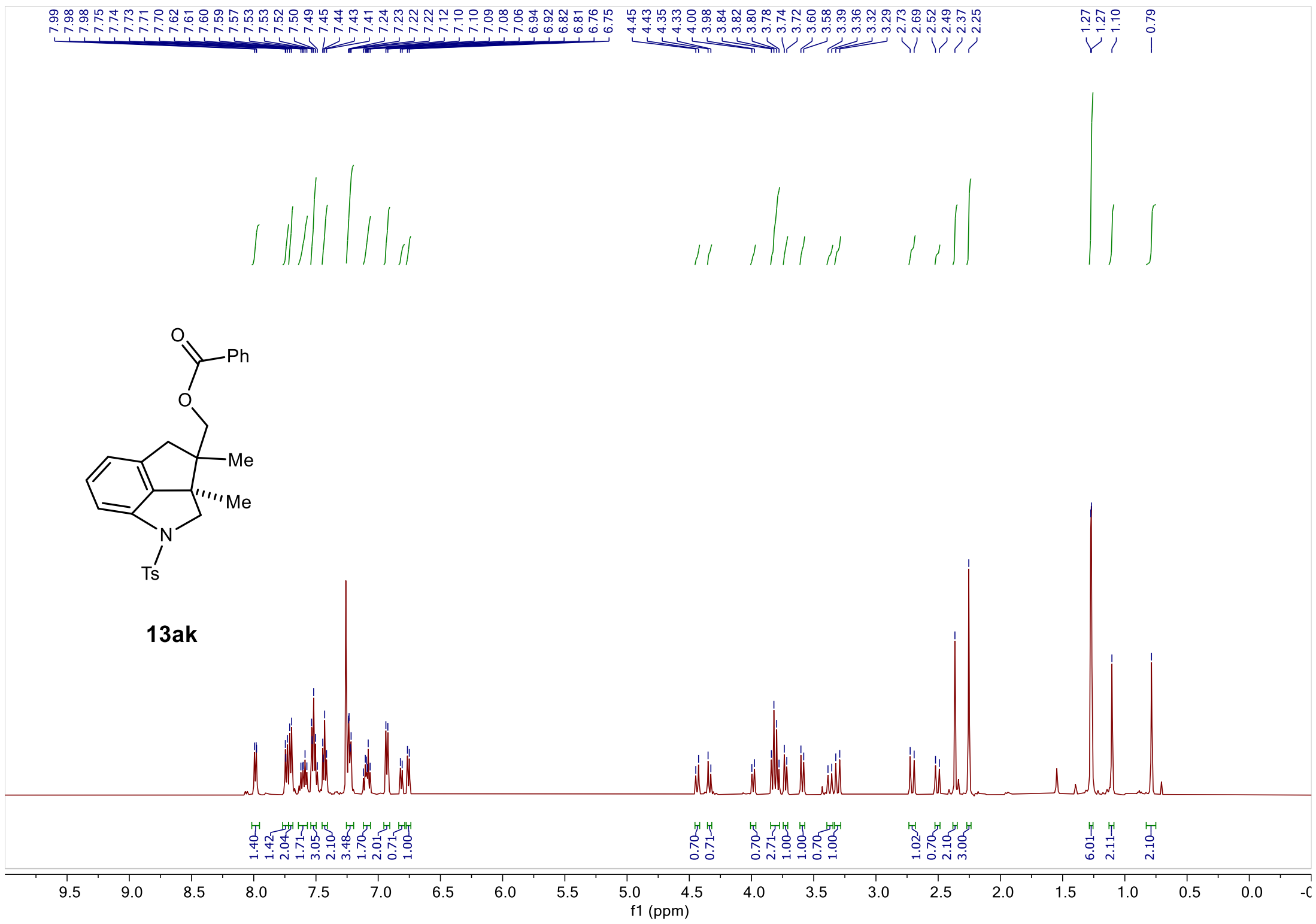


$^{13}C$ NMR (101 MHz, $CDCl_3$)

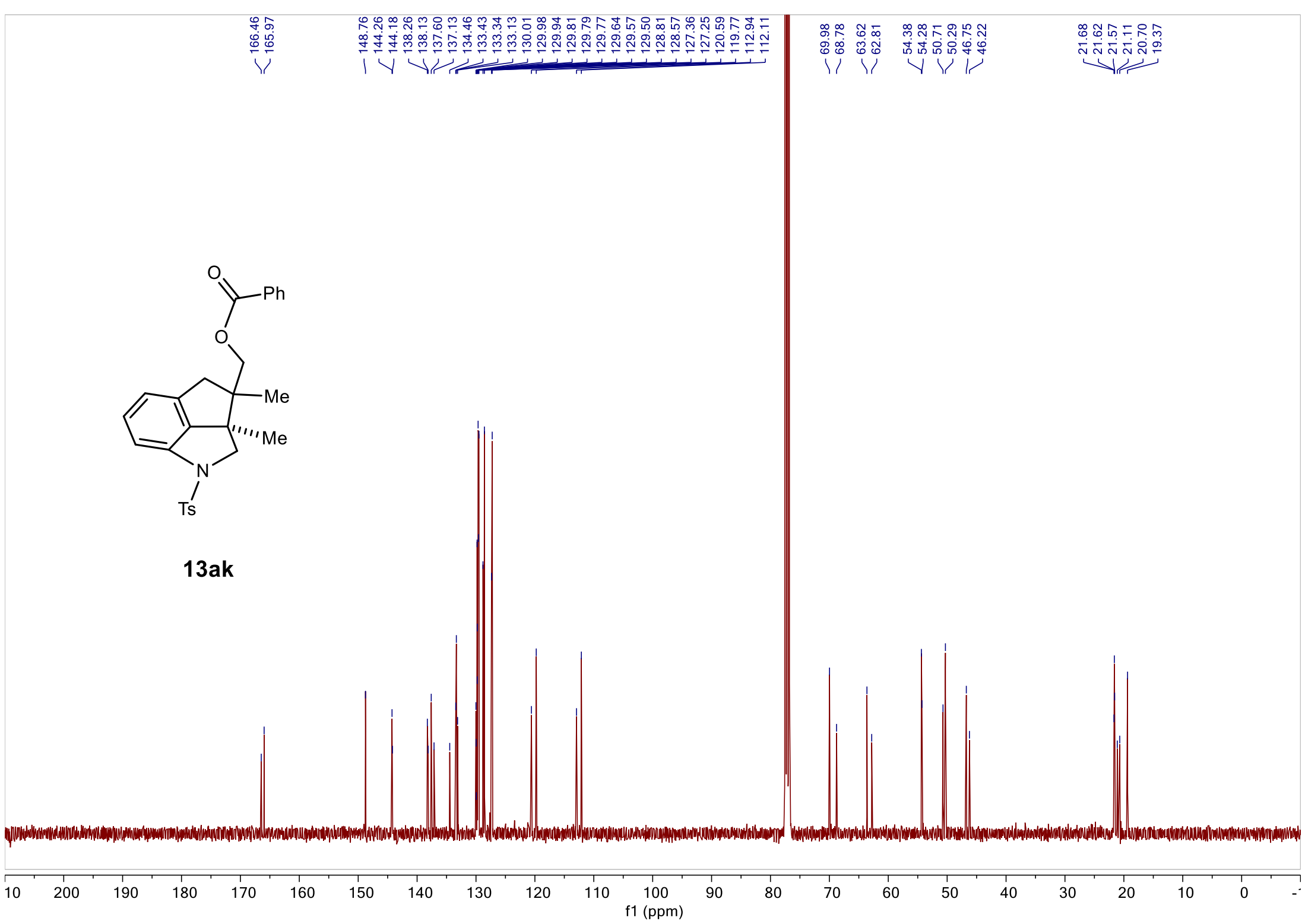

$^{1}H$ NMR (500 MHz, $CDCl_3$)

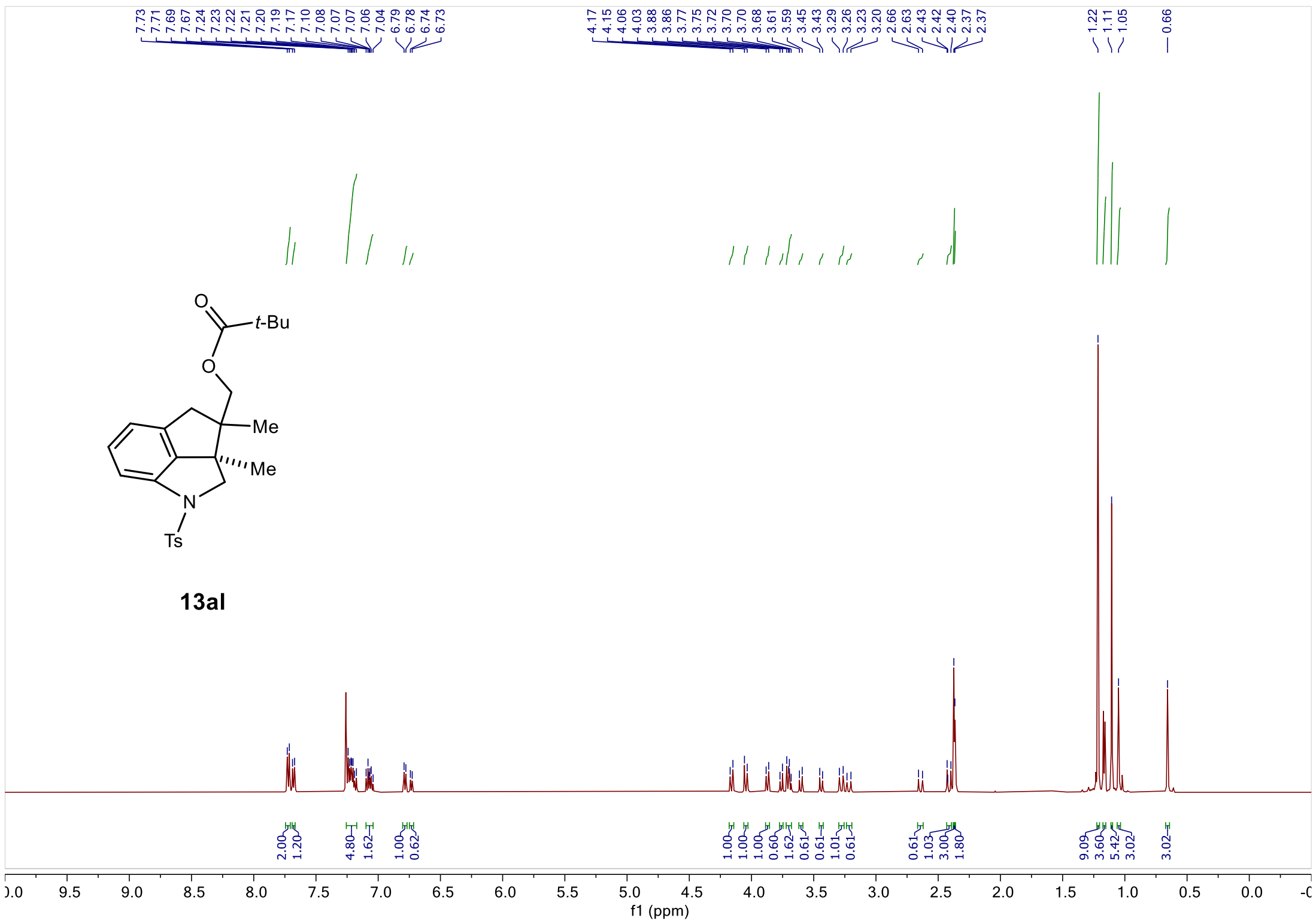

$^{13}C$ NMR (101 MHz, $CDCl_3$)

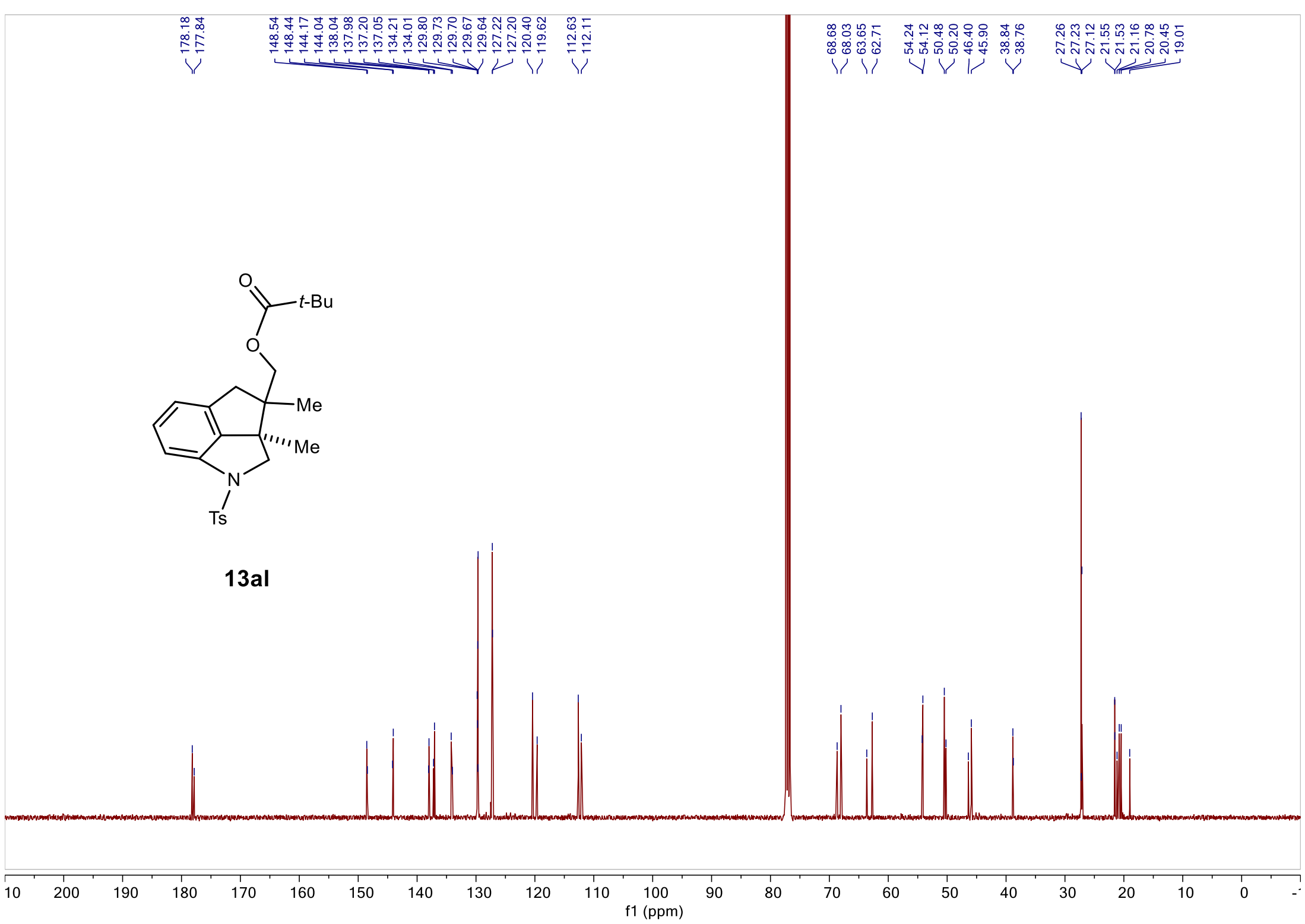

$^{1}H$ NMR (400 MHz, $CDCl_3$)

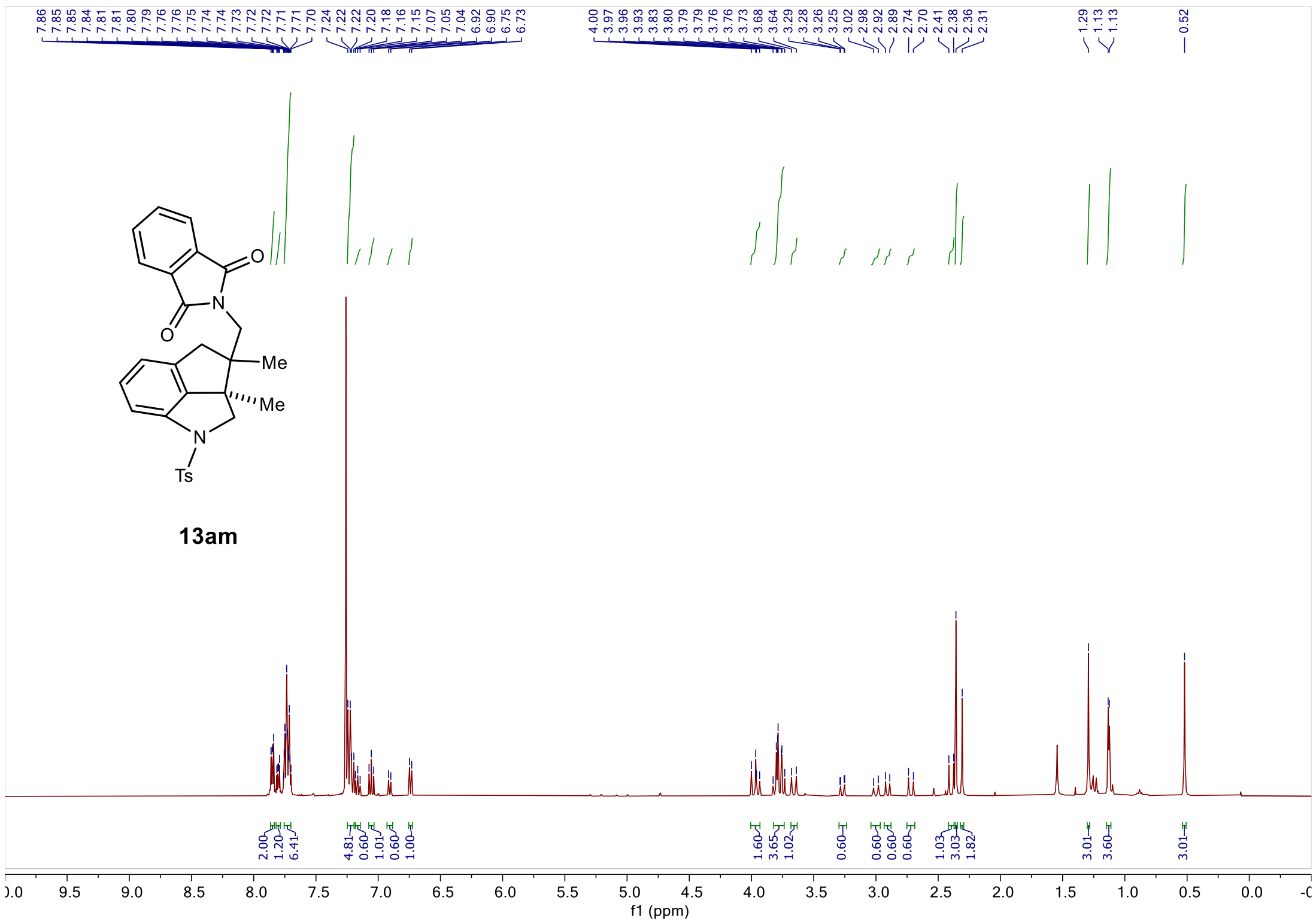


$^{13}C$ NMR (101 MHz, $CDCl_3$)

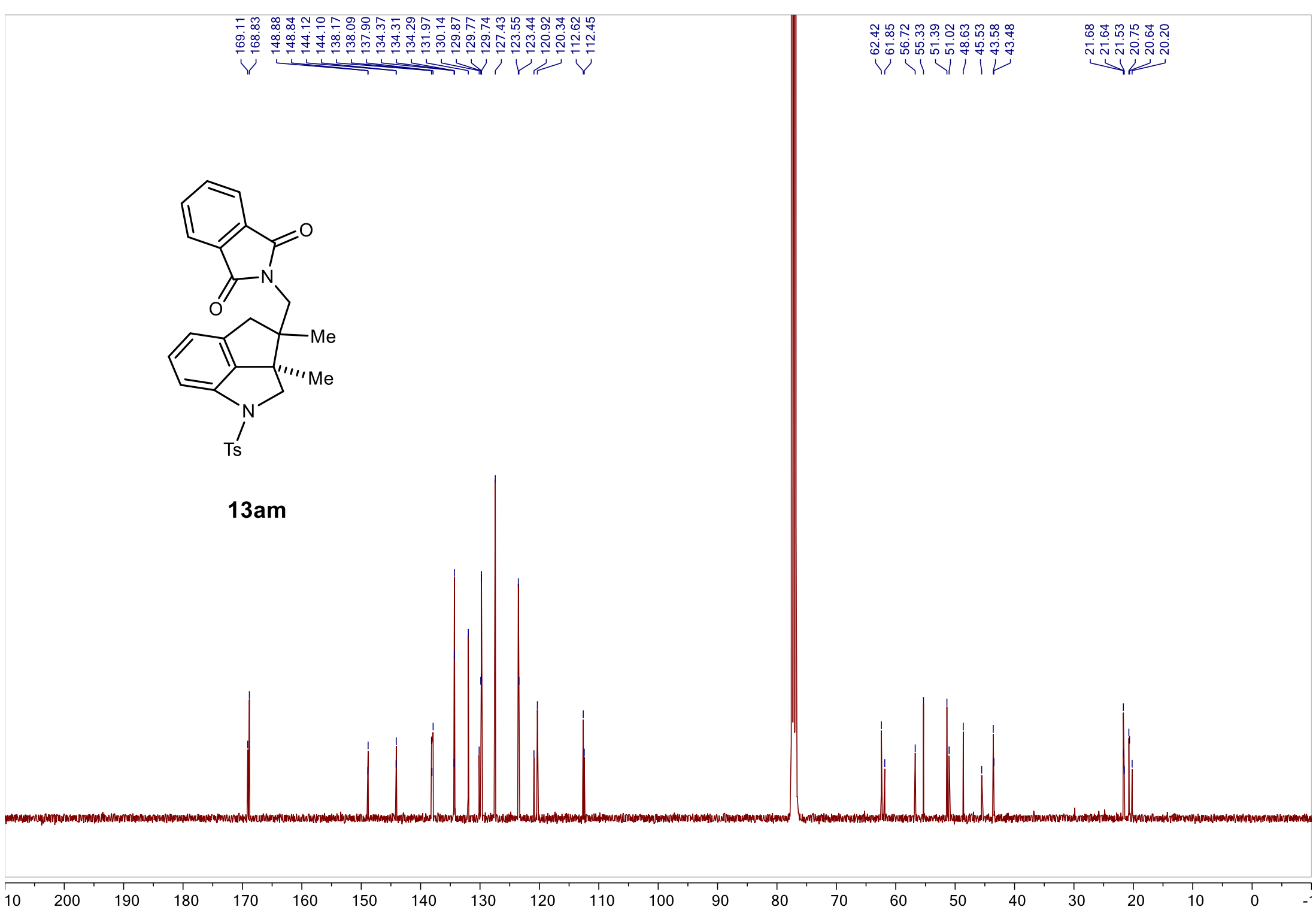

$^{1}H$ NMR (400 MHz, $CDCl_3$)

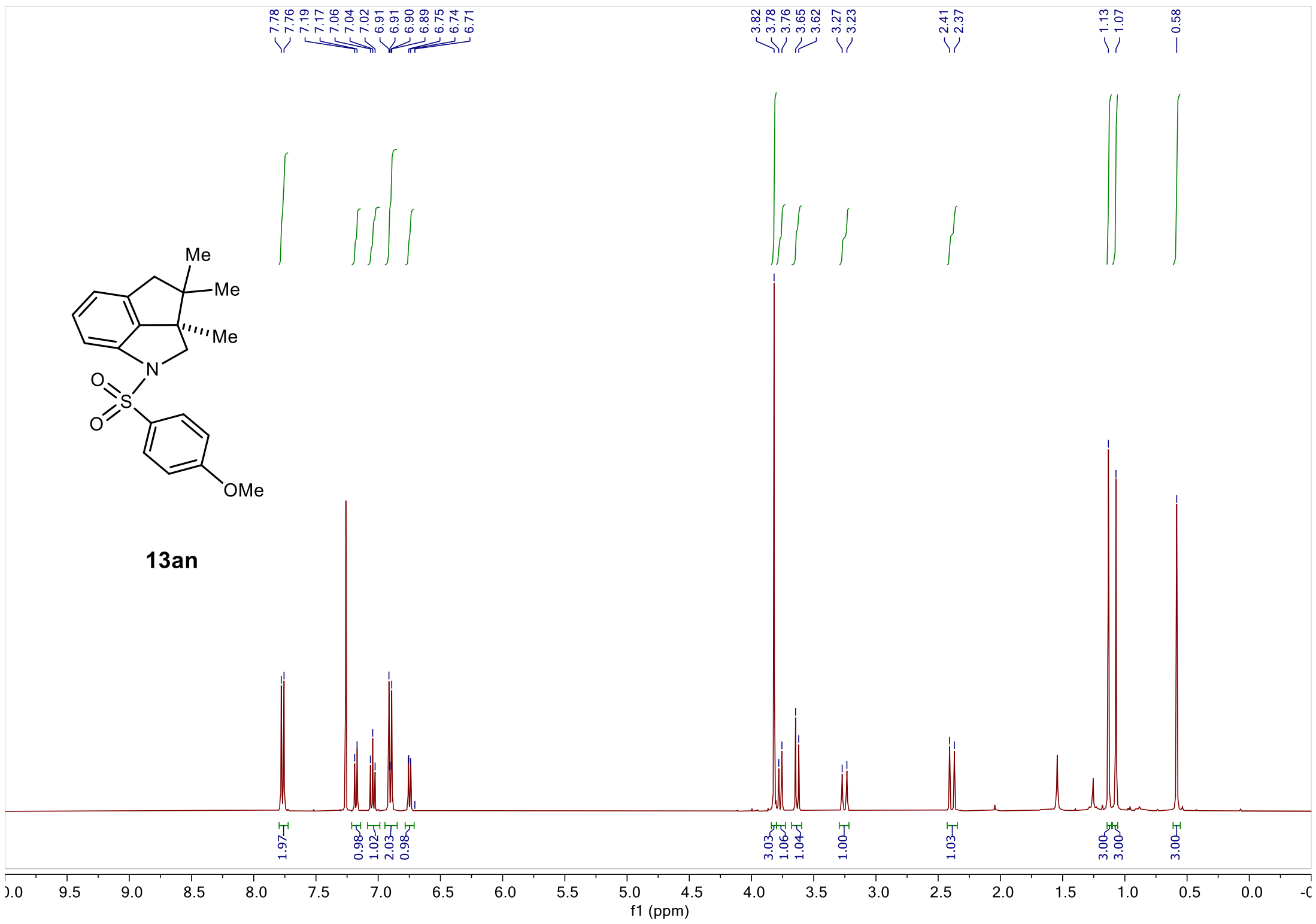

13an

$^{13}C$ NMR (101 MHz, $CDCl_3$)

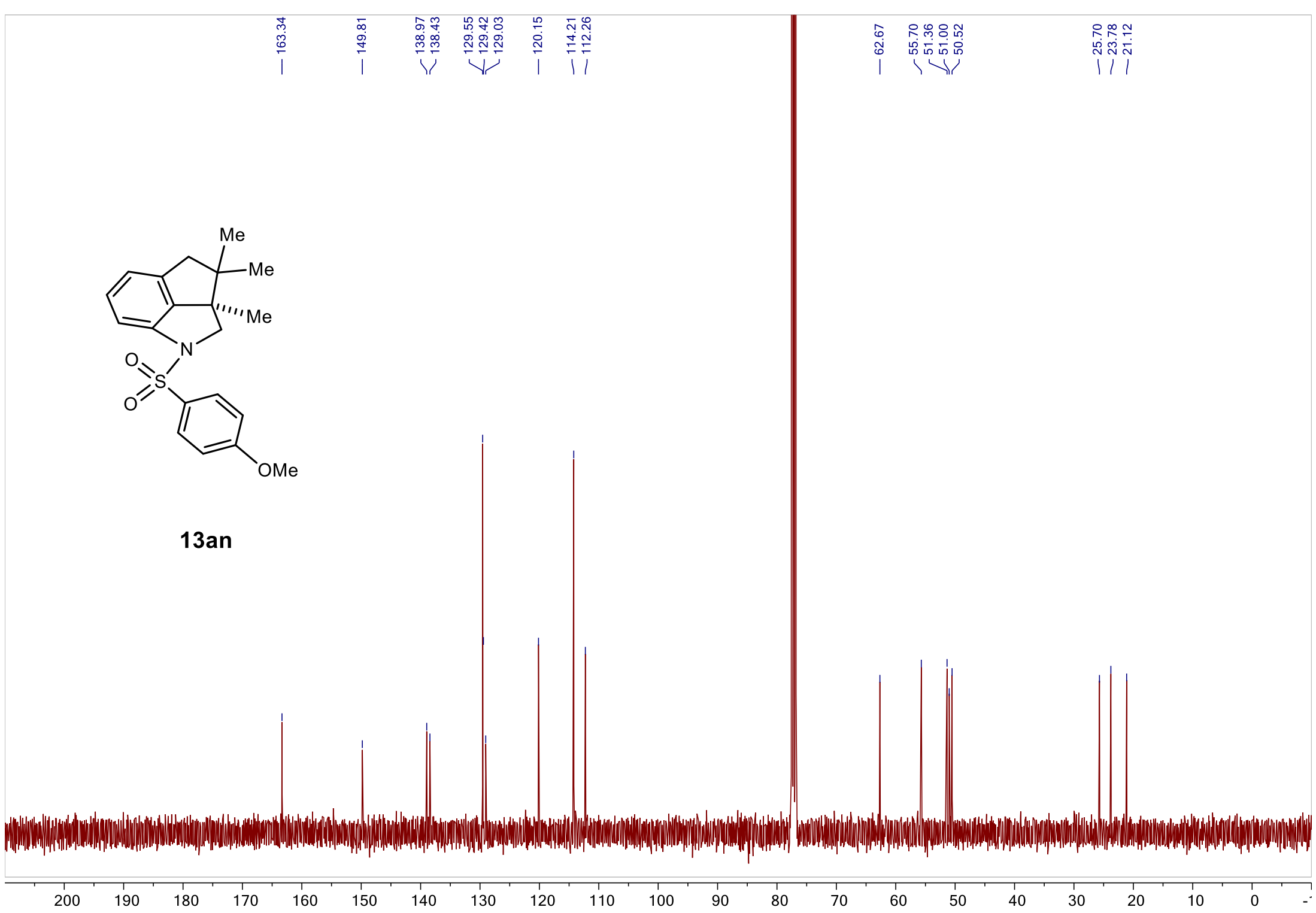

13an

$^{1}H$ NMR (400 MHz, $CDCl_3$)

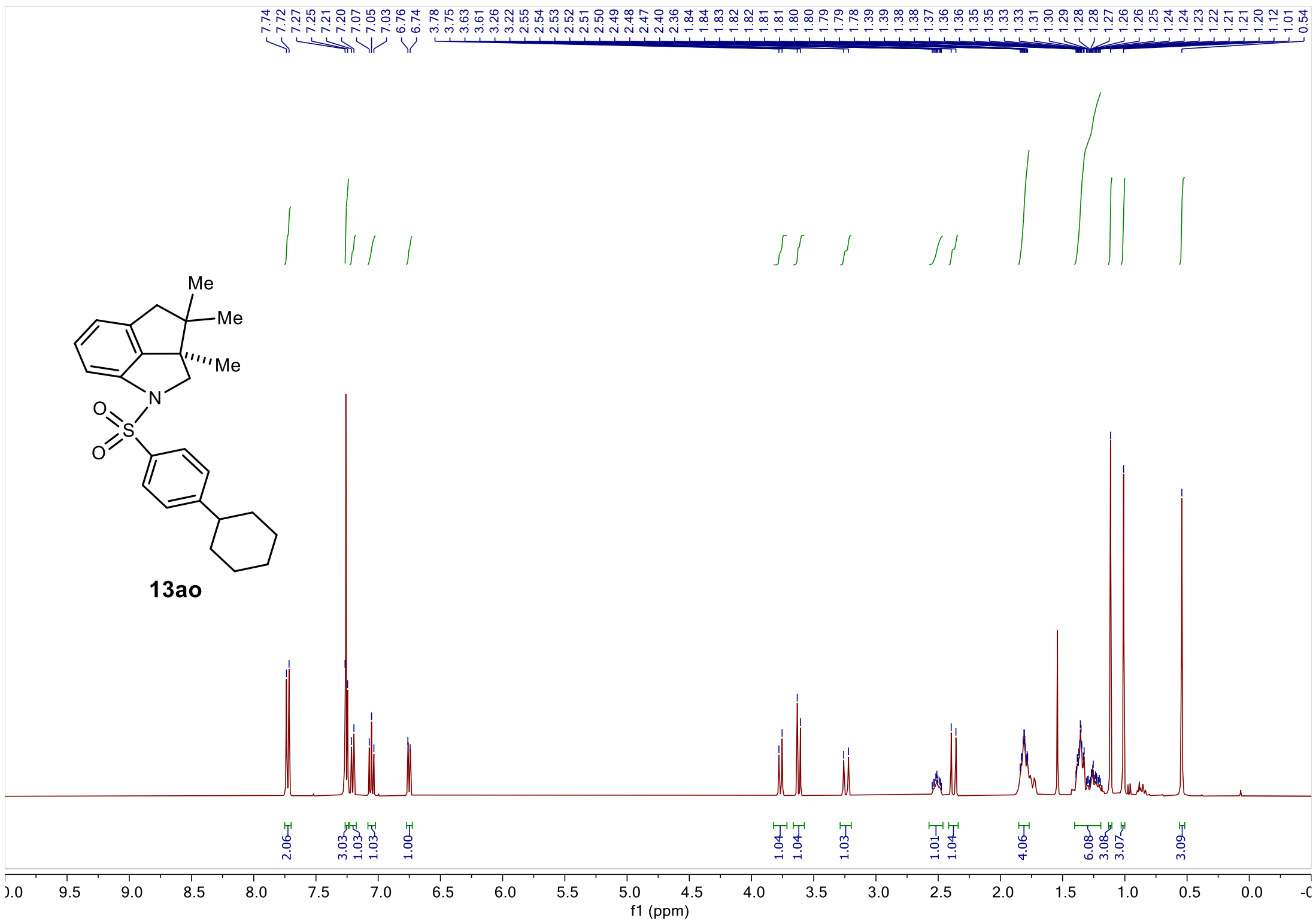


$^{13}C$ NMR (101 MHz, $CDCl_3$)

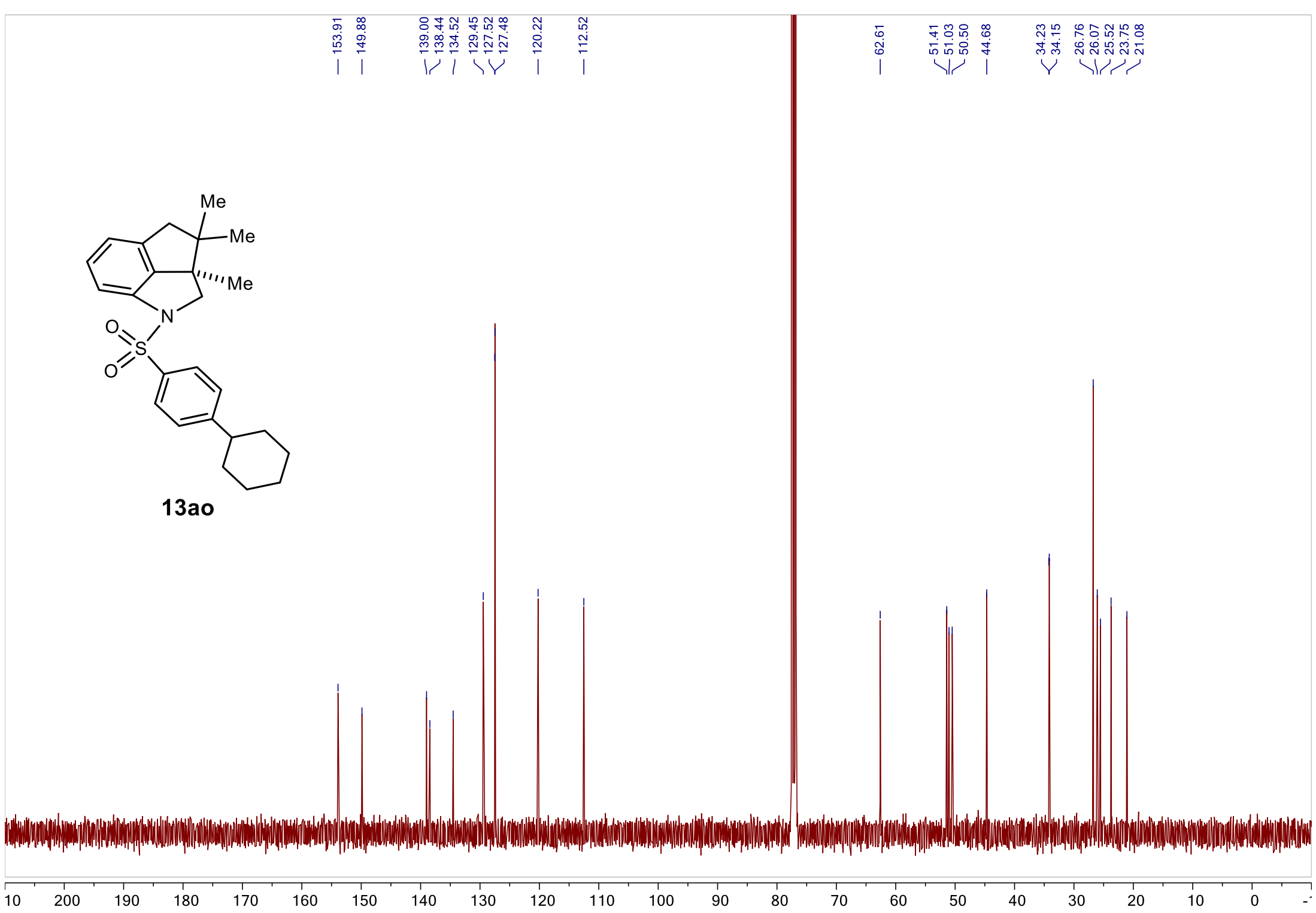

$^{1}H$ NMR (500 MHz, $CDCl_3$)

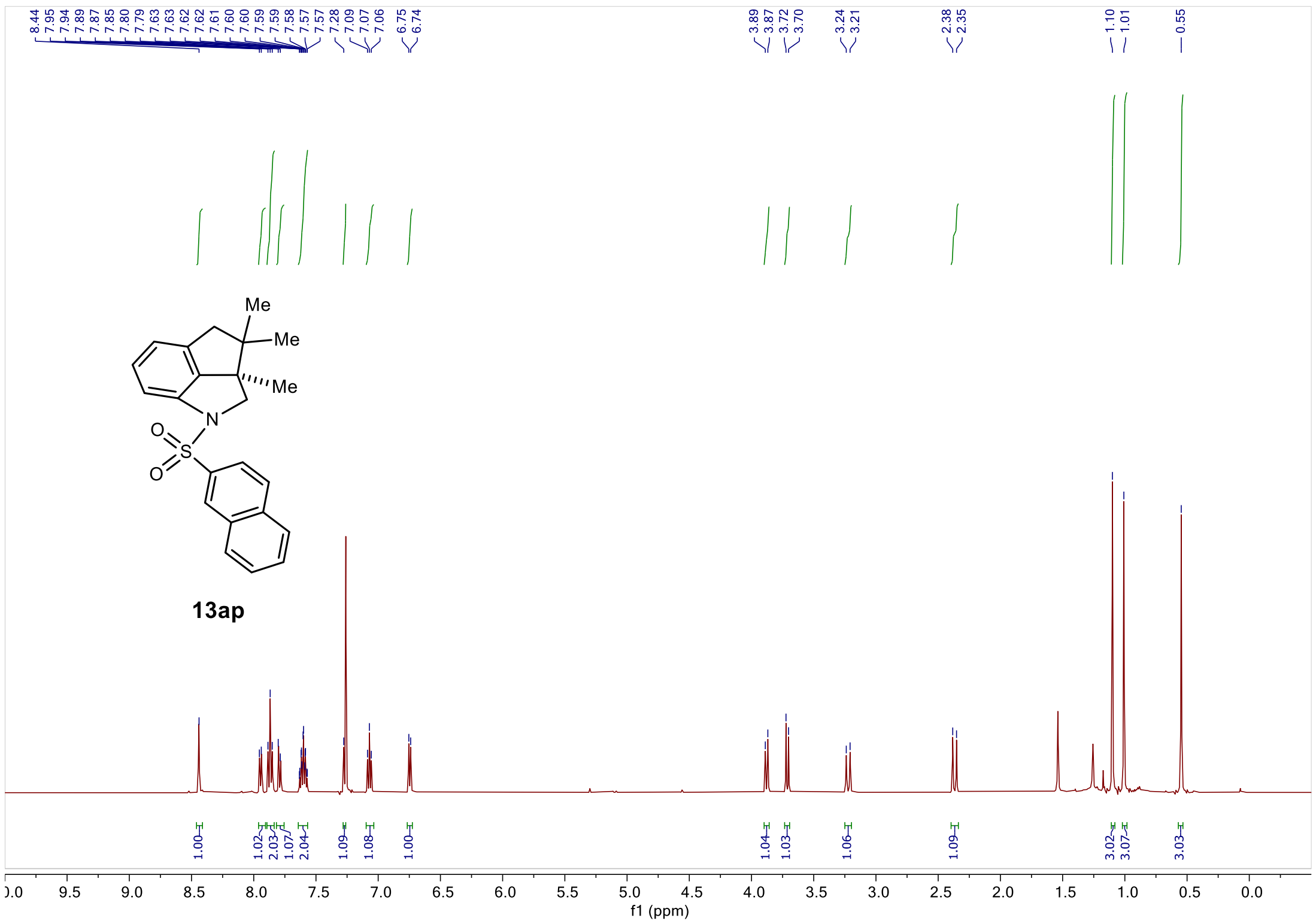


$^{13}C$ NMR (126 MHz, $CDCl_3$)

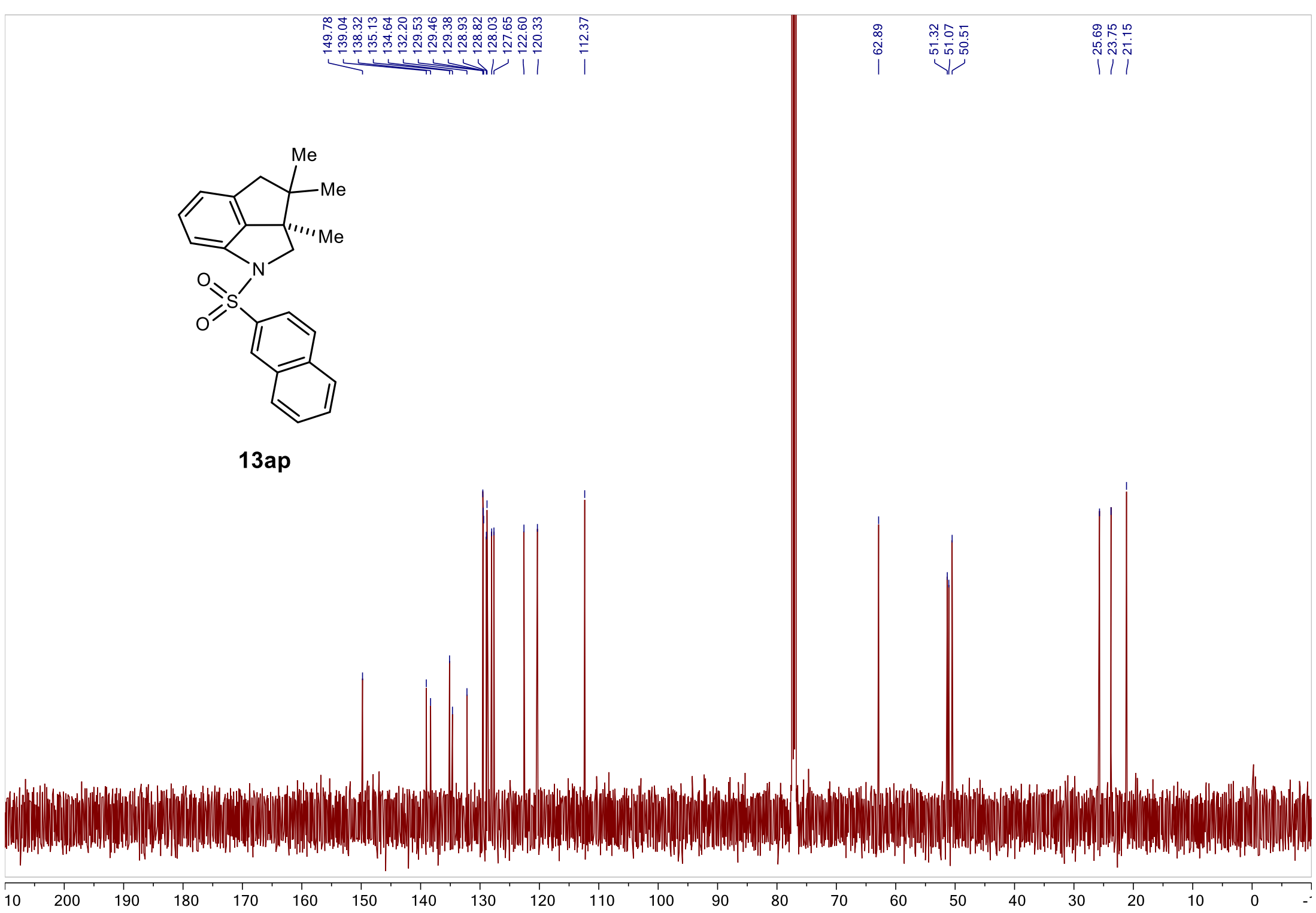

$^{1}H$ NMR (400 MHz, $CDCl_3$)

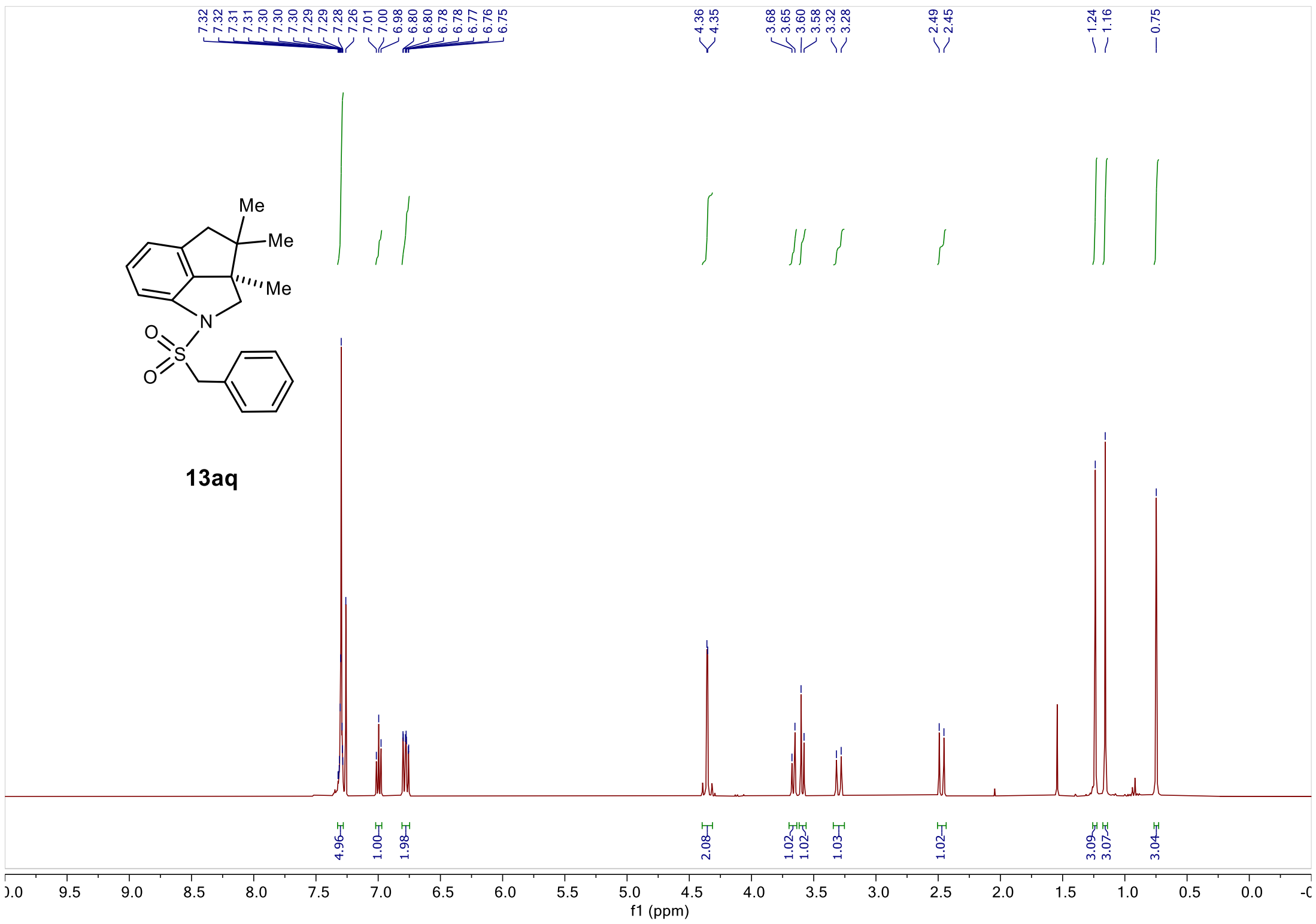


$^{13}C$ NMR (101 MHz, $CDCl_3$)

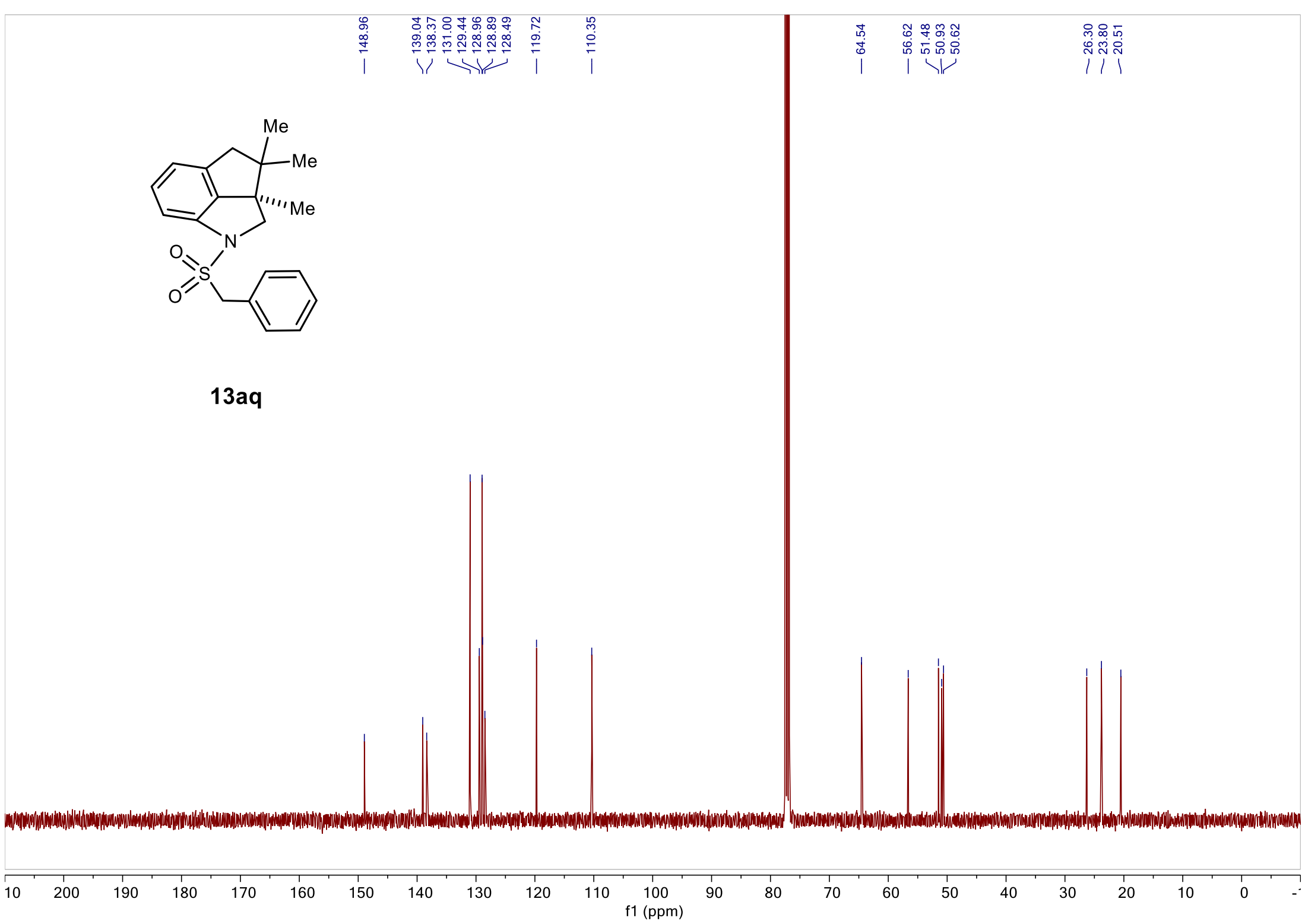

$^{1}$H NMR (400 MHz, $CDCl_3$)

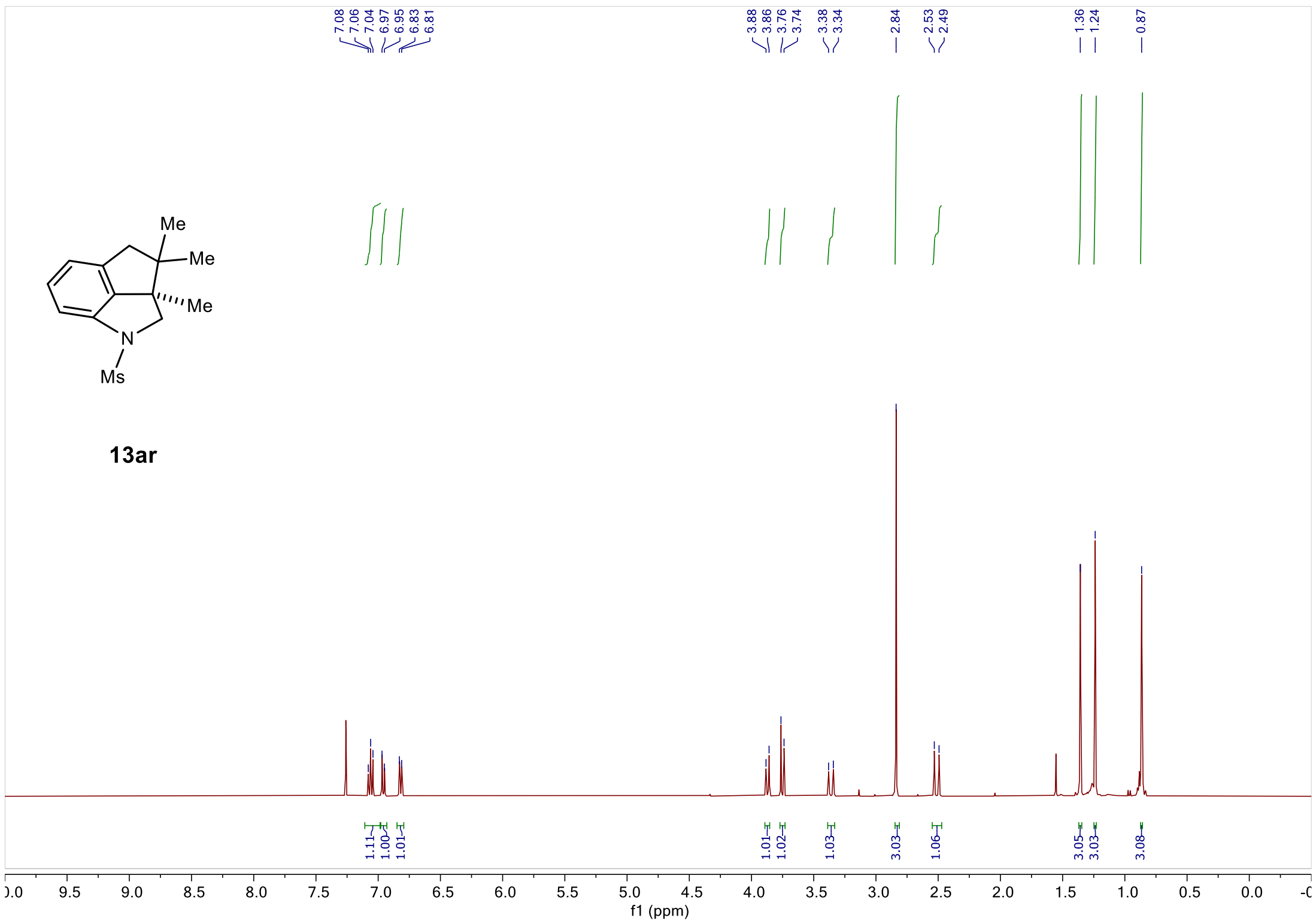


$^{13}$C NMR (101 MHz, $CDCl_3$)

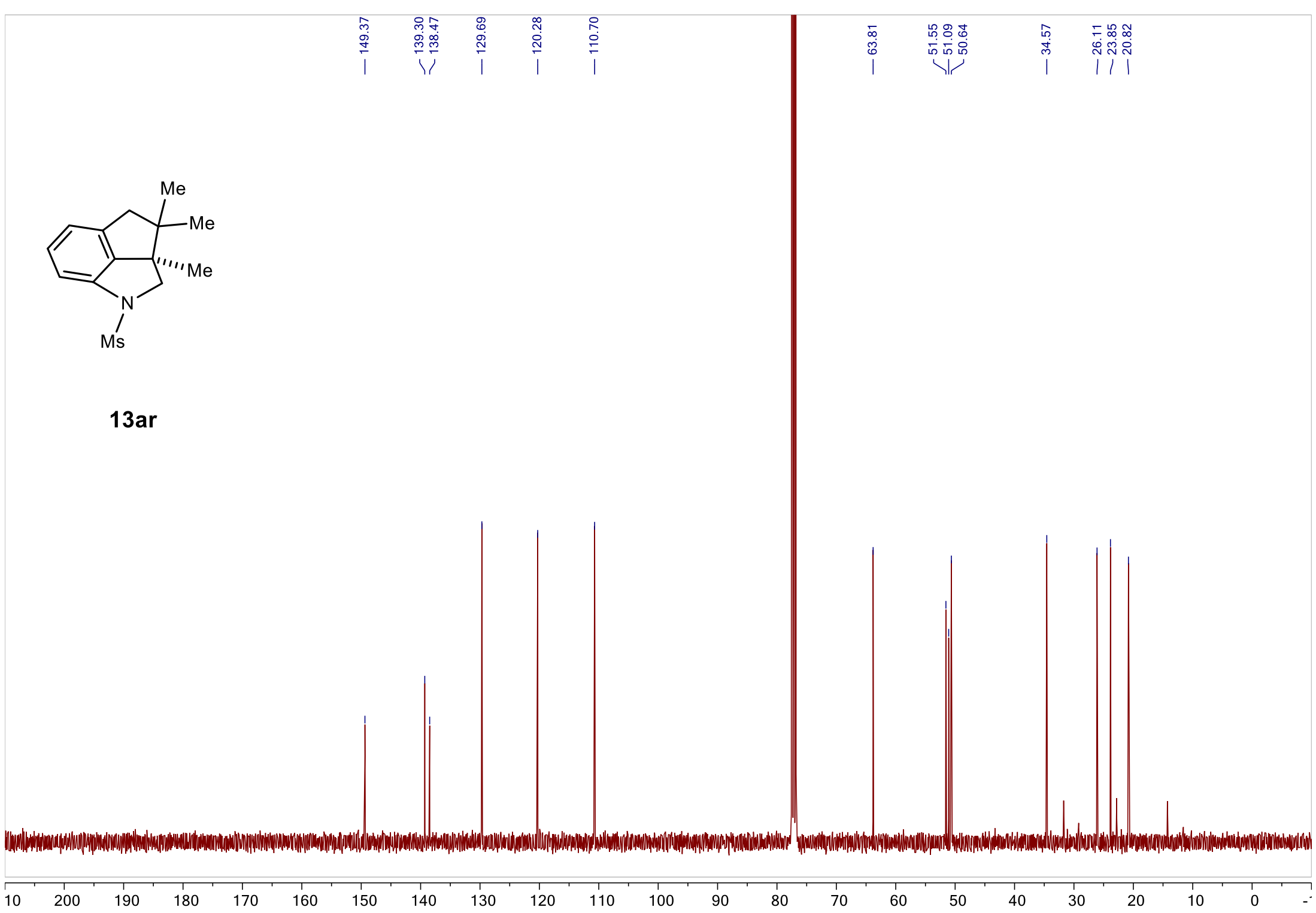

$^{1}$H NMR (400 MHz, $CDCl_3$)

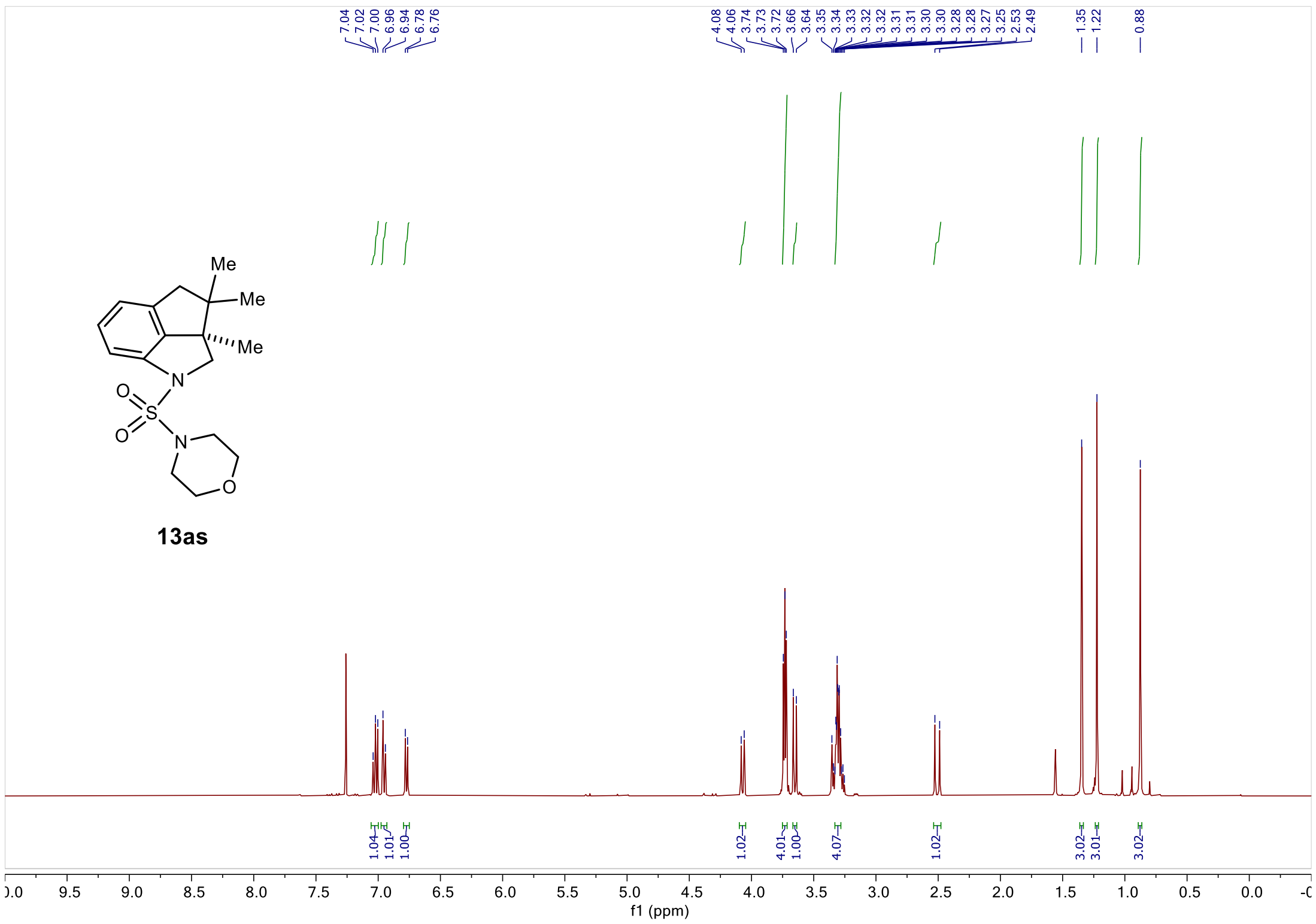


$^{13}$C NMR (101 MHz, $CDCl_3$)

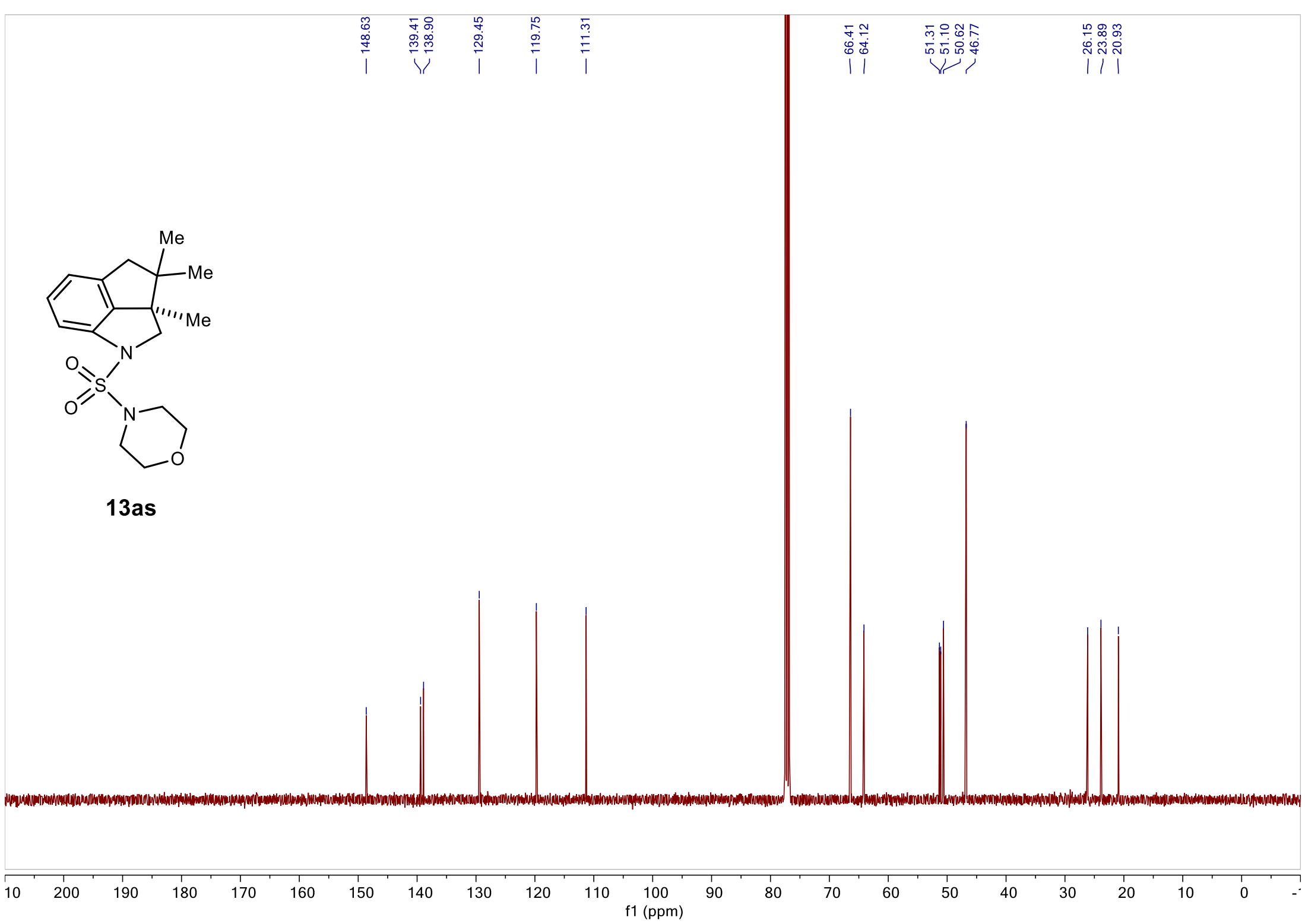

Supplement: Supplementary file 1 [file ja6c12509_si_001.pdf]
